# Supplementary material for: Functional Innovation in the Evolution of the Calcium-Dependent System of the Eukaryotic Endoplasmic Reticulum
Source: Front Genet. 2020 Feb 6;11:34. doi: 10.3389/fgene.2020.00034 (PMC7016017; doi:10.3389/fgene.2020.00034)
Supplement: Supplementary file 1 [file DataSheet_1.pdf]

Functional innovation in the evolution of the calcium-dependent system of the eukaryotic endoplasmic reticulum

Supplementary Material

Daniel E. Schäffer[1,2], Lakshminarayan M. Iyer[1], Alexander M. Burroughs[1], L Aravind*[1]

1-National Center for Biotechnology Information, National Library of Medicine, National Institutes of Health, Bethesda, MD 20894, USA

2-Montgomery Blair High School, Science, Mathematics, and Computer Science Magnet Program, Silver Spring, MD 20901, USA

*-corresponding authors

*To whom correspondence should be addressed: L Aravind: Building 38A, Room 5N503, 8600 Rockville Pike, Bethesda, MD 20894 USA; aravind@ncbi.nlm.nih.gov; Tel. (301)594-2445; Fax. (301)435-7794.

Table of Contents

I. Supplementary Figure Legends
II. Supplementary Table S1.
III. Supplementary Table S2.
IV. Supplementary Table S3.
V. Supplementary Table S4.
VI. Supplementary Figure S1.
VII. Supplementary Figure S2.
VIII. Supplementary Figure S3.
IX. Supplementary Figure S4.
X. Supplementary Data.

I. Supplementary Figure Legends

Supplementary Figure S1. Summary of evolutionary analysis of representative proteins involved in calcium signaling, including wolframin. The figure shows a matrix showing the presence (P / green) or absence (A / red) of homologs of 34 ER-calcium-signaling-related proteins in 41 extant taxonomic groups. Additionally, inferred presence/absence patters are shown for five key ancestral states, denoted in **bold**. Each row represents one protein or protein family, and each column represents one eukaryotic taxonomic group, sorted left-to-right by approximate time of divergence from the most recent ancestor shared with Vertebrata. 'Ties' among the columns are broken arbitrarily with some effort made to put related groups close to each other. The rightmost (i.e. most basal) "P" for each protein is bolded. The proteins are sorted top to bottom in approximate order of evolutionary occurrence, as determined by the most basal group in which a homolog of that protein was identified. The "Accession Used" column gives the RefSeq accession of the specific sequence used in the searches used to generate that row (see Methods).

Supplementary Figure S2. Six examples of conserved gene neighborhoods showing the associations between bacterial P-type ATPases and other proteins involved in ion homeostasis.

Supplementary Figure S3. Multiple-sequence alignment of the transmembrane region of wolframin. The transmembrane helices (as reported in Qian et al., 2015) are indicated by red cylinders above the alignment. Sequences are identified on the left by organism abbreviation (see Supplementary Table S3) and NCBI GenBank accession number, separated by two underscores. The alignment is colored as follows: h (hydrophobic), l (aliphatic), and a (aromatic) are shown on a yellow background; o (alcohol) is shown in salmon font; p (polar) is shown in blue font; + (positively charged), − (negatively charged), and c (charged) are shown in pink font; s (small) is shown in green font; u (tiny) is shown on a green background; b (big) is shaded gray. Columns in which the same amino acid is in at least 9 out of the 10 sequences are denoted with that amino acid's one-letter abbreviation in the consensus row and are colored based on a category to which that amino acid belongs.

Supplementary Figure S4. Renderings ('pepwheels') of amino acid positions on the circumference of wolframin's transmembrane helices. Each subfigure corresponds to one of wolframin's nine TM helices; the positions in human wolframin are indicated above

the figure. The first amino acid is at the top, and the amino acids move clockwise at a rate of 18 amino acids per five rotations. Hydrophilic amino acids are in red.

II. Supplementary Table S1

Major Eukaryotic Lineages
alveolata
amoebozoa
apusozoa
choanoflagellida
cristidiscoidea
cryptophyta
diplomonadida
euglenozoa
filasterea
fungi
haptophyceae
heterolobosea
ichthyosporea
metazoa
parabasalia
rhizaria
rhodophyta
stramenopiles
viridiplantae

III. Supplementary Table S2

Protein / protein family	Domain annotation
Calmodulin family	EF-hand_7+EF-hand_7
ERp57 (PDIA3)	Thioredoxin+Thioredoxin_6+Thioredoxin+Thioredoxin_8
SERCA	Cation_ATPase_N+TM+TM+E1-E2_ATPase+TM+TM+Cation_ATPase+Hydrolase+Hydrolase_3+TM+ Cation_ATPase_C
CaMK / CDPK	Pkinase^
Calstabin	FKBP_c
PPP2R3	EF-hand_7*
Calcineurin A	Metallophos
Calcineurin B	EF-hand_7+EF-hand_7+EF-hand_6+EF-hand_4
TMX1	Thioredoxin+TM
Calreticulin	Calreticulin
Calnexin	Calreticulin+TM
TRIC	TRIC
Homer (WH1 dom.)	EVH1^
ERdj5	DnaJ+Thioredoxin+Thioredoxin+Thioredoxin+Thioredoxin
Sarcalumenin	EHD_N+Dynamin_N
IP3R	Ins145_P3_rec+MIR+RYDR_ITPR+RYDR_ITPR+RIH_assoc+TM+TM+TM+TM+TM+TM
ORAI	Orai-1
NCS1	EF-hand_7+EF-hand_7+EF-hand_7+EF-hand_6
Calcipressin	Calcipressin
STIM	EF-hand+SAM+TM+SOAR
ERp44	Thioredoxin+Thioredoxin_6
RyR	Ins145_P3_rec+MIR+RYDR_ITPR+SPRY+RyR+RyR+SPRY+SPRY+RYDR_ITPR+RyR+RyR+RIH_assoc+ EF-hand_8+TM+TM+TM+TM+TM+TM
Sorcin	EF-hand_8+EF-hand_7
L-type VGCC (pore)	TM+TM+TM+TM+TM+TM+TM+TM+TM+TM+TM+TM+TM+TM+TM+TM+TM+TM+TM+TM+TM+TM+TM+ TM+GPHH+Ca_chan_IQ+CAC1F_C
TRPC	Ank_2+TRP_2+TM+TM+TM+TM+TM+TM

WFS2 (CISD2)	MitoNEET_N+zf-CDGSH
CREC family	EF-hand_7+EF-hand_7+EF-hand_7
Bcl2	BH4+Bcl-2+TM†
SelN	EF-hand_6
Wolframin	Sel1+Sel1+Sel1+wEF-hand+TM+TM+TM+TM+TM+TM+TM+TM+TM+wOB‡
Neurabin	PDZ*
Tespa1 (KRAP dom.)	KRAP_IP3R_bind
Calsequestrin	Calsequestrin
S100	S_100+EF-hand_7

^: Other domains vary

*: Other domains vary and are not very globular

†: Domain architecture varies between family members

‡: The domains wEP-hand and wOB refer to the wolframin-specific version of the EF-hand and OB-fold domains reported in this paper

IV. Supplementary Table S3

Alignment Abbreviation	Scientific name	Common name	Clade
Hsapiens	*Homo sapiens*	Human	*Mammalia*
Mmusculus	*Mus musculus*	House mouse	*Mammalia*
Ggallus	*Gallus gallus*	Chicken	*Aves*
Drerio	*Danio rerio*	Zebrafish	*Actinopterygii*
Skowalevskii	*Saccoglossus kowalevskii*	Acorn worm	*Hemichordata*
Dmelanogaster	*Drosophila melanogaster*	Fruit fly	*Hexapoda*
Tcastaneum	*Tribolium castaneum*	Red flour beetle	*Hexapoda*
Dpulex	Daphnia pulex	Water flea	*Crustacea*
Lpolyphemus	*Limulus polyphemus*	Atlantic horseshoe crab	*Chelicerata*
Lgigantea	*Lottia gigantea*	Owl limpet	*Mollusca*
Obimaculodies	*Octopus bimaculodies*	California two-spot octopus	*Mollusca*
Hrobusta	*Helobdella robusta*	Leech	*Annelida*
Celegans	*Caenorhabditis elegans*		*Nematoda*
Epallida	*Exaiptasia pallida*	Brown glass anemone	*Cnidaria*
Tadhaerens	*Trichoplax adhaerens*		*Placozoa*
Aqueesnlandica	*Amphimedon queenslandica*	Sponge	*Porifera*
Srosetta	*Salpingoeca rosetta*		*Choanoflagellatea*
Smoellendorffii	*Selaginella moellendorffii*	Spikemoss	*Streptophyta*
Vcarteri	*Volvox carteri f. nagariensis*		*Chlorophyta*
Tpseudonana	*Thalassiosira pseudonana CCMP1335*		*Bacillariophyta*
Pinfestans	*Phytophthora infestans T30-4*	Late potato blight	*Oomycetes*
Tgondii	*Toxoplasma gondii ME49*		*Apicomplexa*
Slemnae	*Stylonychia lemnae*		*Ciliophora*
Ehuxleyi	*Emiliania huxleyi CCMP1516*		*Haptophyta*

V. Supplementary Table S4

Human wolframin repeat 1		Human wolframin repeat 2		Human wolframin repeat 3		Canonical SLR^	
Position	Residue	Position	Residue	Position	Residue	Position	Residue
65	A	102	A	141	A	3	A
69	R	106	V	145	L	7	L
70	S	107	G	146	R	8	G
73	R	110	Y	149	L	11	Y
76	G	113	L	154	G	14	G
83	D	123	S	158	E	28	D
87	P	126	A	162	E	32	A
94	R	133	A	169	E	39	A
95	A	134	A	170	T	40	A
98	G	137	G	173	E	43	G

^: From Mittl & Schneider-Brachert, 2007

VI. Supplementary Figure S1

Name	Accession Used	metazoa>chordata>vertebrata	metazoa>chordata>tunicata	metazoa>chordata>cephalochordata	metazoa>echinodermata	metazoa>hemichordata	metazoa>arthropoda>hexapoda	metazoa>arthropoda>chelicerata	metazoa>arthropoda>crustacea	metazoa>nematoda	metazoa>priapulida	metazoa>annelida	metazoa>mollusca	metazoa>brachiopoda	metazoa>platyhelminthe	metazoa>cnidaria	metazoa>placozoa	metazoa>porifera	**Metazoan progenitor**	choanoflagellida	filasterea	**Holozoan progenitor**	fungi>dikarya	fungi>chytridiomycota	fungi>mucoromycotina	fungi>kickxellomycotina	**Fungal progenitor**	cristidiscoidea	apusozoa>apusomonadidae	amoebozoa>acanthamoebidae	amoebozoa>entamoebidae	amoebozoa>mycetozoa>dictyostelida	**Unnikont progenitor**	viridiplantae>streptophyta	viridiplantae>chlorophyta	rhodophyta	stramenopiles>bacillariophyta	stramenopiles>oomycetes	alveolata>apicomplexa	alveolata>ciliophora	rhizaria	**SAR progenitor**	haptophyceae	heterolobosea	euglenozoa>kinetoplastida	diplomonadida	parabasalia	**LECA**
Calmodulin family	NP_008819.1	P	P	P	P	P	P	P	P	P	P	P	P	P	P	P	P	P	P	P	P	P	P	P	P	P	P	P	P	P	P	P	P	P	P	P	P	P	P	P	P	P	P	P	P	P	P	P
ERp57 (PDIA3)	NP_005304.3	P	P	P	P	P	P	P	P	P	P	P	P	P	P	P	P	P	P	P	P	P	P	P	P	P	P	P	P	P	P	P	P	P	P	P	A	P	P	P	P	P	P	P	P	P	P	P
SERCA	NP_733765.1	P	P	P	P	P	P	P	P	P	P	P	P	P	P	P	P	P	P	P	P	P	P	P	P	P	P	P	P	P	P	A	P	P	P	P	P	P	P	P	P	P	P	P	P	A	P	P
CaMK / CDPK	NP_001308498.1	P	P	P	P	P	P	P	P	P	P	P	P	P	P	P	P	P	P	P	P	P	P	P	P	P	P	A	P	P	P	P	P	P	P	P	P	P	P	P	P	P	P	P	P	A	P	P
Calstabin	NP_463460.1	P	P	P	P	P	P	P	P	P	P	P	P	P	P	P	P	P	P	P	P	P	P	P	P	P	P	A	P	P	A	P	P	P	P	P	P	P	P	P	P	P	P	P	P	P	P	P
PPP2R3	NP_002709.2	P	P	P	P	P	P	P	P	P	P	P	P	P	P	P	P	P	P	P	A	P	A	P	P	P	P	P	P	P	A	P	P	P	P	P	P	P	P	P	P	P	P	P	P	P	P	P
Calcineurin A	NP_000935.1	P	P	P	P	P	P	P	P	P	P	P	P	P	P	P	P	P	P	P	P	P	P	P	P	P	P	P	P	P	P	P	P	A	A	A	A	P	P	P	P	P	A	P	P	P	P	P
Calcineurin B	NP_000936.1	P	P	P	P	P	P	P	P	P	P	P	P	P	P	P	P	P	P	P	P	P	P	P	P	P	P	P	P	P	P	P	P	P	P	P	A	A	P	P	P	P	P	P	P	P	P	P
TMX1	NP_110382.3	P	P	P	P	P	P	P	P	P	P	P	P	P	P	P	P	P	P	A	P	P	P	P	P	A	P	P	A	P	A	P	P	P	P	P	P	P	P	P	P	P	P	P	P	A	P	P
Calreticulin	NP_004334.1	P	P	P	P	P	P	P	P	P	P	P	P	P	P	P	P	P	P	P	P	P	A	P	P	P	P	P	A	P	P	P	P	P	P	A	P	P	A	P	P	P	P	P	P	A	P	P
Calnexin	NP_001737.1	P	P	P	P	P	P	P	P	P	P	P	P	P	P	P	P	P	P	P	A	P	P	P	A	P	P	A	P	A	A	P	P	P	P	P	A	P	P	A	P	P	P	P	A	A	P	P
TRIC	NP_076979.1	P	P	P	P	P	P	P	P	P	P	P	P	P	P	P	P	P	P	A	P	P	P	P	P	P	P	P	P	P	A	P	P	A	P	P	P	A	A	A	P	P	P	P	A	A	P	P
Homer (WH1 dom.)	NP_004263.1	P	P	P	P	P	P	P	P	P	P	P	P	P	P	P	P	P	P	P	P	P	P	P	P	A	P	P	P	P	A	P	P	A	A	P	A	A	A	A	P	P	A	P	A	A	P	P
ERdj5	NP_061854.1	P	P	P	P	P	P	P	P	P	A	P	P	P	P	P	P	A	P	P	A	P	A	A	A	A	A	A	A	P	A	A	P	P	P	A	A	P	P	P	P	P	P	A	A	A	P	P
Sarcalumenin	NP_001092284.1	P	P	P	P	P	P	P	P	P	P	P	P	P	P	P	P	P	P	P	P	P	A	P	P	P	P	P	P	P	P	P	P	P	P	P	P	P	P	A	P	P	P	A	P	A	A	A
IP3R	NP_001161744.1	P	P	P	P	P	P	P	P	P	P	P	P	P	P	P	P	P	P	P	P	P	A	P	P	A	P	P	P	P	A	P	P	A	P	A	A	P	A	P	P	P	P	P	P	A	A	A
ORAI	NP_116179.2	P	P	P	P	P	P	P	P	P	P	P	P	P	P	P	P	P	P	P	P	P	A	A	A	A	A	A	A	A	A	A	P	P	P	A	P	P	P	P	A	P	P	A	A	A	A	A
NCS1	NP_055101.2	P	P	P	P	P	P	P	P	P	P	P	P	P	P	P	P	P	P	P	P	P	P	P	P	P	P	A	P	P	A	P	P	A	A	A	A	A	A	A	A	A	A	A	A	A	A	A
Calcipressin	NP_001272320.2	P	P	P	P	P	P	P	P	P	P	P	P	P	P	P	P	P	P	P	P	P	P	P	P	P	P	A	A	P	A	P	P	A	A	A	A	A	A	A	A	A	A	A	A	A	A	A
STIM	NP_001264890.1	P	P	P	P	P	P	P	P	P	P	P	P	P	P	P	P	P	P	P	P	P	A	A	A	A	A	A	A	A	A	A	A	A	A	A	A	A	A	A	A	A	A	A	A	A	A	A
ERp44	NP_055866.1	P	P	P	P	P	P	P	P	P	P	P	P	P	P	P	P	P	P	P	P	P	A	A	A	A	A	A	A	A	A	A	A	A	A	A	A	A	A	A	A	A	A	A	A	A	A	A
RyR	NP_000531.2	P	P	P	P	P	P	P	P	P	P	P	P	P	P	A	P	P	P	P	P	P	A	A	A	A	A	A	A	A	A	A	A	A	A	A	A	A	A	A	A	A	A	A	A	A	A	A
Sorcin	NP_003121.1	P	A	P	P	P	A	A	A	A	A	A	P	P	A	P	P	P	P	P	P	P	A	A	A	A	A	A	A	A	A	A	A	A	A	A	A	A	A	A	A	A	A	A	A	A	A	A
L-type VGCC (pore)	NP_000060.2	P	P	P	P	P	P	P	P	P	P	P	P	P	P	P	P	P	P	P	A	A	A	A	A	A	A	A	A	A	A	A	A	A	A	A	A	A	A	A	A	A	A	A	A	A	A	A
TRPC	NP_003297.1	P	P	P	P	P	P	P	P	P	P	P	P	P	P	P	A	A	P	P	A	A	A	A	A	A	A	A	A	A	A	A	A	A	A	A	A	A	A	A	A	A	A	A	A	A	A	A
WFS2 (CISD2)	NP_001008389.1	P	P	P	P	P	P	A	P	A	P	P	P	P	P	P	P	P	P	P	A	A	A	A	A	A	A	A	A	A	A	A	A	A	A	A	A	P	A	A	A	A	A	A	A	A	A	A
CREC family	NP_001186601.1	P	P	P	P	P	P	P	P	P	P	P	P	P	P	P	P	P	P	A	A	A	A	A	A	A	A	A	A	A	A	A	A	P	A	A	A	A	A	A	A	A	A	A	A	A	A	A
Bcl2	NP_000624.2	P	P	P	P	P	P	P	P	P	P	P	P	P	P	P	P	P	P	A	A	A	A	A	A	A	A	A	A	A	A	A	A	A	A	A	A	A	A	A	A	A	A	A	A	A	A	A
SelN	NP_065184.2	P	P	P	P	P	A	P	A	A	P	P	P	P	P	P	A	P	P	A	A	A	A	A	A	A	A	A	A	A	A	A	A	A	A	A	A	A	A	A	A	A	A	A	A	A	A	A
Wolframin	NP_005996.2	P	P	A	A	P	P	P	P	P	A	P	P	P	A	P	P	P	P	A	A	A	A	A	A	A	A	A	A	A	A	A	A	A	A	A	A	A	A	A	A	A	A	A	A	A	A	A
Neurabin	NP_115984.3	P	P	P	P	P	P	P	P	P	P	P	P	P	P	P	P	A	A	A	A	A	A	A	A	A	A	A	A	A	A	A	A	A	A	A	A	A	A	A	A	A	A	A	A	A	A	A
Tespa1 (KRAP dom.)	NP_001129502.1	P	P	P	P	P	P	P	P	A	P	P	P	P	A	P	P	A	A	A	A	A	A	A	A	A	A	A	A	A	A	A	A	A	A	A	A	A	A	A	A	A	A	A	A	A	A	A
Calsequestrin	NP_001222.3	P	P	P	A	P	A	P	A	P	A	A	A	A	A	P	P	A	A	A	A	A	A	A	A	A	A	A	A	A	A	A	A	A	A	A	A	A	A	A	A	A	A	A	A	A	A	A
S100	NP_006262.1	P	P	A	A	A	A	A	A	A	A	A	A	A	A	A	A	A	A	A	A	A	A	A	A	A	A	A	A	A	A	A	A	A	A	A	A	A	A	A	A	A	A	A	A	A	A	A

VII. Supplementary Figure S2

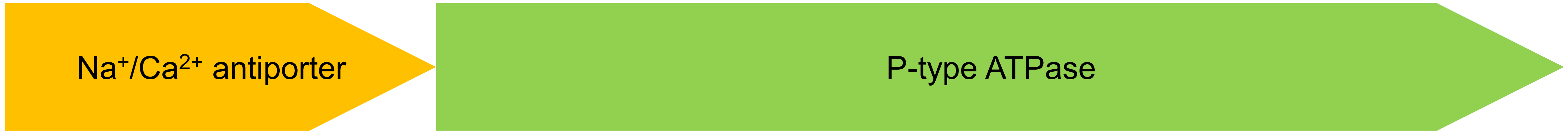

ABQ30334, ABQ30333 from *Acidiphilium cryptum JF-5* (α-proteobacteria)

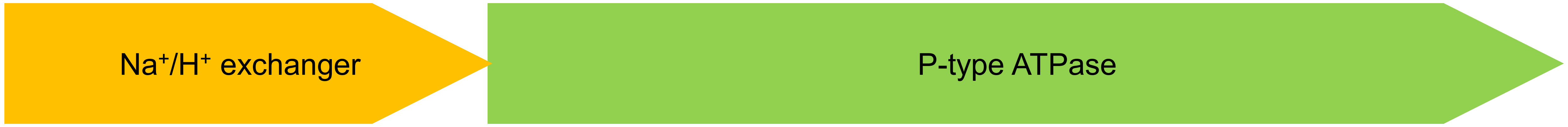

ADN08417, ADN08416 from *Sulfurimonas autotrophica DSM 16294* (ε-proteobacteria)

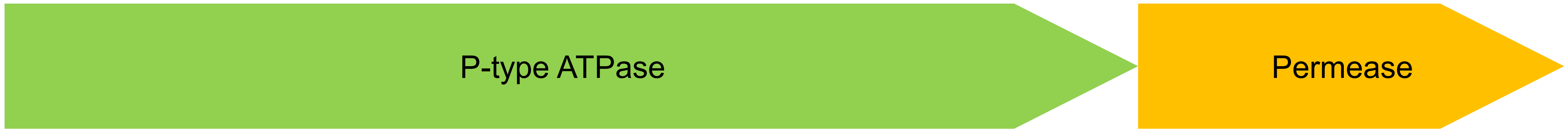

AFL87427, AFL87428 from *Terriglobus roseus DSM 18391* (acidobacteria)

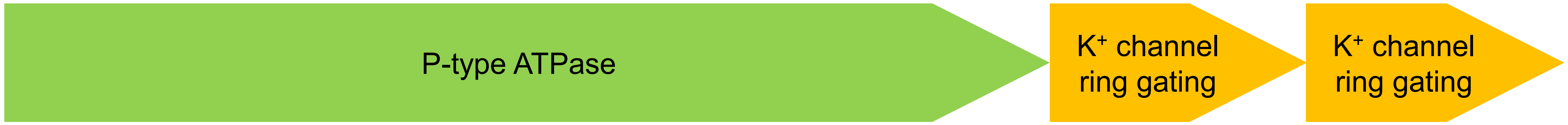

AEY94042, AEY94041, AEY94040 from *Streptomyces hygroscopicus subsp. jinggangensis 5008* (actinobacteria)

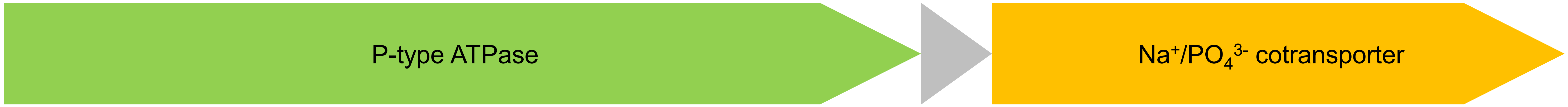

APC18922, APC18921, APC18920 from *Pseudomonas frederiksbergensis* (γ-proteobacteria)

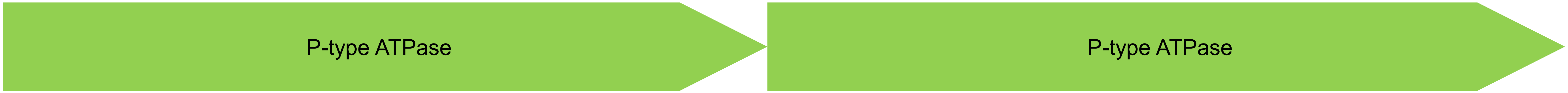

AEN97664, AEN97663 from *Roseburia hominis A2-183* (firmicutes)

VIII. Supplementary Figure S3

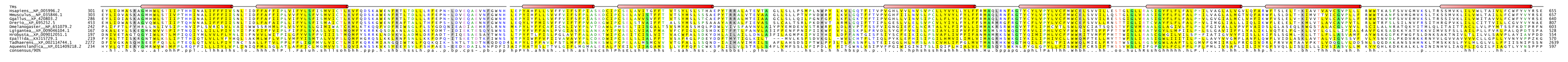

IX. Supplementary Figure S4

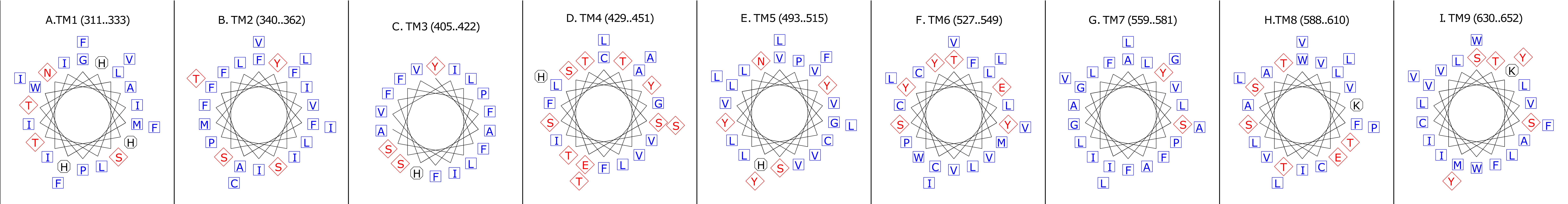

SUPPLEMENTARY DATA

Functional innovation in the evolution of the calcium-dependent system of the eukaryotic endoplasmic reticulum

Daniel E. Schäffer[1,2], Lakshminarayan M. Iyer[1], Alexander M. Burroughs[1], L Aravind[1]

1 - National Center for Biotechnology Information, National Library of Medicine, National Institutes of Health
2 - Montgomery Blair High School, Science, Mathematics, and Computer Science Magnet Program

ABSTRACT

The origin of eukaryotes was marked by the emergence of several novel subcellular systems. One such is the calcium (Ca2+)-stores system of the endoplasmic reticulum, which profoundly influences diverse aspects of cellular function including signal transduction, motility, division, and biomineralization. We use comparative genomics and sensitive sequence and structure analyses to investigate the evolution of this system. Our findings reconstruct the core form of the Ca2+- stores system in the last eukaryotic common ancestor as having at least 15 proteins that constituted a basic system for facilitating both Ca2+ flux across endomembranes and Ca2+-dependent signaling. We present evidence that the key EF-hand Ca2+-binding components had their origins in a likely bacterial symbiont other than the mitochondrial progenitor, whereas the protein phosphatase subunit of the ancestral calcineurin complex was likely inherited from the asgardarchaeal progenitor of the stem eukaryote. This further points to the potential origin of the eukaryotes in a Ca2+-rich biomineralized environment such as stromatolites. We further show that throughout eukaryotic evolution there were several acquisitions from bacteria of key components of the Ca2+-stores system, even though no prokaryotic lineage possesses a comparable system. Further, using quantitative measures derived from comparative genomics we show that there were several rounds of lineage-specific gene expansions, innovations of novel gene families, and gene losses correlated with biological innovation such as the biomineralized molluscan shells, coccolithophores, and animal motility. The burst of innovation of new genes in animals included the wolframin protein associated with Wolfram syndrome in humans. We show for the first time that it contains previously unidentified Sel1, EF-hand, and OB-fold domains, which might have key roles in its biochemistry.

TABLE OF CONTENTS

I. Edges in the protein-protein interaction network of ER-dependent Ca2+ storage and signaling
II. Accession numbers for homologs of proteins and protein families key to calcium storage and signaling, with their phylogenies
A. Calmodulin family
B. ERp57 (PDIA3)
C. SERCA
D. CaMK / CDPK
E. Calstabin
F. PPP2R3
G. Calcineurin A
H. Calcineurin B
I. TMX1
J. Calreticulin
K. Calnexin
L. TRIC
M. HOMER (WH1 dom.)
N. ERdj5
O. Sarcalumenin/EHD
P. IP3R
Q. ORAI
R. NCS1
S. Calcipressin
T. STIM
U. ERp44
V. RyR
W. Sorcin
X. L-type VGCC (pore)
Y. TRPC
Z. WFS2 (CISD2)
AA. CREC family
AB. Bcl2
AC. SelN
AD. Wolframin
AE. Neurabin
AF. Tespa1 (KRAP dom.)
AG. Calsequestrin
AH. S100
III. Accession numbers for prokaryotic relatives of proteins and protein families key to calcium storage and signaling, with their phylogenies
A. Accession numbers for selected calmodulin-like proteins with domain fusions
i. Fusions to Tic110-like domains
ii. Fusions to Heme-Oxygenase domains
iii Fusions to NO synthase domains
iv. Fusions to NADPH oxidase domains
v. Fusions to cNMP-binding domains
vi. Fusions to thioredoxin and/or sulfatase domains
vii. Fusions to glyoxalase domains
B. Accession numbers for selected bacterial PPIases
IV. Newick-format trees
A. Sarcalumenin, EHD, and bacterial relatives thereof
B. Calcineurin-like phosphatases, archaeal relatives thereof, and MRE11/rad32/sbcD-like phosphatases
C. Calcium/calmodulin-dependent kinases
D. ORAI and Jiraiya
V. Alignments
A. Alignment of representative Jiraya and ORAI family members
VI. List of completely sequenced curated eukaryotic genomes used for the analysis.

I. Edges in the protein-protein interaction network of ER-dependent Ca2+ storage and signaling

Edges which were included in the protein-protein interaction network presented in the paper are provided below in a tab-seperated format, where the first and second columns are two interacting proteins (represented by their encoding gene), the third column is the interaction type, and the fourth and subsequent columns (if any) provide the source of the interaction. There are three possible interaction types: 1 (interactions reported in the literature in which each partner gene either has no other paralog or has been specifically identified), 3 (reported interactions where the specific paralog for at least one of the two genes is unclear), and 4 (interactions found in FunCoup 4.0 with P = 0.9). Sources are in the form of PubMed IDs of papers that describe the interactions for types 1 and 3, and "FC P" for type 4 interactions, where P is the FunCoup P-score.

Gene 1	Gene 2	Type	Source	Second source
ACYP2	ATP2A2	1	PMID16105684	
AGTRAP	ATP2A2	1	PMID27015675	
AHCYL1	CAMK2A	1	PMID19133301	PMID25922519
AHCYL1	CISD2	1	PMID19133301	
AHCYL1	ITPR1	1	PMID19133301	
AHCYL1	ITPR2	1	PMID17429043	
AHCYL1	ITPR3	1	PMID17429043	
AHCYL1	PPP1CA	1	PMID19133301	
AHCYL1	SRL	1	PMID10398759	
AKAP6	PDE4D	1	PMID19345240	
AKAP6	RYR1	1	PMID12709444	
AKAP6	RYR2	1	PMID19345240	
AKAP9	ITPR1	1	PMID19133301	
AKAP9	PPP1CA	1	PMID19133301	PMID14982933
AKT1	CAMK2G	1	PMID26803057	
AKT1	ITPR1	1	PMID19133301	
AKT1	ITPR2	1	PMID19133301	
AKT1	ITPR3	1	PMID19133301	
AKT1	PML	1	PMID21030605	
ALDOA	CAPN3	1	PMID18676612	
ALDOA	RYR1	1	PMID18676612	
ANK1	ATP2A1	1	PMID28487373	
ANK1	OBSCN	1	PMID19002483	
ANK1	SLN	1	PMID28487373	
ANK2	ITPR1	1	PMID17429043	
ANK2	SIGMAR1	1	PMID17429043	
ANXA7	SRI	1	PMID9268363	PMID10767422
ASPH	CASQ1	1	PMID20961976	
ASPH	CASQ2	1	PMID20961976	
ASPH	ORAI1	1	PMID22586105	
ASPH	RYR1	1	PMID19398037	
ASPH	RYR2	1	PMID15041652	
ASPH	STIM1	1	PMID22586105	
ASPH	TRDN	1	PMID20961976	
ASPH	TRPC2	1	PMID16153633	
ASPH	TRPC5	1	PMID16153633	
ATF6	WFS1	1	PMID20160352	
ATG5	BCL2	1	PMID19043451	
ATP2A1	BCL2	1	PMID16105864	
ATP2A1	IRS1	1	PMID16105684	
ATP2A1	IRS2	1	PMID16105684	
ATP2A1	MRLN	1	PMID26816378	
ATP2A1	SLN	1	PMID28487373	
ATP2A1	SMIM6	1	PMID27923914	
ATP2A1	SRL	1	PMID16105684	
ATP2A1	STIM1	1	PMID24077737	
ATP2A2	BCL2	1	PMID16105684	PMID26772784
ATP2A2	CALR	1	PMID16105684	
ATP2A2	CALU	1	PMID20356454	PMID18562801
ATP2A2	CAMK2A	1	PMID16105684	PMID25515219
ATP2A2	CAMK2B	1	PMID16105684	PMID25515219
ATP2A2	CANX	1	PMID16105684	PMID21234650
ATP2A2	DNAJC10	1	PMID28614305	PMID27694578
ATP2A2	HRC	1	PMID22952658	
ATP2A2	IRS1	1	PMID16105684	
ATP2A2	IRS2	1	PMID16105684	
ATP2A2	MRLN	1	PMID26816378	
ATP2A2	NOX4	1	PMID24386930	
ATP2A2	OPRD1	1	PMID21234650	
ATP2A2	PLN	1	PMID27923914	
ATP2A2	PRKG1	1	PMID16105684	
ATP2A2	PSEN1	1	PMID18591429	
ATP2A2	PSEN2	1	PMID18591429	
ATP2A2	S100A1	1	PMID16105684	
ATP2A2	SDF4	1	PMID25772237	
ATP2A2	SELENON	1	PMID28506443	PMID25452428
ATP2A2	SLN	1	PMID27923914	
ATP2A2	SMIM6	1	PMID27923914	
ATP2A2	STIM1	1	PMID25772237	
ATP2A2	TMEM203	1	PMID25996873	
ATP2A2	TMX1	1	PMID28619231	
ATP2A2	TP53	1	PMID26772784	PMID25624484
ATP2A2	WFS1	1	PMID25274773	
ATP2A3	BCL2	1	PMID27639965	
ATP2A3	IRS1	1	PMID16105684	
ATP2A3	MRLN	1	PMID26316378	
ATP2A3	SMIM6	1	PMID27923914	
ATP2A3	STIM1	1	PMID18439569	
BAK1	ITPR1	1	PMID15613488	
BANK1	ITPR1	1	PMID19133301	
BANK1	ITPR2	1	PMID19133301	
BANK1	LYN	1	PMID19133301	
BAP1	ITPR3	1	PMID28614305	PMID28619231
BAX	BID	1	PMID19043451	
BAX	ITPR1	1	PMID15613488	
BAX	TMBIM6	1	PMID21718971	
BCL2	BECN1	1	PMID28725634	PMID11309306
BCL2	CISD2	1	PMID22617439	
BCL2	FKBP8	1	PMID19043451	PMID12510191
BCL2	ITPR1	1	PMID15613488	PMID19133301, PMID22617439
BCL2	ITPR2	1	PMID19133301	PMID19752026, PMID22617439
BCL2	ITPR3	1	PMID19133301	PMID22617439
BCL2	NR4A1	1	PMID19043451	
BCL2	PPP1CA	1	PMID19133301	
BCL2	RYR1	1	PMID24762814	
BCL2	RYR2	1	PMID24762814	
BCL2	RYR3	1	PMID25957473	PMID24762814
BCL2	TMBIM6	1	PMID21718971	
BCL2L1	FKBP8	1	PMID12510191	
BCL2L1	ITPR1	1	PMID19133301	
BCL2L1	ITPR2	1	PMID19133301	
BCL2L1	ITPR3	1	PMID19133301	
BCL2L1	RYR3	1	PMID25872771	
BCL2L1	TMBIM6	1	PMID21718971	
BECN1	CAV1	1	PMID28542134	
BECN1	ITPR1	1	PMID17256008	
BECN1	ITPR3	1	PMID17256008	
BECN1	PIK3C3	1	PMID28725634	PMID28542134
BOK	ITPR1	1	PMID26772784	
BOK	ITPR2	1	PMID26772784	
BRCA1	ITPR1	1	PMID26772784	
CABP1	ITPR1	1	PMID17429043	PMID14685260
CABP1	ITPR2	1	PMID17429043	
CABP1	ITPR3	1	PMID17429043	
CABP2	ITPR1	1	PMID12032348	
CABP2	ITPR2	1	PMID12032348	
CABP2	ITPR3	1	PMID12032348	
CABP5	ITPR1	1	PMID12032348	
CABP5	ITPR2	1	PMID12032348	
CABP5	ITPR3	1	PMID12032348	
CACNA1C	CALM1	1	PMID16299511	
CACNA1C	CALM2	1	PMID16299511	
CACNA1C	CALM3	1	PMID16299511	
CACNA1C	OPRD1	1	PMID25552382	
CACNA1C	RYR2	1	PMID20961976	
CACNA1S	CAV3	1	PMID22020936	
CACNA1S	JPH1	1	PMID22020936	
CACNA1S	JPH2	1	PMID22020936	
CACNA1S	RYR1	1	PMID22020936	PMID20961976
CALM1	ITPR1	1	PMID19133301	
CALM1	ITPR2	1	PMID19133301	
CALM1	ITPR3	1	PMID19133301	
CALM1	ORAI1	1	PMID23109337	
CALM1	PPP3CA	1	PMID12809556	PMID21115349
CALM1	RYR1	1	PMID20961976	
CALM1	RYR2	1	PMID20961976	
CALM1	RYR3	1	PMID20961976	
CALM1	WFS1	1	PMID19292454	
CALM2	ORAI1	1	PMID23109337	
CALM2	PPP3CA	1	PMID21115349	
CALM2	RYR1	1	PMID20961976	
CALM2	WFS1	1	PMID19292454	
CALM3	ORAI1	1	PMID23109337	
CALM3	PPP3CA	1	PMID21115349	
CALM3	RYR1	1	PMID20961976	
CALM3	WFS1	1	PMID19292454	
CALR	PDIA3	1	PMID16105684	PMID28619231
CALU	RYR1	1	PMID16527250	
CALU	RYR2	1	PMID20356454	
CAMK2A	FYN	1	PMID28212797	
CAMK2A	RYR3	1	PMID23825405	
CAMK2D	FYN	1	PMID28212797	
CAMK2D	ITPR2	1	PMID19133301	PMID23019322
CAMK2D	PLN	1	PMID28209766	
CAMK2D	RYR2	1	PMID28209766	PMID20961976
CAMK2D	SRI	1	PMID17707398	
CANX	CSNK2A1	1	PMID10994779	
CANX	CSNK2A2	1	PMID10994779	
CANX	PACS2	1	PMID18417615	
CANX	PDIA3	1	PMID16105684	
CAPN2	CAPNS1	1	PMID25422446	
CAPN2	CISD2	1	PMID25422446	
CAPN3	RYR1	1	PMID18676612	
CAPNS1	CISD2	1	PMID25422446	
CASP10	TNFRSF10A	1	PMID9325248	
CASP10	TNFRSF10B	1	PMID9325248	
CASP3	CASP8	1	PMID28049841	
CASP3	ITPR1	1	PMID17429043	PMID11013462
CASP3	ITPR2	1	PMID17429043	PMID11013462
CASP7	CASP8	1	PMID28049841	
CASP8	RYR2	1	PMID21788490	
CASP8	TNFRSF10A	1	PMID9325248	
CASP8	TNFRSF10B	1	PMID9325248	
CASQ1	CSNK2A1	1	PMID10398759	PMID1985907
CASQ1	CSNK2A2	1	PMID10398759	PMID1985907
CASQ1	RYR1	1	PMID20961976	
CASQ1	TRDN	1	PMID20961976	
CASQ2	CSNK2A1	1	PMID10398759	PMID1985907
CASQ2	CSNK2A2	1	PMID10398759	PMID1985907
CASQ2	PDIA3	1	PMID10398759	
CASQ2	RYR2	1	PMID20961976	
CASQ2	TRDN	1	PMID20961976	
CAV1	PIK3C3	1	PMID28542134	
CAV3	JPH1	1	PMID22020936	
CAV3	JPH2	1	PMID22020936	
CAV3	RYR1	1	PMID22020936	
CD38	RYR1	1	PMID16751979	
CD38	TRPC1	1	PMID16751979	
CD38	TRPC3	1	PMID16751979	
CD38	TRPC4	1	PMID16751979	
CD38	TRPC5	1	PMID16751979	
CD38	TRPC6	1	PMID16751979	
CD38	TRPC7	1	PMID16751979	
CDK1	ITPR1	1	PMID19133301	
CDK1	ITPR3	1	PMID19133301	
CHGA	ITPR1	1	PMID17429043	
CHGB	ITPR1	1	PMID17429043	
CIB1	ITPR1	1	PMID17429043	
CIB1	ITPR2	1	PMID17429043	
CIB1	ITPR3	1	PMID17429043	
CLIC2	RYR1	1	PMID19356589	
CSNK2A1	CSNK2A2	1	PMID10398759	
CSNK2A1	CSNK2B	1	PMID10398759	
CSNK2A1	SRL	1	PMID10398759	
CSNK2A2	CSNK2B	1	PMID10398759	
CSNK2A2	SRL	1	PMID10398759	
CYBB	RYR2	1	PMID24386930	
EPB41L1	GRIA1	1	PMID17429043	
EPB41L1	ITPR1	1	PMID17429043	
ERLIN1	ERLIN2	1	PMID27378757	
ERLIN1	ITPR1	1	PMID27378757	PMID19240031
ERLIN1	ITPR2	1	PMID27378757	
ERLIN1	ITPR3	1	PMID27378757	
ERLIN1	RNF170	1	PMID27378757	
ERLIN2	ITPR1	1	PMID27378757	
ERLIN2	ITPR2	1	PMID27378757	
ERLIN2	ITPR3	1	PMID27378757	
ERLIN2	RNF170	1	PMID27378757	
ERO1A	ERP44	1	PMID28619231	
ERO1A	ITPR1	1	PMID28619231	PMID19752026
FBXL2	ITPR3	1	PMID28614300	
FKBP1A	ITPR1	1	PMID17429043	
FKBP1A	ITPR2	1	PMID17429043	
FKBP1A	ITPR3	1	PMID17429043	
FKBP1A	PPP3CA	1	PMID9346894	
FKBP1A	RYR1	1	PMID24509862	
FKBP1A	RYR2	1	PMID24509862	
FKBP1A	RYR3	1	PMID24509862	
FKBP1A	TRPC3	1	PMID23228564	
FKBP1A	TRPC6	1	PMID23228564	
FKBP1B	RYR1	1	PMID20961976	
FKBP1B	RYR2	1	PMID20961976	
FKBP1B	RYR3	1	PMID20961976	
FKBP4	ORAI1	1	PMID23228564	
FKBP4	TRPC1	1	PMID23228564	
FKBP4	TRPC4	1	PMID23228564	
FKBP4	TRPC5	1	PMID23228564	
FKBP8	PPP3CA	1	PMID15757646	
FYN	ITPR1	1	PMID19133301	
GAPDH	ITPR1	1	PMID17429043	
GRM1	HOMER1	1	PMID17429043	PMID12072556
GRM1	HOMER3	1	PMID17429043	PMID12072556
GRM5	HOMER1	1	PMID17429043	PMID12072556
GRM5	HOMER3	1	PMID17429043	PMID12072556
GSTM2	RYR2	1	PMID18308613	
HAP1	HTT	1	PMID17429043	
HAP1	ITPR1	1	PMID12877301	PMID17429043
HOMER1	ITPR1	1	PMID16226241	PMID12072556
HOMER1	RYR1	1	PMID16226241	PMID 12223488
HOMER1	TRPC1	1	PMID17429043	PMID14505576
HOMER2	RYR1	1	PMID16226241	PMID12223488
HOMER3	ITPR1	1	PMID16226241	PMID12072556
HOMER3	RYR1	1	PMID16226241	PMID12223488
HRC	TRDN	1	PMID22952658	
HSP90B1	WFS1	1	PMID19125190	
HSPA5	ITPR1	1	PMID28619231	PMID21145001
HSPA5	ITPR3	1	PMID28619231	
HSPA5	SIGMAR1	1	PMID28619231	
HTT	ITPR1	1	PMID12877301	PMID17429043
ITPR1	ITPRIP	1	PMID16990268	
ITPR1	KRAS	1	PMID26772784	
ITPR1	LYN	1	PMID19133301	
ITPR1	MAPK1	1	PMID19133301	
ITPR1	MAPK3	1	PMID19133301	
ITPR1	MCL1	1	PMID26772784	
ITPR1	MRVI1	1	PMID19133301	
ITPR1	NCS1	1	PMID21104311	PMID16691292
ITPR1	ORAI1	1	PMID25506690	
ITPR1	PKD2	1	PMID16223735	
ITPR1	PLK1	1	PMID19133301	
ITPR1	PRKG1	1	PMID19133301	PMID10724174
ITPR1	PTEN	1	PMID26772784	
ITPR1	RACK1	1	PMID25506690	
ITPR1	RCN1	1	PMID28319095	
ITPR1	NRIP1	1	PMID25066731	
ITPR1	RNF170	1	PMID27378757	PMID21610068
ITPR1	ROCK2	1	PMID19133301	
ITPR1	SELENOK	1	PMID25368151	
ITPR1	SIGMAR1	1	PMID28619231	
ITPR1	SRC	1	PMID19133301	
ITPR1	STIM1	1	PMID25506690	
ITPR1	STIM2	1	PMID25506690	
ITPR1	TESPA1	1	PMID28598420	
ITPR1	TMEM203	1	PMID25996873	
ITPR1	TRPC3	1	PMID25506690	
ITPR1	ZDHHC6	1	PMID25368151	
ITPR2	LYN	1	PMID19133301	
ITPR2	RNF170	1	PMID27378757	PMID21610068
ITPR2	ROCK2	1	PMID19133301	
ITPR2	TRPC1	1	PMID23228564	
ITPR3	KRAS	1	PMID26772784	
ITPR3	PLK1	1	PMID19133301	
ITPR3	PML	1	PMID26772784	PMID28515000
ITPR3	PTEN	1	PMID26772784	
ITPR3	RNF170	1	PMID27378757	PMID21610068
ITPR3	ROCK2	1	PMID19133301	
ITPR3	SIGMAR1	1	PMID28619231	PMID17981125
JPH1	RYR1	1	PMID22020936	
JPH2	RYR1	1	PMID22020936	
JPH2	RYR2	1	PMID22020936	
JPH2	SPEG	1	PMID27729468	
MRLN	PLN	1	PMID26816378	
MRLN	SLN	1	PMID26816378	
MRVI1	PRKG1	1	PMID19133301	
NOD1	RYR2	1	PMID28126160	
NOX4	RYR1	1	PMID24386930	
OPRD1	RYR2	1	PMID25552382	
ORAI1	RACK1	1	PMID25506690	
ORAI1	SARAF	1	PMID29223474	
ORAI1	STIM1	1	PMID25506690	
ORAI1	STIM2	1	PMID25506690	PMID20807283
ORAI1	TRPC1	1	PMID23228564	
ORAI1	TRPC3	1	PMID25506690	
ORAI1	TRPC4	1	PMID23228564	
ORAI1	TRPC6	1	PMID23228564	
PDE4D	RYR2	1	PMID19345240	
PDIA3	STIM1	1	PMID21941299	PMID28619231
PKD1	PKD2	1	PMID16223735	
PLN	S100A1	1	PMID12804600	
PPP1R9B	RYR2	1	PMID19345240	
PPP2R3A	RYR2	1	PMID19345240	
PPP3CA	PPP3R1	1	PMID21115349	
PPP3CA	PPP3R2	1	PMID21115349	
PPP3CA	RCAN1	1	PMID12809556	
PPP5C	S100A1	1	PMID27600583	
PRKG1	RYR2	1	PMID20961976	
RACK1	STIM1	1	PMID25506690	
RACK1	TRPC3	1	PMID25506690	
RYR1	S100A1	1	PMID18650434	
RYR1	SELENON	1	PMID28506443	
RYR1	TMEM38A	1	PMID28387457	
RYR1	TRPC3	1	PMID16751979	
RYR1	TRPC5	1	PMID16751979	
RYR2	S100A1	1	PMID18650434	
RYR2	SPEG	1	PMID27729468	
RYR2	SRI	1	PMID17707398	
RYR2	TMEM38A	1	PMID24526676	
RYR2	TRDN	1	PMID15041652	
SARAF	STIM1	1	PMID29223474	
SARAF	TRPC1	1	PMID29223474	
SELENOK	ZDHHC6	1	PMID25368151	
STIM1	STIM2	1	PMID25506690	
STIM1	TMEM110	1	PMID26419802	
STIM1	TMEM203	1	PMID25996873	
STIM1	TRPC1	1	PMID25772237	
STIM1	TRPC3	1	PMID25506690	
TNFRSF10A	TNFSF10	1	PMID9325248	
TNFRSF10B	TNFSF10	1	PMID9325248	
ATP2A2	CAMK2D	3	PMID16105684	
ATP2A2	CAMK2G	3	PMID16105684	
BECN1	CAMK2A	3	PMID28725634	
BECN1	CAMK2B	3	PMID28725634	
BECN1	CAMK2D	3	PMID28725634	
BECN1	CAMK2G	3	PMID28725634	
CALM1	CAMK2A	3	PMID20961976	
CALM1	CAMK2B	3	PMID20961976	
CALM1	CAMK2D	3	PMID20961976	
CALM1	CAMK2G	3	PMID20961976	
CALM2	CAMK2A	3	PMID20961976	
CALM2	CAMK2B	3	PMID20961976	
CALM2	CAMK2D	3	PMID20961976	
CALM2	CAMK2G	3	PMID20961976	
CALM3	CAMK2A	3	PMID20961976	
CALM3	CAMK2B	3	PMID20961976	
CALM3	CAMK2D	3	PMID20961976	
CALM3	CAMK2G	3	PMID20961976	
CAMK2A	PLN	3	PMID20961976	
CAMK2A	RYR1	3	PMID20961976	
CAMK2A	RYR2	3	PMID20961976	
CAMK2B	PLN	3	PMID20961976	
CAMK2B	RYR1	3	PMID20961976	
CAMK2B	RYR2	3	PMID20961976	
CAMK2D	PLN	3	PMID20961976	
CAMK2D	RYR1	3	PMID20961976	
CAMK2G	PLN	3	PMID20961976	
CAMK2G	RYR1	3	PMID20961976	
CAMK2G	RYR2	3	PMID20961976	
CISD2	ITPR1	3	PMID22617439	
CISD2	ITPR2	3	PMID22617439	
CISD2	ITPR3	3	PMID22617439	
GRM5	ITPR2	3	PMID19133301	
GRM5	ITPR3	3	PMID19133301	
AHCYL1	CALU	4	FC0.92	
AHCYL1	ERP44	4	FC0.957	
AKAP6	CALM1	4	FC0.979	
AKAP6	CALM3	4	FC0.921	
AKAP9	CALM1	4	FC0.983	
AKAP9	CALM2	4	FC0.983	
AKAP9	CALM3	4	FC0.982	
AKT1	CALM1	4	FC0.957	
AKT1	CALM2	4	FC0.963	
AKT1	CALM3	4	FC0.957	
AKT1	CAMK2A	4	FC0.972	
AKT1	PPP3CA	4	FC0.987	
ALDOA	ATP2A2	4	FC0.998	
ALDOA	CALM1	4	FC1.0	
ALDOA	CALM2	4	FC1.0	
ALDOA	CALM3	4	FC1.0	
ALDOA	CALR	4	FC1.0	
ALDOA	CALU	4	FC0.999	
ALDOA	CANX	4	FC0.997	
ALDOA	FKBP1A	4	FC1.0	
ALDOA	FKBP1B	4	FC0.966	
ALDOA	PDIA3	4	FC1.0	
ALDOA	SRI	4	FC0.998	
ALDOA	TMX1	4	FC0.999	
ANK2	HOMER1	4	FC0.927	
ASPH	ATP2A2	4	FC0.993	
ASPH	CALM1	4	FC0.955	
ASPH	CALM2	4	FC0.945	
ASPH	CALM3	4	FC0.97	
ASPH	CALU	4	FC0.999	
ASPH	CANX	4	FC0.993	
ASPH	ERP44	4	FC0.999	
ASPH	TMX1	4	FC0.992	
ATP2A1	ATP2A2	4	FC0.994	
ATP2A1	ATP2A3	4	FC0.991	
ATP2A1	HSP90B1	4	FC0.963	
ATP2A1	PLN	4	FC0.918	
ATP2A2	ATP2A3	4	FC1.0	
ATP2A2	BAX	4	FC0.997	
ATP2A2	CALM1	4	FC1.0	
ATP2A2	CALM2	4	FC1.0	
ATP2A2	CALM3	4	FC1.0	
ATP2A2	CAPNS1	4	FC0.995	
ATP2A2	CSNK2A1	4	FC0.929	
ATP2A2	ERLIN2	4	FC0.986	
ATP2A2	ERO1A	4	FC1.0	
ATP2A2	ERP44	4	FC0.999	
ATP2A2	FKBP8	4	FC0.911	
ATP2A2	GAPDH	4	FC1.0	
ATP2A2	HSP90B1	4	FC1.0	
ATP2A2	HSPA5	4	FC1.0	
ATP2A2	ITPR3	4	FC0.9	
ATP2A2	PDIA3	4	FC0.999	
ATP2A2	PPP1CA	4	FC0.998	
ATP2A2	RACK1	4	FC0.999	
ATP2A3	CALM1	4	FC0.988	
ATP2A3	CALM2	4	FC0.988	
ATP2A3	CALM3	4	FC0.988	
ATP2A3	CALR	4	FC0.999	
ATP2A3	CALU	4	FC0.954	
ATP2A3	CANX	4	FC0.986	
ATP2A3	ERLIN2	4	FC0.904	
ATP2A3	ERO1A	4	FC1.0	
ATP2A3	GAPDH	4	FC0.994	
ATP2A3	HSP90B1	4	FC1.0	
ATP2A3	HSPA5	4	FC0.984	
ATP2A3	PDIA3	4	FC0.924	
ATP2A3	TMX1	4	FC0.996	
BAK1	BCL2	4	FC0.999	
BAX	BCL2	4	FC1.0	
BAX	CALM1	4	FC1.0	
BAX	CALM2	4	FC1.0	
BAX	CALM3	4	FC1.0	
BAX	CANX	4	FC1.0	
BAX	ERP44	4	FC0.992	
BAX	PDIA3	4	FC0.907	
BAX	TMX1	4	FC0.999	
BCL2	BCL2L1	4	FC1.0	
BCL2	BID	4	FC0.928	
BCL2	CASP8	4	FC0.927	
BCL2	MAPK1	4	FC0.969	
BCL2	MCL1	4	FC0.996	
BCL2	PSEN1	4	FC0.927	
BECN1	CALM1	4	FC0.999	
BECN1	CALM2	4	FC0.999	
BECN1	CALM3	4	FC0.998	
BECN1	CALR	4	FC0.99	
CACNA1S	CALM1	4	FC0.942	
CACNA1S	CALM2	4	FC0.915	
CACNA1S	CALM3	4	FC0.942	
CALM1	CALM2	4	FC0.918	
CALM1	CALM3	4	FC0.992	
CALM1	CALR	4	FC1.0	
CALM1	CALU	4	FC1.0	
CALM1	CANX	4	FC1.0	
CALM1	CAPNS1	4	FC0.999	
CALM1	CSNK2A1	4	FC1.0	
CALM1	CSNK2A2	4	FC0.924	
CALM1	CSNK2B	4	FC1.0	
CALM1	ERLIN2	4	FC0.998	
CALM1	ERO1A	4	FC1.0	
CALM1	ERP44	4	FC0.95	
CALM1	FKBP1A	4	FC1.0	
CALM1	FKBP1B	4	FC0.989	
CALM1	FKBP4	4	FC1.0	
CALM1	GAPDH	4	FC1.0	
CALM1	GRM5	4	FC0.998	
CALM1	HOMER1	4	FC0.93	
CALM1	HSP90B1	4	FC1.0	
CALM1	HSPA5	4	FC1.0	
CALM1	HTT	4	FC0.998	
CALM1	KRAS	4	FC1.0	
CALM1	LYN	4	FC0.985	
CALM1	MAPK1	4	FC1.0	
CALM1	MAPK3	4	FC0.998	
CALM1	PDIA3	4	FC1.0	
CALM1	PLK1	4	FC0.99	
CALM1	PPP1CA	4	FC1.0	
CALM1	PPP3R1	4	FC1.0	
CALM1	PPP3R2	4	FC0.985	
CALM1	PPP5C	4	FC0.999	
CALM1	RACK1	4	FC1.0	
CALM1	SDF4	4	FC0.975	
CALM1	SRI	4	FC0.996	
CALM1	TMBIM6	4	FC0.986	
CALM1	TMX1	4	FC0.984	
CALM2	CALR	4	FC1.0	
CALM2	CALU	4	FC1.0	
CALM2	CANX	4	FC1.0	
CALM2	CAPNS1	4	FC0.999	
CALM2	CSNK2A1	4	FC1.0	

CALM2	CSNK2A2	4	FC0.922
CALM2	CSNK2B	4	FC1.0
CALM2	ERLIN2	4	FC0.997
CALM2	ERO1A	4	FC1.0
CALM2	FKBP1A	4	FC1.0
CALM2	FKBP1B	4	FC0.99
CALM2	FKBP4	4	FC1.0
CALM2	GAPDH	4	FC1.0
CALM2	GRM5	4	FC0.99
CALM2	HSP90B1	4	FC1.0
CALM2	HSPA5	4	FC1.0
CALM2	HTT	4	FC0.998
CALM2	ITPR1	4	FC0.982
CALM2	KRAS	4	FC0.999
CALM2	LYN	4	FC0.986
CALM2	MAPK1	4	FC1.0
CALM2	MAPK3	4	FC0.999
CALM2	PDIA3	4	FC1.0
CALM2	PLK1	4	FC0.99
CALM2	PPP1CA	4	FC1.0
CALM2	PPP3R1	4	FC1.0
CALM2	PPP3R2	4	FC0.985
CALM2	PPP5C	4	FC0.998
CALM2	RACK1	4	FC1.0
CALM2	RYR2	4	FC1.0
CALM2	SDF4	4	FC0.971
CALM2	SRI	4	FC0.998
CALM2	TMBIM6	4	FC0.976
CALM2	TMX1	4	FC0.989
CALM3	CALR	4	FC1.0
CALM3	CALU	4	FC1.0
CALM3	CANX	4	FC1.0
CALM3	CAPNS1	4	FC0.999
CALM3	CSNK2A1	4	FC1.0
CALM3	CSNK2A2	4	FC0.922
CALM3	CSNK2B	4	FC1.0
CALM3	ERLIN2	4	FC0.997
CALM3	ERO1A	4	FC1.0
CALM3	FKBP1A	4	FC1.0
CALM3	FKBP1B	4	FC0.989
CALM3	FKBP4	4	FC1.0
CALM3	GAPDH	4	FC1.0
CALM3	GRM5	4	FC0.998
CALM3	HOMER1	4	FC0.906
CALM3	HSP90B1	4	FC1.0
CALM3	HSPA5	4	FC1.0
CALM3	HTT	4	FC0.998
CALM3	ITPR1	4	FC0.907
CALM3	KRAS	4	FC1.0
CALM3	LYN	4	FC0.986
CALM3	MAPK1	4	FC1.0
CALM3	MAPK3	4	FC0.999
CALM3	PDIA3	4	FC1.0
CALM3	PLK1	4	FC0.991
CALM3	PPP1CA	4	FC1.0
CALM3	PPP3R1	4	FC1.0
CALM3	PPP3R2	4	FC0.985
CALM3	PPP5C	4	FC0.999
CALM3	RACK1	4	FC1.0
CALM3	RYR2	4	FC1.0
CALM3	SDF4	4	FC0.975
CALM3	SRI	4	FC0.997
CALM3	TMBIM6	4	FC0.959
CALM3	TMX1	4	FC0.986
CALR	CALU	4	FC1.0
CALR	CANX	4	FC1.0
CALR	CAPN2	4	FC0.996
CALR	ERLIN2	4	FC0.992
CALR	ERO1A	4	FC1.0
CALR	ERP44	4	FC1.0
CALR	FKBP1A	4	FC1.0
CALR	FKBP4	4	FC1.0
CALR	GAPDH	4	FC1.0
CALR	HSP90B1	4	FC1.0
CALR	HSPA5	4	FC1.0
CALR	MAPK1	4	FC0.942
CALR	PPP1CA	4	FC1.0
CALR	PSEN1	4	FC0.984
CALR	RACK1	4	FC1.0
CALR	SDF4	4	FC0.999
CALR	TMX1	4	FC1.0
CALU	CANX	4	FC1.0
CALU	CAPN2	4	FC0.982
CALU	CISD2	4	FC0.942
CALU	CSNK2A1	4	FC0.996
CALU	CSNK2A2	4	FC0.998
CALU	DNAJC10	4	FC0.999
CALU	ERLIN1	4	FC0.906
CALU	ERLIN2	4	FC1.0
CALU	ERO1A	4	FC1.0
CALU	ERP44	4	FC1.0
CALU	FKBP1A	4	FC0.966
CALU	FKBP4	4	FC0.996
CALU	GAPDH	4	FC0.997
CALU	HSP90B1	4	FC1.0
CALU	HSPA5	4	FC1.0
CALU	MAPK1	4	FC0.987
CALU	PDIA3	4	FC1.0
CALU	PML	4	FC0.963
CALU	PPP5C	4	FC1.0
CALU	SDF4	4	FC1.0
CALU	SRI	4	FC0.937
CALU	TMX1	4	FC1.0
CAMK2A	CAMK2B	4	FC1.0
CAMK2A	CAMK2D	4	FC1.0
CAMK2A	CAMK2G	4	FC1.0
CAMK2A	GRIA1	4	FC0.996
CAMK2A	PPP3CA	4	FC0.96
CAMK2B	CAMK2D	4	FC1.0
CAMK2B	CAMK2G	4	FC0.999
CAMK2B	GRIA1	4	FC0.978
CAMK2D	CAMK2G	4	FC1.0
CAMK2D	FKBP4	4	FC0.922
CAMK2D	HSPA5	4	FC0.977
CAMK2D	ITPR3	4	FC0.985
CAMK2D	MAPK1	4	FC0.993
CAMK2D	PPP1CA	4	FC0.985
CAMK2D	PPP3CA	4	FC1.0
CAMK2G	GRIA1	4	FC0.998
CAMK2G	ITPR1	4	FC0.907
CAMK2G	PPP3CA	4	FC0.94
CAMK2G	RACK1	4	FC0.908
CANX	CAPN2	4	FC0.96
CANX	CAPNS1	4	FC0.997
CANX	DNAJC10	4	FC0.988
CANX	ERLIN2	4	FC1.0
CANX	ERO1A	4	FC1.0
CANX	ERP44	4	FC1.0
CANX	FKBP1A	4	FC0.988
CANX	FKBP4	4	FC1.0
CANX	FKBP8	4	FC0.982
CANX	GAPDH	4	FC1.0
CANX	HSP90B1	4	FC1.0
CANX	HSPA5	4	FC1.0
CANX	ITPR3	4	FC0.993
CANX	MAPK1	4	FC0.997
CANX	PKD2	4	FC0.997
CANX	PPP1CA	4	FC0.917
CANX	PPP5C	4	FC0.921
CANX	RACK1	4	FC0.999
CANX	SDF4	4	FC0.998
CANX	TMBIM6	4	FC0.998
CANX	TMX1	4	FC0.999
CANX	WFS1	4	FC0.942
CAPN2	FKBP1A	4	FC0.93
CAPN2	PDIA3	4	FC0.995
CAPN2	SRI	4	FC0.954
CAPNS1	PDIA3	4	FC0.908
CASQ1	CAV3	4	FC0.96
CASQ1	JPH2	4	FC0.921
CASQ2	JPH2	4	FC0.959
CAV1	ITPR3	4	FC1.0
CDK1	ERP44	4	FC1.0
CISD2	ERP44	4	FC0.952
CISD2	PDIA3	4	FC0.961
CISD2	TMX1	4	FC0.942
CSNK2A1	PDIA3	4	FC0.997
CSNK2A1	PPP1R9B	4	FC0.97
CSNK2A1	PPP3CA	4	FC0.927
CSNK2A1	SRI	4	FC0.999
CSNK2B	PDIA3	4	FC0.935
CSNK2B	PPP1R9B	4	FC0.943
CSNK2B	SRI	4	FC0.982
DNAJC10	HSPA5	4	FC1.0
DNAJC10	SDF4	4	FC0.905
ERLIN2	ERP44	4	FC0.984
ERLIN2	FKBP1A	4	FC0.963
ERLIN2	PDIA3	4	FC0.999
ERLIN2	SRI	4	FC0.999
ERLIN2	TMX1	4	FC0.999
ERO1A	PDIA3	4	FC1.0
ERO1A	TMX1	4	FC1.0
ERP44	FKBP1A	4	FC0.98
ERP44	FKBP4	4	FC0.984
ERP44	FKBP8	4	FC0.99
ERP44	HSP90B1	4	FC1.0
ERP44	HSPA5	4	FC1.0
ERP44	ITPR1	4	FC0.985
ERP44	MAPK1	4	FC0.975
ERP44	PDIA3	4	FC1.0
ERP44	PPP1CA	4	FC1.0
ERP44	SDF4	4	FC0.998
ERP44	SRI	4	FC0.973
ERP44	TMX1	4	FC0.992
FKBP1A	FKBP1B	4	FC0.976
FKBP1A	FKBP4	4	FC1.0
FKBP1A	GAPDH	4	FC0.999
FKBP1A	HSP90B1	4	FC1.0
FKBP1A	HSPA5	4	FC0.999
FKBP1A	MAPK1	4	FC1.0
FKBP1A	MAPK3	4	FC0.995
FKBP1A	PDIA3	4	FC0.999
FKBP1A	PPP5C	4	FC0.928
FKBP1A	RACK1	4	FC0.997
FKBP1A	SRI	4	FC0.907
FKBP1B	GAPDH	4	FC0.933
FKBP4	PDIA3	4	FC0.997
FKBP4	SRI	4	FC0.999
FKBP4	STIM1	4	FC0.936
FKBP4	TMX1	4	FC0.977
GAPDH	PDIA3	4	FC1.0
GAPDH	SRI	4	FC0.985
GAPDH	TMX1	4	FC0.998
GRM1	HOMER2	4	FC0.992
HOMER1	HOMER2	4	FC1.0
HOMER1	HOMER3	4	FC0.997
HOMER1	PPP3CA	4	FC0.992
HSP90B1	PDIA3	4	FC1.0
HSP90B1	RCN1	4	FC1.0
HSP90B1	SDF4	4	FC1.0
HSP90B1	SRI	4	FC0.956
HSP90B1	TMX1	4	FC1.0
HSPA5	PDIA3	4	FC1.0
HSPA5	PPP3CA	4	FC0.999
HSPA5	RCN1	4	FC1.0
HSPA5	SDF4	4	FC1.0
HSPA5	SRI	4	FC1.0
HSPA5	TMX1	4	FC0.994
ITPR1	ITPR3	4	FC1.0
ITPR1	PPP3CA	4	FC1.0
ITPR1	RYR2	4	FC0.987
ITPR1	TRPC4	4	FC0.966
ITPR3	TMX1	4	FC0.998
ITPR3	TRPC4	4	FC0.958
JPH1	TMEM38A	4	FC0.904
KRAS	PDIA3	4	FC0.947
LYN	TMX1	4	FC0.945
MAPK1	PDIA3	4	FC1.0
MAPK1	PPP1R9B	4	FC1.0
MAPK1	PPP3CA	4	FC0.981
MAPK1	SRI	4	FC0.999
MAPK1	TMX1	4	FC0.923
MAPK3	PDIA3	4	FC0.991
PDIA3	PPP1CA	4	FC0.984
PDIA3	RACK1	4	FC1.0
PDIA3	TMX1	4	FC1.0
PKD2	TRPC1	4	FC0.936
PPP1CA	PPP1R9B	4	FC1.0
PPP1CA	SRI	4	FC1.0
PPP1R9B	PPP3CA	4	FC0.994
RACK1	RYR2	4	FC0.933
RACK1	TMX1	4	FC0.915
RYR1	RYR2	4	FC0.999
RYR1	TRDN	4	FC0.992
RYR2	RYR3	4	FC0.999
SDF4	TMX1	4	FC0.985
TMX1	ZDHHC6	4	FC0.908
TRPC1	TRPC3	4	FC0.919
TRPC4	TRPC5	4	FC0.968

II. Accession numbers for homologs of proteins and protein families key to calcium storage and signaling, with their phylogenies

Accession numbers which were included in the phylogenetic analyses presented in the paper are provided below in clusters which correspond to the 34 proteins and protein families studied. Accession numbers are further annotated by the following columns in order from left to right: length, GenBank gene name, phylogeny, genus, species, and GenBank defline. Some accessions (in the format Aaaa##########) correspond to translationed gene predictions done by our group for genomes without published protein predictions.

A. Calmodulin family

NP_001031523.1	289	ARABIDOPSIS,THALIANA,CALMODULIN,LIKE,4,ATCAL4,calmodulin-like,12,CALMODULIN-RELATED,PROTEIN,CML12,T3K9.13,T3K9_13,TOUCH,3	eukaryota>viridiplantae	Arabidopsis	thaliana	Calcium-binding EF hand family protein [Arabidopsis thaliana]
NP_001318400.1	289	ARABIDOPSIS,THALIANA,CALMODULIN,LIKE,4,ATCAL4,calmodulin-like,12,CALMODULIN-RELATED,PROTEIN,CML12,T3K9.13,T3K9_13,TOUCH,3	eukaryota>viridiplantae	Arabidopsis	thaliana	Calcium-binding EF hand family protein [Arabidopsis thaliana]
NP_001324806.1	289	ARABIDOPSIS,THALIANA,CALMODULIN,LIKE,4,ATCAL4,calmodulin-like,12,CALMODULIN-RELATED,PROTEIN,CML12,T3K9.13,T3K9_13,TOUCH,3	eukaryota>viridiplantae	Arabidopsis	thaliana	Calcium-binding EF hand family protein [Arabidopsis thaliana]
XP_015838420.1	276	LOC659938	eukaryota>metazoa>hexapoda	Tribolium	castaneum	PREDICTED: calmodulin-beta isoform X1 [Tribolium castaneum]
XP_624589.2	276	LOC552209	eukaryota>metazoa>hexapoda	Apis	mellifera	PREDICTED: calmodulin-beta-like isoform X1 [Apis mellifera]
NP_660201.1	275	CRSD,CSNB2B	eukaryota>metazoa>chordata>vertebrata	Homo	sapiens	calcium-binding protein 4 isoform a [Homo sapiens]
XP_003273984.1	275	CABP4	eukaryota>metazoa>chordata>vertebrata	Nomascus	leucogenys	PREDICTED: calcium-binding protein 4 [Nomascus leucogenys]
XP_005577127.1	275	CABP4	eukaryota>metazoa>chordata>vertebrata	Macaca	fascicularis	PREDICTED: calcium-binding protein 4 isoform X2 [Macaca fascicularis]
XP_011150174.1	274	LOC105189634	eukaryota>metazoa>hexapoda	Harpegnathos	saltator	PREDICTED: calmodulin-beta isoform X1 [Harpegnathos saltator]
OQV24127.1	274	BV898_02079	eukaryota>metazoa	Hypsibius	dujardini	putative Calmodulin [Hypsibius dujardini]
XP_016771317.1	268	LOC552209	eukaryota>metazoa>hexapoda	Apis	mellifera	PREDICTED: calmodulin-A-like isoform X2 [Apis mellifera]
XP_002602906.1	266	BRAFLDRAFT_98094	eukaryota>metazoa>chordata	Branchiostoma	floridae	hypothetical protein BRAFLDRAFT_98094 [Branchiostoma floridae]
KXJ07556.1	265	AC249_AIPGENE20456	eukaryota>metazoa>cnidaria	Exaiptasia	pallida	Calmodulin [Exaiptasia pallida]
XP_015766476.1	265	LOC107345284	eukaryota>metazoa>cnidaria	Acropora	digitifera	PREDICTED: calmodulin-like [Acropora digitifera]
KXJ21637.1	263	AC249_AIPGENE18020	eukaryota>metazoa>cnidaria	Exaiptasia	pallida	Calmodulin [Exaiptasia pallida]
XP_006558596.1	262	LOC552209	eukaryota>metazoa>hexapoda	Apis	mellifera	PREDICTED: neo-calmodulin-like isoform X3 [Apis mellifera]
XP_008197239.1	262	LOC659938	eukaryota>metazoa>hexapoda	Tribolium	castaneum	PREDICTED: neo-calmodulin isoform X2 [Tribolium castaneum]
XP_016875727.1	262	CALBRAIN,HCALB_BR	eukaryota>metazoa>chordata>vertebrata	Homo	sapiens	PREDICTED: calcium-binding protein 1 isoform X4 [Homo sapiens]
XP_008197240.1	260	LOC659938	eukaryota>metazoa>hexapoda	Tribolium	castaneum	PREDICTED: neo-calmodulin isoform X3 [Tribolium castaneum]
XP_011150175.1	260	LOC105189634	eukaryota>metazoa>hexapoda	Harpegnathos	saltator	PREDICTED: calmodulin-A isoform X2 [Harpegnathos saltator]
XP_016771318.1	259	LOC552209	eukaryota>metazoa>hexapoda	Apis	mellifera	PREDICTED: neo-calmodulin-like isoform X4 [Apis mellifera]
XP_001948572.2	259	LOC100162151	eukaryota>metazoa>hexapoda	Acyrthosiphon	pisum	PREDICTED: neo-calmodulin-like isoform X1 [Acyrthosiphon pisum]
XP_015141709.1	258	CABP2	eukaryota>metazoa>chordata>vertebrata	Gallus	gallus	PREDICTED: calcium-binding protein 2 isoform X1 [Gallus gallus]
XP_006531829.1	256	Cabp2	eukaryota>metazoa>chordata>vertebrata	Mus	musculus	PREDICTED: calcium-binding protein 2 isoform X4 [Mus musculus]
XP_006531830.1	256	Cabp2	eukaryota>metazoa>chordata>vertebrata	Mus	musculus	PREDICTED: calcium-binding protein 2 isoform X4 [Mus musculus]
XP_012356868.1	256	CALM2	eukaryota>metazoa>chordata>vertebrata	Nomascus	leucogenys	PREDICTED: calmodulin isoform X1 [Nomascus leucogenys]
XP_003074444.1	255	Ot01g03840	eukaryota>viridiplantae>chlorophyta	Ostreococcus	tauri	calmodulin mutant SYNCAM9 (ISS) [Ostreococcus tauri]
XP_004927631.1	255	LOC101742890	eukaryota>metazoa>hexapoda	Bombyx	mori	neo-calmodulin [Bombyx mori]
XP_006558597.1	254	LOC552209	eukaryota>metazoa>hexapoda	Apis	mellifera	PREDICTED: neo-calmodulin-like isoform X5 [Apis mellifera]
XP_011608941.1	253	LOC101063225	eukaryota>metazoa>chordata>vertebrata>actinopterygii	Takifugu	rubripes	PREDICTED: calcium-binding protein 2-like [Takifugu rubripes]
KXJ25153.1	252	AC249_AIPGENE22463	eukaryota>metazoa>cnidaria	Exaiptasia	pallida	Calmodulin [Exaiptasia pallida]
XP_018099550.1	247	LOC108707103	eukaryota>metazoa>chordata>vertebrata	Xenopus	laevis	PREDICTED: calcium-binding protein 1-like isoform X2 [Xenopus laevis]
XP_971297.2	246	LOC659938	eukaryota>metazoa>hexapoda	Tribolium	castaneum	PREDICTED: neo-calmodulin isoform X4 [Tribolium castaneum]
XP_006558598.1	245	LOC552209	eukaryota>metazoa>hexapoda	Apis	mellifera	PREDICTED: neo-calmodulin-like isoform X6 [Apis mellifera]
XP_016657504.1	245	LOC100162151	eukaryota>metazoa>hexapoda	Acyrthosiphon	pisum	PREDICTED: neo-calmodulin-like isoform X2 [Acyrthosiphon pisum]
XP_011150176.1	244	LOC105189634	eukaryota>metazoa>hexapoda	Harpegnathos	saltator	PREDICTED: neo-calmodulin isoform X3 [Harpegnathos saltator]
XP_002161262.1	243	LOC100215966	eukaryota>metazoa>cnidaria	Hydra	vulgaris	PREDICTED: calmodulin-A-like [Hydra vulgaris]
Smar1000005545	243	Smar1000005545	eukaryota>metazoa>arthropoda>myriapoda	Strigamia	maritima	>Smar1000005545
XP_002168034.1	242	LOC100203928	eukaryota>metazoa>cnidaria	Hydra	vulgaris	PREDICTED: calmodulin-like [Hydra vulgaris]
XP_002503099.1	242	MICPUN_108387	eukaryota>viridiplantae>chlorophyta	Micromonas	commoda	predicted protein [Micromonas commoda]
CAF93636.1	242	GSTEN:00009382:G:001	eukaryota>metazoa>chordata>vertebrata>actinopterygii	Tetraodon	nigroviridis	unnamed protein product, partial [Tetraodon nigroviridis]
XP_016440228.1	241	LOC107766031	eukaryota>viridiplantae	Nicotiana	tabacum	PREDICTED: calmodulin-like protein 3 [Nicotiana tabacum]
XP_016463804.1	241	LOC107786814	eukaryota>viridiplantae	Nicotiana	tabacum	PREDICTED: calmodulin-like protein 3 [Nicotiana tabacum]
XP_003379560.1	241	Tsp_03217	eukaryota>metazoa>nematoda	Trichinella	spiralis	calmodulin-1 [Trichinella spiralis]
XP_016450019.1	240	LOC107774876	eukaryota>viridiplantae	Nicotiana	tabacum	PREDICTED: calmodulin-like protein 3 [Nicotiana tabacum]
XP_016479889.1	240	LOC107801119	eukaryota>viridiplantae	Nicotiana	tabacum	PREDICTED: calmodulin-like protein 3 [Nicotiana tabacum]
XP_017211029.1	240	zgc:112300	eukaryota>metazoa>chordata>vertebrata>actinopterygii	Danio	rerio	PREDICTED: calcium-binding protein 2 isoform X1 [Danio rerio]
XP_003372739.1	239	Tsp_11726	eukaryota>metazoa>nematoda	Trichinella	spiralis	EF hand domain containing protein [Trichinella spiralis]
XP_017328917.1	238	LOC108268443	eukaryota>metazoa>chordata>vertebrata>actinopterygii	Ictalurus	punctatus	PREDICTED: calcium-binding protein 2-like isoform X1 [Ictalurus punctatus]
XP_017338502.1	238	LOC108273649	eukaryota>metazoa>chordata>vertebrata>actinopterygii	Ictalurus	punctatus	PREDICTED: calcium-binding protein 5-like [Ictalurus punctatus]
XP_006345220.1	237	LOC102595634	eukaryota>viridiplantae	Solanum	tuberosum	PREDICTED: calmodulin-like protein 3 [Solanum tuberosum]
XP_003064500.1	237	MICPUCDRAFT_54225	eukaryota>viridiplantae>chlorophyta	Micromonas	pusilla CCMP1545	predicted protein [Micromonas pusilla CCMP1545]
NP_001025439.2	236	zgc:112300	eukaryota>metazoa>chordata>vertebrata>actinopterygii	Danio	rerio	calcium-binding protein 2 [Danio rerio]
XP_005160309.1	236	zgc:112300	eukaryota>metazoa>chordata>vertebrata>actinopterygii	Danio	rerio	PREDICTED: calcium-binding protein 2 isoform X2 [Danio rerio]
NP_001255489.1	236	cal-4	eukaryota>metazoa>nematoda	Caenorhabditis	elegans	CALmodulin related genes [Caenorhabditis elegans]
XP_003539163.2	236	LOC100780797	eukaryota>viridiplantae	Glycine	max	PREDICTED: probable calcium-binding protein CML17 [Glycine max]
XP_011401016.1	235	F751_0028	eukaryota>viridiplantae>chlorophyta	Auxenochlorella	protothecoides	Calmodulin [Auxenochlorella protothecoides]
OQV22258.1	235	BV898_03761	eukaryota>metazoa	Hypsibius	dujardini	putative Calmodulin [Hypsibius dujardini]
NP_001167949.1	234	GRMZM2G072052	eukaryota>viridiplantae	Zea	mays	uncharacterized protein LOC100381663 [Zea mays]
NP_500421.2	234	cal-3	eukaryota>metazoa>nematoda	Caenorhabditis	elegans	CALmodulin related genes [Caenorhabditis elegans]
XP_006358302.1	234	LOC102585648	eukaryota>viridiplantae	Solanum	tuberosum	PREDICTED: calmodulin-like protein 3 [Solanum tuberosum]
XP_015296417.1	234	CALM3	eukaryota>metazoa>chordata>vertebrata	Macaca	fascicularis	PREDICTED: calmodulin isoform X1 [Macaca fascicularis]
XP_003641327.1	234	CABP4	eukaryota>metazoa>chordata>vertebrata	Gallus	gallus	PREDICTED: calcium-binding protein 2 [Gallus gallus]
XP_009291662.1	233	zgc:112300	eukaryota>metazoa>chordata>vertebrata>actinopterygii	Danio	rerio	calcium-binding protein 2 isoform X1 [Danio rerio]
XP_017318617.1	233	cabp2	eukaryota>metazoa>chordata>vertebrata>actinopterygii	Ictalurus	punctatus	PREDICTED: calcium-binding protein 2 isoform X1 [Ictalurus punctatus]
XP_016515675.1	231	LOC107832358	eukaryota>viridiplantae	Nicotiana	tabacum	PREDICTED: calmodulin-like protein 1 [Nicotiana tabacum]
XP_017318618.1	231	cabp2	eukaryota>metazoa>chordata>vertebrata>actinopterygii	Ictalurus	punctatus	PREDICTED: calcium-binding protein 2 isoform X2 [Ictalurus punctatus]
XP_011610696.1	230	LOC101066819	eukaryota>metazoa>chordata>vertebrata>actinopterygii	Takifugu	rubripes	PREDICTED: calcium-binding protein 2-like [Takifugu rubripes]
XP_017318619.1	230	cabp2	eukaryota>metazoa>chordata>vertebrata>actinopterygii	Ictalurus	punctatus	PREDICTED: calcium-binding protein 2 isoform X3 [Ictalurus punctatus]
XP_016487971.1	230	LOC107808003	eukaryota>viridiplantae	Nicotiana	tabacum	PREDICTED: probable calcium-binding protein CML25 [Nicotiana tabacum]
NP_001240157.1	229	LOC100810081	eukaryota>viridiplantae	Glycine	max	calmodulin-like protein [Glycine max]
XP_003549743.1	229	LOC100803032	eukaryota>viridiplantae	Glycine	max	PREDICTED: calmodulin-like protein 3 [Glycine max]
CAF96460.1	229	GSTEN:00013499:G:001	eukaryota>metazoa>chordata>vertebrata>actinopterygii	Tetraodon	nigroviridis	unnamed protein product [Tetraodon nigroviridis]
XP_009037109.1	229	AURANDRAFT_26147	eukaryota>stramenopiles	Aureococcus	anophagefferens	hypothetical protein AURANDRAFT_26147 [Aureococcus anophagefferens]
XP_003372845.1	228	Tsp_10406	eukaryota>metazoa>nematoda	Trichinella	spiralis	calmodulin [Trichinella spiralis]
XP_006823622.1	228	LOC102802917	eukaryota>metazoa>hemichordata	Saccoglossus	kowalevskii	PREDICTED: calmodulin-B-like [Saccoglossus kowalevskii]
NP_038907.1	227	caldendrin	eukaryota>metazoa>chordata>vertebrata	Mus	musculus	calcium-binding protein 1 isoform 2 [Mus musculus]
NP_112482.1	227	CALBRAIN,HCALB_BR	eukaryota>metazoa>chordata>vertebrata	Homo	sapiens	calcium-binding protein 1 isoform 1 [Homo sapiens]
XP_003280334.1	227	CABP1	eukaryota>metazoa>chordata>vertebrata	Nomascus	leucogenys	PREDICTED: calcium-binding protein 1 isoform X1 [Nomascus leucogenys]
XP_005572469.1	227	CABP1	eukaryota>metazoa>chordata>vertebrata	Macaca	fascicularis	PREDICTED: calcium-binding protein 1 isoform X2 [Macaca fascicularis]
CDS36359.1	227	EmuJ_000344500	eukaryota>metazoa	Echinococcus	multilocularis	calmodulin [Echinococcus multilocularis]
ETO13322.1	227	RFI_24053	eukaryota>rhizaria	Reticulomyxa	filosa	calmodulin 2-like protein [Reticulomyxa filosa]
NP_001305425.1	226	DFNB93	eukaryota>metazoa>chordata>vertebrata	Homo	sapiens	calcium-binding protein 2 isoform 3 [Homo sapiens]
XP_012351438.1	226	CABP2	eukaryota>metazoa>chordata>vertebrata	Nomascus	leucogenys	PREDICTED: calcium-binding protein 2 isoform X1 [Nomascus leucogenys]
XP_015289856.1	226	CABP2	eukaryota>metazoa>chordata>vertebrata	Macaca	fascicularis	PREDICTED: calcium-binding protein 2 isoform X1 [Macaca fascicularis]
NP_001147205.1	226	LOC100280813	eukaryota>viridiplantae	Zea	mays	calmodulin [Zea mays]
XP_001233509.2	224	CABP1	eukaryota>metazoa>chordata>vertebrata	Gallus	gallus	PREDICTED: calcium-binding protein 1 isoform X5 [Gallus gallus]
XP_016612824.1	224	SPPG_00489	eukaryota>fungi>chytridiomycota	Spizellomyces	punctatus DAOM BR117	hypothetical protein SPPG_00489 [Spizellomyces punctatus DAOM BR117]
XP_019925473.1	223	LOC105334726	eukaryota>metazoa>mollusca	Crassostrea	gigas	PREDICTED: calmodulin-beta isoform X1 [Crassostrea gigas]
XP_019925474.1	223	LOC105334726	eukaryota>metazoa>mollusca	Crassostrea	gigas	PREDICTED: calmodulin isoform X2 [Crassostrea gigas]
XP_015141710.1	222	CABP2	eukaryota>metazoa>chordata>vertebrata	Gallus	gallus	PREDICTED: calcium-binding protein 2 isoform X2 [Gallus gallus]
NP_038906.2	221	Cabp2	eukaryota>metazoa>chordata>vertebrata	Mus	musculus	calcium-binding protein 2 isoform 1 [Mus musculus]
XP_001897398.1	221	Bm1_29780	eukaryota>metazoa>nematoda	Brugia	malayi	EF hand family protein [Brugia malayi]
NP_057450.2	220	DFNB93	eukaryota>metazoa>chordata>vertebrata	Homo	sapiens	calcium-binding protein 2 isoform 1 [Homo sapiens]
XP_003273991.1	220	CABP2	eukaryota>metazoa>chordata>vertebrata	Nomascus	leucogenys	PREDICTED: calcium-binding protein 2 isoform X2 [Nomascus leucogenys]
XP_005577112.1	220	CABP2	eukaryota>metazoa>chordata>vertebrata	Macaca	fascicularis	PREDICTED: calcium-binding protein 2 isoform X2 [Macaca fascicularis]
ELR18329.1	220	ACA1_371900	eukaryota>amoebozoa>acanthamoebidae	Acanthamoeba	castellanii str. Neff	EF hand domain containing protein [Acanthamoeba castellanii str. Neff]
XP_002285404.1	220	LOC100245811	eukaryota>viridiplantae	Vitis	vinifera	PREDICTED: calmodulin-like protein 3 [Vitis vinifera]
XP_016460667.1	219	LOC107784110	eukaryota>viridiplantae	Nicotiana	tabacum	PREDICTED: probable calcium-binding protein CML36 [Nicotiana tabacum]
XP_016462443.1	219	LOC107785616	eukaryota>viridiplantae	Nicotiana	tabacum	PREDICTED: probable calcium-binding protein CML36 [Nicotiana tabacum]
XP_006347296.1	219	LOC102598823	eukaryota>viridiplantae	Solanum	tuberosum	PREDICTED: calmodulin-like protein 3 [Solanum tuberosum]
XP_003554140.1	218	LOC100815559	eukaryota>viridiplantae	Glycine	max	PREDICTED: probable calcium-binding protein CML36 [Glycine max]
XP_017343770.1	217	LOC108276532	eukaryota>metazoa>chordata>vertebrata>actinopterygii	Ictalurus	punctatus	PREDICTED: calcium-binding protein 1-like isoform X2 [Ictalurus punctatus]
XP_011604896.1	216	LOC101066197	eukaryota>metazoa>chordata>vertebrata>actinopterygii	Takifugu	rubripes	PREDICTED: calcium-binding protein 2-like [Takifugu rubripes]
XP_017322763.1	216	LOC108265140	eukaryota>metazoa>chordata>vertebrata>actinopterygii	Ictalurus	punctatus	PREDICTED: calcium-binding protein 2-like isoform X2 [Ictalurus punctatus]
ERG84951.1	216	ASU_04582	eukaryota>metazoa>nematoda	Ascaris	suum	putative calcium-binding protein [Ascaris suum]
XP_003524630.2	216	LOC100786354	eukaryota>viridiplantae	Glycine	max	PREDICTED: calmodulin-like protein 1 [Glycine max]
NP_181672.1	216	F13H10.4,F13H10_4	eukaryota>viridiplantae	Arabidopsis	thaliana	Calcium-binding EF-hand family protein [Arabidopsis thaliana]
NP_001264540.1	215	CABP2	eukaryota>metazoa>chordata>vertebrata	Gallus	gallus	calcium-binding protein 2 [Gallus gallus]
NP_565996.1	215	F14B2.33,multicopy,suppressors,of,snf4,deficiency,in,yeast,3	eukaryota>viridiplantae	Arabidopsis	thaliana	Calcium-binding EF-hand family protein [Arabidopsis thaliana]
XP_003373971.1	215	Tsp_08698	eukaryota>metazoa>nematoda	Trichinella	spiralis	calmodulin [Trichinella spiralis]
XP_013426777.1	215	M436DRAFT_82463	eukaryota>fungi>ascomycota	Aureobasidium	namibiae CBS 147.97	EF-hand [Aureobasidium namibiae CBS 147.97]
Caps1000024397	215	Caps1000024397	eukaryota>metazoa>annelida	Capitella	spI	>Caps1000024397
XP_017316965.1	214	LOC108260839	eukaryota>metazoa>chordata>vertebrata>actinopterygii	Ictalurus	punctatus	PREDICTED: calcium-binding protein 2-like [Ictalurus punctatus]
XP_002266359.1	214	LOC100254364	eukaryota>viridiplantae	Vitis	vinifera	PREDICTED: calmodulin-like protein 3 [Vitis vinifera]
NP_001326523.1	214	AtCAM7,calmodulin,7,CALMODULIN,7,Z-box,Binding,Factor,3,ZBF3	eukaryota>viridiplantae	Arabidopsis	thaliana	calmodulin 7 [Arabidopsis thaliana]
KNE55575.1	213	AMAG_01466	eukaryota>fungi	Allomyces	macrogynus ATCC 38327	calmodulin [Allomyces macrogynus ATCC 38327]
XP_003874723.1	213	LMXM_36_3675	eukaryota>euglenozoa>kinetoplastida	Leishmania	mexicana MHOM/GT/2001/U1103	putative calmodulin [Leishmania mexicana MHOM/GT/2001/U1103]
XP_780862.3	213	LOC575365	eukaryota>metazoa>echinodermata	Strongylocentrotus	purpuratus	PREDICTED: calmodulin [Strongylocentrotus purpuratus]
XP_013400665.1	212	LOC106166584	eukaryota>metazoa	Lingula	anatina	PREDICTED: neo-calmodulin-like isoform X1 [Lingula anatina]
Smar1000001531	212	Smar1000001531	eukaryota>metazoa>arthropoda>myriapoda	Strigamia	maritima	>Smar1000001531
Smed1000023664	212	Smed1000023664	eukaryota>metazoa>platyhelminthe	Schmidtea	mediterranea	>Smed1000023664
XP_010663735.1	212	LOC100262849	eukaryota>viridiplantae	Vitis	vinifera	PREDICTED: putative calcium-binding protein CML19 [Vitis vinifera]
NP_001152651.1	211	LOC100286292	eukaryota>viridiplantae	Zea	mays	calmodulin [Zea mays]
XP_006351283.1	211	LOC102603406	eukaryota>viridiplantae	Solanum	tuberosum	PREDICTED: probable calcium-binding protein CML36 [Solanum tuberosum]
XP_011427010.1	210	LOC105328000	eukaryota>metazoa>mollusca	Crassostrea	gigas	PREDICTED: calmodulin-A isoform X1 [Crassostrea gigas]
XP_016493926.1	209	LOC107813208	eukaryota>viridiplantae	Nicotiana	tabacum	PREDICTED: probable calcium-binding protein CML36 [Nicotiana tabacum]
XP_016499615.1	209	LOC107818184	eukaryota>viridiplantae	Nicotiana	tabacum	PREDICTED: probable calcium-binding protein CML36 [Nicotiana tabacum]
XP_011455014.1	209	LOC105347567	eukaryota>metazoa>mollusca	Crassostrea	gigas	PREDICTED: neo-calmodulin [Crassostrea gigas]
NP_187630.1	209	AT3G10190	eukaryota>viridiplantae	Arabidopsis	thaliana	Calcium-binding EF-hand family protein [Arabidopsis thaliana]
NP_001255490.1	208	cal-4	eukaryota>metazoa>nematoda	Caenorhabditis	elegans	CALmodulin related genes [Caenorhabditis elegans]
Lhya1000005584	208	Lhya1000005584	eukaryota>fungi>mucoromycotina	Lichtheimia	hyalospora	>Lhya1000005584
XP_808089.1	207	Tc00.1047053507483.30	eukaryota>euglenozoa>kinetoplastida	Trypanosoma	cruzi strain CL Brener	calmodulin [Trypanosoma cruzi strain CL Brener]
Caps1000022106	207	Caps1000022106	eukaryota>metazoa>annelida	Capitella	spI	>Caps1000022106
XP_017318620.1	207	cabp2	eukaryota>metazoa>chordata>vertebrata>actinopterygii	Ictalurus	punctatus	PREDICTED: calcium-binding protein 2 isoform X4 [Ictalurus punctatus]
XP_018082625.1	207	LOC108697221	eukaryota>metazoa>chordata>vertebrata	Xenopus	laevis	PREDICTED: calcium-binding protein 2-like [Xenopus laevis]
NP_001256433.1	206	E02A10.3	eukaryota>metazoa>nematoda	Caenorhabditis	elegans	Uncharacterized protein CELE_E02A10.3 [Caenorhabditis elegans]
XP_011441728.2	206	LOC105338356	eukaryota>metazoa>mollusca	Crassostrea	gigas	PREDICTED: neo-calmodulin-like [Crassostrea gigas]
XP_012894039.1	206	GSBLH_T00000385001	eukaryota>stramenopiles	Blastocystis	hominis	uncharacterized protein [Blastocystis hominis]
XP_018117078.1	206	LOC108716018	eukaryota>metazoa>chordata>vertebrata	Xenopus	laevis	PREDICTED: calcium-binding protein 1 isoform X2 [Xenopus laevis]
XP_006351257.1	205	LOC102595230	eukaryota>viridiplantae	Solanum	tuberosum	PREDICTED: probable calcium-binding protein CML36 [Solanum tuberosum]
XP_003961445.1	205	LOC101077996	eukaryota>metazoa>chordata>vertebrata>actinopterygii	Takifugu	rubripes	PREDICTED: calcium-binding protein 5-like [Takifugu rubripes]
NP_190646.1	205	calmodulin-like,41	eukaryota>viridiplantae	Arabidopsis	thaliana	calmodulin-like 41 [Arabidopsis thaliana]
[illegible]	[illegible]	[illegible]	[illegible]	[illegible]	[illegible]	[illegible]

NP_001231973.1	170	LOC494376	eukaryota>metazoa>chordata	Ciona	intestinalis	calmodulin-like [Ciona intestinalis]
XP_005843158.1	170	CHLNCDRAFT_55421	eukaryota>viridiplantae>chlorophyta	Chlorella	variabilis	hypothetical protein CHLNCDRAFT_55421 [Chlorella variabilis]
XP_013236837.1	170	DI09_71p40	eukaryota>fungi>microsporidia	Mitosporidium	daphniae	calmodulin [Mitosporidium daphniae]
XP_789104.4	170	LOC584132	eukaryota>metazoa>echinodermata	Strongylocentrotus	purpuratus	PREDICTED: calmodulin-A [Strongylocentrotus purpuratus]
Caps1000019672	170	Caps1000019672	eukaryota>metazoa>annelida	Capitella	spI	>Caps1000019672
XP_017322402.1	170	cabp1	eukaryota>metazoa>chordata>vertebrata>actinopterygii	Ictalurus	punctatus	PREDICTED: calcium-binding protein 1 isoform X2 [Ictalurus punctatus]
NP_001146794.1	169	calmodulin,GRMZM2G444621	eukaryota>viridiplantae	Zea	mays	uncharacterized protein LOC100280399 [Zea mays]
XP_002946868.1	169	fap272	eukaryota>viridiplantae>chlorophyta	Volvox	carteri f. nagariensis	calmodulin-like protein Fap272 [Volvox carteri f. nagariensis]
XP_003543563.3	169	LOC100813656	eukaryota>viridiplantae	Glycine	max	PREDICTED: calmodulin-like protein 3 [Glycine max]
XP_005786038.1	169	CAM3	eukaryota>haptophyceae	Emiliania	huxleyi CCMP1516	calmodulin [Emiliania huxleyi CCMP1516]
XP_006678916.1	169	BATDEDRAFT_19649	eukaryota>fungi>chytridiomycota	Batrachochytrium	dendrobatidis JAM81	calmodulin [Batrachochytrium dendrobatidis JAM81]
XP_008648304.1	169	ZMCALM1	eukaryota>viridiplantae	Zea	mays	calmodulin isoform X1 [Zea mays]
XP_014771770.1	169	LOC106870257	eukaryota>metazoa>mollusca	Octopus	bimaculoides	PREDICTED: neo-calmodulin-like isoform X2 [Octopus bimaculoides]
NP_001018568.1	169	zgc:109977	eukaryota>metazoa>chordata>vertebrata>actinopterygii	Danio	rerio	calcium binding protein 5 [Danio rerio]
XP_001752070.1	169	PHYPADRAFT_111790	eukaryota>viridiplantae	Physcomitrella	patens	predicted protein [Physcomitrella patens]
XP_006345065.1	169	LOC102599351	eukaryota>viridiplantae	Solanum	tuberosum	PREDICTED: probable calcium-binding protein CML15 [Solanum tuberosum]
XP_011441749.1	168	LOC105338364	eukaryota>metazoa>mollusca	Crassostrea	gigas	PREDICTED: calmodulin-1-like [Crassostrea gigas]
XP_019927029.1	168	LOC105338364	eukaryota>metazoa>mollusca	Crassostrea	gigas	PREDICTED: calmodulin-1-like [Crassostrea gigas]
XP_019927030.1	168	LOC105338364	eukaryota>metazoa>mollusca	Crassostrea	gigas	PREDICTED: calmodulin-1-like [Crassostrea gigas]
ERG83959.1	168	ASU_06137	eukaryota>metazoa>nematoda	Ascaris	suum	calmodulin-like protein [Ascaris suum]
GAQ86360.1	168	KFL_002850040	eukaryota>viridiplantae	Klebsormidium	nitens	calmodulin [Klebsormidium nitens]
XP_013400705.1	168	LOC106166584	eukaryota>metazoa	Lingula	anatina	PREDICTED: calmodulin-A-like isoform X6 [Lingula anatina]
XP_015755705.1	168	LOC107335225	eukaryota>metazoa>cnidaria	Acropora	digitifera	PREDICTED: calmodulin-A-like [Acropora digitifera]
NP_001153724.1	168	Cabp2	eukaryota>metazoa>chordata>vertebrata	Mus	musculus	calcium-binding protein 2 isoform 2 [Mus musculus]
XP_003964546.1	168	LOC101075297	eukaryota>metazoa>chordata>vertebrata>actinopterygii	Takifugu	rubripes	PREDICTED: calcium-binding protein 5-like [Takifugu rubripes]
XP_005589779.1	168	CABP5	eukaryota>metazoa>chordata>vertebrata	Macaca	fascicularis	PREDICTED: calcium-binding protein 5 [Macaca fascicularis]
XP_011617066.1	168	cabp4	eukaryota>metazoa>chordata>vertebrata>actinopterygii	Takifugu	rubripes	PREDICTED: calcium-binding protein 4 isoform X3 [Takifugu rubripes]
XP_002277499.1	168	LOC100247858	eukaryota>viridiplantae	Vitis	vinifera	PREDICTED: putative calcium-binding protein CML19 [Vitis vinifera]
NP_001002414.1	167	Cabp1,zgc:92680	eukaryota>metazoa>chordata>vertebrata>actinopterygii	Danio	rerio	calcium-binding protein 1 [Danio rerio]
NP_001297644.1	167	caldendrin	eukaryota>metazoa>chordata>vertebrata	Mus	musculus	calcium-binding protein 1 isoform 3 [Mus musculus]
NP_004267.2	167	CALBRAIN,HCALB_BR	eukaryota>metazoa>chordata>vertebrata	Homo	sapiens	calcium-binding protein 1 isoform 2 [Homo sapiens]
XP_003280335.1	167	CABP1	eukaryota>metazoa>chordata>vertebrata	Nomascus	leucogenys	PREDICTED: calcium-binding protein 1 isoform X2 [Nomascus leucogenys]
XP_004934447.1	167	CABP1	eukaryota>metazoa>chordata>vertebrata	Gallus	gallus	PREDICTED: calcium-binding protein 1 isoform X6 [Gallus gallus]
XP_017343771.1	167	LOC108276532	eukaryota>metazoa>chordata>vertebrata>actinopterygii	Ictalurus	punctatus	PREDICTED: calcium-binding protein 1-like isoform X3 [Ictalurus punctatus]
XP_001901898.1	167	Bm1_52150	eukaryota>metazoa>nematoda	Brugia	malayi	Calmodulin-like protein [Brugia malayi]
ETO09562.1	167	RFI_27815	eukaryota>rhizaria	Reticulomyxa	filosa	calmodulin [Reticulomyxa filosa]
XP_001849785.1	167	CpipJ_CPIJ007602	eukaryota>metazoa>hexapoda	Culex	quinquefasciatus	calmodulin [Culex quinquefasciatus]
XP_003726780.1	167	LOC100891445	eukaryota>metazoa>echinodermata	Strongylocentrotus	purpuratus	PREDICTED: calmodulin-A [Strongylocentrotus purpuratus]
XP_011416018.1	167	LOC105319972	eukaryota>metazoa>mollusca	Crassostrea	gigas	PREDICTED: neo-calmodulin-like isoform X1 [Crassostrea gigas]
XP_016657505.1	167	LOC100162151	eukaryota>metazoa>hexapoda	Acyrthosiphon	pisum	PREDICTED: neo-calmodulin-like isoform X4 [Acyrthosiphon pisum]
Caps1000024396	167	Caps1000024396	eukaryota>metazoa>annelida	Capitella	spI	>Caps1000024396
XP_015160192.1	167	LOC107058689	eukaryota>viridiplantae	Solanum	tuberosum	PREDICTED: probable calcium-binding protein CML16 [Solanum tuberosum]
XP_011603190.1	166	LOC101065517	eukaryota>metazoa>chordata>vertebrata>actinopterygii	Takifugu	rubripes	PREDICTED: calcium-binding protein 1-like [Takifugu rubripes]
NP_174504.1	166	F27G20.1	eukaryota>viridiplantae	Arabidopsis	thaliana	Calcium-binding EF-hand family protein [Arabidopsis thaliana]
ERG83873.1	166	ASU_06315	eukaryota>metazoa>nematoda	Ascaris	suum	hypothetical protein ASU_06315 [Ascaris suum]
KXJ22231.1	166	calm	eukaryota>metazoa>cnidaria	Exaiptasia	pallida	Calmodulin [Exaiptasia pallida]
XP_009295966.1	166	calml4b	eukaryota>metazoa>chordata>vertebrata>actinopterygii	Danio	rerio	PREDICTED: calmodulin-like protein 4 [Danio rerio]
XP_015761991.1	166	LOC107341081	eukaryota>metazoa>cnidaria	Acropora	digitifera	PREDICTED: calmodulin-A-like [Acropora digitifera]
CAF91408.1	165	GSTEN:00006151:G:001	eukaryota>metazoa>chordata>vertebrata>actinopterygii	Tetraodon	nigroviridis	unnamed protein product, partial [Tetraodon nigroviridis]
NP_956992.1	165	zgc:73060	eukaryota>metazoa>chordata>vertebrata>actinopterygii	Danio	rerio	calcium binding protein 5a [Danio rerio]
XP_005156057.1	165	zgc:73060	eukaryota>metazoa>chordata>vertebrata>actinopterygii	Danio	rerio	calcium binding protein 5a isoform X1 [Danio rerio]
OAF65977.1	165	A3Q56_06298	eukaryota>metazoa	Intoshia	linei	hypothetical protein A3Q56_06298 [Intoshia linei]
XP_002733157.1	165	LOC100377516	eukaryota>metazoa>hemichordata	Saccoglossus	kowalevskii	PREDICTED: calmodulin-like [Saccoglossus kowalevskii]
XP_002946692.1	165	camA	eukaryota>viridiplantae>chlorophyta	Volvox	carteri f. nagariensis	calmodulin [Volvox carteri f. nagariensis]
XP_009050133.1	165	LOTGIDRAFT_142024	eukaryota>metazoa>mollusca	Lottia	gigantea	hypothetical protein LOTGIDRAFT_142024, partial [Lottia gigantea]
XP_009171875.1	165	T265_07946	eukaryota>metazoa	Opisthorchis	viverrini	hypothetical protein T265_07946 [Opisthorchis viverrini]
XP_969500.1	165	LOC657988	eukaryota>metazoa>hexapoda	Tribolium	castaneum	PREDICTED: calcium-binding protein E63-1 [Tribolium castaneum]
NP_186950.1	165	F13E7.5,F13E7_5	eukaryota>viridiplantae	Arabidopsis	thaliana	EF hand calcium-binding protein family [Arabidopsis thaliana]
XP_001765534.1	165	PHYPADRAFT_129576	eukaryota>viridiplantae	Physcomitrella	patens	predicted protein [Physcomitrella patens]
NP_001154754.1	164	ACAM-1,calmodulin,1,K22F20.20,K22F20_20,TCH1,TOUCH,1	eukaryota>viridiplantae	Arabidopsis	thaliana	calmodulin 1 [Arabidopsis thaliana]
XP_003279757.1	164	CALML6	eukaryota>metazoa>chordata>vertebrata	Nomascus	leucogenys	PREDICTED: calmodulin-like protein 6 [Nomascus leucogenys]
XP_011539143.1	164	CAGLP	eukaryota>metazoa>chordata>vertebrata	Homo	sapiens	PREDICTED: calmodulin-like protein 6 isoform X2 [Homo sapiens]
XP_006341535.1	164	LOC102599328	eukaryota>viridiplantae	Solanum	tuberosum	PREDICTED: calcium-binding allergen Ole e 8 [Solanum tuberosum]
XP_016468045.1	164	LOC107790609	eukaryota>viridiplantae	Nicotiana	tabacum	PREDICTED: calcium-binding allergen Ole e 8-like [Nicotiana tabacum]
XP_016503788.1	164	LOC107821840	eukaryota>viridiplantae	Nicotiana	tabacum	PREDICTED: calcium-binding allergen Ole e 8-like [Nicotiana tabacum]
CBJ26717.1	164	Esi_0042_0063	eukaryota>stramenopiles	Ectocarpus	siliculosus	conserved unknown protein [Ectocarpus siliculosus]
KXJ24804.1	164	AC249_AIPGENE21054	eukaryota>metazoa>cnidaria	Exaiptasia	pallida	Calmodulin [Exaiptasia pallida]
NP_001294291.1	164	cal-3	eukaryota>metazoa>nematoda	Caenorhabditis	elegans	CALmodulin related genes [Caenorhabditis elegans]
NP_651432.1	164	Dmel\CG17770,gene2,MLC2	eukaryota>metazoa>hexapoda	Drosophila	melanogaster	uncharacterized protein Dmel_CG17770 [Drosophila melanogaster]
XP_005535553.1	164	CYME_CME034C	eukaryota>rhodophyta	Cyanidioschyzon	merolae strain 10D	similar to calmodulin [Cyanidioschyzon merolae strain 10D]
XP_009061568.1	164	LOTGIDRAFT_219848	eukaryota>metazoa>mollusca	Lottia	gigantea	hypothetical protein LOTGIDRAFT_219848 [Lottia gigantea]
XP_012557820.1	164	LOC105845059	eukaryota>metazoa>cnidaria	Hydra	vulgaris	PREDICTED: calmodulin-beta-like [Hydra vulgaris]
Bcir1000004353	164	Bcir1000004353	eukaryota>fungi>mucoromycotina	Backusella	circina	>Bcir1000004353
XP_005169680.1	163	cabp2b	eukaryota>metazoa>chordata>vertebrata>actinopterygii	Danio	rerio	calcium-binding protein 2-like isoform X4 [Danio rerio]
XP_006352896.1	163	LOC102582093	eukaryota>viridiplantae	Solanum	tuberosum	PREDICTED: probable calcium-binding protein CML18 [Solanum tuberosum]
XP_010663082.1	163	LOC100241974	eukaryota>viridiplantae	Vitis	vinifera	PREDICTED: probable calcium-binding protein CML18 [Vitis vinifera]
XP_015166598.1	163	LOC102582093	eukaryota>viridiplantae	Solanum	tuberosum	PREDICTED: probable calcium-binding protein CML18 [Solanum tuberosum]
XP_016443439.1	163	LOC107768801	eukaryota>viridiplantae	Nicotiana	tabacum	PREDICTED: probable calcium-binding protein CML18 [Nicotiana tabacum]
XP_016443440.1	163	LOC107768801	eukaryota>viridiplantae	Nicotiana	tabacum	PREDICTED: probable calcium-binding protein CML18 [Nicotiana tabacum]
XP_006347583.1	163	LOC102583396	eukaryota>viridiplantae	Solanum	tuberosum	PREDICTED: probable calcium-binding protein CML18 [Solanum tuberosum]
XP_016446705.1	163	LOC107771773	eukaryota>viridiplantae	Nicotiana	tabacum	PREDICTED: probable calcium-binding protein CML18 [Nicotiana tabacum]
XP_016475017.1	163	LOC107796730	eukaryota>viridiplantae	Nicotiana	tabacum	PREDICTED: probable calcium-binding protein CML18 [Nicotiana tabacum]
XP_016504334.1	163	LOC107822318	eukaryota>viridiplantae	Nicotiana	tabacum	PREDICTED: probable calcium-binding protein CML18 [Nicotiana tabacum]
XP_019081382.1	163	LOC100266587	eukaryota>viridiplantae	Vitis	vinifera	PREDICTED: probable calcium-binding protein CML18 [Vitis vinifera]
XP_002973044.1	163	SELMODRAFT_413463	eukaryota>viridiplantae	Selaginella	moellendorffii	hypothetical protein SELMODRAFT_413463 [Selaginella moellendorffii]
XP_002984512.1	163	SELMODRAFT_423620	eukaryota>viridiplantae	Selaginella	moellendorffii	hypothetical protein SELMODRAFT_423620 [Selaginella moellendorffii]
XP_005704340.1	163	Gasu_46430	eukaryota>rhodophyta	Galdieria	sulphuraria	calmodulin isoform 2 [Galdieria sulphuraria]
XP_015769999.1	163	LOC107348473	eukaryota>metazoa>cnidaria	Acropora	digitifera	PREDICTED: calmodulin-like isoform X1 [Acropora digitifera]
XP_015770000.1	163	LOC107348473	eukaryota>metazoa>cnidaria	Acropora	digitifera	PREDICTED: calmodulin-like isoform X1 [Acropora digitifera]
ABP00426.1	163	OSTLU_42106	eukaryota>viridiplantae>chlorophyta	Ostreococcus	lucimarinus CCE9901	predicted protein [Ostreococcus lucimarinus CCE9901]
ERG81872.1	163	ASU_09517	eukaryota>metazoa>nematoda	Ascaris	suum	calmodulin-2 [Ascaris suum]
XP_001703420.1	163	CAM1	eukaryota>viridiplantae>chlorophyta	Chlamydomonas	reinhardtii	calmodulin [Chlamydomonas reinhardtii]
XP_003262876.1	163	CALM2	eukaryota>metazoa>chordata>vertebrata	Nomascus	leucogenys	PREDICTED: calmodulin isoform X2 [Nomascus leucogenys]
XP_011780243.1	163	TbgDal_XI10980	eukaryota>euglenozoa>kinetoplastida	Trypanosoma	brucei gambiense DAL972	calmodulin, putative [Trypanosoma brucei gambiense DAL972]
XP_013898473.1	163	MNEG_8507	eukaryota>viridiplantae>chlorophyta	Monoraphidium	neglectum	Calmodulin [Monoraphidium neglectum]
XP_017993026.1	163	Malapachy_2066	eukaryota>fungi>basidiomycota	Malassezia	pachydermatis	calmodulin [Malassezia pachydermatis]
Caps1000002071	163	Caps1000002071	eukaryota>metazoa>annelida	Capitella	spI	>Caps1000002071
XP_017322762.1	163	LOC108265140	eukaryota>metazoa>chordata>vertebrata>actinopterygii	Ictalurus	punctatus	PREDICTED: calcium-binding protein 2-like isoform X1 [Ictalurus punctatus]
NP_177504.1	163	F25P22.4,F25P22_4	eukaryota>viridiplantae	Arabidopsis	thaliana	EF hand calcium-binding protein family [Arabidopsis thaliana]
XP_001751654.1	163	PHYPADRAFT_64092	eukaryota>viridiplantae	Physcomitrella	patens	predicted protein [Physcomitrella patens]
XP_006592332.1	163	LOC100820547	eukaryota>viridiplantae	Glycine	max	PREDICTED: probable calcium-binding protein CML18 [Glycine max]
XP_010663678.1	163	LOC100264572	eukaryota>viridiplantae	Vitis	vinifera	PREDICTED: calcium-binding allergen Ole e 8 [Vitis vinifera]
XP_017328918.1	162	LOC108268443	eukaryota>metazoa>chordata>vertebrata>actinopterygii	Ictalurus	punctatus	PREDICTED: calcium-binding protein 2-like isoform X2 [Ictalurus punctatus]
XP_002969393.1	162	SELMODRAFT_91501	eukaryota>viridiplantae	Selaginella	moellendorffii	hypothetical protein SELMODRAFT_91501 [Selaginella moellendorffii]
CEL96874.1	162	Vbra_12077	eukaryota>alveolata	Vitrella	brassicaformis CCMP3155	unnamed protein product [Vitrella brassicaformis CCMP3155]
XP_015777729.1	162	LOC107355663	eukaryota>metazoa>cnidaria	Acropora	digitifera	PREDICTED: calmodulin-A-like [Acropora digitifera]
XP_018278324.1	162	CC85DRAFT_261315	eukaryota>fungi>basidiomycota	Cutaneotrichosporon	oleaginosum	EF-hand [Cutaneotrichosporon oleaginosum]
XP_796091.1	162	LOC591437	eukaryota>metazoa>echinodermata	Strongylocentrotus	purpuratus	PREDICTED: calmodulin-beta [Strongylocentrotus purpuratus]
Caps1000013555	162	Caps1000013555	eukaryota>metazoa>annelida	Capitella	spI	>Caps1000013555
XP_017318621.1	162	cabp2	eukaryota>metazoa>chordata>vertebrata>actinopterygii	Ictalurus	punctatus	PREDICTED: calcium-binding protein 2 isoform X5 [Ictalurus punctatus]
XP_003631231.1	162	LOC100264548	eukaryota>viridiplantae	Vitis	vinifera	PREDICTED: probable calcium-binding protein CML16 [Vitis vinifera]
NP_001189724.1	161	ATCAL5,calmodulin,2,CALMODULIN,2,T3K9.12,T3K9_12	eukaryota>viridiplantae	Arabidopsis	thaliana	calmodulin 2 [Arabidopsis thaliana]
NP_189188.4	161	T5M7.6	eukaryota>viridiplantae	Arabidopsis	thaliana	Calcium-binding EF-hand family protein [Arabidopsis thaliana]
XP_006367528.1	161	LOC102594874	eukaryota>viridiplantae	Solanum	tuberosum	PREDICTED: probable calcium-binding protein CML15 [Solanum tuberosum]
XP_016494339.1	161	LOC107813572	eukaryota>viridiplantae	Nicotiana	tabacum	PREDICTED: probable calcium-binding protein CML16 [Nicotiana tabacum]
XP_016514165.1	161	LOC107830979	eukaryota>viridiplantae	Nicotiana	tabacum	PREDICTED: probable calcium-binding protein CML16 [Nicotiana tabacum]
NP_001318691.1	161	AtCML24,CALMODULIN-LIKE,24,CML24,K22F20.10,K22F20_10,TOUCH,2	eukaryota>viridiplantae	Arabidopsis	thaliana	EF hand calcium-binding protein family [Arabidopsis thaliana]
NP_198593.1	161	AtCML24,CALMODULIN-LIKE,24,CML24,K22F20.10,K22F20_10,TOUCH,2	eukaryota>viridiplantae	Arabidopsis	thaliana	EF hand calcium-binding protein family [Arabidopsis thaliana]
XP_015299311.1	161	CALML6	eukaryota>metazoa>chordata>vertebrata	Macaca	fascicularis	PREDICTED: calmodulin-like protein 6 isoform X3 [Macaca fascicularis]
ERG81147.1	161	ASU_10655	eukaryota>metazoa>nematoda	Ascaris	suum	calmodulin [Ascaris suum]
NP_001256429.1	161	cal-1	eukaryota>metazoa>nematoda	Caenorhabditis	elegans	Calmodulin-like protein [Caenorhabditis elegans]
XP_001636954.1	161	NEMVEDRAFT_v1g228777	eukaryota>metazoa>cnidaria	Nematostella	vectensis	predicted protein [Nematostella vectensis]
XP_002736800.1	161	LOC100367223	eukaryota>metazoa>hemichordata	Saccoglossus	kowalevskii	PREDICTED: calmodulin-like [Saccoglossus kowalevskii]
XP_003063495.1	161	MICPUCDRAFT_22291	eukaryota>viridiplantae>chlorophyta	Micromonas	pusilla CCMP1545	predicted protein [Micromonas pusilla CCMP1545]
XP_006820195.1	161	LOC100366976	eukaryota>metazoa>hemichordata	Saccoglossus	kowalevskii	PREDICTED: calmodulin-like [Saccoglossus kowalevskii]
XP_013402365.1	161	LOC106167995	eukaryota>metazoa	Lingula	anatina	PREDICTED: neo-calmodulin-like [Lingula anatina]
XP_013892536.1	161	MNEG_14446	eukaryota>viridiplantae>chlorophyta	Monoraphidium	neglectum	hypothetical protein MNEG_14446 [Monoraphidium neglectum]
XP_014666516.1	161	LOC106808343	eukaryota>metazoa	Priapulus	caudatus	PREDICTED: calmodulin-A-like isoform X1 [Priapulus caudatus]
Caps1000025199	161	Caps1000025199	eukaryota>metazoa>annelida	Capitella	spI	>Caps1000025199
Smed1000007464	161	Smed1000007464	eukaryota>metazoa>platyhelminthe	Schmidtea	mediterranea	>Smed1000007464
XP_003525634.1	161	LOC100795885	eukaryota>viridiplantae	Glycine	max	PREDICTED: probable calcium-binding protein CML15 [Glycine max]
XP_016511235.1	161	LOC107828445	eukaryota>viridiplantae	Nicotiana	tabacum	PREDICTED: putative calcium-binding protein CML19 [Nicotiana tabacum]
OQV19215.1	161	BV898_06850	eukaryota>metazoa	Hypsibius	dujardini	putative Calmodulin [Hypsibius dujardini]
XP_003547855.1	160	LOC100797705	eukaryota>viridiplantae	Glycine	max	PREDICTED: probable calcium-binding protein CML16 [Glycine max]
XP_016455993.1	160	LOC107779999	eukaryota>viridiplantae	Nicotiana	tabacum	PREDICTED: probable calcium-binding protein CML15 [Nicotiana tabacum]
XP_016493556.1	160	LOC107812891	eukaryota>viridiplantae	Nicotiana	tabacum	PREDICTED: probable calcium-binding protein CML15 [Nicotiana tabacum]
XP_002970730.1	160	SELMODRAFT_94292	eukaryota>viridiplantae	Selaginella	moellendorffii	hypothetical protein SELMODRAFT_94292 [Selaginella moellendorffii]
CDS38034.1	160	EmuJ_000532700	eukaryota>metazoa	Echinococcus	multilocularis	calmodulin-related protein [Echinococcus multilocularis]
EUB55563.1	160	EGR_09570	eukaryota>metazoa	Echinococcus	granulosus	Calmodulin [Echinococcus granulosus]
XP_002274532.1	160	LOC100247540	eukaryota>viridiplantae	Vitis	vinifera	PREDICTED: probable calcium-binding protein CML15 [Vitis vinifera]
XP_003528258.1	160	LOC100790108	eukaryota>viridiplantae	Glycine	max	PREDICTED: probable calcium-binding protein CML15 [Glycine max]
NP_001151507.1	160	LOC100285141	eukaryota>viridiplantae	Zea	mays	calmodulin [Zea mays]
XP_002124710.1	160	LOC100186502	eukaryota>metazoa>chordata	Ciona	intestinalis	PREDICTED: calmodulin-beta-like [Ciona intestinalis]
XP_002165178.1	160	LOC100212129	eukaryota>metazoa>cnidaria	Hydra	vulgaris	PREDICTED: neo-calmodulin-like isoform X5 [Hydra vulgaris]
XP_009014331.1	160	HELRODRAFT_170263	eukaryota>metazoa>annelida	Helobdella	robusta	hypothetical protein HELRODRAFT_170263 [Helobdella robusta]
XP_009065760.1	160	LOTGIDRAFT_133270	eukaryota>metazoa>mollusca	Lottia	gigantea	hypothetical protein LOTGIDRAFT_133270, partial [Lottia gigantea]
XP_011416026.1	160	LOC105319979	eukaryota>metazoa>mollusca	Crassostrea	gigas	PREDICTED: calmodulin-beta-like [Crassostrea gigas]
XP_018651491.1	160	Smp_018940	eukaryota>metazoa	Schistosoma	mansoni	putative calmodulin [Schistosoma mansoni]
Uram1000008849	160	Uram1000008849	eukaryota>fungi>mucoromycotina	Umbelopsis	ramanniana	>Uram1000008849
EFX70616.1	160	EIP	eukaryota>metazoa>crustacea	Daphnia	pulex	ecdysone-induced protein [Daphnia pulex]
NP_001185330.1	159	ACAM-4,calmodulin,4,CALMODULIN,4,T27F4.1,T27F4_1	eukaryota>viridiplantae	Arabidopsis	thaliana	calmodulin 4 [Arabidopsis thaliana]
XP_019923294.1	159	LOC105329480	eukaryota>metazoa>mollusca	Crassostrea	gigas	PREDICTED: calmodulin-A-like isoform X1 [Crassostrea gigas]
XP_005704341.1	159	Gasu_46430	eukaryota>rhodophyta	Galdieria	sulphuraria	calmodulin isoform 1 [Galdieria sulphuraria]
NP_001151813.1	159	LOC100285448	eukaryota>viridiplantae	Zea	mays	EF hand family protein [Zea mays]
NP_508818.1	159	F43C9.2	eukaryota>metazoa>nematoda	Caenorhabditis	elegans	Uncharacterized protein CELE_F43C9.2 [Caenorhabditis elegans]
XP_002404770.1	159	IscW_ISCW015592	eukaryota>metazoa	Ixodes	scapularis	calmodulin, putative [Ixodes scapularis]
XP_006821627.1	159	LOC102809615	eukaryota>metazoa>hemichordata	Saccoglossus	kowalevskii	PREDICTED: calmodulin-like [Saccoglossus kowalevskii]
XP_011434020.1	159	LOC105332960	eukaryota>metazoa>mollusca	Crassostrea	gigas	PREDICTED: calmodulin-A isoform X1 [Crassostrea gigas]
XP_011449948.1	159	LOC105344067	eukaryota>metazoa>mollusca	Crassostrea	gigas	PREDICTED: calmodulin-A [Crassostrea gigas]
Bcir1000000009	159	Bcir1000000009	eukaryota>fungi>mucoromycotina	Backusella	circina	>Bcir1000000009
Caps1000019680	159	Caps1000019680	eukaryota>metazoa>annelida	Capitella	spI	>Caps1000019680
Crev1000003810	159	Crev1000003810	eukaryota>fungi>kickxellomycotina	Coemansia	reversa	>Crev1000003810
ORX89043.1	159	K493DRAFT_326325	eukaryota>fungi	Basidiobolus	meristosporus CBS 931.73	EF-hand [Basidiobolus meristosporus CBS 931.73]
ORY37136.1	159	LY90DRAFT_661966	eukaryota>fungi>chytridiomycota	Neocallimastix	californiae	EF-hand [Neocallimastix californiae]
XP_016441174.1	158	LOC107766818	eukaryota>viridiplantae	Nicotiana	tabacum	PREDICTED: calcium-binding protein CML24-like [Nicotiana tabacum]
XP_016449529.1	158	LOC107774495	eukaryota>viridiplantae	Nicotiana	tabacum	PREDICTED: probable calcium-binding protein CML18 [Nicotiana tabacum]
KFH62663.1	158	MVEG_12055	eukaryota>fungi	Mortierella	verticillata NRRL 6337	hypothetical protein MVEG_12055 [Mortierella verticillata NRRL 6337]
OMH80684.1	158	AX774_g5870	eukaryota>fungi	Zancudomyces	culisetae	Calmodulin [Zancudomyces culisetae]
OMJ84413.1	158	SteCoe_14489	eukaryota>alveolata>ciliophora	Stentor	coeruleus	hypothetical protein SteCoe_14489 [Stentor coeruleus]
XP_001027948.1	158	TTHERM_00497600	eukaryota>alveolata>ciliophora	Tetrahymena	thermophila SB210	EF-hand pair protein [Tetrahymena thermophila SB210]
XP_001744898.1	158	MONBRDRAFT_24630	eukaryota>choanoflagellida	Monosiga	brevicollis MX1	hypothetical protein [Monosiga brevicollis MX1]
XP_001861617.1	158	CpipJ_CPIJ011379	eukaryota>metazoa>hexapoda	Culex	quinquefasciatus	calmodulin [Culex quinquefasciatus]
XP_002593253.1	158	BRAFLDRAFT_87234	eukaryota>metazoa>chordata	Branchiostoma	floridae	hypothetical protein BRAFLDRAFT_87234 [Branchiostoma floridae]
XP_003632229.1	158	LOC100261610	eukaryota>viridiplantae	Vitis	vinifera	PREDICTED: calmodulin-7 isoform X2 [Vitis vinifera]
XP_006819032.1	158	LOC100369331	eukaryota>metazoa>hemichordata	Saccoglossus	kowalevskii	PREDICTED: calmodulin-like [Saccoglossus kowalevskii]
XP_012757958.1	158	SAMD00019534_019130	eukaryota>amoebozoa>mycetozoa>dictyosteliida	Acytostelium	subglobosum LB1	hypothetical protein SAMD00019534_019130 [Acytostelium subglobosum LB1]
XP_013777284.1	158	LOC106461961	eukaryota>metazoa	Limulus	polyphemus	PREDICTED: calmodulin-A-like [Limulus polyphemus]
XP_019925477.1	158	LOC105334733	eukaryota>metazoa>mollusca	Crassostrea	gigas	PREDICTED: calmodulin isoform X1 [Crassostrea gigas]
Bnat1000021401	158	Bnat1000021401	eukaryota>rhizaria	Bigelowiella	natans	>Bnat1000021401
XP_016434740.1	157	LOC107761087	eukaryota>viridiplantae	Nicotiana	tabacum	PREDICTED: probable calcium-binding protein CML23 [Nicotiana tabacum]
XP_016437007.1	157	LOC107763092	eukaryota>viridiplantae	Nicotiana	tabacum	PREDICTED: probable calcium-binding protein CML23 [Nicotiana tabacum]
EFX77418.1	157	CAM2	eukaryota>metazoa>crustacea	Daphnia	pulex	calmodulin-like protein [Daphnia pulex]
XP_001620254.1	157	NEMVEDRAFT_v1g223299	eukaryota>metazoa>cnidaria	Nematostella	vectensis	hypothetical protein NEMVEDRAFT_v1g223299 [Nematostella vectensis]
XP_002112230.1	157	TRIADDRAFT_24722	eukaryota>metazoa>placozoa	Trichoplax	adhaerens	hypothetical protein TRIADDRAFT_24722 [Trichoplax adhaerens]
XP_002405159.1	157	IscW_ISCW009217	eukaryota>metazoa	Ixodes	scapularis	calmodulin, putative [Ixodes scapularis]
XP_002678523.1	157	NAEGRDRAFT_32696	eukaryota>heterolobosea	Naegleria	gruberi strain NEG-M	predicted protein [Naegleria gruberi strain NEG-M]
XP_004998277.1	157	PTSG_00808	eukaryota>choanoflagellida	Salpingoeca	rosetta	hypothetical protein PTSG_00808 [Salpingoeca rosetta]
XP_011427024.1	157	LOC105328005	eukaryota>metazoa>mollusca	Crassostrea	gigas	PREDICTED: calmodulin-A-like isoform X1 [Crassostrea gigas]
XP_011427026.1	157	LOC105328008	eukaryota>metazoa>mollusca	Crassostrea	gigas	PREDICTED: calmodulin-A [Crassostrea gigas]
XP_013431669.1	157	M436DRAFT_35400	eukaryota>fungi>ascomycota	Aureobasidium	namibiae CBS 147.97	calmodulin-like protein [Aureobasidium namibiae CBS 147.97]
XP_017173787.1	157	2410038D05Rik	eukaryota>metazoa>chordata>vertebrata	Mus	musculus	PREDICTED: calcium-binding protein 4 isoform X1 [Mus musculus]
NP_173288.1	157	F25I16.13,F25I16_13	eukaryota>viridiplantae	Arabidopsis	thaliana	EF hand calcium-binding protein family [Arabidopsis thaliana]
NP_564874.1	157	calmodulin,like,23,T27F4.15,T27F4_15	eukaryota>viridiplantae	Arabidopsis	thaliana	calmodulin like 23 [Arabidopsis thaliana]
XP_016443374.1	157	LOC107768745	eukaryota>viridiplantae	Nicotiana	tabacum	PREDICTED: calcium-binding allergen Ole e 8-like [Nicotiana tabacum]
XP_021880457.1	157	BCR41DRAFT_337686	eukaryota>fungi	Lobosporangium	transversale	calmodulin [Lobosporangium transversale]
XP_021885586.1	157	BCR41DRAFT_346175	eukaryota>fungi	Lobosporangium	transversale	calmodulin-like protein 5 [Lobosporangium transversale]
OAD07167.1	157	MUCCIDRAFT_155487	eukaryota>fungi	Mucor	circinelloides f. lusitanicus CBS 277.49	hypothetical protein MUCCIDRAFT_155487 [Mucor circinelloides f. lusitanicus CBS 277.49]
XP_012648635.1	156	BBM_III00120	eukaryota>alveolata>apicomplexa	Babesia	microti strain RI	uncharacterized protein [Babesia microti strain RI]
XP_006357771.1	156	LOC102593516	eukaryota>viridiplantae	Solanum	tuberosum	PREDICTED: calcium-binding protein CML24-like [Solanum tuberosum]
XP_015299313.1	156	CALML6	eukaryota>metazoa>chordata>vertebrata	Macaca	fascicularis	PREDICTED: calmodulin-like protein 6 isoform X4 [Macaca fascicularis]
XP_001177619.1	156	LOC591003	eukaryota>metazoa>echinodermata	Strongylocentrotus	purpuratus	PREDICTED: calmodulin isoform X1 [Strongylocentrotus purpuratus]
XP_017177786.1	156	Cabp5	eukaryota>metazoa>chordata>vertebrata	Mus	musculus	PREDICTED: calcium-binding protein 5 isoform X1 [Mus musculus]
XP_017177787.1	156	Cabp5	eukaryota>metazoa>chordata>vertebrata	Mus	musculus	PREDICTED: calcium-binding protein 5 isoform X1 [Mus musculus]
Lhya1000008663	156	Lhya1000008663	eukaryota>fungi>mucoromycotina	Lichtheimia	hyalospora	>Lhya1000008663
Uram1000002140	156	Uram1000002140	eukaryota>fungi>mucoromycotina	Umbelopsis	ramanniana	>Uram1000002140
EPB83340.1	156	HMPREF1544_09930	eukaryota>fungi	Mucor	circinelloides f. circinelloides 1006PhL	calmodulin [Mucor circinelloides f. circinelloides 1006PhL]
NP_508864.2	156	cal-5	eukaryota>metazoa>nematoda	Caenorhabditis	elegans	CALmodulin related genes [Caenorhabditis elegans]
XP_002671503.1	156	NAEGRDRAFT_83288	eukaryota>heterolobosea	Naegleria	gruberi strain NEG-M	predicted protein [Naegleria gruberi strain NEG-M]
XP_004027517.1	156	IMG5_181770	eukaryota>alveolata>ciliophora	Ichthyophthirius	multifiliis	hypothetical protein IMG5_181770 [Ichthyophthirius multifiliis]
XP_006822821.1	156	LOC100367142	eukaryota>metazoa>hemichordata	Saccoglossus	kowalevskii	PREDICTED: calmodulin-like [Saccoglossus kowalevskii]
XP_011434023.1	156	LOC105332960	eukaryota>metazoa>mollusca	Crassostrea	gigas	PREDICTED: calmodulin-A isoform X2 [Crassostrea gigas]
XP_012554755.1	156	LOC100212129	eukaryota>metazoa>cnidaria	Hydra	vulgaris	PREDICTED: neo-calmodulin-like isoform X6 [Hydra vulgaris]
XP_019024813.1	156	SAICODRAFT_24917	eukaryota>fungi>ascomycota	Saitoella	complicata NRRL Y-17804	calmodulin B [Saitoella complicata NRRL Y-17804]
XP_019922692.1	156	LOC105328002	eukaryota>metazoa>mollusca	Crassostrea	gigas	PREDICTED: calmodulin-A isoform X1 [Crassostrea gigas]
CAG04763.1	156	GSTEN:00024594:G:001	eukaryota>metazoa>chordata>vertebrata>actinopterygii	Tetraodon	nigroviridis	unnamed protein product, partial [Tetraodon nigroviridis]
XP_018267609.1	155	RHOBADRAFT_56596	eukaryota>fungi>basidiomycota	Rhodotorula	graminis WP1	calmodulin [Rhodotorula graminis WP1]
Bnat1000006505	155	Bnat1000006505	eukaryota>rhizaria	Bigelowiella	natans	>Bnat1000006505
XP_015750025.1	155	LOC107329887	eukaryota>metazoa>cnidaria	Acropora	digitifera	PREDICTED: neo-calmodulin-like [Acropora digitifera]
XP_015770829.1	155	LOC107349229	eukaryota>metazoa>cnidaria	Acropora	digitifera	PREDICTED: neo-calmodulin-like [Acropora digitifera]
XP_015770830.1	155	LOC107349229	eukaryota>metazoa>cnidaria	Acropora	digitifera	PREDICTED: neo-calmodulin-like [Acropora digitifera]
XP_015770831.1	155	LOC107349229	eukaryota>metazoa>cnidaria	Acropora	digitifera	PREDICTED: neo-calmodulin-like [Acropora digitifera]
EPY31553.1	155	STCU_03398	eukaryota>euglenozoa>kinetoplastida	Strigomonas	culicis	calmodulin [Strigomonas culicis]
EPY32920.1	155	STCU_02576	eukaryota>euglenozoa>kinetoplastida	Strigomonas	culicis	calmodulin [Strigomonas culicis]
EPY34608.1	155	STCU_01494	eukaryota>euglenozoa>kinetoplastida	Strigomonas	culicis	calmodulin [Strigomonas culicis]
XP_001469648.1	155	LINJ_36_3860	eukaryota>euglenozoa>kinetoplastida	Leishmania	infantum JPCM5	putative calmodulin [Leishmania infantum JPCM5]
XP_001686950.1	155	LMJF_36_3675	eukaryota>euglenozoa>kinetoplastida	Leishmania	major strain Friedlin	putative calmodulin [Leishmania major strain Friedlin]
XP_003865500.1	155	LDBPK_363860	eukaryota>euglenozoa>kinetoplastida	Leishmania	donovani	calmodulin, putative [Leishmania donovani]
XP_002683533.1	155	NAEGRDRAFT_55564	eukaryota>heterolobosea	Naegleria	gruberi strain NEG-M	flagellar calmodulin [Naegleria gruberi strain NEG-M]
XP_001569010.1	155	LBRM_35_3910	eukaryota>euglenozoa>kinetoplastida	Leishmania	braziliensis MHOM/BR/75/M2904	putative calmodulin [Leishmania braziliensis MHOM/BR/75/M2904]
XP_010703313.1	155	LPMP_353780	eukaryota>euglenozoa>kinetoplastida	Leishmania	panamensis	calmodulin, putative [Leishmania panamensis]
Caps1000002976	155	Caps1000002976	eukaryota>metazoa>annelida	Capitella	spI	>Caps1000002976
CBN74265.1	155	Esi_0019_0013	eukaryota>stramenopiles	Ectocarpus	siliculosus	calmodulin [Ectocarpus siliculosus]
KPI85675.1	155	ABL78_5255	eukaryota>euglenozoa>kinetoplastida	Leptomonas	seymouri	putative calmodulin [Leptomonas seymouri]
KXJ24794.1	155	AC249_AIPGENE20951	eukaryota>metazoa>cnidaria	Exaiptasia	pallida	Calmodulin [Exaiptasia pallida]
NP_001187945.1	155	calgl	eukaryota>metazoa>chordata>vertebrata>actinopterygii	Ictalurus	punctatus	calmodulin-like protein 6 [Ictalurus punctatus]
XP_001310191.1	155	TVAG_037680	eukaryota>parabasalia	Trichomonas	vaginalis G3	EF hand family protein [Trichomonas vaginalis G3]
XP_002129217.1	155	LOC100181538	eukaryota>metazoa>chordata	Ciona	intestinalis	PREDICTED: calglandulin-like [Ciona intestinalis]
XP_002610052.1	155	BRAFLDRAFT_238039	eukaryota>metazoa>chordata	Branchiostoma	floridae	hypothetical protein BRAFLDRAFT_238039, partial [Branchiostoma floridae]
XP_002734093.1	155	LOC100371717	eukaryota>metazoa>hemichordata	Saccoglossus	kowalevskii	PREDICTED: calmodulin, striated muscle-like [Saccoglossus kowalevskii]
XP_002736799.1	155	LOC100367075	eukaryota>metazoa>hemichordata	Saccoglossus	kowalevskii	PREDICTED: calmodulin-B-like [Saccoglossus kowalevskii]
XP_006819034.1	155	LOC102806135	eukaryota>metazoa>hemichordata	Saccoglossus	kowalevskii	PREDICTED: calmodulin-like [Saccoglossus kowalevskii]
XP_011413059.1	155	LOC105317937	eukaryota>metazoa>mollusca	Crassostrea	gigas	PREDICTED: calmodulin [Crassostrea gigas]
XP_012554756.1	155	LOC100212129	eukaryota>metazoa>cnidaria	Hydra	vulgaris	PREDICTED: neo-calmodulin-like isoform X7 [Hydra vulgaris]
XP_013761197.1	155	AMSG_02176	eukaryota>apusozoa>apusomonadidae	Thecamonas	trahens ATCC 50062	calmodulin mutant syncam9 [Thecamonas trahens ATCC 50062]
XP_015659821.1	155	ABB37_03761	eukaryota>euglenozoa>kinetoplastida	Leptomonas	pyrrhocoris	putative calmodulin [Leptomonas pyrrhocoris]
Caps1000006459	155	Caps1000006459	eukaryota>metazoa>annelida	Capitella	spI	>Caps1000006459
Uram1000004473	155	Uram1000004473	eukaryota>fungi>mucoromycotina	Umbelopsis	ramanniana	>Uram1000004473
XP_013400747.1	154	LOC106166637	eukaryota>metazoa	Lingula	anatina	PREDICTED: calmodulin-2/4-like [Lingula anatina]
CBN79612.1	154	Esi_0271_0046	eukaryota>stramenopiles	Ectocarpus	siliculosus	yellow cameleon 2.60 [Ectocarpus siliculosus]
ELR12677.1	154	ACA1_091870	eukaryota>amoebozoa>acanthamoebidae	Acanthamoeba	castellanii str. Neff	calmodulin, putative [Acanthamoeba castellanii str. Neff]
EUB61289.1	154	EGR_03775	eukaryota>metazoa	Echinococcus	granulosus	Calmodulin [Echinococcus granulosus]
KPI85335.1	154	ABL78_5617	eukaryota>euglenozoa>kinetoplastida	Leptomonas	seymouri	calmodulin-like protein [Leptomonas seymouri]
NP_192238.1	154	F4C21.22,F4C21_22	eukaryota>viridiplantae	Arabidopsis	thaliana	EF hand calcium-binding protein family [Arabidopsis thaliana]
NP_611714.1	154	BcDNA:AT13807,CBP8,Dmel\CG13526	eukaryota>metazoa>hexapoda	Drosophila	melanogaster	uncharacterized protein Dmel_CG13526 [Drosophila melanogaster]
OMJ69735.1	154	SteCoe_32460	eukaryota>alveolata>ciliophora	Stentor	coeruleus	hypothetical protein SteCoe_32460 [Stentor coeruleus]
XP_001637998.1	154	NEMVEDRAFT_v1g161862	eukaryota>metazoa>cnidaria	Nematostella	vectensis	predicted protein [Nematostella vectensis]
XP_002128183.1	154	LOC100182008	eukaryota>metazoa>chordata	Ciona	intestinalis	PREDICTED: neo-calmodulin-like [Ciona intestinalis]
XP_002185048.1	154	CaM2	eukaryota>stramenopiles	Phaeodactylum	tricornutum CCAP 1055/1	calmodulin [Phaeodactylum tricornutum CCAP 1055/1]
XP_002605441.1	154	BRAFLDRAFT_278516	eukaryota>metazoa>chordata	Branchiostoma	floridae	hypothetical protein BRAFLDRAFT_278516 [Branchiostoma floridae]
XP_003632448.1	154	LOC100854822	eukaryota>viridiplantae	Vitis	vinifera	PREDICTED: calmodulin-like protein 3 [Vitis vinifera]
XP_006823621.1	154	LOC102802805	eukaryota>metazoa>hemichordata	Saccoglossus	kowalevskii	PREDICTED: calmodulin-like [Saccoglossus kowalevskii]
XP_008665400.1	154	LOC606468	eukaryota>viridiplantae	Zea	mays	PREDICTED: calmodulin-like protein 4 [Zea mays]
XP_011412829.1	154	LOC105317800	eukaryota>metazoa>mollusca	Crassostrea	gigas	PREDICTED: calmodulin-alpha-like [Crassostrea gigas]
XP_011663943.1	154	LOC593629	eukaryota>metazoa>echinodermata	Strongylocentrotus	purpuratus	PREDICTED: neo-calmodulin [Strongylocentrotus purpuratus]
XP_013400713.1	154	LOC106166584	eukaryota>metazoa	Lingula	anatina	PREDICTED: neo-calmodulin-like isoform X7 [Lingula anatina]
XP_013402352.1	154	LOC106167983	eukaryota>metazoa	Lingula	anatina	PREDICTED: neo-calmodulin-like [Lingula anatina]
XP_014776698.1	154	LOC106873729	eukaryota>metazoa>mollusca	Octopus	bimaculoides	PREDICTED: neo-calmodulin-like isoform X1 [Octopus bimaculoides]
XP_014781511.1	154	LOC106877186	eukaryota>metazoa>mollusca	Octopus	bimaculoides	PREDICTED: neo-calmodulin-like isoform X1 [Octopus bimaculoides]
XP_016443585.1	154	LOC107768927	eukaryota>viridiplantae	Nicotiana	tabacum	PREDICTED: calmodulin-like protein 3 [Nicotiana tabacum]
XP_018655558.1	154	Smp_026560.1	eukaryota>metazoa	Schistosoma	mansoni	putative calmodulin [Schistosoma mansoni]
XP_019925478.1	154	LOC109619586	eukaryota>metazoa>mollusca	Crassostrea	gigas	PREDICTED: calmodulin-like [Crassostrea gigas]
Caps1000025386	154	Caps1000025386	eukaryota>metazoa>annelida	Capitella	spI	>Caps1000025386
Lhya1000007009	154	Lhya1000007009	eukaryota>fungi>mucoromycotina	Lichtheimia	hyalospora	>Lhya1000007009
Smed1000001300	154	Smed1000001300	eukaryota>metazoa>platyhelminthe	Schmidtea	mediterranea	>Smed1000001300
XP_006365410.1	154	LOC102587258	eukaryota>viridiplantae	Solanum	tuberosum	PREDICTED: calcium-binding allergen Ole e 8-like [Solanum tuberosum]
XP_016493415.1	154	LOC107812761	eukaryota>viridiplantae	Nicotiana	tabacum	PREDICTED: calcium-binding allergen Ole e 8-like [Nicotiana tabacum]
XP_014666517.1	153	LOC106808343	eukaryota>metazoa	Priapulus	caudatus	PREDICTED: calmodulin-A-like isoform X2 [Priapulus caudatus]
NP_001124246.1	153	zgc:193500	eukaryota>metazoa>chordata>vertebrata>actinopterygii	Danio	rerio	calmodulin-like protein 4 [Danio rerio]
NP_001188172.1	153	call4	eukaryota>metazoa>chordata>vertebrata>actinopterygii	Ictalurus	punctatus	calmodulin-like protein 4 [Ictalurus punctatus]
NP_001264558.1	153	CALML4	eukaryota>metazoa>chordata>vertebrata	Gallus	gallus	calmodulin-like protein 4 [Gallus gallus]
NP_612177.1	153	2010002G05Rik	eukaryota>metazoa>chordata>vertebrata	Mus	musculus	calmodulin-like protein 4 isoform a [Mus musculus]
XP_011608263.1	153	calml4	eukaryota>metazoa>chordata>vertebrata>actinopterygii	Takifugu	rubripes	PREDICTED: calmodulin-like protein 4 isoform X3 [Takifugu rubripes]
XP_018110726.1	153	LOC108712798	eukaryota>metazoa>chordata>vertebrata	Xenopus	laevis	PREDICTED: calmodulin-like protein 4 [Xenopus laevis]
XP_006356103.1	153	LOC102587410	eukaryota>viridiplantae	Solanum	tuberosum	PREDICTED: calmodulin-2/4 [Solanum tuberosum]
EST48754.1	153	SS50377_11076	eukaryota>diplomonadida	Spironucleus	salmonicida	Calmodulin [Spironucleus salmonicida]
ESU41092.1	153	GSB_5333	eukaryota>diplomonadida	Giardia	intestinalis	Calcium-binding protein, EF-Hand superfamily protein [Giardia intestinalis]
XP_001705820.1	153	GL50803_5333	eukaryota>diplomonadida	Giardia	lamblia ATCC 50803	Calmodulin [Giardia lamblia ATCC 50803]
XP_002605572.1	153	BRAFLDRAFT_266760	eukaryota>metazoa>chordata	Branchiostoma	floridae	hypothetical protein BRAFLDRAFT_266760 [Branchiostoma floridae]
XP_002605578.1	153	BRAFLDRAFT_266773	eukaryota>metazoa>chordata	Branchiostoma	floridae	hypothetical protein BRAFLDRAFT_266773 [Branchiostoma floridae]
CBY11950.1	153	GSOID_T00017359001	eukaryota>metazoa>chordata	Oikopleura	dioica	unnamed protein product [Oikopleura dioica]
CCW69259.1	153	GSHART1_T00006414001	eukaryota>euglenozoa>kinetoplastida	Phytomonas	sp. isolate Hart1	unnamed protein product [Phytomonas sp. isolate Hart1]
NP_187405.1	153	ARF-GAP,domain,11,AtCML3,calmodulin-like,3,CML3	eukaryota>viridiplantae	Arabidopsis	thaliana	ARF-GAP domain 11 [Arabidopsis thaliana]
XP_001326924.1	153	TVAG_038070	eukaryota>parabasalia	Trichomonas	vaginalis G3	calmodulin [Trichomonas vaginalis G3]
XP_001625680.1	153	NEMVEDRAFT_v1g216465	eukaryota>metazoa>cnidaria	Nematostella	vectensis	predicted protein [Nematostella vectensis]
XP_002295754.1	153	THAPS_23399	eukaryota>stramenopiles	Thalassiosira	pseudonana CCMP1335	predicted protein [Thalassiosira pseudonana CCMP1335]
XP_002672922.1	153	NAEGRDRAFT_72063	eukaryota>heterolobosea	Naegleria	gruberi strain NEG-M	predicted protein [Naegleria gruberi strain NEG-M]
XP_009012944.1	153	HELRODRAFT_109943	eukaryota>metazoa>annelida	Helobdella	robusta	hypothetical protein HELRODRAFT_109943 [Helobdella robusta]
XP_009053694.1	153	LOTGIDRAFT_227222	eukaryota>metazoa>mollusca	Lottia	gigantea	hypothetical protein LOTGIDRAFT_227222 [Lottia gigantea]
XP_009058463.1	153	LOTGIDRAFT_122753	eukaryota>metazoa>mollusca	Lottia	gigantea	hypothetical protein LOTGIDRAFT_122753, partial [Lottia gigantea]
XP_011131521.1	153	GNI_111450	eukaryota>alveolata>apicomplexa	Gregarina	niphandrodes	calmodulin [Gregarina niphandrodes]
XP_011412828.1	153	LOC105317799	eukaryota>metazoa>mollusca	Crassostrea	gigas	PREDICTED: calmodulin-A-like [Crassostrea gigas]
XP_012546645.1	153	LOC692927	eukaryota>metazoa>hexapoda	Bombyx	mori	calmodulin isoform X1 [Bombyx mori]
XP_015755717.1	153	LOC107335234	eukaryota>metazoa>cnidaria	Acropora	digitifera	PREDICTED: calmodulin-A-like [Acropora digitifera]
XP_016455014.1	153	LOC107779166	eukaryota>viridiplantae	Nicotiana	tabacum	PREDICTED: calmodulin-7-like isoform X3 [Nicotiana tabacum]
XP_019080322.1	153	LOC100241881	eukaryota>viridiplantae	Vitis	vinifera	PREDICTED: calmodulin [Vitis vinifera]
Caps1000000304	153	Caps1000000304	eukaryota>metazoa>annelida	Capitella	spI	>Caps1000000304
Caps1000009436	153	Caps1000009436	eukaryota>metazoa>annelida	Capitella	spI	>Caps1000009436
Caps1000019815	153	Caps1000019815	eukaryota>metazoa>annelida	Capitella	spI	>Caps1000019815
XP_001763426.1	153	PHYPADRAFT_163851	eukaryota>viridiplantae	Physcomitrella	patens	predicted protein [Physcomitrella patens]
OAC98495.1	153	MUCCIDRAFT_150169	eukaryota>fungi	Mucor	circinelloides f. lusitanicus CBS 277.49	hypothetical protein MUCCIDRAFT_150169 [Mucor circinelloides f. lusitanicus CBS 277.49]
XP_011429056.1	152	LOC105329480	eukaryota>metazoa>mollusca	Crassostrea	gigas	PREDICTED: calmodulin-A-like isoform X2 [Crassostrea gigas]
XP_018108808.1	152	LOC108711508	eukaryota>metazoa>chordata>vertebrata	Xenopus	laevis	PREDICTED: calmodulin-like protein 4 [Xenopus laevis]
NP_193022.1	152	T20K18.210,T20K18_210,unfertilized,embryo,sac,14	eukaryota>viridiplantae	Arabidopsis	thaliana	EF hand calcium-binding protein family [Arabidopsis thaliana]
XP_016440139.1	152	LOC107765954	eukaryota>viridiplantae	Nicotiana	tabacum	PREDICTED: calmodulin-like protein 3 [Nicotiana tabacum]
XP_016450622.1	152	LOC107775410	eukaryota>viridiplantae	Nicotiana	tabacum	PREDICTED: calmodulin-like protein 3 [Nicotiana tabacum]
XP_009310096.1	152	DQ04_02381030	eukaryota>euglenozoa>kinetoplastida	Trypanosoma	grayi	calmodulin [Trypanosoma grayi]
XP_811578.1	152	Tc00.1047053506963.90	eukaryota>euglenozoa>kinetoplastida	Trypanosoma	cruzi strain CL Brener	calmodulin [Trypanosoma cruzi strain CL Brener]
XP_815818.1	152	Tc00.1047053511233.80	eukaryota>euglenozoa>kinetoplastida	Trypanosoma	cruzi strain CL Brener	calmodulin [Trypanosoma cruzi strain CL Brener]
EPY39669.1	152	AGDE_04259	eukaryota>euglenozoa>kinetoplastida	Angomonas	deanei	calmodulin [Angomonas deanei]
EPY42609.1	152	AGDE_01314	eukaryota>euglenozoa>kinetoplastida	Angomonas	deanei	calmodulin [Angomonas deanei]
XP_002965450.1	152	SELMODRAFT_83841	eukaryota>viridiplantae	Selaginella	moellendorffii	hypothetical protein SELMODRAFT_83841 [Selaginella moellendorffii]
XP_002984541.1	152	SELMODRAFT_120223	eukaryota>viridiplantae	Selaginella	moellendorffii	hypothetical protein SELMODRAFT_120223 [Selaginella moellendorffii]
XP_002984822.1	152	SELMODRAFT_181303	eukaryota>viridiplantae	Selaginella	moellendorffii	hypothetical protein SELMODRAFT_181303 [Selaginella moellendorffii]
XP_002985880.1	152	SELMODRAFT_157693	eukaryota>viridiplantae	Selaginella	moellendorffii	hypothetical protein SELMODRAFT_157693 [Selaginella moellendorffii]
XP_003554123.1	152	LOC100806637	eukaryota>viridiplantae	Glycine	max	PREDICTED: calmodulin-like protein 3 [Glycine max]
XP_006576779.1	152	LOC100798114	eukaryota>viridiplantae	Glycine	max	PREDICTED: calmodulin-like protein 3 [Glycine max]
XP_012353498.1	152	CALM1	eukaryota>metazoa>chordata>vertebrata	Nomascus	leucogenys	PREDICTED: calmodulin isoform X1 [Nomascus leucogenys]
XP_013782320.1	152	LOC106466573	eukaryota>metazoa	Limulus	polyphemus	calcium-binding protein E63-1-like [Limulus polyphemus]
XP_013789191.1	152	LOC106473059	eukaryota>metazoa	Limulus	polyphemus	calcium-binding protein E63-1-like [Limulus polyphemus]
CEP02126.1	152	PBRA_002391	eukaryota>rhizaria>cercozoa>plasmodiophorida	Plasmodiophora	brassicae	hypothetical protein PBRA_002391 [Plasmodiophora brassicae]
OMJ85133.1	152	SteCoe_13599	eukaryota>alveolata>ciliophora	Stentor	coeruleus	hypothetical protein SteCoe_13599 [Stentor coeruleus]
XP_001640841.1	152	NEMVEDRAFT_v1g178268	eukaryota>metazoa>cnidaria	Nematostella	vectensis	predicted protein [Nematostella vectensis]
XP_004184032.1	152	EIN_173610	eukaryota>amoebozoa>entamoebidae	Entamoeba	invadens IP1	calmodulin, putative [Entamoeba invadens IP1]
XP_004206918.1	152	LOC100210377	eukaryota>metazoa>cnidaria	Hydra	vulgaris	PREDICTED: calmodulin-like [Hydra vulgaris]
XP_006679011.1	152	BATDEDRAFT_25085	eukaryota>fungi>chytridiomycota	Batrachochytrium	dendrobatidis JAM81	hypothetical protein BATDEDRAFT_25085 [Batrachochytrium dendrobatidis JAM81]
XP_008656412.1	152	LOC103635808	eukaryota>viridiplantae	Zea	mays	probable calcium-binding protein CML9 [Zea mays]
XP_009049302.1	152	LOTGIDRAFT_203644	eukaryota>metazoa>mollusca	Lottia	gigantea	hypothetical protein LOTGIDRAFT_203644 [Lottia gigantea]
XP_009064501.1	152	LOTGIDRAFT_204683	eukaryota>metazoa>mollusca	Lottia	gigantea	hypothetical protein LOTGIDRAFT_204683 [Lottia gigantea]
XP_011416019.1	152	LOC105319972	eukaryota>metazoa>mollusca	Crassostrea	gigas	PREDICTED: neo-calmodulin-like isoform X2 [Crassostrea gigas]
XP_011418915.2	152	LOC105322083	eukaryota>metazoa>mollusca	Crassostrea	gigas	PREDICTED: calmodulin-beta-like [Crassostrea gigas]
XP_504759.1	152	YALI0_E34111g	eukaryota>fungi>ascomycota	Yarrowia	lipolytica CLIB122	YALI0E34111p [Yarrowia lipolytica CLIB122]
XP_641695.1	152	calA	eukaryota>amoebozoa>mycetozoa>dictyosteliida	Dictyostelium	discoideum AX4	hypothetical protein DDB_G0279407 [Dictyostelium discoideum AX4]
Crev1000004668	152	Crev1000004668	eukaryota>fungi>kickxellomycotina	Coemansia	reversa	>Crev1000004668
Pisp1000006470	152	Pisp1000006470	eukaryota>fungi>neocallimastigomycota	Piromyces	sp	>Pisp1000006470
XP_003550642.1	152	LOC100814980	eukaryota>viridiplantae	Glycine	max	PREDICTED: probable calcium-binding protein CML26 [Glycine max]
XP_014617716.1	152	LOC100818136	eukaryota>viridiplantae	Glycine	max	PREDICTED: probable calcium-binding protein CML25 [Glycine max]
OQV17184.1	152	BV898_08762	eukaryota>metazoa	Hypsibius	dujardini	Troponin C [Hypsibius dujardini]
CDW91107.1	151	Contig17513.g18635	eukaryota>alveolata>ciliophora	Stylonychia	lemnae	calmodulin [Stylonychia lemnae]
EJY80148.1	151	OXYTRI_22570	eukaryota>alveolata>ciliophora	Oxytricha	trifallax	Calmodulin (macronuclear) [Oxytricha trifallax]
XP_002129194.1	151	LOC100184552	eukaryota>metazoa>chordata	Ciona	intestinalis	PREDICTED: calmodulin-like [Ciona intestinalis]
XP_002611701.1	151	BRAFLDRAFT_56804	eukaryota>metazoa>chordata	Branchiostoma	floridae	hypothetical protein BRAFLDRAFT_56804 [Branchiostoma floridae]
XP_003293234.1	151	DICPUDRAFT_93011	eukaryota>amoebozoa>mycetozoa>dictyosteliida	Dictyostelium	purpureum	calmodulin [Dictyostelium purpureum]
XP_005648308.1	151	COCSUDRAFT_23637	eukaryota>viridiplantae>chlorophyta	Coccomyxa	subellipsoidea C-169	EF-hand [Coccomyxa subellipsoidea C-169]
XP_009013108.1	151	HELRODRAFT_110019	eukaryota>metazoa>annelida	Helobdella	robusta	hypothetical protein HELRODRAFT_110019 [Helobdella robusta]
XP_014780626.1	151	LOC106876551	eukaryota>metazoa>mollusca	Octopus	bimaculoides	PREDICTED: neo-calmodulin-like [Octopus bimaculoides]
Crev1000002305	151	Crev1000002305	eukaryota>fungi>kickxellomycotina	Coemansia	reversa	>Crev1000002305
XP_008621504.1	151	SDRG_17050	eukaryota>stramenopiles	Saprolegnia	diclina VS20	hypothetical protein SDRG_17050 [Saprolegnia diclina VS20]
XP_008864005.1	151	H310_02319	eukaryota>stramenopiles	Aphanomyces	invadans	hypothetical protein H310_02319 [Aphanomyces invadans]
XP_009824355.1	151	H257_02426	eukaryota>stramenopiles	Aphanomyces	astaci	hypothetical protein H257_02426 [Aphanomyces astaci]
XP_012201320.1	151	SPRG_07133	eukaryota>stramenopiles	Saprolegnia	parasitica CBS 223.65	hypothetical protein SPRG_07133 [Saprolegnia parasitica CBS 223.65]
XP_012213561.1	151	SPRG_18727	eukaryota>stramenopiles	Saprolegnia	parasitica CBS 223.65	hypothetical protein SPRG_18727 [Saprolegnia parasitica CBS 223.65]
EPY25972.1	151	AGDE_11605	eukaryota>euglenozoa>kinetoplastida	Angomonas	deanei	calmodulin [Angomonas deanei]
EPY32237.1	151	AGDE_08793	eukaryota>euglenozoa>kinetoplastida	Angomonas	deanei	calmodulin [Angomonas deanei]
EPY40774.1	151	AGDE_03153	eukaryota>euglenozoa>kinetoplastida	Angomonas	deanei	calmodulin [Angomonas deanei]
GAT94789.1	151	CL6EHI_100270	eukaryota>amoebozoa>entamoebidae	Entamoeba	histolytica	calmodulin putative [Entamoeba histolytica]
XP_001734679.1	151	EDI_127110	eukaryota>amoebozoa>entamoebidae	Entamoeba	dispar SAW760	calmodulin [Entamoeba dispar SAW760]
XP_008858434.1	151	ENU1_136850	eukaryota>amoebozoa>entamoebidae	Entamoeba	nuttalli P19	calmodulin, putative [Entamoeba nuttalli P19]
XP_011422879.1	151	LOC105325152	eukaryota>metazoa>mollusca	Crassostrea	gigas	PREDICTED: calmodulin-like [Crassostrea gigas]
XP_011422880.1	151	LOC105325152	eukaryota>metazoa>mollusca	Crassostrea	gigas	PREDICTED: calmodulin-like [Crassostrea gigas]
XP_011437495.1	151	LOC105335363	eukaryota>metazoa>mollusca	Crassostrea	gigas	PREDICTED: calmodulin-like [Crassostrea gigas]
CBY17793.1	151	GSOID_T00016524001	eukaryota>metazoa>chordata	Oikopleura	dioica	unnamed protein product [Oikopleura dioica]
CBY22473.1	151	GSOID_T00013229001	eukaryota>metazoa>chordata	Oikopleura	dioica	unnamed protein product [Oikopleura dioica]
OHS96782.1	151	TRFO_36968	eukaryota>parabasalia	Tritrichomonas	foetus	Calmodulin [Tritrichomonas foetus]
OHT13262.1	151	TRFO_16574	eukaryota>parabasalia	Tritrichomonas	foetus	Calmodulin [Tritrichomonas foetus]
OMJ70083.1	151	SteCoe_32024	eukaryota>alveolata>ciliophora	Stentor	coeruleus	hypothetical protein SteCoe_32024 [Stentor coeruleus]
OMJ72245.1	151	SteCoe_29355	eukaryota>alveolata>ciliophora	Stentor	coeruleus	hypothetical protein SteCoe_29355 [Stentor coeruleus]
XP_013771959.1	151	LOC106457120	eukaryota>metazoa	Limulus	polyphemus	calmodulin-A-like isoform X1 [Limulus polyphemus]
XP_013779341.1	151	LOC106463817	eukaryota>metazoa	Limulus	polyphemus	calmodulin-A-like [Limulus polyphemus]
ELR14478.1	151	ACA1_192080	eukaryota>amoebozoa>acanthamoebidae	Acanthamoeba	castellanii str. Neff	hypothetical protein ACA1_192080 [Acanthamoeba castellanii str. Neff]
NP_190760.1	151	ATCML9,calmodulin,9,CALMODULIN,9,CALMODULIN,LIKE,PROTEIN,9,CML9	eukaryota>viridiplantae	Arabidopsis	thaliana	calmodulin 9 [Arabidopsis thaliana]
NP_193200.1	151	AtCML8,calmodulin,8,CALMODULIN,8,calmodulin-like,8,CML8,DL3360W,FCAALL.157	eukaryota>viridiplantae	Arabidopsis	thaliana	calmodulin 8 [Arabidopsis thaliana]
NP_612058.1	151	CBP7,Dmel\CG13898	eukaryota>metazoa>hexapoda	Drosophila	melanogaster	uncharacterized protein Dmel_CG13898 [Drosophila melanogaster]
NP_999769.1	151	spec2c	eukaryota>metazoa>echinodermata	Strongylocentrotus	purpuratus	calcium-binding protein SPEC 2C [Strongylocentrotus purpuratus]
OAF71029.1	151	A3Q56_01203	eukaryota>metazoa	Intoshia	linei	hypothetical protein A3Q56_01203 [Intoshia linei]
XP_001898823.1	151	Bm1_36880	eukaryota>metazoa>nematoda	Brugia	malayi	EF hand family protein [Brugia malayi]
XP_002593251.1	151	BRAFLDRAFT_115029	eukaryota>metazoa>chordata	Branchiostoma	floridae	hypothetical protein BRAFLDRAFT_115029 [Branchiostoma floridae]
XP_009021868.1	151	HELRODRAFT_83483	eukaryota>metazoa>annelida	Helobdella	robusta	hypothetical protein HELRODRAFT_83483 [Helobdella robusta]
XP_009038061.1	151	AURANDRAFT_27787	eukaryota>stramenopiles	Aureococcus	anophagefferens	hypothetical protein AURANDRAFT_27787 [Aureococcus anophagefferens]
XP_011131952.1	151	GNI_124140	eukaryota>alveolata>apicomplexa	Gregarina	niphandrodes	calmodulin [Gregarina niphandrodes]
XP_011436591.1	151	LOC105334735	eukaryota>metazoa>mollusca	Crassostrea	gigas	PREDICTED: calmodulin-like [Crassostrea gigas]
XP_016494040.1	151	LOC107813304	eukaryota>viridiplantae	Nicotiana	tabacum	PREDICTED: calmodulin-like protein 3 [Nicotiana tabacum]
XP_784808.2	151	LOC579610	eukaryota>metazoa>echinodermata	Strongylocentrotus	purpuratus	PREDICTED: neo-calmodulin [Strongylocentrotus purpuratus]
Lhya1000008046	151	Lhya1000008046	eukaryota>fungi>mucoromycotina	Lichtheimia	hyalospora	>Lhya1000008046
Lhya1000008063	151	Lhya1000008063	eukaryota>fungi>mucoromycotina	Lichtheimia	hyalospora	>Lhya1000008063
Smed1000006476	151	Smed1000006476	eukaryota>metazoa>platyhelminthe	Schmidtea	mediterranea	>Smed1000006476
ORX88165.1	151	K493DRAFT_268493	eukaryota>fungi	Basidiobolus	meristosporus CBS 931.73	calmodulin mutant SYNCAM71A [Basidiobolus meristosporus CBS 931.73]
ORY22310.1	151	LY90DRAFT_430985	eukaryota>fungi>chytridiomycota	Neocallimastix	californiae	putative calmodulin [Neocallimastix californiae]
ORY52785.1	151	LY90DRAFT_508143	eukaryota>fungi>chytridiomycota	Neocallimastix	californiae	calmodulin mutant SYNCAM35 [Neocallimastix californiae]
OWA54057.1	151	BV898_18479	eukaryota>metazoa	Hypsibius	dujardini	Calmodulin [Hypsibius dujardini]
NP_001236170.1	150	LOC100500308	eukaryota>viridiplantae	Glycine	max	uncharacterized protein LOC100500308 [Glycine max]
NP_001237902.1	150	cam-4	eukaryota>viridiplantae	Glycine	max	calmodulin [Glycine max]
NP_593340.1	150	cam1	eukaryota>fungi>ascomycota	Schizosaccharomyces	pombe	calmodulin Cam1 [Schizosaccharomyces pombe]
XP_002175972.1	150	SJAG_04901	eukaryota>fungi>ascomycota	Schizosaccharomyces	japonicus yFS275	calmodulin Cam1 [Schizosaccharomyces japonicus yFS275]
XP_003260925.1	150	CALM1	eukaryota>metazoa>chordata>vertebrata	Nomascus	leucogenys	PREDICTED: calmodulin isoform X2 [Nomascus leucogenys]
XP_005779994.1	150	EMIHUDRAFT_443126	eukaryota>haptophyceae	Emiliania	huxleyi CCMP1516	hypothetical protein EMIHUDRAFT_443126 [Emiliania huxleyi CCMP1516]
XP_005782374.1	150	CAM1	eukaryota>haptophyceae	Emiliania	huxleyi CCMP1516	calmodulin [Emiliania huxleyi CCMP1516]
XP_005787466.1	150	EMIHUDRAFT_373343	eukaryota>haptophyceae	Emiliania	huxleyi CCMP1516	hypothetical protein EMIHUDRAFT_373343 [Emiliania huxleyi CCMP1516]
XP_005792425.1	150	CAM2	eukaryota>haptophyceae	Emiliania	huxleyi CCMP1516	calmodulin [Emiliania huxleyi CCMP1516]
XP_006720321.1	150	CALML2,caM,CAMI,CPVT4,DD132,LQT14,PHKD	eukaryota>metazoa>chordata>vertebrata	Homo	sapiens	PREDICTED: calmodulin isoform X1 [Homo sapiens]
XP_009031530.1	150	HELRODRAFT_185219	eukaryota>metazoa>annelida	Helobdella	robusta	hypothetical protein HELRODRAFT_185219 [Helobdella robusta]
XP_013381514.1	150	LOC106152471	eukaryota>metazoa	Lingula	anatina	PREDICTED: calmodulin-alpha isoform X1 [Lingula anatina]
XP_019925479.1	150	LOC105334743	eukaryota>metazoa>mollusca	Crassostrea	gigas	PREDICTED: calmodulin-A-like [Crassostrea gigas]
Bcir1000013903	150	Bcir1000013903	eukaryota>fungi>mucoromycotina	Backusella	circina	>Bcir1000013903
Bcir1000016156	150	Bcir1000016156	eukaryota>fungi>mucoromycotina	Backusella	circina	>Bcir1000016156
Caps1000000131	150	Caps1000000131	eukaryota>metazoa>annelida	Capitella	spI	>Caps1000000131
Caps1000019447	150	Caps1000019447	eukaryota>metazoa>annelida	Capitella	spI	>Caps1000019447
Chet1000000433	150	Chet1000000433	eukaryota>fungi>ascomycota	Cochliobolus	heterostrophus	>Chet1000000433
Lhya1000002953	150	Lhya1000002953	eukaryota>fungi>mucoromycotina	Lichtheimia	hyalospora	>Lhya1000002953
Pisp1000011655	150	Pisp1000011655	eukaryota>fungi>neocallimastigomycota	Piromyces	sp	>Pisp1000011655
Smar1000011353	150	Smar1000011353	eukaryota>metazoa>arthropoda>myriapoda	Strigamia	maritima	>Smar1000011353
Smed1000011894	150	Smed1000011894	eukaryota>metazoa>platyhelminthe	Schmidtea	mediterranea	>Smed1000011894
XP_013393908.1	150	LOC106161494	eukaryota>metazoa	Lingula	anatina	PREDICTED: calmodulin-like isoform X2 [Lingula anatina]
XP_013393909.1	150	LOC106161494	eukaryota>metazoa	Lingula	anatina	PREDICTED: calmodulin-like isoform X2 [Lingula anatina]
XP_013393910.1	150	LOC106161494	eukaryota>metazoa	Lingula	anatina	PREDICTED: calmodulin-like isoform X2 [Lingula anatina]
XP_013393911.1	150	LOC106161494	eukaryota>metazoa	Lingula	anatina	PREDICTED: calmodulin-like isoform X2 [Lingula anatina]
XP_013393912.1	150	LOC106161494	eukaryota>metazoa	Lingula	anatina	PREDICTED: calmodulin-like isoform X2 [Lingula anatina]
XP_013393913.1	150	LOC106161494	eukaryota>metazoa	Lingula	anatina	PREDICTED: calmodulin-like isoform X2 [Lingula anatina]
XP_013393915.1	150	LOC106161494	eukaryota>metazoa	Lingula	anatina	PREDICTED: calmodulin-like isoform X2 [Lingula anatina]
XP_013393916.1	150	LOC106161494	eukaryota>metazoa	Lingula	anatina	PREDICTED: calmodulin-like isoform X2 [Lingula anatina]
XP_013405063.1	150	LOC106169937	eukaryota>metazoa	Lingula	anatina	PREDICTED: calmodulin-like isoform X2 [Lingula anatina]
NP_001236910.1	150	LOC100526987	eukaryota>viridiplantae	Glycine	max	uncharacterized protein LOC100526987 [Glycine max]
NP_001238254.1	150	SCAM-5	eukaryota>viridiplantae	Glycine	max	calmodulin [Glycine max]
XP_006350654.1	150	LOC102584290	eukaryota>viridiplantae	Solanum	tuberosum	PREDICTED: calmodulin-like protein 8 [Solanum tuberosum]
XP_016486568.1	150	LOC107806831	eukaryota>viridiplantae	Nicotiana	tabacum	PREDICTED: calmodulin-like protein 8 [Nicotiana tabacum]
XP_016500978.1	150	LOC107819387	eukaryota>viridiplantae	Nicotiana	tabacum	PREDICTED: calmodulin-like protein 8 [Nicotiana tabacum]
XP_003537938.1	150	LOC100781506	eukaryota>viridiplantae	Glycine	max	PREDICTED: calmodulin-like protein 3 [Glycine max]
XP_006345533.1	150	LOC102595420	eukaryota>viridiplantae	Solanum	tuberosum	PREDICTED: calmodulin-like protein 3 [Solanum tuberosum]
XP_006353827.1	150	LOC102585172	eukaryota>viridiplantae	Solanum	tuberosum	PREDICTED: calmodulin-like protein 3 [Solanum tuberosum]
XP_006592147.1	150	LOC100796922	eukaryota>viridiplantae	Glycine	max	PREDICTED: calmodulin-like protein 3 [Glycine max]
XP_016455923.1	150	LOC107779930	eukaryota>viridiplantae	Nicotiana	tabacum	PREDICTED: calmodulin-like protein 3 [Nicotiana tabacum]
XP_016458844.1	150	LOC107782471	eukaryota>viridiplantae	Nicotiana	tabacum	PREDICTED: calmodulin-like protein 3 [Nicotiana tabacum]
XP_009048149.1	150	LOTGIDRAFT_148903	eukaryota>metazoa>mollusca	Lottia	gigantea	hypothetical protein LOTGIDRAFT_148903 [Lottia gigantea]
XP_009056059.1	150	LOTGIDRAFT_216192	eukaryota>metazoa>mollusca	Lottia	gigantea	hypothetical protein LOTGIDRAFT_216192 [Lottia gigantea]
XP_009056060.1	150	LOTGIDRAFT_216194	eukaryota>metazoa>mollusca	Lottia	gigantea	hypothetical protein LOTGIDRAFT_216194 [Lottia gigantea]
XP_009056061.1	150	LOTGIDRAFT_216207	eukaryota>metazoa>mollusca	Lottia	gigantea	hypothetical protein LOTGIDRAFT_216207 [Lottia gigantea]
XP_009056062.1	150	LOTGIDRAFT_190032	eukaryota>metazoa>mollusca	Lottia	gigantea	hypothetical protein LOTGIDRAFT_190032 [Lottia gigantea]
XP_002274476.1	150	LOC100253496	eukaryota>viridiplantae	Vitis	vinifera	PREDICTED: probable calcium-binding protein CML23 [Vitis vinifera]
EPB82214.1	150	HMPREF1544_11058	eukaryota>fungi	Mucor	circinelloides f. circinelloides 1006PhL	calmodulin [Mucor circinelloides f. circinelloides 1006PhL]
XP_011416023.1	150	LOC105319977	eukaryota>metazoa>mollusca	Crassostrea	gigas	PREDICTED: calmodulin-2/4-like [Crassostrea gigas]
XP_019919518.1	150	LOC105319977	eukaryota>metazoa>mollusca	Crassostrea	gigas	PREDICTED: calmodulin-2/4-like [Crassostrea gigas]
XP_019919519.1	150	LOC105319977	eukaryota>metazoa>mollusca	Crassostrea	gigas	PREDICTED: calmodulin-2/4-like [Crassostrea gigas]
XP_011429058.1	150	LOC105329481	eukaryota>metazoa>mollusca	Crassostrea	gigas	PREDICTED: troponin C isoform X1 [Crassostrea gigas]
XP_011429059.1	150	LOC105329481	eukaryota>metazoa>mollusca	Crassostrea	gigas	PREDICTED: troponin C isoform X2 [Crassostrea gigas]
XP_014768462.1	150	LOC106867907	eukaryota>metazoa>mollusca	Octopus	bimaculoides	PREDICTED: troponin C-like [Octopus bimaculoides]
XP_014628533.1	150	LOC100778454	eukaryota>viridiplantae	Glycine	max	PREDICTED: calmodulin-like [Glycine max]
CEO94473.1	150	PBRA_000258	eukaryota>rhizaria>cercozoa>plasmodiophorida	Plasmodiophora	brassicae	hypothetical protein PBRA_000258 [Plasmodiophora brassicae]
EJY70654.1	150	OXYTRI_08484	eukaryota>alveolata>ciliophora	Oxytricha	trifallax	Calmodulin-related protein (macronuclear) [Oxytricha trifallax]
EPB84209.1	150	HMPREF1544_09001	eukaryota>fungi	Mucor	circinelloides f. circinelloides 1006PhL	calmodulin [Mucor circinelloides f. circinelloides 1006PhL]
NP_172089.1	150	RHS1,ROOT,HAIR,SPECIFIC,1,ROOT,HAIR,SPECIFIC,2,T21E18.4,T21E18_4	eukaryota>viridiplantae	Arabidopsis	thaliana	EF hand calcium-binding protein family [Arabidopsis thaliana]
OMJ78689.1	150	SteCoe_21455	eukaryota>alveolata>ciliophora	Stentor	coeruleus	hypothetical protein SteCoe_21455 [Stentor coeruleus]
XP_002501086.1	150	MICPUN_97186	eukaryota>viridiplantae>chlorophyta	Micromonas	commoda	predicted protein [Micromonas commoda]
XP_002586767.1	150	BRAFLDRAFT_224371	eukaryota>metazoa>chordata	Branchiostoma	floridae	hypothetical protein BRAFLDRAFT_224371, partial [Branchiostoma floridae]
XP_002593379.1	150	BRAFLDRAFT_57687	eukaryota>metazoa>chordata	Branchiostoma	floridae	hypothetical protein BRAFLDRAFT_57687 [Branchiostoma floridae]
XP_002678297.1	150	NAEGRDRAFT_36339	eukaryota>heterolobosea	Naegleria	gruberi strain NEG-M	predicted protein [Naegleria gruberi strain NEG-M]
XP_002738870.1	150	LOC100378235	eukaryota>metazoa>hemichordata	Saccoglossus	kowalevskii	PREDICTED: calmodulin-like [Saccoglossus kowalevskii]
XP_005717351.1	150	CHC_T00009119001	eukaryota>rhodophyta	Chondrus	crispus	Calmodulin [Chondrus crispus]
XP_006825918.1	150	LOC102806662	eukaryota>metazoa>hemichordata	Saccoglossus	kowalevskii	PREDICTED: calmodulin-like [Saccoglossus kowalevskii]
XP_009166212.1	150	T265_13247	eukaryota>metazoa	Opisthorchis	viverrini	hypothetical protein T265_13247, partial [Opisthorchis viverrini]
XP_011403658.1	150	LOC105312588	eukaryota>metazoa	Amphimedon	queenslandica	PREDICTED: squidulin-like [Amphimedon queenslandica]
XP_011443792.1	150	LOC105339773	eukaryota>metazoa>mollusca	Crassostrea	gigas	PREDICTED: neo-calmodulin-like [Crassostrea gigas]
XP_013421070.1	150	LOC106181286	eukaryota>metazoa	Lingula	anatina	PREDICTED: calmodulin-A-like [Lingula anatina]
XP_958218.3	150	NCU06948	eukaryota>fungi>ascomycota	Neurospora	crassa OR74A	efhand domain-containing protein [Neurospora crassa OR74A]
ORX90035.1	150	K493DRAFT_266301	eukaryota>fungi	Basidiobolus	meristosporus CBS 931.73	EF-hand [Basidiobolus meristosporus CBS 931.73]
ORX92738.1	150	K493DRAFT_285470	eukaryota>fungi	Basidiobolus	meristosporus CBS 931.73	calmodulin mutant SYNCAM64A [Basidiobolus meristosporus CBS 931.73]
XP_021884001.1	150	BCR41DRAFT_319669	eukaryota>fungi	Lobosporangium	transversale	hypothetical protein BCR41DRAFT_319669 [Lobosporangium transversale]
OAC98694.1	150	MUCCIDRAFT_181344	eukaryota>fungi	Mucor	circinelloides f. lusitanicus CBS 277.49	hypothetical protein MUCCIDRAFT_181344 [Mucor circinelloides f. lusitanicus CBS 277.49]
OWA50828.1	150	BV898_15333	eukaryota>metazoa	Hypsibius	dujardini	Calmodulin [Hypsibius dujardini]
ABO94197.1	149	OSTLU_39965	eukaryota>viridiplantae>chlorophyta	Ostreococcus	lucimarinus CCE9901	predicted protein [Ostreococcus lucimarinus CCE9901]
CBJ33511.1	149	Calmodulin	eukaryota>stramenopiles	Ectocarpus	siliculosus	Calcium-binding protein [Ectocarpus siliculosus]
CBY09922.1	149	GSOID_T00010740001	eukaryota>metazoa>chordata	Oikopleura	dioica	unnamed protein product [Oikopleura dioica]
CBY21565.1	149	GSOID_T00009337001	eukaryota>metazoa>chordata	Oikopleura	dioica	unnamed protein product [Oikopleura dioica]
CCW66691.1	149	GSHART1_T00001001001	eukaryota>euglenozoa>kinetoplastida	Phytomonas	sp. isolate Hart1	unnamed protein product [Phytomonas sp. isolate Hart1]
CDS37648.1	149	EmuJ_000491400	eukaryota>metazoa	Echinococcus	multilocularis	calmodulin [Echinococcus multilocularis]
CDW80161.1	149	Contig11946.g12780	eukaryota>alveolata>ciliophora	Stylonychia	lemnae	calmodulin [Stylonychia lemnae]
CEM02155.1	149	Vbra_1718	eukaryota>alveolata	Vitrella	brassicaformis CCMP3155	unnamed protein product [Vitrella brassicaformis CCMP3155]
CEM13742.1	149	Vbra_15560	eukaryota>alveolata	Vitrella	brassicaformis CCMP3155	unnamed protein product [Vitrella brassicaformis CCMP3155]
EFA81078.1	149	calA	eukaryota>amoebozoa>mycetozoa>dictyosteliida	Polysphondylium	pallidum PN500	calmodulin [Polysphondylium pallidum PN500]
EFX72807.1	149	CAM	eukaryota>metazoa>crustacea	Daphnia	pulex	calmodulin [Daphnia pulex]
EIE76027.1	149	RO3G_00731	eukaryota>fungi	Rhizopus	delemar RA 99-880	calmodulin [Rhizopus delemar RA 99-880]
EIE80772.1	149	RO3G_05477	eukaryota>fungi	Rhizopus	delemar RA 99-880	calmodulin [Rhizopus delemar RA 99-880]
EIE87194.1	149	RO3G_11905	eukaryota>fungi	Rhizopus	delemar RA 99-880	calmodulin [Rhizopus delemar RA 99-880]
EJK58168.1	149	THAOC_21730	eukaryota>stramenopiles	Thalassiosira	oceanica	hypothetical protein THAOC_21730 [Thalassiosira oceanica]
EJK77863.1	149	THAOC_00268	eukaryota>stramenopiles	Thalassiosira	oceanica	hypothetical protein THAOC_00268 [Thalassiosira oceanica]
EJY86595.1	149	OXYTRI_12398	eukaryota>alveolata>ciliophora	Oxytricha	trifallax	Calmodulin (macronuclear) [Oxytricha trifallax]
ELR14060.1	149	ACA1_366720	eukaryota>amoebozoa>acanthamoebidae	Acanthamoeba	castellanii str. Neff	Calmodulin, putative [Acanthamoeba castellanii str. Neff]
EPT04782.1	149	FOMPIDRAFT_1021634	eukaryota>fungi>basidiomycota	Fomitopsis	pinicola FP-58527 SS1	hypothetical protein FOMPIDRAFT_1021634 [Fomitopsis pinicola FP-58527 SS1]
EPY19237.1	149	STCU_09560	eukaryota>euglenozoa>kinetoplastida	Strigomonas	culicis	calmodulin [Strigomonas culicis]
EPY31669.1	149	AGDE_08936	eukaryota>euglenozoa>kinetoplastida	Angomonas	deanei	calmodulin [Angomonas deanei]
EPY34383.1	149	STCU_01612	eukaryota>euglenozoa>kinetoplastida	Strigomonas	culicis	calmodulin [Strigomonas culicis]
EPY35437.1	149	AGDE_07347	eukaryota>euglenozoa>kinetoplastida	Angomonas	deanei	calmodulin [Angomonas deanei]
EPY41887.1	149	AGDE_02036	eukaryota>euglenozoa>kinetoplastida	Angomonas	deanei	calmodulin [Angomonas deanei]
GAQ91141.1	149	KFL_007340010	eukaryota>viridiplantae	Klebsormidium	nitens	calmodulin [Klebsormidium nitens]
KFH70124.1	149	MVEG_04927	eukaryota>fungi	Mortierella	verticillata NRRL 6337	calmodulin [Mortierella verticillata NRRL 6337]
KPI82396.1	149	ABL78_8594	eukaryota>euglenozoa>kinetoplastida	Leptomonas	seymouri	calmodulin-like protein [Leptomonas seymouri]
KXN66162.1	149	CONCODRAFT_131644	eukaryota>fungi	Conidiobolus	coronatus NRRL 28638	calmodulin [Conidiobolus coronatus NRRL 28638]
NP_001027633.1	149	Ci-CaM	eukaryota>metazoa>chordata	Ciona	intestinalis	calmodulin [Ciona intestinalis]
NP_001040234.1	149	LOC692927	eukaryota>metazoa>hexapoda	Bombyx	mori	calmodulin [Bombyx mori]
NP_001075166.1	149	CALM3,CCM1	eukaryota>metazoa>chordata>vertebrata	Gallus	gallus	calmodulin, striated muscle [Gallus gallus]
NP_001080864.1	149	calm1,calm2,calm2-a,calm2-b,camii,phkd,phkd2	eukaryota>metazoa>chordata>vertebrata	Xenopus	laevis	calmodulin-2 A [Xenopus laevis]
NP_001084025.1	149	calm1,calm2-a,calm2-b,cam	eukaryota>metazoa>chordata>vertebrata	Xenopus	laevis	calmodulin-2 B [Xenopus laevis]
NP_001089059.1	149	calm1,calm2-a,calm2-b,calm2a,calml2,cami,dd132	eukaryota>metazoa>chordata>vertebrata	Xenopus	laevis	calmodulin-1 [Xenopus laevis]
NP_001103834.1	149	CALM1	eukaryota>metazoa>chordata>vertebrata	Gallus	gallus	neo-calmodulin [Gallus gallus]
NP_001104884.1	149	GRMZM2G004703,Zmrcalm	eukaryota>viridiplantae	Zea	mays	calmodulin [Zea mays]
NP_001105455.1	149	ZMCALM1	eukaryota>viridiplantae	Zea	mays	calmodulin [Zea mays]
NP_001105459.1	149	CaM1	eukaryota>viridiplantae	Zea	mays	calmodulin [Zea mays]
NP_001105547.1	149	CaM2,GRMZM2G067511	eukaryota>viridiplantae	Zea	mays	calmodulin 2 [Zea mays]
NP_001119640.2	149	Cam	eukaryota>metazoa>hexapoda	Acyrthosiphon	pisum	calmodulin [Acyrthosiphon pisum]
NP_001136954.1	149	GRMZM2G395244	eukaryota>viridiplantae	Zea	mays	uncharacterized protein LOC100217114 [Zea mays]
NP_001167666.1	149	GRMZM2G430600	eukaryota>viridiplantae	Zea	mays	calmodulin [Zea mays]
NP_001171813.1	149	LOC100372664	eukaryota>metazoa>hemichordata	Saccoglossus	kowalevskii	calmodulin [Saccoglossus kowalevskii]
NP_001236109.1	149	LOC100527439	eukaryota>viridiplantae	Glycine	max	uncharacterized protein LOC100527439 [Glycine max]
NP_001236711.1	149	SCAM-3	eukaryota>viridiplantae	Glycine	max	calmodulin [Glycine max]
NP_001237883.1	149	CaM-2	eukaryota>viridiplantae	Glycine	max	calmodulin-2 [Glycine max]
NP_001238237.1	149	SCAM-1	eukaryota>viridiplantae	Glycine	max	calmodulin [Glycine max]
NP_001242690.1	149	cam-1	eukaryota>viridiplantae	Glycine	max	calmodulin-like [Glycine max]
NP_001246276.1	149	3909,anon-EST:Posey59,Cal,Cal49A,CalA,cam,CaM,CAM,Cam1,CG8472,D-CaM,dCaM,DmCalm,DmCaM,Dmel\CG8472,l(2)03909,l(2)3909,lincRNA.S2634	eukaryota>metazoa>hexapoda	Drosophila	melanogaster	calmodulin, isoform C [Drosophila melanogaster]
NP_001246277.1	149	3909,anon-EST:Posey59,Cal,Cal49A,CalA,cam,CaM,CAM,Cam1,CG8472,D-CaM,dCaM,DmCalm,DmCaM,Dmel\CG8472,l(2)03909,l(2)3909,lincRNA.S2634	eukaryota>metazoa>hexapoda	Drosophila	melanogaster	calmodulin, isoform D [Drosophila melanogaster]
NP_001271050.1	149	CALM3	eukaryota>metazoa>chordata>vertebrata	Macaca	fascicularis	calmodulin [Macaca fascicularis]
NP_001271970.1	149	CALM2	eukaryota>metazoa>chordata>vertebrata	Macaca	fascicularis	calmodulin [Macaca fascicularis]
NP_001275042.1	149	CaM-1,CaM1,PCM1,StCaM4	eukaryota>viridiplantae	Solanum	tuberosum	calmodulin-1 [Solanum tuberosum]
NP_001275359.1	149	CALMODULIN,PCM5,PCM6,PCM7,StCaM1	eukaryota>viridiplantae	Solanum	tuberosum	calmodulin-5/6/7/8 [Solanum tuberosum]
NP_001286337.1	149	3909,anon-EST:Posey59,Cal,Cal49A,CalA,cam,CaM,CAM,Cam1,CG8472,D-CaM,dCaM,DmCalm,DmCaM,Dmel\CG8472,l(2)03909,l(2)3909,lincRNA.S2634	eukaryota>metazoa>hexapoda	Drosophila	melanogaster	calmodulin, isoform E [Drosophila melanogaster]
NP_001734.1	149	caM,CAMII,LQT15,PHKD,PHKD2	eukaryota>metazoa>chordata>vertebrata	Homo	sapiens	calmodulin-2 isoform 2 [Homo sapiens]
NP_005175.2	149	CaM,CaMIII,HEL-S-72,PHKD,PHKD3	eukaryota>metazoa>chordata>vertebrata	Homo	sapiens	calmodulin-3 isoform 1 [Homo sapiens]
NP_005176.1	149	CLP	eukaryota>metazoa>chordata>vertebrata	Homo	sapiens	calmodulin-like protein 3 [Homo sapiens]
NP_008819.1	149	CALML2,caM,CAMI,CPVT4,DD132,LQT14,PHKD	eukaryota>metazoa>chordata>vertebrata	Homo	sapiens	calmodulin-1 [Homo sapiens]
NP_031615.1	149	1500001E21Rik,AL024017,Cam2,CamC	eukaryota>metazoa>chordata>vertebrata	Mus	musculus	calmodulin-2 isoform 1 [Mus musculus]
NP_031616.1	149	Cam3,CaMA,Camc,R75142	eukaryota>metazoa>chordata>vertebrata	Mus	musculus	calmodulin-3 [Mus musculus]
NP_033920.1	149	AI256814,AI327027,AI461935,AL024000,Calm,CaM,Cam1	eukaryota>metazoa>chordata>vertebrata	Mus	musculus	calmodulin-1 isoform 2 [Mus musculus]
NP_081692.1	149	2310068O22Rik,AI326174	eukaryota>metazoa>chordata>vertebrata	Mus	musculus	calmodulin-like protein 3 [Mus musculus]
NP_176814.1	149	ACAM-4,calmodulin,4,CALMODULIN,4,T27F4.1,T27F4_1	eukaryota>viridiplantae	Arabidopsis	thaliana	calmodulin 4 [Arabidopsis thaliana]
NP_180271.1	149	1357,ACAM-2,calmodulin,2,calmodulin,5,CAM2,T20P8.8	eukaryota>viridiplantae	Arabidopsis	thaliana	calmodulin 5 [Arabidopsis thaliana]
NP_189967.1	149	AtCAM7,calmodulin,7,CALMODULIN,7,Z-box,Binding,Factor,3,ZBF3	eukaryota>viridiplantae	Arabidopsis	thaliana	calmodulin 7 [Arabidopsis thaliana]
NP_191239.1	149	acam-3,ACAM-3,calmodulin,3,CALMODULIN,3,T8M16.130	eukaryota>viridiplantae	Arabidopsis	thaliana	calmodulin 3 [Arabidopsis thaliana]
NP_198594.1	149	ACAM-1,calmodulin,1,K22F20.20,K22F20_20,TCH1,TOUCH,1	eukaryota>viridiplantae	Arabidopsis	thaliana	calmodulin 1 [Arabidopsis thaliana]
NP_503386.1	149	cmd-1	eukaryota>metazoa>nematoda	Caenorhabditis	elegans	Calmodulin [Caenorhabditis elegans]
NP_523710.1	149	3909,anon-EST:Posey59,Cal,Cal49A,CalA,cam,CaM,CAM,Cam1,CG8472,D-CaM,dCaM,DmCalm,DmCaM,Dmel\CG8472,l(2)03909,l(2)3909,lincRNA.S2634	eukaryota>metazoa>hexapoda	Drosophila	melanogaster	calmodulin, isoform A [Drosophila melanogaster]
NP_725120.1	149	3909,anon-EST:Posey59,Cal,Cal49A,CalA,cam,CaM,CAM,Cam1,CG8472,D-CaM,dCaM,DmCalm,DmCaM,Dmel\CG8472,l(2)03909,l(2)3909,lincRNA.S2634	eukaryota>metazoa>hexapoda	Drosophila	melanogaster	calmodulin, isoform B [Drosophila melanogaster]
NP_850344.1	149	ATCAL5,calmodulin,2,CALMODULIN,2,T3K9.12,T3K9_12	eukaryota>viridiplantae	Arabidopsis	thaliana	calmodulin 2 [Arabidopsis thaliana]
NP_850860.1	149	ACAM-6,calmodulin,6	eukaryota>viridiplantae	Arabidopsis	thaliana	calmodulin 6 [Arabidopsis thaliana]
NP_892012.1	149	calm2g,CaMgamma,wu:fi05b08,zgc:86728	eukaryota>metazoa>chordata>vertebrata>actinopterygii	Danio	rerio	calmodulin-3 [Danio rerio]
NP_955864.1	149	wu:fb36a09,zgc:55591,zgc:76987	eukaryota>metazoa>chordata>vertebrata>actinopterygii	Danio	rerio	calmodulin [Danio rerio]
NP_956290.1	149	calm2d,CaMalpha,cb815,wu:fj49d04,zgc:73045	eukaryota>metazoa>chordata>vertebrata>actinopterygii	Danio	rerio	calmodulin 2a [Danio rerio]
NP_956376.1	149	CaMbeta-2,zgc:73137	eukaryota>metazoa>chordata>vertebrata>actinopterygii	Danio	rerio	calmodulin 1b [Danio rerio]
NP_990336.1	149	CALM	eukaryota>metazoa>chordata>vertebrata	Gallus	gallus	calmodulin [Gallus gallus]
NP_998516.1	149	CaMbeta,sb:cb617,wu:fb08d09,wu:fb69c08,wu:fj34a08,zgc:63926	eukaryota>metazoa>chordata>vertebrata>actinopterygii	Danio	rerio	calmodulin 1a [Danio rerio]
NP_999901.1	149	calm2,cb169,zgc:64036	eukaryota>metazoa>chordata>vertebrata>actinopterygii	Danio	rerio	calmodulin-2 [Danio rerio]
OAF71028.1	149	A3Q56_01202	eukaryota>metazoa	Intoshia	linei	CaM [Intoshia linei]
OEU16674.1	149	FRACYDRAFT_269076	eukaryota>stramenopiles	Fragilariopsis	cylindrus CCMP1102	calmodulin [Fragilariopsis cylindrus CCMP1102]
OLP83472.1	149	Calmodulin	eukaryota>alveolata	Symbiodinium	microadriaticum	Calmodulin [Symbiodinium microadriaticum]
OLP83473.1	149	Calmodulin	eukaryota>alveolata	Symbiodinium	microadriaticum	Calmodulin [Symbiodinium microadriaticum]
OMJ80243.1	149	SteCoe_19560	eukaryota>alveolata>ciliophora	Stentor	coeruleus	hypothetical protein SteCoe_19560 [Stentor coeruleus]
OMJ88554.1	149	SteCoe_9508	eukaryota>alveolata>ciliophora	Stentor	coeruleus	hypothetical protein SteCoe_9508 [Stentor coeruleus]
XP_001022775.2	149	TTHERM_00630500	eukaryota>alveolata>ciliophora	Tetrahymena	thermophila SB210	calmodulin 1 [Tetrahymena thermophila SB210]
XP_001224933.1	149	CHGG_07277	eukaryota>fungi>ascomycota	Chaetomium	globosum CBS 148.51	calmodulin [Chaetomium globosum CBS 148.51]
XP_001348497.1	149	PF14_0323	eukaryota>alveolata>apicomplexa	Plasmodium	falciparum 3D7	calmodulin [Plasmodium falciparum 3D7]
XP_001388192.1	149	cgd2_810	eukaryota>alveolata>apicomplexa	Cryptosporidium	parvum Iowa II	calmodulin [Cryptosporidium parvum Iowa II]
XP_001448363.1	149	GSPATT00015825001	eukaryota>alveolata>ciliophora	Paramecium	tetraurelia strain d4-2	hypothetical protein (macronuclear) [Paramecium tetraurelia strain d4-2]
XP_001463554.1	149	LINJ_09_0970	eukaryota>euglenozoa>kinetoplastida	Leishmania	infantum JPCM5	putative calmodulin [Leishmania infantum JPCM5]
XP_001463555.1	149	LINJ_09_0980	eukaryota>euglenozoa>kinetoplastida	Leishmania	infantum JPCM5	putative calmodulin [Leishmania infantum JPCM5]
XP_001544278.1	149	HCAG_01325	eukaryota>fungi>ascomycota	Histoplasma	capsulatum NAm1	calmodulin [Histoplasma capsulatum NAm1]
XP_001562710.1	149	LBRM_09_0960	eukaryota>euglenozoa>kinetoplastida	Leishmania	braziliensis MHOM/BR/75/M2904	putative calmodulin [Leishmania braziliensis MHOM/BR/75/M2904]
XP_001562711.1	149	LBRM_09_0970	eukaryota>euglenozoa>kinetoplastida	Leishmania	braziliensis MHOM/BR/75/M2904	putative calmodulin [Leishmania braziliensis MHOM/BR/75/M2904]
XP_001562712.1	149	LBRM_09_0980	eukaryota>euglenozoa>kinetoplastida	Leishmania	braziliensis MHOM/BR/75/M2904	putative calmodulin [Leishmania braziliensis MHOM/BR/75/M2904]
XP_001609057.1	149	19.m02335	eukaryota>alveolata>apicomplexa	Babesia	bovis T2Bo	calmodulin [Babesia bovis T2Bo]
XP_001616632.1	149	PVX_084825	eukaryota>alveolata>apicomplexa	Plasmodium	vivax Sal-1	calmodulin [Plasmodium vivax Sal-1]
XP_001638581.1	149	NEMVEDRAFT_v1g239788	eukaryota>metazoa>cnidaria	Nematostella	vectensis	predicted protein [Nematostella vectensis]
XP_001681265.1	149	LMJF_09_0910	eukaryota>euglenozoa>kinetoplastida	Leishmania	major strain Friedlin	putative calmodulin [Leishmania major strain Friedlin]
XP_001681266.1	149	LMJF_09_0920	eukaryota>euglenozoa>kinetoplastida	Leishmania	major strain Friedlin	putative calmodulin [Leishmania major strain Friedlin]
XP_001681267.1	149	LMJF_09_0930	eukaryota>euglenozoa>kinetoplastida	Leishmania	major strain Friedlin	putative calmodulin [Leishmania major strain Friedlin]
XP_001749021.1	149	MONBRDRAFT_33928	eukaryota>choanoflagellida	Monosiga	brevicollis MX1	hypothetical protein [Monosiga brevicollis MX1]
XP_001749022.1	149	MONBRDRAFT_11217	eukaryota>choanoflagellida	Monosiga	brevicollis MX1	hypothetical protein [Monosiga brevicollis MX1]
XP_001749024.1	149	MONBRDRAFT_11222	eukaryota>choanoflagellida	Monosiga	brevicollis MX1	hypothetical protein [Monosiga brevicollis MX1]
XP_001755625.1	149	PHYPADRAFT_116985	eukaryota>viridiplantae	Physcomitrella	patens	predicted protein [Physcomitrella patens]
XP_001758487.1	149	PHYPADRAFT_161424	eukaryota>viridiplantae	Physcomitrella	patens	predicted protein [Physcomitrella patens]
XP_001760060.1	149	PHYPADRAFT_180168	eukaryota>viridiplantae	Physcomitrella	patens	predicted protein [Physcomitrella patens]
XP_001763057.1	149	PHYPADRAFT_210000	eukaryota>viridiplantae	Physcomitrella	patens	predicted protein [Physcomitrella patens]
XP_001765847.1	149	PHYPADRAFT_185037	eukaryota>viridiplantae	Physcomitrella	patens	predicted protein [Physcomitrella patens]
XP_001834963.2	149	CC1G_11477	eukaryota>fungi>basidiomycota	Coprinopsis	cinerea okayama7#130	calmodulin [Coprinopsis cinerea okayama7#130]
XP_001868623.1	149	CpipJ_CPIJ018366	eukaryota>metazoa>hexapoda	Culex	quinquefasciatus	calmodulin [Culex quinquefasciatus]
XP_001879332.1	149	LACBIDRAFT_318312	eukaryota>fungi>basidiomycota	Laccaria	bicolor S238N-H82	predicted protein [Laccaria bicolor S238N-H82]
XP_002109063.1	149	TRIADDRAFT_37105	eukaryota>metazoa>placozoa	Trichoplax	adhaerens	calmodulin [Trichoplax adhaerens]
XP_002140815.1	149	CMU_009580	eukaryota>alveolata>apicomplexa	Cryptosporidium	muris RN66	calmodulin [Cryptosporidium muris RN66]
XP_002154349.2	149	LOC100200547	eukaryota>metazoa>cnidaria	Hydra	vulgaris	PREDICTED: calmodulin-A [Hydra vulgaris]
XP_002185049.1	149	CaM1	eukaryota>stramenopiles	Phaeodactylum	tricornutum CCAP 1055/1	calmoduline [Phaeodactylum tricornutum CCAP 1055/1]
XP_002295755.1	149	CAM1	eukaryota>stramenopiles	Thalassiosira	pseudonana CCMP1335	calmodulin [Thalassiosira pseudonana CCMP1335]
XP_002367258.1	149	TGME49_249240	eukaryota>alveolata>apicomplexa	Toxoplasma	gondii ME49	calmodulin, putative [Toxoplasma gondii ME49]
XP_002473838.1	149	CAM1	eukaryota>fungi>basidiomycota	Postia	placenta Mad-698-R	calmodulin [Postia placenta Mad-698-R]
XP_002507130.1	149	CAM	eukaryota>viridiplantae>chlorophyta	Micromonas	commoda	calmodulin [Micromonas commoda]
XP_002593254.1	149	BRAFLDRAFT_87235	eukaryota>metazoa>chordata	Branchiostoma	floridae	hypothetical protein BRAFLDRAFT_87235 [Branchiostoma floridae]
XP_002593255.1	149	BRAFLDRAFT_124869	eukaryota>metazoa>chordata	Branchiostoma	floridae	hypothetical protein BRAFLDRAFT_124869 [Branchiostoma floridae]
XP_002593257.1	149	BRAFLDRAFT_59748	eukaryota>metazoa>chordata	Branchiostoma	floridae	hypothetical protein BRAFLDRAFT_59748 [Branchiostoma floridae]
XP_002598032.1	149	BRAFLDRAFT_58814	eukaryota>metazoa>chordata	Branchiostoma	floridae	hypothetical protein BRAFLDRAFT_58814 [Branchiostoma floridae]
XP_002734090.1	149	LOC100371257	eukaryota>metazoa>hemichordata	Saccoglossus	kowalevskii	PREDICTED: calmodulin-like [Saccoglossus kowalevskii]
XP_002739903.1	149	LOC100376745	eukaryota>metazoa>hemichordata	Saccoglossus	kowalevskii	PREDICTED: calmodulin-like [Saccoglossus kowalevskii]
XP_002767178.1	149	Pmar_PMAR006570	eukaryota>alveolata	Perkinsus	marinus ATCC 50983	calmodulin, putative [Perkinsus marinus ATCC 50983]

XP_002779137.1	149	Pmar_PMAR021412	eukaryota>alveolata	Perkinsus	marinus ATCC 50983	calmodulin, putative [Perkinsus marinus ATCC 50983]
XP_002785606.1	149	Pmar_PMAR022352	eukaryota>alveolata	Perkinsus	marinus ATCC 50983	calmodulin, putative [Perkinsus marinus ATCC 50983]
XP_002963446.1	149	SELMODRAFT_141966	eukaryota>viridiplantae	Selaginella	moellendorffii	hypothetical protein SELMODRAFT_141966 [Selaginella moellendorffii]
XP_002971581.1	149	SELMODRAFT_172372	eukaryota>viridiplantae	Selaginella	moellendorffii	hypothetical protein SELMODRAFT_172372 [Selaginella moellendorffii]
XP_002977832.1	149	SELMODRAFT_107557	eukaryota>viridiplantae	Selaginella	moellendorffii	hypothetical protein SELMODRAFT_107557 [Selaginella moellendorffii]
XP_002979493.1	149	SELMODRAFT_233374	eukaryota>viridiplantae	Selaginella	moellendorffii	hypothetical protein SELMODRAFT_233374 [Selaginella moellendorffii]
XP_002998616.1	149	PITG_06514	eukaryota>stramenopiles	Phytophthora	infestans T30-4	calmodulin [Phytophthora infestans T30-4]
XP_003056131.1	149	CAM	eukaryota>viridiplantae>chlorophyta	Micromonas	pusilla CCMP1545	calmodulin [Micromonas pusilla CCMP1545]
XP_003257649.1	149	CALML3	eukaryota>metazoa>chordata>vertebrata	Nomascus	leucogenys	PREDICTED: calmodulin-like protein 3 [Nomascus leucogenys]
XP_003260924.1	149	CALM1	eukaryota>metazoa>chordata>vertebrata	Nomascus	leucogenys	PREDICTED: calmodulin isoform X3 [Nomascus leucogenys]
XP_003262870.1	149	CALM2	eukaryota>metazoa>chordata>vertebrata	Nomascus	leucogenys	PREDICTED: calmodulin isoform X3 [Nomascus leucogenys]
XP_003277701.2	149	CALM3	eukaryota>metazoa>chordata>vertebrata	Nomascus	leucogenys	PREDICTED: calmodulin isoform X1 [Nomascus leucogenys]
XP_003329587.1	149	PGTG_11337	eukaryota>fungi>basidiomycota	Puccinia	graminis f. sp. tritici CRL 75-36-700-3	calmodulin [Puccinia graminis f. sp. tritici CRL 75-36-700-3]
XP_003519492.1	149	LOC100793700	eukaryota>viridiplantae	Glycine	max	PREDICTED: calmodulin [Glycine max]
XP_003545473.1	149	LOC100791702	eukaryota>viridiplantae	Glycine	max	PREDICTED: calmodulin [Glycine max]
XP_003726932.1	149	LOC591003	eukaryota>metazoa>echinodermata	Strongylocentrotus	purpuratus	PREDICTED: calmodulin isoform X2 [Strongylocentrotus purpuratus]
XP_003858776.1	149	LDBPK_090970	eukaryota>euglenozoa>kinetoplastida	Leishmania	donovani	calmodulin, putative [Leishmania donovani]
XP_003858777.1	149	LDBPK_090980	eukaryota>euglenozoa>kinetoplastida	Leishmania	donovani	calmodulin, putative [Leishmania donovani]
XP_003872774.1	149	LMXM_09_0910	eukaryota>euglenozoa>kinetoplastida	Leishmania	mexicana MHOM/GT/2001/U1103	putative calmodulin [Leishmania mexicana MHOM/GT/2001/U1103]
XP_003872775.1	149	LMXM_09_0920	eukaryota>euglenozoa>kinetoplastida	Leishmania	mexicana MHOM/GT/2001/U1103	putative calmodulin [Leishmania mexicana MHOM/GT/2001/U1103]
XP_003872776.1	149	LMXM_09_0930	eukaryota>euglenozoa>kinetoplastida	Leishmania	mexicana MHOM/GT/2001/U1103	putative calmodulin [Leishmania mexicana MHOM/GT/2001/U1103]
XP_003886144.1	149	NCLIV_065440	eukaryota>alveolata>apicomplexa	Neospora	caninum Liverpool	hypothetical protein NCLIV_065440 [Neospora caninum Liverpool]
XP_003963666.1	149	calm2	eukaryota>metazoa>chordata>vertebrata>actinopterygii	Takifugu	rubripes	PREDICTED: calmodulin [Takifugu rubripes]
XP_003968409.1	149	calm3	eukaryota>metazoa>chordata>vertebrata>actinopterygii	Takifugu	rubripes	PREDICTED: calmodulin [Takifugu rubripes]
XP_003971505.1	149	LOC101068488	eukaryota>metazoa>chordata>vertebrata>actinopterygii	Takifugu	rubripes	PREDICTED: calmodulin [Takifugu rubripes]
XP_003976021.1	149	LOC101064505	eukaryota>metazoa>chordata>vertebrata>actinopterygii	Takifugu	rubripes	PREDICTED: calmodulin-alpha-like [Takifugu rubripes]
XP_004039912.1	149	IMG5_006070	eukaryota>alveolata>ciliophora	Ichthyophthirius	multifiliis	hypothetical protein IMG5_006070 [Ichthyophthirius multifiliis]
XP_004091507.1	149	CALM2	eukaryota>metazoa>chordata>vertebrata	Nomascus	leucogenys	PREDICTED: calmodulin isoform X3 [Nomascus leucogenys]
XP_004349444.1	149	CAOG_02694	eukaryota>ichthyosporea	Capsaspora	owczarzaki ATCC 30864	calmodulin [Capsaspora owczarzaki ATCC 30864]
XP_004829358.1	149	BEWA_025410	eukaryota>alveolata>apicomplexa	Theileria	equi	calmodulin, putative [Theileria equi]
XP_004993230.1	149	PTSG_06340	eukaryota>choanoflagellida	Salpingoeca	rosetta	calmodulin [Salpingoeca rosetta]
XP_005562071.1	149	CALM1	eukaryota>metazoa>chordata>vertebrata	Macaca	fascicularis	PREDICTED: calmodulin [Macaca fascicularis]
XP_005564612.1	149	CALML3	eukaryota>metazoa>chordata>vertebrata	Macaca	fascicularis	PREDICTED: calmodulin-like protein 3 [Macaca fascicularis]
XP_005836584.1	149	GUITHDRAFT_151413	eukaryota>cryptophyta	Guillardia	theta CCMP2712	hypothetical protein GUITHDRAFT_151413 [Guillardia theta CCMP2712]
XP_005839886.1	149	GUITHDRAFT_150466	eukaryota>cryptophyta	Guillardia	theta CCMP2712	hypothetical protein GUITHDRAFT_150466 [Guillardia theta CCMP2712]
XP_005843378.1	149	CHLNCDRAFT_59820	eukaryota>viridiplantae>chlorophyta	Chlorella	variabilis	hypothetical protein CHLNCDRAFT_59820 [Chlorella variabilis]
XP_006339234.1	149	LOC102579473	eukaryota>viridiplantae	Solanum	tuberosum	PREDICTED: calmodulin-7 [Solanum tuberosum]
XP_006364752.1	149	LOC102599353	eukaryota>viridiplantae	Solanum	tuberosum	PREDICTED: calmodulin [Solanum tuberosum]
XP_006565317.1	149	LOC551859	eukaryota>metazoa>hexapoda	Apis	mellifera	PREDICTED: calmodulin [Apis mellifera]
XP_006959848.1	149	WALSEDRAFT_70094	eukaryota>fungi>basidiomycota	Wallemia	mellicola CBS 633.66	EF-hand [Wallemia mellicola CBS 633.66]
XP_007331882.1	149	AGABI1DRAFT_86553	eukaryota>fungi>basidiomycota	Agaricus	bisporus var. burnettii JB137-S8	hypothetical protein AGABI1DRAFT_86553 [Agaricus bisporus var. burnettii JB137-S8]
XP_008607073.1	149	SDRG_03224	eukaryota>stramenopiles	Saprolegnia	diclina VS20	calmodulin [Saprolegnia diclina VS20]
XP_008655487.1	149	LOC103634668	eukaryota>viridiplantae	Zea	mays	calmodulin [Zea mays]
XP_008660204.1	149	LOC103639205	eukaryota>viridiplantae	Zea	mays	calmodulin [Zea mays]
XP_008883927.1	149	HHA_249240	eukaryota>alveolata>apicomplexa	Hammondia	hammondi	calmodulin, putative [Hammondia hammondi]
XP_008908788.1	149	PPTG_13130	eukaryota>stramenopiles	Phytophthora	parasitica INRA-310	calmodulin [Phytophthora parasitica INRA-310]
XP_009033803.1	149	AURANDRAFT_36419	eukaryota>stramenopiles	Aureococcus	anophagefferens	hypothetical protein AURANDRAFT_36419 [Aureococcus anophagefferens]
XP_009033883.1	149	AURANDRAFT_58710	eukaryota>stramenopiles	Aureococcus	anophagefferens	hypothetical protein AURANDRAFT_58710 [Aureococcus anophagefferens]
XP_009054739.1	149	LOTGIDRAFT_215397	eukaryota>metazoa>mollusca	Lottia	gigantea	hypothetical protein LOTGIDRAFT_215397 [Lottia gigantea]
XP_009056063.1	149	LOTGIDRAFT_203998	eukaryota>metazoa>mollusca	Lottia	gigantea	hypothetical protein LOTGIDRAFT_203998 [Lottia gigantea]
XP_009056065.1	149	LOTGIDRAFT_216228	eukaryota>metazoa>mollusca	Lottia	gigantea	hypothetical protein LOTGIDRAFT_216228 [Lottia gigantea]
XP_009315838.1	149	DQ04_17211000	eukaryota>euglenozoa>kinetoplastida	Trypanosoma	grayi	calmodulin [Trypanosoma grayi]
XP_009539924.1	149	PHYSODRAFT_292780	eukaryota>stramenopiles	Phytophthora	sojae	hypothetical protein PHYSODRAFT_292780 [Phytophthora sojae]
XP_009690713.1	149	TOT_020000669	eukaryota>alveolata>apicomplexa	Theileria	orientalis strain Shintoku	calmodulin [Theileria orientalis strain Shintoku]
XP_010645766.1	149	LOC100260795	eukaryota>viridiplantae	Vitis	vinifera	PREDICTED: calmodulin [Vitis vinifera]
XP_010704329.1	149	LPMP_090920	eukaryota>euglenozoa>kinetoplastida	Leishmania	panamensis	calmodulin, putative [Leishmania panamensis]
XP_010704330.1	149	LPMP_090930	eukaryota>euglenozoa>kinetoplastida	Leishmania	panamensis	calmodulin, putative [Leishmania panamensis]
XP_011129786.1	149	GNI_051490	eukaryota>alveolata>apicomplexa	Gregarina	niphandrodes	calmodulin [Gregarina niphandrodes]
XP_011139526.1	149	LOC105183241	eukaryota>metazoa>hexapoda	Harpegnathos	saltator	PREDICTED: calmodulin isoform X1 [Harpegnathos saltator]
XP_011139527.1	149	LOC105183241	eukaryota>metazoa>hexapoda	Harpegnathos	saltator	PREDICTED: calmodulin isoform X2 [Harpegnathos saltator]
XP_011390388.1	149	UMAG_03910	eukaryota>fungi>basidiomycota	Ustilago	maydis 521	putative calmodulin [Ustilago maydis 521]
XP_011416022.2	149	LOC105319976	eukaryota>metazoa>mollusca	Crassostrea	gigas	PREDICTED: neo-calmodulin-like [Crassostrea gigas]
XP_011427008.1	149	LOC105327998	eukaryota>metazoa>mollusca	Crassostrea	gigas	PREDICTED: calmodulin-A-like [Crassostrea gigas]
XP_011427009.1	149	LOC105327999	eukaryota>metazoa>mollusca	Crassostrea	gigas	PREDICTED: calmodulin-A-like [Crassostrea gigas]
[illegible]	[illegible]	[illegible]	[illegible]	[illegible]	[illegible]	[illegible]

XP_002285864.1	499	LOC100259138	eukaryota>viridiplantae	Vitis	vinifera	PREDICTED: protein disulfide-isomerase [Vitis vinifera]
XP_005837233.1	499	GUITHDRAFT_85427	eukaryota>cryptophyta	Guillardia	theta CCMP2712	hypothetical protein GUITHDRAFT_85427 [Guillardia theta CCMP2712]
XP_014785522.1	499	LOC106880182	eukaryota>metazoa>mollusca	Octopus	bimaculoides	PREDICTED: protein disulfide-isomerase A3-like [Octopus bimaculoides]
ORY00427.1	499	K493DRAFT_103679	eukaryota>fungi	Basidiobolus	meristosporus CBS 931.73	hypothetical protein K493DRAFT_103679 [Basidiobolus meristosporus CBS 931.73]
EIE76133.1	498	RO3G_00837	eukaryota>fungi	Rhizopus	delemar RA 99-880	hypothetical protein RO3G_00837 [Rhizopus delemar RA 99-880]
KXN72927.1	498	CONCODRAFT_15852	eukaryota>fungi	Conidiobolus	coronatus NRRL 28638	protein disulfide-isomerase [Conidiobolus coronatus NRRL 28638]
XP_001766340.1	498	PHYPADRAFT_130646	eukaryota>viridiplantae	Physcomitrella	patens	predicted protein [Physcomitrella patens]
XP_008190988.1	498	LOC103312345	eukaryota>metazoa>hexapoda	Tribolium	castaneum	PREDICTED: protein disulfide-isomerase [Tribolium castaneum]
XP_008877298.1	498	H310_12113	eukaryota>stramenopiles	Aphanomyces	invadans	protein disulfide-isomerase domain [Aphanomyces invadans]
XP_009833015.1	498	H257_08690	eukaryota>stramenopiles	Aphanomyces	astaci	protein disulfide-isomerase domain [Aphanomyces astaci]
Lhya1000002422	498	Lhya1000002422	eukaryota>fungi>mucoromycotina	Lichtheimia	hyalospora	>Lhya1000002422
XP_001897232.1	497	Bm1_28935	eukaryota>metazoa>nematoda	Brugia	malayi	transglutaminase [Brugia malayi]
XP_006363890.1	497	LOC102592089	eukaryota>viridiplantae	Solanum	tuberosum	PREDICTED: protein disulfide-isomerase-like [Solanum tuberosum]
XP_011138070.1	497	LOC105182389	eukaryota>metazoa>hexapoda	Harpegnathos	saltator	PREDICTED: protein disulfide-isomerase [Harpegnathos saltator]
XP_013778350.1	497	LOC106462929	eukaryota>metazoa	Limulus	polyphemus	PREDICTED: protein disulfide-isomerase 2-like [Limulus polyphemus]
XP_015159154.1	497	LOC102592089	eukaryota>viridiplantae	Solanum	tuberosum	PREDICTED: protein disulfide-isomerase-like [Solanum tuberosum]
XP_015159155.1	497	LOC102592089	eukaryota>viridiplantae	Solanum	tuberosum	PREDICTED: protein disulfide-isomerase-like [Solanum tuberosum]
Bcir1000001554	497	Bcir1000001554	eukaryota>fungi>mucoromycotina	Backusella	circina	>Bcir1000001554
XP_011778178.1	497	TbgDal_X10050	eukaryota>euglenozoa>kinetoplastida	Trypanosoma	brucei gambiense DAL972	bloodstream-specific protein 2 precursor,putative [Trypanosoma brucei gambiense DAL972]
OWA54404.1	497	BV898_18808	eukaryota>metazoa	Hypsibius	dujardini	Protein disulfide-isomerase [Hypsibius dujardini]
EPB91883.1	496	HMPREF1544_01177	eukaryota>fungi	Mucor	circinelloides f. circinelloides 1006PhL	hypothetical protein HMPREF1544_01177 [Mucor circinelloides f. circinelloides 1006PhL]
NP_001287070.1	496	CG6988,Dmel\CG6988,dpdi,Dpdi,EP(3)1022,pdi,PDI	eukaryota>metazoa>hexapoda	Drosophila	melanogaster	protein disulfide isomerase, isoform E [Drosophila melanogaster]
NP_524079.1	496	CG6988,Dmel\CG6988,dpdi,Dpdi,EP(3)1022,pdi,PDI	eukaryota>metazoa>hexapoda	Drosophila	melanogaster	protein disulfide isomerase, isoform A [Drosophila melanogaster]
NP_999697.1	496	ECaSt/PDI	eukaryota>metazoa>echinodermata	Strongylocentrotus	purpuratus	ER calcistorin precursor [Strongylocentrotus purpuratus]
XP_003545643.1	496	LOC100788702	eukaryota>viridiplantae	Glycine	max	PREDICTED: protein disulfide-isomerase-like isoform X1 [Glycine max]
XP_005647426.1	496	COCSUDRAFT_16178	eukaryota>viridiplantae>chlorophyta	Coccomyxa	subellipsoidea C-169	protein disulfide isomerase [Coccomyxa subellipsoidea C-169]
XP_006593329.1	496	LOC100305460	eukaryota>viridiplantae	Glycine	max	PREDICTED: protein disulfide isomerase like protein isoform X1 [Glycine max]
XP_008616243.1	496	SDRG_11966	eukaryota>stramenopiles	Saprolegnia	diclina VS20	hypothetical protein SDRG_11966 [Saprolegnia diclina VS20]
XP_009028587.1	496	HELRODRAFT_194128	eukaryota>metazoa>annelida	Helobdella	robusta	hypothetical protein HELRODRAFT_194128 [Helobdella robusta]
XP_012198778.1	496	SPRG_04464	eukaryota>stramenopiles	Saprolegnia	parasitica CBS 223.65	hypothetical protein SPRG_04464 [Saprolegnia parasitica CBS 223.65]
XP_017330169.1	496	pdia3	eukaryota>metazoa>chordata>vertebrata>actinopterygii	Ictalurus	punctatus	PREDICTED: protein disulfide-isomerase A3 [Ictalurus punctatus]
CEM39955.1	495	Vbra_19861	eukaryota>alveolata	Vitrella	brassicaformis CCMP3155	unnamed protein product [Vitrella brassicaformis CCMP3155]
XP_001761068.1	495	PHYPADRAFT_123708	eukaryota>viridiplantae	Physcomitrella	patens	predicted protein [Physcomitrella patens]
XP_003969188.1	495	LOC101067334	eukaryota>metazoa>chordata>vertebrata>actinopterygii	Takifugu	rubripes	PREDICTED: protein disulfide-isomerase A3-like [Takifugu rubripes]
XP_006593605.1	495	LOC100789569	eukaryota>viridiplantae	Glycine	max	PREDICTED: protein disulfide-isomerase-like [Glycine max]
XP_011445708.1	495	LOC105341095	eukaryota>metazoa>mollusca	Crassostrea	gigas	PREDICTED: protein disulfide-isomerase [Crassostrea gigas]
XP_016770060.1	495	LOC551435	eukaryota>metazoa>hexapoda	Apis	mellifera	PREDICTED: protein disulfide-isomerase [Apis mellifera]
XP_017337773.1	495	LOC108273190	eukaryota>metazoa>chordata>vertebrata>actinopterygii	Ictalurus	punctatus	PREDICTED: protein disulfide-isomerase A3-like [Ictalurus punctatus]
ORX80283.1	495	K493DRAFT_320917	eukaryota>fungi	Basidiobolus	meristosporus CBS 931.73	protein disulfide isomerase [Basidiobolus meristosporus CBS 931.73]
OAD04586.1	495	MUCCIDRAFT_177573	eukaryota>fungi	Mucor	circinelloides f. lusitanicus CBS 277.49	hypothetical protein MUCCIDRAFT_177573 [Mucor circinelloides f. lusitanicus CBS 277.49]
CBJ29913.1	494	Pdi3	eukaryota>stramenopiles	Ectocarpus	siliculosus	protein disulfide isomerase [Ectocarpus siliculosus]
NP_001037171.1	494	Pdi	eukaryota>metazoa>hexapoda	Bombyx	mori	protein disulfide isomerase precursor [Bombyx mori]
NP_001186666.1	494	Grp58,sb:cb825	eukaryota>metazoa>chordata>vertebrata>actinopterygii	Danio	rerio	protein disulfide-isomerase A3 precursor [Danio rerio]
XP_003967256.1	494	pdia3	eukaryota>metazoa>chordata>vertebrata>actinopterygii	Takifugu	rubripes	PREDICTED: protein disulfide-isomerase A3 [Takifugu rubripes]
Smar1000001458	494	Smar1000001458	eukaryota>metazoa>arthropoda>myriapoda	Strigamia	maritima	>Smar1000001458
NP_001003517.1	493	zgc:100906	eukaryota>metazoa>chordata>vertebrata>actinopterygii	Danio	rerio	protein disulfide-isomerase A3 precursor [Danio rerio]
NP_508778.1	493	pdi-2	eukaryota>metazoa>nematoda	Caenorhabditis	elegans	Protein disulfide-isomerase 2 [Caenorhabditis elegans]
XP_001784758.1	493	PHYPADRAFT_154382	eukaryota>viridiplantae	Physcomitrella	patens	predicted protein [Physcomitrella patens]
XP_001847538.1	493	CpipJ_CPIJ005219	eukaryota>metazoa>hexapoda	Culex	quinquefasciatus	disulfide isomerase [Culex quinquefasciatus]
XP_001864249.1	493	CpipJ_CPIJ013559	eukaryota>metazoa>hexapoda	Culex	quinquefasciatus	disulfide isomerase [Culex quinquefasciatus]
XP_002964632.1	493	SELMODRAFT_142613	eukaryota>viridiplantae	Selaginella	moellendorffii	hypothetical protein SELMODRAFT_142613 [Selaginella moellendorffii]
XP_011151095.1	493	LOC105190195	eukaryota>metazoa>hexapoda	Harpegnathos	saltator	PREDICTED: protein disulfide-isomerase A3 [Harpegnathos saltator]
XP_016605428.1	493	SPPG_07316	eukaryota>fungi>chytridiomycota	Spizellomyces	punctatus DAOM BR117	protein disulfide isomerase [Spizellomyces punctatus DAOM BR117]
XP_017327509.1	493	LOC108267688	eukaryota>metazoa>chordata>vertebrata>actinopterygii	Ictalurus	punctatus	PREDICTED: protein disulfide-isomerase A3-like isoform X1 [Ictalurus punctatus]
Bcir1000015014	493	Bcir1000015014	eukaryota>fungi>mucoromycotina	Backusella	circina	>Bcir1000015014
Uram1000005567	493	Uram1000005567	eukaryota>fungi>mucoromycotina	Umbelopsis	ramanniana	>Uram1000005567
XP_002788655.1	493	Pmar_PMAR010193	eukaryota>alveolata	Perkinsus	marinus ATCC 50983	protein disulfide isomerase, putative [Perkinsus marinus ATCC 50983]
NP_592871.1	492	SPAC1F5.02	eukaryota>fungi>ascomycota	Schizosaccharomyces	pombe	protein disulfide isomerase (predicted) [Schizosaccharomyces pombe]
XP_003380462.1	492	Tsp_06198	eukaryota>metazoa>nematoda	Trichinella	spiralis	protein disulfide-isomerase 2 [Trichinella spiralis]
XP_011453191.1	492	LOC105346349	eukaryota>metazoa>mollusca	Crassostrea	gigas	PREDICTED: protein disulfide-isomerase A3 [Crassostrea gigas]
XP_012053226.1	492	CNAG_06240	eukaryota>fungi>basidiomycota	Cryptococcus	neoformans var. grubii H99	protein disulfide-isomerase [Cryptococcus neoformans var. grubii H99]
XP_013389396.1	492	LOC106158083	eukaryota>metazoa	Lingula	anatina	PREDICTED: LOW QUALITY PROTEIN: protein disulfide-isomerase A3-like [Lingula anatina]
XP_014571096.1	492	L969DRAFT_84341	eukaryota>fungi>basidiomycota	Mixia	osmundae IAM 14324	hypothetical protein L969DRAFT_84341 [Mixia osmundae IAM 14324]
XP_018268022.1	492	RHOBADRAFT_66973	eukaryota>fungi>basidiomycota	Rhodotorula	graminis WP1	hypothetical protein RHOBADRAFT_66973 [Rhodotorula graminis WP1]
[illegible]						

NP 499385.3	1004	sca-1	eukaryota>metaz[illegible]	[illegible]	[illegible]
XP_001696690.1	1004	CHLREDRAFT_104709	eukaryota>virid[illegible]	[illegible]	[illegible]
ORX91705.1	1004	K493DRAFT 264186	eukaryota>fungi[illegible]	[illegible]	[illegible]
XP 004992033.1	1003	PTSG 06645	eukaryota>choan[illegible]	[illegible]	[illegible]
XP_005821433.1	1003	GUITHDRAFT_158817	eukaryota>crypt[illegible]	[illegible]	[illegible]
KFH69757.1	1003	MVEG 04563	eukaryota>fungi[illegible]	[illegible]	[illegible]
Crev1000001104	1003	Crev1000001104	eukaryota>fungi[illegible]	[illegible]	[illegible]
XP_002122235.2	1003	LOC100175957	eukaryota>metaz[illegible]	[illegible]	[illegible]
XP 009861042.2	1003	LOC100182988	eukaryota>metaz[illegible]	[illegible]	[illegible]
XP 009861043.2	1003	LOC100182988	eukaryota>metaz[illegible]	[illegible]	[illegible]
XP_003970855.1	1003	atp2a3	eukaryota>metaz[illegible]	[illegible]	[illegible]
XP 016770981.1	1003	LOC410371	eukaryota>metaz[illegible]	[illegible]	[illegible]
ORX95125.1	1003	K493DRAFT 283100	eukaryota>fungi[illegible]	[illegible]	[illegible]
XP_009496868.1	1002	H696_04730	eukaryota	[illegible]	[illegible]
XP 004226981.1	1002	LOC100180638	eukaryota>metaz[illegible]	[illegible]	[illegible]
XP 011663710.1	1002	SERCA	eukaryota>metaz[illegible]	[illegible]	[illegible]
NP_476832.1	1002	17136664,A4UZU0_DROME,ATC1_DROME,Ca-ATPase,Ca-p,Ca-P60,Ca-P60A,CA-P60A,CaP60A,Ca[2+]ATPase,CG3725,Dmel\CG3725,dserca,dSERCA,kum,Kum,l(2)k08308ab,l(2)k09025,l(2)SH0211,serca,Serca,SERCA2,Serca2a	eukaryota>metaz[illegible]	[illegible]	[illegible]
XP 016770982.1	1002	LOC410371	eukaryota>metaz[illegible]	[illegible]	[illegible]
XP 019697794.1	1002	LOC105184991	eukaryota>metaz[illegible]	[illegible]	[illegible]
XP_018296061.1	1001	PHYBLDRAFT_185726	eukaryota>fungi[illegible]	[illegible]	[illegible]
XP 013243079.1	1001	K437DRAFT 256669	eukaryota>fungi[illegible]	[illegible]	[illegible]
NP_775293.1	1001	ATP2A,SERCA1	eukaryota>metaz[illegible]	[illegible]	[illegible]
XP_015297988.1	1001	ATP2A1	eukaryota>metaz[illegible]	[illegible]	[illegible]
XP 006507331.1	1001	SERCA1	eukaryota>metaz[illegible]	[illegible]	[illegible]
XP_012360942.1	1001	ATP2A1	eukaryota>metaz[illegible]	[illegible]	[illegible]
XP 015839082.1	1001	LOC658328	eukaryota>metaz[illegible]	[illegible]	[illegible]
XP 002294029.1	1001	THAPSDRAFT 37662	eukaryota>stram[illegible]	[illegible]	[illegible]
XP_003524018.1	1001	LOC100783509	eukaryota>virid[illegible]	[illegible]	[illegible]
XP 001696782.1	1001	CHLREDRAFT_130931	eukaryota>virid[illegible]	[illegible]	[illegible]
XP 012647248.1	1000	BBM I00885	eukaryota>alveo[illegible]	[illegible]	[illegible]
OMH84339.1	1000	AX774_g2139	eukaryota>fungi[illegible]	[illegible]	[illegible]
XP 007326281.1	1000	AGABI1DRAFT_69139	eukaryota>fungi[illegible]	[illegible]	[illegible]
XP 009168054.1	1000	T265 04922	eukaryota>metaz[illegible]	[illegible]	[illegible]
XP_002127180.1	1000	LOC100180619	eukaryota>metaz[illegible]	[illegible]	[illegible]
XP 002119917.1	1000	LOC100180638	eukaryota>metaz[illegible]	[illegible]	[illegible]
XP 015839084.1	1000	LOC658328	eukaryota>metaz[illegible]	[illegible]	[illegible]
XP_012895050.1	1000	GSBLH_T00001230001	eukaryota>stram[illegible]	[illegible]	[illegible]
XP 001778460.1	1000	PHYPADRAFT 222630	eukaryota>virid[illegible]	[illegible]	[illegible]
XP 006347865.1	1000	LOC102595434	eukaryota>virid[illegible]	[illegible]	[illegible]
XP_010664492.1	1000	LOC100266003	eukaryota>virid[illegible]	[illegible]	[illegible]
XP 006458484.1	1000	AGABI2DRAFT 183521	eukaryota>fungi[illegible]	[illegible]	[illegible]
XP 013425005.1	999	M436DRAFT 84359	eukaryota>fungi[illegible]	[illegible]	[illegible]
Lhya1000006398	999	Lhya1000006398	eukaryota>fungi[illegible]	[illegible]	[illegible]
XP 015151158.1	999	SERCA	eukaryota>metaz[illegible]	[illegible]	[illegible]
XP_011536704.1	999	ATP2B,DAR,DD,SERCA2	eukaryota>metaz[illegible]	[illegible]	[illegible]
XP 005253945.1	999	ATP2B,DAR,DD,SERCA2	eukaryota>metaz[illegible]	[illegible]	[illegible]
NP 005164.2	999	SERCA3	eukaryota>metaz[illegible]	[illegible]	[illegible]
XP_015287004.1	999	ATP2A2	eukaryota>metaz[illegible]	[illegible]	[illegible]
XP 005572291.1	999	ATP2A2	eukaryota>metaz[illegible]	[illegible]	[illegible]
NP 001156808.1	999	Serca3,SERCA3b	eukaryota>metaz[illegible]	[illegible]	[illegible]
NP_001286796.1	999	17136664,A4UZU0_DROME,ATC1_DROME,Ca-ATPase,Ca-p,Ca-P60,Ca-P60A,CA-P60A,CaP60A,Ca[2+]ATPase,CG3725,Dmel\CG3725,dserca,dSERCA,kum,Kum,l(2)k08308ab,l(2)k09025,l(2)SH0211,serca,Serca,SERCA2,Serca2a	eukaryota>metaz[illegible]	[illegible]	[illegible]
OZJ04612.1	999	BZG36 02032	eukaryota>fungi[illegible]	[illegible]	[illegible]
ORY72275.1	999	LY90DRAFT 379315	eukaryota>fungi[illegible]	[illegible]	[illegible]
XP_001744818.1	998	MONBRDRAFT_7291	eukaryota>choan[illegible]	[illegible]	[illegible]
KXN70998.1	998	CONCODRAFT 6371	eukaryota>fungi[illegible]	[illegible]	[illegible]
XP 011121099.1	998	AOL s00076g244	eukaryota>fungi[illegible]	[illegible]	[illegible]
XP_013421506.1	998	LOC106181601	eukaryota>metaz[illegible]	[illegible]	[illegible]
[illegible]	[illegible]	[illegible]	[illegible]	[illegible]	[illegible]

XP_005156418.1	627	camk2ga,zgc:109908	eukaryota>metazoa>chordata>vertebrata>actinopterygii	Danio	rerio	calcium/calmodulin-dependent protein kinase type II subunit gamma isoform X1 [Danio rerio]
XP_006514532.1	627	Camk2b	eukaryota>metazoa>chordata>vertebrata	Mus	musculus	PREDICTED: calcium/calmodulin-dependent protein kinase type II subunit beta isoform X6 [Mus musculus]
XP_017306963.1	627	LOC108255499	eukaryota>metazoa>chordata>vertebrata>actinopterygii	Ictalurus	punctatus	PREDICTED: calcium/calmodulin-dependent protein kinase type II delta chain-like isoform X15 [Ictalurus punctatus]
XP_008664763.1	627	LOC103643377	eukaryota>viridiplantae	Zea	mays	CDPK-related kinase 3 [Zea mays]
XP_005156961.1	626	camk2g2	eukaryota>metazoa>chordata>vertebrata>actinopterygii	Danio	rerio	calcium/calmodulin-dependent protein kinase type II subunit gamma isoform X1 [Danio rerio]
XP_006514533.1	626	Camk2b	eukaryota>metazoa>chordata>vertebrata	Mus	musculus	PREDICTED: calcium/calmodulin-dependent protein kinase type II subunit beta isoform X7 [Mus musculus]
XP_006715844.1	626	CAM2,CAMK2,CAMKB	eukaryota>metazoa>chordata>vertebrata	Homo	sapiens	PREDICTED: calcium/calmodulin-dependent protein kinase type II subunit beta isoform X27 [Homo sapiens]
XP_002903060.1	626	PITG_09039	eukaryota>stramenopiles	Phytophthora	infestans T30-4	calcium/calmodulin-dependent protein kinase, putative [Phytophthora infestans T30-4]
XP_011121085.1	626	AOL_s00076g230	eukaryota>fungi>ascomycota	Arthrobotrys	oligospora ATCC 24927	hypothetical protein AOL_s00076g230 [Arthrobotrys oligospora ATCC 24927]
XP_015833937.1	625	LOC655266	eukaryota>metazoa>hexapoda	Tribolium	castaneum	PREDICTED: calcium/calmodulin-dependent protein kinase type II alpha chain isoform X1 [Tribolium castaneum]
NP_001105740.1	625	GRMZM2G115518,MCK1	eukaryota>viridiplantae	Zea	mays	calcium dependent protein kinase 1 [Zea mays]
XP_012757847.1	625	SAMD00019534_018020	eukaryota>amoebozoa>mycetozoa>dictyosteliida	Acytostelium	subglobosum LB1	hypothetical protein SAMD00019534_018020 [Acytostelium subglobosum LB1]
XP_005156419.1	624	camk2ga,zgc:109908	eukaryota>metazoa>chordata>vertebrata>actinopterygii	Danio	rerio	calcium/calmodulin-dependent protein kinase type II subunit gamma isoform X2 [Danio rerio]
XP_017338896.1	624	LOC108273829	eukaryota>metazoa>chordata>vertebrata>actinopterygii	Ictalurus	punctatus	PREDICTED: calcium/calmodulin-dependent protein kinase type II subunit gamma isoform X1 [Ictalurus punctatus]
CBJ27073.1	624	CDPK2	eukaryota>stramenopiles	Ectocarpus	siliculosus	conserved unknown protein [Ectocarpus siliculosus]
XP_009305633.1	623	camk2g2	eukaryota>metazoa>chordata>vertebrata>actinopterygii	Danio	rerio	calcium/calmodulin-dependent protein kinase type II subunit gamma isoform X2 [Danio rerio]
XP_015833938.1	623	LOC655266	eukaryota>metazoa>hexapoda	Tribolium	castaneum	PREDICTED: calcium/calmodulin-dependent protein kinase type II alpha chain isoform X2 [Tribolium castaneum]
XP_018106263.1	623	cam2,camk2,camk2b,camkb	eukaryota>metazoa>chordata>vertebrata	Xenopus	laevis	PREDICTED: calcium/calmodulin-dependent protein kinase (CaM kinase) II beta L homeolog isoform X1 [Xenopus laevis]
XP_018106264.1	623	cam2,camk2,camk2b,camkb	eukaryota>metazoa>chordata>vertebrata	Xenopus	laevis	PREDICTED: calcium/calmodulin-dependent protein kinase (CaM kinase) II beta L homeolog isoform X2 [Xenopus laevis]
XP_018109671.1	623	camk2,camk2a,camk2b	eukaryota>metazoa>chordata>vertebrata	Xenopus	laevis	PREDICTED: calcium/calmodulin-dependent protein kinase II beta isoform X1 [Xenopus laevis]
XP_018109672.1	623	camk2,camk2a,camk2b	eukaryota>metazoa>chordata>vertebrata	Xenopus	laevis	PREDICTED: calcium/calmodulin-dependent protein kinase II beta isoform X2 [Xenopus laevis]
EPY26434.1	623	AGDE_11328	eukaryota>euglenozoa>kinetoplastida	Angomonas	deanei	protein kinase [Angomonas deanei]
XP_012656331.1	623	TTHERM_000841299	eukaryota>alveolata>ciliophora	Tetrahymena	thermophila SB210	calmodulin-domain kinase [Tetrahymena thermophila SB210]
XP_018080932.1	622	LOC108696257	eukaryota>metazoa>chordata>vertebrata	Xenopus	laevis	PREDICTED: calcium/calmodulin-dependent protein kinase type II subunit gamma-like isoform X3 [Xenopus laevis]
XP_016496330.1	622	LOC107815283	eukaryota>viridiplantae	Nicotiana	tabacum	PREDICTED: calcium-dependent protein kinase 26-like [Nicotiana tabacum]
XP_016510658.1	622	LOC107827935	eukaryota>viridiplantae	Nicotiana	tabacum	PREDICTED: calcium-dependent protein kinase 26-like [Nicotiana tabacum]
XP_003884375.1	622	NCLIV_047750	eukaryota>alveolata>apicomplexa	Neospora	caninum Liverpool	hypothetical protein NCLIV_047750 [Neospora caninum Liverpool]
XP_001388076.1	622	cgd4_3330	eukaryota>alveolata>apicomplexa	Cryptosporidium	parvum Iowa II	hypothetical protein, partial [Cryptosporidium parvum Iowa II]
XP_012759150.1	622	SAMD00019534_002620	eukaryota>amoebozoa>mycetozoa>dictyosteliida	Acytostelium	subglobosum LB1	hypothetical protein SAMD00019534_002620 [Acytostelium subglobosum LB1]
XP_017338897.1	621	LOC108273829	eukaryota>metazoa>chordata>vertebrata>actinopterygii	Ictalurus	punctatus	PREDICTED: calcium/calmodulin-dependent protein kinase type II subunit gamma isoform X2 [Ictalurus punctatus]
CDW74871.1	621	Contig12712.g13564	eukaryota>alveolata>ciliophora	Stylonychia	lemnae	protein kinase domain containing protein [Stylonychia lemnae]
XP_018080933.1	620	LOC108696257	eukaryota>metazoa>chordata>vertebrata	Xenopus	laevis	PREDICTED: calcium/calmodulin-dependent protein kinase type II subunit gamma-like isoform X4 [Xenopus laevis]
CEM28668.1	619	Vbra_17763	eukaryota>alveolata	Vitrella	brassicaformis CCMP3155	unnamed protein product [Vitrella brassicaformis CCMP3155]
XP_006560571.1	617	Camkii	eukaryota>metazoa>hexapoda	Apis	mellifera	PREDICTED: calcium/calmodulin-dependent protein kinase II isoform X4 [Apis mellifera]
XP_015833939.1	617	LOC655266	eukaryota>metazoa>hexapoda	Tribolium	castaneum	PREDICTED: calcium/calmodulin-dependent protein kinase type II alpha chain isoform X3 [Tribolium castaneum]
XP_018082303.1	617	camk2,camk2g	eukaryota>metazoa>chordata>vertebrata	Xenopus	laevis	PREDICTED: calcium/calmodulin-dependent protein kinase (CaM kinase) II gamma S homeolog isoform X1 [Xenopus laevis]
XP_002774607.1	616	Pmar_PMAR006234	eukaryota>alveolata	Perkinsus	marinus ATCC 50983	calcium-dependent protein kinase, putative [Perkinsus marinus ATCC 50983]
XP_006560572.1	615	Camkii	eukaryota>metazoa>hexapoda	Apis	mellifera	PREDICTED: calcium/calmodulin-dependent protein kinase II isoform X5 [Apis mellifera]
XP_006560573.1	615	Camkii	eukaryota>metazoa>hexapoda	Apis	mellifera	PREDICTED: calcium/calmodulin-dependent protein kinase II isoform X6 [Apis mellifera]
XP_015833940.1	615	LOC655266	eukaryota>metazoa>hexapoda	Tribolium	castaneum	PREDICTED: calcium/calmodulin-dependent protein kinase type II alpha chain isoform X4 [Tribolium castaneum]
XP_019081311.1	615	LOC100250518	eukaryota>viridiplantae	Vitis	vinifera	PREDICTED: CDPK-related kinase 3 isoform X2 [Vitis vinifera]
XP_016872205.1	615	CAMK,CAMK-II,CAMKG	eukaryota>metazoa>chordata>vertebrata	Homo	sapiens	PREDICTED: calcium/calmodulin-dependent protein kinase type II subunit gamma isoform X1 [Homo sapiens]
EJY84673.1	615	OXYTRI_17480	eukaryota>alveolata>ciliophora	Oxytricha	trifallax	Protein kinase domain containing protein (macronuclear) [Oxytricha trifallax]
XP_001691570.1	613	CDPK1	eukaryota>viridiplantae>chlorophyta	Chlamydomonas	reinhardtii	calcium-dependent protein kinase 1 [Chlamydomonas reinhardtii]
CBN78721.1	613	PK	eukaryota>stramenopiles	Ectocarpus	siliculosus	calmodulin-dependent protein kinase I [Ectocarpus siliculosus]
XP_009299603.1	612	camk2b,CAMK2Gb,fj90d04,si:dkey-174n20.2,wu:fj90d04,zgc:165373	eukaryota>metazoa>chordata>vertebrata>actinopterygii	Danio	rerio	calcium/calmodulin-dependent protein kinase type II subunit beta isoform X20 [Danio rerio]
XP_002949744.1	612	VOLCADRAFT_74309	eukaryota>viridiplantae>chlorophyta	Volvox	carteri f. nagariensis	hypothetical protein VOLCADRAFT_74309 [Volvox carteri f. nagariensis]
XP_012759910.1	612	SAMD00019534_035640	eukaryota>amoebozoa>mycetozoa>dictyosteliida	Acytostelium	subglobosum LB1	hypothetical protein SAMD00019534_035640 [Acytostelium subglobosum LB1]
XP_005156962.1	611	camk2g2	eukaryota>metazoa>chordata>vertebrata>actinopterygii	Danio	rerio	calcium/calmodulin-dependent protein kinase type II subunit gamma isoform X3 [Danio rerio]
CEM12751.1	611	Vbra_15452	eukaryota>alveolata	Vitrella	brassicaformis CCMP3155	unnamed protein product [Vitrella brassicaformis CCMP3155]
NP_196107.1	610	ATCPK1,CALCIUM,DEPENDENT,PROTEIN,KINASE,calcium,dependent,protein,kinase,1	eukaryota>viridiplantae	Arabidopsis	thaliana	calcium dependent protein kinase 1 [Arabidopsis thaliana]
XP_016482898.1	610	LOC107803647	eukaryota>viridiplantae	Nicotiana	tabacum	PREDICTED: calcium-dependent protein kinase 26-like [Nicotiana tabacum]
XP_006338215.1	610	LOC102588824	eukaryota>viridiplantae	Solanum	tuberosum	PREDICTED: CDPK-related kinase 5 [Solanum tuberosum]
XP_017306964.1	610	LOC108255499	eukaryota>metazoa>chordata>vertebrata>actinopterygii	Ictalurus	punctatus	PREDICTED: calcium/calmodulin-dependent protein kinase type II subunit gamma-like isoform X16 [Ictalurus punctatus]
XP_008674183.1	609	LOC103650380	eukaryota>viridiplantae	Zea	mays	calcium-dependent protein kinase 27 [Zea mays]
XP_009305634.1	608	camk2g2	eukaryota>metazoa>chordata>vertebrata>actinopterygii	Danio	rerio	calcium/calmodulin-dependent protein kinase type II subunit gamma isoform X5 [Danio rerio]
XP_015143790.1	608	CAMK2G	eukaryota>metazoa>chordata>vertebrata	Gallus	gallus	PREDICTED: calcium/calmodulin-dependent protein kinase type II subunit gamma isoform X1 [Gallus gallus]
XP_015143791.1	608	CAMK2G	eukaryota>metazoa>chordata>vertebrata	Gallus	gallus	PREDICTED: calcium/calmodulin-dependent protein kinase type II subunit gamma isoform X1 [Gallus gallus]
XP_018106265.1	608	cam2,camk2,camk2b,camkb	eukaryota>metazoa>chordata>vertebrata	Xenopus	laevis	PREDICTED: calcium/calmodulin-dependent protein kinase (CaM kinase) II beta L homeolog isoform X3 [Xenopus laevis]
XP_018109673.1	608	camk2,camk2a,camk2b	eukaryota>metazoa>chordata>vertebrata	Xenopus	laevis	PREDICTED: calcium/calmodulin-dependent protein kinase II beta isoform X3 [Xenopus laevis]
XP_015833941.1	607	LOC655266	eukaryota>metazoa>hexapoda	Tribolium	castaneum	PREDICTED: calcium/calmodulin-dependent protein kinase type II alpha chain isoform X5 [Tribolium castaneum]
XP_006339179.2	607	LOC102589594	eukaryota>viridiplantae	Solanum	tuberosum	PREDICTED: calcium-dependent protein kinase 26-like [Solanum tuberosum]
XP_016479782.1	607	LOC107801034	eukaryota>viridiplantae	Nicotiana	tabacum	PREDICTED: calcium-dependent protein kinase 26-like [Nicotiana tabacum]
XP_016479783.1	607	LOC107801034	eukaryota>viridiplantae	Nicotiana	tabacum	PREDICTED: calcium-dependent protein kinase 26-like [Nicotiana tabacum]
NP_001105305.1	607	cdpk*-D84508	eukaryota>viridiplantae	Zea	mays	LOC542225 [Zea mays]
XP_016872206.1	607	CAMK,CAMK-II,CAMKG	eukaryota>metazoa>chordata>vertebrata	Homo	sapiens	PREDICTED: calcium/calmodulin-dependent protein kinase type II subunit gamma isoform X2 [Homo sapiens]
XP_011513860.1	607	CAM2,CAMK2,CAMKB	eukaryota>metazoa>chordata>vertebrata	Homo	sapiens	PREDICTED: calcium/calmodulin-dependent protein kinase type II subunit beta isoform X13 [Homo sapiens]
XP_005156420.1	606	camk2ga,zgc:109908	eukaryota>metazoa>chordata>vertebrata>actinopterygii	Danio	rerio	calcium/calmodulin-dependent protein kinase type II subunit gamma isoform X3 [Danio rerio]
XP_015833942.1	606	LOC655266	eukaryota>metazoa>hexapoda	Tribolium	castaneum	PREDICTED: calcium/calmodulin-dependent protein kinase type II alpha chain isoform X6 [Tribolium castaneum]
XP_016663543.1	606	LOC100161639	eukaryota>metazoa>hexapoda	Acyrthosiphon	pisum	PREDICTED: calcium/calmodulin-dependent protein kinase type II delta chain, partial [Acyrthosiphon pisum]
XP_016767049.1	606	Camkii	eukaryota>metazoa>hexapoda	Apis	mellifera	PREDICTED: calcium/calmodulin-dependent protein kinase II isoform X7 [Apis mellifera]
XP_018082304.1	606	camk2,camk2g	eukaryota>metazoa>chordata>vertebrata	Xenopus	laevis	PREDICTED: calcium/calmodulin-dependent protein kinase (CaM kinase) II gamma S homeolog isoform X2 [Xenopus laevis]
NP_175381.1	606	F14J22.18,F14J22_18	eukaryota>viridiplantae	Arabidopsis	thaliana	Calcium-dependent protein kinase (CDPK) family protein [Arabidopsis thaliana]
XP_016610836.1	606	SPPG_01878	eukaryota>fungi>chytridiomycota	Spizellomyces	punctatus DAOM BR117	CAMK protein kinase [Spizellomyces punctatus DAOM BR117]
XP_005156963.1	605	camk2g2	eukaryota>metazoa>chordata>vertebrata>actinopterygii	Danio	rerio	calcium/calmodulin-dependent protein kinase type II subunit gamma isoform X6 [Danio rerio]
XP_017319823.1	605	camk2g	eukaryota>metazoa>chordata>vertebrata>actinopterygii	Ictalurus	punctatus	PREDICTED: calcium/calmodulin-dependent protein kinase type II subunit gamma isoform X1 [Ictalurus punctatus]
XP_018080934.1	605	LOC108696257	eukaryota>metazoa>chordata>vertebrata	Xenopus	laevis	PREDICTED: calcium/calmodulin-dependent protein kinase type II subunit gamma-like isoform X5 [Xenopus laevis]
ETO24613.1	605	RFI_12547	eukaryota>rhizaria	Reticulomyxa	filosa	calcium/calmodulin-dependent protein kinase type 1 (camki) [Reticulomyxa filosa]
XP_014631600.1	604	LOC100794177	eukaryota>viridiplantae	Glycine	max	PREDICTED: CDPK-related kinase 5-like isoform X1 [Glycine max]
XP_016872207.1	604	CAMK,CAMK-II,CAMKG	eukaryota>metazoa>chordata>vertebrata	Homo	sapiens	PREDICTED: calcium/calmodulin-dependent protein kinase type II subunit gamma isoform X3 [Homo sapiens]
XP_002366770.1	604	CDPK2B	eukaryota>alveolata>apicomplexa	Toxoplasma	gondii ME49	calcium-dependent protein kinase CDPK2B [Toxoplasma gondii ME49]
XP_008884424.1	604	HHA_242400	eukaryota>alveolata>apicomplexa	Hammondia	hammondi	calcium-dependent protein kinase CDPK2B [Hammondia hammondi]
XP_016868151.1	603	CAM2,CAMK2,CAMKB	eukaryota>metazoa>chordata>vertebrata	Homo	sapiens	PREDICTED: calcium/calmodulin-dependent protein kinase type II subunit beta isoform X14 [Homo sapiens]
XP_017338898.1	603	LOC108273829	eukaryota>metazoa>chordata>vertebrata>actinopterygii	Ictalurus	punctatus	PREDICTED: calcium/calmodulin-dependent protein kinase type II delta chain isoform X3 [Ictalurus punctatus]
XP_017319824.1	602	camk2g	eukaryota>metazoa>chordata>vertebrata>actinopterygii	Ictalurus	punctatus	PREDICTED: calcium/calmodulin-dependent protein kinase type II subunit gamma isoform X2 [Ictalurus punctatus]
NP_001146922.1	602	GRMZM2G109843	eukaryota>viridiplantae	Zea	mays	uncharacterized protein LOC100280531 [Zea mays]
XP_002262855.3	602	LOC100264733	eukaryota>viridiplantae	Vitis	vinifera	PREDICTED: CDPK-related kinase 5 [Vitis vinifera]
XP_016447921.1	602	LOC107772979	eukaryota>viridiplantae	Nicotiana	tabacum	PREDICTED: CDPK-related kinase 5 isoform X1 [Nicotiana tabacum]
XP_016508430.1	602	LOC107826012	eukaryota>viridiplantae	Nicotiana	tabacum	PREDICTED: CDPK-related kinase 5-like [Nicotiana tabacum]
XP_001613119.1	602	PVX_119610	[illegible]	[illegible]	[illegible]	[illegible]
CEM08065.1	602	Vbra_14581	[illegible]	[illegible]	[illegible]	[illegible]
CEM22071.1	602	Vbra_9613	[illegible]	[illegible]	[illegible]	[illegible]
ETO17436.1	602	RFI_19885	[illegible]	[illegible]	[illegible]	[illegible]
XP_018080936.1	601	LOC108696257	[illegible]	[illegible]	[illegible]	[illegible]
NP_190622.1	601	CDPK-related,kinase	[illegible]	[illegible]	[illegible]	[illegible]
XP_016872208.1	601	CAMK,CAMK-II,CAMKG	[illegible]	[illegible]	[illegible]	[illegible]
XP_004032254.1	601	IMG5_126490	[illegible]	[illegible]	[illegible]	[illegible]
XP_016767050.1	600	Camkii	[illegible]	[illegible]	[illegible]	[illegible]
XP_006588896.1	600	LOC100813232	[illegible]	[illegible]	[illegible]	[illegible]
XP_010645740.1	600	LOC100253693	[illegible]	[illegible]	[illegible]	[illegible]
XP_013232711.1	600	ETH_00010685	[illegible]	[illegible]	[illegible]	[illegible]
XP_018080937.1	599	LOC108696257	[illegible]	[illegible]	[illegible]	[illegible]
XP_018106266.1	599	cam2,camk2,camk2b,camkb	[illegible]	[illegible]	[illegible]	[illegible]
XP_018106267.1	599	cam2,camk2,camk2b,camkb	[illegible]	[illegible]	[illegible]	[illegible]
XP_018109674.1	599	camk2,camk2a,camk2b	[illegible]	[illegible]	[illegible]	[illegible]
XP_018109675.1	599	camk2,camk2a,camk2b	[illegible]	[illegible]	[illegible]	[illegible]
XP_001456018.1	599	GSPATT00021789001	[illegible]	[illegible]	[illegible]	[illegible]
NP_188541.1	599	AT3G19100	[illegible]	[illegible]	[illegible]	[illegible]
XP_003526744.1	599	LOC100794478	[illegible]	[illegible]	[illegible]	[illegible]
XP_016484519.1	599	LOC107805057	[illegible]	[illegible]	[illegible]	[illegible]
XP_016507739.1	599	LOC107825396	[illegible]	[illegible]	[illegible]	[illegible]
OLQ04095.1	599	CPK2	[illegible]	[illegible]	[illegible]	[illegible]
XP_005156421.1	598	camk2ga,zgc:109908	[illegible]	[illegible]	[illegible]	[illegible]
XP_006348435.2	598	LOC102596327	[illegible]	[illegible]	[illegible]	[illegible]
XP_001773184.1	598	crk2	[illegible]	[illegible]	[illegible]	[illegible]
XP_003520170.1	598	LOC100807672	[illegible]	[illegible]	[illegible]	[illegible]
XP_003528501.1	598	LOC100798913	[illegible]	[illegible]	[illegible]	[illegible]
XP_006347491.1	598	LOC102581410	[illegible]	[illegible]	[illegible]	[illegible]
XP_008658484.1	598	GRMZM2G117796	[illegible]	[illegible]	[illegible]	[illegible]
XP_008668447.1	598	GRMZM2G056612	[illegible]	[illegible]	[illegible]	[illegible]
CAG05367.1	597	GSTEN:00025360:G:001	[illegible]	[illegible]	[illegible]	[illegible]

[illegible]

XP_002769463.1	535	Pmar PMAR008962	eukaryota>alveolata	Perkinsus	marinus ATCC 50983	calcium-dependent protein kinase 1, putative [Perkinsus marinus ATCC 50983]
XP_009304937.1	534	camk2d,camk2da,wu:fq17a12,zgc:92792	eukaryota>metazoa>chordata>vertebrata>actinopterygii	Danio	rerio	calcium/calmodulin-dependent protein kinase type II delta 2 chain isoform X5 [Danio rerio]
NP_001132098.1	534	LOC100193514	eukaryota>viridiplantae	Zea	mays	uncharacterized protein LOC100193514 [Zea mays]
XP_002276630.2	534	LOC100251670	eukaryota>viridiplantae	Vitis	vinifera	PREDICTED: calcium-dependent protein kinase 34 [Vitis vinifera]
XP_003521182.1	534	LOC100808399	eukaryota>viridiplantae	Glycine	max	PREDICTED: calcium-dependent protein kinase 32-like [Glycine max]
XP_016477399.1	534	LOC107798882	eukaryota>viridiplantae	Nicotiana	tabacum	PREDICTED: calcium-dependent protein kinase 21-like [Nicotiana tabacum]
XP_016479757.1	534	LOC107801007	eukaryota>viridiplantae	Nicotiana	tabacum	PREDICTED: calcium-dependent protein kinase 8-like [Nicotiana tabacum]
NP_195331.2	534	calcium-dependent,protein,kinase,18,T19K4.200,T19K4_200	eukaryota>viridiplantae	Arabidopsis	thaliana	calcium-dependent protein kinase 18 [Arabidopsis thaliana]
NP_001023297.1	533	unc-43	eukaryota>metazoa>nematoda	Caenorhabditis	elegans	Calcium/calmodulin-dependent protein kinase type II [Caenorhabditis elegans]
NP_001308498.1	533	CAMKD	eukaryota>metazoa>chordata>vertebrata	Homo	sapiens	calcium/calmodulin-dependent protein kinase type II subunit delta isoform 9 [Homo sapiens]
NP_001308500.1	533	CAMKD	eukaryota>metazoa>chordata>vertebrata	Homo	sapiens	calcium/calmodulin-dependent protein kinase type II subunit delta isoform 11 [Homo sapiens]
XP_003269380.1	533	CAMK2D	eukaryota>metazoa>chordata>vertebrata	Nomascus	leucogenys	PREDICTED: calcium/calmodulin-dependent protein kinase type II subunit delta isoform X1 [Nomascus leucogenys]
XP_005565529.1	533	CAMK2G	eukaryota>metazoa>chordata>vertebrata	Macaca	fascicularis	PREDICTED: calcium/calmodulin-dependent protein kinase type II subunit gamma isoform X12 [Macaca fascicularis]
XP_006500879.1	533	2810011D23Rik,8030469K03Rik,CaMK,II,[d]-CaMKII	eukaryota>metazoa>chordata>vertebrata	Mus	musculus	PREDICTED: calcium/calmodulin-dependent protein kinase type II subunit delta isoform X1 [Mus musculus]
XP_006500880.1	533	2810011D23Rik,8030469K03Rik,CaMK,II,[d]-CaMKII	eukaryota>metazoa>chordata>vertebrata	Mus	musculus	PREDICTED: calcium/calmodulin-dependent protein kinase type II subunit delta isoform X2 [Mus musculus]
XP_006518560.1	533	Camkg	eukaryota>metazoa>chordata>vertebrata	Mus	musculus	PREDICTED: calcium/calmodulin-dependent protein kinase type II subunit gamma isoform X10 [Mus musculus]
XP_009304938.1	533	camk2d,camk2da,wu:fq17a12,zgc:92792	eukaryota>metazoa>chordata>vertebrata>actinopterygii	Danio	rerio	calcium/calmodulin-dependent protein kinase type II delta 2 chain isoform X6 [Danio rerio]
XP_013779505.1	533	LOC106463959	eukaryota>metazoa	Limulus	polyphemus	calcium/calmodulin-dependent protein kinase type II alpha chain isoform X2 [Limulus polyphemus]
XP_015305988.1	533	CAMK2D	eukaryota>metazoa>chordata>vertebrata	Macaca	fascicularis	PREDICTED: calcium/calmodulin-dependent protein kinase type II subunit delta isoform X1 [Macaca fascicularis]
XP_015305989.1	533	CAMK2D	eukaryota>metazoa>chordata>vertebrata	Macaca	fascicularis	PREDICTED: calcium/calmodulin-dependent protein kinase type II subunit delta isoform X2 [Macaca fascicularis]
GAQ88622.1	533	KFL 004450090	eukaryota>viridiplantae	Klebsormidium	nitens	Serine Threonine protein kinase [Klebsormidium nitens]
NP_001190672.1	533	ATCPK23,calcium-dependent,protein,kinase,23,T4B21.15,T4B21_15	eukaryota>viridiplantae	Arabidopsis	thaliana	calcium-dependent protein kinase 23 [Arabidopsis thaliana]
NP_197446.1	533	calcium-dependent,protein,kinase,19,CALCIUM-DEPENDENT,PROTEIN,KINASE,19,CPK8,F7K24.200,F7K24_200	eukaryota>viridiplantae	Arabidopsis	thaliana	calcium-dependent protein kinase 19 [Arabidopsis thaliana]
NP_850853.1	533	calcium-dependent,protein,kinase,19,CALCIUM-DEPENDENT,PROTEIN,KINASE,19,CPK8,F7K24.200,F7K24_200	eukaryota>viridiplantae	Arabidopsis	thaliana	calcium-dependent protein kinase 19 [Arabidopsis thaliana]
XP_003529159.1	533	LOC100783922	eukaryota>viridiplantae	Glycine	max	PREDICTED: calcium-dependent protein kinase 13 [Glycine max]
XP_003540704.1	533	LOC100801716	eukaryota>viridiplantae	Glycine	max	PREDICTED: calcium-dependent protein kinase 24-like [Glycine max]
XP_006366539.1	533	LOC102594770	eukaryota>viridiplantae	Solanum	tuberosum	PREDICTED: calcium-dependent protein kinase 8-like [Solanum tuberosum]
XP_001434254.1	533	GSPATT00035988001	eukaryota>alveolata>ciliophora	Paramecium	tetraurelia strain d4-2	hypothetical protein (macronuclear) [Paramecium tetraurelia strain d4-2]
XP_001438645.1	533	GSPATT00000961001	eukaryota>alveolata>ciliophora	Paramecium	tetraurelia strain d4-2	hypothetical protein (macronuclear) [Paramecium tetraurelia strain d4-2]
XP_001439733.1	533	GSPATT00008778001	eukaryota>alveolata>ciliophora	Paramecium	tetraurelia strain d4-2	hypothetical protein (macronuclear) [Paramecium tetraurelia strain d4-2]
XP_016872218.1	533	CAMK,CAMK-II,CAMKG	eukaryota>metazoa>chordata>vertebrata	Homo	sapiens	PREDICTED: calcium/calmodulin-dependent protein kinase type II subunit gamma isoform X25 [Homo sapiens]
XP_005263310.1	532	CAMKD	eukaryota>metazoa>chordata>vertebrata	Homo	sapiens	PREDICTED: calcium/calmodulin-dependent protein kinase type II subunit delta isoform X1 [Homo sapiens]
XP_005263311.1	532	CAMKD	eukaryota>metazoa>chordata>vertebrata	Homo	sapiens	PREDICTED: calcium/calmodulin-dependent protein kinase type II subunit delta isoform X2 [Homo sapiens]
XP_006500881.1	532	2810011D23Rik,8030469K03Rik,CaMK,II,[d]-CaMKII	eukaryota>metazoa>chordata>vertebrata	Mus	musculus	PREDICTED: calcium/calmodulin-dependent protein kinase type II subunit delta isoform X3 [Mus musculus]
XP_015305990.1	532	CAMK2D	eukaryota>metazoa>chordata>vertebrata	Macaca	fascicularis	PREDICTED: calcium/calmodulin-dependent protein kinase type II subunit delta isoform X3 [Macaca fascicularis]
XP_015305991.1	532	CAMK2D	eukaryota>metazoa>chordata>vertebrata	Macaca	fascicularis	PREDICTED: calcium/calmodulin-dependent protein kinase type II subunit delta isoform X4 [Macaca fascicularis]
XP_017174904.1	532	2810011D23Rik,8030469K03Rik,CaMK,II,[d]-CaMKII	eukaryota>metazoa>chordata>vertebrata	Mus	musculus	PREDICTED: calcium/calmodulin-dependent protein kinase type II subunit delta isoform X4 [Mus musculus]
XP_017306968.1	532	LOC108255499	eukaryota>metazoa>chordata>vertebrata>actinopterygii	Ictalurus	punctatus	PREDICTED: calcium/calmodulin-dependent protein kinase type II subunit beta-like isoform X20 [Ictalurus punctatus]
NP_001275322.1	532	CDPK	eukaryota>viridiplantae	Solanum	tuberosum	calcium-dependent protein kinase [Solanum tuberosum]
XP_002983821.1	532	CPK3-2	eukaryota>viridiplantae	Selaginella	moellendorffii	calcium-dependent protein kinase 3 [Selaginella moellendorffii]
XP_002989102.1	532	CPK3-1	eukaryota>viridiplantae	Selaginella	moellendorffii	calcium dependent protein kinase [Selaginella moellendorffii]
XP_006351684.1	532	LOC102602736	eukaryota>viridiplantae	Solanum	tuberosum	PREDICTED: calcium-dependent protein kinase 7-like [Solanum tuberosum]
XP_015166078.1	532	LOC102602736	eukaryota>viridiplantae	Solanum	tuberosum	PREDICTED: calcium-dependent protein kinase 7-like [Solanum tuberosum]
XP_016470886.1	532	LOC107793116	eukaryota>viridiplantae	Nicotiana	tabacum	PREDICTED: calcium-dependent protein kinase 34-like isoform X1 [Nicotiana tabacum]
XP_019077317.1	532	LOC100258857	eukaryota>viridiplantae	Vitis	vinifera	PREDICTED: calcium-dependent protein kinase 32 isoform X1 [Vitis vinifera]
XP_001434671.1	532	GSPATT00006580001	eukaryota>alveolata>ciliophora	Paramecium	tetraurelia strain d4-2	hypothetical protein (macronuclear) [Paramecium tetraurelia strain d4-2]
XP_001438861.1	532	GSPATT00008011001	eukaryota>alveolata>ciliophora	Paramecium	tetraurelia strain d4-2	hypothetical protein (macronuclear) [Paramecium tetraurelia strain d4-2]
XP_019075935.1	532	LOC100267353	eukaryota>viridiplantae	Vitis	vinifera	PREDICTED: calcium-dependent protein kinase 8 isoform X2 [Vitis vinifera]
NP_001162833.1	531	Ca2+/calmodulin-dependent,protein,kinase,calcium/calmodulin-dependent,protein,kinase,II,Cam,CaM,CaM,II,CaM,KII,CaM,kinase,CaMK,II,CaMK-II,camkII,camKII,Camkii,CamKII,CA[illegible]Fb,l(4)16,l(4)ar	eukaryota>metazoa>hexapoda	Drosophila	melanogaster	Calcium/calmodulin-dependent protein kinase II, isoform J [Drosophila melanogaster]
NP_001258322.1	531	camk2b,CAMK2Gb,fj90d04,si:dkey-174n20.2,wu:fj90d04,zgc:165373	eukaryota>metazoa>chordata>vertebrata>actinopterygii	Danio	rerio	calcium/calmodulin-dependent protein kinase type II subunit beta [Danio rerio]
XP_016767053.1	531	Camkii	eukaryota>metazoa>hexapoda	Apis	mellifera	PREDICTED: calcium/calmodulin-dependent protein kinase II isoform X18 [Apis mellifera]
XP_018654510.1	531	Smp 011660.1	eukaryota>metazoa	Schistosoma	mansoni	protein kinase [Schistosoma mansoni]
NP_001105306.1	531	GRMZM2G168706	eukaryota>viridiplantae	Zea	mays	calcium-dependent protein kinase [Zea mays]
NP_001319867.1	531	AtCPK21,calcium-dependent,protein,kinase,21,T4B21.13,T4B21_13	eukaryota>viridiplantae	Arabidopsis	thaliana	calcium-dependent protein kinase 21 [Arabidopsis thaliana]
NP_192381.1	531	AtCPK21,calcium-dependent,protein,kinase,21,T4B21.13,T4B21_13	eukaryota>viridiplantae	Arabidopsis	thaliana	calcium-dependent protein kinase 21 [Arabidopsis thaliana]
XP_002976252.1	531	CPK13-1	eukaryota>viridiplantae	Selaginella	moellendorffii	calcium-dependent protein kinase 7 [Selaginella moellendorffii]
XP_002987149.1	531	CPK13-2	eukaryota>viridiplantae	Selaginella	moellendorffii	calcium dependent protein kinase [Selaginella moellendorffii]
[illegible]	[illegible]	[illegible]	[illegible]	[illegible]	[illegible]	[illegible]

OMJ88622.1	496	SteCoe_9402	eukaryota>alveolata>ciliophora	Stentor	coeruleus	hypothetical protein SteCoe_9402 [Stentor coeruleus]
XP_001452952.1	496	GSPATT00019272001	eukaryota>alveolata>ciliophora	Paramecium	tetraurelia strain d4-2	hypothetical protein (macronuclear) [Paramecium tetraurelia strain d4-2]
XP_001012485.2	496	TTHERM_01052880	eukaryota>alveolata>ciliophora	Tetrahymena	thermophila SB210	calmodulin-domain kinase [Tetrahymena thermophila SB210]
XP_005646351.1	496	COCSUDRAFT_29803	eukaryota>viridiplantae>chlorophyta	Coccomyxa	subellipsoidea C-169	Pkinase-domain-containing protein [Coccomyxa subellipsoidea C-169]
NP_001034228.1	495	Camkg	eukaryota>metazoa>chordata>vertebrata	Mus	musculus	calcium/calmodulin-dependent protein kinase type II subunit gamma isoform 3 [Mus musculus]
NP_001213.2	495	CAMK,CAMK-II,CAMKG	eukaryota>metazoa>chordata>vertebrata	Homo	sapiens	calcium/calmodulin-dependent protein kinase type II subunit gamma isoform 4 [Homo sapiens]
XP_003978131.2	495	LOC101071995	eukaryota>metazoa>chordata>vertebrata>actinopterygii	Takifugu	rubripes	PREDICTED: calcium/calmodulin-dependent protein kinase type II delta chain-like [Takifugu rubripes]
XP_005565537.1	495	CAMK2G	eukaryota>metazoa>chordata>vertebrata	Macaca	fascicularis	PREDICTED: calcium/calmodulin-dependent protein kinase type II subunit gamma isoform X18 [Macaca fascicularis]
XP_006560593.1	495	Camkii	eukaryota>metazoa>hexapoda	Apis	mellifera	PREDICTED: calcium/calmodulin-dependent protein kinase II isoform X30 [Apis mellifera]
XP_015833952.1	495	LOC655266	eukaryota>metazoa>hexapoda	Tribolium	castaneum	PREDICTED: calcium/calmodulin-dependent protein kinase type II alpha chain isoform X15 [Tribolium castaneum]
NP_174807.1	495	ATCDPK2,ATCPK11,calcium-dependent,protein,kinase,2,CALCIUM-DEPENDENT,PROTEIN,KINASE,2,CPK11,F15O4.8	eukaryota>viridiplantae	Arabidopsis	thaliana	calcium-dependent protein kinase 2 [Arabidopsis thaliana]
XP_001699889.1	495	CHLREDRAFT_126628	eukaryota>viridiplantae>chlorophyta	Chlamydomonas	reinhardtii	predicted protein, partial [Chlamydomonas reinhardtii]
EJY79908.1	495	OXYTRI_22813	eukaryota>alveolata>ciliophora	Oxytricha	trifallax	Protein kinase domain containing protein (macronuclear) [Oxytricha trifallax]
OMJ68897.1	495	SteCoe_33514	eukaryota>alveolata>ciliophora	Stentor	coeruleus	hypothetical protein SteCoe_33514 [Stentor coeruleus]
OMJ88179.1	495	SteCoe_9912	eukaryota>alveolata>ciliophora	Stentor	coeruleus	hypothetical protein SteCoe_9912 [Stentor coeruleus]
XP_002777779.1	495	Pmar_PMAR008714	eukaryota>alveolata	Perkinsus	marinus ATCC 50983	Calcium-dependent protein kinase, putative [Perkinsus marinus ATCC 50983]
XP_002779826.1	495	Pmar_PMAR009835	eukaryota>alveolata	Perkinsus	marinus ATCC 50983	Calcium-dependent protein kinase, putative [Perkinsus marinus ATCC 50983]
XP_002781466.1	495	Pmar_PMAR029698	eukaryota>alveolata	Perkinsus	marinus ATCC 50983	Calcium-dependent protein kinase, putative [Perkinsus marinus ATCC 50983]
OMJ85303.1	495	SteCoe_13397	eukaryota>alveolata>ciliophora	Stentor	coeruleus	hypothetical protein SteCoe_13397 [Stentor coeruleus]
OMJ90109.1	495	SteCoe_7597	eukaryota>alveolata>ciliophora	Stentor	coeruleus	hypothetical protein SteCoe_7597 [Stentor coeruleus]
OMJ74649.1	495	SteCoe_26394	eukaryota>alveolata>ciliophora	Stentor	coeruleus	hypothetical protein SteCoe_26394 [Stentor coeruleus]
OMJ79795.1	495	SteCoe_20095	eukaryota>alveolata>ciliophora	Stentor	coeruleus	hypothetical protein SteCoe_20095 [Stentor coeruleus]
OMJ85801.1	495	SteCoe_12794	eukaryota>alveolata>ciliophora	Stentor	coeruleus	hypothetical protein SteCoe_12794 [Stentor coeruleus]
OMJ68694.1	495	SteCoe_33780	eukaryota>alveolata>ciliophora	Stentor	coeruleus	hypothetical protein SteCoe_33780 [Stentor coeruleus]
XP_002615124.1	495	CLUG_05139	eukaryota>fungi>ascomycota	Clavispora	lusitaniae ATCC 42720	hypothetical protein CLUG_05139 [Clavispora lusitaniae ATCC 42720]
XP_009037159.1	495	AURANDRAFT_26376	eukaryota>stramenopiles	Aureococcus	anophagefferens	hypothetical protein AURANDRAFT_26376 [Aureococcus anophagefferens]
XP_005249921.1	494	CAM2,CAMK2,CAMKB	eukaryota>metazoa>chordata>vertebrata	Homo	sapiens	PREDICTED: calcium/calmodulin-dependent protein kinase type II subunit beta isoform X20 [Homo sapiens]
XP_011142947.1	494	LOC105185279	eukaryota>metazoa>hexapoda	Harpegnathos	saltator	PREDICTED: calcium/calmodulin-dependent protein kinase type II alpha chain isoform X13 [Harpegnathos saltator]
XP_015131771.1	494	CAMK2D	eukaryota>metazoa>chordata>vertebrata	Gallus	gallus	PREDICTED: calcium/calmodulin-dependent protein kinase type II delta chain isoform X7 [Gallus gallus]
XP_015143807.1	494	CAMK2G	eukaryota>metazoa>chordata>vertebrata	Gallus	gallus	PREDICTED: calcium/calmodulin-dependent protein kinase type II subunit gamma isoform X16 [Gallus gallus]
XP_016864164.1	494	CAMKD	eukaryota>metazoa>chordata>vertebrata	Homo	sapiens	PREDICTED: calcium/calmodulin-dependent protein kinase type II subunit delta isoform X9 [Homo sapiens]
XP_016864165.1	494	CAMKD	eukaryota>metazoa>chordata>vertebrata	Homo	sapiens	PREDICTED: calcium/calmodulin-dependent protein kinase type II subunit delta isoform X9 [Homo sapiens]
XP_018080949.1	494	LOC108696257	eukaryota>metazoa>chordata>vertebrata	Xenopus	laevis	PREDICTED: calcium/calmodulin-dependent protein kinase type II subunit gamma-like isoform X19 [Xenopus laevis]
XP_018082324.1	494	camk2,camk2g	eukaryota>metazoa>chordata>vertebrata	Xenopus	laevis	PREDICTED: calcium/calmodulin-dependent protein kinase (CaM kinase) II gamma S homeolog isoform X21 [Xenopus laevis]
XP_001781693.1	494	cpk7	eukaryota>viridiplantae	Physcomitrella	patens	cpk7 calcium-dependent protein kinase [Physcomitrella patens]
OMJ79435.1	494	SteCoe_20536	eukaryota>alveolata>ciliophora	Stentor	coeruleus	hypothetical protein SteCoe_20536 [Stentor coeruleus]
OMJ67272.1	494	SteCoe_35610	eukaryota>alveolata>ciliophora	Stentor	coeruleus	hypothetical protein SteCoe_35610 [Stentor coeruleus]
OMJ73324.1	494	SteCoe_28008	eukaryota>alveolata>ciliophora	Stentor	coeruleus	hypothetical protein SteCoe_28008 [Stentor coeruleus]
EJY85924.1	494	OXYTRI_16088	eukaryota>alveolata>ciliophora	Oxytricha	trifallax	Protein kinase domain containing protein (macronuclear) [Oxytricha trifallax]
CBJ27498.1	494	CAMK	eukaryota>stramenopiles	Ectocarpus	siliculosus	Ca2+/calmodulin-dependent protein kinase, EF-Hand protein superfamily [Ectocarpus siliculosus]
XP_005768816.1	494	EMIHUDRAFT_451551	eukaryota>haptophyceae	Emiliania	huxleyi CCMP1516	hypothetical protein EMIHUDRAFT_451551 [Emiliania huxleyi CCMP1516]
Bnat1000019719	494	Bnat1000019719	eukaryota>rhizaria	Bigelowiella	natans	>Bnat1000019719
NP_001002542.1	493	camk2d,camk2da,wu:fq17a12,zgc:92792	eukaryota>metazoa>chordata>vertebrata>actinopterygii	Danio	rerio	calcium/calmodulin-dependent protein kinase type II delta 2 chain [Danio rerio]
XP_005156428.1	493	camk2ga,zgc:109908	eukaryota>metazoa>chordata>vertebrata>actinopterygii	Danio	rerio	calcium/calmodulin-dependent protein kinase type II subunit gamma isoform X15 [Danio rerio]
XP_009174101.1	493	T265_09682	eukaryota>metazoa	Opisthorchis	viverrini	hypothetical protein T265_09682 [Opisthorchis viverrini]
XP_015131772.1	493	CAMK2D	eukaryota>metazoa>chordata>vertebrata	Gallus	gallus	PREDICTED: calcium/calmodulin-dependent protein kinase type II delta chain isoform X8 [Gallus gallus]
XP_015131773.1	493	CAMK2D	eukaryota>metazoa>chordata>vertebrata	Gallus	gallus	PREDICTED: calcium/calmodulin-dependent protein kinase type II delta chain isoform X9 [Gallus gallus]
XP_016868154.1	493	CAM2,CAMK2,CAMKB	eukaryota>metazoa>chordata>vertebrata	Homo	sapiens	PREDICTED: calcium/calmodulin-dependent protein kinase type II subunit beta isoform X21 [Homo sapiens]
XP_017327127.1	493	LOC108267517	eukaryota>metazoa>chordata>vertebrata>actinopterygii	Ictalurus	punctatus	PREDICTED: calcium/calmodulin-dependent protein kinase type II delta 1 chain isoform X5 [Ictalurus punctatus]
XP_017327128.1	493	LOC108267517	eukaryota>metazoa>chordata>vertebrata>actinopterygii	Ictalurus	punctatus	PREDICTED: calcium/calmodulin-dependent protein kinase type II delta 1 chain isoform X6 [Ictalurus punctatus]
XP_017327132.1	493	LOC108267517	eukaryota>metazoa>chordata>vertebrata>actinopterygii	Ictalurus	punctatus	PREDICTED: calcium/calmodulin-dependent protein kinase type II delta 1 chain isoform X10 [Ictalurus punctatus]
XP_017338908.1	493	LOC108273829	eukaryota>metazoa>chordata>vertebrata>actinopterygii	Ictalurus	punctatus	PREDICTED: calcium/calmodulin-dependent protein kinase type II subunit gamma isoform X13 [Ictalurus punctatus]
XP_001029735.2	493	TTHERM_01333220	eukaryota>alveolata>ciliophora	Tetrahymena	thermophila SB210	calmodulin-domain kinase [Tetrahymena thermophila SB210]
XP_001442992.1	493	GSPATT00011227001	eukaryota>alveolata>ciliophora	Paramecium	tetraurelia strain d4-2	hypothetical protein (macronuclear) [Paramecium tetraurelia strain d4-2]
EJY88048.1	493	OXYTRI_21209	eukaryota>alveolata>ciliophora	Oxytricha	trifallax	Protein kinase domain containing protein (macronuclear) [Oxytricha trifallax]
OMJ89974.1	493	SteCoe_7762	eukaryota>alveolata>ciliophora	Stentor	coeruleus	hypothetical protein SteCoe_7762 [Stentor coeruleus]
OMJ86407.1	493	SteCoe_12063	eukaryota>alveolata>ciliophora	Stentor	coeruleus	hypothetical protein SteCoe_12063 [Stentor coeruleus]
[illegible]	[illegible]	[illegible]	[illegible]	[illegible]	[illegible]	[illegible]

XP_013780769.1	416	LOC106465117	eukaryota>metazoa	Limulus	polyphemus	serine/threonine-protein kinase H1 homolog [Limulus polyphemus]
CEM36746.1	416	Vbra_3415	eukaryota>alveolata	Vitrella	brassicaformis CCMP3155	unnamed protein product [Vitrella brassicaformis CCMP3155]
NP_001167827.1	415	GRMZM2G422576	eukaryota>viridiplantae	Zea	mays	putative protein kinase superfamily protein [Zea mays]
XP_006579812.1	415	LOC100803313	eukaryota>viridiplantae	Glycine	max	PREDICTED: CDPK-related kinase 5-like isoform X2 [Glycine max]
CAG08692.1	414	GSTEN:00029739:G:001	eukaryota>metazoa>chordata>vertebrata>actinopterygii	Tetraodon	nigroviridis	unnamed protein product [Tetraodon nigroviridis]
XP_001539950.1	414	HCAG_05417	eukaryota>fungi>ascomycota	Histoplasma	capsulatum NAm1	calcium/calmodulin-dependent protein kinase type I [Histoplasma capsulatum NAm1]
XP_015155457.1	414	CAMK4L	eukaryota>metazoa>chordata>vertebrata	Gallus	gallus	PREDICTED: calcium/calmodulin-dependent protein kinase type IV-like isoform X1 [Gallus gallus]
XP_015155458.1	414	CAMK4L	eukaryota>metazoa>chordata>vertebrata	Gallus	gallus	PREDICTED: calcium/calmodulin-dependent protein kinase type IV-like isoform X1 [Gallus gallus]
CBN79381.1	414	Esi_0202_0009	eukaryota>stramenopiles	Ectocarpus	siliculosus	conserved unknown protein [Ectocarpus siliculosus]
XP_001440154.1	414	GSPATT00009149001	eukaryota>alveolata>ciliophora	Paramecium	tetraurelia strain d4-2	hypothetical protein (macronuclear) [Paramecium tetraurelia strain d4-2]
XP_012897343.1	414	GSBLH_T00003190001	eukaryota>stramenopiles	Blastocystis	hominis	uncharacterized protein [Blastocystis hominis]
XP_003967700.1	413	LOC101070748	eukaryota>metazoa>chordata>vertebrata>actinopterygii	Takifugu	rubripes	PREDICTED: calcium/calmodulin-dependent protein kinase type 1D-like [Takifugu rubripes]
XP_005265573.1	413	CAMKI	eukaryota>metazoa>chordata>vertebrata	Homo	sapiens	PREDICTED: calcium/calmodulin-dependent protein kinase type 1 isoform X1 [Homo sapiens]
XP_011393243.1	413	camk-1	eukaryota>fungi>ascomycota	Neurospora	crassa OR74A	Ca/CaM-dependent kinase-1, variant 1 [Neurospora crassa OR74A]
XP_011393244.1	413	camk-1	eukaryota>fungi>ascomycota	Neurospora	crassa OR74A	Ca/CaM-dependent kinase-1 [Neurospora crassa OR74A]
XP_011393245.1	413	camk-1	eukaryota>fungi>ascomycota	Neurospora	crassa OR74A	Ca/CaM-dependent kinase-1, variant 2 [Neurospora crassa OR74A]
XP_012352361.1	413	CAMK2B	eukaryota>metazoa>chordata>vertebrata	Nomascus	leucogenys	PREDICTED: calcium/calmodulin-dependent protein kinase type II subunit beta [Nomascus leucogenys]
XP_016868155.1	413	CAM2,CAMK2,CAMKB	eukaryota>metazoa>chordata>vertebrata	Homo	sapiens	PREDICTED: calcium/calmodulin-dependent protein kinase type II subunit beta isoform X22 [Homo sapiens]
XP_009300936.2	413	camk1a	eukaryota>metazoa>chordata>vertebrata>actinopterygii	Danio	rerio	PREDICTED: calcium/calmodulin-dependent protein kinase type 1-like isoform X2 [Danio rerio]
XP_005845710.1	413	CHLNCDRAFT_32147	eukaryota>viridiplantae>chlorophyta	Chlorella	variabilis	hypothetical protein CHLNCDRAFT_32147 [Chlorella variabilis]
XP_004936827.1	412	MKNK1	eukaryota>metazoa>chordata>vertebrata	Gallus	gallus	PREDICTED: MAP kinase-interacting serine/threonine-protein kinase 1 isoform X3 [Gallus gallus]
XP_012895897.1	412	GSBLH_T00001952001	eukaryota>stramenopiles	Blastocystis	hominis	uncharacterized protein [Blastocystis hominis]
XP_016516068.1	411	LOC107832719	eukaryota>viridiplantae	Nicotiana	tabacum	PREDICTED: calcium-dependent protein kinase 5-like [Nicotiana tabacum]
XP_018184414.1	410	L228DRAFT_271757	eukaryota>fungi>ascomycota	Xylona	heveae TC161	CMK1 Ca2+/calmodulin-dependent protein kinase [Xylona heveae TC161]
XP_018734691.1	410	CMK2	eukaryota>fungi>ascomycota	Sugiyamaella	lignohabitans	calmodulin-dependent protein kinase CMK2 [Sugiyamaella lignohabitans]
XP_001750784.1	410	MONBRDRAFT_39303	eukaryota>choanoflagellida	Monosiga	brevicollis MX1	hypothetical protein [Monosiga brevicollis MX1]
EFA77412.1	410	PPL_12628	eukaryota>amoebozoa>mycetozoa>dictyosteliida	Polysphondylium	pallidum PN500	putative protein serine/threonine kinase [Polysphondylium pallidum PN500]
XP_660016.1	409	AN2412.2	eukaryota>fungi>ascomycota	Aspergillus	nidulans FGSC A4	KCC1_EMENI Calcium/calmodulin-dependent protein kinase (CMPK) [Aspergillus nidulans FGSC A4]
Chet1000006811	409	Chet1000006811	eukaryota>fungi>ascomycota	Cochliobolus	heterostrophus	>Chet1000006811
XP_009843353.1	408	H257_16578	eukaryota>stramenopiles	Aphanomyces	astaci	CAMK/CAMK1 protein kinase [Aphanomyces astaci]
XP_013424048.1	408	M436DRAFT_54633	eukaryota>fungi>ascomycota	Aureobasidium	namibiae CBS 147.97	Ca2+/calmodulin-dependent protein kinase [Aureobasidium namibiae CBS 147.97]
XP_001448757.1	408	GSPATT00016202001	eukaryota>alveolata>ciliophora	Paramecium	tetraurelia strain d4-2	hypothetical protein (macronuclear) [Paramecium tetraurelia strain d4-2]
XP_006681018.1	408	BATDEDRAFT_17461	eukaryota>fungi>chytridiomycota	Batrachochytrium	dendrobatidis JAM81	hypothetical protein BATDEDRAFT_17461 [Batrachochytrium dendrobatidis JAM81]
XP_013761964.1	408	AMSG_01358	eukaryota>apusozoa>apusomonadidae	Thecamonas	trahens ATCC 50062	CAMK/CAMK1 protein kinase [Thecamonas trahens ATCC 50062]
XP_009843349.1	407	H257_16578	eukaryota>stramenopiles	Aphanomyces	astaci	CAMK/CAMK1 protein kinase, variant 1 [Aphanomyces astaci]
XP_008878422.1	407	H310_12916	eukaryota>stramenopiles	Aphanomyces	invadans	CAMK/CAMK1 protein kinase [Aphanomyces invadans]
ELR16696.1	407	ACA1_089840	eukaryota>amoebozoa>acanthamoebidae	Acanthamoeba	castellanii str. Neff	calcium/calmodulindependent protein kinase [Acanthamoeba castellanii str. Neff]
OHT10759.1	407	TRFO_19894	eukaryota>parabasalia	Tritrichomonas	foetus	CAMK family protein kinase [Tritrichomonas foetus]
XP_009843333.1	406	H257_16565	eukaryota>stramenopiles	Aphanomyces	astaci	CAMK/CAMK1 protein kinase [Aphanomyces astaci]
XP_014532050.1	406	PDIP_78930	eukaryota>fungi>ascomycota	Penicillium	digitatum Pd1	Calcium/calmodulin-dependent protein kinase, putative [Penicillium digitatum Pd1]
XP_017306206.1	406	camkv	eukaryota>metazoa>chordata>vertebrata>actinopterygii	Ictalurus	punctatus	PREDICTED: caM kinase-like vesicle-associated protein [Ictalurus punctatus]
XP_005703958.1	406	Gasu_50310	eukaryota>rhodophyta	Galdieria	sulphuraria	serine/threonine protein kinase [Galdieria sulphuraria]
NP_524622.1	405	Calcium/calmodulin-dependent,protein,kinase,CaMK1,camKI,caMKI,CamKI,CG1495,dCKI,Dmel\CG1495	eukaryota>metazoa>hexapoda	Drosophila	melanogaster	Calcium/calmodulin-dependent protein kinase I, isoform G [Drosophila melanogaster]
NP_726569.1	405	Calcium/calmodulin-dependent,protein,kinase,CaMK1,camKI,caMKI,CamKI,CG1495,dCKI,Dmel\CG1495	eukaryota>metazoa>hexapoda	Drosophila	melanogaster	Calcium/calmodulin-dependent protein kinase I, isoform A [Drosophila melanogaster]
NP_726570.1	405	Calcium/calmodulin-dependent,protein,kinase,CaMK1,camKI,caMKI,CamKI,CG1495,dCKI,Dmel\CG1495	eukaryota>metazoa>hexapoda	Drosophila	melanogaster	Calcium/calmodulin-dependent protein kinase I, isoform B [Drosophila melanogaster]
XP_008620433.1	405	SDRG_16022	eukaryota>stramenopiles	Saprolegnia	diclina VS20	CAMK/CAMK1 protein kinase [Saprolegnia diclina VS20]
XP_012198712.1	405	SPRG_04395	eukaryota>stramenopiles	Saprolegnia	parasitica CBS 223.65	CAMK/CAMK1 protein kinase [Saprolegnia parasitica CBS 223.65]
XP_009830637.1	405	H257_06948	eukaryota>stramenopiles	Aphanomyces	astaci	CAMK/CAMK1 protein kinase [Aphanomyces astaci]
XP_014531764.1	404	PDIP_76070	eukaryota>fungi>ascomycota	Penicillium	digitatum Pd1	hypothetical protein PDIP_76070 [Penicillium digitatum Pd1]
XP_660669.1	404	AN3065.2	eukaryota>fungi>ascomycota	Aspergillus	nidulans FGSC A4	hypothetical protein AN3065.2 [Aspergillus nidulans FGSC A4]
XP_019026599.1	404	SAICODRAFT_64097	eukaryota>fungi>ascomycota	Saitoella	complicata NRRL Y-17804	Pkinase-domain-containing protein [Saitoella complicata NRRL Y-17804]
XP_006500894.1	404	2810011D23Rik,8030469K03Rik,CaMK,II,[d]-CaMKII	eukaryota>metazoa>chordata>vertebrata	Mus	musculus	PREDICTED: calcium/calmodulin-dependent protein kinase type II subunit delta isoform X19 [Mus musculus]
XP_011530594.1	404	CAMKD	eukaryota>metazoa>chordata>vertebrata	Homo	sapiens	PREDICTED: calcium/calmodulin-dependent protein kinase type II subunit delta isoform X14 [Homo sapiens]
XP_015306010.1	404	CAMK2D	eukaryota>metazoa>chordata>vertebrata	Macaca	fascicularis	PREDICTED: calcium/calmodulin-dependent protein kinase type II subunit delta isoform X22 [Macaca fascicularis]
XP_008896367.1	404	PPTG_04245	eukaryota>stramenopiles	Phytophthora	parasitica INRA-310	CAMK/CAMK1 protein kinase [Phytophthora parasitica INRA-310]
XP_017177432.1	404	4921505G21Rik,AW061264	eukaryota>metazoa>chordata>vertebrata	Mus	musculus	PREDICTED: serine/threonine-protein kinase 33 isoform X5 [Mus musculus]
XP_012208773.1	403	SPRG_14268	eukaryota>stramenopiles	Saprolegnia	parasitica CBS 223.65	CAMK/CAMK1 protein kinase [Saprolegnia parasitica CBS 223.65]
XP_016513616.1	402	LOC107830535	eukaryota>viridiplantae	Nicotiana	tabacum	PREDICTED: calcium-dependent protein kinase 20-like [Nicotiana tabacum]
[illegible]	[illegible]	[illegible]	[illegible]	[illegible]	[illegible]	[illegible]

[illegible]

XP_009830617.1	125	H257_06931	eukaryota>stramenopiles	Aphanomyces	astaci	hypothetical protein H257_06931 [Aphanomyces astaci]
Chet1000006047	125	Chet1000006047	eukaryota>fungi>ascomycota	Cochliobolus	heterostrophus	>Chet1000006047
XP_002289889.1	124	THAPSDRAFT_18469	eukaryota>stramenopiles	Thalassiosira	pseudonana CCMP1335	predicted protein, partial [Thalassiosira pseudonana CCMP1335]
XP_002508429.1	124	MICPUN_85736	eukaryota>viridiplantae>chlorophyta	Micromonas	commoda	predicted protein [Micromonas commoda]
XP_005763244.1	124	EMIHUDRAFT_78676	eukaryota>haptophyceae	Emiliania	huxleyi CCMP1516	FKBP-type peptidyl-prolyl cis-trans isomerase, partial [Emiliania huxleyi CCMP1516]
XP_009037672.1	124	AURANDRAFT_16370	eukaryota>stramenopiles	Aureococcus	anophagefferens	hypothetical protein AURANDRAFT_16370, partial [Aureococcus anophagefferens]
XP_013759174.1	124	AMSG_03924	eukaryota>apusozoa>apusomonadidae	Thecamonas	trahens ATCC 50062	peptidyl-prolyl cis-trans isomerase [Thecamonas trahens ATCC 50062]
XP_018191044.1	124	L228DRAFT_265958	eukaryota>fungi>ascomycota	Xylona	heveae TC161	peptidyl-prolyl cis-trans isomerase [Xylona heveae TC161]
XP_710751.1	124	RBP1	eukaryota>fungi>ascomycota	Candida	albicans SC5314	peptidylprolyl isomerase [Candida albicans SC5314]
Bnat1000011072	124	Bnat1000011072	eukaryota>rhizaria	Bigelowiella	natans	>Bnat1000011072
Pram1000007139	124	Pram1000007139	eukaryota>stramenopiles	Phytophthora	ramorum	>Pram1000007139
ABP00115.1	123	FBP12	eukaryota>viridiplantae>chlorophyta	Ostreococcus	lucimarinus CCE9901	peptidyl-prolyl cis-trans isomerase, FKBP-type [Ostreococcus lucimarinus CCE9901]
XP_003055776.1	123	MICPUCDRAFT_8507	eukaryota>viridiplantae>chlorophyta	Micromonas	pusilla CCMP1545	predicted protein, partial [Micromonas pusilla CCMP1545]
Bnat1000014841	123	Bnat1000014841	eukaryota>rhizaria	Bigelowiella	natans	>Bnat1000014841
KPI88222.1	122	ABL78_2726	eukaryota>euglenozoa>kinetoplastida	Leptomonas	seymouri	putative peptidyl-prolyl cis-trans isomerase [Leptomonas seymouri]
XP_002508612.1	122	MICPUN_62181	eukaryota>viridiplantae>chlorophyta	Micromonas	commoda	predicted protein [Micromonas commoda]
XP_008908615.1	122	PPTG_13900	eukaryota>stramenopiles	Phytophthora	parasitica INRA-310	hypothetical protein PPTG_13900 [Phytophthora parasitica INRA-310]
XP_011127622.1	122	AOL_s00215g118	eukaryota>fungi>ascomycota	Arthrobotrys	oligospora ATCC 24927	hypothetical protein AOL_s00215g118 [Arthrobotrys oligospora ATCC 24927]
XP_014537689.1	122	PDIP_15200	eukaryota>fungi>ascomycota	Penicillium	digitatum Pd1	FKBP-type peptidyl-prolyl isomerase, putative [Penicillium digitatum Pd1]
Bnat1000005560	122	Bnat1000005560	eukaryota>rhizaria	Bigelowiella	natans	>Bnat1000005560
XP_005771612.1	121	EMIHUDRAFT_46588	eukaryota>haptophyceae	Emiliania	huxleyi CCMP1516	FKBP-type peptidyl-prolyl cis-trans isomerase, partial [Emiliania huxleyi CCMP1516]
XP_005833559.1	121	GUITHDRAFT_175442	eukaryota>cryptophyta	Guillardia	theta CCMP2712	FKBP-type peptidyl-prolyl cis-trans isomerase [Guillardia theta CCMP2712]
XP_009039102.1	121	AURANDRAFT_6781	eukaryota>stramenopiles	Aureococcus	anophagefferens	hypothetical protein AURANDRAFT_6781, partial [Aureococcus anophagefferens]
XP_014536553.1	121	PDIP_30920	eukaryota>fungi>ascomycota	Penicillium	digitatum Pd1	FK506-binding protein 1B [Penicillium digitatum Pd1]
ORX94734.1	120	K493DRAFT_324755	eukaryota>fungi	Basidiobolus	meristosporus CBS 931.73	peptidyl-prolyl cis-trans isomerase [Basidiobolus meristosporus CBS 931.73]
CEO95487.1	120	PBRA_004213	eukaryota>rhizaria>cercozoa>plasmodiophorida	Plasmodiophora	brassicae	hypothetical protein PBRA_004213 [Plasmodiophora brassicae]
EST42124.1	120	SS50377_18432	eukaryota>diplomonadida	Spironucleus	salmonicida	FKBP-type peptidyl-prolyl cis-trans isomerase [Spironucleus salmonicida]
XP_003383441.1	120	LOC100638671	eukaryota>metazoa	Amphimedon	queenslandica	PREDICTED: peptidyl-prolyl cis-trans isomerase FKBP12-like [Amphimedon queenslandica]
XP_005781001.1	120	EMIHUDRAFT_58004	eukaryota>haptophyceae	Emiliania	huxleyi CCMP1516	FKBP-type peptidyl-prolyl cis-trans isomerase [Emiliania huxleyi CCMP1516]
XP_006675582.1	120	BATDEDRAFT_22495	eukaryota>fungi>chytridiomycota	Batrachochytrium	dendrobatidis JAM81	hypothetical protein BATDEDRAFT_22495 [Batrachochytrium dendrobatidis JAM81]
XP_014528683.1	120	JH06_1212	eukaryota>stramenopiles	Blastocystis	sp. subtype 4	FKPB-type peptidyl-prolyl cis-trans isomerase [Blastocystis sp. subtype 4]
OLQ01587.1	119	FKBP12	eukaryota>alveolata	Symbiodinium	microadriaticum	Peptidyl-prolyl cis-trans isomerase FKBP12 [Symbiodinium microadriaticum]
XP_013428097.1	119	M436DRAFT_63031	eukaryota>fungi>ascomycota	Aureobasidium	namibiae CBS 147.97	FKBP peptidyl-prolyl cis-trans isomerase [Aureobasidium namibiae CBS 147.97]
XP_013772828.1	119	LOC106457927	eukaryota>metazoa	Limulus	polyphemus	peptidyl-prolyl cis-trans isomerase FKBP12-like [Limulus polyphemus]
XP_014156216.1	119	SARC_05406	eukaryota>ichthyosporea	Sphaeroforma	arctica JP610	peptidyl-prolyl isomerase FKBP12 [Sphaeroforma arctica JP610]
CEM25102.1	118	Vbra_3277	eukaryota>alveolata	Vitrella	brassicaformis CCMP3155	unnamed protein product [Vitrella brassicaformis CCMP3155]
XP_002109901.1	118	TRIADDRAFT_21313	eukaryota>metazoa>placozoa	Trichoplax	adhaerens	hypothetical protein TRIADDRAFT_21313 [Trichoplax adhaerens]
XP_002287222.1	118	THAPSDRAFT_32093	eukaryota>stramenopiles	Thalassiosira	pseudonana CCMP1335	predicted protein, partial [Thalassiosira pseudonana CCMP1335]
XP_002898522.1	118	PITG_14620	eukaryota>stramenopiles	Phytophthora	infestans T30-4	FKBP-type peptidyl-prolyl cis-trans isomerase, putative [Phytophthora infestans T30-4]
XP_005767562.1	118	EMIHUDRAFT_445648	eukaryota>haptophyceae	Emiliania	huxleyi CCMP1516	hypothetical protein EMIHUDRAFT_445648 [Emiliania huxleyi CCMP1516]
XP_009034527.1	118	AURANDRAFT_22069	eukaryota>stramenopiles	Aureococcus	anophagefferens	hypothetical protein AURANDRAFT_22069 [Aureococcus anophagefferens]
XP_009042283.1	118	AURANDRAFT_34605	eukaryota>stramenopiles	Aureococcus	anophagefferens	hypothetical protein AURANDRAFT_34605 [Aureococcus anophagefferens]
XP_009530593.1	118	PHYSODRAFT_513680	eukaryota>stramenopiles	Phytophthora	sojae	hypothetical protein PHYSODRAFT_513680 [Phytophthora sojae]
Smed1000010218	118	Smed1000010218	eukaryota>metazoa>platyhelminthe	Schmidtea	mediterranea	>Smed1000010218
EPB90228.1	117	HMPREF1544_02913	eukaryota>fungi	Mucor	circinelloides f. circinelloides 1006PhL	FK506-binding protein 1A [Mucor circinelloides f. circinelloides 1006PhL]
[illegible]						

XP_008643437.1	543	GRMZM2G080083	eukaryota>viridiplantae	Zea	mays	serine/threonine protein phosphatase 2A regulatory subunit B''alpha isoform X1 [Zea mays]
XP_008643438.1	543	GRMZM2G080083	eukaryota>viridiplantae	Zea	mays	serine/threonine protein phosphatase 2A regulatory subunit B''alpha isoform X1 [Zea mays]
XP_001438471.1	541	GSPATT00000786001	eukaryota>alveolata>ciliophora	Paramecium	tetraurelia strain d4-2	hypothetical protein (macronuclear) [Paramecium tetraurelia strain d4-2]
XP_002966764.1	541	SELMODRAFT_85336	eukaryota>viridiplantae	Selaginella	moellendorffii	hypothetical protein SELMODRAFT_85336 [Selaginella moellendorffii]
XP_003536224.1	541	LOC100793000	eukaryota>viridiplantae	Glycine	max	PREDICTED: serine/threonine protein phosphatase 2A regulatory subunit B''beta-like [Glycine max]
EST46160.1	540	SS50377_13752	eukaryota>diplomonadida	Spironucleus	salmonicida	Serine/threonine-protein phosphatase 2A [Spironucleus salmonicida]
KPI86584.1	540	ABL78_4359	eukaryota>euglenozoa>kinetoplastida	Leptomonas	seymouri	hypothetical protein ABL78_4359 [Leptomonas seymouri]
XP_006345897.1	540	LOC102590984	eukaryota>viridiplantae	Solanum	tuberosum	PREDICTED: serine/threonine protein phosphatase 2A regulatory subunit B''beta-like isoform X1 [Solanum tuberosum]
XP_008664881.1	540	LOC103643458	eukaryota>viridiplantae	Zea	mays	probable serine/threonine protein phosphatase 2A regulatory subunit B''delta isoform X1 [Zea mays]
Bnat1000013915	540	Bnat1000013915	eukaryota>rhizaria	Bigelowiella	natans	>Bnat1000013915 Bnat1000013915
EPY20970.1	539	STCU_08752	eukaryota>euglenozoa>kinetoplastida	Strigomonas	culicis	protein phosphatase 2 (formerly 2A), regulatory subunit B'' [Strigomonas culicis]
XP_002272394.1	539	LOC100246385	eukaryota>viridiplantae	Vitis	vinifera	PREDICTED: serine/threonine protein phosphatase 2A regulatory subunit B''beta isoform X3 [Vitis vinifera]
XP_006345898.1	539	LOC102590984	eukaryota>viridiplantae	Solanum	tuberosum	PREDICTED: serine/threonine protein phosphatase 2A regulatory subunit B''beta-like isoform X2 [Solanum tuberosum]
XP_006361409.1	539	LOC102584799	eukaryota>viridiplantae	Solanum	tuberosum	PREDICTED: serine/threonine protein phosphatase 2A regulatory subunit B''beta-like [Solanum tuberosum]
XP_010660207.1	539	LOC100250118	eukaryota>viridiplantae	Vitis	vinifera	PREDICTED: serine/threonine protein phosphatase 2A regulatory subunit B''beta [Vitis vinifera]
XP_016467583.1	539	LOC107790195	eukaryota>viridiplantae	Nicotiana	tabacum	PREDICTED: serine/threonine protein phosphatase 2A regulatory subunit B''beta-like [Nicotiana tabacum]
XP_016490243.1	539	LOC107810035	eukaryota>viridiplantae	Nicotiana	tabacum	PREDICTED: serine/threonine protein phosphatase 2A regulatory subunit B''beta-like [Nicotiana tabacum]
XP_016490244.1	539	LOC107810035	eukaryota>viridiplantae	Nicotiana	tabacum	PREDICTED: serine/threonine protein phosphatase 2A regulatory subunit B''beta-like [Nicotiana tabacum]
XP_016490245.1	539	LOC107810035	eukaryota>viridiplantae	Nicotiana	tabacum	PREDICTED: serine/threonine protein phosphatase 2A regulatory subunit B''beta-like [Nicotiana tabacum]
XP_016514670.1	539	LOC107831426	eukaryota>viridiplantae	Nicotiana	tabacum	PREDICTED: serine/threonine protein phosphatase 2A regulatory subunit B''beta-like [Nicotiana tabacum]
XP_016514671.1	539	LOC107831426	eukaryota>viridiplantae	Nicotiana	tabacum	PREDICTED: serine/threonine protein phosphatase 2A regulatory subunit B''beta-like [Nicotiana tabacum]
XP_016514672.1	539	LOC107831426	eukaryota>viridiplantae	Nicotiana	tabacum	PREDICTED: serine/threonine protein phosphatase 2A regulatory subunit B''beta-like [Nicotiana tabacum]
XP_018284469.1	539	PHYBLDRAFT_70884	eukaryota>fungi	Phycomyces	blakesleeanus NRRL 1555(-)	hypothetical protein PHYBLDRAFT_70884 [Phycomyces blakesleeanus NRRL 1555(-)]
CCW69367.1	538	GSHART1_T00006588001	eukaryota>euglenozoa>kinetoplastida	Phytomonas	sp. isolate Hart1	unnamed protein product [Phytomonas sp. isolate Hart1]
NP_199222.1	538	AT5G44090	eukaryota>viridiplantae	Arabidopsis	thaliana	Calcium-binding EF-hand family protein [Arabidopsis thaliana]
XP_015774277.1	538	LOC107352461	eukaryota>metazoa>cnidaria	Acropora	digitifera	PREDICTED: serine/threonine-protein phosphatase 2A regulatory subunit B'' subunit beta-like, partial [Acropora digitifera]
XP_003521774.1	537	LOC100807531	eukaryota>viridiplantae	Glycine	max	PREDICTED: probable serine/threonine protein phosphatase 2A regulatory subunit B''delta [Glycine max]
XP_003554724.1	537	LOC100815724	eukaryota>viridiplantae	Glycine	max	PREDICTED: probable serine/threonine protein phosphatase 2A regulatory subunit B''delta [Glycine max]
XP_003556454.1	537	LOC100799748	eukaryota>viridiplantae	Glycine	max	PREDICTED: probable serine/threonine protein phosphatase 2A regulatory subunit B''delta isoform X1 [Glycine max]
XP_006589210.1	537	LOC100792998	eukaryota>viridiplantae	Glycine	max	PREDICTED: probable serine/threonine protein phosphatase 2A regulatory subunit B''delta [Glycine max]
XP_006589211.1	537	LOC100792998	eukaryota>viridiplantae	Glycine	max	PREDICTED: probable serine/threonine protein phosphatase 2A regulatory subunit B''delta [Glycine max]
XP_006589212.1	537	LOC100792998	eukaryota>viridiplantae	Glycine	max	PREDICTED: probable serine/threonine protein phosphatase 2A regulatory subunit B''delta [Glycine max]
XP_006606445.1	537	LOC100799748	eukaryota>viridiplantae	Glycine	max	PREDICTED: probable serine/threonine protein phosphatase 2A regulatory subunit B''delta isoform X1 [Glycine max]
XP_006606446.1	537	LOC100799748	eukaryota>viridiplantae	Glycine	max	PREDICTED: probable serine/threonine protein phosphatase 2A regulatory subunit B''delta isoform X1 [Glycine max]
XP_014629523.1	537	LOC100807531	eukaryota>viridiplantae	Glycine	max	PREDICTED: probable serine/threonine protein phosphatase 2A regulatory subunit B''delta [Glycine max]
EPY19001.1	536	STCU_09675	eukaryota>euglenozoa>kinetoplastida	Strigomonas	culicis	hypothetical protein STCU_09675 [Strigomonas culicis]
EPY33753.1	536	STCU_02013	eukaryota>euglenozoa>kinetoplastida	Strigomonas	culicis	hypothetical protein STCU_02013 [Strigomonas culicis]
NP_198242.1	536	F7P1.80,F7P1_80	eukaryota>viridiplantae	Arabidopsis	thaliana	Calcium-binding EF-hand family protein [Arabidopsis thaliana]
NP_568509.1	536	F7P1.30,F7P1_30	eukaryota>viridiplantae	Arabidopsis	thaliana	Calcium-binding EF-hand family protein [Arabidopsis thaliana]
XP_001568114.1	536	LBRM_33_3500	eukaryota>euglenozoa>kinetoplastida	Leishmania	braziliensis MHOM/BR/75/M2904	conserved hypothetical protein [Leishmania braziliensis MHOM/BR/75/M2904]
XP_008917473.1	536	PPTG_20421	eukaryota>stramenopiles	Phytophthora	parasitica INRA-310	hypothetical protein PPTG_20421, partial [Phytophthora parasitica INRA-310]
XP_010702423.1	536	LPMP_333350	eukaryota>euglenozoa>kinetoplastida	Leishmania	panamensis	hypothetical protein LPMP_333350 [Leishmania panamensis]
EPY25465.1	535	AGDE_11742	eukaryota>euglenozoa>kinetoplastida	Angomonas	deanei	hypothetical protein AGDE_11742 [Angomonas deanei]
NP_175847.1	535	F20D21.27,F20D21_27	eukaryota>viridiplantae	Arabidopsis	thaliana	Calcium-binding EF-hand family protein [Arabidopsis thaliana]
XP_001468382.1	534	LINJ_33_3370	eukaryota>euglenozoa>kinetoplastida	Leishmania	infantum JPCM5	conserved hypothetical protein [Leishmania infantum JPCM5]
XP_001686084.1	534	LMJF_33_3220	eukaryota>euglenozoa>kinetoplastida	Leishmania	major strain Friedlin	conserved hypothetical protein [Leishmania major strain Friedlin]
XP_003864160.1	534	LDBPK_333370	eukaryota>euglenozoa>kinetoplastida	Leishmania	donovani	hypothetical protein, conserved [Leishmania donovani]
XP_003878578.1	534	LMXM_32_3220	eukaryota>euglenozoa>kinetoplastida	Leishmania	mexicana MHOM/GT/2001/U1103	conserved hypothetical protein [Leishmania mexicana MHOM/GT/2001/U1103]
ERG81877.1	533	ASU_09497	eukaryota>metazoa>nematoda	Ascaris	suum	serine threonine-protein phosphatase 2a regulatory subunit b subunit alpha [Ascaris suum]
XP_017324900.1	533	LOC108266308	eukaryota>metazoa>chordata>vertebrata>actinopterygii	Ictalurus	punctatus	PREDICTED: serine/threonine-protein phosphatase 2A regulatory subunit B'' subunit beta-like isoform X2 [Ictalurus punctatus]
ABO97244.1	531	OSTLU_32696	eukaryota>viridiplantae>chlorophyta	Ostreococcus	lucimarinus CCE9901	predicted protein [Ostreococcus lucimarinus CCE9901]
NP_742156.2	530	3222402P14Rik,A730042E07	eukaryota>metazoa>chordata>vertebrata	Mus	musculus	serine/threonine-protein phosphatase 2A regulatory subunit B'' subunit alpha isoform 2 [Mus musculus]
NP_171892.2	529	F21M11.11,F21M11_11	eukaryota>viridiplantae	Arabidopsis	thaliana	Calcium-binding EF hand family protein [Arabidopsis thaliana]
NP_871626.1	529	PPP2R3,PR130,PR72	eukaryota>metazoa>chordata>vertebrata	Homo	sapiens	serine/threonine-protein phosphatase 2A regulatory subunit B'' subunit alpha isoform 2 [Homo sapiens]
OMJ71500.1	529	SteCoe_30282	eukaryota>alveolata>ciliophora	Stentor	coeruleus	hypothetical protein SteCoe_30282 [Stentor coeruleus]
OMJ93943.1	529	SteCoe_2999	eukaryota>alveolata>ciliophora	Stentor	coeruleus	hypothetical protein SteCoe_2999 [Stentor coeruleus]
XP_003265305.1	529	PPP2R3A	eukaryota>metazoa>chordata>vertebrata	Nomascus	leucogenys	PREDICTED: serine/threonine-protein phosphatase 2A regulatory subunit B'' subunit alpha isoform X2 [Nomascus leucogenys]
XP_005545890.1	529	PPP2R3A	eukaryota>metazoa>chordata>vertebrata	Macaca	fascicularis	PREDICTED: serine/threonine-protein phosphatase 2A regulatory subunit B'' subunit alpha isoform X2 [Macaca fascicularis]
XP_009312624.1	528	DQ04_05721050	eukaryota>euglenozoa>kinetoplastida	Trypanosoma	grayi	hypothetical protein DQ04_05721050 [Trypanosoma grayi]
XP_806034.1	528	Tc00.1047053506815.50	eukaryota>euglenozoa>kinetoplastida	Trypanosoma	cruzi strain CL Brener	hypothetical protein [Trypanosoma cruzi strain CL Brener]
XP_811419.1	528	Tc00.1047053507105.30	eukaryota>euglenozoa>kinetoplastida	Trypanosoma	cruzi strain CL Brener	hypothetical protein [Trypanosoma cruzi strain CL Brener]
OMJ79973.1	526	SteCoe_19892	eukaryota>alveolata>ciliophora	Stentor	coeruleus	hypothetical protein SteCoe_19892 [Stentor coeruleus]
OMJ92953.1	526	SteCoe_4171	eukaryota>alveolata>ciliophora	Stentor	coeruleus	hypothetical protein SteCoe_4171 [Stentor coeruleus]
XP_011613645.1	526	LOC101070572	eukaryota>metazoa>chordata>vertebrata>actinopterygii	Takifugu	rubripes	PREDICTED: serine/threonine-protein phosphatase 2A regulatory subunit B'' subunit alpha-like [Takifugu rubripes]
NP_001156887.1	525	PP2A,B''-PR59,PP2A,PR59,Ppp2r3,Ppp2r3a,PR59	eukaryota>metazoa>chordata>vertebrata	Mus	musculus	serine/threonine-protein phosphatase 2A regulatory subunit B'' subunit delta isoform 1 [Mus musculus]
XP_001764395.1	524	PHYPADRAFT_211123	eukaryota>viridiplantae	Physcomitrella	patens	predicted protein [Physcomitrella patens]
GAQ87516.1	522	KFL_003580090	eukaryota>viridiplantae	Klebsormidium	nitens	protein phosphatase 2 regulatory subunit [Klebsormidium nitens]
XP_001775422.1	521	PHYPADRAFT_193001	eukaryota>viridiplantae	Physcomitrella	patens	predicted protein [Physcomitrella patens]
EPZ31957.1	520	O9G_001967	eukaryota>fungi	Rozella	allomycis CSF55	EF-Hand 1, calcium-binding site domain-containing protein [Rozella allomycis CSF55]
XP_005163463.1	520	ppp2r3a	eukaryota>metazoa>chordata>vertebrata>actinopterygii	Danio	rerio	serine/threonine-protein phosphatase 2A regulatory subunit B'' subunit alpha isoform X2 [Danio rerio]
CBJ29457.1	519	PPRS	eukaryota>stramenopiles	Ectocarpus	siliculosus	calcium ion binding [Ectocarpus siliculosus]
XP_018117237.1	519	LOC398609	eukaryota>metazoa>chordata>vertebrata	Xenopus	laevis	PREDICTED: PP2A B'' subunit PR130 isoform X2 [Xenopus laevis]
ORX85691.1	517	K493DRAFT_307203	eukaryota>fungi	Basidiobolus	meristosporus CBS 931.73	EF-hand [Basidiobolus meristosporus CBS 931.73]
XP_002612377.1	517	BRAFLDRAFT_280116	eukaryota>metazoa>chordata	Branchiostoma	floridae	hypothetical protein BRAFLDRAFT_280116, partial [Branchiostoma floridae]
Pram1000013356	517	Pram1000013356	eukaryota>stramenopiles	Phytophthora	ramorum	>Pram1000013356 Pram1000013356
XP_004938510.1	514	PPP2R3B	eukaryota>metazoa>chordata>vertebrata	Gallus	gallus	serine/threonine-protein phosphatase 2A regulatory subunit B'' subunit beta isoform X2 [Gallus gallus]
XP_003080381.1	513	Ot07g02750	eukaryota>viridiplantae>chlorophyta	Ostreococcus	tauri	protein phosphatase 2A 62 kDa B'' regulatory subunit (ISS) [Ostreococcus tauri]
XP_018105318.1	513	LOC734216	eukaryota>metazoa>chordata>vertebrata	Xenopus	laevis	PREDICTED: serine/threonine-protein phosphatase 2A regulatory subunit B'' subunit beta [Xenopus laevis]
XP_002977985.1	512	SELMODRAFT_152267	eukaryota>viridiplantae	Selaginella	moellendorffii	hypothetical protein SELMODRAFT_152267 [Selaginella moellendorffii]
XP_009060742.1	512	LOTGIDRAFT_125808	eukaryota>metazoa>mollusca	Lottia	gigantea	hypothetical protein LOTGIDRAFT_125808 [Lottia gigantea]
XP_015133228.1	512	PPP2R3B	eukaryota>metazoa>chordata>vertebrata	Gallus	gallus	serine/threonine-protein phosphatase 2A regulatory subunit B'' subunit beta isoform X3 [Gallus gallus]
XP_018100380.1	509	ppp2r3b	eukaryota>metazoa>chordata>vertebrata	Xenopus	laevis	PREDICTED: protein phosphatase 2 regulatory subunit B', beta L homeolog isoform X1 [Xenopus laevis]
XP_017324901.1	508	LOC108266308	eukaryota>metazoa>chordata>vertebrata>actinopterygii	Ictalurus	punctatus	PREDICTED: serine/threonine-protein phosphatase 2A regulatory subunit B'' subunit beta-like isoform X3 [Ictalurus punctatus]
XP_003057811.1	506	MICPUCDRAFT_26419	eukaryota>viridiplantae>chlorophyta	Micromonas	pusilla CCMP1545	predicted protein [Micromonas pusilla CCMP1545]
CBY07883.1	505	GSOID_T00003241001	eukaryota>metazoa>chordata	Oikopleura	dioica	unnamed protein product [Oikopleura dioica]
CEO94397.1	503	PBRA_000182	eukaryota>rhizaria>cercozoa>plasmodiophorida	Plasmodiophora	brassicae	hypothetical protein PBRA_000182 [Plasmodiophora brassicae]
Caps1000021892	502	Caps1000021892	eukaryota>metazoa>annelida	Capitella	spI	>Caps1000021892 Caps1000021892
EPB90326.1	500	HMPREF1544_02851	eukaryota>fungi	Mucor	circinelloides f. circinelloides 1006PhL	hypothetical protein HMPREF1544_02851 [Mucor circinelloides f. circinelloides 1006PhL]
EPY36640.1	500	STCU_00480	eukaryota>euglenozoa>kinetoplastida	Strigomonas	culicis	protein phosphatase 2 (formerly 2A), regulatory subunit B'' [Strigomonas culicis]
NP_001320292.1	500	F21M11.11,F21M11_11	eukaryota>viridiplantae	Arabidopsis	thaliana	Calcium-binding EF hand family protein [Arabidopsis thaliana]
XP_013794632.1	496	LOC106478622	eukaryota>metazoa	Limulus	polyphemus	serine/threonine-protein phosphatase 2A regulatory subunit B'' subunit beta-like [Limulus polyphemus]
XP_627769.1	496	cgd6_4380	eukaryota>alveolata>apicomplexa	Cryptosporidium	parvum Iowa II	phosphatase subunit gene g4-1; of possible animal origin; EF-hand, calcium binding domains [Cryptosporidium parvum Iowa II]
XP_018117238.1	494	LOC398609	eukaryota>metazoa>chordata>vertebrata	Xenopus	laevis	PREDICTED: PP2A B'' subunit PR130 isoform X3 [Xenopus laevis]
XP_011772439.1	492	TbgDal_III4910	eukaryota>euglenozoa>kinetoplastida	Trypanosoma	brucei gambiense DAL972	hypothetical protein, conserved [Trypanosoma brucei gambiense DAL972]
NP_035284.2	491	PP2A,B''-PR59,PP2A,PR59,Ppp2r3,Ppp2r3a,PR59	eukaryota>metazoa>chordata>vertebrata	Mus	musculus	serine/threonine-protein phosphatase 2A regulatory subunit B'' subunit delta isoform 2 [Mus musculus]
XP_004039012.1	491	IMG5_037820	eukaryota>alveolata>ciliophora	Ichthyophthirius	multifiliis	hypothetical protein IMG5_037820 [Ichthyophthirius multifiliis]
XP_006824861.1	491	LOC100377701	eukaryota>metazoa>hemichordata	Saccoglossus	kowalevskii	PREDICTED: serine/threonine-protein phosphatase 2A regulatory subunit B'' subunit gamma-like [Saccoglossus kowalevskii]
XP_814521.1	490	Tc00.1047053507257.100	eukaryota>euglenozoa>kinetoplastida	Trypanosoma	cruzi strain CL Brener	hypothetical protein [Trypanosoma cruzi strain CL Brener]
XP_821208.1	490	Tc00.1047053510329.310	eukaryota>euglenozoa>kinetoplastida	Trypanosoma	cruzi strain CL Brener	hypothetical protein [Trypanosoma cruzi strain CL Brener]
XP_009310462.1	489	DQ04_02751070	eukaryota>euglenozoa>kinetoplastida	Trypanosoma	grayi	protein phosphatase 2 (formerly 2A), regulatory subunit B [Trypanosoma grayi]
XP_001625769.1	488	NEMVEDRAFT_v1g235854	eukaryota>metazoa>cnidaria	Nematostella	vectensis	predicted protein [Nematostella vectensis]
XP_006511767.1	487	3222402P14Rik,A730042E07	eukaryota>metazoa>chordata>vertebrata	Mus	musculus	PREDICTED: serine/threonine-protein phosphatase 2A regulatory subunit B'' subunit alpha isoform X4 [Mus musculus]
XP_008662973.1	485	LOC103641393	eukaryota>viridiplantae	Zea	mays	probable serine/threonine-protein phosphatase 2A regulatory subunit B'' subunit TON2 [Zea mays]
NP_001148688.1	484	LOC100282304	eukaryota>viridiplantae	Zea	mays	phosphatase subunit g4-1 [Zea mays]
XP_011400271.1	483	F751_4574	eukaryota>viridiplantae>chlorophyta	Auxenochlorella	protothecoides	Serine/threonine-protein phosphatase 2A regulatory subunit B'' subunit alpha [Auxenochlorella protothecoides]
EPY21321.1	480	STCU_08593	eukaryota>euglenozoa>kinetoplastida	Strigomonas	culicis	hypothetical protein STCU_08593 [Strigomonas culicis]
NP_568364.1	480	EMB40,EMBRYO,DEFECTIVE,40,FASS,1,FASS,2,FS1,GDO,GORDO,T28N17.60,T28N17_60,TON2,TONNEAU,2	eukaryota>viridiplantae	Arabidopsis	thaliana	tonneau 2 (TON2) [Arabidopsis thaliana]
XP_001774695.1	480	PHYPADRAFT_89210	eukaryota>viridiplantae	Physcomitrella	patens	predicted protein [Physcomitrella patens]
OHT02233.1	479	TRFO_30695	eukaryota>parabasalia	Tritrichomonas	foetus	phosphoprotein phosphatase [Tritrichomonas foetus]
OHT16480.1	479	TRFO_02761	eukaryota>parabasalia	Tritrichomonas	foetus	EF hand family protein [Tritrichomonas foetus]
XP_016480891.1	479	LOC107801975	eukaryota>viridiplantae	Nicotiana	tabacum	PREDICTED: probable serine/threonine-protein phosphatase 2A regulatory subunit B'' subunit TON2 [Nicotiana tabacum]
XP_016507994.1	479	LOC107825632	eukaryota>viridiplantae	Nicotiana	tabacum	PREDICTED: probable serine/threonine-protein phosphatase 2A regulatory subunit B'' subunit TON2 [Nicotiana tabacum]
XP_004922532.1	478	LOC101745272	eukaryota>metazoa>hexapoda	Bombyx	mori	serine/threonine-protein phosphatase 2A regulatory subunit B'' subunit gamma [Bombyx mori]
XP_006342761.1	478	LOC102596844	eukaryota>viridiplantae	Solanum	tuberosum	PREDICTED: probable serine/threonine-protein phosphatase 2A regulatory subunit B'' subunit TON2 [Solanum tuberosum]
XP_014527398.1	478	JH06_2068	eukaryota>stramenopiles	Blastocystis	sp. subtype 4	serine threonine-protein phosphatase [Blastocystis sp. subtype 4]
XP_002968661.1	477	SELMODRAFT_90971	eukaryota>viridiplantae	Selaginella	moellendorffii	hypothetical protein SELMODRAFT_90971, partial [Selaginella moellendorffii]
XP_003529585.1	477	LOC100789934	eukaryota>viridiplantae	Glycine	max	PREDICTED: probable serine/threonine-protein phosphatase 2A regulatory subunit B'' subunit TON2 [Glycine max]
XP_003550185.1	477	LOC100776298	eukaryota>viridiplantae	Glycine	max	PREDICTED: probable serine/threonine-protein phosphatase 2A regulatory subunit B'' subunit TON2 [Glycine max]
XP_002275193.1	476	LOC100247694	eukaryota>viridiplantae	Vitis	vinifera	PREDICTED: probable serine/threonine-protein phosphatase 2A regulatory subunit B'' subunit TON2 [Vitis vinifera]
XP_002989910.1	475	LOC9629990	eukaryota>viridiplantae	Selaginella	moellendorffii	probable serine/threonine-protein phosphatase 2A regulatory subunit B'' subunit TON2 [Selaginella moellendorffii]
CAF91539.1	474	GSTEN:00006357:G:001	eukaryota>metazoa>chordata>vertebrata>actinopterygii	Tetraodon	nigroviridis	unnamed protein product, partial [Tetraodon nigroviridis]
XP_009015085.1	474	HELRODRAFT_191272	eukaryota>metazoa>annelida	Helobdella	robusta	hypothetical protein HELRODRAFT_191272 [Helobdella robusta]
Lhya1000001203	474	Lhya1000001203	eukaryota>fungi>mucoromycotina	Lichtheimia	hyalospora	>Lhya1000001203 Lhya1000001203
OHT14025.1	472	TRFO_15682	eukaryota>parabasalia	Tritrichomonas	foetus	phosphoprotein phosphatase [Tritrichomonas foetus]
XP_009840335.1	472	H257_14266	eukaryota>stramenopiles	Aphanomyces	astaci	hypothetical protein H257_14266 [Aphanomyces astaci]
NP_001145723.1	471	GRMZM2G080083	eukaryota>viridiplantae	Zea	mays	Serine/threonine protein phosphatase 2A regulatory subunit B''alpha [Zea mays]
XP_001584343.1	471	TVAG_070200	eukaryota>parabasalia	Trichomonas	vaginalis G3	phosphoprotein phosphatase [Trichomonas vaginalis G3]
XP_008878567.1	470	H310_13014	eukaryota>stramenopiles	Aphanomyces	invadans	hypothetical protein H310_13014 [Aphanomyces invadans]
XP_008878565.1	469	H310_13014	eukaryota>stramenopiles	Aphanomyces	invadans	hypothetical protein, variant 1 [Aphanomyces invadans]
OHT15373.1	468	TRFO_14125	eukaryota>parabasalia	Tritrichomonas	foetus	phosphoprotein phosphatase [Tritrichomonas foetus]
NP_001320289.1	467	F21M11.11,F21M11_11	eukaryota>viridiplantae	Arabidopsis	thaliana	Calcium-binding EF hand family protein [Arabidopsis thaliana]
XP_628641.1	467	cgd7_4970	eukaryota>alveolata>apicomplexa	Cryptosporidium	parvum Iowa II	protein phosphatase 2A regulatory subunit (contains a conserved version of EF hands) [Cryptosporidium parvum Iowa II]
EPY21786.1	466	AGDE_13747	eukaryota>euglenozoa>kinetoplastida	Angomonas	deanei	protein phosphatase 2 (formerly 2A), regulatory subunit B'' [Angomonas deanei]
XP_001844598.1	466	CpipJ_CPIJ003227	eukaryota>metazoa>hexapoda	Culex	quinquefasciatus	phosphatase 2 [Culex quinquefasciatus]
XP_008891485.1	465	PPTG_02214	eukaryota>stramenopiles	Phytophthora	parasitica INRA-310	hypothetical protein PPTG_02214 [Phytophthora parasitica INRA-310]
XP_008891491.1	463	PPTG_02214	eukaryota>stramenopiles	Phytophthora	parasitica INRA-310	hypothetical protein, variant 1 [Phytophthora parasitica INRA-310]
OHT10997.1	462	TRFO_19490	eukaryota>parabasalia	Tritrichomonas	foetus	hypothetical protein TRFO_19490 [Tritrichomonas foetus]
Smar1000004913	462	Smar1000004913	eukaryota>metazoa>arthropoda>myriapoda	Strigamia	maritima	>Smar1000004913 Smar1000004913
XP_421240.3	461	PPP2R3C	eukaryota>metazoa>chordata>vertebrata	Gallus	gallus	serine/threonine-protein phosphatase 2A regulatory subunit B'' subunit gamma isoform X1 [Gallus gallus]
XP_008878566.1	460	H310_13014	eukaryota>stramenopiles	Aphanomyces	invadans	hypothetical protein, variant 2 [Aphanomyces invadans]
XP_018084242.1	460	ppp2r3c	eukaryota>metazoa>chordata>vertebrata	Xenopus	laevis	PREDICTED: protein phosphatase 2 regulatory subunit B', gamma L homeolog isoform X1 [Xenopus laevis]
XP_019929912.1	460	LOC105345875	eukaryota>metazoa>mollusca	Crassostrea	gigas	PREDICTED: serine/threonine-protein phosphatase 2A regulatory subunit B'' subunit gamma isoform X1 [Crassostrea gigas]
XP_014669024.1	459	LOC106810248	eukaryota>metazoa	Priapulus	caudatus	PREDICTED: serine/threonine-protein phosphatase 2A regulatory subunit B'' subunit gamma-like [Priapulus caudatus]
XP_005844952.1	458	CHLNCDRAFT_138319	eukaryota>viridiplantae>chlorophyta	Chlorella	variabilis	hypothetical protein CHLNCDRAFT_138319 [Chlorella variabilis]
NP_956425.1	457	zgc:55473	eukaryota>metazoa>chordata>vertebrata>actinopterygii	Danio	rerio	serine/threonine-protein phosphatase 2A regulatory subunit B'' subunit gamma [Danio rerio]
OHS92702.1	457	TRFO_40976	eukaryota>parabasalia	Tritrichomonas	foetus	EF hand family protein [Tritrichomonas foetus]
XP_003976026.1	457	ppp2r3c	eukaryota>metazoa>chordata>vertebrata>actinopterygii	Takifugu	rubripes	PREDICTED: serine/threonine-protein phosphatase 2A regulatory subunit B'' subunit gamma [Takifugu rubripes]
KNE61607.1	455	AMAG_06420	eukaryota>fungi	Allomyces	macrogynus ATCC 38327	hypothetical protein AMAG_06420 [Allomyces macrogynus ATCC 38327]
XP_001949584.1	455	LOC100161436	eukaryota>metazoa>hexapoda	Acyrthosiphon	pisum	PREDICTED: serine/threonine-protein phosphatase 2A regulatory subunit B'' subunit gamma [Acyrthosiphon pisum]
XP_008184333.1	455	LOC100161436	eukaryota>metazoa>hexapoda	Acyrthosiphon	pisum	PREDICTED: serine/threonine-protein phosphatase 2A regulatory subunit B'' subunit gamma [Acyrthosiphon pisum]
XP_017331483.1	455	ppp2r3c	eukaryota>metazoa>chordata>vertebrata>actinopterygii	Ictalurus	punctatus	PREDICTED: serine/threonine-protein phosphatase 2A regulatory subunit B'' subunit gamma [Ictalurus punctatus]
XP_003382540.1	454	LOC100636686	eukaryota>metazoa	Amphimedon	queenslandica	PREDICTED: serine/threonine-protein phosphatase 2A regulatory subunit B'' subunit gamma-like [Amphimedon queenslandica]
XP_011452511.1	454	LOC105345875	eukaryota>metazoa>mollusca	Crassostrea	gigas	PREDICTED: serine/threonine-protein phosphatase 2A regulatory subunit B'' subunit gamma isoform X2 [Crassostrea gigas]
CEP00546.1	453	PBRA_001599	eukaryota>rhizaria>cercozoa>plasmodiophorida	Plasmodiophora	brassicae	hypothetical protein PBRA_001599 [Plasmodiophora brassicae]
ESU40922.1	453	GSB_17157	eukaryota>diplomonadida	Giardia	intestinalis	Putative calcium-binding family protein [Giardia intestinalis]
NP_060387.2	453	C14orf10,G4-1,G5pr	eukaryota>metazoa>chordata>vertebrata	Homo	sapiens	serine/threonine-protein phosphatase 2A regulatory subunit B'' subunit gamma isoform 1 [Homo sapiens]
NP_067504.2	453	4930511A21Rik,5730412A08Rik,G4-1,G5pr	eukaryota>metazoa>chordata>vertebrata	Mus	musculus	serine/threonine-protein phosphatase 2A regulatory subunit B'' subunit gamma [Mus musculus]
XP_001705084.1	453	GL50803_17157	eukaryota>diplomonadida	Giardia	lamblia ATCC 50803	Phosphatase subunit gene g4-1 [Giardia lamblia ATCC 50803]
XP_002609725.1	453	BRAFLDRAFT_102460	eukaryota>metazoa>chordata	Branchiostoma	floridae	hypothetical protein BRAFLDRAFT_102460 [Branchiostoma floridae]
XP_003263755.1	453	PPP2R3C	eukaryota>metazoa>chordata>vertebrata	Nomascus	leucogenys	PREDICTED: serine/threonine-protein phosphatase 2A regulatory subunit B'' subunit gamma isoform X1 [Nomascus leucogenys]
XP_005267839.1	453	C14orf10,G4-1,G5pr	eukaryota>metazoa>chordata>vertebrata	Homo	sapiens	serine/threonine-protein phosphatase 2A regulatory subunit B'' subunit gamma isoform X1 [Homo sapiens]
XP_005561131.1	453	PPP2R3C	eukaryota>metazoa>chordata>vertebrata	Macaca	fascicularis	PREDICTED: serine/threonine-protein phosphatase 2A regulatory subunit B'' subunit gamma isoform X1 [Macaca fascicularis]
XP_011151378.1	452	LOC105190361	eukaryota>metazoa>hexapoda	Harpegnathos	saltator	serine/threonine-protein phosphatase 2A regulatory subunit B'' subunit gamma [Harpegnathos saltator]
Caps1000015038	452	Caps1000015038	eukaryota>metazoa>annelida	Capitella	spI	>Caps1000015038 Caps1000015038
XP_002142643.1	452	CMU_020380	eukaryota>alveolata>apicomplexa	Cryptosporidium	muris RN66	hypothetical protein [Cryptosporidium muris RN66]
EPY35833.1	451	AGDE_07216	eukaryota>euglenozoa>kinetoplastida	Angomonas	deanei	hypothetical protein AGDE_07216 [Angomonas deanei]
XP_793008.2	451	LOC588222	eukaryota>metazoa>echinodermata	Strongylocentrotus	purpuratus	PREDICTED: serine/threonine-protein phosphatase 2A regulatory subunit B'' subunit gamma [Strongylocentrotus purpuratus]
KXJ24784.1	450	Ppp2r3c	eukaryota>metazoa>cnidaria	Exaiptasia	pallida	Serine/threonine-protein phosphatase 2A regulatory subunit B'' subunit gamma [Exaiptasia pallida]
XP_001436313.1	450	GSPATT00007128001	eukaryota>alveolata>ciliophora	Paramecium	tetraurelia strain d4-2	hypothetical protein (macronuclear) [Paramecium tetraurelia strain d4-2]
OHT17664.1	449	TRFO_00957	eukaryota>parabasalia	Tritrichomonas	foetus	EF hand family protein [Tritrichomonas foetus]
XP_001017961.2	449	TTHERM_00621360	eukaryota>alveolata>ciliophora	Tetrahymena	thermophila SB210	serine/threonine-protein phosphatase 2A regulatory subunit B subunit gamma [Tetrahymena thermophila SB210]
XP_004035024.1	449	IMG5_107580	eukaryota>alveolata>ciliophora	Ichthyophthirius	multifiliis	protein phosphatase 2 regulatory subunit b gamma, putative [Ichthyophthirius multifiliis]
XP_001449122.1	448	GSPATT00016538001	eukaryota>alveolata>ciliophora	Paramecium	tetraurelia strain d4-2	hypothetical protein (macronuclear) [Paramecium tetraurelia strain d4-2]
XP_001638819.1	448	NEMVEDRAFT_v1g161077	eukaryota>metazoa>cnidaria	Nematostella	vectensis	predicted protein [Nematostella vectensis]
XP_012554832.1	448	LOC100199816	eukaryota>metazoa>cnidaria	Hydra	vulgaris	PREDICTED: serine/threonine-protein phosphatase 2A regulatory subunit B'' subunit gamma-like [Hydra vulgaris]
XP_018084243.1	448	ppp2r3c	eukaryota>metazoa>chordata>vertebrata	Xenopus	laevis	PREDICTED: protein phosphatase 2 regulatory subunit B', gamma L homeolog isoform X2 [Xenopus laevis]

[illegible]

NP_001124163.1	511	CALN,CALNA,CALNA1,CCN1,CNA1,IECEE,IECEE1,PPP2B	eukaryota>metazoa>chordata>vertebrata	Homo	sapiens	serine/threonine-protein phosphatase 2B catalytic subunit alpha isoform isoform 2 [Homo sapiens]
NP_001087372.1	511	calcineurin,caln,calna,calna1,ccn1,cna1,ppp2b,ppp3ca	eukaryota>metazoa>chordata>vertebrata	Xenopus	laevis	protein phosphatase 3, catalytic subunit, alpha isozyme L homeolog [Xenopus laevis]
NP_001017856.1	511	wu:fq19c08,zgc:110141	eukaryota>metazoa>chordata>vertebrata>actinopterygii	Danio	rerio	serine/threonine-protein phosphatase 2B catalytic subunit alpha [Danio rerio]
XP_005155370.1	511	im:7163784,zgc:158685	eukaryota>metazoa>chordata>vertebrata>actinopterygii	Danio	rerio	protein phosphatase 3, catalytic subunit, gamma isoform X1 [Danio rerio]
XP_017327655.1	511	ppp3ca	eukaryota>metazoa>chordata>vertebrata>actinopterygii	Ictalurus	punctatus	PREDICTED: serine/threonine-protein phosphatase 2B catalytic subunit alpha isoform isoform X1 [Ictalurus punctatus]
EMH74210.1	511	EHI8A_054460	eukaryota>amoebozoa>entamoebidae	Entamoeba	histolytica HM-1:IMSS-B	calcineurin catalytic subunit A, putative [Entamoeba histolytica HM-1:IMSS-B]
EMD46097.1	511	EHI5A_107660	eukaryota>amoebozoa>entamoebidae	Entamoeba	histolytica KU27	serine/threonine protein phosphatase catalytic subunit gamma, putative [Entamoeba histolytica KU27]
EMH75764.1	511	EHI8A_075050	eukaryota>amoebozoa>entamoebidae	Entamoeba	histolytica HM-1:IMSS-B	calcineurin catalytic subunit A, putative [Entamoeba histolytica HM-1:IMSS-B]
EMS16518.1	511	KM1_134500	eukaryota>amoebozoa>entamoebidae	Entamoeba	histolytica HM-3:IMSS	serine/threonine protein phosphatase 2B catalytic subunit gamma isoform, putative [Entamoeba histolytica HM-3:IMSS]
ENY62767.1	511	EHI7A_072900	eukaryota>amoebozoa>entamoebidae	Entamoeba	histolytica HM-1:IMSS-A	serine/threonine protein phosphatase 2B catalytic subunit gamma isoform, putative [Entamoeba histolytica HM-1:IMSS-A]
GAT92021.1	511	CL6EHI_050920	eukaryota>amoebozoa>entamoebidae	Entamoeba	histolytica	calcineurin catalytic subunit a putative [Entamoeba histolytica]
XP_654180.2	511	EHI_050920	eukaryota>amoebozoa>entamoebidae	Entamoeba	histolytica HM-1:IMSS	calcineurin catalytic subunit A, putative [Entamoeba histolytica HM-1:IMSS]
ORY48385.1	510	LY90DRAFT_523579	eukaryota>fungi>chytridiomycota	Neocallimastix	californiae	Serine/threonine-protein phosphatase 2B catalytic subunit [Neocallimastix californiae]
XP_018080855.1	510	ppp3cc.L	eukaryota>metazoa>chordata>vertebrata	Xenopus	laevis	PREDICTED: serine/threonine-protein phosphatase 2B catalytic subunit beta isoform isoform X5 [Xenopus laevis]
XP_015141109.1	510	PPP3CA	eukaryota>metazoa>chordata>vertebrata	Gallus	gallus	serine/threonine-protein phosphatase 2B catalytic subunit alpha isoform isoform X6 [Gallus gallus]
XP_005562855.1	510	PPP3CC	eukaryota>metazoa>chordata>vertebrata	Macaca	fascicularis	PREDICTED: serine/threonine-protein phosphatase 2B catalytic subunit gamma isoform isoform X3 [Macaca fascicularis]
NP_001025511.1	510	PPP3CB	eukaryota>metazoa>chordata>vertebrata	Gallus	gallus	serine/threonine-protein phosphatase 2B catalytic subunit beta isoform [Gallus gallus]
XP_021336694.1	510	wu:fq19c08,zgc:110141	eukaryota>metazoa>chordata>vertebrata>actinopterygii	Danio	rerio	serine/threonine-protein phosphatase 2B catalytic subunit alpha isoform X2 [Danio rerio]
XP_015833482.1	510	LOC657135	eukaryota>metazoa>hexapoda	Tribolium	castaneum	PREDICTED: serine/threonine-protein phosphatase 2B catalytic subunit 2 isoform X1 [Tribolium castaneum]
XP_015833483.1	510	LOC657135	eukaryota>metazoa>hexapoda	Tribolium	castaneum	PREDICTED: serine/threonine-protein phosphatase 2B catalytic subunit 2 isoform X1 [Tribolium castaneum]
GAT93915.1	510	CL6EHI_118600	eukaryota>amoebozoa>entamoebidae	Entamoeba	histolytica	calcineurin catalytic subunit a putative [Entamoeba histolytica]
XP_001913593.1	510	EHI_118600	eukaryota>amoebozoa>entamoebidae	Entamoeba	histolytica HM-1:IMSS	calcineurin catalytic subunit A, putative [Entamoeba histolytica HM-1:IMSS]
XP_008856053.1	510	ENU1_050070	eukaryota>amoebozoa>entamoebidae	Entamoeba	nuttalli P19	calcineurin catalytic subunit A, putative [Entamoeba nuttalli P19]
XP_001448076.1	509	GSPATT00015557001	eukaryota>alveolata>ciliophora	Paramecium	tetraurelia strain d4-2	hypothetical protein (macronuclear) [Paramecium tetraurelia strain d4-2]
XP_022245394.1	509	LOC106465252	eukaryota>metazoa	Limulus	polyphemus	serine/threonine-protein phosphatase 2B catalytic subunit 2-like isoform X4 [Limulus polyphemus]
XP_022240920.1	509	LOC106458858	eukaryota>metazoa	Limulus	polyphemus	serine/threonine-protein phosphatase 2B catalytic subunit 2-like isoform X1 [Limulus polyphemus]
XP_016863854.1	509	CALN,CALNA,CALNA1,CCN1,CNA1,IECEE,IECEE1,PPP2B	eukaryota>metazoa>chordata>vertebrata	Homo	sapiens	serine/threonine-protein phosphatase 2B catalytic subunit alpha isoform isoform X1 [Homo sapiens]
XP_008199522.1	509	LOC657135	eukaryota>metazoa>hexapoda	Tribolium	castaneum	PREDICTED: serine/threonine-protein phosphatase 2B catalytic subunit 2 isoform X2 [Tribolium castaneum]
XP_015833484.1	509	LOC657135	eukaryota>metazoa>hexapoda	Tribolium	castaneum	PREDICTED: serine/threonine-protein phosphatase 2B catalytic subunit 2 isoform X3 [Tribolium castaneum]
XP_011445770.2	509	LOC105341116	eukaryota>metazoa>mollusca	Crassostrea	gigas	PREDICTED: serine/threonine-protein phosphatase 2B catalytic subunit 3 isoform X1 [Crassostrea gigas]
XP_001443973.1	508	GSPATT00012128001	eukaryota>alveolata>ciliophora	Paramecium	tetraurelia strain d4-2	hypothetical protein (macronuclear) [Paramecium tetraurelia strain d4-2]
XP_018089403.1	508	ppp3ca	eukaryota>metazoa>chordata>vertebrata	Xenopus	laevis	PREDICTED: protein phosphatase 3, catalytic subunit, alpha isoform isoform X1 [Xenopus laevis]
XP_005171933.1	508	ppp3cc,sb:cb767,si:dkey-21k4.5	eukaryota>metazoa>chordata>vertebrata>actinopterygii	Danio	rerio	protein phosphatase 3, catalytic subunit, gamma isoform-like isoform X1 [Danio rerio]
XP_015833485.1	508	LOC657135	eukaryota>metazoa>hexapoda	Tribolium	castaneum	PREDICTED: serine/threonine-protein phosphatase 2B catalytic subunit 2 isoform X4 [Tribolium castaneum]
XP_001450977.1	507	GSPATT00017910001	eukaryota>alveolata>ciliophora	Paramecium	tetraurelia strain d4-2	hypothetical protein (macronuclear) [Paramecium tetraurelia strain d4-2]
XP_001431776.1	507	GSPATT00033854001	eukaryota>alveolata>ciliophora	Paramecium	tetraurelia strain d4-2	hypothetical protein (macronuclear) [Paramecium tetraurelia strain d4-2]
XP_001430129.1	507	GSPATT00032583001	eukaryota>alveolata>ciliophora	Paramecium	tetraurelia strain d4-2	hypothetical protein (macronuclear) [Paramecium tetraurelia strain d4-2]
EJY65152.1	507	OXYTRI_14698	eukaryota>alveolata>ciliophora	Oxytricha	trifallax	Serine/threonine-protein phosphatase (macronuclear) [Oxytricha trifallax]
NP_001085039.1	507	calna2,calnb,cna2,pp2bbeta,ppp3cb	eukaryota>metazoa>chordata>vertebrata	Xenopus	laevis	protein phosphatase 3, catalytic subunit, beta isozyme L homeolog [Xenopus laevis]
XP_001456473.1	506	GSPATT00022245001	eukaryota>alveolata>ciliophora	Paramecium	tetraurelia strain d4-2	hypothetical protein (macronuclear) [Paramecium tetraurelia strain d4-2]
XP_001012424.2	506	TTHERM_00804750	eukaryota>alveolata>ciliophora	Tetrahymena	thermophila SB210	serine/threonine-protein phosphatase PP1 [Tetrahymena thermophila SB210]
XP_022240921.1	506	LOC106458858	eukaryota>metazoa	Limulus	polyphemus	serine/threonine-protein phosphatase 2B catalytic subunit 2-like isoform X2 [Limulus polyphemus]
NP_001074063.2	506	im:7163784,zgc:158685	eukaryota>metazoa>chordata>vertebrata>actinopterygii	Danio	rerio	protein phosphatase 3, catalytic subunit, gamma isoform [Danio rerio]
XP_009305999.1	506	wu:fq19c08,zgc:110141	eukaryota>metazoa>chordata>vertebrata>actinopterygii	Danio	rerio	serine/threonine-protein phosphatase 2B catalytic subunit alpha isoform X3 [Danio rerio]
XP_011678779.1	506	LOC588695	eukaryota>metazoa>echinodermata	Strongylocentrotus	purpuratus	PREDICTED: serine/threonine-protein phosphatase 2B catalytic subunit alpha isoform isoform X1 [Strongylocentrotus purpuratus]
CDW79014.1	505	Contig16048.g787	eukaryota>alveolata>ciliophora	Stylonychia	lemnae	UNKNOWN [Stylonychia lemnae]
XP_005161416.1	505	fj46e08,si:dkeyp-79c2.2,wu:fj46e08	eukaryota>metazoa>chordata>vertebrata>actinopterygii	Danio	rerio	protein phosphatase 3, catalytic subunit, alpha isozyme isoform X1 [Danio rerio]
XP_017307366.1	505	LOC108255714	eukaryota>metazoa>chordata>vertebrata>actinopterygii	Ictalurus	punctatus	PREDICTED: serine/threonine-protein phosphatase 2B catalytic subunit gamma isoform-like isoform X1 [Ictalurus punctatus]
XP_017340418.1	505	LOC108274683	eukaryota>metazoa>chordata>vertebrata>actinopterygii	Ictalurus	punctatus	PREDICTED: serine/threonine-protein phosphatase 2B catalytic subunit alpha isoform-like isoform X3 [Ictalurus punctatus]
XP_011678780.1	505	LOC588695	eukaryota>metazoa>echinodermata	Strongylocentrotus	purpuratus	PREDICTED: serine/threonine-protein phosphatase 2B catalytic subunit alpha isoform isoform X2 [Strongylocentrotus purpuratus]
XP_011445772.2	505	LOC105341116	eukaryota>metazoa>mollusca	Crassostrea	gigas	PREDICTED: serine/threonine-protein phosphatase 2B catalytic subunit alpha isoform isoform X2 [Crassostrea gigas]
XP_627753.1	504	cgd6_4200	eukaryota>alveolata>apicomplexa	Cryptosporidium	parvum Iowa II	protein phosphatase 2B catalytic subunit, calcineurin like phosphtase superfamily [Cryptosporidium parvum Iowa II]
XP_001452806.1	504	GSPATT00002809001	eukaryota>alveolata>ciliophora	Paramecium	tetraurelia strain d4-2	hypothetical protein (macronuclear) [Paramecium tetraurelia strain d4-2]
XP_001442415.1	504	GSPATT00001220001	eukaryota>alveolata>ciliophora	Paramecium	tetraurelia strain d4-2	hypothetical protein (macronuclear) [Paramecium tetraurelia strain d4-2]
XP_013379079.1	504	LOC106150691	eukaryota>metazoa	Lingula	anatina	serine/threonine-protein phosphatase 2B catalytic subunit 2-like isoform X1 [Lingula anatina]
XP_003876556.1	503	LMXM_26_2530	eukaryota>euglenozoa>kinetoplastida	Leishmania	mexicana MHOM/GT/2001/U1103	putative serine/threonine protein phosphatase [Leishmania mexicana MHOM/GT/2001/U1103]
XP_006518777.1	503	Calnc,PP2BA,gamma	eukaryota>metazoa>chordata>vertebrata	Mus	musculus	PREDICTED: serine/threonine-protein phosphatase 2B catalytic subunit gamma isoform isoform X1 [Mus musculus]
[illegible]	[illegible]	[illegible]	[illegible]	[illegible]	[illegible]	[illegible]

[illegible]

XP_004261838.1	179	EIN_253310	eukaryota>amoebozoa>entamoebidae	Entamoeba	invadens IP1	calcineurin subunit B, putative [Entamoeba invadens IP1]
XP_008860323.1	179	ENU1_200580	eukaryota>amoebozoa>entamoebidae	Entamoeba	nuttalli P19	calcineurin B subunit, putative [Entamoeba nuttalli P19]
XP_011777215.1	179	TbgDal_X300	eukaryota>euglenozoa>kinetoplastida	Trypanosoma	brucei gambiense DAL972	calcineurin B subunit, putative [Trypanosoma brucei gambiense DAL972]
NP_001004025.1	179	CaNB2,CnB2	eukaryota>metazoa>chordata>vertebrata	Mus	musculus	calcineurin subunit B type 2 [Mus musculus]
XP_016612902.1	178	SPPG_00562	eukaryota>fungi>chytridiomycota	Spizellomyces	punctatus DAOM BR117	hypothetical protein SPPG_00562 [Spizellomyces punctatus DAOM BR117]
XP_004349371.2	178	CAOG_02621	eukaryota>ichthyosporea	Capsaspora	owczarzaki ATCC 30864	calcium-binding protein p22 [Capsaspora owczarzaki ATCC 30864]
CEM08560.1	177	Vbra_14626	eukaryota>alveolata	Vitrella	brassicaformis CCMP3155	unnamed protein product [Vitrella brassicaformis CCMP3155]
XP_001348666.2	177	PF3D7_1451700	eukaryota>alveolata>apicomplexa	Plasmodium	falciparum 3D7	calcineurin subunit B [Plasmodium falciparum 3D7]
XP_001615872.1	177	PVX_117895	eukaryota>alveolata>apicomplexa	Plasmodium	vivax	calcineurin B subunit isoform 1, putative [Plasmodium vivax]
XP_002371117.1	177	TGME49_213800	eukaryota>alveolata>apicomplexa	Toxoplasma	gondii ME49	protein phosphatase 2b regulatory subunit, putative [Toxoplasma gondii ME49]
XP_003881594.1	177	NCLIV_013540	eukaryota>alveolata>apicomplexa	Neospora	caninum Liverpool	hypothetical protein NCLIV_013540 [Neospora caninum Liverpool]
XP_008884045.1	177	HHA_213800	eukaryota>alveolata>apicomplexa	Hammondia	hammondi	protein phosphatase 2b regulatory subunit, putative [Hammondia hammondi]
XP_013234209.1	177	ETH_00025715	eukaryota>alveolata>apicomplexa	Eimeria	tenella	protein phosphatase 2b regulatory subunit, putative [Eimeria tenella]
OMJ81238.1	177	SteCoe_18356	eukaryota>alveolata>ciliophora	Stentor	coeruleus	hypothetical protein SteCoe_18356 [Stentor coeruleus]
XP_009310883.1	176	DQ04_03191000	eukaryota>euglenozoa>kinetoplastida	Trypanosoma	grayi	calcineurin B subunit [Trypanosoma grayi]
XP_808544.1	176	Tc00.1047053506869.50	eukaryota>euglenozoa>kinetoplastida	Trypanosoma	cruzi strain CL Brener	calcineurin B subunit [Trypanosoma cruzi strain CL Brener]
XP_812709.1	176	Tc00.1047053510519.60	eukaryota>euglenozoa>kinetoplastida	Trypanosoma	cruzi strain CL Brener	calcineurin B subunit [Trypanosoma cruzi strain CL Brener]
XP_019015494.1	176	PICMEDRAFT_18295	eukaryota>fungi>ascomycota	Pichia	membranifaciens NRRL Y-2026	hypothetical protein PICMEDRAFT_18295 [Pichia membranifaciens NRRL Y-2026]
Pisp1000007246	176	Pisp1000007246	eukaryota>fungi>neocallimastigomycota	Piromyces	sp	>Pisp1000007246
EST47010.1	176	SS50377_12965	eukaryota>diplomonadida	Spironucleus	salmonicida	Calcineurin regulatory subunit B [Spironucleus salmonicida]
XP_006459824.1	175	AGABI2DRAFT_135141	eukaryota>fungi>basidiomycota	Agaricus	bisporus var. bisporus H97	hypothetical protein AGABI2DRAFT_135141 [Agaricus bisporus var. bisporus H97]
XP_021868731.1	175	BD324DRAFT_636044	eukaryota>fungi>basidiomycota	Kockovaella	imperatae	putative calcium-dependent protein serine/threonine phosphatase [Kockovaella imperatae]
ORY76768.1	175	LY90DRAFT_377314	eukaryota>fungi>chytridiomycota	Neocallimastix	californiae	EF-hand [Neocallimastix californiae]
ORY75892.1	175	LY90DRAFT_377653	eukaryota>fungi>chytridiomycota	Neocallimastix	californiae	EF-hand [Neocallimastix californiae]
CCW70383.1	175	GSHART1_T00007958001	eukaryota>euglenozoa>kinetoplastida	Phytomonas	sp. isolate Hart1	unnamed protein product [Phytomonas sp. isolate Hart1]
EPY36052.1	175	STCU_00779	eukaryota>euglenozoa>kinetoplastida	Strigomonas	culicis	protein phosphatase 3, regulatory subunit [Strigomonas culicis]
KPI84786.1	175	ABL78_6169	eukaryota>euglenozoa>kinetoplastida	Leptomonas	seymouri	putative calcineurin B subunit [Leptomonas seymouri]
XP_001465459.1	175	LINJ_21_1990	eukaryota>euglenozoa>kinetoplastida	Leishmania	infantum JPCM5	putative calcineurin B subunit [Leishmania infantum JPCM5]
XP_001564893.1	175	LBRM_21_1920	eukaryota>euglenozoa>kinetoplastida	Leishmania	braziliensis MHOM/BR/75/M2904	putative calcineurin B subunit [Leishmania braziliensis MHOM/BR/75/M2904]
XP_001683127.1	175	LMJF_21_1630	eukaryota>euglenozoa>kinetoplastida	Leishmania	major strain Friedlin	putative calcineurin B subunit [Leishmania major strain Friedlin]
XP_003860716.1	175	LDBPK_211990	eukaryota>euglenozoa>kinetoplastida	Leishmania	donovani	calcineurin B subunit, putative [Leishmania donovani]
XP_003875430.1	175	LMXM_21_1630	eukaryota>euglenozoa>kinetoplastida	Leishmania	mexicana MHOM/GT/2001/U1103	putative calcineurin B subunit [Leishmania mexicana MHOM/GT/2001/U1103]
XP_010698978.1	175	CNB	eukaryota>euglenozoa>kinetoplastida	Leishmania	panamensis	calcineurin B subunit, putative [Leishmania panamensis]
XP_015656821.1	175	ABB37_06498	eukaryota>euglenozoa>kinetoplastida	Leptomonas	pyrrhocoris	calcineurin b subunit (CNB) [Leptomonas pyrrhocoris]
XP_015656822.1	175	ABB37_06498	eukaryota>euglenozoa>kinetoplastida	Leptomonas	pyrrhocoris	calcineurin b subunit (CNB) [Leptomonas pyrrhocoris]
XP_015656823.1	175	ABB37_06498	eukaryota>euglenozoa>kinetoplastida	Leptomonas	pyrrhocoris	calcineurin b subunit (CNB) [Leptomonas pyrrhocoris]
KNE65487.1	175	AMAG_11106	eukaryota>fungi	Allomyces	macrogynus ATCC 38327	calcineurin subunit B [Allomyces macrogynus ATCC 38327]
KNE72653.1	175	AMAG_16414	eukaryota>fungi	Allomyces	macrogynus ATCC 38327	calcineurin subunit B [Allomyces macrogynus ATCC 38327]
NP_012731.1	175	CRV1,YCN2	eukaryota>fungi>ascomycota	Saccharomyces	cerevisiae S288C	calcineurin regulatory subunit B [Saccharomyces cerevisiae S288C]
XP_002837238.1	175	GSTUM_00005170001	eukaryota>fungi>ascomycota	Tuber	melanosporum Mel28	hypothetical protein [Tuber melanosporum Mel28]
XP_453201.1	175	KLLA0_D02992g	eukaryota>fungi>ascomycota	Kluyveromyces	lactis	uncharacterized protein KLLA0_D02992g [Kluyveromyces lactis]
Chet1000004836	175	Chet1000004836	eukaryota>fungi>ascomycota	Cochliobolus	heterostrophus	>Chet1000004836
EPT03113.1	175	FOMPIDRAFT_1022481	eukaryota>fungi>basidiomycota	Fomitopsis	pinicola FP-58527 SS1	hypothetical protein FOMPIDRAFT_1022481 [Fomitopsis pinicola FP-58527 SS1]
XP_001839327.2	175	CC1G_08194	eukaryota>fungi>basidiomycota	Coprinopsis	cinerea okayama7#130	calcium-dependent protein serine/threonine phosphatase [Coprinopsis cinerea okayama7#130]
XP_001884421.1	175	LACBIDRAFT_291275	eukaryota>fungi>basidiomycota	Laccaria	bicolor S238N-H82	predicted protein [Laccaria bicolor S238N-H82]
XP_002473674.1	175	CAR1	eukaryota>fungi>basidiomycota	Postia	placenta Mad-698-R	calcium/calmodulin-dependent protein phosphatase [Postia placenta Mad-698-R]
XP_003322771.1	175	PGTG_04308	eukaryota>fungi>basidiomycota	Puccinia	graminis f. sp. tritici CRL 75-36-700-3	calcineurin subunit B [Puccinia graminis f. sp. tritici CRL 75-36-700-3]
XP_006958264.1	175	WALSEDRAFT_60317	eukaryota>fungi>basidiomycota	Wallemia	mellicola CBS 633.66	calcium-dependent protein serine/threonine phosphatase [Wallemia mellicola CBS 633.66]
XP_007333662.1	175	AGABI1DRAFT_116228	eukaryota>fungi>basidiomycota	Agaricus	bisporus var. burnettii JB137-S8	hypothetical protein AGABI1DRAFT_116228 [Agaricus bisporus var. burnettii JB137-S8]
XP_011387312.1	175	UMAG_10226	eukaryota>fungi>basidiomycota	Ustilago	maydis 521	putative calcineurin regulatory subunit B [Ustilago maydis 521]
XP_012049171.1	175	CNAG_00888	eukaryota>fungi>basidiomycota	Cryptococcus	neoformans var. grubii H99	calcineurin subunit B [Cryptococcus neoformans var. grubii H99]
XP_013240503.1	175	K437DRAFT_259661	eukaryota>fungi>basidiomycota	Tilletiaria	anomala UBC 951	putative CNB1-calcineurin B, regulatory subunit [Tilletiaria anomala UBC 951]
XP_014571409.1	175	L969DRAFT_101264	eukaryota>fungi>basidiomycota	Mixia	osmundae IAM 14324	hypothetical protein L969DRAFT_101264 [Mixia osmundae IAM 14324]
XP_017992920.1	175	Malapachy_2482	eukaryota>fungi>basidiomycota	Malassezia	pachydermatis	calcium calmodulin-dependent protein phosphatase [Malassezia pachydermatis]
XP_018273257.1	175	RHOBADRAFT_64678	eukaryota>fungi>basidiomycota	Rhodotorula	graminis WP1	hypothetical protein RHOBADRAFT_64678 [Rhodotorula graminis WP1]
XP_018280614.1	175	CC85DRAFT_311339	eukaryota>fungi>basidiomycota	Cutaneotrichosporon	oleaginosum	calcium-dependent protein serine/threonine phosphatase [Cutaneotrichosporon oleaginosum]
XP_006677028.1	175	BATDEDRAFT_86479	eukaryota>fungi>chytridiomycota	Batrachochytrium	dendrobatidis JAM81	hypothetical protein BATDEDRAFT_86479 [Batrachochytrium dendrobatidis JAM81]
XP_016609494.1	175	SPPG_03259	eukaryota>fungi>chytridiomycota	Spizellomyces	punctatus DAOM BR117	calcineurin subunit B, variant 2 [Spizellomyces punctatus DAOM BR117]
XP_016609495.1	175	SPPG_03259	eukaryota>fungi>chytridiomycota	Spizellomyces	punctatus DAOM BR117	calcineurin subunit B, variant 1 [Spizellomyces punctatus DAOM BR117]
XP_016609496.1	175	SPPG_03259	eukaryota>fungi>chytridiomycota	Spizellomyces	punctatus DAOM BR117	calcineurin subunit B [Spizellomyces punctatus DAOM BR117]
XP_009305321.1	175	canbl,ppp3r1,si:ch211-274c22.8	eukaryota>metazoa>chordata>vertebrata>actinopterygii	Danio	rerio	calcineurin subunit B type 1 isoform X1 [Danio rerio]
OZJ06015.1	174	BZG36_01073	eukaryota>fungi	Bifiguratus	adelaidae	Calcineurin subunit B [Bifiguratus adelaidae]
ESA14576.1	174	GLOINDRAFT_78398	eukaryota>fungi	Rhizophagus	irregularis DAOM 181602	hypothetical protein GLOINDRAFT_78398 [Rhizophagus irregularis DAOM 181602]
EPY40284.1	174	AGDE_03644	eukaryota>euglenozoa>kinetoplastida	Angomonas	deanei	protein phosphatase 3, regulatory subunit [Angomonas deanei]
EPY41157.1	174	AGDE_02768	eukaryota>euglenozoa>kinetoplastida	Angomonas	deanei	protein phosphatase 3, regulatory subunit [Angomonas deanei]
KFH64201.1	174	MVEG_10026	eukaryota>fungi	Mortierella	verticillata NRRL 6337	calcineurin subunit B [Mortierella verticillata NRRL 6337]
KXN71831.1	174	CONCODRAFT_81546	eukaryota>fungi	Conidiobolus	coronatus NRRL 28638	calcineurin regulatory subunit B [Conidiobolus coronatus NRRL 28638]
NP_588476.1	174	SPCC830.06	eukaryota>fungi>ascomycota	Schizosaccharomyces	pombe	putative calcineurin regulatory subunit B [Schizosaccharomyces pombe]
XP_001224922.1	174	CHGG_07266	eukaryota>fungi>ascomycota	Chaetomium	globosum CBS 148.51	calcineurin B subunit [Chaetomium globosum CBS 148.51]
XP_002174924.1	174	SJAG_03793	eukaryota>fungi>ascomycota	Schizosaccharomyces	japonicus yFS275	calcineurin regulatory subunit [Schizosaccharomyces japonicus yFS275]
XP_002619548.1	174	CLUG_00707	eukaryota>fungi>ascomycota	Clavispora	lusitaniae ATCC 42720	hypothetical protein CLUG_00707 [Clavispora lusitaniae ATCC 42720]
XP_011128081.1	174	AOL_s00215g577	eukaryota>fungi>ascomycota	Arthrobotrys	oligospora ATCC 24927	hypothetical protein AOL_s00215g577 [Arthrobotrys oligospora ATCC 24927]
XP_011394604.1	174	cnb-1	eukaryota>fungi>ascomycota	Neurospora	crassa OR74A	calcineurin subunit B, variant [Neurospora crassa OR74A]
XP_011394605.1	174	cnb-1	eukaryota>fungi>ascomycota	Neurospora	crassa OR74A	calcineurin subunit B [Neurospora crassa OR74A]
XP_013431767.1	174	M436DRAFT_36010	eukaryota>fungi>ascomycota	Aureobasidium	namibiae CBS 147.97	calcineurin regulatory beta sub-unit [Aureobasidium namibiae CBS 147.97]
XP_018188214.1	174	L228DRAFT_132894	eukaryota>fungi>ascomycota	Xylona	heveae TC161	calcineurin regulatory subunit B [Xylona heveae TC161]
XP_019026807.1	174	SAICODRAFT_4925	eukaryota>fungi>ascomycota	Saitoella	complicata NRRL Y-17804	EF-hand [Saitoella complicata NRRL Y-17804]
XP_019039581.1	174	WICANDRAFT_30997	eukaryota>fungi>ascomycota	Wickerhamomyces	anomalus NRRL Y-366-8	hypothetical protein WICANDRAFT_30997 [Wickerhamomyces anomalus NRRL Y-366-8]
Bcir1000010804	174	Bcir1000010804	eukaryota>fungi>mucoromycotina	Backusella	circina	>Bcir1000010804
Uram1000004173	174	Uram1000004173	eukaryota>fungi>mucoromycotina	Umbelopsis	ramanniana	>Uram1000004173
OAD01308.1	173	MUCCIDRAFT_156765	eukaryota>fungi	Mucor	circinelloides f. lusitanicus CBS 277.49	hypothetical protein MUCCIDRAFT_156765 [Mucor circinelloides f. lusitanicus CBS 277.49]
XP_002774673.1	173	Pmar_PMAR006301	eukaryota>alveolata	Perkinsus	marinus ATCC 50983	protein phosphatase 2B regulatory subunit, putative [Perkinsus marinus ATCC 50983]
XP_004993056.1	173	PTSG_06164	eukaryota>choanoflagellida	Salpingoeca	rosetta	calcineurin B [Salpingoeca rosetta]
EPB89794.1	173	HMPREF1544_03315	eukaryota>fungi	Mucor	circinelloides f. circinelloides 1006PhL	calcineurin subunit B [Mucor circinelloides f. circinelloides 1006PhL]
XP_018285925.1	173	Pp2B2	eukaryota>fungi	Phycomyces	blakesleeanus NRRL 1555(-)	calcineurin regulatory B subunit [Phycomyces blakesleeanus NRRL 1555(-)]
XP_018293120.1	173	PHYBLDRAFT_32453	eukaryota>fungi	Phycomyces	blakesleeanus NRRL 1555(-)	hypothetical protein PHYBLDRAFT_32453 [Phycomyces blakesleeanus NRRL 1555(-)]
XP_500266.1	173	YALI0_A19976g	eukaryota>fungi>ascomycota	Yarrowia	lipolytica CLIB122	YALI0A19976p [Yarrowia lipolytica CLIB122]
XP_721917.2	173	CNB1	eukaryota>fungi>ascomycota	Candida	albicans SC5314	calcineurin regulatory subunit B [Candida albicans SC5314]
XP_009015627.1	173	HELRODRAFT_65068	eukaryota>metazoa>annelida	Helobdella	robusta	hypothetical protein HELRODRAFT_65068 [Helobdella robusta]
NP_001271902.1	173	PPP3R2	eukaryota>metazoa>chordata>vertebrata	Macaca	fascicularis	calcineurin subunit B type 2 [Macaca fascicularis]
NP_671709.1	173	PPP3RL	eukaryota>metazoa>chordata>vertebrata	Homo	sapiens	calcineurin subunit B type 2 [Homo sapiens]
OQV25169.1	172	BV898_00858	eukaryota>metazoa	Hypsibius	dujardini	Calcineurin subunit B type 1 [Hypsibius dujardini]
OQV24122.1	172	BV898_02074	eukaryota>metazoa	Hypsibius	dujardini	Calcineurin subunit B type 1 [Hypsibius dujardini]
OQV15884.1	172	BV898_09980	eukaryota>metazoa	Hypsibius	dujardini	Calcineurin subunit B type 2 [Hypsibius dujardini]
CDW90481.1	172	Contig17535.g18653	eukaryota>alveolata>ciliophora	Stylonychia	lemnae	protein phosphatase 2b regulatory subunit [Stylonychia lemnae]
EJY75358.1	172	OXYTRI_03256	eukaryota>alveolata>ciliophora	Oxytricha	trifallax	Ca2+-binding protein (EF-Hand superfamily) (macronuclear) [Oxytricha trifallax]
OAF69791.1	172	A3Q56_02450	eukaryota>metazoa	Intoshia	linei	Calcineurin regulatory subunit [Intoshia linei]
XP_005706567.1	172	Gasu_26330	eukaryota>rhodophyta	Galdieria	sulphuraria	calcineurin subunit B [Galdieria sulphuraria]
XP_005710147.1	172	CHC_T00006835001	eukaryota>rhodophyta	Chondrus	crispus	unnamed protein product [Chondrus crispus]
XP_004365186.1	171	CAOG_00315	eukaryota>ichthyosporea	Capsaspora	owczarzaki ATCC 30864	calcineurin b subunit [Capsaspora owczarzaki ATCC 30864]
XP_003387288.1	171	LOC100640962	eukaryota>metazoa	Amphimedon	queenslandica	PREDICTED: calcineurin subunit B type 1-like [Amphimedon queenslandica]
Caps1000013019	171	Caps1000013019	eukaryota>metazoa>annelida	Capitella	spI	>Caps1000013019
ERG86812.1	171	ASU_01634	eukaryota>metazoa>nematoda	Ascaris	suum	calcineurin subunit b type 2 [Ascaris suum]
NP_001256318.1	171	cnb-1	eukaryota>metazoa>nematoda	Caenorhabditis	elegans	Protein phosphatase 2B regulatory subunit cnb-1 [Caenorhabditis elegans]
Smed1000006744	171	Smed1000006744	eukaryota>metazoa>platyhelminthe	Schmidtea	mediterranea	>Smed1000006744
Smed1000020783	171	Smed1000020783	eukaryota>metazoa>platyhelminthe	Schmidtea	mediterranea	>Smed1000020783
XP_012753544.1	170	SAMD00019534_069780	eukaryota>amoebozoa>mycetozoa>dictyosteliida	Acytostelium	subglobosum LB1	hypothetical protein SAMD00019534_069780 [Acytostelium subglobosum LB1]
XP_013395931.1	170	LOC106163012	eukaryota>metazoa	Lingula	anatina	calcineurin subunit B type 1 [Lingula anatina]
XP_013774201.1	170	LOC106459153	eukaryota>metazoa	Limulus	polyphemus	calcineurin subunit B type 2 [Limulus polyphemus]
XP_013775420.1	170	LOC106460277	eukaryota>metazoa	Limulus	polyphemus	calcineurin subunit B type 2-like [Limulus polyphemus]
XP_013778693.1	170	LOC106463237	eukaryota>metazoa	Limulus	polyphemus	calcineurin subunit B type 2-like [Limulus polyphemus]
XP_013793679.1	170	LOC106477682	eukaryota>metazoa	Limulus	polyphemus	calcineurin subunit B type 2-like [Limulus polyphemus]
XP_014665054.1	170	LOC106807272	eukaryota>metazoa	Priapulus	caudatus	PREDICTED: calcineurin subunit B type 1 [Priapulus caudatus]
XP_009028846.1	170	HELRODRAFT_69604	eukaryota>metazoa>annelida	Helobdella	robusta	hypothetical protein HELRODRAFT_69604, partial [Helobdella robusta]
XP_002130765.1	170	LOC100187330	eukaryota>metazoa>chordata	Ciona	intestinalis	PREDICTED: calcineurin subunit B type 1 [Ciona intestinalis]
NP_000936.1	170	CALNB1,CNB,CNB1	eukaryota>metazoa>chordata>vertebrata	Homo	sapiens	calcineurin subunit B type 1 [Homo sapiens]
NP_001083966.1	170	canb,ppp3r1	eukaryota>metazoa>chordata>vertebrata	Xenopus	laevis	calcineurin subunit B type 1 [Xenopus laevis]
NP_077779.2	170	CaNB1,Cnb1,MCIP1	eukaryota>metazoa>chordata>vertebrata	Mus	musculus	calcineurin subunit B type 1 [Mus musculus]
NP_989707.1	170	HZGJ	eukaryota>metazoa>chordata>vertebrata	Gallus	gallus	calcineurin subunit B type 1 [Gallus gallus]
XP_003262525.1	170	PPP3R1	eukaryota>metazoa>chordata>vertebrata	Nomascus	leucogenys	PREDICTED: calcineurin subunit B type 1 isoform X2 [Nomascus leucogenys]
XP_005575763.1	170	PPP3R1	eukaryota>metazoa>chordata>vertebrata	Macaca	fascicularis	PREDICTED: calcineurin subunit B type 1 isoform X2 [Macaca fascicularis]
XP_018118297.1	170	LOC108716580	eukaryota>metazoa>chordata>vertebrata	Xenopus	laevis	PREDICTED: calcineurin subunit B type 1 [Xenopus laevis]
NP_001004553.1	170	zgc:92169	eukaryota>metazoa>chordata>vertebrata>actinopterygii	Danio	rerio	protein phosphatase 3 (formerly 2B), regulatory subunit B, alpha isoform, b [Danio rerio]
NP_001314701.1	170	canbl,ppp3r1,si:ch211-274c22.8	eukaryota>metazoa>chordata>vertebrata>actinopterygii	Danio	rerio	calcineurin subunit B type 1 [Danio rerio]
XP_003972235.1	170	LOC101071292	eukaryota>metazoa>chordata>vertebrata>actinopterygii	Takifugu	rubripes	PREDICTED: calcineurin subunit B type 1-like [Takifugu rubripes]
XP_011601394.1	170	ppp3r1	eukaryota>metazoa>chordata>vertebrata>actinopterygii	Takifugu	rubripes	PREDICTED: calcineurin subunit B type 1 [Takifugu rubripes]
XP_017312712.1	170	LOC108258530	eukaryota>metazoa>chordata>vertebrata>actinopterygii	Ictalurus	punctatus	PREDICTED: calcineurin subunit B type 1 [Ictalurus punctatus]
XP_017319310.1	170	ppp3r1	eukaryota>metazoa>chordata>vertebrata>actinopterygii	Ictalurus	punctatus	PREDICTED: calcineurin subunit B type 1 [Ictalurus punctatus]
KXJ10544.1	170	Ppp3r1	eukaryota>metazoa>cnidaria	Exaiptasia	pallida	Calcineurin subunit B type 1 [Exaiptasia pallida]
XP_001640598.1	170	NEMVEDRAFT_v1g159419	eukaryota>metazoa>cnidaria	Nematostella	vectensis	predicted protein [Nematostella vectensis]
XP_004206833.1	170	LOC100213359	eukaryota>metazoa>cnidaria	Hydra	vulgaris	PREDICTED: calcineurin subunit B type 1-like [Hydra vulgaris]
XP_012554555.1	170	LOC100213359	eukaryota>metazoa>cnidaria	Hydra	vulgaris	PREDICTED: calcineurin subunit B type 1-like [Hydra vulgaris]
EFX75418.1	170	DAPPUDRAFT_306765	eukaryota>metazoa>crustacea	Daphnia	pulex	hypothetical protein DAPPUDRAFT_306765 [Daphnia pulex]
XP_011666356.1	170	LOC590811	eukaryota>metazoa>echinodermata	Strongylocentrotus	purpuratus	PREDICTED: calcineurin subunit B type 2 isoform X1 [Strongylocentrotus purpuratus]
XP_795492.2	170	LOC590811	eukaryota>metazoa>echinodermata	Strongylocentrotus	purpuratus	PREDICTED: calcineurin subunit B type 2 isoform X2 [Strongylocentrotus purpuratus]
NP_001161511.1	170	LOC100313566	eukaryota>metazoa>hemichordata	Saccoglossus	kowalevskii	calcineurin B regulatory subunit [Saccoglossus kowalevskii]
NP_001037026.1	170	Cnb	eukaryota>metazoa>hexapoda	Bombyx	mori	calcineurin B [Bombyx mori]
NP_001155538.1	170	LOC100162564	eukaryota>metazoa>hexapoda	Acyrthosiphon	pisum	calcineurin B-like [Acyrthosiphon pisum]
NP_001245531.1	170	BcDNA:RH11383,Can,CaNB,canB-4F,CG4209,CnB,CNB,dCnB1,Dmel\CG4209	eukaryota>metazoa>hexapoda	Drosophila	melanogaster	calcineurin B, isoform B [Drosophila melanogaster]
NP_001246192.1	170	canB2,CG11217,CS2-1,dCnB2,dCNB2,Dmel\CG11217	eukaryota>metazoa>hexapoda	Drosophila	melanogaster	calcineurin B2, isoform B [Drosophila melanogaster]
NP_524741.1	170	BcDNA:RH11383,Can,CaNB,canB-4F,CG4209,CnB,CNB,dCnB1,Dmel\CG4209	eukaryota>metazoa>hexapoda	Drosophila	melanogaster	calcineurin B, isoform A [Drosophila melanogaster]
NP_524874.2	170	canB2,CG11217,CS2-1,dCnB2,dCNB2,Dmel\CG11217	eukaryota>metazoa>hexapoda	Drosophila	melanogaster	calcineurin B2, isoform A [Drosophila melanogaster]
XP_001864848.1	170	CpipJ_CPIJ014603	eukaryota>metazoa>hexapoda	Culex	quinquefasciatus	calcineurin b subunit [Culex quinquefasciatus]
XP_008185525.1	170	LOC100162564	eukaryota>metazoa>hexapoda	Acyrthosiphon	pisum	PREDICTED: calcineurin B-like isoform X1 [Acyrthosiphon pisum]
XP_008185526.1	170	LOC100162564	eukaryota>metazoa>hexapoda	Acyrthosiphon	pisum	PREDICTED: calcineurin B-like isoform X1 [Acyrthosiphon pisum]
XP_011138781.1	170	LOC105182789	eukaryota>metazoa>hexapoda	Harpegnathos	saltator	calcineurin subunit B type 2 [Harpegnathos saltator]
XP_975270.1	170	LOC664163	eukaryota>metazoa>hexapoda	Tribolium	castaneum	PREDICTED: calcineurin subunit B type 2 [Tribolium castaneum]
XP_009055806.1	170	LOTGIDRAFT_216002	eukaryota>metazoa>mollusca	Lottia	gigantea	hypothetical protein LOTGIDRAFT_216002 [Lottia gigantea]
XP_014783704.1	170	LOC106878873	eukaryota>metazoa>mollusca	Octopus	bimaculoides	PREDICTED: calcineurin subunit B type 1 [Octopus bimaculoides]
NP_001256317.1	170	cnb-1	eukaryota>metazoa>nematoda	Caenorhabditis	elegans	CalciNeurin B [Caenorhabditis elegans]
XP_001433234.1	169	GSPATT00006336001	eukaryota>alveolata>ciliophora	Paramecium	tetraurelia strain d4-2	hypothetical protein (macronuclear) [Paramecium tetraurelia strain d4-2]
XP_001440742.1	169	GSPATT00009660001	eukaryota>alveolata>ciliophora	Paramecium	tetraurelia strain d4-2	hypothetical protein (macronuclear) [Paramecium tetraurelia strain d4-2]
Crev1000005114	169	Crev1000005114	eukaryota>fungi>kickxellomycotina	Coemansia	reversa	>Crev1000005114
CDS37303.1	169	EmuJ_000454300	eukaryota>metazoa	Echinococcus	multilocularis	calcineurin b [Echinococcus multilocularis]
EUB62734.1	169	EGR_02530	eukaryota>metazoa	Echinococcus	granulosus	Calcineurin subunit B type [Echinococcus granulosus]
XP_018655028.1	169	Smp_064950	eukaryota>metazoa	Schistosoma	mansoni	putative calcineurin B [Schistosoma mansoni]
XP_002610272.1	169	BRAFLDRAFT_92996	eukaryota>metazoa>chordata	Branchiostoma	floridae	hypothetical protein BRAFLDRAFT_92996 [Branchiostoma floridae]
CAF92532.1	169	GSTEN:00007813:G:001	eukaryota>metazoa>chordata>vertebrata>actinopterygii	Tetraodon	nigroviridis	unnamed protein product, partial [Tetraodon nigroviridis]
XP_015142553.1	168	CHP	eukaryota>metazoa>chordata>vertebrata	Gallus	gallus	PREDICTED: calcineurin B homologous protein 1 isoform X1 [Gallus gallus]
OMJ73447.1	167	SteCoe_27864	eukaryota>alveolata>ciliophora	Stentor	coeruleus	hypothetical protein SteCoe_27864 [Stentor coeruleus]
OMJ76356.1	167	SteCoe_24298	eukaryota>alveolata>ciliophora	Stentor	coeruleus	hypothetical protein SteCoe_24298 [Stentor coeruleus]
OMJ78341.1	167	SteCoe_21859	eukaryota>alveolata>ciliophora	Stentor	coeruleus	hypothetical protein SteCoe_21859 [Stentor coeruleus]
OMJ78523.1	167	SteCoe_21628	eukaryota>alveolata>ciliophora	Stentor	coeruleus	hypothetical protein SteCoe_21628 [Stentor coeruleus]
OMJ82650.1	167	SteCoe_16571	eukaryota>alveolata>ciliophora	Stentor	coeruleus	hypothetical protein SteCoe_16571 [Stentor coeruleus]
OMJ83526.1	167	SteCoe_15558	eukaryota>alveolata>ciliophora	Stentor	coeruleus	hypothetical protein SteCoe_15558 [Stentor coeruleus]
XP_001011228.1	167	TTHERM_00145890	eukaryota>alveolata>ciliophora	Tetrahymena	thermophila SB210	calcineurin Ca2+binding regulatory subunit CnaB [Tetrahymena thermophila SB210]
XP_004036834.1	167	IMG5_068710	eukaryota>alveolata>ciliophora	Ichthyophthirius	multifiliis	hypothetical protein IMG5_068710 [Ichthyophthirius multifiliis]
XP_002951476.1	167	VOLCADRAFT_75021	eukaryota>viridiplantae>chlorophyta	Volvox	carteri f. nagariensis	hypothetical protein VOLCADRAFT_75021 [Volvox carteri f. nagariensis]
XP_729407.1	166	PY01623	eukaryota>alveolata>apicomplexa	Plasmodium	yoelii yoelii 17XNL	calcineurin b subunit [Plasmodium yoelii yoelii 17XNL]
NP_001084722.1	166	chp	eukaryota>metazoa>chordata>vertebrata	Xenopus	laevis	uncharacterized protein LOC414686 [Xenopus laevis]
XP_005160183.1	166	zgc:92169	eukaryota>metazoa>chordata>vertebrata>actinopterygii	Danio	rerio	protein phosphatase 3 (formerly 2B), regulatory subunit B, alpha isoform, b isoform X1 [Danio rerio]
XP_647297.1	165	cnbA	eukaryota>amoebozoa>mycetozoa>dictyosteliida	Dictyostelium	discoideum AX4	protein phosphatase 2B [Dictyostelium discoideum AX4]
ELR21740.1	165	ACA1_384760	eukaryota>amoebozoa>acanthamoebidae	Acanthamoeba	castellanii str. Neff	EF hand domain containing protein [Acanthamoeba castellanii str. Neff]
Lhya1000008692	164	Lhya1000008692	eukaryota>fungi>mucoromycotina	Lichtheimia	hyalospora	>Lhya1000008692
ORX94388.1	163	K493DRAFT_324847	eukaryota>fungi	Basidiobolus	meristosporus CBS 931.73	calcineurin subunit B [Basidiobolus meristosporus CBS 931.73]
ORX90655.1	163	K493DRAFT_325826	eukaryota>fungi	Basidiobolus	meristosporus CBS 931.73	calcineurin subunit B [Basidiobolus meristosporus CBS 931.73]
ORX90484.1	163	K493DRAFT_330863	eukaryota>fungi	Basidiobolus	meristosporus CBS 931.73	calcineurin subunit B [Basidiobolus meristosporus CBS 931.73]
XP_021881700.1	163	BCR41DRAFT_377690	eukaryota>fungi	Lobosporangium	transversale	calcineurin subunit B [Lobosporangium transversale]
ORE11212.1	163	BCV72DRAFT_246987	eukaryota>fungi	Rhizopus	microsporus var. microsporus	calcineurin subunit B [Rhizopus microsporus var. microsporus]
EIE91821.1	163	RO3G_16532	eukaryota>fungi	Rhizopus	delemar RA 99-880	calcineurin subunit B [Rhizopus delemar RA 99-880]
EXX59223.1	163	RirG_190820	eukaryota>fungi	Rhizophagus	irregularis DAOM 197198w	Cnb1p [Rhizophagus irregularis DAOM 197198w]
EXX59224.1	163	RirG_190820	eukaryota>fungi	Rhizophagus	irregularis DAOM 197198w	Cnb1p [Rhizophagus irregularis DAOM 197198w]
CBY23841.1	162	GSOID_T00001167001	eukaryota>metazoa>chordata	Oikopleura	dioica	unnamed protein product [Oikopleura dioica]
XP_623751.1	162	LOC551313	eukaryota>metazoa>hexapoda	Apis	mellifera	PREDICTED: calcineurin subunit B type 2 [Apis mellifera]
XP_012356681.1	160	PPP3R1	eukaryota>metazoa>chordata>vertebrata	Nomascus	leucogenys	PREDICTED: calcineurin subunit B type 1 isoform X3 [Nomascus leucogenys]
XP_002109034.1	160	TRIADDRAFT_49810	eukaryota>metazoa>placozoa	Trichoplax	adhaerens	conserved hypothetical protein [Trichoplax adhaerens]
XP_001696107.1	159	CHLREDRAFT_119565	eukaryota>viridiplantae>chlorophyta	Chlamydomonas	reinhardtii	predicted protein [Chlamydomonas reinhardtii]
EPY42954.1	158	AGDE_00969	eukaryota>euglenozoa>kinetoplastida	Angomonas	deanei	protein phosphatase 3, regulatory subunit, partial [Angomonas deanei]
EPZ33845.1	158	O9G_004437	eukaryota>fungi	Rozella	allomycis CSF55	Calcineurin subunit B [Rozella allomycis CSF55]
XP_005765881.1	158	EMIHUDRAFT_66731	eukaryota>haptophyceae	Emiliania	huxleyi CCMP1516	hypothetical protein EMIHUDRAFT_66731, partial [Emiliania huxleyi CCMP1516]
XP_664170.1	154	AN6566.2	eukaryota>fungi>ascomycota	Aspergillus	nidulans FGSC A4	conserved hypothetical protein [Aspergillus nidulans FGSC A4]
XP_006676533.1	153	BATDEDRAFT_9620	eukaryota>fungi>chytridiomycota	Batrachochytrium	dendrobatidis JAM81	hypothetical protein BATDEDRAFT_9620, partial [Batrachochytrium dendrobatidis JAM81]
Smar1000001484	151	Smar1000001484	eukaryota>metazoa>arthropoda>myriapoda	Strigamia	maritima	>Smar1000001484
XP_002774759.1	144	Pmar_PMAR025915	eukaryota>alveolata	Perkinsus	marinus ATCC 50983	protein phosphatase 2B regulatory subunit, putative, partial [Perkinsus marinus ATCC 50983]
EXX59225.1	144	RirG_190820	eukaryota>fungi	Rhizophagus	irregularis DAOM 197198w	Cnb1p [Rhizophagus irregularis DAOM 197198w]
XP_018733872.1	144	CNB1	eukaryota>fungi>ascomycota	Sugiyamaella	lignohabitans	calcineurin regulatory subunit B [Sugiyamaella lignohabitans]
XP_009019630.1	139	HELRODRAFT_126208	eukaryota>metazoa>annelida	Helobdella	robusta	hypothetical protein HELRODRAFT_126208, partial [Helobdella robusta]
KWX14988.1	126	QR46_0966	eukaryota>diplomonadida	Giardia	intestinalis assemblage B	EF hand family protein/calcineurin B subunit [Giardia intestinalis assemblage B]

I. TMX1

XP_019019314.1	739	PICMEDRAFT_58479	eukaryota>fungi>ascomycota	Pichia	membranifaciens NRRL Y-2026	hypothetical protein PICMEDRAFT_58479 [Pichia membranifaciens NRRL Y-2026]
XP_714564.2	737	CAALFM_C209620WA	eukaryota>fungi>ascomycota	Candida	albicans SC5314	protein disulfide isomerase [Candida albicans SC5314]
XP_002615305.1	732	CLUG_04187	eukaryota>fungi>ascomycota	Clavispora	lusitaniae ATCC 42720	hypothetical protein CLUG_04187 [Clavispora lusitaniae ATCC 42720]
XP_453687.1	706	KLLA0_D14014g	eukaryota>fungi>ascomycota	Kluyveromyces	lactis	uncharacterized protein KLLA0_D14014g [Kluyveromyces lactis]
NP_012261.1	701	EPS1	eukaryota>fungi>ascomycota	Saccharomyces	cerevisiae S288C	protein disulfide isomerase EPS1 [Saccharomyces cerevisiae S288C]
XP_019039785.1	637	WICANDRAFT_51146	eukaryota>fungi>ascomycota	Wickerhamomyces	anomalus NRRL Y-366-8	hypothetical protein WICANDRAFT_51146, partial [Wickerhamomyces anomalus NRRL Y-366-8]
XP_011773288.1	589	TbgDal_V1390	eukaryota>euglenozoa>kinetoplastida	Trypanosoma	brucei gambiense DAL972	disulfide isomerase, putative [Trypanosoma brucei gambiense DAL972]
XP_010702546.1	564	LPMP_341200	eukaryota>euglenozoa>kinetoplastida	Leishmania	panamensis	hypothetical protein LPMP_341200 [Leishmania panamensis]
[illegible]						

J. Calreticulin

[illegible]						

OMJ88964.1	384	SteCoe 8973	eukaryota>alveolata>ciliophora	Stentor	coeruleus	hypothetical protein SteCoe_8973 [Stentor coeruleus]
XP_001744647.1	384	MONBRDRAFT_20407	eukaryota>choanoflagellida	Monosiga	brevicollis MX1	hypothetical protein [Monosiga brevicollis MX1]
XP_003275837.1	384	CALR3	eukaryota>metazoa>chordata>vertebrata	Nomascus	leucogenys	PREDICTED: calreticulin-3 [Nomascus leucogenys]
XP 016495583.1	381	LOC107814655	eukaryota>viridiplantae	Nicotiana	tabacum	PREDICTED: calreticulin isoform X2 [Nicotiana tabacum]
NP_082776.2	380	1700031L01Rik,6330586I20Rik,Crt2,cspn	eukaryota>metazoa>chordata>vertebrata	Mus	musculus	calreticulin-3 isoform 1 precursor [Mus musculus]
XP 013397065.1	380	LOC106163893	eukaryota>metazoa	Lingula	anatina	calreticulin-like [Lingula anatina]
XP 001312065.1	376	TVAG 115390	eukaryota>parabasalia	Trichomonas	vaginalis G3	Calreticulin family protein [Trichomonas vaginalis G3]
XP_002291609.1	376	THAPSDRAFT_34810	eukaryota>stramenopiles	Thalassiosira	pseudonana CCMP1335	calreticulin-like protein, partial [Thalassiosira pseudonana CCMP1335]
XP 001300977.1	375	TVAG 207320	eukaryota>parabasalia	Trichomonas	vaginalis G3	Calreticulin family protein [Trichomonas vaginalis G3]
EPZ32318.1	370	O9G 002159	eukaryota>fungi	Rozella	allomycis CSF55	Calreticulin domain-containing protein [Rozella allomycis CSF55]
NP 973793.1	370	A.,thaliana,calreticulin,3,AtCRT3,calreticulin,3,CALRETICULIN,3,EBS2,EMS-MUTAGENIZED,BRI1,SUPPRESSOR,2,PRIORITY,IN,SWEET,LIFE,1,PSL1,T27G7.13,T27G7_13	eukaryota>viridiplantae	Arabidopsis	thaliana	calreticulin 3 [Arabidopsis thaliana]
OLP93827.1	369	AK812 SmicGene24221	eukaryota>alveolata	Symbiodinium	microadriaticum	Calreticulin [Symbiodinium microadriaticum]
XP 001640172.1	366	NEMVEDRAFT_v1g61368	eukaryota>metazoa>cnidaria	Nematostella	vectensis	predicted protein, partial [Nematostella vectensis]
KXJ26714.1	363	Crc	eukaryota>metazoa>cnidaria	Exaiptasia	pallida	Calreticulin [Exaiptasia pallida]
XP 006824896.1	360	LOC100375401	eukaryota>metazoa>hemichordata	Saccoglossus	kowalevskii	PREDICTED: calreticulin-like isoform X2 [Saccoglossus kowalevskii]
Pram1000014320	352	Pram1000014320	eukaryota>stramenopiles	Phytophthora	ramorum	>Pram1000014320
XP_016611705.1	350	SPPG_01136	eukaryota>fungi>chytridiomycota	Spizellomyces	punctatus DAOM BR117	hypothetical protein SPPG_01136 [Spizellomyces punctatus DAOM BR117]
EPY31781.1	343	STCU 03244	eukaryota>euglenozoa>kinetoplastida	Strigomonas	culicis	calreticulin [Strigomonas culicis]
XP 006509824.1	336	1700031L01Rik,6330586I20Rik,Crt2,cspn	eukaryota>metazoa>chordata>vertebrata	Mus	musculus	PREDICTED: calreticulin-3 isoform X1 [Mus musculus]
Smed1000027960	331	Smed1000027960	eukaryota>metazoa>platyhelminthe	Schmidtea	mediterranea	>Smed1000027960
XP 001734007.1	318	EDI 348940	eukaryota>amoebozoa>entamoebidae	Entamoeba	dispar SAW760	Calreticulin precursor [Entamoeba dispar SAW760]
NP 001272825.1	313	4933403L16Rik,AU017583	eukaryota>metazoa>chordata>vertebrata	Mus	musculus	calreticulin 4 isoform c [Mus musculus]
NP_001272826.1	313	4933403L16Rik,AU017583	eukaryota>metazoa>chordata>vertebrata	Mus	musculus	calreticulin 4 isoform c [Mus musculus]
NP_001272827.1	313	4933403L16Rik,AU017583	eukaryota>metazoa>chordata>vertebrata	Mus	musculus	calreticulin 4 isoform c [Mus musculus]
XP_017175400.1	313	4933403L16Rik,AU017583	eukaryota>metazoa>chordata>vertebrata	Mus	musculus	PREDICTED: calreticulin 4 isoform X1 [Mus musculus]
XP 009038526.1	311	AURANDRAFT 65433	eukaryota>stramenopiles	Aureococcus	anophagefferens	expressed protein [Aureococcus anophagefferens]
CDW86807.1	298	Contig8228.g8773	eukaryota>alveolata>ciliophora	Stylonychia	lemnae	calreticulin precursor [Stylonychia lemnae]
XP_018115337.1	275	LOC108714990	eukaryota>metazoa>chordata>vertebrata	Xenopus	laevis	PREDICTED: calreticulin-like [Xenopus laevis]
NP 084058.2	272	1700031L01Rik,6330586I20Rik,Crt2,cspn	eukaryota>metazoa>chordata>vertebrata	Mus	musculus	calreticulin-3 isoform 2 [Mus musculus]
XP 013892903.1	254	MNEG 14079	eukaryota>viridiplantae>chlorophyta	Monoraphidium	neglectum	Calreticulin Flags: Precursor [Monoraphidium neglectum]
XP_002900130.1	235	PITG_12738	eukaryota>stramenopiles	Phytophthora	infestans T30-4	conserved hypothetical protein [Phytophthora infestans T30-4]
XP_002769367.1	224	Pmar PMAR004240	eukaryota>alveolata	Perkinsus	marinus ATCC 50983	Calreticulin precursor, putative, partial [Perkinsus marinus ATCC 50983]
ETO20325.1	215	RFI 16892	eukaryota>rhizaria	Reticulomyxa	filosa	Calreticulin precursor, partial [Reticulomyxa filosa]
Smed1000013190	159	Smed1000013190	eukaryota>metazoa>platyhelminthe	Schmidtea	mediterranea	>Smed1000013190

K. Calnexin

XP 006453987.1	1485	AGABI2DRAFT_181983	eukaryota>fungi>basidiomycota	Agaricus	bisporus var. bisporus H97	hypothetical protein AGABI2DRAFT_181983 [Agaricus bisporus var. bisporus H97]
OWA53125.1	1248	BV898 17558	eukaryota>metazoa	Hypsibius	dujardini	Calnexin [Hypsibius dujardini]
XP_002674608.1	1118	NAEGRDRAFT_70209	eukaryota>heterolobosea	Naegleria	gruberi strain NEG-M	predicted protein [Naegleria gruberi strain NEG-M]
XP 011618359.1	718	clgn	eukaryota>metazoa>chordata>vertebrata>actinopterygii	Takifugu	rubripes	PREDICTED: calmegin [Takifugu rubripes]
XP 005536371.1	660	CYME_CMI213C	eukaryota>rhodophyta	Cyanidioschyzon	merolae strain 10D	calnexin [Cyanidioschyzon merolae strain 10D]
XP_004940998.1	655	CLGN	eukaryota>metazoa>chordata>vertebrata	Gallus	gallus	PREDICTED: calmegin isoform X1 [Gallus gallus]
Chet1000006749	655	Chet1000006749	eukaryota>fungi>ascomycota	Cochliobolus	heterostrophus	>Chet1000006749
XP_004940999.1	652	CLGN	eukaryota>metazoa>chordata>vertebrata	Gallus	gallus	calmegin isoform X2 [Gallus gallus]
XP_018087171.1	647	LOC108699598	eukaryota>metazoa>chordata>vertebrata	Xenopus	laevis	PREDICTED: calmegin-like isoform X1 [Xenopus laevis]
XP_018087179.1	647	LOC108699598	eukaryota>metazoa>chordata>vertebrata	Xenopus	laevis	PREDICTED: calmegin-like isoform X1 [Xenopus laevis]
XP_011532966.1	646	CNX,IP90,P90	eukaryota>metazoa>chordata>vertebrata	Homo	sapiens	calnexin isoform X3 [Homo sapiens]
XP 004941000.1	644	CLGN	eukaryota>metazoa>chordata>vertebrata	Gallus	gallus	calmegin isoform X3 [Gallus gallus]
XP 011132126.1	643	GNI 129870	eukaryota>alveolata>apicomplexa	Gregarina	niphandrodes	calreticulin family protein [Gregarina niphandrodes]
XP_013232333.1	640	ETH_00022665	eukaryota>alveolata>apicomplexa	Eimeria	tenella	calnexin, putative [Eimeria tenella]
NP 573131.1	639	CG9906,Cnx,Dmel\CG9906,Xcbp1	eukaryota>metazoa>hexapoda	Drosophila	melanogaster	calnexin 14D [Drosophila melanogaster]
XP 017319913.1	634	clgn	eukaryota>metazoa>chordata>vertebrata>actinopterygii	Ictalurus	punctatus	PREDICTED: calmegin isoform X1 [Ictalurus punctatus]
XP 017319914.1	634	clgn	eukaryota>metazoa>chordata>vertebrata>actinopterygii	Ictalurus	punctatus	PREDICTED: calmegin isoform X1 [Ictalurus punctatus]
XP 017319915.1	634	clgn	eukaryota>metazoa>chordata>vertebrata>actinopterygii	Ictalurus	punctatus	PREDICTED: calmegin isoform X1 [Ictalurus punctatus]
XP 017319916.1	633	clgn	eukaryota>metazoa>chordata>vertebrata>actinopterygii	Ictalurus	punctatus	PREDICTED: calmegin isoform X2 [Ictalurus punctatus]
NP_001071009.1	630	canx,wu:fj24b04,zgc:153946	eukaryota>metazoa>chordata>vertebrata>actinopterygii	Danio	rerio	calmegin precursor [Danio rerio]

[illegible]

XP_014771603.1	679	LOC106870127	eukaryota>metazoa>mollusca	Octopus	bimaculoides	PREDICTED: putative uncharacterized protein DDB_G0282499, partial [Octopus bimaculoides]
XP_019700160.1	678	LOC105190090	eukaryota>metazoa>hexapoda	Harpegnathos	saltator	PREDICTED: protein enabled [Harpegnathos saltator]
XP_012552943.1	672	LOC101735452	eukaryota>metazoa>hexapoda	Bombyx	mori	protein enabled isoform X1 [Bombyx mori]
XP_021209110.1	672	LOC101735452	eukaryota>metazoa>hexapoda	Bombyx	mori	protein enabled isoform X1 [Bombyx mori]
XP_021209111.1	672	LOC101735452	eukaryota>metazoa>hexapoda	Bombyx	mori	protein enabled isoform X1 [Bombyx mori]
XP_021209112.1	671	LOC101735452	eukaryota>metazoa>hexapoda	Bombyx	mori	protein enabled isoform X2 [Bombyx mori]
XP_021209113.1	671	LOC101735452	eukaryota>metazoa>hexapoda	Bombyx	mori	protein enabled isoform X3 [Bombyx mori]
XP_021209114.1	670	LOC101735452	eukaryota>metazoa>hexapoda	Bombyx	mori	protein enabled isoform X4 [Bombyx mori]
XP_014533405.1	666	PDIP_61790	eukaryota>fungi>ascomycota	Penicillium	digitatum Pd1	Proline-rich protein LAS17 [Penicillium digitatum Pd1]
NP_001286602.1	664	CG15112,Dmel\CG15112,Ena,ENA/VASP,enb,Enb,l(2)02029,Vasp,VASP	eukaryota>metazoa>hexapoda	Drosophila	melanogaster	enabled, isoform G [Drosophila melanogaster]
XP_005158849.1	663	Evl,id:ibd2260,zgc:65883,zgc:77388	eukaryota>metazoa>chordata>vertebrata>actinopterygii	Danio	rerio	ena/VASP-like protein isoform X1 [Danio rerio]
ERG80807.1	663	ASU_11206	eukaryota>metazoa>nematoda	Ascaris	suum	sprouty- evh1 domain-containing protein 2 [Ascaris suum]
XP_021322898.1	662	Evl,id:ibd2260,zgc:65883,zgc:77388	eukaryota>metazoa>chordata>vertebrata>actinopterygii	Danio	rerio	ena/VASP-like protein isoform X2 [Danio rerio]
XP_005158850.1	653	Evl,id:ibd2260,zgc:65883,zgc:77388	eukaryota>metazoa>chordata>vertebrata>actinopterygii	Danio	rerio	ena/VASP-like protein isoform X3 [Danio rerio]
XP_452412.1	653	KLLA0_C04774g	eukaryota>fungi>ascomycota	Kluyveromyces	lactis	uncharacterized protein KLLA0_C04774g [Kluyveromyces lactis]
XP_012749022.1	651	SAMD00019534_112390	eukaryota>amoebozoa>mycetozoa>dictyosteliida	Acytostelium	subglobosum LB1	hypothetical protein SAMD00019534_112390 [Acytostelium subglobosum LB1]
XP_015306083.1	650	ENAH	eukaryota>metazoa>chordata>vertebrata	Macaca	fascicularis	PREDICTED: protein enabled homolog isoform X11 [Macaca fascicularis]
EFA85844.1	650	wasA	eukaryota>amoebozoa>mycetozoa	Heterostelium	album PN500	Wiscott-Aldrich syndrome protein [Heterostelium album PN500]
XP_018655478.1	650	Smp_039870.1	eukaryota>metazoa	Schistosoma	mansoni	putative vasodilator-stimulated phosphoprotein [Schistosoma mansoni]
XP_019019119.1	644	PICMEDRAFT_15858	eukaryota>fungi>ascomycota	Pichia	membranifaciens NRRL Y-2026	hypothetical protein PICMEDRAFT_15858 [Pichia membranifaciens NRRL Y-2026]
XP_005158851.1	641	Evl,id:ibd2260,zgc:65883,zgc:77388	eukaryota>metazoa>chordata>vertebrata>actinopterygii	Danio	rerio	ena/VASP-like protein isoform X4 [Danio rerio]
EPZ34321.1	641	O9G_000508	eukaryota>fungi	Rozella	allomycis CSF55	Chitin synthase III catalytic subunit domain-containing protein [Rozella allomycis CSF55]
XP_005158852.1	638	Evl,id:ibd2260,zgc:65883,zgc:77388	eukaryota>metazoa>chordata>vertebrata>actinopterygii	Danio	rerio	ena/VASP-like protein isoform X5 [Danio rerio]
XP_964625.1	636	NCU07438	eukaryota>fungi>ascomycota	Neurospora	crassa OR74A	WASP-like protein las17p [Neurospora crassa OR74A]
XP_017323611.1	635	LOC108265595	eukaryota>metazoa>chordata>vertebrata>actinopterygii	Ictalurus	punctatus	PREDICTED: wiskott-Aldrich syndrome protein-like isoform X1 [Ictalurus punctatus]
NP_014824.1	633	BEE1	eukaryota>fungi>ascomycota	Saccharomyces	cerevisiae S288C	actin-binding protein LAS17 [Saccharomyces cerevisiae S288C]
XP_005541041.1	631	ENAH	eukaryota>metazoa>chordata>vertebrata	Macaca	fascicularis	PREDICTED: protein enabled homolog isoform X12 [Macaca fascicularis]
XP_699919.5	630	wasa	eukaryota>metazoa>chordata>vertebrata>actinopterygii	Danio	rerio	wiskott-Aldrich syndrome protein [Danio rerio]
XP_015306085.1	629	ENAH	eukaryota>metazoa>chordata>vertebrata	Macaca	fascicularis	PREDICTED: protein enabled homolog isoform X13 [Macaca fascicularis]
XP_501207.2	629	YALI0_B22110g	eukaryota>fungi>ascomycota	Yarrowia	lipolytica CLIB122	YALI0B22110p [Yarrowia lipolytica CLIB122]
XP_016857242.1	628	ENA,MENA,NDPP1	eukaryota>metazoa>chordata>vertebrata	Homo	sapiens	PREDICTED: protein enabled homolog isoform X9 [Homo sapiens]
ORY25113.1	628	LY90DRAFT_674938	eukaryota>fungi>chytridiomycota	Neocallimastix	californiae	hypothetical protein LY90DRAFT_674938 [Neocallimastix californiae]
XP_018186371.1	620	L228DRAFT_249631	eukaryota>fungi>ascomycota	Xylona	heveae TC161	WH1-domain-containing protein [Xylona heveae TC161]
XP_002841323.1	618	GSTUM_00010426001	eukaryota>fungi>ascomycota	Tuber	melanosporum Mel28	hypothetical protein [Tuber melanosporum Mel28]
XP_021324089.1	616	id:ibd2798,si:ch211-215p14.1,zgc:103638	eukaryota>metazoa>chordata>vertebrata>actinopterygii	Danio	rerio	protein enabled homolog isoform X5 [Danio rerio]
XP_016857243.1	614	ENA,MENA,NDPP1	eukaryota>metazoa>chordata>vertebrata	Homo	sapiens	PREDICTED: protein enabled homolog isoform X10 [Homo sapiens]
XP_002111223.1	611	TRIADDRAFT_54962	eukaryota>metazoa>placozoa	Trichoplax	adhaerens	hypothetical protein TRIADDRAFT_54962 [Trichoplax adhaerens]
XP_005541042.1	610	ENAH	eukaryota>metazoa>chordata>vertebrata	Macaca	fascicularis	PREDICTED: protein enabled homolog isoform X14 [Macaca fascicularis]
XP_017332339.1	610	LOC108270303	eukaryota>metazoa>chordata>vertebrata>actinopterygii	Ictalurus	punctatus	PREDICTED: ena/VASP-like protein isoform X1 [Ictalurus punctatus]
XP_011125450.1	610	AOL_s00112g118	eukaryota>fungi>ascomycota	Arthrobotrys	oligospora ATCC 24927	hypothetical protein AOL_s00112g118 [Arthrobotrys oligospora ATCC 24927]
XP_017332340.1	609	LOC108270303	eukaryota>metazoa>chordata>vertebrata>actinopterygii	Ictalurus	punctatus	PREDICTED: ena/VASP-like protein isoform X2 [Ictalurus punctatus]
XP_017332341.1	609	LOC108270303	eukaryota>metazoa>chordata>vertebrata>actinopterygii	Ictalurus	punctatus	PREDICTED: ena/VASP-like protein isoform X3 [Ictalurus punctatus]
EUB60407.1	607	EGR_04789	eukaryota>metazoa	Echinococcus	granulosus	Protein enabled [Echinococcus granulosus]
XP_005273244.1	605	ENA,MENA,NDPP1	eukaryota>metazoa>chordata>vertebrata	Homo	sapiens	PREDICTED: protein enabled homolog isoform X11 [Homo sapiens]
XP_018119925.1	602	Xena	eukaryota>metazoa>chordata>vertebrata	Xenopus	laevis	PREDICTED: enabled protein isoform X2 [Xenopus laevis]
XP_016857244.1	601	ENA,MENA,NDPP1	eukaryota>metazoa>chordata>vertebrata	Homo	sapiens	PREDICTED: protein enabled homolog isoform X12 [Homo sapiens]
XP_015139086.1	598	ENAH	eukaryota>metazoa>chordata>vertebrata	Gallus	gallus	PREDICTED: protein enabled homolog isoform X11 [Gallus gallus]
XP_021324090.1	596	id:ibd2798,si:ch211-215p14.1,zgc:103638	eukaryota>metazoa>chordata>vertebrata>actinopterygii	Danio	rerio	protein enabled homolog isoform X6 [Danio rerio]
XP_015139087.1	594	ENAH	eukaryota>metazoa>chordata>vertebrata	Gallus	gallus	protein enabled homolog isoform X8 [Gallus gallus]
XP_006496713.1	593	Mena,NDPP-1,Ndpp1,WBP8	eukaryota>metazoa>chordata>vertebrata	Mus	musculus	PREDICTED: protein enabled homolog isoform X8 [Mus musculus]
XP_001222599.1	592	CHGG_06504	eukaryota>fungi>ascomycota	Chaetomium	globosum CBS 148.51	hypothetical protein CHGG_06504 [Chaetomium globosum CBS 148.51]
NP_001008493.1	591	ENA,MENA,NDPP1	eukaryota>metazoa>chordata>vertebrata	Homo	sapiens	protein enabled homolog isoform a [Homo sapiens]
XP_016885275.1	590	IMD2,SCNX,THC,THC1,WASP,WASPA	eukaryota>metazoa>chordata>vertebrata	Homo	sapiens	wiskott-Aldrich syndrome protein isoform X2 [Homo sapiens]
XP_017323612.1	590	LOC108265595	eukaryota>metazoa>chordata>vertebrata>actinopterygii	Ictalurus	punctatus	PREDICTED: wiskott-Aldrich syndrome protein-like isoform X2 [Ictalurus punctatus]
XP_015306089.1	589	ENAH	eukaryota>metazoa>chordata>vertebrata	Macaca	fascicularis	PREDICTED: protein enabled homolog isoform X15 [Macaca fascicularis]
XP_015306090.1	589	ENAH	eukaryota>metazoa>chordata>vertebrata	Macaca	fascicularis	PREDICTED: protein enabled homolog isoform X15 [Macaca fascicularis]
XP_009013906.1	586	HELRODRAFT_98248	eukaryota>metazoa>annelida	Helobdella	robusta	hypothetical protein HELRODRAFT_98248 [Helobdella robusta]
XP_011419448.1	586	LOC105322431	eukaryota>metazoa>mollusca	Crassostrea	gigas	PREDICTED: homer protein homolog 2 isoform X1 [Crassostrea gigas]
CDS37344.1	586	EmuJ_000458800	eukaryota>metazoa	Echinococcus	multilocularis	vasodilator stimulated phosphoprotein [Echinococcus multilocularis]
XP_018655479.1	585	Smp_039870.1	eukaryota>metazoa	Schistosoma	mansoni	LOW QUALITY PROTEIN: putative vasodilator-stimulated phosphoprotein [Schistosoma mansoni]
XP_002668568.1	582	NAEGRDRAFT_82342	eukaryota>heterolobosea	Naegleria	gruberi strain NEG-M	predicted protein [Naegleria gruberi strain NEG-M]
XP_009292990.1	581	id:ibd2798,si:ch211-215p14.1,zgc:103638	eukaryota>metazoa>chordata>vertebrata>actinopterygii	Danio	rerio	protein enabled homolog isoform X7 [Danio rerio]
XP_011419449.1	581	LOC105322431	eukaryota>metazoa>mollusca	Crassostrea	gigas	PREDICTED: homer protein homolog 2 isoform X2 [Crassostrea gigas]
XP_003275128.1	579	ENAH	eukaryota>metazoa>chordata>vertebrata	Nomascus	leucogenys	PREDICTED: protein enabled homolog [Nomascus leucogenys]
XP_016857245.1	578	ENA,MENA,NDPP1	eukaryota>metazoa>chordata>vertebrata	Homo	sapiens	PREDICTED: protein enabled homolog isoform X13 [Homo sapiens]
XP_016857246.1	578	ENA,MENA,NDPP1	eukaryota>metazoa>chordata>vertebrata	Homo	sapiens	PREDICTED: protein enabled homolog isoform X13 [Homo sapiens]
EFX90289.1	578	DAPPUDRAFT_300115	eukaryota>metazoa>crustacea	Daphnia	pulex	hypothetical protein DAPPUDRAFT_300115 [Daphnia pulex]
XP_013427246.1	577	M436DRAFT_73051	eukaryota>fungi>ascomycota	Aureobasidium	namibiae CBS 147.97	WH1-domain-containing protein [Aureobasidium namibiae CBS 147.97]
XP_001897341.1	575	Bm1_29495	eukaryota>metazoa>nematoda	Brugia	malayi	WH1 domain containing protein [Brugia malayi]
XP_001631290.1	575	NEMVEDRAFT_v1g244007	eukaryota>metazoa>cnidaria	Nematostella	vectensis	predicted protein [Nematostella vectensis]
XP_015139088.1	574	ENAH	eukaryota>metazoa>chordata>vertebrata	Gallus	gallus	protein enabled homolog isoform X9 [Gallus gallus]
NP_594758.1	574	wsp1	eukaryota>fungi>ascomycota	Schizosaccharomyces	pombe	actin assembly factor [Schizosaccharomyces pombe]
XP_006496714.1	572	Mena,NDPP-1,Ndpp1,WBP8	eukaryota>metazoa>chordata>vertebrata	Mus	musculus	PREDICTED: protein enabled homolog isoform X9 [Mus musculus]
XP_018119926.1	572	Xena	eukaryota>metazoa>chordata>vertebrata	Xenopus	laevis	PREDICTED: enabled protein isoform X3 [Xenopus laevis]
NP_060682.2	570	ENA,MENA,NDPP1	eukaryota>metazoa>chordata>vertebrata	Homo	sapiens	protein enabled homolog isoform b [Homo sapiens]
XP_017311958.1	569	enah	eukaryota>metazoa>chordata>vertebrata>actinopterygii	Ictalurus	punctatus	PREDICTED: protein enabled homolog isoform X6 [Ictalurus punctatus]
CEO99970.1	569	PBRA_007704	eukaryota>rhizaria>cercozoa>plasmodiophorida	Plasmodiophora	brassicae	hypothetical protein PBRA_007704 [Plasmodiophora brassicae]
CAG10529.1	567	GSTEN:00032207:G:001	eukaryota>metazoa>chordata>vertebrata>actinopterygii	Tetraodon	nigroviridis	unnamed protein product, partial [Tetraodon nigroviridis]
XP_015306091.1	566	ENAH	eukaryota>metazoa>chordata>vertebrata	Macaca	fascicularis	PREDICTED: protein enabled homolog isoform X16 [Macaca fascicularis]
XP_014662305.1	566	LOC106805282	eukaryota>metazoa	Priapulus	caudatus	PREDICTED: neural Wiskott-Aldrich syndrome protein-like [Priapulus caudatus]
XP_019027289.1	565	SAICODRAFT_4370	eukaryota>fungi>ascomycota	Saitoella	complicata NRRL Y-17804	WH1-domain-containing protein [Saitoella complicata NRRL Y-17804]
NP_001085660.1	562	Ena,enah,Xena	eukaryota>metazoa>chordata>vertebrata	Xenopus	laevis	ENAH, actin regulator L homeolog [Xenopus laevis]
XP_002609962.1	561	BRAFLDRAFT_85919	eukaryota>metazoa>chordata	Branchiostoma	floridae	hypothetical protein BRAFLDRAFT_85919 [Branchiostoma floridae]
XP_015139090.1	561	ENAH	eukaryota>metazoa>chordata>vertebrata	Gallus	gallus	PREDICTED: protein enabled homolog isoform X14 [Gallus gallus]
XP_009292991.1	561	id:ibd2798,si:ch211-215p14.1,zgc:103638	eukaryota>metazoa>chordata>vertebrata>actinopterygii	Danio	rerio	protein enabled homolog isoform X8 [Danio rerio]
OQV12129.1	559	BV898_13608	eukaryota>metazoa	Hypsibius	dujardini	putative Protein enabled-like protein [Hypsibius dujardini]
XP_003380270.1	557	Tsp_06005	eukaryota>metazoa>nematoda	Trichinella	spiralis	putative neural Wiskott-Aldrich syndrome protein [Trichinella spiralis]
ORZ38628.1	553	BCR44DRAFT_1428628	eukaryota>fungi	Catenaria	anguillulae PL171	hypothetical protein BCR44DRAFT_1428628, partial [Catenaria anguillulae PL171]
XP_011419450.1	551	LOC105322431	eukaryota>metazoa>mollusca	Crassostrea	gigas	PREDICTED: homer protein homolog 2 isoform X3 [Crassostrea gigas]
XP_015139091.1	550	ENAH	eukaryota>metazoa>chordata>vertebrata	Gallus	gallus	protein enabled homolog isoform X11 [Gallus gallus]
XP_001835070.1	550	CC1G_06473	eukaryota>fungi>basidiomycota	Coprinopsis	cinerea okayama7#130	hypothetical protein CC1G_06473 [Coprinopsis cinerea okayama7#130]
OWA55093.1	550	BV898_19478	eukaryota>metazoa	Hypsibius	dujardini	putative Sprouty-related, EVH1 domain-containing protein 1 [Hypsibius dujardini]
XP_017311959.1	549	enah	eukaryota>metazoa>chordata>vertebrata>actinopterygii	Ictalurus	punctatus	PREDICTED: protein enabled homolog isoform X7 [Ictalurus punctatus]
KFH74147.1	549	MVEG_01360	eukaryota>fungi	Mortierella	verticillata NRRL 6337	hypothetical protein MVEG_01360 [Mortierella verticillata NRRL 6337]
ORY53338.1	548	LY90DRAFT_285267	eukaryota>fungi>chytridiomycota	Neocallimastix	californiae	hypothetical protein LY90DRAFT_285267 [Neocallimastix californiae]
CBY09705.1	545	GSOID_T00010515001	eukaryota>metazoa>chordata	Oikopleura	dioica	unnamed protein product [Oikopleura dioica]
XP_011601021.1	544	was	eukaryota>metazoa>chordata>vertebrata>actinopterygii	Takifugu	rubripes	PREDICTED: wiskott-Aldrich syndrome protein [Takifugu rubripes]
CBY22289.1	544	GSOID_T00011846001	eukaryota>metazoa>chordata	Oikopleura	dioica	unnamed protein product [Oikopleura dioica]
CBY31400.1	544	GSOID_T00025303001	eukaryota>metazoa>chordata	Oikopleura	dioica	unnamed protein product [Oikopleura dioica]
NP_001076590.1	541	Mena,NDPP-1,Ndpp1,WBP8	eukaryota>metazoa>chordata>vertebrata	Mus	musculus	protein enabled homolog isoform 4 [Mus musculus]
XP_021876053.1	541	BCR41DRAFT_426279	eukaryota>fungi	Lobosporangium	transversale	hypothetical protein BCR41DRAFT_426279 [Lobosporangium transversale]
XP_021323362.1	540	si:ch211-255112.2	eukaryota>metazoa>chordata>vertebrata>actinopterygii	Danio	rerio	sprouty-related, EVH1 domain containing 3 isoform X1 [Danio rerio]
XP_006569628.1	540	LOC409218	eukaryota>metazoa>hexapoda	Apis	mellifera	PREDICTED: neural Wiskott-Aldrich syndrome protein [Apis mellifera]
XP_392742.4	540	LOC409218	eukaryota>metazoa>hexapoda	Apis	mellifera	PREDICTED: neural Wiskott-Aldrich syndrome protein [Apis mellifera]
XP_006957313.1	538	WALSEDRAFT_56819	eukaryota>fungi>basidiomycota	Wallemia	mellicola CBS 633.66	hypothetical protein WALSEDRAFT_56819 [Wallemia mellicola CBS 633.66]
KXJ29816.1	538	WAS	eukaryota>metazoa>cnidaria	Exaiptasia	pallida	Wiskott-Aldrich syndrome protein [Exaiptasia pallida]
XP_015778704.1	538	LOC107356613	eukaryota>metazoa>cnidaria	Acropora	digitifera	PREDICTED: neural Wiskott-Aldrich syndrome protein-like [Acropora digitifera]
XP_015763982.1	538	LOC107342971	eukaryota>metazoa>cnidaria	Acropora	digitifera	PREDICTED: wiskott-Aldrich syndrome protein homolog [Acropora digitifera]
XP_015139092.1	537	ENAH	eukaryota>metazoa>chordata>vertebrata	Gallus	gallus	protein enabled homolog isoform X12 [Gallus gallus]
XP_002610296.1	536	BRAFLDRAFT_126860	eukaryota>metazoa>chordata	Branchiostoma	floridae	hypothetical protein BRAFLDRAFT_126860 [Branchiostoma floridae]
XP_006496715.1	535	Mena,NDPP-1,Ndpp1,WBP8	eukaryota>metazoa>chordata>vertebrata	Mus	musculus	PREDICTED: protein enabled homolog isoform X10 [Mus musculus]
NP_001086441.1	535	ena,Xena	eukaryota>metazoa>chordata>vertebrata	Xenopus	laevis	ENAH, actin regulator S homeolog [Xenopus laevis]
XP_002127752.1	535	LOC100183769	eukaryota>metazoa>chordata	Ciona	intestinalis	PREDICTED: sprouty-related, EVH1 domain-containing protein 2-like [Ciona intestinalis]
XP_001635928.1	534	NEMVEDRAFT_v1g241376	eukaryota>metazoa>cnidaria	Nematostella	vectensis	predicted protein [Nematostella vectensis]
NP_001013539.2	533	id:ibd2798,si:ch211-215p14.1,zgc:103638	eukaryota>metazoa>chordata>vertebrata>actinopterygii	Danio	rerio	protein enabled homolog isoform 1 [Danio rerio]
NP_001036341.2	531	BEST:LD03829,CG11324,dhom,Dhom,Dhomer,Dm,Homer,Dmel\CG11324,Dvh,Hom,Homer,LD03829	eukaryota>metazoa>hexapoda	Drosophila	melanogaster	homer, isoform E [Drosophila melanogaster]
KXN66161.1	529	CONCODRAFT_80577	eukaryota>fungi	Conidiobolus	coronatus NRRL 28638	hypothetical protein CONCODRAFT_80577 [Conidiobolus coronatus NRRL 28638]
OQV18372.1	528	BV898_07575	eukaryota>metazoa	Hypsibius	dujardini	putative Wiskott-Aldrich syndrome protein [Hypsibius dujardini]
XP_001868297.1	528	CpipJ_CPIJ018200	eukaryota>metazoa>hexapoda	Culex	quinquefasciatus	conserved hypothetical protein [Culex quinquefasciatus]
XP_007331884.1	527	AGABI1DRAFT_101672	eukaryota>fungi>basidiomycota	Agaricus	bisporus var. burnettii JB137-S8	hypothetical protein AGABI1DRAFT_101672 [Agaricus bisporus var. burnettii JB137-S8]
XP_014149294.1	527	SARC_12081	eukaryota>ichthyosporea	Sphaeroforma	arctica JP610	hypothetical protein SARC_12081, partial [Sphaeroforma arctica JP610]
NP_001263045.1	527	CG1520,D-WASP,Dm,WASP,Dmel\CG1520,DmWASP,dWASP,wasp,Wasp,WASP,wsp,Wsp,WSP	eukaryota>metazoa>hexapoda	Drosophila	melanogaster	WASp, isoform D [Drosophila melanogaster]
NP_651637.1	527	CG1520,D-WASP,Dm,WASP,Dmel\CG1520,DmWASP,dWASP,wasp,Wasp,WASP,wsp,Wsp,WSP	eukaryota>metazoa>hexapoda	Drosophila	melanogaster	WASp, isoform A [Drosophila melanogaster]
NP_733257.1	527	CG1520,D-WASP,Dm,WASP,Dmel\CG1520,DmWASP,dWASP,wasp,Wasp,WASP,wsp,Wsp,WSP	eukaryota>metazoa>hexapoda	Drosophila	melanogaster	WASp, isoform B [Drosophila melanogaster]
XP_011137229.1	526	LOC105181870	eukaryota>metazoa>hexapoda	Harpegnathos	saltator	neural Wiskott-Aldrich syndrome protein [Harpegnathos saltator]
XP_011137230.1	526	LOC105181870	eukaryota>metazoa>hexapoda	Harpegnathos	saltator	neural Wiskott-Aldrich syndrome protein [Harpegnathos saltator]
XP_011137231.1	526	LOC105181870	eukaryota>metazoa>hexapoda	Harpegnathos	saltator	neural Wiskott-Aldrich syndrome protein [Harpegnathos saltator]
XP_011137232.1	526	LOC105181870	eukaryota>metazoa>hexapoda	Harpegnathos	saltator	neural Wiskott-Aldrich syndrome protein [Harpegnathos saltator]
XP_011616938.1	525	enah	eukaryota>metazoa>chordata>vertebrata>actinopterygii	Takifugu	rubripes	PREDICTED: protein enabled homolog [Takifugu rubripes]
XP_011661587.1	525	LOC592391	eukaryota>metazoa>echinodermata	Strongylocentrotus	purpuratus	PREDICTED: neural Wiskott-Aldrich syndrome protein isoform X1 [Strongylocentrotus purpuratus]
XP_004349458.1	521	CAOG_02708	eukaryota>ichthyosporea	Capsaspora	owczarzaki ATCC 30864	hypothetical protein CAOG_02708 [Capsaspora owczarzaki ATCC 30864]
OQV18373.1	520	BV898_07576	eukaryota>metazoa	Hypsibius	dujardini	putative Wiskott-Aldrich syndrome protein [Hypsibius dujardini]
NP_033541.1	520	U42471,Wasp	eukaryota>metazoa>chordata>vertebrata	Mus	musculus	wiskott-Aldrich syndrome protein homolog [Mus musculus]
XP_022244554.1	519	LOC106461679	eukaryota>metazoa	Limulus	polyphemus	sprouty-related, EVH1 domain-containing protein 2-like isoform X1 [Limulus polyphemus]
NP_001260979.1	519	AE33,CG10155,Dm,AE33,Dmel\CG10155,dSPRED,spred	eukaryota>metazoa>hexapoda	Drosophila	melanogaster	Sprouty-related protein with EVH-1 domain, isoform C [Drosophila melanogaster]
ORY57069.1	519	LY90DRAFT_669265	eukaryota>fungi>chytridiomycota	Neocallimastix	californiae	WH1-domain-containing protein [Neocallimastix californiae]
XP_017341530.1	519	LOC108275301	eukaryota>metazoa>chordata>vertebrata>actinopterygii	Ictalurus	punctatus	PREDICTED: neural Wiskott-Aldrich syndrome protein-like isoform X1 [Ictalurus punctatus]
XP_017320784.1	518	LOC108264009	eukaryota>metazoa>chordata>vertebrata>actinopterygii	Ictalurus	punctatus	PREDICTED: sprouty-related, EVH1 domain-containing protein 1-like [Ictalurus punctatus]
NP_957353.2	518	wasl,zgc:55478,zgc:77237	eukaryota>metazoa>chordata>vertebrata>actinopterygii	Danio	rerio	Wiskott-Aldrich syndrome-like [Danio rerio]
XP_009497032.1	518	H696_04893	eukaryota	Fonticula	alba	hypothetical protein H696_04893 [Fonticula alba]
XP_011605399.1	517	wasl	eukaryota>metazoa>chordata>vertebrata>actinopterygii	Takifugu	rubripes	PREDICTED: neural Wiskott-Aldrich syndrome protein isoform X1 [Takifugu rubripes]
XP_004990404.1	516	PTSG_08614	eukaryota>choanoflagellida	Salpingoeca	rosetta	hypothetical protein PTSG_08614 [Salpingoeca rosetta]
XP_011605400.1	514	wasl	eukaryota>metazoa>chordata>vertebrata>actinopterygii	Takifugu	rubripes	PREDICTED: neural Wiskott-Aldrich syndrome protein isoform X2 [Takifugu rubripes]
XP_011611713.1	514	LOC101069458	eukaryota>metazoa>chordata>vertebrata>actinopterygii	Takifugu	rubripes	PREDICTED: neural Wiskott-Aldrich syndrome protein-like isoform X1 [Takifugu rubripes]
XP_015139093.1	513	ENAH	eukaryota>metazoa>chordata>vertebrata	Gallus	gallus	protein enabled homolog isoform X13 [Gallus gallus]
KFH64279.1	513	MVEG_10104	eukaryota>fungi	Mortierella	verticillata NRRL 6337	hypothetical protein MVEG_10104 [Mortierella verticillata NRRL 6337]
XP_009170364.1	513	T265_06755	eukaryota>metazoa	Opisthorchis	viverrini	hypothetical protein T265_06755 [Opisthorchis viverrini]
XP_017341531.1	513	LOC108275301	eukaryota>metazoa>chordata>vertebrata>actinopterygii	Ictalurus	punctatus	PREDICTED: neural Wiskott-Aldrich syndrome protein-like isoform X2 [Ictalurus punctatus]
XP_006456048.1	512	AGABI2DRAFT_153764	eukaryota>fungi>basidiomycota	Agaricus	bisporus var. bisporus H97	hypothetical protein AGABI2DRAFT_153764 [Agaricus bisporus var. bisporus H97]
NP_001084852.1	512	n-wasp,nwasp,wasl	eukaryota>metazoa>chordata>vertebrata	Xenopus	laevis	Wiskott-Aldrich syndrome like L homeolog [Xenopus laevis]
XP_022256136.1	511	LOC106471824	eukaryota>metazoa	Limulus	polyphemus	vasodilator-stimulated phosphoprotein-like isoform X1 [Limulus polyphemus]
XP_011677535.1	511	LOC576114	eukaryota>metazoa>echinodermata	Strongylocentrotus	purpuratus	PREDICTED: protein enabled homolog isoform X5 [Strongylocentrotus purpuratus]
XP_021885162.1	511	BCR41DRAFT_376692	eukaryota>fungi	Lobosporangium	transversale	hypothetical protein BCR41DRAFT_376692 [Lobosporangium transversale]
XP_017341532.1	511	LOC108275301	eukaryota>metazoa>chordata>vertebrata>actinopterygii	Ictalurus	punctatus	PREDICTED: neural Wiskott-Aldrich syndrome protein-like isoform X3 [Ictalurus punctatus]
XP_005164539.1	510	wasl,zgc:55478,zgc:77237	eukaryota>metazoa>chordata>vertebrata>actinopterygii	Danio	rerio	wiskott-Aldrich syndrome-like isoform X1 [Danio rerio]
XP_018111332.1	509	LOC108713094	eukaryota>metazoa>chordata>vertebrata	Xenopus	laevis	PREDICTED: neural Wiskott-Aldrich syndrome protein-like [Xenopus laevis]
XP_005159304.1	508	si:ch211-255112.2	eukaryota>metazoa>chordata>vertebrata>actinopterygii	Danio	rerio	sprouty-related, EVH1 domain containing 3 isoform X2 [Danio rerio]
XP_011606998.1	508	spred3	eukaryota>metazoa>chordata>vertebrata>actinopterygii	Takifugu	rubripes	PREDICTED: sprouty-related, EVH1 domain-containing protein 3 [Takifugu rubripes]
XP_004210605.2	508	LOC100201341	eukaryota>metazoa>cnidaria	Hydra	vulgaris	PREDICTED: neural Wiskott-Aldrich syndrome protein-like [Hydra vulgaris]
XP_011611714.1	506	LOC101069458	eukaryota>metazoa>chordata>vertebrata>actinopterygii	Takifugu	rubripes	PREDICTED: neural Wiskott-Aldrich syndrome protein-like isoform X2 [Takifugu rubripes]
XP_021209115.1	505	LOC101735452	eukaryota>metazoa>hexapoda	Bombyx	mori	protein enabled isoform X5 [Bombyx mori]
NP_001186429.2	505	WASL	eukaryota>metazoa>chordata>vertebrata	Gallus	gallus	neural Wiskott-Aldrich syndrome protein [Gallus gallus]
NP_003932.3	505	N-WASP,NWASP,WASPB	eukaryota>metazoa>chordata>vertebrata	Homo	sapiens	neural Wiskott-Aldrich syndrome protein [Homo sapiens]
XP_005550703.1	505	WASL	eukaryota>metazoa>chordata>vertebrata	Macaca	fascicularis	PREDICTED: neural Wiskott-Aldrich syndrome protein [Macaca fascicularis]
XP_003261315.1	505	WASL	eukaryota>metazoa>chordata>vertebrata	Nomascus	leucogenys	PREDICTED: neural Wiskott-Aldrich syndrome protein [Nomascus leucogenys]
XP_021209060.1	505	LOC101744543	eukaryota>metazoa>hexapoda	Bombyx	mori	neural Wiskott-Aldrich syndrome protein [Bombyx mori]
XP_013778209.1	504	LOC106462793	eukaryota>metazoa	Limulus	polyphemus	neural Wiskott-Aldrich syndrome protein-like isoform X1 [Limulus polyphemus]
XP_006819312.1	504	LOC102805238	eukaryota>metazoa>hemichordata	Saccoglossus	kowalevskii	PREDICTED: neural Wiskott-Aldrich syndrome protein-like [Saccoglossus kowalevskii]
XP_005593541.1	503	WAS	eukaryota>metazoa>chordata>vertebrata	Macaca	fascicularis	PREDICTED: wiskott-Aldrich syndrome protein isoform X3 [Macaca fascicularis]
XP_022256137.1	502	LOC106471824	eukaryota>metazoa	Limulus	polyphemus	vasodilator-stimulated phosphoprotein-like isoform X2 [Limulus polyphemus]
CAG02855.1	502	GSTEN:00022085:G:001	eukaryota>metazoa>chordata>vertebrata>actinopterygii	Tetraodon	nigroviridis	unnamed protein product, partial [Tetraodon nigroviridis]
NP_000368.1	502	IMD2,SCNX,THC,THC1,WASP,WASPA	eukaryota>metazoa>chordata>vertebrata	Homo	sapiens	wiskott-Aldrich syndrome protein [Homo sapiens]
XP_003276872.1	502	WAS	eukaryota>metazoa>chordata>vertebrata	Nomascus	leucogenys	PREDICTED: wiskott-Aldrich syndrome protein [Nomascus leucogenys]
XP_012354168.1	502	WAS	eukaryota>metazoa>chordata>vertebrata	Nomascus	leucogenys	PREDICTED: wiskott-Aldrich syndrome protein [Nomascus leucogenys]
NP_001076475.1	502	zgc:158395	eukaryota>metazoa>chordata>vertebrata>actinopterygii	Danio	rerio	neural Wiskott-Aldrich syndrome protein [Danio rerio]
XP_006539514.1	501	AW047143,D130060H24Rik,Spred-3	eukaryota>metazoa>chordata>vertebrata	Mus	musculus	PREDICTED: sprouty-related, EVH1 domain-containing protein 3 isoform X1 [Mus musculus]
NP_082735.2	501	2900021I12Rik,3110031I02Rik,N-WASP	eukaryota>metazoa>chordata>vertebrata	Mus	musculus	neural Wiskott-Aldrich syndrome protein 1 [Mus musculus]
XP_017350337.1	500	LOC108280080	eukaryota>metazoa>chordata>vertebrata>actinopterygii	Ictalurus	punctatus	PREDICTED: neural Wiskott-Aldrich syndrome protein-like [Ictalurus punctatus]
EFX81578.1	499	DAPPUDRAFT_102305	eukaryota>metazoa>crustacea	Daphnia	pulex	hypothetical protein DAPPUDRAFT_102305 [Daphnia pulex]
XP_011455973.1	497	LOC105348313	eukaryota>metazoa>mollusca	Crassostrea	gigas	PREDICTED: neural Wiskott-Aldrich syndrome protein isoform X1 [Crassostrea gigas]
CAG01070.1	496	GSTEN:00019699:G:001	eukaryota>metazoa>chordata>vertebrata>actinopterygii	Tetraodon	nigroviridis	unnamed protein product, partial [Tetraodon nigroviridis]
XP_011455974.1	496	LOC105348313	eukaryota>metazoa>mollusca	Crassostrea	gigas	PREDICTED: neural Wiskott-Aldrich syndrome protein isoform X2 [Crassostrea gigas]
XP_011455975.1	495	LOC105348313	eukaryota>metazoa>mollusca	Crassostrea	gigas	PREDICTED: neural Wiskott-Aldrich syndrome protein isoform X3 [Crassostrea gigas]
XP_014571408.1	494	L969DRAFT_70252	eukaryota>fungi>basidiomycota	Mixia	osmundae IAM 14324	hypothetical protein L969DRAFT_70252 [Mixia osmundae IAM 14324]
XP_021326103.1	494	zgc:158395	eukaryota>metazoa>chordata>vertebrata>actinopterygii	Danio	rerio	neural Wiskott-Aldrich syndrome protein isoform X1 [Danio rerio]
NP_788755.1	494	CG1520,D-WASP,Dm,WASP,Dmel\CG1520,DmWASP,dWASP,wasp,Wasp,WASP,wsp,Wsp,WSP	eukaryota>metazoa>hexapoda	Drosophila	melanogaster	WASp, isoform C [Drosophila melanogaster]
XP_019930893.1	494	LOC105348313	eukaryota>metazoa>mollusca	Crassostrea	gigas	PREDICTED: neural Wiskott-Aldrich syndrome protein isoform X4 [Crassostrea gigas]
XP_011677536.1	493	LOC576114	eukaryota>metazoa>echinodermata	Strongylocentrotus	purpuratus	PREDICTED: protein enabled homolog isoform X6 [Strongylocentrotus purpuratus]
XP_017992486.1	491	Malapachy_1048	eukaryota>fungi>basidiomycota	Malassezia	pachydermatis	neural wiskott-aldrich syndrome protein [Malassezia pachydermatis]
XP_006675251.1	490	BATDEDRAFT_21300	eukaryota>fungi>chytridiomycota	Batrachochytrium	dendrobatidis JAM81	hypothetical protein BATDEDRAFT_21300 [Batrachochytrium dendrobatidis JAM81]
XP_021330646.1	490	wasl,zgc:55478,zgc:77237	eukaryota>metazoa>chordata>vertebrata>actinopterygii	Danio	rerio	wiskott-Aldrich syndrome-like isoform X2 [Danio rerio]
XP_003726476.1	489	LOC576114	eukaryota>metazoa>echinodermata	Strongylocentrotus	purpuratus	PREDICTED: protein enabled homolog isoform X7 [Strongylocentrotus purpuratus]
XP_015299070.1	489	WAS	eukaryota>metazoa>chordata>vertebrata	Macaca	fascicularis	PREDICTED: wiskott-Aldrich syndrome protein isoform X1 [Macaca fascicularis]
XP_017323358.1	488	was	eukaryota>metazoa>chordata>vertebrata>actinopterygii	Ictalurus	punctatus	PREDICTED: wiskott-Aldrich syndrome protein [Ictalurus punctatus]
XP_011677537.1	487	LOC576114	eukaryota>metazoa>echinodermata	Strongylocentrotus	purpuratus	PREDICTED: protein enabled homolog isoform X8 [Strongylocentrotus purpuratus]
XP_013404564.1	485	LOC106169606	eukaryota>metazoa	Lingula	anatina	protein enabled homolog isoform X1 [Lingula anatina]
XP_013404565.1	484	LOC106169606	eukaryota>metazoa	Lingula	anatina	protein enabled homolog isoform X2 [Lingula anatina]
XP_022237658.1	484	LOC106478313	eukaryota>metazoa	Limulus	polyphemus	sprouty-related, EVH1 domain-containing protein 2-like isoform X1 [Limulus polyphemus]
XP_011416187.1	484	LOC105320101	eukaryota>metazoa>mollusca	Crassostrea	gigas	PREDICTED: vasodilator-stimulated phosphoprotein isoform X1 [Crassostrea gigas]
XP_006811741.1	484	LOC102809815	eukaryota>metazoa>hemichordata	Saccoglossus	kowalevskii	PREDICTED: neural Wiskott-Aldrich syndrome protein-like [Saccoglossus kowalevskii]
ORY23836.1	482	LY90DRAFT_119424	eukaryota>fungi>chytridiomycota	Neocallimastix	californiae	WH1-domain-containing protein [Neocallimastix californiae]
XP_005705462.1	482	Gasu_36760	eukaryota>rhodophyta	Galdieria	sulphuraria	hypothetical protein Gasu_36760 [Galdieria sulphuraria]
EFX84664.1	481	DAPPUDRAFT_99608	eukaryota>metazoa>crustacea	Daphnia	pulex	hypothetical protein DAPPUDRAFT_99608 [Daphnia pulex]
XP_018650128.1	481	Smp_082780	eukaryota>metazoa	Schistosoma	mansoni	LOW QUALITY PROTEIN: putative wiskott-aldrich syndrome protein [Schistosoma mansoni]
XP_009030533.1	481	HELRODRAFT_165783	eukaryota>metazoa>annelida	Helobdella	robusta	hypothetical protein HELRODRAFT_165783 [Helobdella robusta]
XP_011416188.1	479	LOC105320101	eukaryota>metazoa>mollusca	Crassostrea	gigas	PREDICTED: vasodilator-stimulated phosphoprotein isoform X2 [Crassostrea gigas]
XP_018267610.1	479	RHOBADRAFT_56597	eukaryota>fungi>basidiomycota	Rhodotorula	graminis WP1	hypothetical protein RHOBADRAFT_56597 [Rhodotorula graminis WP1]
NP_956232.1	479	was,wu:fk81c08,zgc:64164	eukaryota>metazoa>chordata>vertebrata>actinopterygii	Danio	rerio	wiskott-Aldrich syndrome protein [Danio rerio]
ELR19008.1	478	ACA1_234760	eukaryota>amoebozoa>acanthamoebidae	Acanthamoeba	castellanii str. Neff	WH1 domain containing protein [Acanthamoeba castellanii str. Neff]
XP_021202025.1	477	LOC101739722	eukaryota>metazoa>hexapoda	Bombyx	mori	sprouty-related, EVH1 domain-containing protein 1 isoform X1 [Bombyx mori]
XP_009065704.1	477	LOTGIDRAFT_169157	eukaryota>metazoa>mollusca	Lottia	gigantea	hypothetical protein LOTGIDRAFT_169157 [Lottia gigantea]
NP_001272147.1	476	EVL	eukaryota>metazoa>chordata>vertebrata	Macaca	fascicularis	ena/VASP-like protein [Macaca fascicularis]
EIE88214.1	476	RO3G_12925	eukaryota>fungi	Rhizopus	delemar RA 99-880	hypothetical protein RO3G_12925 [Rhizopus delemar RA 99-880]
XP_002474407.1	476	POSPLDRAFT_87772	eukaryota>fungi>basidiomycota	Postia	placenta Mad-698-R	predicted protein [Postia placenta Mad-698-R]
XP_002404030.1	476	IscW_ISCW008485	eukaryota>metazoa	Ixodes	scapularis	wiskott-aldrich syndrome protein, putative, partial [Ixodes scapularis]
KXJ29811.1	476	WASL	eukaryota>metazoa>cnidaria	Exaiptasia	pallida	Neural Wiskott-Aldrich syndrome protein [Exaiptasia pallida]
NP_001091372.1	474	imd2,thc,thc1,was,wasp	eukaryota>metazoa>chordata>vertebrata	Xenopus	laevis	Wiskott-Aldrich syndrome L homeolog [Xenopus laevis]
XP_016612873.1	473	SPPG_00537	eukaryota>fungi>chytridiomycota	Spizellomyces	punctatus DAOM BR117	hypothetical protein SPPG_00537 [Spizellomyces punctatus DAOM BR117]
XP_002675872.1	473	NAEGRDRAFT_58361	eukaryota>heterolobosea	Naegleria	gruberi strain NEG-M	wiscott-aldrich syndrome protein [Naegleria gruberi strain NEG-M]
XP_013788137.1	473	LOC106472053	eukaryota>metazoa	Limulus	polyphemus	protein still life, isoform SIF type 1-like, partial [Limulus polyphemus]
NP_001091123.1	472	imd2,thc,thc1,wasp	eukaryota>metazoa>chordata>vertebrata	Xenopus	laevis	uncharacterized protein LOC100036872 [Xenopus laevis]
XP_013404566.1	471	LOC106169606	eukaryota>metazoa	Lingula	anatina	protein enabled homolog isoform X3 [Lingula anatina]
XP_004923743.1	471	LOC101739722	eukaryota>metazoa>hexapoda	Bombyx	mori	sprouty-related, EVH1 domain-containing protein 1 isoform X2 [Bombyx mori]
XP_011416189.1	470	LOC105320101	eukaryota>metazoa>mollusca	Crassostrea	gigas	PREDICTED: vasodilator-stimulated phosphoprotein isoform X3 [Crassostrea gigas]
XP_002402728.1	470	IscW_ISCW007640	eukaryota>metazoa	Ixodes	scapularis	hypothetical protein IscW_ISCW007640 [Ixodes scapularis]
XP_013386482.1	469	LOC106155980	eukaryota>metazoa	Lingula	anatina	sprouty-related, EVH1 domain-containing protein 2 isoform X1 [Lingula anatina]
NP_503360.4	468	unc-34	eukaryota>metazoa>nematoda	Caenorhabditis	elegans	Uncharacterized protein CELE_Y50D4C.1 [Caenorhabditis elegans]
XP_012750742.1	467	SAMD00019534_093630	eukaryota>amoebozoa>mycetozoa>dictyosteliida	Acytostelium	subglobosum LB1	hypothetical protein SAMD00019534_093630 [Acytostelium subglobosum LB1]
XP_022238298.1	467	LOC106478900	eukaryota>metazoa	Limulus	polyphemus	neural Wiskott-Aldrich syndrome protein-like isoform X1 [Limulus polyphemus]
XP_022238299.1	467	LOC106478900	eukaryota>metazoa	Limulus	polyphemus	neural Wiskott-Aldrich syndrome protein-like isoform X1 [Limulus polyphemus]
XP_013404567.1	466	LOC106169606	eukaryota>metazoa	Lingula	anatina	protein enabled homolog isoform X4 [Lingula anatina]
XP_003963906.1	464	LOC101072986	eukaryota>metazoa>chordata>vertebrata>actinopterygii	Takifugu	rubripes	PREDICTED: sprouty-related, EVH1 domain-containing protein 2-like isoform X1 [Takifugu rubripes]
XP_001879567.1	464	LACBIDRAFT_325848	eukaryota>fungi>basidiomycota	Laccaria	bicolor S238N-H82	actin regulatory protein [Laccaria bicolor S238N-H82]
XP_013404568.1	463	LOC106169606	eukaryota>metazoa	Lingula	anatina	protein enabled homolog isoform X5 [Lingula anatina]
CAF92534.1	463	GSTEN:00007816:G:001	eukaryota>metazoa>chordata>vertebrata>actinopterygii	Tetraodon	nigroviridis	unnamed protein product, partial [Tetraodon nigroviridis]
XP_011416190.1	463	LOC105320101	eukaryota>metazoa>mollusca	Crassostrea	gigas	PREDICTED: vasodilator-stimulated phosphoprotein isoform X4 [Crassostrea gigas]
EUB64238.1	462	EGR_00782	eukaryota>metazoa	Echinococcus	granulosus	Homer protein [Echinococcus granulosus]
XP_011390162.1	460	UMAG_03687	eukaryota>fungi>basidiomycota	Ustilago	maydis 521	hypothetical protein UMAG_03687 [Ustilago maydis 521]
XP_011416191.1	459	LOC105320101	eukaryota>metazoa>mollusca	Crassostrea	gigas	PREDICTED: vasodilator-stimulated phosphoprotein isoform X5 [Crassostrea gigas]
XP_014150933.1	459	SARC_10494	eukaryota>ichthyosporea	Sphaeroforma	arctica JP610	hypothetical protein SARC_10494 [Sphaeroforma arctica JP610]
CAG05251.1	458	GSTEN:00025218:G:001	eukaryota>metazoa>chordata>vertebrata>actinopterygii	Tetraodon	nigroviridis	unnamed protein product, partial [Tetraodon nigroviridis]
NP_001300274.1	458	unc-34	eukaryota>metazoa>nematoda	Caenorhabditis	elegans	Uncharacterized protein CELE_Y50D4C.1 [Caenorhabditis elegans]
XP_013787898.1	457	LOC106471824	eukaryota>metazoa	Limulus	polyphemus	vasodilator-stimulated phosphoprotein-like isoform X3 [Limulus polyphemus]
XP_022244561.1	457	LOC106461679	eukaryota>metazoa	Limulus	polyphemus	sprouty-related, EVH1 domain-containing protein 2-like isoform X2 [Limulus polyphemus]
XP_013404569.1	456	LOC106169606	eukaryota>metazoa	Lingula	anatina	protein enabled homolog isoform X6 [Lingula anatina]
XP_022237664.1	456	LOC106478313	eukaryota>metazoa	Limulus	polyphemus	sprouty-related, EVH1 domain-containing protein 2-like isoform X2 [Limulus polyphemus]
XP_005254259.1	456	hSpred1,NFLS,PPP1R147,spred-1	eukaryota>metazoa>chordata>vertebrata	Homo	sapiens	sprouty-related, EVH1 domain-containing protein 1 isoform X1 [Homo sapiens]
XP_015308710.1	456	EVL	eukaryota>metazoa>chordata>vertebrata	Macaca	fascicularis	PREDICTED: ena/VASP-like protein isoform X1 [Macaca fascicularis]
CAG00296.1	456	GSTEN:00018693:G:001	eukaryota>metazoa>chordata>vertebrata>actinopterygii	Tetraodon	nigroviridis	unnamed protein product, partial [Tetraodon nigroviridis]
XP_008193941.1	456	LOC659462	eukaryota>metazoa>hexapoda	Tribolium	castaneum	PREDICTED: neural Wiskott-Aldrich syndrome protein [Tribolium castaneum]
XP_008193942.1	456	LOC659462	eukaryota>metazoa>hexapoda	Tribolium	castaneum	PREDICTED: neural Wiskott-Aldrich syndrome protein [Tribolium castaneum]
XP_015836057.1	456	LOC659462	eukaryota>metazoa>hexapoda	Tribolium	castaneum	PREDICTED: neural Wiskott-Aldrich syndrome protein [Tribolium castaneum]
XP_013404570.1	454	LOC106169606	eukaryota>metazoa	Lingula	anatina	protein enabled homolog isoform X7 [Lingula anatina]
XP_022237668.1	454	LOC106478313	eukaryota>metazoa	Limulus	polyphemus	sprouty-related, EVH1 domain-containing protein 2-like isoform X3 [Limulus polyphemus]
XP_012543798.1	454	LOC101739722	eukaryota>metazoa>hexapoda	Bombyx	mori	sprouty-related, EVH1 domain-containing protein 1 isoform X3 [Bombyx mori]
XP_970236.1	454	LOC658784	eukaryota>metazoa>hexapoda	Tribolium	castaneum	PREDICTED: protein enabled isoform X1 [Tribolium castaneum]
EPB87056.1	454	HMPREF1544_06173	eukaryota>fungi	Mucor	circinelloides f. circinelloides 1006PhL	hypothetical protein HMPREF1544_06173 [Mucor circinelloides f. circinelloides 1006PhL]
XP_002172976.2	453	SJAG_01732	eukaryota>fungi>ascomycota	Schizosaccharomyces	japonicus yFS275	WASp [Schizosaccharomyces japonicus yFS275]
XP_012049873.1	453	CNAG_02029	eukaryota>fungi>basidiomycota	Cryptococcus	neoformans var. grubii H99	wiskott-Aldrich syndrome protein [Cryptococcus neoformans var. grubii H99]
CAF96784.1	453	GSTEN:00013947:G:001	eukaryota>metazoa>chordata>vertebrata>actinopterygii	Tetraodon	nigroviridis	unnamed protein product, partial [Tetraodon nigroviridis]
XP_022243774.1	452	LOC106461201	eukaryota>metazoa	Limulus	polyphemus	sprouty-related, EVH1 domain-containing protein 2-like isoform X1 [Limulus polyphemus]
XP_021872601.1	452	BD324DRAFT_376200	eukaryota>fungi>basidiomycota	Kockovaella	imperatae	hypothetical protein BD324DRAFT_376200 [Kockovaella imperatae]
XP_022257023.1	451	LOC106472788	eukaryota>metazoa	Limulus	polyphemus	protein enabled homolog [Limulus polyphemus]
XP_009022403.1	451	HELRODRAFT_101607	eukaryota>metazoa>annelida	Helobdella	robusta	hypothetical protein HELRODRAFT_101607 [Helobdella robusta]
XP_015289071.1	451	SPRED2	eukaryota>metazoa>chordata>vertebrata	Macaca	fascicularis	PREDICTED: sprouty-related, EVH1 domain-containing protein 2 isoform X1 [Macaca fascicularis]
XP_013776452.1	450	LOC106461201	eukaryota>metazoa	Limulus	polyphemus	sprouty-related, EVH1 domain-containing protein 2-like isoform X2 [Limulus polyphemus]
XP_013788892.1	450	LOC106472781	eukaryota>metazoa	Limulus	polyphemus	sprouty-related, EVH1 domain-containing protein 2-like isoform X1 [Limulus polyphemus]
XP_011542279.1	450	IMD2,SCNX,THC,THC1,WASP,WASPA	eukaryota>metazoa>chordata>vertebrata	Homo	sapiens	wiskott-Aldrich syndrome protein isoform X1 [Homo sapiens]
XP_001944028.1	450	LOC100160932	eukaryota>metazoa>hexapoda	Acyrthosiphon	pisum	PREDICTED: neural Wiskott-Aldrich syndrome protein [Acyrthosiphon pisum]
XP_014780288.1	448	LOC106876307	eukaryota>metazoa>mollusca	Octopus	bimaculoides	PREDICTED: sprouty-related, EVH1 domain-containing protein 2-like [Octopus bimaculoides]
XP_002734199.1	447	LOC100376569	eukaryota>metazoa>hemichordata	Saccoglossus	kowalevskii	PREDICTED: protein enabled homolog isoform X1 [Saccoglossus kowalevskii]
XP_011416192.1	446	LOC105320101	eukaryota>metazoa>mollusca	Crassostrea	gigas	PREDICTED: vasodilator-stimulated phosphoprotein isoform X6 [Crassostrea gigas]
XP_018087247.1	446	LOC100036872	eukaryota>metazoa>chordata>vertebrata	Xenopus	laevis	PREDICTED: uncharacterized protein LOC100036872 isoform X1 [Xenopus laevis]
XP_005173736.1	445	vaspb	eukaryota>metazoa>chordata>vertebrata>actinopterygii	Danio	rerio	vasodilator-stimulated phosphoprotein isoform X1 [Danio rerio]
NP_689807.1	444	hSpred1,NFLS,PPP1R147,spred-1	eukaryota>metazoa>chordata>vertebrata	Homo	sapiens	sprouty-related, EVH1 domain-containing protein 1 [Homo sapiens]
XP_005559192.1	444	SPRED1	eukaryota>metazoa>chordata>vertebrata	Macaca	fascicularis	PREDICTED: sprouty-related, EVH1 domain-containing protein 1 [Macaca fascicularis]
NP_277059.1	444	5730461F13Rik,AL024345,AW047647	eukaryota>metazoa>chordata>vertebrata	Mus	musculus	sprouty-related, EVH1 domain-containing protein 1 isoform 1 [Mus musculus]
XP_003272865.1	444	SPRED1	eukaryota>metazoa>chordata>vertebrata	Nomascus	leucogenys	PREDICTED: sprouty-related, EVH1 domain-containing protein 1 [Nomascus leucogenys]
XP_011601392.1	444	LOC101072986	eukaryota>metazoa>chordata>vertebrata>actinopterygii	Takifugu	rubripes	PREDICTED: sprouty-related, EVH1 domain-containing protein 2-like isoform X2 [Takifugu rubripes]
XP_011416194.1	444	LOC105320101	eukaryota>metazoa>mollusca	Crassostrea	gigas	PREDICTED: vasodilator-stimulated phosphoprotein isoform X7 [Crassostrea gigas]
XP_011416195.1	444	LOC105320101	eukaryota>metazoa>mollusca	Crassostrea	gigas	PREDICTED: vasodilator-stimulated phosphoprotein isoform X8 [Crassostrea gigas]
XP_022243427.1	442	LOC106460914	eukaryota>metazoa	Limulus	polyphemus	homer protein homolog 2-like isoform X1 [Limulus polyphemus]
XP_016663928.1	442	LOC100164896	eukaryota>metazoa>hexapoda	Acyrthosiphon	pisum	PREDICTED: protein enabled isoform X1 [Acyrthosiphon pisum]
ORX77358.1	442	K493DRAFT_321397	eukaryota>fungi	Basidiobolus	meristosporus CBS 931.73	hypothetical protein K493DRAFT_321397 [Basidiobolus meristosporus CBS 931.73]
XP_013241071.1	442	K437DRAFT_279355	eukaryota>fungi>basidiomycota	Tilletiaria	anomala UBC 951	WH1-domain-containing protein [Tilletiaria anomala UBC 951]
XP_022243776.1	441	LOC106461201	eukaryota>metazoa	Limulus	polyphemus	sprouty-related, EVH1 domain-containing protein 2-like isoform X3 [Limulus polyphemus]
XP_015142830.1	441	SPRED1	eukaryota>metazoa>chordata>vertebrata	Gallus	gallus	sprouty-related, EVH1 domain-containing protein 1 isoform X1 [Gallus gallus]
XP_016663929.1	441	LOC100164896	eukaryota>metazoa>hexapoda	Acyrthosiphon	pisum	PREDICTED: protein enabled isoform X2 [Acyrthosiphon pisum]
XP_009062454.1	441	LOTGIDRAFT_235185	eukaryota>metazoa>mollusca	Lottia	gigantea	hypothetical protein LOTGIDRAFT_235185 [Lottia gigantea]
XP_011419451.1	441	LOC105322431	eukaryota>metazoa>mollusca	Crassostrea	gigas	PREDICTED: homer protein homolog 2 isoform X4 [Crassostrea gigas]
XP_013386483.1	440	LOC106155980	eukaryota>metazoa	Lingula	anatina	sprouty-related, EVH1 domain-containing protein 2 isoform X2 [Lingula anatina]
XP_015142831.1	440	SPRED1	eukaryota>metazoa>chordata>vertebrata	Gallus	gallus	sprouty-related, EVH1 domain-containing protein 1 isoform X2 [Gallus gallus]
KNE58346.1	440	AMAG_03930	eukaryota>fungi	Allomyces	macrogynus ATCC 38327	hypothetical protein AMAG_03930 [Allomyces macrogynus ATCC 38327]
XP_022238301.1	440	LOC106478900	eukaryota>metazoa	Limulus	polyphemus	neural Wiskott-Aldrich syndrome protein-like isoform X2 [Limulus polyphemus]
XP_022257018.1	439	LOC106472781	eukaryota>metazoa	Limulus	polyphemus	sprouty-related, EVH1 domain-containing protein 2-like isoform X2 [Limulus polyphemus]
XP_013780830.2	438	LOC106465171	eukaryota>metazoa	Limulus	polyphemus	protein enabled homolog isoform X1 [Limulus polyphemus]
XP_018118304.1	438	LOC101027290	eukaryota>metazoa>chordata>vertebrata	Xenopus	laevis	PREDICTED: sprouty-related, EVH1 domain-containing protein 2 isoform X1 [Xenopus laevis]
XP_022249230.1	437	LOC106465171	eukaryota>metazoa	Limulus	polyphemus	ena/VASP-like protein isoform X2 [Limulus polyphemus]
XP_015308711.1	435	EVL	eukaryota>metazoa>chordata>vertebrata	Macaca	fascicularis	PREDICTED: ena/VASP-like protein isoform X2 [Macaca fascicularis]
XP_015834043.1	435	LOC658784	eukaryota>metazoa>hexapoda	Tribolium	castaneum	PREDICTED: protein enabled isoform X2 [Tribolium castaneum]
XP_015308712.1	434	EVL	eukaryota>metazoa>chordata>vertebrata	Macaca	fascicularis	PREDICTED: ena/VASP-like protein isoform X3 [Macaca fascicularis]
XP_019856526.1	434	LOC100632931	eukaryota>metazoa	Amphimedon	queenslandica	PREDICTED: neural Wiskott-Aldrich syndrome protein-like [Amphimedon queenslandica]
XP_003262512.1	433	SPRED2	eukaryota>metazoa>chordata>vertebrata	Nomascus	leucogenys	PREDICTED: sprouty-related, EVH1 domain-containing protein 2 isoform X1 [Nomascus leucogenys]
XP_022243777.1	431	LOC106461201	eukaryota>metazoa	Limulus	polyphemus	sprouty-related, EVH1 domain-containing protein 2-like isoform X4 [Limulus polyphemus]
XP_008188064.1	431	LOC100164896	eukaryota>metazoa>hexapoda	Acyrthosiphon	pisum	PREDICTED: protein enabled isoform X3 [Acyrthosiphon pisum]
XP_013773340.2	430	LOC106458378	eukaryota>metazoa	Limulus	polyphemus	homer protein homolog 1-like isoform X1 [Limulus polyphemus]
XP_011617856.1	430	spred1	eukaryota>metazoa>chordata>vertebrata>actinopterygii	Takifugu	rubripes	PREDICTED: sprouty-related, EVH1 domain-containing protein 1 [Takifugu rubripes]
XP_016663930.1	430	LOC100164896	eukaryota>metazoa>hexapoda	Acyrthosiphon	pisum	PREDICTED: protein enabled isoform X4 [Acyrthosiphon pisum]
XP_011446805.1	430	LOC105341794	eukaryota>metazoa>mollusca	Crassostrea	gigas	PREDICTED: sprouty-related, EVH1 domain-containing protein 2 isoform X1 [Crassostrea gigas]
KNE72674.1	430	AMAG_16434	eukaryota>fungi	Allomyces	macrogynus ATCC 38327	hypothetical protein AMAG_16434 [Allomyces macrogynus ATCC 38327]
XP_004349279.2	429	CAOG_02529	eukaryota>ichthyosporea	Capsaspora	owczarzaki ATCC 30864	vasodilator-stimulated phosphoprotein [Capsaspora owczarzaki ATCC 30864]
XP_016876852.1	429	RNB6	eukaryota>metazoa>chordata>vertebrata	Homo	sapiens	ena/VASP-like protein isoform X1 [Homo sapiens]
XP_017321265.1	429	vasp	eukaryota>metazoa>chordata>vertebrata>actinopterygii	Ictalurus	punctatus	PREDICTED: vasodilator-stimulated phosphoprotein isoform X1 [Ictalurus punctatus]
XP_017321266.1	428	vasp	eukaryota>metazoa>chordata>vertebrata>actinopterygii	Ictalurus	punctatus	PREDICTED: vasodilator-stimulated phosphoprotein isoform X2 [Ictalurus punctatus]
XP_013776126.1	427	LOC106460914	eukaryota>metazoa	Limulus	polyphemus	homer protein homolog 2-like isoform X2 [Limulus polyphemus]
XP_006514519.1	427	C79158	eukaryota>metazoa>chordata>vertebrata	Mus	musculus	PREDICTED: sprouty-related, EVH1 domain-containing protein 2 isoform X1 [Mus musculus]
XP_003972234.1	427	LOC101071062	eukaryota>metazoa>chordata>vertebrata>actinopterygii	Takifugu	rubripes	PREDICTED: sprouty-related, EVH1 domain-containing protein 2-like [Takifugu rubripes]
XP_686737.2	427	spred2a	eukaryota>metazoa>chordata>vertebrata>actinopterygii	Danio	rerio	sprouty-related, EVH1 domain-containing protein 2 [Danio rerio]
XP_012362561.1	426	LOC100581604	eukaryota>metazoa>chordata>vertebrata	Nomascus	leucogenys	PREDICTED: homer protein homolog 2 [Nomascus leucogenys]
XP_005173737.1	426	vaspb	eukaryota>metazoa>chordata>vertebrata>actinopterygii	Danio	rerio	vasodilator-stimulated phosphoprotein isoform X2 [Danio rerio]
NP_610988.1	426	AE33,CG10155,Dm,AE33,Dmel\CG10155,dSPRED,spred	eukaryota>metazoa>hexapoda	Drosophila	melanogaster	Sprouty-related protein with EVH-1 domain, isoform A [Drosophila melanogaster]
XP_018084657.1	425	xevl	eukaryota>metazoa>chordata>vertebrata	Xenopus	laevis	PREDICTED: ena/VASP-like protein isoform X6 [Xenopus laevis]
XP_017320177.1	424	LOC108263653	eukaryota>metazoa>chordata>vertebrata>actinopterygii	Ictalurus	punctatus	PREDICTED: sprouty-related, EVH1 domain-containing protein 2-like, partial [Ictalurus punctatus]
XP_012751000.1	423	SAMD00019534_091320	eukaryota>amoebozoa>mycetozoa>dictyosteliida	Acytostelium	subglobosum LB1	hypothetical protein SAMD00019534_091320, partial [Acytostelium subglobosum LB1]
XP_011519591.1	423	hSpred1,NFLS,PPP1R147,spred-1	eukaryota>metazoa>chordata>vertebrata	Homo	sapiens	sprouty-related, EVH1 domain-containing protein 1 isoform X2 [Homo sapiens]
XP_018085765.1	423	LOC108698640	eukaryota>metazoa>chordata>vertebrata	Xenopus	laevis	PREDICTED: sprouty-related, EVH1 domain-containing protein 1-like [Xenopus laevis]
XP_785660.2	423	LOC580515	eukaryota>metazoa>echinodermata	Strongylocentrotus	purpuratus	PREDICTED: sprouty-related, EVH1 domain-containing protein 3 [Strongylocentrotus purpuratus]
XP_011150921.1	423	LOC105190107	eukaryota>metazoa>hexapoda	Harpegnathos	saltator	homer protein homolog 1 isoform X1 [Harpegnathos saltator]
XP_395474.3	423	LOC412008	eukaryota>metazoa>hexapoda	Apis	mellifera	PREDICTED: sprouty-related, EVH1 domain-containing protein 1 isoform X1 [Apis mellifera]
XP_011446806.1	423	LOC105341794	eukaryota>metazoa>mollusca	Crassostrea	gigas	PREDICTED: sprouty-related, EVH1 domain-containing protein 2 isoform X2 [Crassostrea gigas]
XP_022245846.1	423	LOC106462793	eukaryota>metazoa	Limulus	polyphemus	neural Wiskott-Aldrich syndrome protein-like isoform X2 [Limulus polyphemus]
XP_015142957.1	422	EVL	eukaryota>metazoa>chordata>vertebrata	Gallus	gallus	ena/VASP-like protein isoform X3 [Gallus gallus]
XP_005267806.1	422	RNB6	eukaryota>metazoa>chordata>vertebrata	Homo	sapiens	ena/VASP-like protein isoform X2 [Homo sapiens]
XP_005562254.1	422	EVL	eukaryota>metazoa>chordata>vertebrata	Macaca	fascicularis	PREDICTED: ena/VASP-like protein isoform X4 [Macaca fascicularis]
XP_018088843.1	422	LOC108700284	eukaryota>metazoa>chordata>vertebrata	Xenopus	laevis	PREDICTED: sprouty-related, EVH1 domain-containing protein 1-like [Xenopus laevis]
XP_008191425.1	422	LOC658784	eukaryota>metazoa>hexapoda	Tribolium	castaneum	PREDICTED: protein enabled isoform X3 [Tribolium castaneum]
XP_396723.3	422	LOC413278	eukaryota>metazoa>hexapoda	Apis	mellifera	PREDICTED: homer protein homolog 1 isoform X1 [Apis mellifera]
XP_006539515.1	421	AW047143,D130060H24Rik,Spred-3	eukaryota>metazoa>chordata>vertebrata	Mus	musculus	PREDICTED: sprouty-related, EVH1 domain-containing protein 3 isoform X2 [Mus musculus]
XP_006515533.1	420	AI528774	eukaryota>metazoa>chordata>vertebrata	Mus	musculus	PREDICTED: ena/VASP-like protein isoform X1 [Mus musculus]
NP_001161662.1	420	LOC100313739	eukaryota>metazoa>hemichordata	Saccoglossus	kowalevskii	sprouty-like protein [Saccoglossus kowalevskii]
XP_013386484.1	419	LOC106155980	eukaryota>metazoa	Lingula	anatina	PREDICTED: sprouty-related, EVH1 domain-containing protein 2-like isoform X3 [Lingula anatina]
XP_019851701.1	419	LOC100640667	eukaryota>metazoa	Amphimedon	queenslandica	PREDICTED: homer protein homolog 2-like isoform X1 [Amphimedon queenslandica]
XP_015133575.1	419	SPRED2	eukaryota>metazoa>chordata>vertebrata	Gallus	gallus	sprouty-related, EVH1 domain-containing protein 2 isoform X1 [Gallus gallus]
NP_001090464.1	419	evl,xevl	eukaryota>metazoa>chordata>vertebrata	Xenopus	laevis	Enah/Vasp-like S homeolog [Xenopus laevis]
XP_018084658.1	419	xevl	eukaryota>metazoa>chordata>vertebrata	Xenopus	laevis	PREDICTED: ena/VASP-like protein isoform X7 [Xenopus laevis]
XP_011419452.1	419	LOC105322431	eukaryota>metazoa>mollusca	Crassostrea	gigas	PREDICTED: homer protein homolog 2 isoform X5 [Crassostrea gigas]
XP_003386457.1	418	LOC100640667	eukaryota>metazoa	Amphimedon	queenslandica	PREDICTED: homer protein homolog 2-like isoform X2 [Amphimedon queenslandica]
NP_001006487.1	418	EVL	eukaryota>metazoa>chordata>vertebrata	Gallus	gallus	ena/VASP-like protein [Gallus gallus]
NP_057421.1	418	RNB6	eukaryota>metazoa>chordata>vertebrata	Homo	sapiens	ena/VASP-like protein isoform 1 [Homo sapiens]
NP_861449.2	418	Spred-2	eukaryota>metazoa>chordata>vertebrata	Homo	sapiens	sprouty-related, EVH1 domain-containing protein 2 isoform a [Homo sapiens]
XP_005562255.1	418	EVL	eukaryota>metazoa>chordata>vertebrata	Macaca	fascicularis	PREDICTED: ena/VASP-like protein isoform X5 [Macaca fascicularis]
XP_005575784.1	418	SPRED2	eukaryota>metazoa>chordata>vertebrata	Macaca	fascicularis	PREDICTED: sprouty-related, EVH1 domain-containing protein 2 isoform X2 [Macaca fascicularis]
XP_012356692.1	418	SPRED2	eukaryota>metazoa>chordata>vertebrata	Nomascus	leucogenys	PREDICTED: sprouty-related, EVH1 domain-containing protein 2 isoform X2 [Nomascus leucogenys]
OQV25854.1	417	BV898_00004	eukaryota>metazoa	Hypsibius	dujardini	putative Homer protein-like protein 1 [Hypsibius dujardini]
XP_015142958.1	416	EVL	eukaryota>metazoa>chordata>vertebrata	Gallus	gallus	PREDICTED: ena/VASP-like protein isoform X2 [Gallus gallus]
XP_016876853.1	416	RNB6	eukaryota>metazoa>chordata>vertebrata	Homo	sapiens	PREDICTED: ena/VASP-like protein isoform X3 [Homo sapiens]
XP_006515534.1	416	AI528774	eukaryota>metazoa>chordata>vertebrata	Mus	musculus	PREDICTED: ena/VASP-like protein isoform X2 [Mus musculus]
XP_017312716.1	416	spred2	eukaryota>metazoa>chordata>vertebrata>actinopterygii	Ictalurus	punctatus	PREDICTED: sprouty-related, EVH1 domain-containing protein 2 [Ictalurus punctatus]
XP_002113570.1	416	TRIADDRAFT_57130	eukaryota>metazoa>placozoa	Trichoplax	adhaerens	hypothetical protein TRIADDRAFT_57130 [Trichoplax adhaerens]
XP_022238302.1	416	LOC106478900	eukaryota>metazoa	Limulus	polyphemus	neural Wiskott-Aldrich syndrome protein-like isoform X3 [Limulus polyphemus]
XP_015142832.1	415	SPRED1	eukaryota>metazoa>chordata>vertebrata	Gallus	gallus	sprouty-related, EVH1 domain-containing protein 1 isoform X3 [Gallus gallus]
NP_001121682.1	415	Spred-2	eukaryota>metazoa>chordata>vertebrata	Homo	sapiens	sprouty-related, EVH1 domain-containing protein 2 isoform b [Homo sapiens]
XP_005575785.1	415	SPRED2	eukaryota>metazoa>chordata>vertebrata	Macaca	fascicularis	PREDICTED: sprouty-related, EVH1 domain-containing protein 2 isoform X3 [Macaca fascicularis]
XP_003262510.1	415	SPRED2	eukaryota>metazoa>chordata>vertebrata	Nomascus	leucogenys	PREDICTED: sprouty-related, EVH1 domain-containing protein 2 isoform X3 [Nomascus leucogenys]
XP_011419453.1	415	LOC105322431	eukaryota>metazoa>mollusca	Crassostrea	gigas	PREDICTED: homer protein homolog 2 isoform X6 [Crassostrea gigas]
NP_001186638.1	414	SPRED1	eukaryota>metazoa>chordata>vertebrata	Gallus	gallus	sprouty-related, EVH1 domain-containing protein 1 [Gallus gallus]
NP_001156866.1	414	AI528774,b2b2600Clo	eukaryota>metazoa>chordata>vertebrata	Mus	musculus	ena/VASP-like protein isoform 1 [Mus musculus]
NP_998399.1	413	fb93f08,spred2,wu:fb93f08,zgc:77284	eukaryota>metazoa>chordata>vertebrata>actinopterygii	Danio	rerio	sprouty-related, EVH1 domain-containing protein 2 [Danio rerio]
EFX81546.1	413	DAPPUDRAFT_50104	eukaryota>metazoa>crustacea	Daphnia	pulex	hypothetical protein DAPPUDRAFT_50104 [Daphnia pulex]
XP_015297414.1	412	SPRED3	eukaryota>metazoa>chordata>vertebrata	Macaca	fascicularis	PREDICTED: sprouty-related, EVH1 domain-containing protein 3 isoform X1 [Macaca fascicularis]
XP_018120328.1	412	LOC108717624	eukaryota>metazoa>chordata>vertebrata	Xenopus	laevis	PREDICTED: sprouty-related, EVH1 domain-containing protein 2-like isoform X1 [Xenopus laevis]
NP_001103922.1	412	vasp,zgc:162320	eukaryota>metazoa>chordata>vertebrata>actinopterygii	Danio	rerio	vasodilator-stimulated phosphoprotein [Danio rerio]
XP_005158854.1	412	Evl,id:ibd2260,zgc:65883,zgc:77388	eukaryota>metazoa>chordata>vertebrata>actinopterygii	Danio	rerio	ena/VASP-like protein isoform X7 [Danio rerio]
XP_017332061.1	412	spred1	eukaryota>metazoa>chordata>vertebrata>actinopterygii	Ictalurus	punctatus	PREDICTED: sprouty-related, EVH1 domain-containing protein 1 [Ictalurus punctatus]
XP_006559192.1	411	LOC412008	eukaryota>metazoa>hexapoda	Apis	mellifera	PREDICTED: sprouty-related, EVH1 domain-containing protein 1 isoform X2 [Apis mellifera]
XP_018118305.1	411	LOC101027290	eukaryota>metazoa>chordata>vertebrata	Xenopus	laevis	PREDICTED: sprouty-related, EVH1 domain-containing protein 2 isoform X2 [Xenopus laevis]
XP_005158855.1	411	Evl,id:ibd2260,zgc:65883,zgc:77388	eukaryota>metazoa>chordata>vertebrata>actinopterygii	Danio	rerio	ena/VASP-like protein isoform X8 [Danio rerio]
XP_001950074.2	411	LOC100160870	eukaryota>metazoa>hexapoda	Acyrthosiphon	pisum	PREDICTED: homer protein homolog 2 [Acyrthosiphon pisum]
XP_011419455.1	411	LOC105322431	eukaryota>metazoa>mollusca	Crassostrea	gigas	PREDICTED: homer protein homolog 2 isoform X7 [Crassostrea gigas]
OQV25855.1	410	BV898_00004	eukaryota>metazoa	Hypsibius	dujardini	putative Homer protein-like protein 1 [Hypsibius dujardini]
NP_001035987.1	410	Eve-3,spred-3	eukaryota>metazoa>chordata>vertebrata	Homo	sapiens	sprouty-related, EVH1 domain-containing protein 3 [Homo sapiens]
XP_006723282.1	410	Eve-3,spred-3	eukaryota>metazoa>chordata>vertebrata	Homo	sapiens	sprouty-related, EVH1 domain-containing protein 3 isoform X1 [Homo sapiens]
XP_005560367.1	410	HOMER2	eukaryota>metazoa>chordata>vertebrata	Macaca	fascicularis	PREDICTED: homer protein homolog 2 isoform X1 [Macaca fascicularis]
XP_005560369.1	410	HOMER2	eukaryota>metazoa>chordata>vertebrata	Macaca	fascicularis	PREDICTED: homer protein homolog 2 isoform X1 [Macaca fascicularis]
XP_005560370.1	410	HOMER2	eukaryota>metazoa>chordata>vertebrata	Macaca	fascicularis	PREDICTED: homer protein homolog 2 isoform X1 [Macaca fascicularis]
NP_277058.1	410	C79158	eukaryota>metazoa>chordata>vertebrata	Mus	musculus	sprouty-related, EVH1 domain-containing protein 2 [Mus musculus]
XP_006514520.1	410	C79158	eukaryota>metazoa>chordata>vertebrata	Mus	musculus	PREDICTED: sprouty-related, EVH1 domain-containing protein 2 isoform X2 [Mus musculus]
XP_018120329.1	410	LOC108717624	eukaryota>metazoa>chordata>vertebrata	Xenopus	laevis	PREDICTED: sprouty-related, EVH1 domain-containing protein 2-like isoform X2 [Xenopus laevis]
XP_017321267.1	410	vasp	eukaryota>metazoa>chordata>vertebrata>actinopterygii	Ictalurus	punctatus	PREDICTED: vasodilator-stimulated phosphoprotein isoform X3 [Ictalurus punctatus]
XP_419341.3	409	SPRED2	eukaryota>metazoa>chordata>vertebrata	Gallus	gallus	sprouty-related, EVH1 domain-containing protein 2 isoform X2 [Gallus gallus]
XP_018118306.1	409	LOC101027290	eukaryota>metazoa>chordata>vertebrata	Xenopus	laevis	PREDICTED: sprouty-related, EVH1 domain-containing protein 2 isoform X3 [Xenopus laevis]
XP_017344565.1	409	LOC108276935	eukaryota>metazoa>chordata>vertebrata>actinopterygii	Ictalurus	punctatus	PREDICTED: vasodilator-stimulated phosphoprotein-like [Ictalurus punctatus]
XP_018087248.1	409	LOC100036872	eukaryota>metazoa>chordata>vertebrata	Xenopus	laevis	PREDICTED: uncharacterized protein LOC100036872 isoform X2 [Xenopus laevis]
XP_009014020.1	408	HELRODRAFT_185433	eukaryota>metazoa>annelida	Helobdella	robusta	hypothetical protein HELRODRAFT_185433 [Helobdella robusta]
NP_891557.3	408	AW047143,D130060H24Rik,Spred-3	eukaryota>metazoa>chordata>vertebrata	Mus	musculus	sprouty-related, EVH1 domain-containing protein 3 [Mus musculus]
XP_006539516.1	408	AW047143,D130060H24Rik,Spred-3	eukaryota>metazoa>chordata>vertebrata	Mus	musculus	PREDICTED: sprouty-related, EVH1 domain-containing protein 3 isoform X3 [Mus musculus]
XP_006539517.1	408	AW047143,D130060H24Rik,Spred-3	eukaryota>metazoa>chordata>vertebrata	Mus	musculus	PREDICTED: sprouty-related, EVH1 domain-containing protein 3 isoform X3 [Mus musculus]
XP_006539518.1	408	AW047143,D130060H24Rik,Spred-3	eukaryota>metazoa>chordata>vertebrata	Mus	musculus	PREDICTED: sprouty-related, EVH1 domain-containing protein 3 isoform X3 [Mus musculus]
XP_006539519.1	408	AW047143,D130060H24Rik,Spred-3	eukaryota>metazoa>chordata>vertebrata	Mus	musculus	PREDICTED: sprouty-related, EVH1 domain-containing protein 3 isoform X3 [Mus musculus]
XP_006514521.1	407	C79158	eukaryota>metazoa>chordata>vertebrata	Mus	musculus	PREDICTED: sprouty-related, EVH1 domain-containing protein 2 isoform X3 [Mus musculus]
XP_002675276.1	407	NAEGRDRAFT_69609	eukaryota>heterolobosea	Naegleria	gruberi strain NEG-M	predicted protein [Naegleria gruberi strain NEG-M]
XP_018087658.1	406	evl	eukaryota>metazoa>chordata>vertebrata	Xenopus	laevis	PREDICTED: enah/Vasp-like isoform X3 [Xenopus laevis]
KXJ22208.1	406	VASP	eukaryota>metazoa>cnidaria	Exaiptasia	pallida	Vasodilator-stimulated phosphoprotein [Exaiptasia pallida]
XP_011419456.1	406	LOC105322431	eukaryota>metazoa>mollusca	Crassostrea	gigas	PREDICTED: homer protein homolog 2 isoform X8 [Crassostrea gigas]
XP_012754311.1	406	SAMD00019534_062590	eukaryota>amoebozoa>mycetozoa>dictyosteliida	Acytostelium	subglobosum LB1	hypothetical protein SAMD00019534_062590 [Acytostelium subglobosum LB1]
XP_002111221.1	406	TRIADDRAFT_54960	eukaryota>metazoa>placozoa	Trichoplax	adhaerens	hypothetical protein TRIADDRAFT_54960 [Trichoplax adhaerens]
NP_001121277.1	405	spred1,Xspred1,Xtspred1	eukaryota>metazoa>chordata>vertebrata	Xenopus	laevis	sprouty related, EVH1 domain containing 1 L homeolog [Xenopus laevis]
XP_003975188.1	405	LOC101074212	eukaryota>metazoa>chordata>vertebrata>actinopterygii	Takifugu	rubripes	PREDICTED: homer protein homolog 1-like [Takifugu rubripes]
XP_018084659.1	404	xevl	eukaryota>metazoa>chordata>vertebrata	Xenopus	laevis	PREDICTED: ena/VASP-like protein isoform X8 [Xenopus laevis]
NP_001260978.1	404	AE33,CG10155,Dm,AE33,Dmel\CG10155,dSPRED,spred	eukaryota>metazoa>hexapoda	Drosophila	melanogaster	Sprouty-related protein with EVH-1 domain, isoform B [Drosophila melanogaster]
EPB89171.1	404	HMPREF1544_04038	eukaryota>fungi	Mucor	circinelloides f. circinelloides 1006PhL	hypothetical protein HMPREF1544_04038 [Mucor circinelloides f. circinelloides 1006PhL]
XP_015142959.1	403	EVL	eukaryota>metazoa>chordata>vertebrata	Gallus	gallus	ena/VASP-like protein isoform X4 [Gallus gallus]
XP_005562257.1	403	EVL	eukaryota>metazoa>chordata>vertebrata	Macaca	fascicularis	PREDICTED: ena/VASP-like protein isoform X6 [Macaca fascicularis]
XP_021324059.1	403	zgc:77220	eukaryota>metazoa>chordata>vertebrata>actinopterygii	Danio	rerio	ena/VASP-like protein isoform X1 [Danio rerio]
XP_002414876.1	402	IscW_ISCW014242	eukaryota>metazoa	Ixodes	scapularis	sprouty protein evh1 domain-containing protein, putative, partial [Ixodes scapularis]
XP_005158856.1	402	Evl,id:ibd2260,zgc:65883,zgc:77388	eukaryota>metazoa>chordata>vertebrata>actinopterygii	Danio	rerio	ena/VASP-like protein isoform X9 [Danio rerio]
XP_015754692.1	402	LOC107334274	eukaryota>metazoa>cnidaria	Acropora	digitifera	PREDICTED: homer protein homolog 2-like [Acropora digitifera]
XP_015142960.1	401	EVL	eukaryota>metazoa>chordata>vertebrata	Gallus	gallus	ena/VASP-like protein isoform X5 [Gallus gallus]
NP_001156868.1	401	AI528774,b2b2600Clo	eukaryota>metazoa>chordata>vertebrata	Mus	musculus	ena/VASP-like protein isoform 4 [Mus musculus]
XP_008200146.1	401	LOC657689	eukaryota>metazoa>hexapoda	Tribolium	castaneum	PREDICTED: homer protein homolog 2 [Tribolium castaneum]
XP_011419457.1	401	LOC105322431	eukaryota>metazoa>mollusca	Crassostrea	gigas	PREDICTED: homer protein homolog 2 isoform X9 [Crassostrea gigas]
XP_003286406.1	401	DICPUDRAFT_87126	eukaryota>amoebozoa>mycetozoa>dictyosteliida	Dictyostelium	purpureum	hypothetical protein DICPUDRAFT_87126 [Dictyostelium purpureum]
XP_006813421.1	400	LOC100313739	eukaryota>metazoa>hemichordata	Saccoglossus	kowalevskii	PREDICTED: sprouty-like protein isoform X1 [Saccoglossus kowalevskii]
NP_001260151.1	400	BEST:LD03829,CG11324,dhom,Dhom,Dhomer,Dm,Homer,Dmel\CG11324,Dvh,Hom,Homer,LD03829	eukaryota>metazoa>hexapoda	Drosophila	melanogaster	homer, isoform G [Drosophila melanogaster]
XP_011419458.1	400	LOC105322431	eukaryota>metazoa>mollusca	Crassostrea	gigas	PREDICTED: homer protein homolog 2 isoform X10 [Crassostrea gigas]
XP_005560371.1	399	HOMER2	eukaryota>metazoa>chordata>vertebrata	Macaca	fascicularis	PREDICTED: homer protein homolog 2 isoform X2 [Macaca fascicularis]
NP_001156867.1	399	AI528774,b2b2600Clo	eukaryota>metazoa>chordata>vertebrata	Mus	musculus	ena/VASP-like protein isoform 3 [Mus musculus]
XP_011615889.1	399	LOC101079576	eukaryota>metazoa>chordata>vertebrata>actinopterygii	Takifugu	rubripes	PREDICTED: ena/VASP-like protein isoform X1 [Takifugu rubripes]
XP_014772474.1	399	LOC106870788	eukaryota>metazoa>mollusca	Octopus	bimaculoides	PREDICTED: homer protein homolog 2-like isoform X1 [Octopus bimaculoides]
XP_628937.1	399	wasA	eukaryota>amoebozoa>mycetozoa>dictyosteliida	Dictyostelium	discoideum AX4	Wiscott-Aldrich syndrome protein [Dictyostelium discoideum AX4]
XP_018084660.1	398	xevl	eukaryota>metazoa>chordata>vertebrata	Xenopus	laevis	PREDICTED: ena/VASP-like protein isoform X9 [Xenopus laevis]
XP_018087659.1	398	evl	eukaryota>metazoa>chordata>vertebrata	Xenopus	laevis	PREDICTED: enah/Vasp-like isoform X4 [Xenopus laevis]
NP_001002496.3	398	homer1,zgc:100854,zgc:92844	eukaryota>metazoa>chordata>vertebrata>actinopterygii	Danio	rerio	homer protein homolog 1 isoform 1 [Danio rerio]
XP_017306767.1	398	LOC108255354	eukaryota>metazoa>chordata>vertebrata>actinopterygii	Ictalurus	punctatus	PREDICTED: homer protein homolog 1-like isoform X1 [Ictalurus punctatus]
XP_011609330.1	397	LOC101062854	eukaryota>metazoa>chordata>vertebrata>actinopterygii	Takifugu	rubripes	PREDICTED: vasodilator-stimulated phosphoprotein-like [Takifugu rubripes]
NP_001260152.1	397	BEST:LD03829,CG11324,dhom,Dhom,Dhomer,Dm,Homer,Dmel\CG11324,Dvh,Hom,Homer,LD03829	eukaryota>metazoa>hexapoda	Drosophila	melanogaster	homer, isoform F [Drosophila melanogaster]
NP_723207.1	397	BEST:LD03829,CG11324,dhom,Dhom,Dhomer,Dm,Homer,Dmel\CG11324,Dvh,Hom,Homer,LD03829	eukaryota>metazoa>hexapoda	Drosophila	melanogaster	homer, isoform B [Drosophila melanogaster]
XP_012365617.1	396	SPRED3	eukaryota>metazoa>chordata>vertebrata	Nomascus	leucogenys	PREDICTED: sprouty-related, EVH1 domain-containing protein 3 [Nomascus leucogenys]
XP_012750741.1	396	SAMD00019534_093620	eukaryota>amoebozoa>mycetozoa>dictyosteliida	Acytostelium	subglobosum LB1	hypothetical protein SAMD00019534_093620 [Acytostelium subglobosum LB1]
XP_013791218.2	394	LOC106475067	eukaryota>metazoa	Limulus	polyphemus	vasodilator-stimulated phosphoprotein-like isoform X1 [Limulus polyphemus]
NP_001260150.1	394	BEST:LD03829,CG11324,dhom,Dhom,Dhomer,Dm,Homer,Dmel\CG11324,Dvh,Hom,Homer,LD03829	eukaryota>metazoa>hexapoda	Drosophila	melanogaster	homer, isoform D [Drosophila melanogaster]
NP_477396.1	394	BEST:LD03829,CG11324,dhom,Dhom,Dhomer,Dm,Homer,Dmel\CG11324,Dvh,Hom,Homer,LD03829	eukaryota>metazoa>hexapoda	Drosophila	melanogaster	homer, isoform A [Drosophila melanogaster]
NP_031991.3	393	AI528774,b2b2600Clo	eukaryota>metazoa>chordata>vertebrata	Mus	musculus	ena/VASP-like protein isoform 2 [Mus musculus]
EFA77317.1	393	wasA	eukaryota>amoebozoa>mycetozoa	Heterostelium	album PN500	Wiscott-Aldrich syndrome protein [Heterostelium album PN500]
EPZ33916.1	393	O9G_002479	eukaryota>fungi	Rozella	allomycis CSF55	PAK-box/P21-Rho-binding domain-containing protein [Rozella allomycis CSF55]
XP_013385142.1	392	LOC106155062	eukaryota>metazoa	Lingula	anatina	homer protein homolog 2 isoform X1 [Lingula anatina]
XP_018651340.1	391	Smp_030150	eukaryota>metazoa	Schistosoma	mansoni	putative homer [Schistosoma mansoni]
NP_998397.1	391	fb14c08,fj51c03,wu:fb14c08,wu:fj51c03,zgc:85707	eukaryota>metazoa>chordata>vertebrata>actinopterygii	Danio	rerio	sprouty-related, EVH1 domain-containing protein 1 [Danio rerio]
XP_011150923.1	391	LOC105190107	eukaryota>metazoa>hexapoda	Harpegnathos	saltator	homer protein homolog 2 isoform X3 [Harpegnathos saltator]
XP_022234992.1	390	LOC106475067	eukaryota>metazoa	Limulus	polyphemus	vasodilator-stimulated phosphoprotein-like isoform X2 [Limulus polyphemus]
XP_017311973.1	390	LOC108258102	eukaryota>metazoa>chordata>vertebrata>actinopterygii	Ictalurus	punctatus	PREDICTED: ena/VASP-like protein [Ictalurus punctatus]
XP_006559185.1	390	LOC413278	eukaryota>metazoa>hexapoda	Apis	mellifera	PREDICTED: homer protein homolog 2 isoform X2 [Apis mellifera]
XP_015833055.1	390	Spred	eukaryota>metazoa>hexapoda	Tribolium	castaneum	PREDICTED: sprouty-related protein with EVH-1 domain isoform X1 [Tribolium castaneum]
NP_955813.3	387	Evl,id:ibd2260,zgc:65883,zgc:77388	eukaryota>metazoa>chordata>vertebrata>actinopterygii	Danio	rerio	ena/VASP-like protein [Danio rerio]
NP_001161569.1	387	LOC100313636	eukaryota>metazoa>hemichordata	Saccoglossus	kowalevskii	homer-like protein [Saccoglossus kowalevskii]
NP_001085488.1	386	vasp-a,vasp-b	eukaryota>metazoa>chordata>vertebrata	Xenopus	laevis	vasodilator stimulated phosphoprotein L homeolog [Xenopus laevis]
XP_008200067.1	386	Spred	eukaryota>metazoa>hexapoda	Tribolium	castaneum	PREDICTED: sprouty-related protein with EVH-1 domain isoform X2 [Tribolium castaneum]
XP_015833056.1	386	Spred	eukaryota>metazoa>hexapoda	Tribolium	castaneum	PREDICTED: sprouty-related protein with EVH-1 domain isoform X3 [Tribolium castaneum]
XP_022238303.1	386	LOC106478900	eukaryota>metazoa	Limulus	polyphemus	neural Wiskott-Aldrich syndrome protein-like isoform X4 [Limulus polyphemus]
XP_022238304.1	386	LOC106478900	eukaryota>metazoa	Limulus	polyphemus	neural Wiskott-Aldrich syndrome protein-like isoform X4 [Limulus polyphemus]
XP_018084358.1	385	vasp-a,vasp-b	eukaryota>metazoa>chordata>vertebrata	Xenopus	laevis	PREDICTED: vasodilator-stimulated phosphoprotein L homeolog isoform X1 [Xenopus laevis]
XP_018083967.1	385	imd2,thc,thc1,was,wasp	eukaryota>metazoa>chordata>vertebrata	Xenopus	laevis	PREDICTED: wiskott-Aldrich syndrome L homeolog isoform X1 [Xenopus laevis]
XP_017348495.1	384	homer1	eukaryota>metazoa>chordata>vertebrata>actinopterygii	Ictalurus	punctatus	PREDICTED: homer protein homolog 1 isoform X1 [Ictalurus punctatus]
XP_014772475.1	384	LOC106870788	eukaryota>metazoa>mollusca	Octopus	bimaculoides	PREDICTED: homer protein homolog 2-like isoform X2 [Octopus bimaculoides]
ORX89741.1	384	K493DRAFT_266691	eukaryota>fungi	Basidiobolus	meristosporus CBS 931.73	WH1-domain-containing protein [Basidiobolus meristosporus CBS 931.73]
XP_002586630.1	384	BRAFLDRAFT_131307	eukaryota>metazoa>chordata	Branchiostoma	floridae	hypothetical protein BRAFLDRAFT_131307 [Branchiostoma floridae]
XP_014661826.1	383	LOC106804929	eukaryota>metazoa	Priapulus	caudatus	PREDICTED: homer protein homolog 2-like isoform X1 [Priapulus caudatus]
XP_014661834.1	383	LOC106804929	eukaryota>metazoa	Priapulus	caudatus	PREDICTED: homer protein homolog 2-like isoform X1 [Priapulus caudatus]
XP_014661843.1	383	LOC106804929	eukaryota>metazoa	Priapulus	caudatus	PREDICTED: homer protein homolog 2-like isoform X1 [Priapulus caudatus]
XP_015147511.1	383	HOMER2	eukaryota>metazoa>chordata>vertebrata	Gallus	gallus	homer protein homolog 2 [Gallus gallus]
XP_017348496.1	383	homer1	eukaryota>metazoa>chordata>vertebrata>actinopterygii	Ictalurus	punctatus	PREDICTED: homer protein homolog 1 isoform X2 [Ictalurus punctatus]
XP_014772476.1	383	LOC106870788	eukaryota>metazoa>mollusca	Octopus	bimaculoides	PREDICTED: homer protein homolog 2-like isoform X3 [Octopus bimaculoides]
KNE65503.1	383	AMAG_11121	eukaryota>fungi	Allomyces	macrogynus ATCC 38327	hypothetical protein AMAG_11121 [Allomyces macrogynus ATCC 38327]
EFA84533.1	382	vasP	eukaryota>amoebozoa>mycetozoa	Heterostelium	album PN500	vasodilator-stimulated phosphoprotein, partial [Heterostelium album PN500]
XP_018106222.1	382	homer2	eukaryota>metazoa>chordata>vertebrata	Xenopus	laevis	PREDICTED: homer 2 isoform X1 [Xenopus laevis]
XP_018110877.1	382	LOC108712888	eukaryota>metazoa>chordata>vertebrata	Xenopus	laevis	PREDICTED: homer protein homolog 2-like [Xenopus laevis]
NP_001164144.1	382	Spred	eukaryota>metazoa>hexapoda	Tribolium	castaneum	sprouty-related protein with EVH-1 domain [Tribolium castaneum]
XP_015297415.1	381	SPRED3	eukaryota>metazoa>chordata>vertebrata	Macaca	fascicularis	PREDICTED: sprouty-related, EVH1 domain-containing protein 3 isoform X2 [Macaca fascicularis]
OHT16464.1	381	TRFO_02736	eukaryota>parabasalia	Tritrichomonas	foetus	hypothetical protein TRFO_02736 [Tritrichomonas foetus]
XP_636196.1	380	vasP	eukaryota>amoebozoa>mycetozoa>dictyosteliida	Dictyostelium	discoideum AX4	vasodilator-stimulated phosphoprotein [Dictyostelium discoideum AX4]
NP_003361.1	380	VASP	eukaryota>metazoa>chordata>vertebrata	Homo	sapiens	vasodilator-stimulated phosphoprotein [Homo sapiens]
XP_014661850.1	379	LOC106804929	eukaryota>metazoa	Priapulus	caudatus	PREDICTED: homer protein homolog 2-like isoform X2 [Priapulus caudatus]
XP_005259256.1	379	VASP	eukaryota>metazoa>chordata>vertebrata	Homo	sapiens	vasodilator-stimulated phosphoprotein isoform X1 [Homo sapiens]
XP_005259257.1	379	VASP	eukaryota>metazoa>chordata>vertebrata	Homo	sapiens	vasodilator-stimulated phosphoprotein isoform X2 [Homo sapiens]
XP_011525280.1	379	Eve-3,spred-3	eukaryota>metazoa>chordata>vertebrata	Homo	sapiens	sprouty-related, EVH1 domain-containing protein 3 isoform X2 [Homo sapiens]
XP_017348497.1	379	homer1	eukaryota>metazoa>chordata>vertebrata>actinopterygii	Ictalurus	punctatus	PREDICTED: homer protein homolog 1 isoform X3 [Ictalurus punctatus]
XP_016882689.1	378	VASP	eukaryota>metazoa>chordata>vertebrata	Homo	sapiens	vasodilator-stimulated phosphoprotein isoform X3 [Homo sapiens]
CAG00769.1	378	GSTEN:00019301:G:001	eukaryota>metazoa>chordata>vertebrata>actinopterygii	Tetraodon	nigroviridis	unnamed protein product, partial [Tetraodon nigroviridis]
XP_011610233.1	378	LOC101061290	eukaryota>metazoa>chordata>vertebrata>actinopterygii	Takifugu	rubripes	PREDICTED: ena/VASP-like protein isoform X1 [Takifugu rubripes]
XP_011613351.1	378	homer3	eukaryota>metazoa>chordata>vertebrata>actinopterygii	Takifugu	rubripes	PREDICTED: homer protein homolog 3 isoform X1 [Takifugu rubripes]
XP_012552139.1	378	LOC101740566	eukaryota>metazoa>hexapoda	Bombyx	mori	homer protein homolog 2 isoform X1 [Bombyx mori]
XP_011615898.1	377	LOC101079576	eukaryota>metazoa>chordata>vertebrata>actinopterygii	Takifugu	rubripes	PREDICTED: ena/VASP-like protein isoform X3 [Takifugu rubripes]
KXJ10164.1	377	Homer2	eukaryota>metazoa>cnidaria	Exaiptasia	pallida	Homer protein-like 2 [Exaiptasia pallida]
KXJ16109.1	377	Homer2	eukaryota>metazoa>cnidaria	Exaiptasia	pallida	Homer protein-like 2 [Exaiptasia pallida]
XP_004933593.1	377	LOC101740566	eukaryota>metazoa>hexapoda	Bombyx	mori	homer protein homolog 2 isoform X2 [Bombyx mori]
XP_013754726.1	376	AMSG_09341	eukaryota>apusozoa>apusomonadidae	Thecamonas	trahens ATCC 50062	hypothetical protein AMSG_09341 [Thecamonas trahens ATCC 50062]
NP_991196.1	376	zgc:77220	eukaryota>metazoa>chordata>vertebrata>actinopterygii	Danio	rerio	ena/VASP-like protein [Danio rerio]
NP_033525.2	375	Vasp	eukaryota>metazoa>chordata>vertebrata	Mus	musculus	vasodilator-stimulated phosphoprotein isoform 1 [Mus musculus]
NP_001268950.1	374	Vasp	eukaryota>metazoa>chordata>vertebrata	Mus	musculus	vasodilator-stimulated phosphoprotein isoform 2 [Mus musculus]
XP_013385143.1	372	LOC106155062	eukaryota>metazoa	Lingula	anatina	homer protein homolog 2 isoform X2 [Lingula anatina]
CAG10404.1	372	GSTEN:00032026:G:001	eukaryota>metazoa>chordata>vertebrata>actinopterygii	Tetraodon	nigroviridis	unnamed protein product [Tetraodon nigroviridis]
XP_011615904.1	371	LOC101079576	eukaryota>metazoa>chordata>vertebrata>actinopterygii	Takifugu	rubripes	PREDICTED: ena/VASP-like protein isoform X4 [Takifugu rubripes]
XP_011535130.1	370	RNB6	eukaryota>metazoa>chordata>vertebrata	Homo	sapiens	ena/VASP-like protein isoform X3 [Homo sapiens]
NP_687036.1	370	homer-1,PSD-Zip45,SYN47,Ves-1,vesl-1	eukaryota>metazoa>chordata>vertebrata	Mus	musculus	homer protein homolog 1 isoform d [Mus musculus]
XP_003275888.1	370	HOMER3	eukaryota>metazoa>chordata>vertebrata	Nomascus	leucogenys	PREDICTED: homer protein homolog 3 [Nomascus leucogenys]
XP_017348499.1	370	homer1	eukaryota>metazoa>chordata>vertebrata>actinopterygii	Ictalurus	punctatus	PREDICTED: homer protein homolog 1 isoform X4 [Ictalurus punctatus]
XP_015833057.1	370	Spred	eukaryota>metazoa>hexapoda	Tribolium	castaneum	PREDICTED: sprouty-related protein with EVH-1 domain isoform X4 [Tribolium castaneum]
XP_011679722.1	369	LOC583805	eukaryota>metazoa>echinodermata	Strongylocentrotus	purpuratus	PREDICTED: homer protein homolog 2 isoform X1 [Strongylocentrotus purpuratus]
XP_013385144.1	368	LOC106155062	eukaryota>metazoa	Lingula	anatina	homer protein homolog 2 isoform X3 [Lingula anatina]
XP_003287187.1	367	DICPUDRAFT_91862	eukaryota>amoebozoa>mycetozoa>dictyosteliida	Dictyostelium	purpureum	hypothetical protein DICPUDRAFT_91862 [Dictyostelium purpureum]
XP_015297148.1	367	VASP	eukaryota>metazoa>chordata>vertebrata	Macaca	fascicularis	PREDICTED: vasodilator-stimulated phosphoprotein isoform X1 [Macaca fascicularis]
XP_017332345.1	367	LOC108270303	eukaryota>metazoa>chordata>vertebrata>actinopterygii	Ictalurus	punctatus	PREDICTED: ena/VASP-like protein isoform X6 [Ictalurus punctatus]
XP_015297149.1	366	VASP	eukaryota>metazoa>chordata>vertebrata	Macaca	fascicularis	PREDICTED: vasodilator-stimulated phosphoprotein isoform X2 [Macaca fascicularis]
XP_015297150.1	366	VASP	eukaryota>metazoa>chordata>vertebrata	Macaca	fascicularis	PREDICTED: vasodilator-stimulated phosphoprotein isoform X3 [Macaca fascicularis]
NP_671705.2	366	homer-1,PSD-Zip45,SYN47,Ves-1,vesl-1	eukaryota>metazoa>chordata>vertebrata	Mus	musculus	homer protein homolog 1 isoform 1 [Mus musculus]
XP_006509742.1	366	AW146114	eukaryota>metazoa>chordata>vertebrata	Mus	musculus	PREDICTED: homer protein homolog 3 isoform X1 [Mus musculus]
XP_006509743.1	366	AW146114	eukaryota>metazoa>chordata>vertebrata	Mus	musculus	PREDICTED: homer protein homolog 3 isoform X1 [Mus musculus]
XP_006509744.1	366	AW146114	eukaryota>metazoa>chordata>vertebrata	Mus	musculus	PREDICTED: homer protein homolog 3 isoform X2 [Mus musculus]
XP_017332346.1	366	LOC108270303	eukaryota>metazoa>chordata>vertebrata>actinopterygii	Ictalurus	punctatus	PREDICTED: ena/VASP-like protein isoform X7 [Ictalurus punctatus]
XP_017332347.1	366	LOC108270303	eukaryota>metazoa>chordata>vertebrata>actinopterygii	Ictalurus	punctatus	PREDICTED: ena/VASP-like protein isoform X8 [Ictalurus punctatus]
XP_015297151.1	365	VASP	eukaryota>metazoa>chordata>vertebrata	Macaca	fascicularis	PREDICTED: vasodilator-stimulated phosphoprotein isoform X4 [Macaca fascicularis]
NP_001268951.1	365	Vasp	eukaryota>metazoa>chordata>vertebrata	Mus	musculus	vasodilator-stimulated phosphoprotein isoform 3 [Mus musculus]
XP_011610234.1	365	LOC101061290	eukaryota>metazoa>chordata>vertebrata>actinopterygii	Takifugu	rubripes	PREDICTED: ena/VASP-like protein isoform X2 [Takifugu rubripes]
XP_017332348.1	365	LOC108270303	eukaryota>metazoa>chordata>vertebrata>actinopterygii	Ictalurus	punctatus	PREDICTED: ena/VASP-like protein isoform X9 [Ictalurus punctatus]
XP_009860149.1	364	LOC101243497	eukaryota>metazoa>chordata	Ciona	intestinalis	PREDICTED: protein enabled-like [Ciona intestinalis]
XP_006720838.1	364	ACPD,CPD,DFNA68,HOMER-2,VESL-2	eukaryota>metazoa>chordata>vertebrata	Homo	sapiens	homer protein homolog 2 isoform X1 [Homo sapiens]
XP_011520533.1	364	ACPD,CPD,DFNA68,HOMER-2,VESL-2	eukaryota>metazoa>chordata>vertebrata	Homo	sapiens	homer protein homolog 2 isoform X1 [Homo sapiens]
XP_011520534.1	364	ACPD,CPD,DFNA68,HOMER-2,VESL-2	eukaryota>metazoa>chordata>vertebrata	Homo	sapiens	homer protein homolog 2 isoform X1 [Homo sapiens]
XP_017351183.1	364	LOC108280570	eukaryota>metazoa>chordata>vertebrata>actinopterygii	Ictalurus	punctatus	PREDICTED: homer protein homolog 3-like isoform X1 [Ictalurus punctatus]
XP_012756217.1	364	SAMD00019534_042580	eukaryota>amoebozoa>mycetozoa>dictyosteliida	Acytostelium	subglobosum LB1	hypothetical protein SAMD00019534_042580, partial [Acytostelium subglobosum LB1]
XP_011520535.1	362	ACPD,CPD,DFNA68,HOMER-2,VESL-2	eukaryota>metazoa>chordata>vertebrata	Homo	sapiens	homer protein homolog 2 isoform X2 [Homo sapiens]
XP_003723345.1	362	LOC583805	eukaryota>metazoa>echinodermata	Strongylocentrotus	purpuratus	PREDICTED: homer protein homolog 2 isoform X2 [Strongylocentrotus purpuratus]
XP_004998981.1	361	PTSG_00432	eukaryota>choanoflagellida	Salpingoeca	rosetta	Homer3 protein [Salpingoeca rosetta]
NP_001139194.1	361	HOMER-3,VESL3	eukaryota>metazoa>chordata>vertebrata	Homo	sapiens	homer protein homolog 3 isoform 1 [Homo sapiens]
NP_004829.3	361	HOMER-3,VESL3	eukaryota>metazoa>chordata>vertebrata	Homo	sapiens	homer protein homolog 3 isoform 1 [Homo sapiens]
XP_006723006.1	361	HOMER-3,VESL3	eukaryota>metazoa>chordata>vertebrata	Homo	sapiens	homer protein homolog 3 isoform X1 [Homo sapiens]
XP_006723007.1	361	HOMER-3,VESL3	eukaryota>metazoa>chordata>vertebrata	Homo	sapiens	homer protein homolog 3 isoform X1 [Homo sapiens]
XP_005588547.1	361	HOMER3	eukaryota>metazoa>chordata>vertebrata	Macaca	fascicularis	PREDICTED: homer protein homolog 3 isoform X1 [Macaca fascicularis]
XP_015295836.1	361	HOMER3	eukaryota>metazoa>chordata>vertebrata	Macaca	fascicularis	PREDICTED: homer protein homolog 3 isoform X1 [Macaca fascicularis]
XP_015295837.1	361	HOMER3	eukaryota>metazoa>chordata>vertebrata	Macaca	fascicularis	PREDICTED: homer protein homolog 3 isoform X1 [Macaca fascicularis]
XP_017351184.1	361	LOC108280570	eukaryota>metazoa>chordata>vertebrata>actinopterygii	Ictalurus	punctatus	PREDICTED: homer protein homolog 3-like isoform X2 [Ictalurus punctatus]
XP_002407576.1	360	IscW_ISCW001721	eukaryota>metazoa	Ixodes	scapularis	homer, putative [Ixodes scapularis]
NP_001313219.1	360	homer1,zgc:100854,zgc:92844	eukaryota>metazoa>chordata>vertebrata>actinopterygii	Danio	rerio	homer protein homolog 1 isoform 2 [Danio rerio]
NP_957407.1	360	homer3,zgc:55557	eukaryota>metazoa>chordata>vertebrata>actinopterygii	Danio	rerio	homer protein homolog 3 [Danio rerio]
XP_017306769.1	360	LOC108255354	eukaryota>metazoa>chordata>vertebrata>actinopterygii	Ictalurus	punctatus	PREDICTED: homer protein homolog 1-like isoform X2 [Ictalurus punctatus]
XP_009010103.1	360	HELRODRAFT_71732	eukaryota>metazoa>annelida	Helobdella	robusta	hypothetical protein HELRODRAFT_71732, partial [Helobdella robusta]
XP_022240270.1	359	LOC106458378	eukaryota>metazoa	Limulus	polyphemus	homer protein homolog 2-like isoform X2 [Limulus polyphemus]
XP_022240271.1	359	LOC106458378	eukaryota>metazoa	Limulus	polyphemus	homer protein homolog 2-like isoform X2 [Limulus polyphemus]
NP_001139625.1	359	AW146114	eukaryota>metazoa>chordata>vertebrata	Mus	musculus	homer protein homolog 3 isoform 1 [Mus musculus]
XP_788792.2	359	LOC583805	eukaryota>metazoa>echinodermata	Strongylocentrotus	purpuratus	PREDICTED: homer protein homolog 2 isoform X3 [Strongylocentrotus purpuratus]
XP_002129074.1	358	LOC100184645	eukaryota>metazoa>chordata	Ciona	intestinalis	PREDICTED: homer protein homolog 2 [Ciona intestinalis]
NP_001139193.1	358	HOMER-3,VESL3	eukaryota>metazoa>chordata>vertebrata	Homo	sapiens	homer protein homolog 3 isoform 2 [Homo sapiens]
XP_015308681.1	358	HOMER2	eukaryota>metazoa>chordata>vertebrata	Macaca	fascicularis	PREDICTED: homer protein homolog 2 isoform X3 [Macaca fascicularis]
XP_011613352.1	358	homer3	eukaryota>metazoa>chordata>vertebrata>actinopterygii	Takifugu	rubripes	PREDICTED: homer protein homolog 3 isoform X2 [Takifugu rubripes]
XP_017333253.1	358	LOC108270816	eukaryota>metazoa>chordata>vertebrata>actinopterygii	Ictalurus	punctatus	PREDICTED: homer protein homolog 3-like [Ictalurus punctatus]
CDS42425.1	357	EmuJ_001013500	eukaryota>metazoa	Echinococcus	multilocularis	EVH1 [Echinococcus multilocularis]
NP_001080624.1	357	homer-3,homer3,vesl3	eukaryota>metazoa>chordata>vertebrata	Xenopus	laevis	homer scaffold protein 3 L homeolog [Xenopus laevis]
NP_001084666.1	357	homer-3,homer3,vesl3	eukaryota>metazoa>chordata>vertebrata	Xenopus	laevis	homer scaffold protein 3 S homeolog [Xenopus laevis]
XP_018095318.1	357	homer3	eukaryota>metazoa>chordata>vertebrata	Xenopus	laevis	PREDICTED: homer homolog 3 isoform X1 [Xenopus laevis]
XP_017348500.1	357	homer1	eukaryota>metazoa>chordata>vertebrata>actinopterygii	Ictalurus	punctatus	PREDICTED: homer protein homolog 1 isoform X5 [Ictalurus punctatus]
XP_022243428.1	356	LOC106460914	eukaryota>metazoa	Limulus	polyphemus	homer protein homolog 2-like isoform X3 [Limulus polyphemus]
XP_015155617.1	356	HOMER3	eukaryota>metazoa>chordata>vertebrata	Gallus	gallus	homer protein homolog 3 isoform X3 [Gallus gallus]
NP_036114.2	356	AW146114	eukaryota>metazoa>chordata>vertebrata	Mus	musculus	homer protein homolog 3 isoform 2 [Mus musculus]
XP_003978172.1	356	LOC101063326	eukaryota>metazoa>chordata>vertebrata>actinopterygii	Takifugu	rubripes	PREDICTED: homer protein homolog 1-like [Takifugu rubripes]
XP_011617735.1	356	LOC101063326	eukaryota>metazoa>chordata>vertebrata>actinopterygii	Takifugu	rubripes	PREDICTED: homer protein homolog 1-like [Takifugu rubripes]
XP_001637685.1	356	NEMVEDRAFT_v1g91920	eukaryota>metazoa>cnidaria	Nematostella	vectensis	predicted protein [Nematostella vectensis]
XP_424768.3	355	HOMER1	eukaryota>metazoa>chordata>vertebrata	Gallus	gallus	homer protein homolog 1 [Gallus gallus]
NP_001089052.1	355	homer,homer1,xhomer1	eukaryota>metazoa>chordata>vertebrata	Xenopus	laevis	homer scaffold protein 1 L homeolog [Xenopus laevis]
XP_018099953.1	355	LOC108707277	eukaryota>metazoa>chordata>vertebrata	Xenopus	laevis	PREDICTED: homer protein homolog 1 [Xenopus laevis]
XP_018099954.1	355	LOC108707277	eukaryota>metazoa>chordata>vertebrata	Xenopus	laevis	PREDICTED: homer protein homolog 1 [Xenopus laevis]
XP_018104799.1	355	homer,homer1,xhomer1	eukaryota>metazoa>chordata>vertebrata	Xenopus	laevis	PREDICTED: homer scaffolding protein 1 L homeolog isoform X1 [Xenopus laevis]
XP_011608343.1	355	homer2	eukaryota>metazoa>chordata>vertebrata>actinopterygii	Takifugu	rubripes	PREDICTED: homer protein homolog 2 isoform X1 [Takifugu rubripes]
NP_004263.1	354	HOMER,HOMER1A,HOMER1B,HOMER1C,SYN47,Ves-1	eukaryota>metazoa>chordata>vertebrata	Homo	sapiens	homer protein homolog 1 isoform 1 [Homo sapiens]
NP_955362.1	354	ACPD,CPD,DFNA68,HOMER-2,VESL-2	eukaryota>metazoa>chordata>vertebrata	Homo	sapiens	homer protein homolog 2 isoform 2 [Homo sapiens]
XP_006720839.1	354	ACPD,CPD,DFNA68,HOMER-2,VESL-2	eukaryota>metazoa>chordata>vertebrata	Homo	sapiens	homer protein homolog 2 isoform X3 [Homo sapiens]
XP_015307117.1	354	HOMER1	eukaryota>metazoa>chordata>vertebrata	Macaca	fascicularis	PREDICTED: homer protein homolog 1 isoform X1 [Macaca fascicularis]
NP_001271118.1	354	homer-1,PSD-Zip45,SYN47,Ves-1,vesl-1	eukaryota>metazoa>chordata>vertebrata	Mus	musculus	homer protein homolog 1 isoform b [Mus musculus]
NP_036113.1	354	9330120H11Rik,AW539445,CPD,Vesl-2	eukaryota>metazoa>chordata>vertebrata	Mus	musculus	homer protein homolog 2 isoform 1 [Mus musculus]
XP_003261566.1	354	HOMER1	eukaryota>metazoa>chordata>vertebrata	Nomascus	leucogenys	PREDICTED: homer protein homolog 1 isoform X1 [Nomascus leucogenys]
XP_003969915.1	354	homer2	eukaryota>metazoa>chordata>vertebrata>actinopterygii	Takifugu	rubripes	PREDICTED: homer protein homolog 2 isoform X2 [Takifugu rubripes]
XP_011608344.1	354	homer2	eukaryota>metazoa>chordata>vertebrata>actinopterygii	Takifugu	rubripes	PREDICTED: homer protein homolog 2 isoform X3 [Takifugu rubripes]
XP_418233.1	353	HOMER3	eukaryota>metazoa>chordata>vertebrata	Gallus	gallus	homer protein homolog 3 isoform X4 [Gallus gallus]
XP_005272505.1	353	ACPD,CPD,DFNA68,HOMER-2,VESL-2	eukaryota>metazoa>chordata>vertebrata	Homo	sapiens	homer protein homolog 2 isoform X4 [Homo sapiens]
XP_012561566.1	353	LOC100208831	eukaryota>metazoa>cnidaria	Hydra	vulgaris	PREDICTED: ena/VASP-like protein [Hydra vulgaris]
XP_022257326.1	352	LOC106473120	eukaryota>metazoa	Limulus	polyphemus	protein enabled homolog, partial [Limulus polyphemus]
CAF99806.1	352	GSTEN:00018023:G:001	eukaryota>metazoa>chordata>vertebrata>actinopterygii	Tetraodon	nigroviridis	unnamed protein product, partial [Tetraodon nigroviridis]
XP_011150924.1	351	LOC105190107	eukaryota>metazoa>hexapoda	Harpegnathos	saltator	PREDICTED: homer protein homolog 1 isoform X3 [Harpegnathos saltator]
CAG01973.1	351	GSTEN:00020925:G:001	eukaryota>metazoa>chordata>vertebrata>actinopterygii	Tetraodon	nigroviridis	unnamed protein product, partial [Tetraodon nigroviridis]
XP_012352108.1	350	VASP	eukaryota>metazoa>chordata>vertebrata	Nomascus	leucogenys	PREDICTED: vasodilator-stimulated phosphoprotein [Nomascus leucogenys]
XP_017321575.1	350	homer2	eukaryota>metazoa>chordata>vertebrata>actinopterygii	Ictalurus	punctatus	PREDICTED: homer protein homolog 2 isoform X1 [Ictalurus punctatus]
XP_017321576.1	350	homer2	eukaryota>metazoa>chordata>vertebrata>actinopterygii	Ictalurus	punctatus	PREDICTED: homer protein homolog 2 isoform X1 [Ictalurus punctatus]
XP_017321577.1	350	homer2	eukaryota>metazoa>chordata>vertebrata>actinopterygii	Ictalurus	punctatus	PREDICTED: homer protein homolog 2 isoform X1 [Ictalurus punctatus]
XP_017321578.1	350	homer2	eukaryota>metazoa>chordata>vertebrata>actinopterygii	Ictalurus	punctatus	PREDICTED: homer protein homolog 2 isoform X1 [Ictalurus punctatus]
XP_017321579.1	349	homer2	eukaryota>metazoa>chordata>vertebrata>actinopterygii	Ictalurus	punctatus	PREDICTED: homer protein homolog 2 isoform X2 [Ictalurus punctatus]
XP_011614836.1	347	LOC101067063	eukaryota>metazoa>chordata>vertebrata>actinopterygii	Takifugu	rubripes	PREDICTED: homer protein homolog 3-like [Takifugu rubripes]
ELR20892.1	345	ACA1_278330	eukaryota>amoebozoa>acanthamoebidae	Acanthamoeba	castellanii str. Neff	WH1 domain containing protein [Acanthamoeba castellanii str. Neff]
NP_004830.2	343	ACPD,CPD,DFNA68,HOMER-2,VESL-2	eukaryota>metazoa>chordata>vertebrata	Homo	sapiens	homer protein homolog 2 isoform 1 [Homo sapiens]
XP_005272506.1	343	ACPD,CPD,DFNA68,HOMER-2,VESL-2	eukaryota>metazoa>chordata>vertebrata	Homo	sapiens	homer protein homolog 2 isoform X5 [Homo sapiens]
NP_001157558.1	343	9330120H11Rik,AW539445,CPD,Vesl-2	eukaryota>metazoa>chordata>vertebrata	Mus	musculus	homer protein homolog 2 isoform 2 [Mus musculus]
XP_002473857.1	341	POSPLDRAFT_105134	eukaryota>fungi>basidiomycota	Postia	placenta Mad-698-R	predicted protein [Postia placenta Mad-698-R]
XP_018089244.1	340	LOC108700560	eukaryota>metazoa>chordata>vertebrata	Xenopus	laevis	PREDICTED: vasodilator-stimulated phosphoprotein-like [Xenopus laevis]
XP_012353605.1	337	EVL	eukaryota>metazoa>chordata>vertebrata	Nomascus	leucogenys	PREDICTED: ena/VASP-like protein [Nomascus leucogenys]
CBY12821.1	336	GSOID_T00002881001	eukaryota>metazoa>chordata	Oikopleura	dioica	unnamed protein product [Oikopleura dioica]
XP_011248712.2	335	AW047143,D130060H24Rik,Spred-3	eukaryota>metazoa>chordata>vertebrata	Mus	musculus	PREDICTED: sprouty-related, EVH1 domain-containing protein 3 isoform X4 [Mus musculus]
XP_013404861.1	335	LOC106169792	eukaryota>metazoa	Lingula	anatina	PREDICTED: neural Wiskott-Aldrich syndrome protein-like, partial [Lingula anatina]
XP_002411260.1	334	IscW_ISCW008989	eukaryota>metazoa	Ixodes	scapularis	vasodilator-stimulated phosphoprotein, putative, partial [Ixodes scapularis]

CDS40850.1	331	EmuJ_000845000	eukaryota>metazoa	Echinococcus	multilocularis	wiskott Aldrich syndrome protein [Echinococcus multilocularis]
XP_011520536.1	330	ACPD,CPD,DFNA68,HOMER-2,VESL-2	eukaryota>metazoa>chordata>vertebrata	Homo	sapiens	homer protein homolog 2 isoform X6 [Homo sapiens]
NP_001139196.1	325	HOMER-3,VESL3	eukaryota>metazoa>chordata>vertebrata	Homo	sapiens	homer protein homolog 3 isoform 3 [Homo sapiens]
XP_015295838.1	325	HOMER3	eukaryota>metazoa>chordata>vertebrata	Macaca	fascicularis	PREDICTED: homer protein homolog 3 isoform X2 [Macaca fascicularis]
NP_001300273.1	324	unc-34	eukaryota>metazoa>nematoda	Caenorhabditis	elegans	Uncharacterized protein CELE_Y50D4C.1 [Caenorhabditis elegans]
XP_014156175.1	324	SARC_05445	eukaryota>ichthyosporea	Sphaeroforma	arctica JP610	hypothetical protein SARC_05445 [Sphaeroforma arctica JP610]
XP_012353371.1	320	HOMER2	eukaryota>metazoa>chordata>vertebrata	Nomascus	leucogenys	PREDICTED: LOW QUALITY PROTEIN: homer protein homolog 2 [Nomascus leucogenys]
CBY32835.1	318	GSOID_T00032191001	eukaryota>metazoa>chordata	Oikopleura	dioica	unnamed protein product [Oikopleura dioica]
XP_001850671.1	317	CpipJ_CPIJ009183	eukaryota>metazoa>hexapoda	Culex	quinquefasciatus	homer [Culex quinquefasciatus]
NP_001018470.1	314	zgc:111960,zmp:0000000677	eukaryota>metazoa>chordata>vertebrata>actinopterygii	Danio	rerio	homer protein homolog 2 [Danio rerio]
CAG01507.1	312	GSTEN:00020297:G:001	eukaryota>metazoa>chordata>vertebrata>actinopterygii	Tetraodon	nigroviridis	unnamed protein product, partial [Tetraodon nigroviridis]
NP_001080709.1	310	homer2	eukaryota>metazoa>chordata>vertebrata	Xenopus	laevis	homer 2 [Xenopus laevis]
NP_001024254.1	310	unc-34	eukaryota>metazoa>nematoda	Caenorhabditis	elegans	Uncharacterized protein CELE_Y50D4C.1 [Caenorhabditis elegans]
XP_013382185.1	306	LOC106152976	eukaryota>metazoa	Lingula	anatina	homer protein homolog 2-like [Lingula anatina]
XP_001749347.1	304	MONBRDRAFT_28813	eukaryota>choanoflagellida	Monosiga	brevicollis MX1	hypothetical protein [Monosiga brevicollis MX1]
CBY23156.1	301	GSOID_T00015088001	eukaryota>metazoa>chordata	Oikopleura	dioica	unnamed protein product [Oikopleura dioica]
XP_001919116.3	299	si:dkey-197j19.6	eukaryota>metazoa>chordata>vertebrata>actinopterygii	Danio	rerio	proline-rich protein LAS17-like [Danio rerio]
XP_017335405.1	296	LOC108271935	eukaryota>metazoa>chordata>vertebrata>actinopterygii	Ictalurus	punctatus	PREDICTED: wiskott-Aldrich syndrome protein-like isoform X1 [Ictalurus punctatus]
XP_013759887.1	293	AMSG_03539	eukaryota>apusozoa>apusomonadidae	Thecamonas	trahens ATCC 50062	hypothetical protein AMSG_03539 [Thecamonas trahens ATCC 50062]
NP_001157559.1	290	9330120H11Rik,AW539445,CPD,Vesl-2	eukaryota>metazoa>chordata>vertebrata	Mus	musculus	homer protein homolog 2 isoform 3 [Mus musculus]
XP_006540972.1	290	9330120H11Rik,AW539445,CPD,Vesl-2	eukaryota>metazoa>chordata>vertebrata	Mus	musculus	PREDICTED: homer protein homolog 2 isoform X1 [Mus musculus]
XP_017177403.1	290	AW047143,D130060H24Rik,Spred-3	eukaryota>metazoa>chordata>vertebrata	Mus	musculus	PREDICTED: sprouty-related, EVH1 domain-containing protein 3 isoform X5 [Mus musculus]
XP_021329015.1	279	vaspb	eukaryota>metazoa>chordata>vertebrata>actinopterygii	Danio	rerio	protein enabled homolog isoform X3 [Danio rerio]
XP_001848257.1	279	CpipJ_CPIJ006699	eukaryota>metazoa>hexapoda	Culex	quinquefasciatus	wiskott-aldrich syndrome protein [Culex quinquefasciatus]
XP_022238305.1	277	LOC106478900	eukaryota>metazoa	Limulus	polyphemus	neural Wiskott-Aldrich syndrome protein-like isoform X5 [Limulus polyphemus]
XP_001846730.1	271	CpipJ_CPIJ004706	eukaryota>metazoa>hexapoda	Culex	quinquefasciatus	vasodilator-stimulated phosphoprotein [Culex quinquefasciatus]
XP_022250318.1	267	LOC106466578	eukaryota>metazoa	Limulus	polyphemus	homer protein homolog 2-like, partial [Limulus polyphemus]
XP_009497368.1	266	H696_05232	eukaryota	Fonticula	alba	hypothetical protein H696_05232 [Fonticula alba]
XP_015770466.1	262	LOC107348895	eukaryota>metazoa>cnidaria	Acropora	digitifera	PREDICTED: uncharacterized protein LOC107348895 isoform X1 [Acropora digitifera]
XP_017177404.1	261	AW047143,D130060H24Rik,Spred-3	eukaryota>metazoa>chordata>vertebrata	Mus	musculus	PREDICTED: sprouty-related, EVH1 domain-containing protein 3 isoform X6 [Mus musculus]
XP_017177405.1	261	AW047143,D130060H24Rik,Spred-3	eukaryota>metazoa>chordata>vertebrata	Mus	musculus	PREDICTED: sprouty-related, EVH1 domain-containing protein 3 isoform X7 [Mus musculus]
ORX84632.1	254	K493DRAFT_239192	eukaryota>fungi	Basidiobolus	meristosporus CBS 931.73	WH1-domain-containing protein [Basidiobolus meristosporus CBS 931.73]
EPT04783.1	253	FOMPIDRAFT_1096116	eukaryota>fungi>basidiomycota	Fomitopsis	pinicola FP-58527 SS1	hypothetical protein FOMPIDRAFT_1096116, partial [Fomitopsis pinicola FP-58527 SS1]
XP_014775879.1	250	LOC106873164	eukaryota>metazoa>mollusca	Octopus	bimaculoides	PREDICTED: vasodilator-stimulated phosphoprotein-like, partial [Octopus bimaculoides]
XP_005264257.2	248	Spred-2	eukaryota>metazoa>chordata>vertebrata	Homo	sapiens	sprouty-related, EVH1 domain-containing protein 2 isoform X2 [Homo sapiens]
XP_021324005.1	246	LOC100330028	eukaryota>metazoa>chordata>vertebrata>actinopterygii	Danio	rerio	homer protein homolog 3-like isoform X1 [Danio rerio]
XP_017207954.1	243	LOC100330028	eukaryota>metazoa>chordata>vertebrata>actinopterygii	Danio	rerio	homer protein homolog 3-like isoform X2 [Danio rerio]
XP_004365930.1	243	CAOG_01059	eukaryota>ichthyosporea	Capsaspora	owczarzaki ATCC 30864	hypothetical protein CAOG_01059, partial [Capsaspora owczarzaki ATCC 30864]
ORE11625.1	239	BCV72DRAFT_324576	eukaryota>fungi	Rhizopus	microsporus var. microsporus	WH1-domain-containing protein, partial [Rhizopus microsporus var. microsporus]
CAG07864.1	237	GSTEN:00028670:G:001	eukaryota>metazoa>chordata>vertebrata>actinopterygii	Tetraodon	nigroviridis	unnamed protein product [Tetraodon nigroviridis]
XP_009010952.1	236	HELRODRAFT_96961	eukaryota>metazoa>annelida	Helobdella	robusta	hypothetical protein HELRODRAFT_96961, partial [Helobdella robusta]
XP_015770467.1	232	LOC107348895	eukaryota>metazoa>cnidaria	Acropora	digitifera	PREDICTED: uncharacterized protein LOC107348895 isoform X2 [Acropora digitifera]
XP_021331746.1	231	homer1,zgc:100854,zgc:92844	eukaryota>metazoa>chordata>vertebrata>actinopterygii	Danio	rerio	homer protein homolog 1 isoform X1 [Danio rerio]
XP_018274847.1	231	CC85DRAFT_294450	eukaryota>fungi>basidiomycota	Cutaneotrichosporon	oleaginosum	PH domain-like protein, partial [Cutaneotrichosporon oleaginosum]
NP_001264185.1	229	5730461F13Rik,AL024345,AW047647	eukaryota>metazoa>chordata>vertebrata	Mus	musculus	sprouty-related, EVH1 domain-containing protein 1 isoform 2 [Mus musculus]
XP_009061757.1	229	LOTGIDRAFT_127578	eukaryota>metazoa>mollusca	Lottia	gigantea	hypothetical protein LOTGIDRAFT_127578, partial [Lottia gigantea]
NP_001264006.1	224	HOMER,HOMER1A,HOMER1B,HOMER1C,SYN47,Ves-1	eukaryota>metazoa>chordata>vertebrata	Homo	sapiens	homer protein homolog 1 isoform 2 [Homo sapiens]
XP_012361038.1	224	HOMER1	eukaryota>metazoa>chordata>vertebrata	Nomascus	leucogenys	PREDICTED: homer protein homolog 1 isoform X2 [Nomascus leucogenys]
XP_015770468.1	223	LOC107348895	eukaryota>metazoa>cnidaria	Acropora	digitifera	PREDICTED: uncharacterized protein LOC107348895 isoform X3 [Acropora digitifera]
XP_015770469.1	223	LOC107348895	eukaryota>metazoa>cnidaria	Acropora	digitifera	PREDICTED: uncharacterized protein LOC107348895 isoform X3 [Acropora digitifera]
XP_019856528.1	219	LOC105314031	eukaryota>metazoa	Amphimedon	queenslandica	PREDICTED: neural Wiskott-Aldrich syndrome protein-like isoform X1 [Amphimedon queenslandica]
XP_019856529.1	219	LOC105314031	eukaryota>metazoa	Amphimedon	queenslandica	PREDICTED: neural Wiskott-Aldrich syndrome protein-like isoform X1 [Amphimedon queenslandica]
XP_005264259.1	218	Spred-2	eukaryota>metazoa>chordata>vertebrata	Homo	sapiens	sprouty-related, EVH1 domain-containing protein 2 isoform X4 [Homo sapiens]
XP_017335406.1	215	LOC108271935	eukaryota>metazoa>chordata>vertebrata>actinopterygii	Ictalurus	punctatus	PREDICTED: wiskott-Aldrich syndrome protein-like isoform X2 [Ictalurus punctatus]
ESA14394.1	210	GLOINDRAFT_171470	eukaryota>fungi	Rhizophagus	irregularis DAOM 181602	hypothetical protein GLOINDRAFT_171470 [Rhizophagus irregularis DAOM 181602]
XP_001901591.1	205	Bm1_50615	eukaryota>metazoa>nematoda	Brugia	malayi	WH1 domain containing protein [Brugia malayi]
XP_017171007.1	203	homer-1,PSD-Zip45,SYN47,Ves-1,vesl-1	eukaryota>metazoa>chordata>vertebrata	Mus	musculus	PREDICTED: homer protein homolog 1 isoform X1 [Mus musculus]
XP_018292777.1	203	PHYBLDRAFT_124118	eukaryota>fungi	Phycomyces	blakesleeanus NRRL 1555(-)	hypothetical protein PHYBLDRAFT_124118, partial [Phycomyces blakesleeanus NRRL 1555(-)]
XP_012567082.1	199	LOC100215288	eukaryota>metazoa>cnidaria	Hydra	vulgaris	PREDICTED: homer protein homolog 2-like [Hydra vulgaris]
XP_018089156.1	196	LOC108700459	eukaryota>metazoa>chordata>vertebrata	Xenopus	laevis	PREDICTED: sprouty-related, EVH1 domain-containing protein 1-like [Xenopus laevis]
KNE72913.1	196	AMAG_20633	eukaryota>fungi	Allomyces	macrogynus ATCC 38327	hypothetical protein AMAG_20633 [Allomyces macrogynus ATCC 38327]
ELR25045.1	194	ACA1_287240	eukaryota>amoebozoa>acanthamoebidae	Acanthamoeba	castellanii str. Neff	WH1 domain containing protein [Acanthamoeba castellanii str. Neff]
XP_005157353.1	193	homer1,zgc:100854,zgc:92844	eukaryota>metazoa>chordata>vertebrata>actinopterygii	Danio	rerio	homer protein homolog 1 isoform X2 [Danio rerio]
NP_001161217.1	193	2900021I12Rik,3110031I02Rik,N-WASP	eukaryota>metazoa>chordata>vertebrata	Mus	musculus	neural Wiskott-Aldrich syndrome protein isoform 2 [Mus musculus]
CAG12079.1	191	GSTEN:00034222:G:001	eukaryota>metazoa>chordata>vertebrata>actinopterygii	Tetraodon	nigroviridis	unnamed protein product, partial [Tetraodon nigroviridis]
XP_001636870.1	190	NEMVEDRAFT_v1g202473	eukaryota>metazoa>cnidaria	Nematostella	vectensis	predicted protein [Nematostella vectensis]
XP_016865548.1	188	HOMER,HOMER1A,HOMER1B,HOMER1C,SYN47,Ves-1	eukaryota>metazoa>chordata>vertebrata	Homo	sapiens	homer protein homolog 1 isoform X1 [Homo sapiens]
NP_036112.1	186	homer-1,PSD-Zip45,SYN47,Ves-1,vesl-1	eukaryota>metazoa>chordata>vertebrata	Mus	musculus	homer protein homolog 1 isoform S [Mus musculus]
XP_005557308.1	184	HOMER1	eukaryota>metazoa>chordata>vertebrata	Macaca	fascicularis	PREDICTED: homer protein homolog 1 isoform X2 [Macaca fascicularis]
XP_013753353.1	184	AMSG_10645	eukaryota>apusozoa>apusomonadidae	Thecamonas	trahens ATCC 50062	hypothetical protein AMSG_10645 [Thecamonas trahens ATCC 50062]
XP_013389218.1	182	LOC106157956	eukaryota>metazoa	Lingula	anatina	protein enabled homolog [Lingula anatina]
XP_011606554.1	181	LOC105417035	eukaryota>metazoa>chordata>vertebrata>actinopterygii	Takifugu	rubripes	PREDICTED: vasodilator-stimulated phosphoprotein-like [Takifugu rubripes]
XP_019856530.1	179	LOC105314031	eukaryota>metazoa	Amphimedon	queenslandica	PREDICTED: neural Wiskott-Aldrich syndrome protein-like isoform X2 [Amphimedon queenslandica]
NP_001264007.1	177	HOMER,HOMER1A,HOMER1B,HOMER1C,SYN47,Ves-1	eukaryota>metazoa>chordata>vertebrata	Homo	sapiens	homer protein homolog 1 isoform 3 [Homo sapiens]
XP_012361039.1	177	HOMER1	eukaryota>metazoa>chordata>vertebrata	Nomascus	leucogenys	PREDICTED: homer protein homolog 1 isoform X3 [Nomascus leucogenys]
CBY34382.1	177	GSOID_T00024405001	eukaryota>metazoa>chordata	Oikopleura	dioica	unnamed protein product [Oikopleura dioica]
XP_021323364.1	176	si:ch211-255112.2	eukaryota>metazoa>chordata>vertebrata>actinopterygii	Danio	rerio	sprouty-related, EVH1 domain containing 3 isoform X4 [Danio rerio]
XP_009044835.1	173	LOTGIDRAFT_170749	eukaryota>metazoa>mollusca	Lottia	gigantea	hypothetical protein LOTGIDRAFT_170749 [Lottia gigantea]
EFX81577.1	161	DAPPUDRAFT_242162	eukaryota>metazoa>crustacea	Daphnia	pulex	hypothetical protein DAPPUDRAFT_242162 [Daphnia pulex]
ETO21130.1	156	RFI_16075	eukaryota>rhizaria	Reticulomyxa	filosa	hypothetical protein RFI_16075 [Reticulomyxa filosa]
XP_019040119.1	154	WICANDRAFT_29494	eukaryota>fungi>ascomycota	Wickerhamomyces	anomalus NRRL Y-366-8	hypothetical protein WICANDRAFT_29494, partial [Wickerhamomyces anomalus NRRL Y-366-8]
XP_013389219.1	152	LOC106157956	eukaryota>metazoa	Lingula	anatina	PREDICTED: protein enabled homolog isoform X2 [Lingula anatina]
EFX77865.1	151	DAPPUDRAFT_213242	eukaryota>metazoa>crustacea	Daphnia	pulex	hypothetical protein DAPPUDRAFT_213242, partial [Daphnia pulex]
XP_022249327.1	145	LOC111087333	eukaryota>metazoa	Limulus	polyphemus	homer protein homolog 2-like [Limulus polyphemus]
XP_004212513.1	145	LOC101238251	eukaryota>metazoa>cnidaria	Hydra	vulgaris	PREDICTED: wiskott-Aldrich syndrome protein-like [Hydra vulgaris]
XP_006822068.1	138	LOC100367435	eukaryota>metazoa>hemichordata	Saccoglossus	kowalevskii	PREDICTED: protein still life, isoform SIF type 1-like [Saccoglossus kowalevskii]
XP_009018259.1	134	HELRODRAFT_79834	eukaryota>metazoa>annelida	Helobdella	robusta	hypothetical protein HELRODRAFT_79834, partial [Helobdella robusta]
XP_009055943.1	134	LOTGIDRAFT_119577	eukaryota>metazoa>mollusca	Lottia	gigantea	hypothetical protein LOTGIDRAFT_119577 [Lottia gigantea]
XP_001633271.1	128	NEMVEDRAFT_v1g104827	eukaryota>metazoa>cnidaria	Nematostella	vectensis	predicted protein, partial [Nematostella vectensis]
XP_009166784.1	128	T265_03919	eukaryota>metazoa	Opisthorchis	viverrini	hypothetical protein T265_03919 [Opisthorchis viverrini]
XP_018668300.1	127	LOC100179781	eukaryota>metazoa>chordata	Ciona	intestinalis	PREDICTED: neural Wiskott-Aldrich syndrome protein-like [Ciona intestinalis]
XP_002585829.1	126	BRAFLDRAFT_256955	eukaryota>metazoa>chordata	Branchiostoma	floridae	hypothetical protein BRAFLDRAFT_256955 [Branchiostoma floridae]
XP_009016952.1	125	HELRODRAFT_78249	eukaryota>metazoa>annelida	Helobdella	robusta	hypothetical protein HELRODRAFT_78249, partial [Helobdella robusta]
XP_001743073.1	119	MONBRDRAFT_14345	eukaryota>choanoflagellida	Monosiga	brevicollis MX1	hypothetical protein, partial [Monosiga brevicollis MX1]
XP_009021879.1	118	HELRODRAFT_127531	eukaryota>metazoa>annelida	Helobdella	robusta	hypothetical protein HELRODRAFT_127531, partial [Helobdella robusta]
XP_009052802.1	118	LOTGIDRAFT_214569	eukaryota>metazoa>mollusca	Lottia	gigantea	hypothetical protein LOTGIDRAFT_214569 [Lottia gigantea]
XP_681984.1	118	AN8715.2	eukaryota>fungi>ascomycota	Aspergillus	nidulans FGSC A4	hypothetical protein AN8715.2 [Aspergillus nidulans FGSC A4]
XP_001619064.1	117	NEMVEDRAFT_v1g224549	eukaryota>metazoa>cnidaria	Nematostella	vectensis	hypothetical protein NEMVEDRAFT_v1g224549 [Nematostella vectensis]
OAD07496.1	106	MUCCIDRAFT_123792	eukaryota>fungi	Mucor	circinelloides f. lusitanicus CBS 277.49	hypothetical protein MUCCIDRAFT_123792, partial [Mucor circinelloides f. lusitanicus CBS 277.49]
OAC98115.1	104	MUCCIDRAFT_123755	eukaryota>fungi	Mucor	circinelloides f. lusitanicus CBS 277.49	hypothetical protein MUCCIDRAFT_123755, partial [Mucor circinelloides f. lusitanicus CBS 277.49]
XP_009022206.1	101	HELRODRAFT_127019	eukaryota>metazoa>annelida	Helobdella	robusta	hypothetical protein HELRODRAFT_127019, partial [Helobdella robusta]
XP_002111045.1	98	TRIADDRAFT_22944	eukaryota>metazoa>placozoa	Trichoplax	adhaerens	hypothetical protein TRIADDRAFT_22944, partial [Trichoplax adhaerens]

N. ERdj5

OLP80009.1	1597	DNAJB11	eukaryota>alveolata	Symbiodinium	microadriaticum	DnaJ-like subfamily B member 11, partial [Symbiodinium microadriaticum]
CEO95860.1	918	PBRA_004573	eukaryota>rhizaria>cercozoa>plasmodiophorida	Plasmodiophora	brassicae	hypothetical protein PBRA_004573 [Plasmodiophora brassicae]
KXJ25509.1	875	dnajc10	eukaryota>metazoa>cnidaria	Exaiptasia	pallida	DnaJ-like subfamily C member 10 [Exaiptasia pallida]
XP_009026746.1	872	HELRODRAFT_193633	eukaryota>metazoa>annelida	Helobdella	robusta	hypothetical protein HELRODRAFT_193633 [Helobdella robusta]
XP_001635821.1	844	NEMVEDRAFT_v1g163820	eukaryota>metazoa>cnidaria	Nematostella	vectensis	predicted protein [Nematostella vectensis]
XP_001894589.1	839	Bm1_15660	eukaryota>metazoa>nematoda	Brugia	malayi	DnaJ domain containing protein [Brugia malayi]
CAG01766.1	831	GSTEN:00020649:G:001	eukaryota>metazoa>chordata>vertebrata>actinopterygii	Tetraodon	nigroviridis	unnamed protein product, partial [Tetraodon nigroviridis]
XP_001862421.1	831	CpipJ_CPIJ012337	eukaryota>metazoa>hexapoda	Culex	quinquefasciatus	conserved hypothetical protein [Culex quinquefasciatus]
XP_017325246.1	830	dnajc10	eukaryota>metazoa>chordata>vertebrata>actinopterygii	Ictalurus	punctatus	PREDICTED: dnaJ homolog subfamily C member 10 isoform X1 [Ictalurus punctatus]
ERG81725.1	830	ASU_09741	eukaryota>metazoa>nematoda	Ascaris	suum	hypothetical protein ASU_09741 [Ascaris suum]
Caps1000007577	826	Caps1000007577	eukaryota>metazoa>annelida	Capitella	spI	>Caps1000007577 Caps1000007577
XP_395584.4	815	LOC412118	eukaryota>metazoa>hexapoda	Apis	mellifera	PREDICTED: dnaJ homolog subfamily C member 16 [Apis mellifera]
NP_001040753.1	813	dnj-8	eukaryota>metazoa>nematoda	Caenorhabditis	elegans	DNaJ domain (prokaryotic heat shock protein) [Caenorhabditis elegans]
XP_011146374.1	812	LOC105187332	eukaryota>metazoa>hexapoda	Harpegnathos	saltator	dnaJ homolog subfamily C member 16 [Harpegnathos saltator]
XP_002122728.1	811	LOC100179175	eukaryota>metazoa>chordata	Ciona	intestinalis	PREDICTED: dnaJ homolog subfamily C member 10 [Ciona intestinalis]
XP_019700629.1	810	LOC105191156	eukaryota>metazoa>hexapoda	Harpegnathos	saltator	dnaJ homolog subfamily C member 10 isoform X1 [Harpegnathos saltator]
CEL95543.1	810	Vbra_11973	eukaryota>alveolata	Vitrella	brassicaformis CCMP3155	unnamed protein product [Vitrella brassicaformis CCMP3155]
XP_003372029.1	809	Tsp_05680	eukaryota>metazoa>nematoda	Trichinella	spiralis	DnaJ protein subfamily C member 10 [Trichinella spiralis]
CBY20690.1	807	GSOID_T00000693001	eukaryota>metazoa>chordata	Oikopleura	dioica	unnamed protein product [Oikopleura dioica]
EFX65186.1	807	DAPPUDRAFT_333446	eukaryota>metazoa>crustacea	Daphnia	pulex	hypothetical protein DAPPUDRAFT_333446 [Daphnia pulex]
XP_019700630.1	807	LOC105191156	eukaryota>metazoa>hexapoda	Harpegnathos	saltator	dnaJ homolog subfamily C member 10 isoform X3 [Harpegnathos saltator]
XP_782974.2	807	LOC577666	eukaryota>metazoa>echinodermata	Strongylocentrotus	purpuratus	PREDICTED: dnaJ homolog subfamily C member 10 [Strongylocentrotus purpuratus]
XP_003973687.1	807	LOC101061594	eukaryota>metazoa>chordata>vertebrata>actinopterygii	Takifugu	rubripes	PREDICTED: dnaJ homolog subfamily C member 16-like [Takifugu rubripes]
XP_011152658.1	806	LOC105191156	eukaryota>metazoa>hexapoda	Harpegnathos	saltator	dnaJ homolog subfamily C member 10 isoform X4 [Harpegnathos saltator]
XP_015287942.1	802	DNAJC10	eukaryota>metazoa>chordata>vertebrata	Macaca	fascicularis	PREDICTED: dnaJ homolog subfamily C member 10 isoform X1 [Macaca fascicularis]
XP_015287943.1	802	DNAJC10	eukaryota>metazoa>chordata>vertebrata	Macaca	fascicularis	PREDICTED: dnaJ homolog subfamily C member 10 isoform X1 [Macaca fascicularis]
XP_015287944.1	802	DNAJC10	eukaryota>metazoa>chordata>vertebrata	Macaca	fascicularis	PREDICTED: dnaJ homolog subfamily C member 10 isoform X1 [Macaca fascicularis]
XP_017325247.1	801	dnajc10	eukaryota>metazoa>chordata>vertebrata>actinopterygii	Ictalurus	punctatus	PREDICTED: dnaJ homolog subfamily C member 10 isoform X2 [Ictalurus punctatus]
XP_017325248.1	801	dnajc10	eukaryota>metazoa>chordata>vertebrata>actinopterygii	Ictalurus	punctatus	PREDICTED: dnaJ homolog subfamily C member 10 isoform X2 [Ictalurus punctatus]

[illegible]

Uram1000008023	529	Uram1000008023	eukaryota>fungi>mucoromycotina	Umbelopsis	ramanniana	>Uram1000008023 Uram1000008023
XP_008620887.1	528	SDRG_16454	eukaryota>stramenopiles	Saprolegnia	diclina VS20	hypothetical protein SDRG_16454 [Saprolegnia diclina VS20]
XP_012205107.1	528	SPRG_10591	eukaryota>stramenopiles	Saprolegnia	parasitica CBS 223.65	hypothetical protein SPRG_10591 [Saprolegnia parasitica CBS 223.65]
Pram1000000294	528	Pram1000000294	eukaryota>stramenopiles	Phytophthora	ramorum	>Pram1000000294 Pram1000000294
CAG09040.1	527	GSTEN:00030205:G:001	eukaryota>metazoa>chordata>vertebrata>actinopterygii	Tetraodon	nigroviridis	unnamed protein product, partial [Tetraodon nigroviridis]
XP_002897239.1	527	PITG_17035	eukaryota>stramenopiles	Phytophthora	infestans T30-4	conserved hypothetical protein [Phytophthora infestans T30-4]
XP_008914002.1	527	PPTG_17882	eukaryota>stramenopiles	Phytophthora	parasitica INRA-310	hypothetical protein PPTG_17882 [Phytophthora parasitica INRA-310]
XP_003055188.1	526	MICPUCDRAFT_31096	eukaryota>viridiplantae>chlorophyta	Micromonas	pusilla CCMP1545	predicted protein [Micromonas pusilla CCMP1545]
XP_002785185.1	525	Pmar_PMAR008147	eukaryota>alveolata	Perkinsus	marinus ATCC 50983	EH-domain-containing protein, putative [Perkinsus marinus ATCC 50983]
XP_013758078.1	525	AMSG_05001	eukaryota>apusozoa>apusomonadidae	Thecamonas	trahens ATCC 50062	EH domain-containing protein 3 [Thecamonas trahens ATCC 50062]
XP_018299378.1	525	PHYBLDRAFT_120878	eukaryota>fungi	Phycomyces	blakesleeanus NRRL 1555(-)	hypothetical protein PHYBLDRAFT_120878 [Phycomyces blakesleeanus NRRL 1555(-)]
Bcir1000003721	524	Bcir1000003721	eukaryota>fungi>mucoromycotina	Backusella	circina	>Bcir1000003721 Bcir1000003721
Pram1000015057	524	Pram1000015057	eukaryota>stramenopiles	Phytophthora	ramorum	>Pram1000015057 Pram1000015057
XP_003369002.1	523	Tsp_04432	eukaryota>metazoa>nematoda	Trichinella	spiralis	putative EH domain-containing protein 3, partial [Trichinella spiralis]
XP_001784382.1	522	PHYPADRAFT_153849	eukaryota>viridiplantae	Physcomitrella	patens	predicted protein [Physcomitrella patens]
XP_012758189.1	520	SAMD00019534_021440	eukaryota>amoebozoa>mycetozoa>dictyosteliida	Acytostelium	subglobosum LB1	hypothetical protein SAMD00019534_021440, partial [Acytostelium subglobosum LB1]
OQV22217.1	519	BV898_03719	eukaryota>metazoa	Hypsibius	dujardini	hypothetical protein BV898_03719 [Hypsibius dujardini]
XP_008860576.1	517	ENU1_210180	eukaryota>amoebozoa>entamoebidae	Entamoeba	nuttalli P19	ENTH domain containing protein, putative [Entamoeba nuttalli P19]
XP_008861213.1	517	H310_00281	eukaryota>stramenopiles	Aphanomyces	invadans	hypothetical protein H310_00281 [Aphanomyces invadans]
XP_011778973.1	517	TbgDal_X18120	eukaryota>euglenozoa>kinetoplastida	Trypanosoma	brucei gambiense DAL972	sarcalumenin precursor, putative [Trypanosoma brucei gambiense DAL972]
XP_012203446.1	517	SPRG_08827	eukaryota>stramenopiles	Saprolegnia	parasitica CBS 223.65	hypothetical protein SPRG_08827 [Saprolegnia parasitica CBS 223.65]
XP_013239309.1	516	DI09_12p50	eukaryota>fungi>microsporidia	Mitosporidium	daphniae	hypothetical protein DI09_12p50 [Mitosporidium daphniae]
XP_015664083.1	516	ABB37_00043	eukaryota>euglenozoa>kinetoplastida	Leptomonas	pyrrhocoris	putative mitochondrial hypothetical protein [Leptomonas pyrrhocoris]
XP_015664084.1	516	ABB37_00043	eukaryota>euglenozoa>kinetoplastida	Leptomonas	pyrrhocoris	putative mitochondrial hypothetical protein [Leptomonas pyrrhocoris]
Smar1000012255	516	Smar1000012255	eukaryota>metazoa>arthropoda>myriapoda	Strigamia	maritima	>Smar1000012255 Smar1000012255
CAG01795.1	515	GSTEN:00020682:G:001	eukaryota>metazoa>chordata>vertebrata>actinopterygii	Tetraodon	nigroviridis	unnamed protein product, partial [Tetraodon nigroviridis]
XP_009040221.1	514	AURANDRAFT_31432	eukaryota>stramenopiles	Aureococcus	anophagefferens	hypothetical protein AURANDRAFT_31432 [Aureococcus anophagefferens]
KFH65080.1	511	MVEG_08561	eukaryota>fungi	Mortierella	verticillata NRRL 6337	hypothetical protein MVEG_08561 [Mortierella verticillata NRRL 6337]
XP_008870165.1	509	H310_06767	eukaryota>stramenopiles	Aphanomyces	invadans	hypothetical protein H310_06767 [Aphanomyces invadans]
XP_009822927.1	509	H257_01428	eukaryota>stramenopiles	Aphanomyces	astaci	hypothetical protein H257_01428 [Aphanomyces astaci]
GAT91521.1	508	CL6EHI_152680	eukaryota>amoebozoa>entamoebidae	Entamoeba	histolytica	EH-domain containing protein putative [Entamoeba histolytica]
XP_006683041.1	508	BATDEDRAFT_14976	eukaryota>fungi>chytridiomycota	Batrachochytrium	dendrobatidis JAM81	hypothetical protein BATDEDRAFT_14976 [Batrachochytrium dendrobatidis JAM81]
XP_018650317.1	508	Smp_065180	eukaryota>metazoa	Schistosoma	mansoni	eh domain containing/past-1-related [Schistosoma mansoni]
GAT94904.1	507	CL6EHI_105270	eukaryota>amoebozoa>entamoebidae	Entamoeba	histolytica	receptor mediated endocytosis protein putative [Entamoeba histolytica]
GAT95987.1	507	CL6EHI_052870	eukaryota>amoebozoa>entamoebidae	Entamoeba	histolytica	enth domain protein putative [Entamoeba histolytica]
XP_001734264.1	507	EDI_210720	eukaryota>amoebozoa>entamoebidae	Entamoeba	dispar SAW760	EH domain-containing protein [Entamoeba dispar SAW760]
XP_001735333.1	507	EDI_315860	eukaryota>amoebozoa>entamoebidae	Entamoeba	dispar SAW760	EH domain-containing protein [Entamoeba dispar SAW760]
XP_004183208.1	507	EIN_198150	eukaryota>amoebozoa>entamoebidae	Entamoeba	invadens IP1	EH domain containing protein, putative [Entamoeba invadens IP1]
XP_004261439.1	507	EIN_499380	eukaryota>amoebozoa>entamoebidae	Entamoeba	invadens IP1	EH domain containing protein, putative [Entamoeba invadens IP1]
XP_004261078.1	505	EIN_130360	eukaryota>amoebozoa>entamoebidae	Entamoeba	invadens IP1	EH domain containing protein, putative [Entamoeba invadens IP1]
XP_005841179.1	503	GUITHDRAFT_160834	eukaryota>cryptophyta	Guillardia	theta CCMP2712	hypothetical protein GUITHDRAFT_160834 [Guillardia theta CCMP2712]
ELR12122.1	502	ACA1_139840	eukaryota>amoebozoa>acanthamoebidae	Acanthamoeba	castellanii str. Neff	receptor mediated endocytosis family member (rme1), putative [Acanthamoeba castellanii str. Neff]
XP_009830450.1	501	H257_06788	eukaryota>stramenopiles	Aphanomyces	astaci	hypothetical protein H257_06788 [Aphanomyces astaci]
XP_008861214.1	500	H310_00281	eukaryota>stramenopiles	Aphanomyces	invadans	hypothetical protein, variant [Aphanomyces invadans]
XP_008615202.1	498	SDRG_10963	eukaryota>stramenopiles	Saprolegnia	diclina VS20	hypothetical protein SDRG_10963 [Saprolegnia diclina VS20]
XP_012196138.1	498	SPRG_02197	eukaryota>stramenopiles	Saprolegnia	parasitica CBS 223.65	hypothetical protein SPRG_02197 [Saprolegnia parasitica CBS 223.65]
XP_003641397.2	494	EHD4	eukaryota>metazoa>chordata>vertebrata	Gallus	gallus	EH domain-containing protein 4 isoform X2 [Gallus gallus]
XP_001564962.1	493	LBRM_22_0430	eukaryota>euglenozoa>kinetoplastida	Leishmania	braziliensis MHOM/BR/75/M2904	conserved hypothetical protein [Leishmania braziliensis MHOM/BR/75/M2904]
XP_010699047.1	493	LPMP_220460	eukaryota>euglenozoa>kinetoplastida	Leishmania	panamensis	hypothetical protein LPMP_220460 [Leishmania panamensis]
XP_017317733.1	490	LOC108261333	eukaryota>metazoa>chordata>vertebrata>actinopterygii	Ictalurus	punctatus	PREDICTED: sarcalumenin-like [Ictalurus punctatus]
XP_014668558.1	489	LOC106809844	eukaryota>metazoa	Priapulus	caudatus	PREDICTED: LOW QUALITY PROTEIN: EH domain-containing protein 3-like [Priapulus caudatus]
EPY29863.1	487	AGDE_09748	eukaryota>euglenozoa>kinetoplastida	Angomonas	deanei	hypothetical protein AGDE_09748 [Angomonas deanei]
EPY41112.1	487	AGDE_02813	eukaryota>euglenozoa>kinetoplastida	Angomonas	deanei	hypothetical protein AGDE_02813 [Angomonas deanei]
XP_018094740.1	484	LOC446267	eukaryota>metazoa>chordata>vertebrata	Xenopus	laevis	PREDICTED: sarcalumenin isoform X1 [Xenopus laevis]
CBJ30463.1	483	RME1L1	eukaryota>stramenopiles	Ectocarpus	siliculosus	RME1L1, RME1-like GTPase/ATPase without a C-terminal EH domain [Ectocarpus siliculosus]
CAG00163.1	482	GSTEN:00018510:G:001	eukaryota>metazoa>chordata>vertebrata>actinopterygii	Tetraodon	nigroviridis	unnamed protein product, partial [Tetraodon nigroviridis]
NP_001070999.1	482	im:6899248,wu:fb94b10,zgc:153844	eukaryota>metazoa>chordata>vertebrata>actinopterygii	Danio	rerio	sarcalumenin precursor [Danio rerio]
XP_003964691.1	482	srl	eukaryota>metazoa>chordata>vertebrata>actinopterygii	Takifugu	rubripes	PREDICTED: sarcalumenin isoform X1 [Takifugu rubripes]
XP_013413281.1	482	LOC106175703	eukaryota>metazoa	Lingula	anatina	sarcalumenin [Lingula anatina]
XP_006823804.1	477	LOC102801464	eukaryota>metazoa>hemichordata	Saccoglossus	kowalevskii	PREDICTED: sarcalumenin-like [Saccoglossus kowalevskii]
XP_002946165.1	476	VOLCADRAFT_55400	eukaryota>viridiplantae>chlorophyta	Volvox	carteri f. nagariensis	hypothetical protein VOLCADRAFT_55400 [Volvox carteri f. nagariensis]
NP_001092284.1	473	SAR	eukaryota>metazoa>chordata>vertebrata	Homo	sapiens	sarcalumenin isoform 1 precursor [Homo sapiens]
XP_002772566.1	473	Pmar_PMAR021539	eukaryota>alveolata	Perkinsus	marinus ATCC 50983	EH-domain-containing protein, putative [Perkinsus marinus ATCC 50983]
XP_017172333.1	473	9830004M20Rik,AW108387,sar	eukaryota>metazoa>chordata>vertebrata	Mus	musculus	PREDICTED: sarcalumenin isoform X3 [Mus musculus]
XP_017172334.1	472	9830004M20Rik,AW108387,sar	eukaryota>metazoa>chordata>vertebrata	Mus	musculus	PREDICTED: sarcalumenin isoform X4 [Mus musculus]
XP_018092022.1	472	srl.L	eukaryota>metazoa>chordata>vertebrata	Xenopus	laevis	PREDICTED: sarcalumenin isoform X3 [Xenopus laevis]
XP_018094741.1	472	LOC446267	eukaryota>metazoa>chordata>vertebrata	Xenopus	laevis	PREDICTED: sarcalumenin isoform X2 [Xenopus laevis]
NP_990660.1	471	SRL	eukaryota>metazoa>chordata>vertebrata	Gallus	gallus	sarcalumenin precursor [Gallus gallus]
OEU18895.1	471	FRACYDRAFT_183357	eukaryota>stramenopiles	Fragilariopsis	cylindrus CCMP1102	hypothetical protein FRACYDRAFT_183357 [Fragilariopsis cylindrus CCMP1102]
XP_003964690.1	470	srl	eukaryota>metazoa>chordata>vertebrata>actinopterygii	Takifugu	rubripes	PREDICTED: sarcalumenin isoform X2 [Takifugu rubripes]
XP_009298079.1	470	im:6899248,wu:fb94b10,zgc:153844	eukaryota>metazoa>chordata>vertebrata>actinopterygii	Danio	rerio	sarcalumenin isoform X1 [Danio rerio]
XP_016452176.1	467	LOC107776767	eukaryota>viridiplantae	Nicotiana	tabacum	PREDICTED: EH domain-containing protein 1-like isoform X2 [Nicotiana tabacum]
XP_017208913.1	466	wu:fb80c09	eukaryota>metazoa>chordata>vertebrata>actinopterygii	Danio	rerio	PREDICTED: sarcalumenin, partial [Danio rerio]
XP_009857606.1	460	LOC104265388	eukaryota>metazoa>chordata	Ciona	intestinalis	PREDICTED: sarcalumenin-like [Ciona intestinalis]
XP_014526523.1	458	JH06_3112	eukaryota>stramenopiles	Blastocystis	sp. subtype 4	hypothetical protein JH06_3112 [Blastocystis sp. subtype 4]
XP_016877589.1	453	PAST4	eukaryota>metazoa>chordata>vertebrata	Homo	sapiens	PREDICTED: EH domain-containing protein 4 isoform X1 [Homo sapiens]
XP_017174841.1	453	2210022F10Rik,AI197390,AI846352,AV006278,Past2	eukaryota>metazoa>chordata>vertebrata	Mus	musculus	PREDICTED: EH domain-containing protein 4 isoform X1 [Mus musculus]
XP_012895831.1	449	GSBLH_T00001899001	eukaryota>stramenopiles	Blastocystis	hominis	uncharacterized protein [Blastocystis hominis]
XP_009014569.1	446	HELRODRAFT_76627	eukaryota>metazoa>annelida	Helobdella	robusta	hypothetical protein HELRODRAFT_76627, partial [Helobdella robusta]
XP_001465567.1	444	LINJ_22_0360	eukaryota>euglenozoa>kinetoplastida	Leishmania	infantum JPCM5	conserved hypothetical protein [Leishmania infantum JPCM5]
XP_001683200.1	444	LMJF_22_0480	eukaryota>euglenozoa>kinetoplastida	Leishmania	major strain Friedlin	conserved hypothetical protein [Leishmania major strain Friedlin]
XP_003860786.1	444	LDBPK_220360	eukaryota>euglenozoa>kinetoplastida	Leishmania	donovani	hypothetical protein, conserved [Leishmania donovani]
XP_003875503.1	444	LMXM_22_0480	eukaryota>euglenozoa>kinetoplastida	Leishmania	mexicana MHOM/GT/2001/U1103	conserved hypothetical protein [Leishmania mexicana MHOM/GT/2001/U1103]
CAG06078.1	441	GSTEN:00026313:G:001	eukaryota>metazoa>chordata>vertebrata>actinopterygii	Tetraodon	nigroviridis	unnamed protein product, partial [Tetraodon nigroviridis]
OLP82663.1	439	Ehd1	eukaryota>alveolata	Symbiodinium	microadriaticum	EH domain-containing protein 1 [Symbiodinium microadriaticum]
XP_002765121.1	439	Pmar_PMAR003862	eukaryota>alveolata	Perkinsus	marinus ATCC 50983	EH-domain-containing protein, putative [Perkinsus marinus ATCC 50983]
XP_009039421.1	437	AURANDRAFT_30262	eukaryota>stramenopiles	Aureococcus	anophagefferens	hypothetical protein AURANDRAFT_30262 [Aureococcus anophagefferens]
NP_001310596.1	431	SAR	eukaryota>metazoa>chordata>vertebrata	Homo	sapiens	sarcalumenin isoform 2 [Homo sapiens]
NP_001310597.1	431	SAR	eukaryota>metazoa>chordata>vertebrata	Homo	sapiens	sarcalumenin isoform 2 [Homo sapiens]
XP_016879017.1	431	SRL	eukaryota>metazoa>chordata>vertebrata	Homo	sapiens	sarcalumenin isoform X2 [Homo sapiens]
XP_016879018.1	431	SRL	eukaryota>metazoa>chordata>vertebrata	Homo	sapiens	sarcalumenin isoform X2 [Homo sapiens]
XP_017172335.1	431	9830004M20Rik,AW108387,sar	eukaryota>metazoa>chordata>vertebrata	Mus	musculus	PREDICTED: sarcalumenin isoform X5 [Mus musculus]
XP_017172336.1	431	9830004M20Rik,AW108387,sar	eukaryota>metazoa>chordata>vertebrata	Mus	musculus	PREDICTED: sarcalumenin isoform X5 [Mus musculus]
XP_002499973.1	427	MICPUN_107789	eukaryota>viridiplantae>chlorophyta	Micromonas	commoda	predicted protein [Micromonas commoda]
XP_016486423.1	427	LOC107806716	eukaryota>viridiplantae	Nicotiana	tabacum	PREDICTED: EH domain-containing protein 1-like isoform X1 [Nicotiana tabacum]
EWM25448.1	420	RME1L1	eukaryota>stramenopiles	Nannochloropsis	gaditana	receptor mediated endocytosis family member (rme-1) [Nannochloropsis gaditana]
Pisp1000006440	420	Pisp1000006440	eukaryota>fungi>neocallimastigomycota	Piromyces	sp	>Pisp1000006440 Pisp1000006440
XP_005835236.1	414	GUITHDRAFT_157516	eukaryota>cryptophyta	Guillardia	theta CCMP2712	hypothetical protein GUITHDRAFT_157516 [Guillardia theta CCMP2712]
XP_012352130.1	413	EHD2	eukaryota>metazoa>chordata>vertebrata	Nomascus	leucogenys	PREDICTED: EH domain-containing protein 2 [Nomascus leucogenys]
EFX74164.1	412	DAPPUDRAFT_307451	eukaryota>metazoa>crustacea	Daphnia	pulex	hypothetical protein DAPPUDRAFT_307451 [Daphnia pulex]
ETO05470.1	412	RFI_31925	eukaryota>rhizaria	Reticulomyxa	filosa	hypothetical protein RFI_31925 [Reticulomyxa filosa]
XP_009040272.1	405	AURANDRAFT_1674	eukaryota>stramenopiles	Aureococcus	anophagefferens	hypothetical protein AURANDRAFT_1674, partial [Aureococcus anophagefferens]
Smed1000013153	398	Smed1000013153	eukaryota>metazoa>platyhelminthe	Schmidtea	mediterranea	>Smed1000013153 Smed1000013153
XP_016484342.1	397	LOC107804905	eukaryota>viridiplantae	Nicotiana	tabacum	PREDICTED: EH domain-containing protein 1-like, partial [Nicotiana tabacum]
XP_016487400.1	397	LOC107807506	eukaryota>viridiplantae	Nicotiana	tabacum	PREDICTED: EH domain-containing protein 1-like, partial [Nicotiana tabacum]
OEU22607.1	391	FRACYDRAFT_166535	eukaryota>stramenopiles	Fragilariopsis	cylindrus CCMP1102	Dynamin N-domain-containing protein [Fragilariopsis cylindrus CCMP1102]
XP_002177526.1	390	PHATRDRAFT_8770	eukaryota>stramenopiles	Phaeodactylum	tricornutum CCAP 1055/1	predicted protein [Phaeodactylum tricornutum CCAP 1055/1]
XP_002291221.1	385	THAPSDRAFT_13909	eukaryota>stramenopiles	Thalassiosira	pseudonana CCMP1335	eh domain-containing protein, partial [Thalassiosira pseudonana CCMP1335]
XP_002966377.1	385	SELMODRAFT_85478	eukaryota>viridiplantae	Selaginella	moellendorffii	hypothetical protein SELMODRAFT_85478, partial [Selaginella moellendorffii]
XP_002978188.1	385	SELMODRAFT_108442	eukaryota>viridiplantae	Selaginella	moellendorffii	hypothetical protein SELMODRAFT_108442, partial [Selaginella moellendorffii]
XP_009036435.1	383	AURANDRAFT_1666	eukaryota>stramenopiles	Aureococcus	anophagefferens	hypothetical protein AURANDRAFT_1666, partial [Aureococcus anophagefferens]
XP_002289284.1	382	THAPSDRAFT_33868	eukaryota>stramenopiles	Thalassiosira	pseudonana CCMP1335	probable eh domain-containing protein, partial [Thalassiosira pseudonana CCMP1335]
XP_013898083.1	381	MNEG_8899	eukaryota>viridiplantae>chlorophyta	Monoraphidium	neglectum	EH domain-containing protein 4 [Monoraphidium neglectum]
ETO26663.1	374	RFI_10471	eukaryota>rhizaria	Reticulomyxa	filosa	EH-domain containing 4, partial [Reticulomyxa filosa]
XP_009293166.1	374	si:ch211-147c1.2	eukaryota>metazoa>chordata>vertebrata>actinopterygii	Danio	rerio	PREDICTED: EH domain-containing protein 3 isoform X1 [Danio rerio]
Bnat1000005148	374	Bnat1000005148	eukaryota>rhizaria	Bigelowiella	natans	>Bnat1000005148 Bnat1000005148
XP_015129283.1	372	LOC107049991	eukaryota>metazoa>chordata>vertebrata	Gallus	gallus	PREDICTED: LOW QUALITY PROTEIN: EH domain-containing protein 2-like, partial [Gallus gallus]
XP_001896504.1	361	Bm1_25225	eukaryota>metazoa>nematoda	Brugia	malayi	EH-domain containing protein 3 [Brugia malayi]
ERG82150.1	353	ASU_09059	eukaryota>metazoa>nematoda	Ascaris	suum	eh domain-containing protein 1 [Ascaris suum]
ETO26762.1	351	RFI_10373	eukaryota>rhizaria	Reticulomyxa	filosa	hypothetical protein RFI_10373, partial [Reticulomyxa filosa]
XP_002779611.1	331	Pmar_PMAR004572	eukaryota>alveolata	Perkinsus	marinus ATCC 50983	conserved hypothetical protein, partial [Perkinsus marinus ATCC 50983]
Bnat1000007215	329	Bnat1000007215	eukaryota>rhizaria	Bigelowiella	natans	>Bnat1000007215 Bnat1000007215
XP_002411757.1	324	IscW_ISCW021581	eukaryota>metazoa	Ixodes	scapularis	endocytosis/signaling protein EHD1, putative [Ixodes scapularis]
XP_011531108.1	322	PAST3	eukaryota>metazoa>chordata>vertebrata	Homo	sapiens	EH domain-containing protein 3 isoform X1 [Homo sapiens]
ETO08814.1	320	RFI_28574	eukaryota>rhizaria	Reticulomyxa	filosa	EH domain-containing protein 4, partial [Reticulomyxa filosa]
XP_002769901.1	320	Pmar_PMAR018864	eukaryota>alveolata	Perkinsus	marinus ATCC 50983	EH-domain-containing protein, putative, partial [Perkinsus marinus ATCC 50983]
ETN97991.1	312	RFI_39531	eukaryota>rhizaria	Reticulomyxa	filosa	hypothetical protein RFI_39531, partial [Reticulomyxa filosa]
XP_005837811.1	310	GUITHDRAFT_157255	eukaryota>cryptophyta	Guillardia	theta CCMP2712	hypothetical protein GUITHDRAFT_157255 [Guillardia theta CCMP2712]
XP_012557383.1	301	LOC100211925	eukaryota>metazoa>cnidaria	Hydra	vulgaris	PREDICTED: EH domain-containing protein 3-like, partial [Hydra vulgaris]
Smed1000027697	296	Smed1000027697	eukaryota>metazoa>platyhelminthe	Schmidtea	mediterranea	>Smed1000027697 Smed1000027697
Bnat1000019652	282	Bnat1000019652	eukaryota>rhizaria	Bigelowiella	natans	>Bnat1000019652 Bnat1000019652
EPY43845.1	276	AGDE_00076	eukaryota>euglenozoa>kinetoplastida	Angomonas	deanei	hypothetical protein AGDE_00076 [Angomonas deanei]
OLP76986.1	271	Ehd1	eukaryota>alveolata	Symbiodinium	microadriaticum	EH domain-containing protein 1 [Symbiodinium microadriaticum]
XP_005833601.1	265	GUITHDRAFT_52547	eukaryota>cryptophyta	Guillardia	theta CCMP2712	hypothetical protein GUITHDRAFT_52547, partial [Guillardia theta CCMP2712]
XP_006585912.1	248	LOC100794877	eukaryota>viridiplantae	Glycine	max	PREDICTED: EH domain-containing protein 2-like isoform X1 [Glycine max]
XP_006585913.1	246	LOC100794877	eukaryota>viridiplantae	Glycine	max	PREDICTED: EH domain-containing protein 2-like isoform X2 [Glycine max]
CAF87806.1	245	GSTEN:00000798:G:001	eukaryota>metazoa>chordata>vertebrata>actinopterygii	Tetraodon	nigroviridis	unnamed protein product, partial [Tetraodon nigroviridis]
XP_013406319.1	242	LOC106170870	eukaryota>metazoa	Lingula	anatina	EH domain-containing protein 1-like [Lingula anatina]
XP_016486424.1	242	LOC107806716	eukaryota>viridiplantae	Nicotiana	tabacum	PREDICTED: EH domain-containing protein 1-like isoform X2 [Nicotiana tabacum]
XP_006585914.1	241	LOC100794877	eukaryota>viridiplantae	Glycine	max	PREDICTED: EH domain-containing protein 2-like isoform X3 [Glycine max]
XP_006585915.1	241	LOC100794877	eukaryota>viridiplantae	Glycine	max	PREDICTED: EH domain-containing protein 2-like isoform X3 [Glycine max]
XP_006585916.1	236	LOC100794877	eukaryota>viridiplantae	Glycine	max	PREDICTED: EH domain-containing protein 2-like isoform X4 [Glycine max]
XP_006585917.1	236	LOC100794877	eukaryota>viridiplantae	Glycine	max	PREDICTED: EH domain-containing protein 2-like isoform X4 [Glycine max]
XP_004995955.1	230	PTSG_03370	eukaryota>choanoflagellida	Salpingoeca	rosetta	hypothetical protein PTSG_03370 [Salpingoeca rosetta]
XP_002411758.1	210	IscW_ISCW021582	eukaryota>metazoa	Ixodes	scapularis	hypothetical protein IscW_ISCW021582, partial [Ixodes scapularis]
XP_002182541.1	204	PHATRDRAFT_14787	eukaryota>stramenopiles	Phaeodactylum	tricornutum CCAP 1055/1	predicted protein, partial [Phaeodactylum tricornutum CCAP 1055/1]
XP_016489207.1	204	LOC107809121	eukaryota>viridiplantae	Nicotiana	tabacum	PREDICTED: EH domain-containing protein 1-like [Nicotiana tabacum]
EWM24454.1	203	RME1	eukaryota>stramenopiles	Nannochloropsis	gaditana	dynamin GTPase family protein [Nannochloropsis gaditana]
ETO13471.1	196	RFI_23901	eukaryota>rhizaria	Reticulomyxa	filosa	EH domain-containing protein 4, partial [Reticulomyxa filosa]
XP_015129697.1	185	LOC107050461	eukaryota>metazoa>chordata>vertebrata	Gallus	gallus	PREDICTED: EH domain-containing protein 2-like, partial [Gallus gallus]
NP_001170407.1	177	GRMZM2G113613	eukaryota>viridiplantae	Zea	mays	uncharacterized protein LOC100384394 isoform 2 [Zea mays]
XP_008859898.1	162	ENU1_187130	eukaryota>amoebozoa>entamoebidae	Entamoeba	nuttalli P19	receptor mediated endocytosis protein, putative, partial [Entamoeba nuttalli P19]
XP_005792863.1	148	RME1A-2	eukaryota>haptophyceae	Emiliania	huxleyi CCMP1516	RME1A, RME-1/EHD family protein, partial [Emiliania huxleyi CCMP1516]
ENY62486.1	125	EHI7A_091100	eukaryota>amoebozoa>entamoebidae	Entamoeba	histolytica HM-1:IMSS-A	receptor mediated endocytosis protein, putative, partial [Entamoeba histolytica HM-1:IMSS-A]
EWM24449.1	116	RME1	eukaryota>stramenopiles	Nannochloropsis	gaditana	receptor mediated endocytosis protein, partial [Nannochloropsis gaditana]
ETO08813.1	106	RFI_28573	eukaryota>rhizaria	Reticulomyxa	filosa	receptor mediated endocytosis protein-like protein, partial [Reticulomyxa filosa]
EIE88190.1	88	RO3G_12901	eukaryota>fungi	Rhizopus	delemar RA 99-880	hypothetical protein RO3G_12901 [Rhizopus delemar RA 99-880]

P. IP3R

Bnat1000003017	4130	Bnat1000003017	eukaryota>rhizaria	Bigelowiella	natans	[illegible]
EJY82846.1	3898	OXYTRI_19537	eukaryota>alveolata>ciliophora	Oxytricha	trifallax	[illegible]
XP_001947344.2	3831	LOC100163622	eukaryota>metazoa>hexapoda	Acyrthosiphon	pisum	[illegible]
XP_008185766.1	3831	LOC100163622	eukaryota>metazoa>hexapoda	Acyrthosiphon	pisum	[illegible]
KPI87904.1	3823	ABL78_3014	eukaryota>euglenozoa>kinetoplastida	Leptomonas	seymouri	[illegible]
XP_001013602.2	3659	TTHERM_00885700	eukaryota>alveolata>ciliophora	Tetrahymena	thermophila SB210	[illegible]
CDW75428.1	3530	Contig1440.g1580	eukaryota>alveolata>ciliophora	Stylonychia	lemnae	[illegible]
XP_001013405.2	3525	TTHERM_00454060	eukaryota>alveolata>ciliophora	Tetrahymena	thermophila SB210	[illegible]
XP_976640.2	3489	TTHERM_00545830	eukaryota>alveolata>ciliophora	Tetrahymena	thermophila SB210	[illegible]
XP_001009504.2	3449	TTHERM_00370740	eukaryota>alveolata>ciliophora	Tetrahymena	thermophila SB210	[illegible]
XP_009493566.1	3443	H696_01395	eukaryota	Fonticula	alba	[illegible]
XP_001029961.2	3412	TTHERM_01205340	eukaryota>alveolata>ciliophora	Tetrahymena	thermophila SB210	[illegible]
XP_001030533.2	3401	TTHERM_01076970	eukaryota>alveolata>ciliophora	Tetrahymena	thermophila SB210	[illegible]
XP_017334767.1	3389	LOC108271603	eukaryota>metazoa>chordata>vertebrata>actinopterygii	Ictalurus	punctatus	[illegible]
CAG03532.1	3383	GSTEN:00022986:G:001	eukaryota>metazoa>chordata>vertebrata>actinopterygii	Tetraodon	nigroviridis	[illegible]
XP_001007910.2	3380	TTHERM_00538860	eukaryota>alveolata>ciliophora	Tetrahymena	thermophila SB210	[illegible]
XP_001007911.2	3344	TTHERM_00538870	eukaryota>alveolata>ciliophora	Tetrahymena	thermophila SB210	[illegible]
ELR11529.1	3344	ACA1_256780	eukaryota>amoebozoa>acanthamoebidae	Acanthamoeba	castellanii str. Neff	[illegible]
XP_001010363.2	3260	TTHERM_00354640	eukaryota>alveolata>ciliophora	Tetrahymena	thermophila SB210	[illegible]
XP_001024368.2	3243	TTHERM_00532470	eukaryota>alveolata>ciliophora	Tetrahymena	thermophila SB210	[illegible]
XP_004347577.1	3232	CAOG_04826	eukaryota>ichthyosporea	Capsaspora	owczarzaki ATCC 30864	[illegible]
XP_009839519.1	3199	H257_13636	eukaryota>stramenopiles	Aphanomyces	astaci	[illegible]
XP_013762131.1	3179	AMSG_00953	eukaryota>apusozoa>apusomonadidae	Thecamonas	trahens ATCC 50062	[illegible]
XP_629509.1	3177	iplA	eukaryota>amoebozoa>mycetozoa>dictyosteliida	Dictyostelium	discoideum AX4	[illegible]
XP_002959187.1	3167	VOLCADRAFT_121781	eukaryota>viridiplantae>chlorophyta	Volvox	carteri f. nagariensis	[illegible]
XP_001019190.2	3160	TTHERM_00997500	eukaryota>alveolata>ciliophora	Tetrahymena	thermophila SB210	[illegible]
XP_001701472.1	3140	RYR1	eukaryota>viridiplantae>chlorophyta	Chlamydomonas	reinhardtii	[illegible]

[illegible]

XP_005773691.1	382	EMIHUDRAFT_444540	eukaryota>haptophyceae	Emiliania	huxleyi CCMP1516	hypothetical protein EMIHUDRAFT_444540, partial [Emiliania huxleyi CCMP1516]
XP_005763709.1	381	EMIHUDRAFT_247965	eukaryota>haptophyceae	Emiliania	huxleyi CCMP1516	hypothetical protein EMIHUDRAFT_247965 [Emiliania huxleyi CCMP1516]
XP_006559876.1	377	LOC102655332	eukaryota>metazoa>hexapoda	Apis	mellifera	PREDICTED: protein orai-2-like [Apis mellifera]
XP_005775253.1	374	EMIHUDRAFT_95445	eukaryota>haptophyceae	Emiliania	huxleyi CCMP1516	hypothetical protein EMIHUDRAFT_95445 [Emiliania huxleyi CCMP1516]
XP_021205517.1	374	LOC101739927	eukaryota>metazoa>hexapoda	Bombyx	mori	calcium release-activated calcium channel protein 1 isoform X1 [Bombyx mori]
XP_012548235.1	371	LOC101739927	eukaryota>metazoa>hexapoda	Bombyx	mori	calcium release-activated calcium channel protein 1 isoform X2 [Bombyx mori]
XP_012548236.1	370	LOC101739927	eukaryota>metazoa>hexapoda	Bombyx	mori	calcium release-activated calcium channel protein 1 isoform X3 [Bombyx mori]
XP_012548237.1	366	LOC101739927	eukaryota>metazoa>hexapoda	Bombyx	mori	calcium release-activated calcium channel protein 1 isoform X4 [Bombyx mori]
XP_011144695.1	359	LOC105186300	eukaryota>metazoa>hexapoda	Harpegnathos	saltator	calcium release-activated calcium channel protein 1 [Harpegnathos saltator]
XP_001758939.1	353	PHYPADRAFT_161647	eukaryota>viridiplantae	Physcomitrella	patens	predicted protein [Physcomitrella patens]
NP_611273.1	351	BcDNA:LD03241,CG11430,CRACM1,Dmel\CG11430,dorai,dOrai,DOrai,dOrai1,l(2)k11505,Olf186-F,orai,Orai,Orai1,SPH206	eukaryota>metazoa>hexapoda	Drosophila	melanogaster	olf186-F, isoform B [Drosophila melanogaster]
NP_725727.1	351	BcDNA:LD03241,CG11430,CRACM1,Dmel\CG11430,dorai,dOrai,DOrai,dOrai1,l(2)k11505,Olf186-F,orai,Orai,Orai1,SPH206	eukaryota>metazoa>hexapoda	Drosophila	melanogaster	olf186-F, isoform A [Drosophila melanogaster]
NP_995881.2	351	BcDNA:LD03241,CG11430,CRACM1,Dmel\CG11430,dorai,dOrai,DOrai,dOrai1,l(2)k11505,Olf186-F,orai,Orai,Orai1,SPH206	eukaryota>metazoa>hexapoda	Drosophila	melanogaster	olf186-F, isoform F [Drosophila melanogaster]
XP_013227949.1	350	ETH_00012845	eukaryota>alveolata>apicomplexa	Eimeria	tenella	hypothetical protein, conserved [Eimeria tenella]
XP_002953952.1	340	ssa12	eukaryota>viridiplantae>chlorophyta	Volvox	carteri f. nagariensis	hypothetical protein VOLCADRAFT_127367 [Volvox carteri f. nagariensis]
XP_004993864.1	339	PTSG_05291	eukaryota>choanoflagellida	Salpingoeca	rosetta	hypothetical protein PTSG_05291 [Salpingoeca rosetta]
XP_021205518.1	339	LOC101739927	eukaryota>metazoa>hexapoda	Bombyx	mori	calcium release-activated calcium channel protein 1 isoform X5 [Bombyx mori]
OLP89363.1	333	AK812_SmicGene29195	eukaryota>alveolata	Symbiodinium	microadriaticum	hypothetical protein AK812_SmicGene29195 [Symbiodinium microadriaticum]
XP_002366727.1	329	TGME49_241180	eukaryota>alveolata>apicomplexa	Toxoplasma	gondii ME49	hypothetical protein TGME49_241180 [Toxoplasma gondii ME49]
XP_005777770.1	329	EMIHUDRAFT_471701	eukaryota>haptophyceae	Emiliania	huxleyi CCMP1516	hypothetical protein EMIHUDRAFT_471701 [Emiliania huxleyi CCMP1516]
XP_008887798.1	329	HHA_241180	eukaryota>alveolata>apicomplexa	Hammondia	hammondi	hypothetical protein HHA_241180 [Hammondia hammondi]
XP_008194887.1	323	LOC657890	eukaryota>metazoa>hexapoda	Tribolium	castaneum	PREDICTED: calcium release-activated calcium channel protein 1 isoform X1 [Tribolium castaneum]
XP_018670031.1	323	LOC100180182	eukaryota>metazoa>chordata	Ciona	intestinalis	PREDICTED: calcium release-activated calcium channel protein 1 isoform X1 [Ciona intestinalis]
XP_001850020.1	322	CpipJ_CPIJ007873	eukaryota>metazoa>hexapoda	Culex	quinquefasciatus	conserved hypothetical protein [Culex quinquefasciatus]
XP_013904039.1	320	MNEG_2934	eukaryota>viridiplantae>chlorophyta	Monoraphidium	neglectum	hypothetical protein MNEG_2934 [Monoraphidium neglectum]
XP_969411.1	320	LOC657890	eukaryota>metazoa>hexapoda	Tribolium	castaneum	PREDICTED: calcium release-activated calcium channel protein 1 isoform X2 [Tribolium castaneum]
XP_005782447.1	317	EMIHUDRAFT_462903	eukaryota>haptophyceae	Emiliania	huxleyi CCMP1516	hypothetical protein EMIHUDRAFT_462903 [Emiliania huxleyi CCMP1516]
XP_002970256.1	316	SELMODRAFT_441115	eukaryota>viridiplantae	Selaginella	moellendorffii	hypothetical protein SELMODRAFT_441115 [Selaginella moellendorffii]
XP_002978414.1	316	SELMODRAFT_418284	eukaryota>viridiplantae	Selaginella	moellendorffii	hypothetical protein SELMODRAFT_418284 [Selaginella moellendorffii]
CDW80608.1	314	Contig5165.g5534	eukaryota>alveolata>ciliophora	Stylonychia	lemnae	superoxide dismutase [Stylonychia lemnae]
XP_005768431.1	313	EMIHUDRAFT_210980	eukaryota>haptophyceae	Emiliania	huxleyi CCMP1516	hypothetical protein EMIHUDRAFT_210980 [Emiliania huxleyi CCMP1516]
XP_005770971.1	313	EMIHUDRAFT_243748	eukaryota>haptophyceae	Emiliania	huxleyi CCMP1516	hypothetical protein EMIHUDRAFT_243748 [Emiliania huxleyi CCMP1516]
XP_002125402.1	312	LOC100180182	eukaryota>metazoa>chordata	Ciona	intestinalis	PREDICTED: calcium release-activated calcium channel protein 1 isoform X2 [Ciona intestinalis]
XP_005763455.1	312	EMIHUDRAFT_446144	eukaryota>haptophyceae	Emiliania	huxleyi CCMP1516	hypothetical protein EMIHUDRAFT_446144 [Emiliania huxleyi CCMP1516]
XP_005767816.1	312	EMIHUDRAFT_436817	eukaryota>haptophyceae	Emiliania	huxleyi CCMP1516	hypothetical protein EMIHUDRAFT_436817 [Emiliania huxleyi CCMP1516]
XP_012196003.1	311	SPRG_02056	eukaryota>stramenopiles	Saprolegnia	parasitica CBS 223.65	hypothetical protein SPRG_02056 [Saprolegnia parasitica CBS 223.65]
XP_003249558.1	310	LOC412524	eukaryota>metazoa>hexapoda	Apis	mellifera	PREDICTED: calcium release-activated calcium channel protein 1 isoform X1 [Apis mellifera]
XP_009518362.1	309	PHYSODRAFT_486152	eukaryota>stramenopiles	Phytophthora	sojae	hypothetical protein PHYSODRAFT_486152 [Phytophthora sojae]
OEU19244.1	307	FRACYDRAFT_235292	eukaryota>stramenopiles	Fragilariopsis	cylindrus CCMP1102	hypothetical protein FRACYDRAFT_235292 [Fragilariopsis cylindrus CCMP1102]
XP_008612529.1	306	SDRG_08420	eukaryota>stramenopiles	Saprolegnia	diclina VS20	hypothetical protein SDRG_08420 [Saprolegnia diclina VS20]
NP_780632.1	304	D730049H07Rik,orai-1,Tmem142a	eukaryota>metazoa>chordata>vertebrata	Mus	musculus	calcium release-activated calcium channel protein 1 [Mus musculus]
XP_005645699.1	304	COCSUDRAFT_17677	eukaryota>viridiplantae>chlorophyta	Coccomyxa	subellipsoidea C-169	hypothetical protein COCSUDRAFT_17677, partial [Coccomyxa subellipsoidea C-169]
XP_008612530.1	304	SDRG_08420	eukaryota>stramenopiles	Saprolegnia	diclina VS20	hypothetical protein, variant [Saprolegnia diclina VS20]
XP_009518359.1	304	PHYSODRAFT_295628	eukaryota>stramenopiles	Phytophthora	sojae	hypothetical protein PHYSODRAFT_295628 [Phytophthora sojae]
NP_116179.2	301	CRACM1,IMD9,ORAT1,TAM2,TMEM142A	eukaryota>metazoa>chordata>vertebrata	Homo	sapiens	calcium release-activated calcium channel protein 1 [Homo sapiens]
XP_008861378.1	301	H310_00383	eukaryota>stramenopiles	Aphanomyces	invadans	hypothetical protein H310_00383 [Aphanomyces invadans]
XP_011142627.1	301	LOC105185086	eukaryota>metazoa>hexapoda	Harpegnathos	saltator	PREDICTED: calcium release-activated calcium channel protein 1 isoform X1 [Harpegnathos saltator]
EJY70280.1	300	OXYTRI_08975	eukaryota>alveolata>ciliophora	Oxytricha	trifallax	Superoxide dismutase [Cu-Zn] (macronuclear) [Oxytricha trifallax]
XP_005591748.1	300	ORAI3	eukaryota>metazoa>chordata>vertebrata	Macaca	fascicularis	PREDICTED: protein orai-3 isoform X1 [Macaca fascicularis]
XP_004928907.1	299	LOC101739927	eukaryota>metazoa>hexapoda	Bombyx	mori	calcium release-activated calcium channel protein 1 isoform X6 [Bombyx mori]
NP_001254835.1	297	orai-1	eukaryota>metazoa>nematoda	Caenorhabditis	elegans	ORAI (store-operated calcium channel) homolog [Caenorhabditis elegans]
XP_002984309.1	297	SELMODRAFT_423451	eukaryota>viridiplantae	Selaginella	moellendorffii	hypothetical protein SELMODRAFT_423451 [Selaginella moellendorffii]
EJK58875.1	296	THAOC_20967	eukaryota>stramenopiles	Thalassiosira	oceanica	hypothetical protein THAOC_20967 [Thalassiosira oceanica]
NP_689501.1	295	TMEM142C	eukaryota>metazoa>chordata>vertebrata	Homo	sapiens	protein orai-3 [Homo sapiens]
XP_003280496.1	295	ORAI3	eukaryota>metazoa>chordata>vertebrata	Nomascus	leucogenys	PREDICTED: protein orai-3 [Nomascus leucogenys]
XP_005591749.1	295	ORAI3	eukaryota>metazoa>chordata>vertebrata	Macaca	fascicularis	PREDICTED: protein orai-3 isoform X2 [Macaca fascicularis]
NP_001254834.1	293	orai-1	eukaryota>metazoa>nematoda	Caenorhabditis	elegans	Protein orai [Caenorhabditis elegans]
XP_009822282.1	292	H257_01002	eukaryota>stramenopiles	Aphanomyces	astaci	hypothetical protein H257_01002 [Aphanomyces astaci]
XP_002898394.1	291	PITG_15443	eukaryota>stramenopiles	Phytophthora	infestans T30-4	conserved hypothetical protein [Phytophthora infestans T30-4]
NP_940816.1	290	9930124N15,BC061259,Tmem142c	eukaryota>metazoa>chordata>vertebrata	Mus	musculus	protein orai-3 [Mus musculus]
XP_003881962.1	289	NCLIV_017210	eukaryota>alveolata>apicomplexa	Neospora	caninum Liverpool	conserved hypothetical protein [Neospora caninum Liverpool]
XP_003882987.1	288	NCLIV_027440	eukaryota>alveolata>apicomplexa	Neospora	caninum Liverpool	conserved hypothetical protein [Neospora caninum Liverpool]
XP_018637036.1	286	TGME49_259050	eukaryota>alveolata>apicomplexa	Toxoplasma	gondii ME49	hypothetical protein TGME49_259050 [Toxoplasma gondii ME49]
XP_008883102.1	283	HHA_259050	eukaryota>alveolata>apicomplexa	Hammondia	hammondi	hypothetical protein HHA_259050 [Hammondia hammondi]
XP_005165627.1	281	si:dkey-261j4.7	eukaryota>metazoa>chordata>vertebrata>actinopterygii	Danio	rerio	protein orai-2 isoform X1 [Danio rerio]
XP_002183393.1	280	PHATRDRAFT_48896	eukaryota>stramenopiles	Phaeodactylum	tricornutum CCAP 1055/1	predicted protein [Phaeodactylum tricornutum CCAP 1055/1]
XP_017315171.1	280	LOC108259892	eukaryota>metazoa>chordata>vertebrata>actinopterygii	Ictalurus	punctatus	PREDICTED: protein orai-2-like [Ictalurus punctatus]
XP_017315172.1	280	LOC108259892	eukaryota>metazoa>chordata>vertebrata>actinopterygii	Ictalurus	punctatus	PREDICTED: protein orai-2-like [Ictalurus punctatus]
XP_017315173.1	280	LOC108259892	eukaryota>metazoa>chordata>vertebrata>actinopterygii	Ictalurus	punctatus	PREDICTED: protein orai-2-like [Ictalurus punctatus]
OEU18309.1	280	FRACYDRAFT_260338	eukaryota>stramenopiles	Fragilariopsis	cylindrus CCMP1102	hypothetical protein FRACYDRAFT_260338 [Fragilariopsis cylindrus CCMP1102]
XP_011142628.1	276	LOC105185086	eukaryota>metazoa>hexapoda	Harpegnathos	saltator	PREDICTED: calcium release-activated calcium channel protein 1 isoform X2 [Harpegnathos saltator]
CBY24244.1	275	GSOID_T00009597001	eukaryota>metazoa>chordata	Oikopleura	dioica	unnamed protein product [Oikopleura dioica]
CDW77297.1	275	Contig239.g274	eukaryota>alveolata>ciliophora	Stylonychia	lemnae	UNKNOWN [Stylonychia lemnae]
XP_003249560.1	271	LOC412524	eukaryota>metazoa>hexapoda	Apis	mellifera	PREDICTED: calcium release-activated calcium channel protein 1 isoform X2 [Apis mellifera]
XP_016766861.1	271	LOC412524	eukaryota>metazoa>hexapoda	Apis	mellifera	PREDICTED: calcium release-activated calcium channel protein 1 isoform X2 [Apis mellifera]
XP_395979.4	271	LOC412524	eukaryota>metazoa>hexapoda	Apis	mellifera	PREDICTED: calcium release-activated calcium channel protein 1 isoform X2 [Apis mellifera]
XP_009845876.1	270	H257_18499	eukaryota>stramenopiles	Aphanomyces	astaci	hypothetical protein H257_18499 [Aphanomyces astaci]
XP_002287927.1	269	THAPSDRAFT_2649	eukaryota>stramenopiles	Thalassiosira	pseudonana CCMP1335	predicted protein [Thalassiosira pseudonana CCMP1335]
XP_013228158.1	269	ETH_00032955	eukaryota>alveolata>apicomplexa	Eimeria	tenella	hypothetical protein, conserved [Eimeria tenella]
CEM24399.1	268	Vbra_3163	eukaryota>alveolata	Vitrella	brassicaformis CCMP3155	unnamed protein product [Vitrella brassicaformis CCMP3155]
NP_001159919.1	267	si:dkey-261j4.7	eukaryota>metazoa>chordata>vertebrata>actinopterygii	Danio	rerio	protein orai-2 [Danio rerio]
XP_005787471.1	267	EMIHUDRAFT_441020	eukaryota>haptophyceae	Emiliania	huxleyi CCMP1516	hypothetical protein EMIHUDRAFT_441020 [Emiliania huxleyi CCMP1516]
XP_006504492.1	264	A730041O15Rik,Tmem142b	eukaryota>metazoa>chordata>vertebrata	Mus	musculus	PREDICTED: protein orai-2 isoform X1 [Mus musculus]
XP_002972478.1	262	SELMODRAFT_412890	eukaryota>viridiplantae	Selaginella	moellendorffii	hypothetical protein SELMODRAFT_412890 [Selaginella moellendorffii]
XP_003242941.1	262	LOC100163796	eukaryota>metazoa>hexapoda	Acyrthosiphon	pisum	PREDICTED: calcium release-activated calcium channel protein 1 isoform X1 [Acyrthosiphon pisum]
XP_008873800.1	262	H310_09487	eukaryota>stramenopiles	Aphanomyces	invadans	hypothetical protein H310_09487 [Aphanomyces invadans]
XP_016658022.1	262	LOC100163796	eukaryota>metazoa>hexapoda	Acyrthosiphon	pisum	PREDICTED: calcium release-activated calcium channel protein 1 isoform X1 [Acyrthosiphon pisum]
XP_002900394.1	261	PITG_12515	eukaryota>stramenopiles	Phytophthora	infestans T30-4	conserved hypothetical protein [Phytophthora infestans T30-4]
XP_003276689.1	261	ORAI2	eukaryota>metazoa>chordata>vertebrata	Nomascus	leucogenys	PREDICTED: protein orai-2 isoform X1 [Nomascus leucogenys]
XP_003276690.1	261	ORAI2	eukaryota>metazoa>chordata>vertebrata	Nomascus	leucogenys	PREDICTED: protein orai-2 isoform X1 [Nomascus leucogenys]
XP_004090955.1	261	ORAI2	eukaryota>metazoa>chordata>vertebrata	Nomascus	leucogenys	PREDICTED: protein orai-2 isoform X1 [Nomascus leucogenys]
XP_005549365.1	261	ORAI2	eukaryota>metazoa>chordata>vertebrata	Macaca	fascicularis	PREDICTED: protein orai-2 [Macaca fascicularis]
XP_005549366.1	261	ORAI2	eukaryota>metazoa>chordata>vertebrata	Macaca	fascicularis	PREDICTED: protein orai-2 [Macaca fascicularis]
XP_009822285.1	261	H257_01004	eukaryota>stramenopiles	Aphanomyces	astaci	hypothetical protein H257_01004 [Aphanomyces astaci]
XP_012351889.1	261	ORAI2	eukaryota>metazoa>chordata>vertebrata	Nomascus	leucogenys	PREDICTED: protein orai-2 isoform X1 [Nomascus leucogenys]
XP_008902125.1	260	PPTG_09031	eukaryota>stramenopiles	Phytophthora	parasitica INRA-310	hypothetical protein PPTG_09031 [Phytophthora parasitica INRA-310]
NP_001089486.1	258	orai-1,orai1,orai2,tmem142a,tmem142b	eukaryota>metazoa>chordata>vertebrata	Xenopus	laevis	calcium release-activated calcium channel protein 1 [Xenopus laevis]
NP_001128236.1	258	Orai1	eukaryota>metazoa>hexapoda	Bombyx	mori	ORAI calcium release-activated calcium modulator 1 isoform A [Bombyx mori]
XP_003970967.1	258	LOC101071969	eukaryota>metazoa>chordata>vertebrata>actinopterygii	Takifugu	rubripes	PREDICTED: protein orai-2-like [Takifugu rubripes]
XP_009532260.1	258	PHYSODRAFT_440123	eukaryota>stramenopiles	Phytophthora	sojae	hypothetical protein PHYSODRAFT_440123, partial [Phytophthora sojae]
XP_015151193.1	258	ORAI2	eukaryota>metazoa>chordata>vertebrata	Gallus	gallus	PREDICTED: protein orai-2 isoform X1 [Gallus gallus]
XP_018118765.1	258	LOC108716807	eukaryota>metazoa>chordata>vertebrata	Xenopus	laevis	PREDICTED: calcium release-activated calcium channel protein 1-like [Xenopus laevis]
NP_001084444.1	257	orai1,orai2,tmem142a	eukaryota>metazoa>chordata>vertebrata	Xenopus	laevis	protein orai-2 [Xenopus laevis]
NP_001025881.1	257	ORAI2	eukaryota>metazoa>chordata>vertebrata	Gallus	gallus	protein orai-2 [Gallus gallus]
XP_018101168.1	257	LOC108707681	eukaryota>metazoa>chordata>vertebrata	Xenopus	laevis	PREDICTED: protein orai-2-like [Xenopus laevis]
XP_005837144.1	257	GUITHDRAFT_135348	eukaryota>cryptophyta	Guillardia	theta CCMP2712	hypothetical protein GUITHDRAFT_135348 [Guillardia theta CCMP2712]
CEM07521.1	255	Vbra_14456	eukaryota>alveolata	Vitrella	brassicaformis CCMP3155	unnamed protein product [Vitrella brassicaformis CCMP3155]
ERG84396.1	254	ASU_05431	eukaryota>metazoa>nematoda	Ascaris	suum	protein orai [Ascaris suum]
NP_001119812.1	254	C7orf19,CBCIP2,MEM142B,TMEM142B	eukaryota>metazoa>chordata>vertebrata	Homo	sapiens	protein orai-2 isoform a [Homo sapiens]
NP_001258747.1	254	C7orf19,CBCIP2,MEM142B,TMEM142B	eukaryota>metazoa>chordata>vertebrata	Homo	sapiens	protein orai-2 isoform a [Homo sapiens]
NP_116220.1	254	C7orf19,CBCIP2,MEM142B,TMEM142B	eukaryota>metazoa>chordata>vertebrata	Homo	sapiens	protein orai-2 isoform a [Homo sapiens]
XP_008612528.1	254	SDRG_08419	eukaryota>stramenopiles	Saprolegnia	diclina VS20	hypothetical protein SDRG_08419 [Saprolegnia diclina VS20]
XP_012196002.1	254	SPRG_02055	eukaryota>stramenopiles	Saprolegnia	parasitica CBS 223.65	hypothetical protein SPRG_02055 [Saprolegnia parasitica CBS 223.65]
XP_012351890.1	254	ORAI2	eukaryota>metazoa>chordata>vertebrata	Nomascus	leucogenys	PREDICTED: protein orai-2 isoform X2 [Nomascus leucogenys]
XP_014676992.1	254	LOC106816863	eukaryota>metazoa	Priapulus	caudatus	PREDICTED: protein orai-3-like [Priapulus caudatus]
XP_008861380.1	252	H310_00385	eukaryota>stramenopiles	Aphanomyces	invadans	hypothetical protein H310_00385 [Aphanomyces invadans]
XP_008861379.1	251	H310_00384	eukaryota>stramenopiles	Aphanomyces	invadans	hypothetical protein H310_00384 [Aphanomyces invadans]
CBJ27923.1	250	Esi_0087_0042	eukaryota>stramenopiles	Ectocarpus	siliculosus	conserved unknown protein [Ectocarpus siliculosus]
NP_848866.2	250	A730041O15Rik,Tmem142b	eukaryota>metazoa>chordata>vertebrata	Mus	musculus	protein orai-2 [Mus musculus]
XP_006504493.1	250	A730041O15Rik,Tmem142b	eukaryota>metazoa>chordata>vertebrata	Mus	musculus	PREDICTED: protein orai-2 isoform X2 [Mus musculus]
XP_006504494.1	250	A730041O15Rik,Tmem142b	eukaryota>metazoa>chordata>vertebrata	Mus	musculus	PREDICTED: protein orai-2 isoform X2 [Mus musculus]
EJK72087.1	249	THAOC_06417	eukaryota>stramenopiles	Thalassiosira	oceanica	hypothetical protein THAOC_06417 [Thalassiosira oceanica]
XP_009822283.1	249	H257_01003	eukaryota>stramenopiles	Aphanomyces	astaci	hypothetical protein H257_01003 [Aphanomyces astaci]
XP_972151.1	249	LOC660858	eukaryota>metazoa>hexapoda	Tribolium	castaneum	PREDICTED: calcium release-activated calcium channel protein 1 [Tribolium castaneum]
XP_008612527.1	248	SDRG_08418	eukaryota>stramenopiles	Saprolegnia	diclina VS20	hypothetical protein SDRG_08418 [Saprolegnia diclina VS20]
XP_012196001.1	248	SPRG_02054	eukaryota>stramenopiles	Saprolegnia	parasitica CBS 223.65	hypothetical protein SPRG_02054 [Saprolegnia parasitica CBS 223.65]
XP_013775850.1	248	LOC106460669	eukaryota>metazoa	Limulus	polyphemus	calcium release-activated calcium channel protein 1-like isoform X1 [Limulus polyphemus]
XP_005829825.1	248	GUITHDRAFT_153454	eukaryota>cryptophyta	Guillardia	theta CCMP2712	hypothetical protein GUITHDRAFT_153454 [Guillardia theta CCMP2712]
CDS42430.1	245	EmuJ_001014000	eukaryota>metazoa	Echinococcus	multilocularis	calcium release activated calcium channel [Echinococcus multilocularis]
XP_009532262.1	245	PHYSODRAFT_515195	eukaryota>stramenopiles	Phytophthora	sojae	hypothetical protein PHYSODRAFT_515195 [Phytophthora sojae]
XP_002900392.1	244	PITG_12513	eukaryota>stramenopiles	Phytophthora	infestans T30-4	conserved hypothetical protein [Phytophthora infestans T30-4]

[illegible]

XP_004092266.1	116	RCAN3	eukaryota>metazoa>chordata>vertebrata	Nomascus	leucogenys	PREDICTED: calcipressin-3 isoform X3 [Nomascus leucogenys]
XP_012353797.1	116	RCAN3	eukaryota>metazoa>chordata>vertebrata	Nomascus	leucogenys	PREDICTED: calcipressin-3 isoform X3 [Nomascus leucogenys]
XP_006825325.1	102	LOC102806619	eukaryota>metazoa>hemichordata	Saccoglossus	kowalevskii	PREDICTED: protein sarah-like, partial [Saccoglossus kowalevskii]
XP_001745353.1	53	MONBRDRAFT_3061	eukaryota>choanoflagellida	Monosiga	brevicollis MX1	hypothetical protein, partial [Monosiga brevicollis MX1]

T. STIM

XP_017317088.1	1028	LOC108260897	eukaryota>metazoa>chordata>vertebrata>actinopterygii	Ictalurus	punctatus	PREDICTED: stromal interaction molecule 2-like [Ictalurus punctatus]
XP_017317089.1	1028	LOC108260897	eukaryota>metazoa>chordata>vertebrata>actinopterygii	Ictalurus	punctatus	PREDICTED: stromal interaction molecule 2-like [Ictalurus punctatus]
XP_017317090.1	1028	LOC108260897	eukaryota>metazoa>chordata>vertebrata>actinopterygii	Ictalurus	punctatus	PREDICTED: stromal interaction molecule 2-like [Ictalurus punctatus]
XP_017317091.1	1028	LOC108260897	eukaryota>metazoa>chordata>vertebrata>actinopterygii	Ictalurus	punctatus	PREDICTED: stromal interaction molecule 2-like [Ictalurus punctatus]
XP_017317092.1	1028	LOC108260897	eukaryota>metazoa>chordata>vertebrata>actinopterygii	Ictalurus	punctatus	PREDICTED: stromal interaction molecule 2-like [Ictalurus punctatus]
XP_002660454.3	992	stim2a	eukaryota>metazoa>chordata>vertebrata>actinopterygii	Danio	rerio	stromal interaction molecule 2 isoform X1 [Danio rerio]
XP_002154218.2	858	LOC100198735	eukaryota>metazoa>cnidaria	Hydra	vulgaris	PREDICTED: uncharacterized protein LOC100198735, partial [Hydra vulgaris]
XP_003258607.1	841	STIM2	eukaryota>metazoa>chordata>vertebrata	Nomascus	leucogenys	PREDICTED: stromal interaction molecule 2 isoform X1 [Nomascus leucogenys]
XP_005554693.1	840	STIM2	eukaryota>metazoa>chordata>vertebrata	Macaca	fascicularis	PREDICTED: stromal interaction molecule 2 isoform X1 [Macaca fascicularis]
XP_003258605.1	833	STIM2	eukaryota>metazoa>chordata>vertebrata	Nomascus	leucogenys	PREDICTED: stromal interaction molecule 2 isoform X2 [Nomascus leucogenys]
XP_005554694.1	832	STIM2	eukaryota>metazoa>chordata>vertebrata	Macaca	fascicularis	PREDICTED: stromal interaction molecule 2 isoform X2 [Macaca fascicularis]
XP_006503745.1	820	Stim2	eukaryota>metazoa>chordata>vertebrata	Mus	musculus	PREDICTED: stromal interaction molecule 2 isoform X1 [Mus musculus]
CAG04573.1	820	GSTEN:00024339:G:001	eukaryota>metazoa>chordata>vertebrata>actinopterygii	Tetraodon	nigroviridis	unnamed protein product, partial [Tetraodon nigroviridis]
XP_013773068.1	809	LOC106458154	eukaryota>metazoa	Limulus	polyphemus	PREDICTED: uncharacterized protein LOC106458154 [Limulus polyphemus]
XP_005173449.1	808	si:dkey-24p1.5	eukaryota>metazoa>chordata>vertebrata>actinopterygii	Danio	rerio	stromal interaction molecule 1 isoform X1 [Danio rerio]
XP_006507596.1	793	SIM	eukaryota>metazoa>chordata>vertebrata	Mus	musculus	PREDICTED: stromal interaction molecule 1 isoform X1 [Mus musculus]
XP_004090689.1	792	STIM1	eukaryota>metazoa>chordata>vertebrata	Nomascus	leucogenys	PREDICTED: stromal interaction molecule 1 isoform X1 [Nomascus leucogenys]
NP_001264890.1	791	D11S4896E,GOK,IMD10,STRMK,TAM,TAM1	eukaryota>metazoa>chordata>vertebrata	Homo	sapiens	stromal interaction molecule 1 isoform 1 precursor [Homo sapiens]
XP_005579046.1	784	STIM1	eukaryota>metazoa>chordata>vertebrata	Macaca	fascicularis	PREDICTED: stromal interaction molecule 1 isoform X1 [Macaca fascicularis]
XP_005173450.1	781	si:dkey-24p1.5	eukaryota>metazoa>chordata>vertebrata>actinopterygii	Danio	rerio	stromal interaction molecule 1 isoform X2 [Danio rerio]
NP_001162589.1	754	STIM2	eukaryota>metazoa>chordata>vertebrata	Homo	sapiens	stromal interaction molecule 2 isoform 1 precursor [Homo sapiens]
XP_009162000.1	753	T265_00079	eukaryota>metazoa	Opisthorchis	viverrini	hypothetical protein T265_00079 [Opisthorchis viverrini]
XP_017327752.1	753	stim2	eukaryota>metazoa>chordata>vertebrata>actinopterygii	Ictalurus	punctatus	PREDICTED: stromal interaction molecule 2 isoform X1 [Ictalurus punctatus]
NP_001074572.2	746	Stim2	eukaryota>metazoa>chordata>vertebrata	Mus	musculus	stromal interaction molecule 2 precursor [Mus musculus]
NP_065911.3	746	STIM2	eukaryota>metazoa>chordata>vertebrata	Homo	sapiens	stromal interaction molecule 2 isoform 2 precursor [Homo sapiens]
XP_018098572.1	746	LOC108706560	eukaryota>metazoa>chordata>vertebrata	Xenopus	laevis	PREDICTED: stromal interaction molecule 2-like [Xenopus laevis]
XP_420749.5	740	STIM2	eukaryota>metazoa>chordata>vertebrata	Gallus	gallus	PREDICTED: stromal interaction molecule 2 isoform X1 [Gallus gallus]
XP_011605056.1	736	stim2	eukaryota>metazoa>chordata>vertebrata>actinopterygii	Takifugu	rubripes	PREDICTED: stromal interaction molecule 2 [Takifugu rubripes]
XP_692356.4	727	stim2b	eukaryota>metazoa>chordata>vertebrata>actinopterygii	Danio	rerio	stromal interaction molecule 2 [Danio rerio]
XP_018124030.1	722	LOC108719591	eukaryota>metazoa>chordata>vertebrata	Xenopus	laevis	PREDICTED: stromal interaction molecule 2-like [Xenopus laevis]
XP_017327753.1	722	stim2	eukaryota>metazoa>chordata>vertebrata>actinopterygii	Ictalurus	punctatus	PREDICTED: stromal interaction molecule 2 isoform X2 [Ictalurus punctatus]
XP_011439185.1	720	LOC105336527	eukaryota>metazoa>mollusca	Crassostrea	gigas	PREDICTED: stromal interaction molecule 1 isoform X1 [Crassostrea gigas]
XP_006507597.1	716	SIM	eukaryota>metazoa>chordata>vertebrata	Mus	musculus	PREDICTED: stromal interaction molecule 1 isoform X2 [Mus musculus]
XP_005579047.1	715	STIM1	eukaryota>metazoa>chordata>vertebrata	Macaca	fascicularis	PREDICTED: stromal interaction molecule 1 isoform X2 [Macaca fascicularis]
XP_011439186.1	712	LOC105336527	eukaryota>metazoa>mollusca	Crassostrea	gigas	PREDICTED: stromal interaction molecule 1 isoform X2 [Crassostrea gigas]
XP_009290146.1	703	si:dkey-24p1.5	eukaryota>metazoa>chordata>vertebrata>actinopterygii	Danio	rerio	stromal interaction molecule 1 isoform X3 [Danio rerio]
XP_013386698.1	701	LOC106156136	eukaryota>metazoa	Lingula	anatina	PREDICTED: stromal interaction molecule homolog [Lingula anatina]
XP_011439187.1	701	LOC105336527	eukaryota>metazoa>mollusca	Crassostrea	gigas	PREDICTED: stromal interaction molecule 1 isoform X3 [Crassostrea gigas]
XP_002126383.1	700	LOC100178893	eukaryota>metazoa>chordata	Ciona	intestinalis	PREDICTED: stromal interaction molecule homolog isoform X1 [Ciona intestinalis]
XP_002598264.1	698	BRAFLDRAFT_119144	eukaryota>metazoa>chordata	Branchiostoma	floridae	hypothetical protein BRAFLDRAFT_119144 [Branchiostoma floridae]
XP_009857576.1	698	LOC100178893	eukaryota>metazoa>chordata	Ciona	intestinalis	PREDICTED: stromal interaction molecule homolog isoform X2 [Ciona intestinalis]
XP_012551138.1	695	Stim1	eukaryota>metazoa>hexapoda	Bombyx	mori	stromal interaction molecule 1 isoform X1 [Bombyx mori]
XP_012551147.1	694	Stim1	eukaryota>metazoa>hexapoda	Bombyx	mori	stromal interaction molecule 1 isoform X2 [Bombyx mori]
XP_011439188.1	693	LOC105336527	eukaryota>metazoa>mollusca	Crassostrea	gigas	PREDICTED: stromal interaction molecule 1 isoform X4 [Crassostrea gigas]
Smar1000002138	692	Smar1000002138	eukaryota>metazoa>arthropoda>myriapoda	Strigamia	maritima	>Smar1000002138
XP_005554695.1	692	STIM2	eukaryota>metazoa>chordata>vertebrata	Macaca	fascicularis	PREDICTED: stromal interaction molecule 2 isoform X3 [Macaca fascicularis]
XP_011609388.1	692	LOC101077194	eukaryota>metazoa>chordata>vertebrata>actinopterygii	Takifugu	rubripes	PREDICTED: stromal interaction molecule 1-like isoform X1 [Takifugu rubripes]
XP_011609389.1	692	LOC101077194	eukaryota>metazoa>chordata>vertebrata>actinopterygii	Takifugu	rubripes	PREDICTED: stromal interaction molecule 1-like isoform X1 [Takifugu rubripes]
XP_011609390.1	691	LOC101077194	eukaryota>metazoa>chordata>vertebrata>actinopterygii	Takifugu	rubripes	PREDICTED: stromal interaction molecule 1-like isoform X2 [Takifugu rubripes]
XP_011609391.1	690	LOC101077194	eukaryota>metazoa>chordata>vertebrata>actinopterygii	Takifugu	rubripes	PREDICTED: stromal interaction molecule 1-like isoform X3 [Takifugu rubripes]
XP_395207.4	687	LOC411739	eukaryota>metazoa>hexapoda	Apis	mellifera	PREDICTED: stromal interaction molecule homolog isoform X1 [Apis mellifera]
XP_003258606.1	686	STIM2	eukaryota>metazoa>chordata>vertebrata	Nomascus	leucogenys	PREDICTED: stromal interaction molecule 2 isoform X3 [Nomascus leucogenys]
NP_003147.2	685	D11S4896E,GOK,IMD10,STRMK,TAM,TAM1	eukaryota>metazoa>chordata>vertebrata	Homo	sapiens	stromal interaction molecule 1 isoform 2 precursor [Homo sapiens]
NP_033313.2	685	SIM	eukaryota>metazoa>chordata>vertebrata	Mus	musculus	stromal interaction molecule 1 precursor [Mus musculus]
XP_003254842.1	685	STIM1	eukaryota>metazoa>chordata>vertebrata	Nomascus	leucogenys	PREDICTED: stromal interaction molecule 1 isoform X2 [Nomascus leucogenys]
XP_005579048.1	684	STIM1	eukaryota>metazoa>chordata>vertebrata	Macaca	fascicularis	PREDICTED: stromal interaction molecule 1 isoform X3 [Macaca fascicularis]
XP_018673097.1	677	LOC100178893	eukaryota>metazoa>chordata	Ciona	intestinalis	PREDICTED: stromal interaction molecule homolog isoform X3 [Ciona intestinalis]
NP_001038264.1	676	si:dkey-24p1.5	eukaryota>metazoa>chordata>vertebrata>actinopterygii	Danio	rerio	stromal interaction molecule 1 precursor [Danio rerio]
XP_011142898.1	676	LOC105185257	eukaryota>metazoa>hexapoda	Harpegnathos	saltator	PREDICTED: stromal interaction molecule homolog [Harpegnathos saltator]
XP_011606881.1	671	LOC101077340	eukaryota>metazoa>chordata>vertebrata>actinopterygii	Takifugu	rubripes	PREDICTED: stromal interaction molecule 1-like [Takifugu rubripes]
XP_013776985.1	669	LOC106461685	eukaryota>metazoa	Limulus	polyphemus	PREDICTED: stromal interaction molecule homolog isoform X1 [Limulus polyphemus]
XP_018105965.1	669	LOC108710082	eukaryota>metazoa>chordata>vertebrata	Xenopus	laevis	PREDICTED: stromal interaction molecule 1-like isoform X1 [Xenopus laevis]
XP_017346476.1	669	LOC108277929	eukaryota>metazoa>chordata>vertebrata>actinopterygii	Ictalurus	punctatus	PREDICTED: stromal interaction molecule 1-like [Ictalurus punctatus]
NP_001090506.1	668	stim1,stim1.S	eukaryota>metazoa>chordata>vertebrata	Xenopus	laevis	stromal interaction molecule 1 L homeolog precursor [Xenopus laevis]
XP_018105966.1	668	LOC108710082	eukaryota>metazoa>chordata>vertebrata	Xenopus	laevis	PREDICTED: stromal interaction molecule 1-like isoform X2 [Xenopus laevis]
XP_005161345.1	668	si:ch211-112117.2,stim1	eukaryota>metazoa>chordata>vertebrata>actinopterygii	Danio	rerio	stromal interaction molecule 1 isoform X1 [Danio rerio]
XP_015778612.1	667	LOC107356518	eukaryota>metazoa>cnidaria	Acropora	digitifera	PREDICTED: stromal interaction molecule 1-like [Acropora digitifera]
XP_017348463.1	666	stim1	eukaryota>metazoa>chordata>vertebrata>actinopterygii	Ictalurus	punctatus	PREDICTED: stromal interaction molecule 1 isoform X1 [Ictalurus punctatus]
XP_003970828.1	661	LOC101077194	eukaryota>metazoa>chordata>vertebrata>actinopterygii	Takifugu	rubripes	PREDICTED: stromal interaction molecule 1-like isoform X4 [Takifugu rubripes]
XP_001951196.2	657	LOC100166274	eukaryota>metazoa>hexapoda	Acyrthosiphon	pisum	PREDICTED: stromal interaction molecule homolog isoform X1 [Acyrthosiphon pisum]
CAF95381.1	656	GSTEN:00012009:G:001	eukaryota>metazoa>chordata>vertebrata>actinopterygii	Tetraodon	nigroviridis	unnamed protein product, partial [Tetraodon nigroviridis]
XP_008192163.1	652	LOC664047	eukaryota>metazoa>hexapoda	Tribolium	castaneum	PREDICTED: stromal interaction molecule homolog isoform X1 [Tribolium castaneum]
XP_019853545.1	650	LOC100633433	eukaryota>metazoa	Amphimedon	queenslandica	PREDICTED: stromal interaction molecule 1-like isoform X1 [Amphimedon queenslandica]
XP_019853546.1	650	LOC100633433	eukaryota>metazoa	Amphimedon	queenslandica	PREDICTED: stromal interaction molecule 1-like isoform X1 [Amphimedon queenslandica]
XP_003387480.1	649	LOC100633433	eukaryota>metazoa	Amphimedon	queenslandica	PREDICTED: stromal interaction molecule 1-like isoform X2 [Amphimedon queenslandica]
CDS35874.1	646	EmuJ_001183000	eukaryota>metazoa	Echinococcus	multilocularis	stromal interaction molecule 1 [Echinococcus multilocularis]
XP_975159.2	646	LOC664047	eukaryota>metazoa>hexapoda	Tribolium	castaneum	PREDICTED: stromal interaction molecule homolog isoform X2 [Tribolium castaneum]
XP_004936189.2	642	STIM2	eukaryota>metazoa>chordata>vertebrata	Gallus	gallus	PREDICTED: stromal interaction molecule 2 isoform X2 [Gallus gallus]
XP_006507598.1	642	SIM	eukaryota>metazoa>chordata>vertebrata	Mus	musculus	PREDICTED: stromal interaction molecule 1 isoform X3 [Mus musculus]
XP_017177567.1	642	SIM	eukaryota>metazoa>chordata>vertebrata	Mus	musculus	PREDICTED: stromal interaction molecule 1 isoform X3 [Mus musculus]
NP_001189470.1	642	si:ch211-112117.2,stim1	eukaryota>metazoa>chordata>vertebrata>actinopterygii	Danio	rerio	stromal interaction molecule 1 precursor [Danio rerio]
XP_009293832.1	641	si:ch211-112117.2,stim1	eukaryota>metazoa>chordata>vertebrata>actinopterygii	Danio	rerio	stromal interaction molecule 1 isoform X2 [Danio rerio]
XP_017348464.1	641	stim1	eukaryota>metazoa>chordata>vertebrata>actinopterygii	Ictalurus	punctatus	PREDICTED: stromal interaction molecule 1 isoform X2 [Ictalurus punctatus]
XP_006818739.1	639	LOC100366516	eukaryota>metazoa>hemichordata	Saccoglossus	kowalevskii	PREDICTED: stromal interaction molecule 1-like [Saccoglossus kowalevskii]
XP_008186734.1	635	LOC100166274	eukaryota>metazoa>hexapoda	Acyrthosiphon	pisum	PREDICTED: stromal interaction molecule homolog isoform X2 [Acyrthosiphon pisum]
XP_003976755.2	631	LOC101080161	eukaryota>metazoa>chordata>vertebrata>actinopterygii	Takifugu	rubripes	PREDICTED: LOW QUALITY PROTEIN: stromal interaction molecule 2-like, partial [Takifugu rubripes]
XP_008192165.1	631	LOC664047	eukaryota>metazoa>hexapoda	Tribolium	castaneum	PREDICTED: stromal interaction molecule homolog isoform X3 [Tribolium castaneum]
XP_013407301.1	627	LOC106171477	eukaryota>metazoa	Lingula	anatina	PREDICTED: stromal interaction molecule 1-like [Lingula anatina]
XP_008192166.1	625	LOC664047	eukaryota>metazoa>hexapoda	Tribolium	castaneum	PREDICTED: stromal interaction molecule homolog isoform X4 [Tribolium castaneum]
XP_013776228.1	616	LOC106461002	eukaryota>metazoa	Limulus	polyphemus	PREDICTED: stromal interaction molecule homolog, partial [Limulus polyphemus]
NP_001026009.1	612	STIM1	eukaryota>metazoa>chordata>vertebrata	Gallus	gallus	stromal interaction molecule 1 [Gallus gallus]
XP_009031508.1	609	HELRODRAFT_189915	eukaryota>metazoa>annelida	Helobdella	robusta	hypothetical protein HELRODRAFT_189915 [Helobdella robusta]
XP_017327754.1	607	stim2	eukaryota>metazoa>chordata>vertebrata>actinopterygii	Ictalurus	punctatus	PREDICTED: stromal interaction molecule 2 isoform X3 [Ictalurus punctatus]
XP_018105967.1	606	LOC108710082	eukaryota>metazoa>chordata>vertebrata	Xenopus	laevis	PREDICTED: stromal interaction molecule 1-like isoform X3 [Xenopus laevis]
KXJ22905.1	605	STIM1	eukaryota>metazoa>cnidaria	Exaiptasia	pallida	Stromal interaction molecule 1 [Exaiptasia pallida]
XP_016767778.1	604	LOC411739	eukaryota>metazoa>hexapoda	Apis	mellifera	PREDICTED: stromal interaction molecule homolog isoform X2 [Apis mellifera]
XP_014788351.1	600	LOC106882253	eukaryota>metazoa>mollusca	Octopus	bimaculoides	PREDICTED: stromal interaction molecule 1-like [Octopus bimaculoides]
NP_001162588.1	599	STIM2	eukaryota>metazoa>chordata>vertebrata	Homo	sapiens	stromal interaction molecule 2 isoform 3 precursor [Homo sapiens]
CAG08997.1	598	GSTEN:00030156:G:001	eukaryota>metazoa>chordata>vertebrata>actinopterygii	Tetraodon	nigroviridis	unnamed protein product, partial [Tetraodon nigroviridis]
XP_001900500.1	590	Bm1_45190	eukaryota>metazoa>nematoda	Brugia	malayi	hypothetical protein Bm1_45190 [Brugia malayi]
XP_001850572.1	589	CpipJ_CPIJ009010	eukaryota>metazoa>hexapoda	Culex	quinquefasciatus	conserved hypothetical protein [Culex quinquefasciatus]

[illegible]

EUB63035.1	2470	EGR_02129	eukaryota>metazoa	Echinococcus	granulosus	Voltage-dependent calcium channel type D subunit alpha-1 [Echinococcus granulosus]
XP_016875415.1	2412	CACH2,CACN2,CACNL1A1,CaV1.2,CCHL1A1,LQT8,TS	eukaryota>metazoa>chordata>vertebrata	Homo	sapiens	PREDICTED: voltage-dependent L-type calcium channel subunit alpha-1C isoform X1 [Homo sapiens]
XP_016875416.1	2377	CACH2,CACN2,CACNL1A1,CaV1.2,CCHL1A1,LQT8,TS	eukaryota>metazoa>chordata>vertebrata	Homo	sapiens	PREDICTED: voltage-dependent L-type calcium channel subunit alpha-1C isoform X2 [Homo sapiens]
CAG03249.1	2340	GSTEN:00022583:G:001	eukaryota>metazoa>chordata>vertebrata>actinopterygii	Tetraodon	nigroviridis	unnamed protein product, partial [Tetraodon nigroviridis]
CDS41920.2	2337	EmuJ_000961000	eukaryota>metazoa	Echinococcus	multilocularis	voltage dependent calcium channel subunit [Echinococcus multilocularis]
XP_011419172.1	2314	LOC105322251	eukaryota>metazoa>mollusca	Crassostrea	gigas	PREDICTED: voltage-dependent calcium channel type A subunit alpha-1 isoform X14 [Crassostrea gigas]
XP_016875417.1	2306	CACH2,CACN2,CACNL1A1,CaV1.2,CCHL1A1,LQT8,TS	eukaryota>metazoa>chordata>vertebrata	Homo	sapiens	PREDICTED: voltage-dependent L-type calcium channel subunit alpha-1C isoform X3 [Homo sapiens]
XP_011670127.1	2304	LOC578128	eukaryota>metazoa>echinodermata	Strongylocentrotus	purpuratus	PREDICTED: voltage dependent calcium channel L-type isoform X1 [Strongylocentrotus purpuratus]
XP_011670128.1	2303	LOC578128	eukaryota>metazoa>echinodermata	Strongylocentrotus	purpuratus	PREDICTED: voltage dependent calcium channel L-type isoform X2 [Strongylocentrotus purpuratus]
XP_011662956.1	2301	LOC574743	eukaryota>metazoa>echinodermata	Strongylocentrotus	purpuratus	PREDICTED: voltage-dependent calcium channel type A subunit alpha-1 [Strongylocentrotus purpuratus]
NP_723953.1	2300	alpha1,alpha1D,BG:DS02795.1,Ca-a1D,Ca-alpha1,ca-alpha1d,ca-alpha1D,Ca-alpha[[1]]D,Ca-_1D,Caalpha1D,cad,caID,Ca_alpha1D,CG4894,DmCa1alpha1,Dmca1D,DmCa1D,Dmel\CG4894,DroCa1,l(2)35Fa,l35Fa	eukaryota>metazoa>hexapoda	Drosophila	melanogaster	Ca[2+]-channel protein alpha[[1]] subunit D, isoform D [Drosophila melanogaster]
XP_011670130.1	2300	LOC578128	eukaryota>metazoa>echinodermata	Strongylocentrotus	purpuratus	PREDICTED: voltage dependent calcium channel L-type isoform X3 [Strongylocentrotus purpuratus]
XP_011670131.1	2299	LOC578128	eukaryota>metazoa>echinodermata	Strongylocentrotus	purpuratus	PREDICTED: voltage dependent calcium channel L-type isoform X4 [Strongylocentrotus purpuratus]
XP_011670132.1	2298	LOC578128	eukaryota>metazoa>echinodermata	Strongylocentrotus	purpuratus	PREDICTED: voltage dependent calcium channel L-type isoform X5 [Strongylocentrotus purpuratus]
XP_011670133.1	2293	LOC578128	eukaryota>metazoa>echinodermata	Strongylocentrotus	purpuratus	PREDICTED: voltage dependent calcium channel L-type isoform X6 [Strongylocentrotus purpuratus]
XP_011670134.1	2289	LOC578128	eukaryota>metazoa>echinodermata	Strongylocentrotus	purpuratus	PREDICTED: voltage dependent calcium channel L-type isoform X7 [Strongylocentrotus purpuratus]
XP_016875418.1	2287	CACH2,CACN2,CACNL1A1,CaV1.2,CCHL1A1,LQT8,TS	eukaryota>metazoa>chordata>vertebrata	Homo	sapiens	PREDICTED: voltage-dependent L-type calcium channel subunit alpha-1C isoform X4 [Homo sapiens]
XP_016875419.1	2287	CACH2,CACN2,CACNL1A1,CaV1.2,CCHL1A1,LQT8,TS	eukaryota>metazoa>chordata>vertebrata	Homo	sapiens	PREDICTED: voltage-dependent L-type calcium channel subunit alpha-1C isoform X5 [Homo sapiens]
XP_011419175.1	2282	LOC105322251	eukaryota>metazoa>mollusca	Crassostrea	gigas	PREDICTED: voltage-dependent calcium channel type A subunit alpha-1 isoform X15 [Crassostrea gigas]
XP_012365582.1	2264	CACNA1D	eukaryota>metazoa>chordata>vertebrata	Nomascus	leucogenys	PREDICTED: LOW QUALITY PROTEIN: voltage-dependent L-type calcium channel subunit alpha-1D [Nomascus leucogenys]
XP_016875420.1	2259	CACH2,CACN2,CACNL1A1,CaV1.2,CCHL1A1,LQT8,TS	eukaryota>metazoa>chordata>vertebrata	Homo	sapiens	PREDICTED: voltage-dependent L-type calcium channel subunit alpha-1C isoform X6 [Homo sapiens]
XP_016875421.1	2254	CACH2,CACN2,CACNL1A1,CaV1.2,CCHL1A1,LQT8,TS	eukaryota>metazoa>chordata>vertebrata	Homo	sapiens	PREDICTED: voltage-dependent L-type calcium channel subunit alpha-1C isoform X7 [Homo sapiens]
XP_011532396.1	2246	CACH3,CACN4,CACNL1A2,Cav1.3,CCHL1A2,PASNA,SANDD	eukaryota>metazoa>chordata>vertebrata	Homo	sapiens	PREDICTED: voltage-dependent L-type calcium channel subunit alpha-1D isoform X2 [Homo sapiens]
XP_016862626.1	2246	CACH3,CACN4,CACNL1A2,Cav1.3,CCHL1A2,PASNA,SANDD	eukaryota>metazoa>chordata>vertebrata	Homo	sapiens	PREDICTED: voltage-dependent L-type calcium channel subunit alpha-1D isoform X1 [Homo sapiens]
XP_016862627.1	2245	CACH3,CACN4,CACNL1A2,Cav1.3,CCHL1A2,PASNA,SANDD	eukaryota>metazoa>chordata>vertebrata	Homo	sapiens	PREDICTED: voltage-dependent L-type calcium channel subunit alpha-1D isoform X3 [Homo sapiens]
XP_015148461.1	2238	CACNA1D	eukaryota>metazoa>chordata>vertebrata	Gallus	gallus	PREDICTED: voltage-dependent L-type calcium channel subunit alpha-1D isoform X1 [Gallus gallus]
XP_015148462.1	2238	CACNA1D	eukaryota>metazoa>chordata>vertebrata	Gallus	gallus	PREDICTED: voltage-dependent L-type calcium channel subunit alpha-1D isoform X2 [Gallus gallus]
XP_016862628.1	2237	CACH3,CACN4,CACNL1A2,Cav1.3,CCHL1A2,PASNA,SANDD	eukaryota>metazoa>chordata>vertebrata	Homo	sapiens	PREDICTED: voltage-dependent L-type calcium channel subunit alpha-1D isoform X4 [Homo sapiens]
XP_011419176.1	2226	LOC105322251	eukaryota>metazoa>mollusca	Crassostrea	gigas	PREDICTED: voltage-dependent calcium channel type A subunit alpha-1 isoform X16 [Crassostrea gigas]
XP_016862629.1	2226	CACH3,CACN4,CACNL1A2,Cav1.3,CCHL1A2,PASNA,SANDD	eukaryota>metazoa>chordata>vertebrata	Homo	sapiens	PREDICTED: voltage-dependent L-type calcium channel subunit alpha-1D isoform X5 [Homo sapiens]
XP_016862630.1	2226	CACH3,CACN4,CACNL1A2,Cav1.3,CCHL1A2,PASNA,SANDD	eukaryota>metazoa>chordata>vertebrata	Homo	sapiens	PREDICTED: voltage-dependent L-type calcium channel subunit alpha-1D isoform X6 [Homo sapiens]
XP_011239477.1	2225	Cav1.2,Cchl1a1,D930026N18Rik,MBC,MELC-CC	eukaryota>metazoa>chordata>vertebrata	Mus	musculus	PREDICTED: voltage-dependent L-type calcium channel subunit alpha-1C isoform X1 [Mus musculus]
XP_011239478.1	2225	Cav1.2,Cchl1a1,D930026N18Rik,MBC,MELC-CC	eukaryota>metazoa>chordata>vertebrata	Mus	musculus	PREDICTED: voltage-dependent L-type calcium channel subunit alpha-1C isoform X3 [Mus musculus]
XP_017176845.1	2225	Cav1.2,Cchl1a1,D930026N18Rik,MBC,MELC-CC	eukaryota>metazoa>chordata>vertebrata	Mus	musculus	PREDICTED: voltage-dependent L-type calcium channel subunit alpha-1C isoform X2 [Mus musculus]
XP_017176846.1	2223	Cav1.2,Cchl1a1,D930026N18Rik,MBC,MELC-CC	eukaryota>metazoa>chordata>vertebrata	Mus	musculus	PREDICTED: voltage-dependent L-type calcium channel subunit alpha-1C isoform X4 [Mus musculus]
NP_001242931.1	2222	Cav1.2,Cchl1a1,D930026N18Rik,MBC,MELC-CC	eukaryota>metazoa>chordata>vertebrata	Mus	musculus	voltage-dependent L-type calcium channel subunit alpha-1C isoform 10 [Mus musculus]
XP_006505478.1	2222	Cav1.2,Cchl1a1,D930026N18Rik,MBC,MELC-CC	eukaryota>metazoa>chordata>vertebrata	Mus	musculus	PREDICTED: voltage-dependent L-type calcium channel subunit alpha-1C isoform X6 [Mus musculus]
XP_016875422.1	2222	CACH2,CACN2,CACNL1A1,CaV1.2,CCHL1A1,LQT8,TS	eukaryota>metazoa>chordata>vertebrata	Homo	sapiens	PREDICTED: voltage-dependent L-type calcium channel subunit alpha-1C isoform X8 [Homo sapiens]
XP_017176847.1	2222	Cav1.2,Cchl1a1,D930026N18Rik,MBC,MELC-CC	eukaryota>metazoa>chordata>vertebrata	Mus	musculus	PREDICTED: voltage-dependent L-type calcium channel subunit alpha-1C isoform X5 [Mus musculus]
NP_955630.3	2221	CACH2,CACN2,CACNL1A1,CaV1.2,CCHL1A1,LQT8,TS	eukaryota>metazoa>chordata>vertebrata	Homo	sapiens	voltage-dependent L-type calcium channel subunit alpha-1C isoform 1 [Homo sapiens]
XP_019697108.1	2221	LOC105183370	eukaryota>metazoa>hexapoda	Harpegnathos	saltator	PREDICTED: muscle calcium channel subunit alpha-1 isoform X6 [Harpegnathos saltator]
XP_015142138.1	2220	CACNA1C	eukaryota>metazoa>chordata>vertebrata	Gallus	gallus	PREDICTED: voltage-dependent L-type calcium channel subunit alpha-1C isoform X31 [Gallus gallus]
XP_016875423.1	2219	CACH2,CACN2,CACNL1A1,CaV1.2,CCHL1A1,LQT8,TS	eukaryota>metazoa>chordata>vertebrata	Homo	sapiens	PREDICTED: voltage-dependent L-type calcium channel subunit alpha-1C isoform X9 [Homo sapiens]
XP_016875424.1	2219	CACH2,CACN2,CACNL1A1,CaV1.2,CCHL1A1,LQT8,TS	eukaryota>metazoa>chordata>vertebrata	Homo	sapiens	PREDICTED: voltage-dependent L-type calcium channel subunit alpha-1C isoform X10 [Homo sapiens]
XP_016875444.1	2219	CACH2,CACN2,CACNL1A1,CaV1.2,CCHL1A1,LQT8,TS	eukaryota>metazoa>chordata>vertebrata	Homo	sapiens	PREDICTED: voltage-dependent L-type calcium channel subunit alpha-1C isoform X32 [Homo sapiens]
[illegible]						

[illegible]

XP_003971275.1	1016	LOC101070073	eukaryota>metazoa>chordata>vertebrata>actinopterygii	Takifugu	rubripes	PREDICTED: short transient receptor potential channel 2-like [Takifugu rubripes]
XP_013384065.1	1014	LOC106154271	eukaryota>metazoa	Lingula	anatina	PREDICTED: short transient receptor potential channel 7-like [Lingula anatina]
XP_018669082.1	1013	LOC100184754	eukaryota>metazoa>chordata	Ciona	intestinalis	PREDICTED: short transient receptor potential channel 4-like isoform X1 [Ciona intestinalis]
XP_018669083.1	1010	LOC100184754	eukaryota>metazoa>chordata	Ciona	intestinalis	PREDICTED: short transient receptor potential channel 4-like isoform X2 [Ciona intestinalis]
ERG81205.1	1007	ASU_10569	eukaryota>metazoa>nematoda	Ascaris	suum	transient receptor potential-gamma protein [Ascaris suum]
CDS41436.1	989	EmuJ_000908800	eukaryota>metazoa	Echinococcus	multilocularis	transient receptor potential gamma protein [Echinococcus multilocularis]
XP_420310.2	989	TRPC5	eukaryota>metazoa>chordata>vertebrata	Gallus	gallus	PREDICTED: short transient receptor potential channel 5 [Gallus gallus]
NP_003297.1	982	HTRP-4,HTRP4,TRP4	eukaryota>metazoa>chordata>vertebrata	Homo	sapiens	short transient receptor potential channel 4 isoform epsilon [Homo sapiens]
XP_012357404.1	982	TRPC4	eukaryota>metazoa>chordata>vertebrata	Nomascus	leucogenys	PREDICTED: short transient receptor potential channel 4 isoform X1 [Nomascus leucogenys]
XP_018085351.1	982	LOC108698404	eukaryota>metazoa>chordata>vertebrata	Xenopus	laevis	PREDICTED: short transient receptor potential channel 5-like [Xenopus laevis]
XP_018088468.1	982	LOC108700107	eukaryota>metazoa>chordata>vertebrata	Xenopus	laevis	PREDICTED: short transient receptor potential channel 5-like [Xenopus laevis]
EUB64483.1	980	EGR_00433	eukaryota>metazoa	Echinococcus	granulosus	Transient receptor potential-gamma protein [Echinococcus granulosus]
NP_057263.1	977	HTRP-4,HTRP4,TRP4	eukaryota>metazoa>chordata>vertebrata	Homo	sapiens	short transient receptor potential channel 4 isoform alpha [Homo sapiens]
XP_003270343.1	977	TRPC4	eukaryota>metazoa>chordata>vertebrata	Nomascus	leucogenys	PREDICTED: short transient receptor potential channel 4 isoform X2 [Nomascus leucogenys]
XP_005585748.1	977	TRPC4	eukaryota>metazoa>chordata>vertebrata	Macaca	fascicularis	PREDICTED: short transient receptor potential channel 4 [Macaca fascicularis]
NP_033454.1	975	CCE2,TRP-5,TRP5,Trrp5	eukaryota>metazoa>chordata>vertebrata	Mus	musculus	short transient receptor potential channel 5 [Mus musculus]
NP_058680.1	974	CCE1,STRPC4,Trp4,Trrp4	eukaryota>metazoa>chordata>vertebrata	Mus	musculus	short transient receptor potential channel 4 isoform 1 [Mus musculus]
XP_003262260.1	974	TRPC5	eukaryota>metazoa>chordata>vertebrata	Nomascus	leucogenys	PREDICTED: short transient receptor potential channel 5 [Nomascus leucogenys]
XP_005594455.1	974	TRPC5	eukaryota>metazoa>chordata>vertebrata	Macaca	fascicularis	PREDICTED: short transient receptor potential channel 5 [Macaca fascicularis]
XP_006501359.1	974	CCE1,STRPC4,Trp4,Trrp4	eukaryota>metazoa>chordata>vertebrata	Mus	musculus	PREDICTED: short transient receptor potential channel 4 isoform X1 [Mus musculus]
XP_417089.2	974	TRPC4	eukaryota>metazoa>chordata>vertebrata	Gallus	gallus	PREDICTED: short transient receptor potential channel 4 [Gallus gallus]
NP_036603.1	973	PPP1R159,TRP5	eukaryota>metazoa>chordata>vertebrata	Homo	sapiens	short transient receptor potential channel 5 [Homo sapiens]
XP_016885263.1	973	PPP1R159,TRP5	eukaryota>metazoa>chordata>vertebrata	Homo	sapiens	PREDICTED: short transient receptor potential channel 5 isoform X1 [Homo sapiens]
XP_011666677.1	970	LOC586844	eukaryota>metazoa>echinodermata	Strongylocentrotus	purpuratus	PREDICTED: short transient receptor potential channel 3 isoform X1 [Strongylocentrotus purpuratus]
XP_011666683.1	970	LOC586844	eukaryota>metazoa>echinodermata	Strongylocentrotus	purpuratus	PREDICTED: short transient receptor potential channel 3 isoform X1 [Strongylocentrotus purpuratus]
XP_009009132.1	967	HELRODRAFT_63393	eukaryota>metazoa>annelida	Helobdella	robusta	hypothetical protein HELRODRAFT_63393 [Helobdella robusta]
XP_018103124.1	966	LOC108708683	eukaryota>metazoa>chordata>vertebrata	Xenopus	laevis	PREDICTED: short transient receptor potential channel 4-like [Xenopus laevis]
XP_003376781.1	957	Tsp_00950	eukaryota>metazoa>nematoda	Trichinella	spiralis	transient receptor potential-gamma protein [Trichinella spiralis]
XP_791700.3	956	LOC586844	eukaryota>metazoa>echinodermata	Strongylocentrotus	purpuratus	PREDICTED: short transient receptor potential channel 3 isoform X2 [Strongylocentrotus purpuratus]
XP_013386796.1	954	LOC106156216	eukaryota>metazoa	Lingula	anatina	PREDICTED: transient-receptor-potential-like protein [Lingula anatina]
XP_002733765.1	953	LOC100378311	eukaryota>metazoa>hemichordata	Saccoglossus	kowalevskii	PREDICTED: transient-receptor-potential-like protein-like [Saccoglossus kowalevskii]
XP_004938897.2	952	TRPC6	eukaryota>metazoa>chordata>vertebrata	Gallus	gallus	PREDICTED: short transient receptor potential channel 6 isoform X1 [Gallus gallus]
XP_006509912.1	949	AV025995,LLHWJM002,LLHWJM003,LLHWJM004,mtrp6,TRP-6,Trrp6	eukaryota>metazoa>chordata>vertebrata	Mus	musculus	PREDICTED: short transient receptor potential channel 6 isoform X3 [Mus musculus]
XP_013398015.1	942	LOC106164593	eukaryota>metazoa	Lingula	anatina	PREDICTED: short transient receptor potential channel 3-like [Lingula anatina]
OAF71995.1	941	A3Q56_00219	eukaryota>metazoa	Intoshia	linei	Transient receptor protein 2 [Intoshia linei]
XP_017343908.1	941	trpc4	eukaryota>metazoa>chordata>vertebrata>actinopterygii	Ictalurus	punctatus	PREDICTED: short transient receptor potential channel 4 [Ictalurus punctatus]
XP_018124202.1	941	LOC108719617	eukaryota>metazoa>chordata>vertebrata	Xenopus	laevis	PREDICTED: short transient receptor potential channel 3-like [Xenopus laevis]
XP_011530519.1	937	SCA41,TRP3	eukaryota>metazoa>chordata>vertebrata	Homo	sapiens	PREDICTED: short transient receptor potential channel 3 isoform X1 [Homo sapiens]
XP_015306276.1	937	TRPC3	eukaryota>metazoa>chordata>vertebrata	Macaca	fascicularis	PREDICTED: short transient receptor potential channel 3 isoform X1 [Macaca fascicularis]
XP_011667283.1	936	LOC580692	eukaryota>metazoa>echinodermata	Strongylocentrotus	purpuratus	PREDICTED: short transient receptor potential channel 3 [Strongylocentrotus purpuratus]
XP_016864067.1	936	SCA41,TRP3	eukaryota>metazoa>chordata>vertebrata	Homo	sapiens	PREDICTED: short transient receptor potential channel 3 isoform X2 [Homo sapiens]
XP_785826.2	936	LOC580692	eukaryota>metazoa>echinodermata	Strongylocentrotus	purpuratus	PREDICTED: short transient receptor potential channel 3 [Strongylocentrotus purpuratus]
NP_001276811.1	934	si:dkey-109i21.3,trpc4b	eukaryota>metazoa>chordata>vertebrata>actinopterygii	Danio	rerio	short transient receptor potential channel 4 [Danio rerio]
XP_683649.2	934	si:dkey-109i21.3,trpc4b	eukaryota>metazoa>chordata>vertebrata>actinopterygii	Danio	rerio	short transient receptor potential channel 4 isoform X1 [Danio rerio]
NP_001244208.1	933	TRPC3	eukaryota>metazoa>chordata>vertebrata	Gallus	gallus	short transient receptor potential channel 3 [Gallus gallus]
NP_004612.2	931	FSGS2,TRP6	eukaryota>metazoa>chordata>vertebrata	Homo	sapiens	short transient receptor potential channel 6 [Homo sapiens]
XP_005579492.1	931	TRPC6	eukaryota>metazoa>chordata>vertebrata	Macaca	fascicularis	PREDICTED: short transient receptor potential channel 6 [Macaca fascicularis]
NP_038866.2	930	AV025995,LLHWJM002,LLHWJM003,LLHWJM004,mtrp6,TRP-6,Trrp6	eukaryota>metazoa>chordata>vertebrata	Mus	musculus	short transient receptor potential channel 6 isoform 1 [Mus musculus]
XP_006509913.1	930	AV025995,LLHWJM002,LLHWJM003,LLHWJM004,mtrp6,TRP-6,Trrp6	eukaryota>metazoa>chordata>vertebrata	Mus	musculus	PREDICTED: short transient receptor potential channel 6 isoform X1 [Mus musculus]
XP_003253082.1	928	TRPC6	eukaryota>metazoa>chordata>vertebrata	Nomascus	leucogenys	PREDICTED: LOW QUALITY PROTEIN: short transient receptor potential channel 6 [Nomascus leucogenys]
XP_011455889.1	926	LOC105348246	eukaryota>metazoa>mollusca	Crassostrea	gigas	PREDICTED: short transient receptor potential channel 3 [Crassostrea gigas]
XP_011420089.1	924	LOC105322886	eukaryota>metazoa>mollusca	Crassostrea	gigas	PREDICTED: short transient receptor potential channel 3 [Crassostrea gigas]
XP_011428523.1	924	LOC105329068	eukaryota>metazoa>mollusca	Crassostrea	gigas	PREDICTED: short transient receptor potential channel 3 isoform X1 [Crassostrea gigas]
XP_002607434.1	923	BRAFLDRAFT_69859	eukaryota>metazoa>chordata	Branchiostoma	floridae	hypothetical protein BRAFLDRAFT_69859 [Branchiostoma floridae]
XP_011428524.1	923	LOC105329068	eukaryota>metazoa>mollusca	Crassostrea	gigas	PREDICTED: short transient receptor potential channel 3 isoform X2 [Crassostrea gigas]
XP_013780206.1	923	LOC106464597	eukaryota>metazoa	Limulus	polyphemus	PREDICTED: transient-receptor-potential-like protein isoform X1 [Limulus polyphemus]
XP_013780207.1	923	LOC106464597	eukaryota>metazoa	Limulus	polyphemus	PREDICTED: transient-receptor-potential-like protein isoform X2 [Limulus polyphemus]
NP_001124170.1	921	SCA41,TRP3	eukaryota>metazoa>chordata>vertebrata	Homo	sapiens	short transient receptor potential channel 3 isoform a [Homo sapiens]
XP_003271391.1	921	TRPC3	eukaryota>metazoa>chordata>vertebrata	Nomascus	leucogenys	PREDICTED: short transient receptor potential channel 3 isoform X1 [Nomascus leucogenys]
XP_005555901.1	921	TRPC3	eukaryota>metazoa>chordata>vertebrata	Macaca	fascicularis	PREDICTED: short transient receptor potential channel 3 isoform X2 [Macaca fascicularis]
XP_011530520.1	920	SCA41,TRP3	eukaryota>metazoa>chordata>vertebrata	Homo	sapiens	PREDICTED: short transient receptor potential channel 3 isoform X3 [Homo sapiens]
XP_003971385.1	918	trpc4	eukaryota>metazoa>chordata>vertebrata>actinopterygii	Takifugu	rubripes	PREDICTED: short transient receptor potential channel 4 [Takifugu rubripes]
XP_013775532.1	917	LOC106460388	eukaryota>metazoa	Limulus	polyphemus	PREDICTED: transient-receptor-potential-like protein [Limulus polyphemus]
XP_003968682.1	916	LOC101062185	eukaryota>metazoa>chordata>vertebrata>actinopterygii	Takifugu	rubripes	PREDICTED: short transient receptor potential channel 4-like [Takifugu rubripes]
[illegible]	[illegible]	[illegible]	[illegible]	[illegible]	[illegible]	[illegible]

EUB65028.1	313	EGR_00297	eukaryota>metazoa	Echinococcus	granulosus	Calumenin-B [Echinococcus granulosus]
NP_001085102.1	313	MGC78878	eukaryota>metazoa>chordata>vertebrata	Xenopus	laevis	uncharacterized protein LOC432173 precursor [Xenopus laevis]
NP_001087007.1	313	rcn2,rcn2.L	eukaryota>metazoa>chordata>vertebrata	Xenopus	laevis	reticulocalbin 2 S homeolog precursor [Xenopus laevis]
NP_001187699.1	313	calu	eukaryota>metazoa>chordata>vertebrata>actinopterygii	Ictalurus	punctatus	reticulocalbin-3 precursor [Ictalurus punctatus]
XP_009043912.1	313	LOTGIDRAFT_103075	eukaryota>metazoa>mollusca	Lottia	gigantea	hypothetical protein LOTGIDRAFT_103075 [Lottia gigantea]
XP_017338425.1	313	rcn3	eukaryota>metazoa>chordata>vertebrata>actinopterygii	Ictalurus	punctatus	PREDICTED: reticulocalbin-3 isoform X1 [Ictalurus punctatus]
ERG83583.1	312	ASU_06763	eukaryota>metazoa>nematoda	Ascaris	suum	calumenin-a [Ascaris suum]
XP_002129310.1	312	LOC100182984	eukaryota>metazoa>chordata	Ciona	intestinalis	PREDICTED: reticulocalbin-2-like [Ciona intestinalis]
XP_002167284.1	312	LOC100206632	eukaryota>metazoa>cnidaria	Hydra	vulgaris	PREDICTED: calumenin-B-like [Hydra vulgaris]
OQV20397.1	312	BV898_05683	eukaryota>metazoa	Hypsibius	dujardini	putative Calumenin [Hypsibius dujardini]
XP_003378780.1	312	Tsp_12034	eukaryota>metazoa>nematoda	Trichinella	spiralis	putative calumenin [Trichinella spiralis]
XP_002123414.1	311	LOC100184283	eukaryota>metazoa>chordata	Ciona	intestinalis	PREDICTED: calumenin-B-like [Ciona intestinalis]
XP_011605652.1	310	rcn2	eukaryota>metazoa>chordata>vertebrata>actinopterygii	Takifugu	rubripes	PREDICTED: reticulocalbin-2 isoform X2 [Takifugu rubripes]
CBY10162.1	309	GSOID_T00010993001	eukaryota>metazoa>chordata	Oikopleura	dioica	unnamed protein product [Oikopleura dioica]
XP_008184569.1	309	LOC100159618	eukaryota>metazoa>hexapoda	Acyrthosiphon	pisum	PREDICTED: calumenin-A [Acyrthosiphon pisum]
XP_009031860.1	309	HELRODRAFT_186226	eukaryota>metazoa>annelida	Helobdella	robusta	hypothetical protein HELRODRAFT_186226 [Helobdella robusta]
KXJ19679.1	308	calua	eukaryota>metazoa>cnidaria	Exaiptasia	pallida	Calumenin-A [Exaiptasia pallida]
KXJ19691.1	308	calub	eukaryota>metazoa>cnidaria	Exaiptasia	pallida	Calumenin-B [Exaiptasia pallida]
XP_001627907.1	308	NEMVEDRAFT_v1g190767	eukaryota>metazoa>cnidaria	Nematostella	vectensis	predicted protein [Nematostella vectensis]
Smar1000001370	308	Smar1000001370	eukaryota>metazoa>arthropoda>myriapoda	Strigamia	maritima	>Smar1000001370
NP_001027627.1	307	calumenin	eukaryota>metazoa>chordata	Ciona	intestinalis	calumenin homologue precursor [Ciona intestinalis]
NP_001280137.1	307	RCN2	eukaryota>metazoa>chordata>vertebrata	Gallus	gallus	reticulocalbin-2 isoform 1 precursor [Gallus gallus]
XP_002122894.2	307	LOC100176707	eukaryota>metazoa>chordata	Ciona	intestinalis	PREDICTED: reticulocalbin-2-like [Ciona intestinalis]
XP_009029673.1	307	HELRODRAFT_89801	eukaryota>metazoa>annelida	Helobdella	robusta	hypothetical protein HELRODRAFT_89801 [Helobdella robusta]
CAF94652.1	306	GSTEN:00010846:G:001	eukaryota>metazoa>chordata>vertebrata>actinopterygii	Tetraodon	nigroviridis	unnamed protein product [Tetraodon nigroviridis]
XP_002590942.1	304	BRAFLDRAFT_101089	eukaryota>metazoa>chordata	Branchiostoma	floridae	hypothetical protein BRAFLDRAFT_101089 [Branchiostoma floridae]
NP_001280159.1	303	RCN2	eukaryota>metazoa>chordata>vertebrata	Gallus	gallus	reticulocalbin-2 isoform 2 precursor [Gallus gallus]
XP_001633892.1	303	NEMVEDRAFT_v1g102868	eukaryota>metazoa>cnidaria	Nematostella	vectensis	predicted protein [Nematostella vectensis]
XP_016440728.1	303	LOC107766464	eukaryota>viridiplantae	Nicotiana	tabacum	PREDICTED: calumenin-A-like, partial [Nicotiana tabacum]
XP_002162407.1	302	LOC100213173	eukaryota>metazoa>cnidaria	Hydra	vulgaris	PREDICTED: calumenin-A-like [Hydra vulgaris]
XP_002109885.1	301	TRIADDRAFT_20674	eukaryota>metazoa>placozoa	Trichoplax	adhaerens	hypothetical protein TRIADDRAFT_20674 [Trichoplax adhaerens]
XP_003376324.1	299	Tsp_00516	eukaryota>metazoa>nematoda	Trichinella	spiralis	calumenin [Trichinella spiralis]
XP_015758332.1	299	LOC107337629	eukaryota>metazoa>cnidaria	Acropora	digitifera	PREDICTED: calumenin-like isoform X3 [Acropora digitifera]
XP_001639736.1	297	NEMVEDRAFT_v1g86027	eukaryota>metazoa>cnidaria	Nematostella	vectensis	predicted protein, partial [Nematostella vectensis]
XP_002118306.1	297	TRIADDRAFT_33976	eukaryota>metazoa>placozoa	Trichoplax	adhaerens	hypothetical protein TRIADDRAFT_33976 [Trichoplax adhaerens]
XP_015760930.1	297	LOC107340092	eukaryota>metazoa>cnidaria	Acropora	digitifera	PREDICTED: calumenin-like [Acropora digitifera]
KXJ10711.1	294	calua	eukaryota>metazoa>cnidaria	Exaiptasia	pallida	Calumenin-A [Exaiptasia pallida]
Smed1000002375	294	Smed1000002375	eukaryota>metazoa>platyhelminthe	Schmidtea	mediterranea	>Smed1000002375
XP_002155513.3	293	LOC100198810	eukaryota>metazoa>cnidaria	Hydra	vulgaris	PREDICTED: calumenin-B-like [Hydra vulgaris]
XP_018648564.1	293	Smp_005230.1	eukaryota>metazoa	Schistosoma	mansoni	putative ef hand containing protein [Schistosoma mansoni]
CBY20674.1	291	GSOID_T00000677001	eukaryota>metazoa>chordata	Oikopleura	dioica	unnamed protein product [Oikopleura dioica]
CDI98589.1	288	EmuJ_000245300	eukaryota>metazoa	Echinococcus	multilocularis	calumenin [Echinococcus multilocularis]
CDS41602.1	286	EmuJ_000926900	eukaryota>metazoa	Echinococcus	multilocularis	reticulocalbin [Echinococcus multilocularis]
NP_491936.3	286	calu-2	eukaryota>metazoa>nematoda	Caenorhabditis	elegans	CALUmenin (calcium-binding protein) homolog [Caenorhabditis elegans]
XP_005589982.1	281	RCN3	eukaryota>metazoa>chordata>vertebrata	Macaca	fascicularis	PREDICTED: reticulocalbin-3 [Macaca fascicularis]
XP_018654312.1	281	Smp_103740.1	eukaryota>metazoa	Schistosoma	mansoni	putative ef hand containing protein [Schistosoma mansoni]
Smed1000012997	281	Smed1000012997	eukaryota>metazoa>platyhelminthe	Schmidtea	mediterranea	>Smed1000012997
XP_012360521.1	280	RCN1	eukaryota>metazoa>chordata>vertebrata	Nomascus	leucogenys	PREDICTED: reticulocalbin-1 isoform X1 [Nomascus leucogenys]
Caps1000012157	272	Caps1000012157	eukaryota>metazoa>annelida	Capitella	spI	>Caps1000012157
XP_001623269.1	269	NEMVEDRAFT_v1g138173	eukaryota>metazoa>cnidaria	Nematostella	vectensis	predicted protein, partial [Nematostella vectensis]
XP_001634852.1	267	NEMVEDRAFT_v1g184260	eukaryota>metazoa>cnidaria	Nematostella	vectensis	predicted protein [Nematostella vectensis]
XP_003279772.2	266	SDF4	eukaryota>metazoa>chordata>vertebrata	Nomascus	leucogenys	PREDICTED: 45 kDa calcium-binding protein [Nomascus leucogenys]
XP_001626862.1	264	NEMVEDRAFT_v1g235717	eukaryota>metazoa>cnidaria	Nematostella	vectensis	predicted protein [Nematostella vectensis]
XP_009016089.1	261	HELRODRAFT_64364	eukaryota>metazoa>annelida	Helobdella	robusta	hypothetical protein HELRODRAFT_64364 [Helobdella robusta]
XP_002410091.1	251	IscW_ISCW020366	eukaryota>metazoa	Ixodes	scapularis	reticulocalbin, putative [Ixodes scapularis]
XP_001641982.1	250	NEMVEDRAFT_v1g79348	eukaryota>metazoa>cnidaria	Nematostella	vectensis	predicted protein, partial [Nematostella vectensis]
CBY14654.1	250	GSOID_T00009701001	eukaryota>metazoa>chordata	Oikopleura	dioica	unnamed protein product [Oikopleura dioica]
XP_013773859.1	244	LOC106458850	eukaryota>metazoa	Limulus	polyphemus	PREDICTED: reticulocalbin-2-like isoform X2 [Limulus polyphemus]
XP_015129037.1	242	LOC107049736	eukaryota>metazoa>chordata>vertebrata	Gallus	gallus	PREDICTED: calumenin-like [Gallus gallus]
XP_005159358.1	238	si:dkey-24111.6,zgc:110594	eukaryota>metazoa>chordata>vertebrata>actinopterygii	Danio	rerio	PREDICTED: reticulocalbin-2 isoform X1 [Danio rerio]
XP_009171630.1	238	T265_07761	eukaryota>metazoa	Opisthorchis	viverrini	hypothetical protein T265_07761 [Opisthorchis viverrini]
XP_015767447.1	237	LOC107346183	eukaryota>metazoa>cnidaria	Acropora	digitifera	PREDICTED: calumenin-B-like [Acropora digitifera]
EUB59098.1	235	EGR_06090	eukaryota>metazoa	Echinococcus	granulosus	Calumenin-A [Echinococcus granulosus]
ERG81497.1	233	ASU_10105	eukaryota>metazoa>nematoda	Ascaris	suum	calumenin-a [Ascaris suum]
XP_018648565.1	228	Smp_005230.1	eukaryota>metazoa	Schistosoma	mansoni	EF hand containing protein [Schistosoma mansoni]
NP_001186602.1	224	CALU	eukaryota>metazoa>chordata>vertebrata	Homo	sapiens	calumenin isoform e precursor [Homo sapiens]
XP_003261347.1	224	CALU	eukaryota>metazoa>chordata>vertebrata	Nomascus	leucogenys	PREDICTED: calumenin isoform X5 [Nomascus leucogenys]
XP_001620654.1	208	NEMVEDRAFT_v1g147476	eukaryota>metazoa>cnidaria	Nematostella	vectensis	hypothetical protein NEMVEDRAFT_v1g147476 [Nematostella vectensis]
XP_011680474.1	196	LOC589149	eukaryota>metazoa>echinodermata	Strongylocentrotus	purpuratus	PREDICTED: calumenin-B-like [Strongylocentrotus purpuratus]
NP_477393.1	192	anon-EST:Posey14,BEST:CK02682,CG9148,CK02682,DCB-45,Dmel\CG9148,SCF	eukaryota>metazoa>hexapoda	Drosophila	melanogaster	supercoiling factor, isoform B [Drosophila melanogaster]
XP_013415619.1	174	LOC106177407	eukaryota>metazoa	Lingula	anatina	PREDICTED: calumenin-B-like [Lingula anatina]
XP_003254421.1	165	RCN1	eukaryota>metazoa>chordata>vertebrata	Nomascus	leucogenys	PREDICTED: reticulocalbin-1 isoform X2 [Nomascus leucogenys]
XP_005259146.1	165	RLP49	eukaryota>metazoa>chordata>vertebrata	Homo	sapiens	PREDICTED: reticulocalbin-3 isoform X2 [Homo sapiens]
NP_001272341.1	164	9530075H20Rik	eukaryota>metazoa>chordata>vertebrata	Mus	musculus	calumenin isoform 3 [Mus musculus]
XP_016868148.1	164	CALU	eukaryota>metazoa>chordata>vertebrata	Homo	sapiens	PREDICTED: calumenin isoform X1 [Homo sapiens]

AB. Bcl2

[illegible]

XP_011438715.1	1663	LOC105336190	eukaryota>metazoa>mollusca	Crassostrea	gigas	PREDICTED: neurabin-1 isoform X5 [Crassostrea gigas]
XP_011438716.1	1662	LOC105336190	eukaryota>metazoa>mollusca	Crassostrea	gigas	PREDICTED: neurabin-1 isoform X6 [Crassostrea gigas]
XP_013422095.1	1656	LOC106182023	eukaryota>metazoa	Lingula	anatina	PREDICTED: uncharacterized protein LOC106182023 isoform X10 [Lingula anatina]
XP_011438717.1	1650	LOC105336190	eukaryota>metazoa>mollusca	Crassostrea	gigas	PREDICTED: neurabin-1 isoform X7 [Crassostrea gigas]
XP_013422096.1	1626	LOC106182023	eukaryota>metazoa	Lingula	anatina	PREDICTED: uncharacterized protein LOC106182023 isoform X11 [Lingula anatina]
XP_011667300.1	1613	LOC577610	eukaryota>metazoa>echinodermata	Strongylocentrotus	purpuratus	PREDICTED: uncharacterized protein LOC577610 isoform X1 [Strongylocentrotus purp[illegible]
XP_011667301.1	1603	LOC577610	eukaryota>metazoa>echinodermata	Strongylocentrotus	purpuratus	PREDICTED: uncharacterized protein LOC577610 isoform X2 [Strongylocentrotus purp[illegible]
XP_011667302.1	1600	LOC577610	eukaryota>metazoa>echinodermata	Strongylocentrotus	purpuratus	PREDICTED: uncharacterized protein LOC577610 isoform X3 [Strongylocentrotus purp[illegible]
XP_011667303.1	1598	LOC577610	eukaryota>metazoa>echinodermata	Strongylocentrotus	purpuratus	PREDICTED: uncharacterized protein LOC577610 isoform X4 [Strongylocentrotus purp[illegible]
XP_011667304.1	1575	LOC577610	eukaryota>metazoa>echinodermata	Strongylocentrotus	purpuratus	PREDICTED: uncharacterized protein LOC577610 isoform X5 [Strongylocentrotus purp[illegible]
XP_011667305.1	1567	LOC577610	eukaryota>metazoa>echinodermata	Strongylocentrotus	purpuratus	PREDICTED: neurabin-1 isoform X6 [Strongylocentrotus purpuratus]
XP_004210881.2	1560	LOC101236396	eukaryota>metazoa>cnidaria	Hydra	vulgaris	PREDICTED: merozoite surface protein 1-like [Hydra vulgaris]
XP_017211649.1	1552	si:ch211-269i23.2	eukaryota>metazoa>chordata>vertebrata>actinopterygii	Danio	rerio	PREDICTED: neurabin-1 isoform X1 [Danio rerio]
XP_011667306.1	1543	LOC577610	eukaryota>metazoa>echinodermata	Strongylocentrotus	purpuratus	PREDICTED: uncharacterized protein LOC577610 isoform X7 [Strongylocentrotus purp[illegible]
XP_002122996.1	1539	LOC100179135	eukaryota>metazoa>chordata	Ciona	intestinalis	PREDICTED: neurabin-1 isoform X1 [Ciona intestinalis]
XP_018668829.1	1538	LOC100179135	eukaryota>metazoa>chordata	Ciona	intestinalis	PREDICTED: neurabin-1 isoform X2 [Ciona intestinalis]
XP_008191010.1	1531	LOC664255	eukaryota>metazoa>hexapoda	Tribolium	castaneum	PREDICTED: uncharacterized protein LOC664255 isoform X1 [Tribolium castaneum]
XP_008191012.1	1530	LOC664255	eukaryota>metazoa>hexapoda	Tribolium	castaneum	PREDICTED: uncharacterized protein LOC664255 isoform X2 [Tribolium castaneum]
XP_008191013.1	1519	LOC664255	eukaryota>metazoa>hexapoda	Tribolium	castaneum	PREDICTED: uncharacterized protein LOC664255 isoform X3 [Tribolium castaneum]
XP_008191014.1	1519	LOC664255	eukaryota>metazoa>hexapoda	Tribolium	castaneum	PREDICTED: uncharacterized protein LOC664255 isoform X4 [Tribolium castaneum]
XP_013422097.1	1515	LOC106182023	eukaryota>metazoa	Lingula	anatina	PREDICTED: neurabin-1-like isoform X12 [Lingula anatina]
XP_009290272.1	1498	si:ch211-269i23.2	eukaryota>metazoa>chordata>vertebrata>actinopterygii	Danio	rerio	PREDICTED: neurabin-1 isoform X2 [Danio rerio]
XP_018668830.1	1498	LOC100179135	eukaryota>metazoa>chordata	Ciona	intestinalis	PREDICTED: neurabin-1 isoform X3 [Ciona intestinalis]
XP_017312924.1	1495	LOC108258654	eukaryota>metazoa>chordata>vertebrata>actinopterygii	Ictalurus	punctatus	PREDICTED: neurabin-1-like isoform X1 [Ictalurus punctatus]
XP_009172250.1	1487	T265_08239	eukaryota>metazoa	Opisthorchis	viverrini	hypothetical protein T265_08239 [Opisthorchis viverrini]
XP_009290277.1	1482	si:ch211-269i23.2	eukaryota>metazoa>chordata>vertebrata>actinopterygii	Danio	rerio	neurabin-1 isoform X1 [Danio rerio]
XP_014782972.1	1473	LOC106878301	eukaryota>metazoa>mollusca	Octopus	bimaculoides	PREDICTED: neurabin-1-like [Octopus bimaculoides]
XP_013422098.1	1470	LOC106182023	eukaryota>metazoa	Lingula	anatina	PREDICTED: neurabin-1-like isoform X13 [Lingula anatina]
XP_017312926.1	1460	LOC108258654	eukaryota>metazoa>chordata>vertebrata>actinopterygii	Ictalurus	punctatus	PREDICTED: neurabin-1-like isoform X2 [Ictalurus punctatus]
XP_001849160.1	1449	CpipJ_CPIJ007358	eukaryota>metazoa>hexapoda	Culex	quinquefasciatus	conserved hypothetical protein [Culex quinquefasciatus]
XP_018668831.1	1443	LOC100179135	eukaryota>metazoa>chordata	Ciona	intestinalis	PREDICTED: neurabin-2 isoform X4 [Ciona intestinalis]
XP_011607982.1	1435	LOC101065336	eukaryota>metazoa>chordata>vertebrata>actinopterygii	Takifugu	rubripes	PREDICTED: neurabin-1-like isoform X1 [Takifugu rubripes]
XP_011607987.1	1431	LOC101065336	eukaryota>metazoa>chordata>vertebrata>actinopterygii	Takifugu	rubripes	PREDICTED: neurabin-1-like isoform X2 [Takifugu rubripes]
XP_011607991.1	1420	LOC101065336	eukaryota>metazoa>chordata>vertebrata>actinopterygii	Takifugu	rubripes	PREDICTED: neurabin-1-like isoform X3 [Takifugu rubripes]
KXJ17399.1	1420	PPP1R9A	eukaryota>metazoa>cnidaria	Exaiptasia	pallida	Neurabin-1 [Exaiptasia pallida]
XP_011608000.1	1416	LOC101065336	eukaryota>metazoa>chordata>vertebrata>actinopterygii	Takifugu	rubripes	PREDICTED: neurabin-1-like isoform X5 [Takifugu rubripes]
XP_011514682.1	1395	Neurabin-I,NRB1,NRBI	eukaryota>metazoa>chordata>vertebrata	Homo	sapiens	PREDICTED: neurabin-1 isoform X1 [Homo sapiens]
XP_011514683.1	1395	Neurabin-I,NRB1,NRBI	eukaryota>metazoa>chordata>vertebrata	Homo	sapiens	PREDICTED: neurabin-1 isoform X1 [Homo sapiens]
XP_011514684.1	1395	Neurabin-I,NRB1,NRBI	eukaryota>metazoa>chordata>vertebrata	Homo	sapiens	PREDICTED: neurabin-1 isoform X1 [Homo sapiens]
XP_011514685.1	1395	Neurabin-I,NRB1,NRBI	eukaryota>metazoa>chordata>vertebrata	Homo	sapiens	PREDICTED: neurabin-1 isoform X1 [Homo sapiens]
XP_017324930.1	1389	LOC108266327	eukaryota>metazoa>chordata>vertebrata>actinopterygii	Ictalurus	punctatus	PREDICTED: neurabin-1-like isoform X1 [Ictalurus punctatus]
XP_017324931.1	1389	LOC108266327	eukaryota>metazoa>chordata>vertebrata>actinopterygii	Ictalurus	punctatus	PREDICTED: neurabin-1-like isoform X1 [Ictalurus punctatus]
XP_003241912.1	1381	LOC100162635	eukaryota>metazoa>hexapoda	Acyrthosiphon	pisum	PREDICTED: neurabin-1 isoform X1 [Acyrthosiphon pisum]
XP_011607996.1	1381	LOC101065336	eukaryota>metazoa>chordata>vertebrata>actinopterygii	Takifugu	rubripes	PREDICTED: neurabin-1-like isoform X4 [Takifugu rubripes]
XP_015303203.1	1379	PPP1R9A	eukaryota>metazoa>chordata>vertebrata	Macaca	fascicularis	PREDICTED: neurabin-1 isoform X1 [Macaca fascicularis]
XP_015303204.1	1379	PPP1R9A	eukaryota>metazoa>chordata>vertebrata	Macaca	fascicularis	PREDICTED: neurabin-1 isoform X1 [Macaca fascicularis]
XP_015303205.1	1379	PPP1R9A	eukaryota>metazoa>chordata>vertebrata	Macaca	fascicularis	PREDICTED: neurabin-1 isoform X1 [Macaca fascicularis]
XP_015303206.1	1379	PPP1R9A	eukaryota>metazoa>chordata>vertebrata	Macaca	fascicularis	PREDICTED: neurabin-1 isoform X1 [Macaca fascicularis]
XP_015303207.1	1379	PPP1R9A	eukaryota>metazoa>chordata>vertebrata	Macaca	fascicularis	PREDICTED: neurabin-1 isoform X1 [Macaca fascicularis]
XP_016867883.1	1377	Neurabin-I,NRB1,NRBI	eukaryota>metazoa>chordata>vertebrata	Homo	sapiens	PREDICTED: neurabin-1 isoform X2 [Homo sapiens]
NP_001159632.1	1374	Neurabin-I,NRB1,NRBI	eukaryota>metazoa>chordata>vertebrata	Homo	sapiens	neurabin-1 isoform 1 [Homo sapiens]
XP_003241913.1	1374	LOC100162635	eukaryota>metazoa>hexapoda	Acyrthosiphon	pisum	PREDICTED: neurabin-1 isoform X2 [Acyrthosiphon pisum]
XP_003252562.1	1374	PPP1R9A	eukaryota>metazoa>chordata>vertebrata	Nomascus	leucogenys	PREDICTED: neurabin-1 [Nomascus leucogenys]
XP_012365960.1	1374	PPP1R9A	eukaryota>metazoa>chordata>vertebrata	Nomascus	leucogenys	PREDICTED: neurabin-1 [Nomascus leucogenys]
XP_017324932.1	1374	LOC108266327	eukaryota>metazoa>chordata>vertebrata>actinopterygii	Ictalurus	punctatus	PREDICTED: neurabin-1-like isoform X2 [Ictalurus punctatus]
XP_016656811.1	1373	LOC100162635	eukaryota>metazoa>hexapoda	Acyrthosiphon	pisum	PREDICTED: neurabin-1 isoform X3 [Acyrthosiphon pisum]
XP_005550235.1	1371	PPP1R9A	eukaryota>metazoa>chordata>vertebrata	Macaca	fascicularis	PREDICTED: neurabin-1 isoform X2 [Macaca fascicularis]
XP_016867884.1	1365	Neurabin-I,NRB1,NRBI	eukaryota>metazoa>chordata>vertebrata	Homo	sapiens	PREDICTED: neurabin-1 isoform X3 [Homo sapiens]
XP_015145102.1	1364	PPP1R9B	eukaryota>metazoa>chordata>vertebrata	Gallus	gallus	PREDICTED: protein phosphatase 1, regulatory (inhibitor) subunit 9B isoform X1 [Gallus gallus]
XP_015145103.1	1364	PPP1R9B	eukaryota>metazoa>chordata>vertebrata	Gallus	gallus	PREDICTED: protein phosphatase 1, regulatory (inhibitor) subunit 9B isoform X1 [Gallus gallus]
XP_015145104.1	1364	PPP1R9B	eukaryota>metazoa>chordata>vertebrata	Gallus	gallus	PREDICTED: protein phosphatase 1, regulatory (inhibitor) subunit 9B isoform X1 [Gallus gallus]
XP_015145105.1	1364	PPP1R9B	eukaryota>metazoa>chordata>vertebrata	Gallus	gallus	PREDICTED: protein phosphatase 1, regulatory (inhibitor) subunit 9B isoform X1 [Gallus gallus]
XP_015145106.1	1364	PPP1R9B	eukaryota>metazoa>chordata>vertebrata	Gallus	gallus	PREDICTED: protein phosphatase 1, regulatory (inhibitor) subunit 9B isoform X1 [Gallus gallus]
XP_015145108.1	1364	PPP1R9B	eukaryota>metazoa>chordata>vertebrata	Gallus	gallus	PREDICTED: protein phosphatase 1, regulatory (inhibitor) subunit 9B isoform X1 [Gallus gallus]
XP_015145109.1	1364	PPP1R9B	eukaryota>metazoa>chordata>vertebrata	Gallus	gallus	PREDICTED: protein phosphatase 1, regulatory (inhibitor) subunit 9B isoform X1 [Gallus gallus]
XP_015145110.1	1364	PPP1R9B	eukaryota>metazoa>chordata>vertebrata	Gallus	gallus	PREDICTED: protein phosphatase 1, regulatory (inhibitor) subunit 9B isoform X1 [Gallus gallus]
XP_003241914.1	1363	LOC100162635	eukaryota>metazoa>hexapoda	Acyrthosiphon	pisum	PREDICTED: neurabin-1 isoform X4 [Acyrthosiphon pisum]
XP_015303208.1	1361	PPP1R9A	eukaryota>metazoa>chordata>vertebrata	Macaca	fascicularis	PREDICTED: neurabin-1 isoform X3 [Macaca fascicularis]
XP_016867885.1	1360	Neurabin-I,NRB1,NRBI	eukaryota>metazoa>chordata>vertebrata	Homo	sapiens	PREDICTED: neurabin-1 isoform X4 [Homo sapiens]
XP_015303209.1	1357	PPP1R9A	eukaryota>metazoa>chordata>vertebrata	Macaca	fascicularis	PREDICTED: neurabin-1 isoform X4 [Macaca fascicularis]
XP_016867886.1	1357	Neurabin-I,NRB1,NRBI	eukaryota>metazoa>chordata>vertebrata	Homo	sapiens	PREDICTED: neurabin-1 isoform X5 [Homo sapiens]
XP_018094349.1	1354	ppp1r9a.S	eukaryota>metazoa>chordata>vertebrata	Xenopus	laevis	PREDICTED: neurabin-1 isoform X1 [Xenopus laevis]
XP_018094350.1	1354	ppp1r9a.S	eukaryota>metazoa>chordata>vertebrata	Xenopus	laevis	PREDICTED: neurabin-1 isoform X1 [Xenopus laevis]
XP_011514691.1	1352	Neurabin-I,NRB1,NRBI	eukaryota>metazoa>chordata>vertebrata	Homo	sapiens	PREDICTED: neurabin-1 isoform X6 [Homo sapiens]
XP_016867887.1	1352	Neurabin-I,NRB1,NRBI	eukaryota>metazoa>chordata>vertebrata	Homo	sapiens	PREDICTED: neurabin-1 isoform X6 [Homo sapiens]
NP_990254.2	1350	PPP1R9B	eukaryota>metazoa>chordata>vertebrata	Gallus	gallus	protein phosphatase 1, regulatory (inhibitor) subunit 9B [Gallus gallus]
XP_018091399.1	1348	LOC108701371	eukaryota>metazoa>chordata>vertebrata	Xenopus	laevis	PREDICTED: neurabin-1-like isoform X1 [Xenopus laevis]
XP_018091400.1	1348	LOC108701371	eukaryota>metazoa>chordata>vertebrata	Xenopus	laevis	PREDICTED: neurabin-1-like isoform X1 [Xenopus laevis]
XP_018091401.1	1348	LOC108701371	eukaryota>metazoa>chordata>vertebrata	Xenopus	laevis	PREDICTED: neurabin-1-like isoform X1 [Xenopus laevis]
XP_015137405.1	1342	PPP1R9A	eukaryota>metazoa>chordata>vertebrata	Gallus	gallus	PREDICTED: neurabin-1 isoform X1 [Gallus gallus]
XP_015137406.1	1342	PPP1R9A	eukaryota>metazoa>chordata>vertebrata	Gallus	gallus	PREDICTED: neurabin-1 isoform X1 [Gallus gallus]
XP_015137407.1	1342	PPP1R9A	eukaryota>metazoa>chordata>vertebrata	Gallus	gallus	PREDICTED: neurabin-1 isoform X1 [Gallus gallus]
XP_016867888.1	1342	Neurabin-I,NRB1,NRBI	eukaryota>metazoa>chordata>vertebrata	Homo	sapiens	PREDICTED: neurabin-1 isoform X7 [Homo sapiens]
XP_015303210.1	1341	PPP1R9A	eukaryota>metazoa>chordata>vertebrata	Macaca	fascicularis	PREDICTED: neurabin-1 isoform X5 [Macaca fascicularis]
XP_017324933.1	1335	LOC108266327	eukaryota>metazoa>chordata>vertebrata>actinopterygii	Ictalurus	punctatus	PREDICTED: neurabin-1-like isoform X3 [Ictalurus punctatus]
XP_015137408.1	1334	PPP1R9A	eukaryota>metazoa>chordata>vertebrata	Gallus	gallus	PREDICTED: neurabin-1 isoform X2 [Gallus gallus]
XP_015303211.1	1333	PPP1R9A	eukaryota>metazoa>chordata>vertebrata	Macaca	fascicularis	PREDICTED: neurabin-1 isoform X6 [Macaca fascicularis]
XP_700731.6	1332	si:ch1073-219n12.1	eukaryota>metazoa>chordata>vertebrata>actinopterygii	Danio	rerio	neurabin-1 isoform X1 [Danio rerio]
XP_009302707.1	1331	si:ch1073-219n12.1	eukaryota>metazoa>chordata>vertebrata>actinopterygii	Danio	rerio	neurabin-1 isoform X2 [Danio rerio]
XP_015145111.1	1325	PPP1R9B	eukaryota>metazoa>chordata>vertebrata	Gallus	gallus	PREDICTED: protein phosphatase 1, regulatory (inhibitor) subunit 9B isoform X2 [Gallus gallus]
XP_006505133.1	1322	2810430P21Rik,4930518N04Rik,5330407E15,A230094E16Rik,BB181831,mKIAA1222,neurabin-I,NRB	eukaryota>metazoa>chordata>vertebrata	Mus	musculus	PREDICTED: neurabin-1 isoform X1 [Mus musculus]
XP_006505134.1	1322	2810430P21Rik,4930518N04Rik,5330407E15,A230094E16Rik,BB181831,mKIAA1222,neurabin-I,NRB	eukaryota>metazoa>chordata>vertebrata	Mus	musculus	PREDICTED: neurabin-1 isoform X1 [Mus musculus]
XP_006505135.1	1322	2810430P21Rik,4930518N04Rik,5330407E15,A230094E16Rik,BB181831,mKIAA1222,neurabin-I,NRB	eukaryota>metazoa>chordata>vertebrata	Mus	musculus	PREDICTED: neurabin-1 isoform X1 [Mus musculus]
XP_016867889.1	1322	Neurabin-I,NRB1,NRBI	eukaryota>metazoa>chordata>vertebrata	Homo	sapiens	PREDICTED: neurabin-1 isoform X8 [Homo sapiens]
XP_016867890.1	1318	Neurabin-I,NRB1,NRBI	eukaryota>metazoa>chordata>vertebrata	Homo	sapiens	PREDICTED: neurabin-1 isoform X9 [Homo sapiens]
XP_009302708.1	1317	si:ch1073-219n12.1	eukaryota>metazoa>chordata>vertebrata>actinopterygii	Danio	rerio	neurabin-1 isoform X3 [Danio rerio]
XP_018094351.1	1315	ppp1r9a.S	eukaryota>metazoa>chordata>vertebrata	Xenopus	laevis	PREDICTED: neurabin-1 isoform X2 [Xenopus laevis]
XP_006505136.1	1314	2810430P21Rik,4930518N04Rik,5330407E15,A230094E16Rik,BB181831,mKIAA1222,neurabin-I,NRB	eukaryota>metazoa>chordata>vertebrata	Mus	musculus	PREDICTED: neurabin-1 isoform X2 [Mus musculus]
XP_015303212.1	1310	PPP1R9A	eukaryota>metazoa>chordata>vertebrata	Macaca	fascicularis	PREDICTED: neurabin-1 isoform X7 [Macaca fascicularis]
XP_018091402.1	1309	LOC108701371	eukaryota>metazoa>chordata>vertebrata	Xenopus	laevis	PREDICTED: neurabin-1-like isoform X2 [Xenopus laevis]
XP_016867891.1	1304	Neurabin-I,NRB1,NRBI	eukaryota>metazoa>chordata>vertebrata	Homo	sapiens	PREDICTED: neurabin-1 isoform X10 [Homo sapiens]
XP_017177079.1	1304	2810430P21Rik,4930518N04Rik,5330407E15,A230094E16Rik,BB181831,mKIAA1222,neurabin-I,NRB	eukaryota>metazoa>chordata>vertebrata	Mus	musculus	PREDICTED: neurabin-1 isoform X3 [Mus musculus]
XP_006505138.1	1300	2810430P21Rik,4930518N04Rik,5330407E15,A230094E16Rik,BB181831,mKIAA1222,neurabin-I,NRB	eukaryota>metazoa>chordata>vertebrata	Mus	musculus	PREDICTED: neurabin-1 isoform X4 [Mus musculus]
NP_001159633.1	1296	Neurabin-I,NRB1,NRBI	eukaryota>metazoa>chordata>vertebrata	Homo	sapiens	neurabin-1 isoform 2 [Homo sapiens]
XP_016867892.1	1296	Neurabin-I,NRB1,NRBI	eukaryota>metazoa>chordata>vertebrata	Homo	sapiens	PREDICTED: neurabin-1 isoform X11 [Homo sapiens]
XP_015303213.1	1293	PPP1R9A	eukaryota>metazoa>chordata>vertebrata	Macaca	fascicularis	PREDICTED: neurabin-1 isoform X8 [Macaca fascicularis]
XP_018094352.1	1293	ppp1r9a.S	eukaryota>metazoa>chordata>vertebrata	Xenopus	laevis	PREDICTED: neurabin-1 isoform X3 [Xenopus laevis]
XP_006505139.1	1292	2810430P21Rik,4930518N04Rik,5330407E15,A230094E16Rik,BB181831,mKIAA1222,neurabin-I,NRB	eukaryota>metazoa>chordata>vertebrata	Mus	musculus	PREDICTED: neurabin-1 isoform X5 [Mus musculus]
OQV23782.1	1288	BV898_02510	eukaryota>metazoa	Hypsibius	dujardini	Neurabin-1 [Hypsibius dujardini]
XP_018091403.1	1287	LOC108701371	eukaryota>metazoa>chordata>vertebrata	Xenopus	laevis	PREDICTED: neurabin-1-like isoform X3 [Xenopus laevis]
XP_009302709.1	1278	si:ch1073-219n12.1	eukaryota>metazoa>chordata>vertebrata>actinopterygii	Danio	rerio	neurabin-1 isoform X4 [Danio rerio]
XP_017324934.1	1272	LOC108266327	eukaryota>metazoa>chordata>vertebrata>actinopterygii	Ictalurus	punctatus	PREDICTED: neurabin-1-like isoform X4 [Ictalurus punctatus]
NP_001159634.1	1253	Neurabin-I,NRB1,NRBI	eukaryota>metazoa>chordata>vertebrata	Homo	sapiens	neurabin-1 isoform 3 [Homo sapiens]
XP_015303214.1	1250	PPP1R9A	eukaryota>metazoa>chordata>vertebrata	Macaca	fascicularis	PREDICTED: neurabin-1 isoform X9 [Macaca fascicularis]
CDS42871.1	1225	EmuJ_001059000	eukaryota>metazoa	Echinococcus	multilocularis	neurabin 1 [Echinococcus multilocularis]
XP_018648611.1	1214	Smp_142650.1	eukaryota>metazoa	Schistosoma	mansoni	hypothetical protein Smp_142650.1 [Schistosoma mansoni]
XP_011614826.1	1210	LOC101070018	eukaryota>metazoa>chordata>vertebrata>actinopterygii	Takifugu	rubripes	PREDICTED: neurabin-1-like isoform X1 [Takifugu rubripes]
XP_011614827.1	1203	LOC101070018	eukaryota>metazoa>chordata>vertebrata>actinopterygii	Takifugu	rubripes	PREDICTED: neurabin-1-like isoform X2 [Takifugu rubripes]
XP_013422099.1	1163	LOC106182023	eukaryota>metazoa	Lingula	anatina	PREDICTED: neurabin-1-like isoform X14 [Lingula anatina]
XP_018094353.1	1145	ppp1r9a.S	eukaryota>metazoa>chordata>vertebrata	Xenopus	laevis	PREDICTED: neurabin-2 isoform X4 [Xenopus laevis]
XP_016867893.1	1141	Neurabin-I,NRB1,NRBI	eukaryota>metazoa>chordata>vertebrata	Homo	sapiens	PREDICTED: neurabin-1 isoform X12 [Homo sapiens]
XP_015137409.1	1120	PPP1R9A	eukaryota>metazoa>chordata>vertebrata	Gallus	gallus	PREDICTED: neurabin-1 isoform X3 [Gallus gallus]
XP_015303215.1	1120	PPP1R9A	eukaryota>metazoa>chordata>vertebrata	Macaca	fascicularis	PREDICTED: neurabin-1 isoform X10 [Macaca fascicularis]
XP_018122809.1	1120	LOC108718842	eukaryota>metazoa>chordata>vertebrata	Xenopus	laevis	PREDICTED: neurabin-1-like isoform X1 [Xenopus laevis]
XP_018124679.1	1119	LOC108719905	eukaryota>metazoa>chordata>vertebrata	Xenopus	laevis	PREDICTED: neurabin-1-like isoform X1 [Xenopus laevis]
XP_006505141.1	1117	2810430P21Rik,4930518N04Rik,5330407E15,A230094E16Rik,BB181831,mKIAA1222,neurabin-I,NRB	eukaryota>metazoa>chordata>vertebrata	Mus	musculus	PREDICTED: neurabin-1 isoform X6 [Mus musculus]
XP_015137410.1	1112	PPP1R9A	eukaryota>metazoa>chordata>vertebrata	Gallus	gallus	PREDICTED: neurabin-1 isoform X4 [Gallus gallus]
XP_017309983.1	1110	ppp1r9a	eukaryota>metazoa>chordata>vertebrata>actinopterygii	Ictalurus	punctatus	PREDICTED: LOW QUALITY PROTEIN: neurabin-1 [Ictalurus punctatus]
XP_018122810.1	1105	LOC108718842	eukaryota>metazoa>chordata>vertebrata	Xenopus	laevis	PREDICTED: neurabin-1-like isoform X2 [Xenopus laevis]
XP_018124680.1	1104	LOC108719905	eukaryota>metazoa>chordata>vertebrata	Xenopus	laevis	PREDICTED: neurabin-1-like isoform X2 [Xenopus laevis]
XP_011607793.1	1100	ppp1r9a	eukaryota>metazoa>chordata>vertebrata>actinopterygii	Takifugu	rubripes	PREDICTED: neurabin-1 isoform X1 [Takifugu rubripes]
XP_011607794.1	1100	ppp1r9a	eukaryota>metazoa>chordata>vertebrata>actinopterygii	Takifugu	rubripes	PREDICTED: neurabin-1 isoform X1 [Takifugu rubripes]
XP_011607795.1	1100	ppp1r9a	eukaryota>metazoa>chordata>vertebrata>actinopterygii	Takifugu	rubripes	PREDICTED: neurabin-1 isoform X1 [Takifugu rubripes]
NP_060120.2	1098	Neurabin-I,NRB1,NRBI	eukaryota>metazoa>chordata>vertebrata	Homo	sapiens	neurabin-1 isoform 4 [Homo sapiens]
XP_015303216.1	1098	PPP1R9A	eukaryota>metazoa>chordata>vertebrata	Macaca	fascicularis	PREDICTED: neurabin-1 isoform X11 [Macaca fascicularis]
XP_016867894.1	1098	Neurabin-I,NRB1,NRBI	eukaryota>metazoa>chordata>vertebrata	Homo	sapiens	PREDICTED: neurabin-1 isoform X13 [Homo sapiens]
XP_016867895.1	1098	Neurabin-I,NRB1,NRBI	eukaryota>metazoa>chordata>vertebrata	Homo	sapiens	PREDICTED: neurabin-1 isoform X13 [Homo sapiens]
NP_853626.1	1095	2810430P21Rik,4930518N04Rik,5330407E15,A230094E16Rik,BB181831,mKIAA1222,neurabin-I,NRB	eukaryota>metazoa>chordata>vertebrata	Mus	musculus	neurabin-1 [Mus musculus]
XP_006505142.1	1095	2810430P21Rik,4930518N04Rik,5330407E15,A230094E16Rik,BB181831,mKIAA1222,neurabin-I,NRB	eukaryota>metazoa>chordata>vertebrata	Mus	musculus	PREDICTED: neurabin-1 isoform X7 [Mus musculus]
XP_011607796.1	1092	ppp1r9a	eukaryota>metazoa>chordata>vertebrata>actinopterygii	Takifugu	rubripes	PREDICTED: neurabin-1 isoform X2 [Takifugu rubripes]
NP_001159635.1	1090	Neurabin-I,NRB1,NRBI	eukaryota>metazoa>chordata>vertebrata	Homo	sapiens	neurabin-1 isoform 5 [Homo sapiens]
XP_015303217.1	1090	PPP1R9A	eukaryota>metazoa>chordata>vertebrata	Macaca	fascicularis	PREDICTED: neurabin-1 isoform X12 [Macaca fascicularis]
XP_016867896.1	1090	Neurabin-I,NRB1,NRBI	eukaryota>metazoa>chordata>vertebrata	Homo	sapiens	PREDICTED: neurabin-1 isoform X14 [Homo sapiens]
XP_002110467.1	1088	TRIADDRAFT_54506	eukaryota>metazoa>placozoa	Trichoplax	adhaerens	hypothetical protein TRIADDRAFT_54506 [Trichoplax adhaerens]
XP_006505143.1	1087	2810430P21Rik,4930518N04Rik,5330407E15,A230094E16Rik,BB181831,mKIAA1222,neurabin-I,NRB	eukaryota>metazoa>chordata>vertebrata	Mus	musculus	PREDICTED: neurabin-1 isoform X8 [Mus musculus]
XP_011607797.1	1085	ppp1r9a	eukaryota>metazoa>chordata>vertebrata>actinopterygii	Takifugu	rubripes	PREDICTED: neurabin-1 isoform X3 [Takifugu rubripes]
XP_009292641.1	1076	ppp1r9a	eukaryota>metazoa>chordata>vertebrata>actinopterygii	Danio	rerio	neurabin-1 isoform X1 [Danio rerio]
XP_689353.6	1076	ppp1r9a	eukaryota>metazoa>chordata>vertebrata>actinopterygii	Danio	rerio	neurabin-1 isoform X1 [Danio rerio]
XP_009292642.1	1068	ppp1r9a	eukaryota>metazoa>chordata>vertebrata>actinopterygii	Danio	rerio	neurabin-1 isoform X2 [Danio rerio]
XP_018648612.1	956	Smp_142650.1	eukaryota>metazoa	Schistosoma	mansoni	hypothetical protein Smp_142650.1 [Schistosoma mansoni]
XP_006817593.1	925	LOC100373924	eukaryota>metazoa>hemichordata	Saccoglossus	kowalevskii	PREDICTED: neurabin-2-like [Saccoglossus kowalevskii]
XP_011239361.1	913	2810430P21Rik,4930518N04Rik,5330407E15,A230094E16Rik,BB181831,mKIAA1222,neurabin-I,NRB	eukaryota>metazoa>chordata>vertebrata	Mus	musculus	PREDICTED: neurabin-1 isoform X9 [Mus musculus]
XP_006505145.1	893	2810430P21Rik,4930518N04Rik,5330407E15,A230094E16Rik,BB181831,mKIAA1222,neurabin-I,NRB	eukaryota>metazoa>chordata>vertebrata	Mus	musculus	PREDICTED: neurabin-1 isoform X10 [Mus musculus]
XP_011601892.1	881	LOC101067768	eukaryota>metazoa>chordata>vertebrata>actinopterygii	Takifugu	rubripes	PREDICTED: neurabin-2-like [Takifugu rubripes]
XP_015129132.1	879	LOC100859037	eukaryota>metazoa>chordata>vertebrata	Gallus	gallus	PREDICTED: neurabin-2 [Gallus gallus]
XP_017177080.1	871	2810430P21Rik,4930518N04Rik,5330407E15,A230094E16Rik,BB181831,mKIAA1222,neurabin-I,NRB	eukaryota>metazoa>chordata>vertebrata	Mus	musculus	PREDICTED: neurabin-1 isoform X11 [Mus musculus]
XP_016867897.1	863	Neurabin-I,NRB1,NRBI	eukaryota>metazoa>chordata>vertebrata	Homo	sapiens	PREDICTED: neurabin-1 isoform X15 [Homo sapiens]
CAG05970.1	853	GSTEN:00026173:G:001	eukaryota>metazoa>chordata>vertebrata>actinopterygii	Tetraodon	nigroviridis	unnamed protein product [Tetraodon nigroviridis]
XP_012358776.1	827	PPP1R9B	eukaryota>metazoa>chordata>vertebrata	Nomascus	leucogenys	PREDICTED: LOW QUALITY PROTEIN: neurabin-2 [Nomascus leucogenys]
XP_005583711.1	818	PPP1R9B	eukaryota>metazoa>chordata>vertebrata	Macaca	fascicularis	PREDICTED: neurabin-2 [Macaca fascicularis]
NP_115984.3	817	PPP1R6,PPP1R9,SPINO,Spn	eukaryota>metazoa>chordata>vertebrata	Homo	sapiens	neurabin-2 [Homo sapiens]
NP_758465.2	817	SPL,Spn	eukaryota>metazoa>chordata>vertebrata	Mus	musculus	neurabin-2 [Mus musculus]
XP_003961061.1	814	LOC101063632	eukaryota>metazoa>chordata>vertebrata>actinopterygii	Takifugu	rubripes	PREDICTED: neurabin-2-like [Takifugu rubripes]
XP_017347267.1	814	ppp1r9b	eukaryota>metazoa>chordata>vertebrata>actinopterygii	Ictalurus	punctatus	PREDICTED: neurabin-2 [Ictalurus punctatus]
XP_686514.3	803	ppp1r9ba	eukaryota>metazoa>chordata>vertebrata>actinopterygii	Danio	rerio	neurabin-2 [Danio rerio]
XP_009304793.1	801	ppp1r9bb	eukaryota>metazoa>chordata>vertebrata>actinopterygii	Danio	rerio	neurabin-2 [Danio rerio]
XP_002402226.1	782	IscW_ISCW009137	eukaryota>metazoa	Ixodes	scapularis	hypothetical protein IscW_ISCW009137 [Ixodes scapularis]
CAG09960.1	777	GSTEN:00031436:G:001	eukaryota>metazoa>chordata>vertebrata>actinopterygii	Tetraodon	nigroviridis	unnamed protein product, partial [Tetraodon nigroviridis]
XP_018090314.1	748	ppp1r9b.L	eukaryota>metazoa>chordata>vertebrata	Xenopus	laevis	PREDICTED: neurabin-2 [Xenopus laevis]
XP_018093561.1	748	LOC108702542	eukaryota>metazoa>chordata>vertebrata	Xenopus	laevis	PREDICTED: neurabin-2-like [Xenopus laevis]
XP_001900978.1	744	Bm1_47585	eukaryota>metazoa>nematoda	Brugia	malayi	Neurabin protein 1, isoform d [Brugia malayi]
XP_016867898.1	743	Neurabin-I,NRB1,NRBI	eukaryota>metazoa>chordata>vertebrata	Homo	sapiens	PREDICTED: neurabin-1 isoform X16 [Homo sapiens]
ERG81279.1	735	ASU_10442	eukaryota>metazoa>nematoda	Ascaris	suum	neurabin-1 [Ascaris suum]
NP_491332.1	721	nab-1	eukaryota>metazoa>nematoda	Caenorhabditis	elegans	NeurABin [Caenorhabditis elegans]
XP_016867899.1	720	Neurabin-I,NRB1,NRBI	eukaryota>metazoa>chordata>vertebrata	Homo	sapiens	PREDICTED: neurabin-1 isoform X17 [Homo sapiens]
XP_014681128.1	717	LOC106821009	eukaryota>metazoa	Priapulus	caudatus	PREDICTED: neurabin-2-like [Priapulus caudatus]
XP_015761351.1	712	LOC107340517	eukaryota>metazoa>cnidaria	Acropora	digitifera	PREDICTED: neurabin-1-like isoform X1 [Acropora digitifera]
XP_016867900.1	709	Neurabin-I,NRB1,NRBI	eukaryota>metazoa>chordata>vertebrata	Homo	sapiens	PREDICTED: neurabin-1 isoform X18 [Homo sapiens]
XP_003373360.1	707	Tsp_07958	eukaryota>metazoa>nematoda	Trichinella	spiralis	putative PDZ domain protein [Trichinella spiralis]
XP_018648613.1	698	Smp_142650.1	eukaryota>metazoa	Schistosoma	mansoni	hypothetical protein Smp_142650.1 [Schistosoma mansoni]
XP_013790281.1	685	LOC106474138	eukaryota>metazoa	Limulus	polyphemus	PREDICTED: neurabin-2-like, partial [Limulus polyphemus]
NP_740837.1	658	nab-1	eukaryota>metazoa>nematoda	Caenorhabditis	elegans	NeurABin [Caenorhabditis elegans]
CAG09961.1	632	GSTEN:00031437:G:001	eukaryota>metazoa>chordata>vertebrata>actinopterygii	Tetraodon	nigroviridis	unnamed protein product [Tetraodon nigroviridis]
XP_011523683.1	631	PPP1R6,PPP1R9,SPINO,Spn	eukaryota>metazoa>chordata>vertebrata	Homo	sapiens	PREDICTED: neurabin-2 isoform X1 [Homo sapiens]
NP_491333.1	561	nab-1	eukaryota>metazoa>nematoda	Caenorhabditis	elegans	NeurABin [Caenorhabditis elegans]
NP_001021041.1	527	nab-1	eukaryota>metazoa>nematoda	Caenorhabditis	elegans	NeurABin [Caenorhabditis elegans]
CBY21187.1	527	GSOID_T00008942001	eukaryota>metazoa>chordata	Oikopleura	dioica	unnamed protein product [Oikopleura dioica]
EUB61930.1	487	EGR_03203	eukaryota>metazoa	Echinococcus	granulosus	Neurabin-1 [Echinococcus granulosus]
CAG06578.1	408	GSTEN:00026989:G:001	eukaryota>metazoa>chordata>vertebrata>actinopterygii	Tetraodon	nigroviridis	unnamed protein product, partial [Tetraodon nigroviridis]
CAG06577.1	366	GSTEN:00026988:G:001	eukaryota>metazoa>chordata>vertebrata>actinopterygii	Tetraodon	nigroviridis	unnamed protein product [Tetraodon nigroviridis]
XP_009061357.1	308	LOTGIDRAFT_126878	eukaryota>metazoa>mollusca	Lottia	gigantea	hypothetical protein LOTGIDRAFT_126878, partial [Lottia gigantea]
XP_014679436.1	271	LOC106819305	eukaryota>metazoa	Priapulus	caudatus	PREDICTED: neurabin-2-like, partial [Priapulus caudatus]
Smed1000019763	204	Smed1000019763	eukaryota>metazoa>platyhelminthe	Schmidtea	mediterranea	>Smed1000019763
EFX69045.1	112	DAPPUDRAFT_62650	eukaryota>metazoa>crustacea	Daphnia	pulex	hypothetical protein DAPPUDRAFT_62650 [Daphnia pulex]
XP_009016652.1	81	HELRODRAFT_137735	eukaryota>metazoa>annelida	Helobdella	robusta	hypothetical protein HELRODRAFT_137735, partial [Helobdella robusta]

AF. Tespa1 (KRAP dom.)

XP_006815276.1	1843	LOC102801861	eukaryota>metazoa>hemichordata	Saccoglossus	kowalevskii	PREDICTED: uncharacterized protein LOC102801861 [Saccoglossus kowalevskii]
XP_006819366.1	1765	LOC100376122	eukaryota>metazoa>hemichordata	Saccoglossus	kowalevskii	PREDICTED: uncharacterized protein LOC100376122 [Saccoglossus kowalevskii]
XP_014674489.1	1643	LOC106814654	eukaryota>metazoa	Priapulus	caudatus	PREDICTED: uncharacterized protein LOC106814654 [Priapulus caudatus]
XP_013397679.1	1466	LOC106164348	eukaryota>metazoa	Lingula	anatina	uncharacterized protein LOC106164348 [Lingula anatina]
XP_013397680.1	1466	LOC106164348	eukaryota>metazoa	Lingula	anatina	uncharacterized protein LOC106164348 [Lingula anatina]
XP_013397682.1	1466	LOC106164348	eukaryota>metazoa	Lingula	anatina	uncharacterized protein LOC106164348 [Lingula anatina]
XP_011661172.1	1430	LOC575011	eukaryota>metazoa>echinodermata	Strongylocentrotus	purpuratus	PREDICTED: uncharacterized protein LOC575011 [Strongylocentrotus purpuratus]
XP_011661173.1	1430	LOC575011	eukaryota>metazoa>echinodermata	Strongylocentrotus	purpuratus	PREDICTED: uncharacterized protein LOC575011 [Strongylocentrotus purpuratus]
XP_019918619.1	1384	LOC105317898	eukaryota>metazoa>mollusca	Crassostrea	gigas	PREDICTED: sperm-specific antigen 2-like isoform X1 [Crassostrea gigas]
XP_011605472.1	1372	ssfa2	eukaryota>metazoa>chordata>vertebrata>actinopterygii	Takifugu	rubripes	PREDICTED: sperm-specific antigen 2 isoform X1 [Takifugu rubripes]
XP_017325253.1	1372	ssfa2	eukaryota>metazoa>chordata>vertebrata>actinopterygii	Ictalurus	punctatus	PREDICTED: sperm-specific antigen 2 isoform X1 [Ictalurus punctatus]
XP_017325254.1	1372	ssfa2	eukaryota>metazoa>chordata>vertebrata>actinopterygii	Ictalurus	punctatus	PREDICTED: sperm-specific antigen 2 isoform X1 [Ictalurus punctatus]
XP_017325256.1	1372	ssfa2	eukaryota>metazoa>chordata>vertebrata>actinopterygii	Ictalurus	punctatus	PREDICTED: sperm-specific antigen 2 isoform X1 [Ictalurus punctatus]
XP_017325257.1	1372	ssfa2	eukaryota>metazoa>chordata>vertebrata>actinopterygii	Ictalurus	punctatus	PREDICTED: sperm-specific antigen 2 isoform X1 [Ictalurus punctatus]
XP_017325258.1	1372	ssfa2	eukaryota>metazoa>chordata>vertebrata>actinopterygii	Ictalurus	punctatus	PREDICTED: sperm-specific antigen 2 isoform X1 [Ictalurus punctatus]
XP_017325259.1	1372	ssfa2	eukaryota>metazoa>chordata>vertebrata>actinopterygii	Ictalurus	punctatus	PREDICTED: sperm-specific antigen 2 isoform X1 [Ictalurus punctatus]
XP_017325260.1	1372	ssfa2	eukaryota>metazoa>chordata>vertebrata>actinopterygii	Ictalurus	punctatus	PREDICTED: sperm-specific antigen 2 isoform X1 [Ictalurus punctatus]
XP_017325261.1	1371	ssfa2	eukaryota>metazoa>chordata>vertebrata>actinopterygii	Ictalurus	punctatus	PREDICTED: sperm-specific antigen 2 isoform X2 [Ictalurus punctatus]
XP_011605477.1	1352	ssfa2	eukaryota>metazoa>chordata>vertebrata>actinopterygii	Takifugu	rubripes	PREDICTED: sperm-specific antigen 2 isoform X2 [Takifugu rubripes]
XP_017325262.1	1347	ssfa2	eukaryota>metazoa>chordata>vertebrata>actinopterygii	Ictalurus	punctatus	PREDICTED: sperm-specific antigen 2 isoform X3 [Ictalurus punctatus]
XP_002588284.1	1341	BRAFLDRAFT_86732	eukaryota>metazoa>chordata	Branchiostoma	floridae	hypothetical protein BRAFLDRAFT_86732 [Branchiostoma floridae]
XP_022253392.1	1306	LOC111088227	eukaryota>metazoa	Limulus	polyphemus	uncharacterized protein LOC111088227 isoform X1 [Limulus polyphemus]
XP_022253393.1	1306	LOC111088227	eukaryota>metazoa	Limulus	polyphemus	uncharacterized protein LOC111088227 isoform X1 [Limulus polyphemus]
XP_022253395.1	1306	LOC111088227	eukaryota>metazoa	Limulus	polyphemus	uncharacterized protein LOC111088227 isoform X1 [Limulus polyphemus]
XP_022253396.1	1306	LOC111088227	eukaryota>metazoa	Limulus	polyphemus	uncharacterized protein LOC111088227 isoform X1 [Limulus polyphemus]
XP_022253397.1	1298	LOC111088227	eukaryota>metazoa	Limulus	polyphemus	uncharacterized protein LOC111088227 isoform X2 [Limulus polyphemus]
XP_017213332.1	1274	ssfa2	eukaryota>metazoa>chordata>vertebrata>actinopterygii	Danio	rerio	sperm-specific antigen 2 [Danio rerio]
XP_021334691.1	1274	ssfa2	eukaryota>metazoa>chordata>vertebrata>actinopterygii	Danio	rerio	sperm-specific antigen 2 [Danio rerio]
XP_015145038.1	1266	SSFA2	eukaryota>metazoa>chordata>vertebrata	Gallus	gallus	sperm-specific antigen 2 [Gallus gallus]
NP_001123917.1	1259	CS-1,CS1,KRAP,SPAG13,SSFA2	eukaryota>metazoa>chordata>vertebrata	Homo	sapiens	sperm-specific antigen 2 isoform 1 [Homo sapiens]
XP_005573685.1	1259	SSFA2	eukaryota>metazoa>chordata>vertebrata	Macaca	fascicularis	PREDICTED: sperm-specific antigen 2 isoform X1 [Macaca fascicularis]
NP_006742.2	1256	CS-1,CS1,KRAP,SPAG13,SSFA2	eukaryota>metazoa>chordata>vertebrata	Homo	sapiens	sperm-specific antigen 2 isoform 2 [Homo sapiens]
XP_005573686.1	1256	SSFA2	eukaryota>metazoa>chordata>vertebrata	Macaca	fascicularis	PREDICTED: sperm-specific antigen 2 isoform X4 [Macaca fascicularis]
XP_005246869.1	1255	CS-1,CS1,KRAP,SPAG13	eukaryota>metazoa>chordata>vertebrata	Homo	sapiens	sperm-specific antigen 2 isoform X1 [Homo sapiens]
XP_005573687.1	1255	SSFA2	eukaryota>metazoa>chordata>vertebrata	Macaca	fascicularis	PREDICTED: sperm-specific antigen 2 isoform X2 [Macaca fascicularis]
NP_542125.3	1252	5730488C15Rik,CS-1,CS1,KRAP,mKIAA1927,SPAG13	eukaryota>metazoa>chordata>vertebrata	Mus	musculus	sperm-specific antigen 2 homolog [Mus musculus]
XP_005246870.1	1252	CS-1,CS1,KRAP,SPAG13	eukaryota>metazoa>chordata>vertebrata	Homo	sapiens	sperm-specific antigen 2 isoform X2 [Homo sapiens]
NP_001274432.1	1237	CS-1,CS1,KRAP,SPAG13,SSFA2	eukaryota>metazoa>chordata>vertebrata	Homo	sapiens	sperm-specific antigen 2 isoform 3 [Homo sapiens]
XP_005573688.1	1237	SSFA2	eukaryota>metazoa>chordata>vertebrata	Macaca	fascicularis	PREDICTED: sperm-specific antigen 2 isoform X3 [Macaca fascicularis]
XP_016860271.1	1234	CS-1,CS1,KRAP,SPAG13	eukaryota>metazoa>chordata>vertebrata	Homo	sapiens	sperm-specific antigen 2 isoform X3 [Homo sapiens]
XP_018094388.1	1227	LOC108703020	eukaryota>metazoa>chordata>vertebrata	Xenopus	laevis	PREDICTED: sperm-specific antigen 2-like isoform X1 [Xenopus laevis]
XP_018094389.1	1227	LOC108703020	eukaryota>metazoa>chordata>vertebrata	Xenopus	laevis	PREDICTED: sperm-specific antigen 2-like isoform X1 [Xenopus laevis]
XP_018094390.1	1226	LOC108703020	eukaryota>metazoa>chordata>vertebrata	Xenopus	laevis	PREDICTED: sperm-specific antigen 2-like isoform X2 [Xenopus laevis]
XP_018091497.1	1224	LOC108701400	eukaryota>metazoa>chordata>vertebrata	Xenopus	laevis	PREDICTED: sperm-specific antigen 2-like isoform X1 [Xenopus laevis]
XP_018091498.1	1223	LOC108701400	eukaryota>metazoa>chordata>vertebrata	Xenopus	laevis	PREDICTED: sperm-specific antigen 2-like isoform X2 [Xenopus laevis]
XP_009023601.1	1208	HELRODRAFT_177142	eukaryota>metazoa>annelida	Helobdella	robusta	hypothetical protein HELRODRAFT_177142 [Helobdella robusta]
XP_018091499.1	1202	LOC108701400	eukaryota>metazoa>chordata>vertebrata	Xenopus	laevis	PREDICTED: sperm-specific antigen 2-like isoform X3 [Xenopus laevis]
XP_018094391.1	1136	LOC108703020	eukaryota>metazoa>chordata>vertebrata	Xenopus	laevis	PREDICTED: sperm-specific antigen 2-like isoform X3 [Xenopus laevis]
XP_012356848.1	1105	SSFA2	eukaryota>metazoa>chordata>vertebrata	Nomascus	leucogenys	PREDICTED: sperm-specific antigen 2 isoform X1 [Nomascus leucogenys]
XP_012356853.1	1105	SSFA2	eukaryota>metazoa>chordata>vertebrata	Nomascus	leucogenys	PREDICTED: sperm-specific antigen 2 isoform X1 [Nomascus leucogenys]
XP_012356855.1	1102	SSFA2	eukaryota>metazoa>chordata>vertebrata	Nomascus	leucogenys	PREDICTED: sperm-specific antigen 2 isoform X2 [Nomascus leucogenys]
NP_001274433.1	1084	CS-1,CS1,KRAP,SPAG13,SSFA2	eukaryota>metazoa>chordata>vertebrata	Homo	sapiens	sperm-specific antigen 2 isoform 4 [Homo sapiens]
XP_003253855.1	1083	SSFA2	eukaryota>metazoa>chordata>vertebrata	Nomascus	leucogenys	PREDICTED: sperm-specific antigen 2 isoform X3 [Nomascus leucogenys]
XP_012356865.1	1083	SSFA2	eukaryota>metazoa>chordata>vertebrata	Nomascus	leucogenys	PREDICTED: sperm-specific antigen 2 isoform X3 [Nomascus leucogenys]
XP_018091500.1	1081	LOC108701400	eukaryota>metazoa>chordata>vertebrata	Xenopus	laevis	PREDICTED: sperm-specific antigen 2-like isoform X4 [Xenopus laevis]
XP_018091501.1	1081	LOC108701400	eukaryota>metazoa>chordata>vertebrata	Xenopus	laevis	PREDICTED: sperm-specific antigen 2-like isoform X4 [Xenopus laevis]
XP_005549906.1	1070	CCDC129	eukaryota>metazoa>chordata>vertebrata	Macaca	fascicularis	PREDICTED: coiled-coil domain-containing protein 129 [Macaca fascicularis]
XP_003270546.2	1065	CCDC129	eukaryota>metazoa>chordata>vertebrata	Nomascus	leucogenys	PREDICTED: coiled-coil domain-containing protein 129 [Nomascus leucogenys]
NP_001244897.1	1062	CCDC129	eukaryota>metazoa>chordata>vertebrata	Homo	sapiens	coiled-coil domain-containing protein 129 isoform 3 [Homo sapiens]
XP_015137147.1	1057	CCDC129	eukaryota>metazoa>chordata>vertebrata	Gallus	gallus	coiled-coil domain-containing protein 129 isoform X1 [Gallus gallus]
NP_001244896.1	1054	CCDC129	eukaryota>metazoa>chordata>vertebrata	Homo	sapiens	coiled-coil domain-containing protein 129 isoform 1 [Homo sapiens]
XP_019918620.1	1051	LOC105317898	eukaryota>metazoa>mollusca	Crassostrea	gigas	PREDICTED: sperm-specific antigen 2-like isoform X2 [Crassostrea gigas]
NP_919276.2	1044	CCDC129	eukaryota>metazoa>chordata>vertebrata	Homo	sapiens	coiled-coil domain-containing protein 129 isoform 2 [Homo sapiens]
XP_011513515.1	1044	CCDC129	eukaryota>metazoa>chordata>vertebrata	Homo	sapiens	coiled-coil domain-containing protein 129 isoform X1 [Homo sapiens]
XP_016867360.1	1044	CCDC129	eukaryota>metazoa>chordata>vertebrata	Homo	sapiens	coiled-coil domain-containing protein 129 isoform X1 [Homo sapiens]
XP_016867361.1	1043	CCDC129	eukaryota>metazoa>chordata>vertebrata	Homo	sapiens	coiled-coil domain-containing protein 129 isoform X2 [Homo sapiens]
XP_015137148.1	1036	CCDC129	eukaryota>metazoa>chordata>vertebrata	Gallus	gallus	coiled-coil domain-containing protein 129 isoform X2 [Gallus gallus]
XP_015137149.1	1036	CCDC129	eukaryota>metazoa>chordata>vertebrata	Gallus	gallus	coiled-coil domain-containing protein 129 isoform X2 [Gallus gallus]
XP_015137150.1	1036	CCDC129	eukaryota>metazoa>chordata>vertebrata	Gallus	gallus	coiled-coil domain-containing protein 129 isoform X2 [Gallus gallus]
XP_006506009.1	1035	D530004J12Rik,Gm458	eukaryota>metazoa>chordata>vertebrata	Mus	musculus	PREDICTED: coiled-coil domain-containing protein 129 isoform X1 [Mus musculus]
XP_006506010.1	1035	D530004J12Rik,Gm458	eukaryota>metazoa>chordata>vertebrata	Mus	musculus	PREDICTED: coiled-coil domain-containing protein 129 isoform X1 [Mus musculus]
NP_001075134.1	1034	D530004J12Rik,Gm458	eukaryota>metazoa>chordata>vertebrata	Mus	musculus	coiled-coil domain-containing protein 129 [Mus musculus]
XP_006506012.1	1034	D530004J12Rik,Gm458	eukaryota>metazoa>chordata>vertebrata	Mus	musculus	PREDICTED: coiled-coil domain-containing protein 129 isoform X3 [Mus musculus]
XP_017177013.1	1034	D530004J12Rik,Gm458	eukaryota>metazoa>chordata>vertebrata	Mus	musculus	PREDICTED: coiled-coil domain-containing protein 129 isoform X2 [Mus musculus]
XP_002110876.1	987	TRIADDRAFT_54246	eukaryota>metazoa>placozoa	Trichoplax	adhaerens	hypothetical protein TRIADDRAFT_54246 [Trichoplax adhaerens]
XP_011510003.1	957	CS-1,CS1,KRAP,SPAG13	eukaryota>metazoa>chordata>vertebrata	Homo	sapiens	sperm-specific antigen 2 isoform X4 [Homo sapiens]
XP_018123069.1	940	LOC108719001	eukaryota>metazoa>chordata>vertebrata	Xenopus	laevis	PREDICTED: coiled-coil domain-containing protein 129-like isoform X1 [Xenopus laevis]
XP_018123070.1	940	LOC108719001	eukaryota>metazoa>chordata>vertebrata	Xenopus	laevis	PREDICTED: coiled-coil domain-containing protein 129-like isoform X1 [Xenopus laevis]
XP_017346686.1	936	ccdc129	eukaryota>metazoa>chordata>vertebrata>actinopterygii	Ictalurus	punctatus	PREDICTED: coiled-coil domain-containing protein 129 isoform X1 [Ictalurus punctatus]
XP_017346752.1	936	ccdc129	eukaryota>metazoa>chordata>vertebrata>actinopterygii	Ictalurus	punctatus	PREDICTED: coiled-coil domain-containing protein 129 isoform X1 [Ictalurus punctatus]
XP_017346831.1	936	ccdc129	eukaryota>metazoa>chordata>vertebrata>actinopterygii	Ictalurus	punctatus	PREDICTED: coiled-coil domain-containing protein 129 isoform X1 [Ictalurus punctatus]
XP_017346891.1	917	ccdc129	eukaryota>metazoa>chordata>vertebrata>actinopterygii	Ictalurus	punctatus	PREDICTED: coiled-coil domain-containing protein 129 isoform X2 [Ictalurus punctatus]
XP_018123071.1	911	LOC108719001	eukaryota>metazoa>chordata>vertebrata	Xenopus	laevis	PREDICTED: coiled-coil domain-containing protein 129-like isoform X2 [Xenopus laevis]
XP_002666731.1	874	ccdc129	eukaryota>metazoa>chordata>vertebrata>actinopterygii	Danio	rerio	coiled-coil domain-containing protein 129 [Danio rerio]
XP_005162917.1	874	ccdc129	eukaryota>metazoa>chordata>vertebrata>actinopterygii	Danio	rerio	coiled-coil domain-containing protein 129 [Danio rerio]
XP_017346965.1	862	ccdc129	eukaryota>metazoa>chordata>vertebrata>actinopterygii	Ictalurus	punctatus	PREDICTED: coiled-coil domain-containing protein 129 isoform X3 [Ictalurus punctatus]
KXJ14117.1	851	AC249_AIPGENE25239	eukaryota>metazoa>cnidaria	Exaiptasia	pallida	hypothetical protein AC249_AIPGENE25239 [Exaiptasia pallida]
XP_022241796.1	850	LOC106459580	eukaryota>metazoa	Limulus	polyphemus	uncharacterized protein LOC106459580 isoform X1 [Limulus polyphemus]
XP_018123073.1	829	LOC108719001	eukaryota>metazoa>chordata>vertebrata	Xenopus	laevis	PREDICTED: coiled-coil domain-containing protein 129-like isoform X3 [Xenopus laevis]
XP_017347047.1	806	ccdc129	eukaryota>metazoa>chordata>vertebrata>actinopterygii	Ictalurus	punctatus	PREDICTED: coiled-coil domain-containing protein 129 isoform X4 [Ictalurus punctatus]
XP_022241797.1	733	LOC106459580	eukaryota>metazoa	Limulus	polyphemus	uncharacterized protein LOC106459580 isoform X2 [Limulus polyphemus]
XP_012562630.1	704	LOC100200540	eukaryota>metazoa>cnidaria	Hydra	vulgaris	PREDICTED: uncharacterized protein LOC100200540, partial [Hydra vulgaris]
XP_017335692.1	689	tespa1	eukaryota>metazoa>chordata>vertebrata>actinopterygii	Ictalurus	punctatus	PREDICTED: protein TESPA1 isoform X1 [Ictalurus punctatus]
XP_017335693.1	688	tespa1	eukaryota>metazoa>chordata>vertebrata>actinopterygii	Ictalurus	punctatus	PREDICTED: protein TESPA1 isoform X2 [Ictalurus punctatus]
XP_019696346.1	652	LOC105181887	eukaryota>metazoa>hexapoda	Harpegnathos	saltator	PREDICTED: uncharacterized protein LOC105181887 isoform X1 [Harpegnathos saltator]
XP_011137256.1	644	LOC105181887	eukaryota>metazoa>hexapoda	Harpegnathos	saltator	uncharacterized protein LOC105181887 isoform X1 [Harpegnathos saltator]
XP_008185170.1	630	LOC100165562	eukaryota>metazoa>hexapoda	Acyrthosiphon	pisum	PREDICTED: uncharacterized protein LOC100165562 [Acyrthosiphon pisum]
XP_016661642.1	630	LOC100165562	eukaryota>metazoa>hexapoda	Acyrthosiphon	pisum	PREDICTED: uncharacterized protein LOC100165562 [Acyrthosiphon pisum]
XP_011137257.1	621	LOC105181887	eukaryota>metazoa>hexapoda	Harpegnathos	saltator	uncharacterized protein LOC105181887 isoform X2 [Harpegnathos saltator]
XP_006567531.1	614	LOC725634	eukaryota>metazoa>hexapoda	Apis	mellifera	PREDICTED: uncharacterized protein LOC725634 isoform X1 [Apis mellifera]
XP_016769437.1	614	LOC725634	eukaryota>metazoa>hexapoda	Apis	mellifera	PREDICTED: uncharacterized protein LOC725634 isoform X1 [Apis mellifera]
EFX74588.1	598	DAPPUDRAFT_307233	eukaryota>metazoa>crustacea	Daphnia	pulex	hypothetical protein DAPPUDRAFT_307233 [Daphnia pulex]
XP_001121463.2	591	LOC725634	eukaryota>metazoa>hexapoda	Apis	mellifera	PREDICTED: uncharacterized protein LOC725634 isoform X2 [Apis mellifera]
XP_002662570.3	585	tespa1	eukaryota>metazoa>chordata>vertebrata>actinopterygii	Danio	rerio	protein TESPA1 [Danio rerio]
XP_015155734.1	583	TESPA1	eukaryota>metazoa>chordata>vertebrata	Gallus	gallus	PREDICTED: protein TESPA1 isoform X1 [Gallus gallus]
XP_001846333.1	566	CpipJ_CPIJ004569	eukaryota>metazoa>hexapoda	Culex	quinquefasciatus	conserved hypothetical protein [Culex quinquefasciatus]
XP_011612430.1	557	tespa1	eukaryota>metazoa>chordata>vertebrata>actinopterygii	Takifugu	rubripes	PREDICTED: protein TESPA1 [Takifugu rubripes]
XP_015155735.1	553	TESPA1	eukaryota>metazoa>chordata>vertebrata	Gallus	gallus	PREDICTED: protein TESPA1 isoform X2 [Gallus gallus]
XP_009054090.1	541	LOTGIDRAFT_175217	eukaryota>metazoa>mollusca	Lottia	gigantea	hypothetical protein LOTGIDRAFT_175217 [Lottia gigantea]
XP_015286476.1	527	TESPA1	eukaryota>metazoa>chordata>vertebrata	Macaca	fascicularis	PREDICTED: protein TESPA1 isoform X1 [Macaca fascicularis]
XP_015286477.1	527	TESPA1	eukaryota>metazoa>chordata>vertebrata	Macaca	fascicularis	PREDICTED: protein TESPA1 isoform X1 [Macaca fascicularis]
NP_001092285.1	521	HSPC257,ITPRID3,KIAA0748	eukaryota>metazoa>chordata>vertebrata	Homo	sapiens	protein TESPA1 isoform 1 [Homo sapiens]
NP_001129502.1	521	HSPC257,ITPRID3,KIAA0748	eukaryota>metazoa>chordata>vertebrata	Homo	sapiens	protein TESPA1 isoform 1 [Homo sapiens]
XP_003252911.1	521	TESPA1	eukaryota>metazoa>chordata>vertebrata	Nomascus	leucogenys	PREDICTED: protein TESPA1 isoform X1 [Nomascus leucogenys]
XP_006719778.1	521	HSPC257,KIAA0748	eukaryota>metazoa>chordata>vertebrata	Homo	sapiens	protein TESPA1 isoform X1 [Homo sapiens]
XP_011537337.1	521	HSPC257,KIAA0748	eukaryota>metazoa>chordata>vertebrata	Homo	sapiens	protein TESPA1 isoform X1 [Homo sapiens]
XP_011537338.1	521	HSPC257,KIAA0748	eukaryota>metazoa>chordata>vertebrata	Homo	sapiens	PREDICTED: protein TESPA1 isoform X1 [Homo sapiens]
XP_011537339.1	521	HSPC257,KIAA0748	eukaryota>metazoa>chordata>vertebrata	Homo	sapiens	protein TESPA1 isoform X1 [Homo sapiens]
XP_012365671.1	521	TESPA1	eukaryota>metazoa>chordata>vertebrata	Nomascus	leucogenys	PREDICTED: protein TESPA1 isoform X1 [Nomascus leucogenys]
XP_015286478.1	521	TESPA1	eukaryota>metazoa>chordata>vertebrata	Macaca	fascicularis	PREDICTED: protein TESPA1 isoform X2 [Macaca fascicularis]
XP_015286479.1	521	TESPA1	eukaryota>metazoa>chordata>vertebrata	Macaca	fascicularis	PREDICTED: protein TESPA1 isoform X2 [Macaca fascicularis]
XP_016875751.1	521	HSPC257,KIAA0748	eukaryota>metazoa>chordata>vertebrata	Homo	sapiens	protein TESPA1 isoform X1 [Homo sapiens]
XP_016875752.1	521	HSPC257,KIAA0748	eukaryota>metazoa>chordata>vertebrata	Homo	sapiens	protein TESPA1 isoform X1 [Homo sapiens]
XP_014786361.1	480	LOC106880779	eukaryota>metazoa>mollusca	Octopus	bimaculoides	PREDICTED: uncharacterized protein LOC106880779 [Octopus bimaculoides]
XP_002125295.1	464	LOC100187085	eukaryota>metazoa>chordata	Ciona	intestinalis	PREDICTED: uncharacterized protein LOC100187085 [Ciona intestinalis]
NP_899087.2	458	5830405N20Rik,A430001F24Rik,AI450549	eukaryota>metazoa>chordata>vertebrata	Mus	musculus	protein TESPA1 [Mus musculus]
XP_006514069.1	458	5830405N20Rik,A430001F24Rik,AI450549	eukaryota>metazoa>chordata>vertebrata	Mus	musculus	PREDICTED: protein TESPA1 isoform X1 [Mus musculus]
NP_001248773.1	383	HSPC257,ITPRID3,KIAA0748	eukaryota>metazoa>chordata>vertebrata	Homo	sapiens	protein TESPA1 isoform 2 [Homo sapiens]
NP_055611.1	383	HSPC257,ITPRID3,KIAA0748	eukaryota>metazoa>chordata>vertebrata	Homo	sapiens	protein TESPA1 isoform 2 [Homo sapiens]
XP_004089667.1	383	TESPA1	eukaryota>metazoa>chordata>vertebrata	Nomascus	leucogenys	PREDICTED: protein TESPA1 isoform X2 [Nomascus leucogenys]
XP_005269304.1	383	HSPC257,KIAA0748	eukaryota>metazoa>chordata>vertebrata	Homo	sapiens	protein TESPA1 isoform X2 [Homo sapiens]
XP_015129428.1	362	LOC101749979	eukaryota>metazoa>chordata>vertebrata	Gallus	gallus	PREDICTED: protein TESPA1-like, partial [Gallus gallus]
XP_021207255.1	289	LOC101741084	eukaryota>metazoa>hexapoda	Bombyx	mori	uncharacterized protein LOC101741084 isoform X1 [Bombyx mori]
XP_018105610.1	273	LOC108709888	eukaryota>metazoa>chordata>vertebrata	Xenopus	laevis	PREDICTED: protein TESPA1-like isoform X1 [Xenopus laevis]
XP_021207256.1	273	LOC101741084	eukaryota>metazoa>hexapoda	Bombyx	mori	uncharacterized protein LOC101741084 isoform X2 [Bombyx mori]
XP_004931503.1	259	LOC101741084	eukaryota>metazoa>hexapoda	Bombyx	mori	uncharacterized protein LOC101741084 isoform X3 [Bombyx mori]
NP_611272.1	254	CG14489,Dmel\CG14489	eukaryota>metazoa>hexapoda	Drosophila	melanogaster	olf186-M [Drosophila melanogaster]
XP_018101040.1	231	LOC108707555	eukaryota>metazoa>chordata>vertebrata	Xenopus	laevis	PREDICTED: protein TESPA1-like [Xenopus laevis]
XP_002412678.1	196	IscW_ISCW022006	eukaryota>metazoa	Ixodes	scapularis	hypothetical protein IscW_ISCW022006 [Ixodes scapularis]
XP_008191206.1	138	LOC655754	eukaryota>metazoa>hexapoda	Tribolium	castaneum	PREDICTED: uncharacterized protein LOC655754 isoform X1 [Tribolium castaneum]
XP_008191207.1	134	LOC655754	eukaryota>metazoa>hexapoda	Tribolium	castaneum	PREDICTED: uncharacterized protein LOC655754 isoform X2 [Tribolium castaneum]
XP_967409.1	132	LOC655754	eukaryota>metazoa>hexapoda	Tribolium	castaneum	PREDICTED: uncharacterized protein LOC655754 isoform X3 [Tribolium castaneum]
XP_008191208.1	128	LOC655754	eukaryota>metazoa>hexapoda	Tribolium	castaneum	PREDICTED: uncharacterized protein LOC655754 isoform X4 [Tribolium castaneum]
CAF93685.1	75	GSTEN:00009447:G:001	eukaryota>metazoa>chordata>vertebrata>actinopterygii	Tetraodon	nigroviridis	unnamed protein product, partial [Tetraodon nigroviridis]
CAF90925.1	25	GSTEN:00005393:G:001	eukaryota>metazoa>chordata>vertebrata>actinopterygii	Tetraodon	nigroviridis	unnamed protein product, partial [Tetraodon nigroviridis]

AG. Calsequestrin

CAF97694.1	821	GSTEN:00015131:G:001	eukaryota>metazoa>chordata>vertebrata>actinopterygii	Tetraodon	nigroviridis	unnamed protein product [Tetraodon nigroviridis]
XP_002604497.1	555	BRAFLDRAFT_122314	eukaryota>metazoa>chordata	Branchiostoma	floridae	hypothetical protein BRAFLDRAFT_122314 [Branchiostoma floridae]
XP_015129971.1	554	LOC107050773	eukaryota>metazoa>chordata>vertebrata	Gallus	gallus	PREDICTED: uncharacterized protein LOC107050773, partial [Gallus gallus]
NP_001003620.2	553	bZ1G18.2,zgc:100957	eukaryota>metazoa>chordata>vertebrata>actinopterygii	Danio	rerio	calsequestrin 1a precursor [Danio rerio]
XP_006812181.1	531	LOC102803517	eukaryota>metazoa>hemichordata	Saccoglossus	kowalevskii	PREDICTED: calsequestrin-1-like [Saccoglossus kowalevskii]
CAG05875.1	518	GSTEN:00026039:G:001	eukaryota>metazoa>chordata>vertebrata>actinopterygii	Tetraodon	nigroviridis	unnamed protein product, partial [Tetraodon nigroviridis]
XP_003267900.3	513	CASQ2	eukaryota>metazoa>chordata>vertebrata	Nomascus	leucogenys	PREDICTED: calsequestrin-2 [Nomascus leucogenys]
XP_003977884.1	464	casq1	eukaryota>metazoa>chordata>vertebrata>actinopterygii	Takifugu	rubripes	PREDICTED: calsequestrin-1 [Takifugu rubripes]
XP_012366344.1	459	CASQ1	eukaryota>metazoa>chordata>vertebrata	Nomascus	leucogenys	PREDICTED: calsequestrin-1 [Nomascus leucogenys]
XP_005166685.1	454	zgc:154027	eukaryota>metazoa>chordata>vertebrata>actinopterygii	Danio	rerio	calsequestrin-1 isoform X1 [Danio rerio]
NP_001070192.1	443	zgc:154027	eukaryota>metazoa>chordata>vertebrata>actinopterygii	Danio	rerio	calsequestrin-1 precursor [Danio rerio]
XP_003961863.1	441	LOC101061703	eukaryota>metazoa>chordata>vertebrata>actinopterygii	Takifugu	rubripes	PREDICTED: calsequestrin-2-like [Takifugu rubripes]
XP_011607264.1	441	LOC101061703	eukaryota>metazoa>chordata>vertebrata>actinopterygii	Takifugu	rubripes	PREDICTED: calsequestrin-2-like [Takifugu rubripes]
XP_002130664.1	436	LOC100187204	eukaryota>metazoa>chordata	Ciona	intestinalis	PREDICTED: calsequestrin-2 [Ciona intestinalis]
XP_003378010.1	435	Tsp_02205	eukaryota>metazoa>nematoda	Trichinella	spiralis	putative calsequestrin-1 [Trichinella spiralis]
XP_006501010.1	432	AA033488,AW146219,cCSQ,Csq2,ESTM52	eukaryota>metazoa>chordata>vertebrata	Mus	musculus	PREDICTED: calsequestrin-2 isoform X1 [Mus musculus]
XP_003977756.1	431	LOC101077453	eukaryota>metazoa>chordata>vertebrata>actinopterygii	Takifugu	rubripes	PREDICTED: calsequestrin-1-like [Takifugu rubripes]
XP_017326378.1	429	LOC108267017	eukaryota>metazoa>chordata>vertebrata>actinopterygii	Ictalurus	punctatus	PREDICTED: calsequestrin-2-like [Ictalurus punctatus]
XP_017326379.1	429	LOC108267017	eukaryota>metazoa>chordata>vertebrata>actinopterygii	Ictalurus	punctatus	PREDICTED: calsequestrin-2-like [Ictalurus punctatus]
XP_017326380.1	429	LOC108267017	eukaryota>metazoa>chordata>vertebrata>actinopterygii	Ictalurus	punctatus	PREDICTED: calsequestrin-2-like [Ictalurus punctatus]
XP_018087000.1	429	LOC108699432	eukaryota>metazoa>chordata>vertebrata	Xenopus	laevis	PREDICTED: calsequestrin-1 [Xenopus laevis]
NP_001080227.1	428	asph,casq,casq1,casq1.L,pdib1	eukaryota>metazoa>chordata>vertebrata	Xenopus	laevis	calsequestrin 1 S homeolog precursor [Xenopus laevis]
XP_017317161.1	422	LOC108260939	eukaryota>metazoa>chordata>vertebrata>actinopterygii	Ictalurus	punctatus	PREDICTED: calsequestrin-1-like isoform X1 [Ictalurus punctatus]
ERG84285.1	421	ASU_05627	eukaryota>metazoa>nematoda	Ascaris	suum	calsequestrin-1 [Ascaris suum]
XP_001899493.1	419	Bm1_40185	eukaryota>metazoa>nematoda	Brugia	malayi	Calsequestrin family protein [Brugia malayi]
NP_510438.1	417	csq-1	eukaryota>metazoa>nematoda	Caenorhabditis	elegans	Calsequestrin [Caenorhabditis elegans]
NP_033944.2	415	AA033488,AW146219,cCSQ,Csq2,ESTM52	eukaryota>metazoa>chordata>vertebrata	Mus	musculus	calsequestrin-2 precursor [Mus musculus]
XP_017312387.1	414	LOC108258342	eukaryota>metazoa>chordata>vertebrata>actinopterygii	Ictalurus	punctatus	PREDICTED: calsequestrin-2-like [Ictalurus punctatus]
XP_018104514.1	412	casq2.L	eukaryota>metazoa>chordata>vertebrata	Xenopus	laevis	PREDICTED: calsequestrin-2 isoform X1 [Xenopus laevis]
XP_017317162.1	411	LOC108260939	eukaryota>metazoa>chordata>vertebrata>actinopterygii	Ictalurus	punctatus	PREDICTED: calsequestrin-1-like isoform X2 [Ictalurus punctatus]
NP_001002682.1	409	wu:fa02h03,zgc:86817	eukaryota>metazoa>chordata>vertebrata>actinopterygii	Danio	rerio	calsequestrin-2 precursor [Danio rerio]
XP_003966504.1	409	LOC101070433	eukaryota>metazoa>chordata>vertebrata>actinopterygii	Takifugu	rubripes	PREDICTED: calsequestrin-2-like [Takifugu rubripes]
CAG00977.1	407	GSTEN:00019584:G:001	eukaryota>metazoa>chordata>vertebrata>actinopterygii	Tetraodon	nigroviridis	unnamed protein product, partial [Tetraodon nigroviridis]
KXJ29675.1	406	CASQ1	eukaryota>metazoa>cnidaria	Exaiptasia	pallida	Calsequestrin-1 [Exaiptasia pallida]
NP_989857.1	406	calsequestrin-2,CASQ1	eukaryota>metazoa>chordata>vertebrata	Gallus	gallus	calsequestrin-2 precursor [Gallus gallus]
NP_033943.2	405	CSQ,CSQ-1,CSQ1,sCSQ	eukaryota>metazoa>chordata>vertebrata	Mus	musculus	calsequestrin-1 precursor [Mus musculus]
XP_013792906.1	405	LOC106476831	eukaryota>metazoa	Limulus	polyphemus	PREDICTED: calsequestrin-1-like [Limulus polyphemus]
CAF97003.1	403	GSTEN:00014242:G:001	eukaryota>metazoa>chordata>vertebrata>actinopterygii	Tetraodon	nigroviridis	unnamed protein product, partial [Tetraodon nigroviridis]
XP_002161594.3	402	LOC100205300	eukaryota>metazoa>cnidaria	Hydra	vulgaris	PREDICTED: calsequestrin-2-like [Hydra vulgaris]
XP_005542276.1	400	CASQ2	eukaryota>metazoa>chordata>vertebrata	Macaca	fascicularis	PREDICTED: calsequestrin-2 [Macaca fascicularis]
NP_001223.2	399	PDIB2	eukaryota>metazoa>chordata>vertebrata	Homo	sapiens	calsequestrin-2 precursor [Homo sapiens]
XP_005541326.1	398	CASQ1	eukaryota>metazoa>chordata>vertebrata	Macaca	fascicularis	PREDICTED: calsequestrin-1 [Macaca fascicularis]
NP_001222.3	396	CASQ,PDIB1,VMCQA	eukaryota>metazoa>chordata>vertebrata	Homo	sapiens	calsequestrin-1 precursor [Homo sapiens]
CBY13690.1	394	GSOID_T00005502001	eukaryota>metazoa>chordata	Oikopleura	dioica	unnamed protein product, partial [Oikopleura dioica]
XP_017212403.1	374	zgc:154027	eukaryota>metazoa>chordata>vertebrata>actinopterygii	Danio	rerio	PREDICTED: calsequestrin-1 isoform X2 [Danio rerio]
XP_002111704.1	373	TRIADDRAFT_56011	eukaryota>metazoa>placozoa	Trichoplax	adhaerens	hypothetical protein TRIADDRAFT_56011 [Trichoplax adhaerens]
XP_001623501.1	372	NEMVEDRAFT_v1g219307	eukaryota>metazoa>cnidaria	Nematostella	vectensis	predicted protein [Nematostella vectensis]
KXJ06619.1	354	CASQ1	eukaryota>metazoa>cnidaria	Exaiptasia	pallida	Calsequestrin-1, partial [Exaiptasia pallida]
XP_015769018.1	350	LOC107347569	eukaryota>metazoa>cnidaria	Acropora	digitifera	PREDICTED: calsequestrin-1-like [Acropora digitifera]
CBY12414.1	323	GSOID_T00001783001	eukaryota>metazoa>chordata	Oikopleura	dioica	unnamed protein product [Oikopleura dioica]
XP_018104515.1	284	casq2.L	eukaryota>metazoa>chordata>vertebrata	Xenopus	laevis	PREDICTED: calsequestrin-2 isoform X2 [Xenopus laevis]
CBY15484.1	59	GSOID_T00013762001	eukaryota>metazoa>chordata	Oikopleura	dioica	unnamed protein product, partial [Oikopleura dioica]

S100

XP_017175331.1	4384	Flg	eukaryota>metazoa>chordata>vertebrata	Mus	musculus	PREDICTED: LOW QUALITY PROTEIN: filaggrin [Mus musculus]
XP_006502049.1	3411	1110033K19Rik,A530063N20Rik,S100a18	eukaryota>metazoa>chordata>vertebrata	Mus	musculus	PREDICTED: hornerin isoform X1 [Mus musculus]
NP_598459.2	3234	1110033K19Rik,A530063N20Rik,S100a18	eukaryota>metazoa>chordata>vertebrata	Mus	musculus	hornerin [Mus musculus]
XP_006502050.1	3234	1110033K19Rik,A530063N20Rik,S100a18	eukaryota>metazoa>chordata>vertebrata	Mus	musculus	PREDICTED: hornerin isoform X2 [Mus musculus]
XP_015312597.1	3046	FLG2	eukaryota>metazoa>chordata>vertebrata	Macaca	fascicularis	PREDICTED: filaggrin-2 [Macaca fascicularis]
NP_001009931.1	2850	FLG3,S100A16,S100a18	eukaryota>metazoa>chordata>vertebrata	Homo	sapiens	hornerin [Homo sapiens]
XP_003259308.1	2827	HRNR	eukaryota>metazoa>chordata>vertebrata	Nomascus	leucogenys	PREDICTED: hornerin [Nomascus leucogenys]
XP_005541890.1	2744	HRNR	eukaryota>metazoa>chordata>vertebrata	Macaca	fascicularis	PREDICTED: hornerin [Macaca fascicularis]
XP_005595167.1	2437	LOC102138001	eukaryota>metazoa>chordata>vertebrata	Macaca	fascicularis	PREDICTED: filaggrin-like [Macaca fascicularis]
NP_001014364.1	2391	IFPS	eukaryota>metazoa>chordata>vertebrata	Homo	sapiens	filaggrin-2 [Homo sapiens]
NP_001013826.1	2362	EG229574	eukaryota>metazoa>chordata>vertebrata	Mus	musculus	filaggrin-2 [Mus musculus]
XP_011507833.1	2239	IFPS	eukaryota>metazoa>chordata>vertebrata	Homo	sapiens	PREDICTED: filaggrin-2 isoform X1 [Homo sapiens]
XP_012366375.1	2011	FLG2	eukaryota>metazoa>chordata>vertebrata	Nomascus	leucogenys	PREDICTED: filaggrin-2 [Nomascus leucogenys]
NP_009044.2	1943	THH,THL,TRHY,UHS3	eukaryota>metazoa>chordata>vertebrata	Homo	sapiens	trichohyalin [Homo sapiens]
XP_005541893.1	1817	TCHH	eukaryota>metazoa>chordata>vertebrata	Macaca	fascicularis	PREDICTED: LOW QUALITY PROTEIN: trichohyalin [Macaca fascicularis]
NP_001156570.1	1599	AHF,AI597080,Thh	eukaryota>metazoa>chordata>vertebrata	Mus	musculus	trichohyalin [Mus musculus]
XP_015312612.1	1160	RPTN	eukaryota>metazoa>chordata>vertebrata	Macaca	fascicularis	PREDICTED: repetin [Macaca fascicularis]
NP_033126.2	1118	Rptn	eukaryota>metazoa>chordata>vertebrata	Mus	musculus	repetin [Mus musculus]
NP_001008536.1	904	S100A17,THHL1	eukaryota>metazoa>chordata>vertebrata	Homo	sapiens	trichohyalin-like protein 1 [Homo sapiens]
XP_003259307.1	904	TCHHL1	eukaryota>metazoa>chordata>vertebrata	Nomascus	leucogenys	PREDICTED: trichohyalin-like protein 1 [Nomascus leucogenys]
XP_005541894.1	901	TCHHL1	eukaryota>metazoa>chordata>vertebrata	Macaca	fascicularis	PREDICTED: trichohyalin-like protein 1 [Macaca fascicularis]
XP_012367057.1	798	RPTN	eukaryota>metazoa>chordata>vertebrata	Nomascus	leucogenys	PREDICTED: repetin [Nomascus leucogenys]
NP_001116437.1	784	RPTN	eukaryota>metazoa>chordata>vertebrata	Homo	sapiens	repetin [Homo sapiens]
XP_012366376.1	645	TCHH	eukaryota>metazoa>chordata>vertebrata	Nomascus	leucogenys	PREDICTED: LOW QUALITY PROTEIN: trichohyalin [Nomascus leucogenys]
NP_082038.2	638	5430400H23Rik,AA589420,S100a17,Thhl1	eukaryota>metazoa>chordata>vertebrata	Mus	musculus	trichohyalin-like protein 1 [Mus musculus]
XP_015153863.1	597	LOC101751678	eukaryota>metazoa>chordata>vertebrata	Gallus	gallus	PREDICTED: trichohyalin-like, partial [Gallus gallus]
NP_001074669.1	520	Gm1015	eukaryota>metazoa>chordata>vertebrata	Mus	musculus	cornulin [Mus musculus]
XP_015312592.1	510	CRNN	eukaryota>metazoa>chordata>vertebrata	Macaca	fascicularis	PREDICTED: cornulin [Macaca fascicularis]
NP_057274.1	495	C1orf10,DRC1,PDRC1,SEP53	eukaryota>metazoa>chordata>vertebrata	Homo	sapiens	cornulin [Homo sapiens]
XP_003259309.1	495	CRNN	eukaryota>metazoa>chordata>vertebrata	Nomascus	leucogenys	PREDICTED: cornulin [Nomascus leucogenys]
XP_018087213.1	480	LOC108699634	eukaryota>metazoa>chordata>vertebrata	Xenopus	laevis	PREDICTED: RNA polymerase II degradation factor 1-like isoform X1 [Xenopus laevis]
XP_018087214.1	444	LOC108699634	eukaryota>metazoa>chordata>vertebrata	Xenopus	laevis	PREDICTED: RNA polymerase II degradation factor 1-like isoform X2 [Xenopus laevis]
XP_018087215.1	430	LOC108699634	eukaryota>metazoa>chordata>vertebrata	Xenopus	laevis	PREDICTED: RNA polymerase II degradation factor 1-like isoform X3 [Xenopus laevis]
XP_015153853.1	414	CRNN	eukaryota>metazoa>chordata>vertebrata	Gallus	gallus	PREDICTED: cornulin isoform X1 [Gallus gallus]
NP_001165847.1	384	CRNN	eukaryota>metazoa>chordata>vertebrata	Gallus	gallus	cornulin [Gallus gallus]
XP_003966267.2	289	LOC101068529	eukaryota>metazoa>chordata>vertebrata>actinopterygii	Takifugu	rubripes	PREDICTED: uncharacterized protein LOC101068529 [Takifugu rubripes]
XP_018089300.1	285	LOC108700619	eukaryota>metazoa>chordata>vertebrata	Xenopus	laevis	PREDICTED: cornulin-like [Xenopus laevis]
NP_001121813.1	240	fc27g02,si:dkey-105n5.2,wu:fc27g02	eukaryota>metazoa>chordata>vertebrata>actinopterygii	Danio	rerio	S100 calcium binding protein U [Danio rerio]
XP_012351626.1	234	LOC100594672	eukaryota>metazoa>chordata>vertebrata	Nomascus	leucogenys	PREDICTED: LOW QUALITY PROTEIN: uncharacterized protein LOC100594672 [Nomascus leucogenys]
XP_003969335.1	230	LOC101064556	eukaryota>metazoa>chordata>vertebrata>actinopterygii	Takifugu	rubripes	PREDICTED: coiled-coil domain-containing protein 8 homolog [Takifugu rubripes]
NP_001085255.1	218	dicalcin,mln70,p26olf,s100a11,s100c	eukaryota>metazoa>chordata>vertebrata	Xenopus	laevis	S100 calcium binding protein A11 L homeolog [Xenopus laevis]
XP_018089079.1	218	LOC108700422	eukaryota>metazoa>chordata>vertebrata	Xenopus	laevis	PREDICTED: uncharacterized protein LOC108700422 [Xenopus laevis]
XP_006501694.1	204	AY465109,Gm1020,S100a15,S100a15a,S100a17l1,S100A7f,S100A7L1	eukaryota>metazoa>chordata>vertebrata	Mus	musculus	PREDICTED: protein S100-A15A isoform X1 [Mus musculus]
XP_005545229.2	200	S100A7L2	eukaryota>metazoa>chordata>vertebrata	Macaca	fascicularis	PREDICTED: protein S100-A7-like 2 [Macaca fascicularis]
XP_017337364.1	171	LOC108272970	eukaryota>metazoa>chordata>vertebrata>actinopterygii	Ictalurus	punctatus	PREDICTED: protein S100-A1-like isoform X1 [Ictalurus punctatus]
XP_428126.5	167	S100A13	eukaryota>metazoa>chordata>vertebrata	Gallus	gallus	PREDICTED: protein S100-A13 [Gallus gallus]
XP_012366958.1	166	S100A8	eukaryota>metazoa>chordata>vertebrata	Nomascus	leucogenys	PREDICTED: protein S100-A8 isoform X1 [Nomascus leucogenys]
NP_033139.2	160	S100a13	eukaryota>metazoa>chordata>vertebrata	Mus	musculus	protein S100-A13 [Mus musculus]
XP_011238348.1	160	S100a13	eukaryota>metazoa>chordata>vertebrata	Mus	musculus	PREDICTED: protein S100-A13 isoform X1 [Mus musculus]
XP_017337367.1	152	LOC108272971	eukaryota>metazoa>chordata>vertebrata>actinopterygii	Ictalurus	punctatus	PREDICTED: protein S100-A16-like [Ictalurus punctatus]
XP_017174997.1	151	42C,AA409961,AL024248,CAL12,Cal1l,CLP11,p10,p11	eukaryota>metazoa>chordata>vertebrata	Mus	musculus	PREDICTED: protein S100-A10 isoform X1 [Mus musculus]
XP_005541839.2	150	LOC102120158	eukaryota>metazoa>chordata>vertebrata	Macaca	fascicularis	PREDICTED: protein S100-A15A isoform X1 [Macaca fascicularis]
NP_001074006.1	147	S100A1L,S100AL,sentan	eukaryota>metazoa>chordata>vertebrata	Homo	sapiens	sentan isoform 2 [Homo sapiens]
NP_808292.1	147	A430083B19Rik	eukaryota>metazoa>chordata>vertebrata	Mus	musculus	sentan [Mus musculus]
XP_003273704.1	147	SNTN	eukaryota>metazoa>chordata>vertebrata	Nomascus	leucogenys	PREDICTED: sentan [Nomascus leucogenys]
XP_003282255.2	147	S100A10	eukaryota>metazoa>chordata>vertebrata	Nomascus	leucogenys	PREDICTED: protein S100-A10 isoform X1 [Nomascus leucogenys]
XP_005547583.1	147	SNTN	eukaryota>metazoa>chordata>vertebrata	Macaca	fascicularis	PREDICTED: LOW QUALITY PROTEIN: sentan [Macaca fascicularis]
XP_017337365.1	145	LOC108272970	eukaryota>metazoa>chordata>vertebrata>actinopterygii	Ictalurus	punctatus	PREDICTED: protein S100-A1-like isoform X2 [Ictalurus punctatus]
NP_001136319.1	144	S100A1L	eukaryota>metazoa>chordata>vertebrata	Gallus	gallus	sentan [Gallus gallus]
XP_005541841.1	140	LOC102120794	eukaryota>metazoa>chordata>vertebrata	Macaca	fascicularis	PREDICTED: protein S100-A7 isoform X2 [Macaca fascicularis]
XP_003965434.2	138	s100z	eukaryota>metazoa>chordata>vertebrata>actinopterygii	Takifugu	rubripes	PREDICTED: protein S100-Z [Takifugu rubripes]
XP_018084073.1	137	42c,anx2l,anx2lg,cal1l,calpactin,clp11,gp11,p11-A,s100a10,s100a10-a,s100a10-b,s100a10b	eukaryota>metazoa>chordata>vertebrata	Xenopus	laevis	PREDICTED: protein S100-A10 isoform X1 [Xenopus laevis]
XP_018084074.1	136	42c,anx2l,anx2lg,cal1l,calpactin,clp11,gp11,p11-A,s100a10,s100a10-a,s100a10-b,s100a10b	eukaryota>metazoa>chordata>vertebrata	Xenopus	laevis	PREDICTED: protein S100-A10 isoform X2 [Xenopus laevis]
XP_011239117.1	135	Gm40318	eukaryota>metazoa>chordata>vertebrata	Mus	musculus	PREDICTED: LOW QUALITY PROTEIN: protein S100-P [Mus musculus]
XP_016857518.1	133	S100D	eukaryota>metazoa>chordata>vertebrata	Homo	sapiens	PREDICTED: protein S100-A5 isoform X1 [Homo sapiens]
XP_004092679.1	128	S100A10	eukaryota>metazoa>chordata>vertebrata	Nomascus	leucogenys	PREDICTED: protein S100-A10 isoform X2 [Nomascus leucogenys]
XP_005541899.1	128	S100A10	eukaryota>metazoa>chordata>vertebrata	Macaca	fascicularis	PREDICTED: protein S100-A10 isoform X1 [Macaca fascicularis]
XP_017336245.1	126	LOC100305037	eukaryota>metazoa>chordata>vertebrata>actinopterygii	Ictalurus	punctatus	PREDICTED: ictacalcin isoform X1 [Ictalurus punctatus]
XP_017336842.1	125	LOC108272706	eukaryota>metazoa>chordata>vertebrata>actinopterygii	Ictalurus	punctatus	PREDICTED: protein S100-A1 [Ictalurus punctatus]
XP_017336854.1	125	LOC108272712	eukaryota>metazoa>chordata>vertebrata>actinopterygii	Ictalurus	punctatus	PREDICTED: protein S100-A1-like [Ictalurus punctatus]
XP_017337366.1	125	LOC108272970	eukaryota>metazoa>chordata>vertebrata>actinopterygii	Ictalurus	punctatus	PREDICTED: protein S100-A1-like isoform X3 [Ictalurus punctatus]
NP_080692.1	124	2300002L21Rik,AI325039,AI663996,DT1P1A7,S100F	eukaryota>metazoa>chordata>vertebrata	Mus	musculus	protein S100-A16 [Mus musculus]
XP_006502011.1	124	2300002L21Rik,AI325039,AI663996,DT1P1A7,S100F	eukaryota>metazoa>chordata>vertebrata	Mus	musculus	PREDICTED: protein S100-A16 isoform X1 [Mus musculus]
XP_006502012.1	124	2300002L21Rik,AI325039,AI663996,DT1P1A7,S100F	eukaryota>metazoa>chordata>vertebrata	Mus	musculus	PREDICTED: protein S100-A16 isoform X1 [Mus musculus]
XP_006502013.1	124	2300002L21Rik,AI325039,AI663996,DT1P1A7,S100F	eukaryota>metazoa>chordata>vertebrata	Mus	musculus	PREDICTED: protein S100-A16 isoform X1 [Mus musculus]
XP_006502014.1	124	2300002L21Rik,AI325039,AI663996,DT1P1A7,S100F	eukaryota>metazoa>chordata>vertebrata	Mus	musculus	PREDICTED: protein S100-A16 isoform X1 [Mus musculus]
XP_006502015.1	124	2300002L21Rik,AI325039,AI663996,DT1P1A7,S100F	eukaryota>metazoa>chordata>vertebrata	Mus	musculus	PREDICTED: protein S100-A16 isoform X1 [Mus musculus]
XP_005159484.1	122	zgc:91995	eukaryota>metazoa>chordata>vertebrata>actinopterygii	Danio	rerio	S100 calcium binding protein S isoform X1 [Danio rerio]
XP_009292314.1	122	zgc:91995	eukaryota>metazoa>chordata>vertebrata>actinopterygii	Danio	rerio	PREDICTED: S100 calcium binding protein S isoform X1 [Danio rerio]
XP_012366969.1	121	S100A3	eukaryota>metazoa>chordata>vertebrata	Nomascus	leucogenys	PREDICTED: protein S100-A3 isoform X1 [Nomascus leucogenys]
XP_004948278.1	120	LOC101747386	eukaryota>metazoa>chordata>vertebrata	Gallus	gallus	PREDICTED: protein S100-A9-like [Gallus gallus]
XP_018115431.1	120	LOC108715114	eukaryota>metazoa>chordata>vertebrata	Xenopus	laevis	PREDICTED: protein S100-G-like [Xenopus laevis]
NP_001292080.1	119	S100A12	eukaryota>metazoa>chordata>vertebrata	Gallus	gallus	protein MRP-126 [Gallus gallus]
NP_001306125.1	117	60B8AG,CAGA,CFAG,CGLA,CP-10,L1Ag,MA387,MIF,MRP8,NIF,P8	eukaryota>metazoa>chordata>vertebrata	Homo	sapiens	protein S100-A8 isoform a [Homo sapiens]
XP_005554491.1	117	S100P	eukaryota>metazoa>chordata>vertebrata	Macaca	fascicularis	PREDICTED: protein S100-P [Macaca fascicularis]
XP_017347914.1	117	s100z	eukaryota>metazoa>chordata>vertebrata>actinopterygii	Ictalurus	punctatus	PREDICTED: protein S100-Z isoform X1 [Ictalurus punctatus]
NP_001306126.1	116	60B8AG,CAGA,CFAG,CGLA,CP-10,L1Ag,MA387,MIF,MRP8,NIF,P8	eukaryota>metazoa>chordata>vertebrata	Homo	sapiens	protein S100-A8 isoform b [Homo sapiens]
XP_018112635.1	116	LOC108713593	eukaryota>metazoa>chordata>vertebrata	Xenopus	laevis	PREDICTED: sentan-like [Xenopus laevis]
NP_002956.1	114	60B8AG,CAGB,CFAG,CGLB,L1AG,LIAG,MAC387,MIF,MRP14,NIF,P14	eukaryota>metazoa>chordata>vertebrata	Homo	sapiens	protein S100-A9 [Homo sapiens]
XP_005159486.1	114	zgc:91995	eukaryota>metazoa>chordata>vertebrata>actinopterygii	Danio	rerio	S100 calcium binding protein S isoform X2 [Danio rerio]
XP_005541846.1	114	S100A9	eukaryota>metazoa>chordata>vertebrata	Macaca	fascicularis	PREDICTED: protein S100-A9 [Macaca fascicularis]
NP_001268781.1	113	60B8Ag,AW546964,BEE22,Cagb,GAGB,L1Ag,MRP14,p14	eukaryota>metazoa>chordata>vertebrata	Mus	musculus	protein S100-A9 [Mus musculus]
NP_033140.1	113	60B8Ag,AW546964,BEE22,Cagb,GAGB,L1Ag,MRP14,p14	eukaryota>metazoa>chordata>vertebrata	Mus	musculus	protein S100-A9 [Mus musculus]
XP_017336138.1	113	s10ab	eukaryota>metazoa>chordata>vertebrata>actinopterygii	Ictalurus	punctatus	PREDICTED: s100-a11 isoform X1 [Ictalurus punctatus]
XP_003259338.1	112	S100A9	eukaryota>metazoa>chordata>vertebrata	Nomascus	leucogenys	PREDICTED: protein S100-A9 isoform X1 [Nomascus leucogenys]
XP_016857519.1	110	S100D	eukaryota>metazoa>chordata>vertebrata	Homo	sapiens	PREDICTED: protein S100-A5 isoform X2 [Homo sapiens]
XP_017324219.1	109	LOC108265897	eukaryota>metazoa>chordata>vertebrata>actinopterygii	Ictalurus	punctatus	PREDICTED: protein S100-A1-like [Ictalurus punctatus]
XP_017324228.1	109	LOC108265897	eukaryota>metazoa>chordata>vertebrata>actinopterygii	Ictalurus	punctatus	PREDICTED: protein S100-A1-like [Ictalurus punctatus]
XP_018102875.1	109	LOC108708559	eukaryota>metazoa>chordata>vertebrata	Xenopus	laevis	PREDICTED: protein S100-B-like isoform X1 [Xenopus laevis]
CBY32784.1	109	GSOID_T00032133001	eukaryota>metazoa>chordata	Oikopleura	dioica	unnamed protein product [Oikopleura dioica]
NP_955454.1	108	AY465109,Gm1020,S100a15,S100a15a,S100a17l1,S100A7f,S100A7L1	eukaryota>metazoa>chordata>vertebrata	Mus	musculus	protein S100-A15A [Mus musculus]
XP_003969387.1	108	LOC101076426	eukaryota>metazoa>chordata>vertebrata>actinopterygii	Takifugu	rubripes	PREDICTED: protein S100-A1-like [Takifugu rubripes]
XP_004089946.1	108	LOC101179466	eukaryota>metazoa>chordata>vertebrata	Nomascus	leucogenys	PREDICTED: protein S100-A15A [Nomascus leucogenys]
XP_005541840.1	108	LOC102120158	eukaryota>metazoa>chordata>vertebrata	Macaca	fascicularis	PREDICTED: protein S100-A15A isoform X2 [Macaca fascicularis]
XP_006501695.1	108	AY465109,Gm1020,S100a15,S100a15a,S100a17l1,S100A7f,S100A7L1	eukaryota>metazoa>chordata>vertebrata	Mus	musculus	PREDICTED: protein S100-A15A isoform X2 [Mus musculus]
XP_006501696.1	108	AY465109,Gm1020,S100a15,S100a15a,S100a17l1,S100A7f,S100A7L1	eukaryota>metazoa>chordata>vertebrata	Mus	musculus	PREDICTED: protein S100-A15A isoform X2 [Mus musculus]
XP_006501698.1	108	AY465109,Gm1020,S100a15,S100a15a,S100a17l1,S100A7f,S100A7L1	eukaryota>metazoa>chordata>vertebrata	Mus	musculus	PREDICTED: protein S100-A15A isoform X2 [Mus musculus]
XP_017323548.1	108	LOC108265566	eukaryota>metazoa>chordata>vertebrata>actinopterygii	Ictalurus	punctatus	PREDICTED: ictacalcin-like isoform X1 [Ictalurus punctatus]
XP_018102876.1	108	LOC108708559	eukaryota>metazoa>chordata>vertebrata	Xenopus	laevis	PREDICTED: protein S100-B-like isoform X2 [Xenopus laevis]
CAG11860.1	106	GSTEN:00033944:G:001	eukaryota>metazoa>chordata>vertebrata>actinopterygii	Tetraodon	nigroviridis	unnamed protein product [Tetraodon nigroviridis]
XP_004092680.1	106	S100A10	eukaryota>metazoa>chordata>vertebrata	Nomascus	leucogenys	PREDICTED: protein S100-A10 isoform X3 [Nomascus leucogenys]
XP_018115391.1	106	LOC108715052	eukaryota>metazoa>chordata>vertebrata	Xenopus	laevis	PREDICTED: sentan-like, partial [Xenopus laevis]
NP_005611.1	105	HEL-S-43,MLN70,S100C	eukaryota>metazoa>chordata>vertebrata	Homo	sapiens	protein S100-A11 [Homo sapiens]
XP_001922814.1	105	s100v1	eukaryota>metazoa>chordata>vertebrata>actinopterygii	Danio	rerio	PREDICTED: protein S100-P [Danio rerio]
XP_003259306.1	105	S100A11	eukaryota>metazoa>chordata>vertebrata	Nomascus	leucogenys	PREDICTED: protein S100-A11 [Nomascus leucogenys]
XP_003642685.1	105	S100A16	eukaryota>metazoa>chordata>vertebrata	Gallus	gallus	PREDICTED: protein S100-A16 [Gallus gallus]
XP_005541895.1	105	S100A11	eukaryota>metazoa>chordata>vertebrata	Macaca	fascicularis	PREDICTED: protein S100-A11 [Macaca fascicularis]
XP_015154043.1	105	S100A16	eukaryota>metazoa>chordata>vertebrata	Gallus	gallus	PREDICTED: protein S100-A16 [Gallus gallus]
NP_001156997.1	104	1110013O05Rik,S100a15,S114	eukaryota>metazoa>chordata>vertebrata	Mus	musculus	protein S100-A14 isoform a [Mus musculus]
NP_001182689.1	104	CaN19,EG628324,S100L	eukaryota>metazoa>chordata>vertebrata	Mus	musculus	predicted gene, EG628324 [Mus musculus]
NP_065723.1	104	BCMP84,S100A15	eukaryota>metazoa>chordata>vertebrata	Homo	sapiens	protein S100-A14 [Homo sapiens]
NP_079669.2	104	1110013O05Rik,S100a15,S114	eukaryota>metazoa>chordata>vertebrata	Mus	musculus	protein S100-A14 isoform a [Mus musculus]
XP_003259367.1	104	S100A5	eukaryota>metazoa>chordata>vertebrata	Nomascus	leucogenys	PREDICTED: protein S100-A5 isoform X1 [Nomascus leucogenys]
XP_003259371.1	104	S100A14	eukaryota>metazoa>chordata>vertebrata	Nomascus	leucogenys	PREDICTED: protein S100-A14 [Nomascus leucogenys]
XP_003259372.1	104	S100A14	eukaryota>metazoa>chordata>vertebrata	Nomascus	leucogenys	PREDICTED: protein S100-A14 [Nomascus leucogenys]
XP_004089939.1	104	S100A14	eukaryota>metazoa>chordata>vertebrata	Nomascus	leucogenys	PREDICTED: protein S100-A14 [Nomascus leucogenys]
XP_005245419.1	104	BCMP84,S100A15	eukaryota>metazoa>chordata>vertebrata	Homo	sapiens	PREDICTED: protein S100-A14 isoform X1 [Homo sapiens]
XP_005541824.1	104	S100A14	eukaryota>metazoa>chordata>vertebrata	Macaca	fascicularis	PREDICTED: protein S100-A14 [Macaca fascicularis]
XP_005541825.1	104	S100A14	eukaryota>metazoa>chordata>vertebrata	Macaca	fascicularis	PREDICTED: protein S100-A14 [Macaca fascicularis]
XP_005541826.1	104	S100A14	eukaryota>metazoa>chordata>vertebrata	Macaca	fascicularis	PREDICTED: protein S100-A14 [Macaca fascicularis]
XP_011508162.1	104	S100D	eukaryota>metazoa>chordata>vertebrata	Homo	sapiens	PREDICTED: protein S100-A5 isoform X3 [Homo sapiens]
XP_015312477.1	104	S100A14	eukaryota>metazoa>chordata>vertebrata	Macaca	fascicularis	PREDICTED: protein S100-A14 [Macaca fascicularis]
XP_016857364.1	104	BCMP84,S100A15	eukaryota>metazoa>chordata>vertebrata	Homo	sapiens	PREDICTED: protein S100-A14 isoform X1 [Homo sapiens]
XP_017336616.1	104	s100a14	eukaryota>metazoa>chordata>vertebrata>actinopterygii	Ictalurus	punctatus	PREDICTED: protein S100-A14 [Ictalurus punctatus]
CAF95168.1	103	GSTEN:00011699:G:001	eukaryota>metazoa>chordata>vertebrata>actinopterygii	Tetraodon	nigroviridis	unnamed protein product [Tetraodon nigroviridis]
CBY08190.1	103	GSOID_T00004203001	eukaryota>metazoa>chordata	Oikopleura	dioica	unnamed protein product [Oikopleura dioica]
NP_001303936.1	103	AAG13,DT1P1A7,S100F	eukaryota>metazoa>chordata>vertebrata	Homo	sapiens	protein S100-A16 [Homo sapiens]
NP_001303937.1	103	AAG13,DT1P1A7,S100F	eukaryota>metazoa>chordata>vertebrata	Homo	sapiens	protein S100-A16 [Homo sapiens]
NP_001313380.1	103	s100w	eukaryota>metazoa>chordata>vertebrata>actinopterygii	Danio	rerio	uncharacterized protein LOC569958 [Danio rerio]
NP_525127.1	103	AAG13,DT1P1A7,S100F	eukaryota>metazoa>chordata>vertebrata	Homo	sapiens	protein S100-A16 [Homo sapiens]
XP_003259355.1	103	S100A3	eukaryota>metazoa>chordata>vertebrata	Nomascus	leucogenys	PREDICTED: protein S100-A3 isoform X2 [Nomascus leucogenys]
XP_003259369.1	103	S100A16	eukaryota>metazoa>chordata>vertebrata	Nomascus	leucogenys	PREDICTED: protein S100-A16 [Nomascus leucogenys]
XP_003259370.1	103	S100A16	eukaryota>metazoa>chordata>vertebrata	Nomascus	leucogenys	PREDICTED: protein S100-A16 [Nomascus leucogenys]
XP_005541827.1	103	S100A16	eukaryota>metazoa>chordata>vertebrata	Macaca	fascicularis	PREDICTED: protein S100-A16 [Macaca fascicularis]
XP_005541828.1	103	S100A16	eukaryota>metazoa>chordata>vertebrata	Macaca	fascicularis	PREDICTED: protein S100-A16 [Macaca fascicularis]
XP_011607658.1	103	LOC105417196	eukaryota>metazoa>chordata>vertebrata>actinopterygii	Takifugu	rubripes	PREDICTED: protein S100-A12-like [Takifugu rubripes]
XP_015312482.1	103	S100A16	eukaryota>metazoa>chordata>vertebrata	Macaca	fascicularis	PREDICTED: protein S100-A16 [Macaca fascicularis]
CBY08190.1	103	GSOID_T00004203001	eukaryota>metazoa>chordata	Oikopleura	dioica	unnamed protein product [Oikopleura dioica]
CBY30360.1	103	GSOID_T00018224001	eukaryota>metazoa>chordata	Oikopleura	dioica	unnamed protein product [Oikopleura dioica]
CAG10837.1	102	GSTEN:00032587:G:001	eukaryota>metazoa>chordata>vertebrata>actinopterygii	Tetraodon	nigroviridis	unnamed protein product, partial [Tetraodon nigroviridis]
NP_001003989.1	102	zgc:91995	eukaryota>metazoa>chordata>vertebrata>actinopterygii	Danio	rerio	S100 calcium binding protein S [Danio rerio]
NP_001005961.1	102	zgc:101880	eukaryota>metazoa>chordata>vertebrata>actinopterygii	Danio	rerio	S100 calcium binding protein A10a [Danio rerio]
XP_012367002.1	102	S100A9	eukaryota>metazoa>chordata>vertebrata	Nomascus	leucogenys	PREDICTED: protein S100-A9 isoform X2 [Nomascus leucogenys]
NP_001106340.1	101	LOC100127300	eukaryota>metazoa>chordata>vertebrata	Xenopus	laevis	uncharacterized protein LOC100127300 [Xenopus laevis]
NP_001187971.1	101	s10aa	eukaryota>metazoa>chordata>vertebrata>actinopterygii	Ictalurus	punctatus	s100-a10 [Ictalurus punctatus]
NP_001306127.1	101	60B8AG,CAGA,CFAG,CGLA,CP-10,L1Ag,MA387,MIF,MRP8,NIF,P8	eukaryota>metazoa>chordata>vertebrata	Homo	sapiens	protein S100-A8 isoform c [Homo sapiens]
NP_002951.1	101	S100E	eukaryota>metazoa>chordata>vertebrata	Homo	sapiens	protein S100-A3 [Homo sapiens]
NP_002952.1	101	18A2,42A,CAPL,FSP1,MTS1,P9KA,PEL98	eukaryota>metazoa>chordata>vertebrata	Homo	sapiens	protein S100-A4 [Homo sapiens]
NP_002954.2	101	PSOR1,S100A7c	eukaryota>metazoa>chordata>vertebrata	Homo	sapiens	protein S100-A7 [Homo sapiens]
NP_035440.1	101	S100E	eukaryota>metazoa>chordata>vertebrata	Mus	musculus	protein S100-A3 [Mus musculus]
NP_035441.1	101	18A2,42a,Capl,FSp1,metastasin,Mts1,PeL98,pk9a	eukaryota>metazoa>chordata>vertebrata	Mus	musculus	protein S100-A4 [Mus musculus]
NP_062427.1	101	18A2,42A,CAPL,FSP1,MTS1,P9KA,PEL98	eukaryota>metazoa>chordata>vertebrata	Homo	sapiens	protein S100-A4 [Homo sapiens]
NP_789793.1	101	NICE-2,NICE2,S100A15,S100A7f,S100A7L1	eukaryota>metazoa>chordata>vertebrata	Homo	sapiens	protein S100-A7A [Homo sapiens]
NP_990497.1	101	S100A11	eukaryota>metazoa>chordata>vertebrata	Gallus	gallus	protein S100-A11 [Gallus gallus]
XP_003259347.1	101	S100A4	eukaryota>metazoa>chordata>vertebrata	Nomascus	leucogenys	PREDICTED: protein S100-A4 [Nomascus leucogenys]
XP_003259348.1	101	S100A4	eukaryota>metazoa>chordata>vertebrata	Nomascus	leucogenys	PREDICTED: protein S100-A4 [Nomascus leucogenys]
XP_003259349.1	101	S100A4	eukaryota>metazoa>chordata>vertebrata	Nomascus	leucogenys	PREDICTED: protein S100-A4 [Nomascus leucogenys]
XP_003259350.1	101	S100A4	eukaryota>metazoa>chordata>vertebrata	Nomascus	leucogenys	PREDICTED: protein S100-A4 [Nomascus leucogenys]
XP_003259354.1	101	S100A3	eukaryota>metazoa>chordata>vertebrata	Nomascus	leucogenys	PREDICTED: protein S100-A3 isoform X3 [Nomascus leucogenys]
XP_003259356.1	101	S100A3	eukaryota>metazoa>chordata>vertebrata	Nomascus	leucogenys	PREDICTED: protein S100-A3 isoform X3 [Nomascus leucogenys]
XP_003259357.1	101	S100A3	eukaryota>metazoa>chordata>vertebrata	Nomascus	leucogenys	PREDICTED: protein S100-A3 isoform X3 [Nomascus leucogenys]
XP_003965864.1	101	LOC101068299	eukaryota>metazoa>chordata>vertebrata>actinopterygii	Takifugu	rubripes	PREDICTED: protein S100-A1-like [Takifugu rubripes]
XP_004089941.1	101	LOC100587124	eukaryota>metazoa>chordata>vertebrata	Nomascus	leucogenys	PREDICTED: protein S100-A7 [Nomascus leucogenys]
XP_004089942.1	101	LOC100587124	eukaryota>metazoa>chordata>vertebrata	Nomascus	leucogenys	PREDICTED: protein S100-A7 [Nomascus leucogenys]
XP_004089943.1	101	S100A8	eukaryota>metazoa>chordata>vertebrata	Nomascus	leucogenys	PREDICTED: protein S100-A8 isoform X2 [Nomascus leucogenys]
XP_004089944.1	101	LOC101179385	eukaryota>metazoa>chordata>vertebrata	Nomascus	leucogenys	PREDICTED: protein S100-A7-like [Nomascus leucogenys]
XP_004089945.1	101	LOC101179385	eukaryota>metazoa>chordata>vertebrata	Nomascus	leucogenys	PREDICTED: protein S100-A7-like [Nomascus leucogenys]
XP_005541835.1	101	S100A4	eukaryota>metazoa>chordata>vertebrata	Macaca	fascicularis	PREDICTED: protein S100-A4 [Macaca fascicularis]
XP_005541838.1	101	LOC102119771	eukaryota>metazoa>chordata>vertebrata	Macaca	fascicularis	PREDICTED: protein S100-A7 [Macaca fascicularis]
XP_005541843.1	101	LOC102120794	eukaryota>metazoa>chordata>vertebrata	Macaca	fascicularis	PREDICTED: protein S100-A7 isoform X1 [Macaca fascicularis]
XP_006501234.1	101	S100E	eukaryota>metazoa>chordata>vertebrata	Mus	musculus	PREDICTED: protein S100-A3 isoform X1 [Mus musculus]
XP_006501236.1	101	S100E	eukaryota>metazoa>chordata>vertebrata	Mus	musculus	PREDICTED: protein S100-A3 isoform X1 [Mus musculus]
XP_006501237.1	101	S100E	eukaryota>metazoa>chordata>vertebrata	Mus	musculus	PREDICTED: protein S100-A3 isoform X1 [Mus musculus]
XP_006501238.1	101	S100E	eukaryota>metazoa>chordata>vertebrata	Mus	musculus	PREDICTED: protein S100-A3 isoform X1 [Mus musculus]
XP_006501239.1	101	S100E	eukaryota>metazoa>chordata>vertebrata	Mus	musculus	PREDICTED: protein S100-A3 isoform X1 [Mus musculus]
XP_006501240.1	101	18A2,42a,Capl,FSp1,metastasin,Mts1,PeL98,pk9a	eukaryota>metazoa>chordata>vertebrata	Mus	musculus	PREDICTED: protein S100-A4 isoform X1 [Mus musculus]
XP_006501241.1	101	18A2,42a,Capl,FSp1,metastasin,Mts1,PeL98,pk9a	eukaryota>metazoa>chordata>vertebrata	Mus	musculus	PREDICTED: protein S100-A4 isoform X1 [Mus musculus]
XP_011238349.1	101	S100E	eukaryota>metazoa>chordata>vertebrata	Mus	musculus	PREDICTED: protein S100-A3 isoform X1 [Mus musculus]
XP_012366970.1	101	S100A4	eukaryota>metazoa>chordata>vertebrata	Nomascus	leucogenys	PREDICTED: protein S100-A4 [Nomascus leucogenys]
XP_012366971.1	101	S100A4	eukaryota>metazoa>chordata>vertebrata	Nomascus	leucogenys	PREDICTED: protein S100-A4 [Nomascus leucogenys]
XP_015312508.1	101	S100A3	eukaryota>metazoa>chordata>vertebrata	Macaca	fascicularis	PREDICTED: protein S100-A3 [Macaca fascicularis]
XP_017174998.1	101	S100E	eukaryota>metazoa>chordata>vertebrata	Mus	musculus	PREDICTED: protein S100-A3 isoform X1 [Mus musculus]
XP_018083990.1	101	LOC100127300	eukaryota>metazoa>chordata>vertebrata	Xenopus	laevis	PREDICTED: uncharacterized protein LOC100127300 isoform X1 [Xenopus laevis]
XP_018083991.1	101	LOC100127300	eukaryota>metazoa>chordata>vertebrata	Xenopus	laevis	PREDICTED: uncharacterized protein LOC100127300 isoform X1 [Xenopus laevis]
NP_001073630.2	100	fk35d04,s100v1,si:dkey-78d16.1,wu:fk35d04	eukaryota>metazoa>chordata>vertebrata>actinopterygii	Danio	rerio	protein S100-A14 [Danio rerio]
NP_998168.1	100	hm:zehn1116,wu:fa94d11,wu:fb05h04,zgc:56142	eukaryota>metazoa>chordata>vertebrata>actinopterygii	Danio	rerio	S100 calcium binding protein A10b [Danio rerio]
XP_003969338.1	100	LOC101065219	eukaryota>metazoa>chordata>vertebrata>actinopterygii	Takifugu	rubripes	PREDICTED: protein S100-A1-like [Takifugu rubripes]
XP_004948276.1	100	S100A4	eukaryota>metazoa>chordata>vertebrata	Gallus	gallus	PREDICTED: protein S100-A4 [Gallus gallus]
XP_018087231.1	100	LOC108699644	eukaryota>metazoa>chordata>vertebrata	Xenopus	laevis	PREDICTED: protein S100-A13-like [Xenopus laevis]
XP_018087233.1	100	LOC108699644	eukaryota>metazoa>chordata>vertebrata	Xenopus	laevis	PREDICTED: protein S100-A13-like [Xenopus laevis]
CAG10829.1	99	GSTEN:00032575:G:001	eukaryota>metazoa>chordata>vertebrata>actinopterygii	Tetraodon	nigroviridis	unnamed protein product, partial [Tetraodon nigroviridis]
NP_001019562.1	99	wu:fj01a12,zgc:110464	eukaryota>metazoa>chordata>vertebrata>actinopterygii	Danio	rerio	protein S100-Z [Danio rerio]
NP_001074628.1	99	Gm625	eukaryota>metazoa>chordata>vertebrata	Mus	musculus	protein S100-Z [Mus musculus]
NP_001091287.1	99	gm625,s100-zeta,s100z	eukaryota>metazoa>chordata>vertebrata	Xenopus	laevis	S100 calcium binding protein Z L homeolog [Xenopus laevis]
NP_570128.2	99	Gm625,S100-zeta	eukaryota>metazoa>chordata>vertebrata	Homo	sapiens	protein S100-Z [Homo sapiens]

XP_003261542.1	99	S100Z	eukaryota>metazoa>chordata>vertebrata	Nomascus	leucogenys	PREDICTED: protein S100-Z [Nomascus leucogenys]
XP_011541541.1	99	Gm625,S100-zeta	eukaryota>metazoa>chordata>vertebrata	Homo	sapiens	PREDICTED: protein S100-Z isoform X1 [Homo sapiens]
XP_011541542.1	99	Gm625,S100-zeta	eukaryota>metazoa>chordata>vertebrata	Homo	sapiens	PREDICTED: protein S100-Z isoform X1 [Homo sapiens]
XP_011541543.1	99	Gm625,S100-zeta	eukaryota>metazoa>chordata>vertebrata	Homo	sapiens	PREDICTED: protein S100-Z isoform X1 [Homo sapiens]
XP_011541546.1	99	Gm625,S100-zeta	eukaryota>metazoa>chordata>vertebrata	Homo	sapiens	PREDICTED: protein S100-Z isoform X1 [Homo sapiens]
XP_011541547.1	99	Gm625,S100-zeta	eukaryota>metazoa>chordata>vertebrata	Homo	sapiens	PREDICTED: protein S100-Z isoform X1 [Homo sapiens]
XP_015154048.1	99	S100A14	eukaryota>metazoa>chordata>vertebrata	Gallus	gallus	PREDICTED: protein S100-A14 [Gallus gallus]
XP_016864660.1	99	Gm625,S100-zeta	eukaryota>metazoa>chordata>vertebrata	Homo	sapiens	PREDICTED: protein S100-Z isoform X1 [Homo sapiens]
XP_016864661.1	99	Gm625,S100-zeta	eukaryota>metazoa>chordata>vertebrata	Homo	sapiens	PREDICTED: protein S100-Z isoform X1 [Homo sapiens]
XP_016864662.1	99	Gm625,S100-zeta	eukaryota>metazoa>chordata>vertebrata	Homo	sapiens	PREDICTED: protein S100-Z isoform X1 [Homo sapiens]
XP_018098399.1	99	LOC108706452	eukaryota>metazoa>chordata>vertebrata	Xenopus	laevis	PREDICTED: protein S100-Z-like [Xenopus laevis]
XP_018115390.1	99	LOC108715051	eukaryota>metazoa>chordata>vertebrata	Xenopus	laevis	PREDICTED: protein S100-B-like [Xenopus laevis]
XP_018123499.1	99	gm625,s100-zeta,s100z	eukaryota>metazoa>chordata>vertebrata	Xenopus	laevis	PREDICTED: S100 calcium binding protein Z L homeolog isoform X1 [Xenopus laevis]
CAF96543.1	98	GSTEN:00013622:G:001	eukaryota>metazoa>chordata>vertebrata>actinopterygii	Tetraodon	nigroviridis	unnamed protein product [Tetraodon nigroviridis]
CAG11861.1	98	GSTEN:00033945:G:001	eukaryota>metazoa>chordata>vertebrata>actinopterygii	Tetraodon	nigroviridis	unnamed protein product [Tetraodon nigroviridis]
NP_001019381.1	98	S100A13	eukaryota>metazoa>chordata>vertebrata	Homo	sapiens	protein S100-A13 [Homo sapiens]
NP_001019382.1	98	S100A13	eukaryota>metazoa>chordata>vertebrata	Homo	sapiens	protein S100-A13 [Homo sapiens]
NP_001019383.1	98	S100A13	eukaryota>metazoa>chordata>vertebrata	Homo	sapiens	protein S100-A13 [Homo sapiens]
NP_001019384.1	98	S100A13	eukaryota>metazoa>chordata>vertebrata	Homo	sapiens	protein S100-A13 [Homo sapiens]
NP_001187547.1	98	s10a1	eukaryota>metazoa>chordata>vertebrata>actinopterygii	Ictalurus	punctatus	s100-a1 [Ictalurus punctatus]
NP_001289127.1	98	S100Z	eukaryota>metazoa>chordata>vertebrata	Gallus	gallus	protein S100-Z [Gallus gallus]
NP_005970.1	98	S100A13	eukaryota>metazoa>chordata>vertebrata	Homo	sapiens	protein S100-A13 [Homo sapiens]
NP_058020.1	98	cal,Emap1,EMAPI,S100a14,S100c	eukaryota>metazoa>chordata>vertebrata	Mus	musculus	protein S100-A11 [Mus musculus]
XP_005245491.1	98	S100A13	eukaryota>metazoa>chordata>vertebrata	Homo	sapiens	PREDICTED: protein S100-A13 isoform X1 [Homo sapiens]
XP_005541821.1	98	S100A13	eukaryota>metazoa>chordata>vertebrata	Macaca	fascicularis	PREDICTED: protein S100-A13 [Macaca fascicularis]
XP_005541822.1	98	S100A13	eukaryota>metazoa>chordata>vertebrata	Macaca	fascicularis	PREDICTED: protein S100-A13 [Macaca fascicularis]
XP_005541829.1	98	S100A2	eukaryota>metazoa>chordata>vertebrata	Macaca	fascicularis	PREDICTED: protein S100-A2 [Macaca fascicularis]
XP_005541830.1	98	S100A2	eukaryota>metazoa>chordata>vertebrata	Macaca	fascicularis	PREDICTED: protein S100-A2 [Macaca fascicularis]
XP_011508164.1	98	S100A13	eukaryota>metazoa>chordata>vertebrata	Homo	sapiens	PREDICTED: protein S100-A13 isoform X1 [Homo sapiens]
XP_011508165.1	98	S100A13	eukaryota>metazoa>chordata>vertebrata	Homo	sapiens	PREDICTED: protein S100-A13 isoform X1 [Homo sapiens]
XP_011508166.1	98	S100A13	eukaryota>metazoa>chordata>vertebrata	Homo	sapiens	PREDICTED: protein S100-A13 isoform X1 [Homo sapiens]
XP_015132839.1	98	S100Z	eukaryota>metazoa>chordata>vertebrata	Gallus	gallus	PREDICTED: protein S100-Z isoform X1 [Gallus gallus]
XP_016857523.1	98	S100A13	eukaryota>metazoa>chordata>vertebrata	Homo	sapiens	PREDICTED: protein S100-A13 isoform X1 [Homo sapiens]
XP_016857524.1	98	S100A13	eukaryota>metazoa>chordata>vertebrata	Homo	sapiens	PREDICTED: protein S100-A13 isoform X1 [Homo sapiens]
XP_016857525.1	98	S100A13	eukaryota>metazoa>chordata>vertebrata	Homo	sapiens	PREDICTED: protein S100-A13 isoform X1 [Homo sapiens]
CAG05262.1	97	GSTEN:00025230:G:001	eukaryota>metazoa>chordata>vertebrata>actinopterygii	Tetraodon	nigroviridis	unnamed protein product, partial [Tetraodon nigroviridis]
NP_001076426.1	97	zgc:171263	eukaryota>metazoa>chordata>vertebrata>actinopterygii	Danio	rerio	ictacalcin 2 [Danio rerio]
NP_002957.1	97	42C,ANX2L,ANX2LG,CAL1L,Ca[1],CLP11,GP11,p10,P11	eukaryota>metazoa>chordata>vertebrata	Homo	sapiens	protein S100-A10 [Homo sapiens]
NP_005969.1	97	CAN19,S100L	eukaryota>metazoa>chordata>vertebrata	Homo	sapiens	protein S100-A2 [Homo sapiens]
NP_033138.1	97	42C,AA409961,AL024248,CAL12,Cal11,CLP11,p10,p11	eukaryota>metazoa>chordata>vertebrata	Mus	musculus	protein S100-A10 [Mus musculus]
NP_990837.1	97	P11	eukaryota>metazoa>chordata>vertebrata	Gallus	gallus	protein S100-A10 [Gallus gallus]
XP_003259351.1	97	S100A2	eukaryota>metazoa>chordata>vertebrata	Nomascus	leucogenys	PREDICTED: protein S100-A2 [Nomascus leucogenys]
XP_003259352.1	97	S100A2	eukaryota>metazoa>chordata>vertebrata	Nomascus	leucogenys	PREDICTED: protein S100-A2 [Nomascus leucogenys]
XP_003282254.1	97	S100A10	eukaryota>metazoa>chordata>vertebrata	Nomascus	leucogenys	PREDICTED: protein S100-A10 isoform X4 [Nomascus leucogenys]
XP_003973567.1	97	s100b	eukaryota>metazoa>chordata>vertebrata>actinopterygii	Takifugu	rubripes	PREDICTED: protein S100-B [Takifugu rubripes]
XP_005541900.1	97	S100A10	eukaryota>metazoa>chordata>vertebrata	Macaca	fascicularis	PREDICTED: protein S100-A10 isoform X2 [Macaca fascicularis]
XP_005541901.1	97	S100A10	eukaryota>metazoa>chordata>vertebrata	Macaca	fascicularis	PREDICTED: protein S100-A10 isoform X2 [Macaca fascicularis]
XP_012366634.1	97	S100A2	eukaryota>metazoa>chordata>vertebrata	Nomascus	leucogenys	PREDICTED: protein S100-A2 [Nomascus leucogenys]
XP_012366636.1	97	S100A2	eukaryota>metazoa>chordata>vertebrata	Nomascus	leucogenys	PREDICTED: protein S100-A2 [Nomascus leucogenys]
XP_015135488.1	97	P11	eukaryota>metazoa>chordata>vertebrata	Gallus	gallus	PREDICTED: protein S100-A10 isoform X1 [Gallus gallus]
XP_015312662.1	97	S100A10	eukaryota>metazoa>chordata>vertebrata	Macaca	fascicularis	PREDICTED: protein S100-A10 isoform X2 [Macaca fascicularis]
XP_017340854.1	97	LOC108274911	eukaryota>metazoa>chordata>vertebrata>actinopterygii	Ictalurus	punctatus	PREDICTED: protein S100-B-like isoform X1 [Ictalurus punctatus]
XP_017347915.1	97	s100z	eukaryota>metazoa>chordata>vertebrata>actinopterygii	Ictalurus	punctatus	PREDICTED: protein S100-Z isoform X2 [Ictalurus punctatus]
XP_017347916.1	97	s100z	eukaryota>metazoa>chordata>vertebrata>actinopterygii	Ictalurus	punctatus	PREDICTED: protein S100-Z isoform X2 [Ictalurus punctatus]
XP_017347917.1	97	s100z	eukaryota>metazoa>chordata>vertebrata>actinopterygii	Ictalurus	punctatus	PREDICTED: protein S100-Z isoform X2 [Ictalurus punctatus]
XP_017347918.1	97	s100z	eukaryota>metazoa>chordata>vertebrata>actinopterygii	Ictalurus	punctatus	PREDICTED: protein S100-Z isoform X2 [Ictalurus punctatus]
NP_001013513.1	96	im:7139587,zgc:110771	eukaryota>metazoa>chordata>vertebrata>actinopterygii	Danio	rerio	S100 calcium binding protein T [Danio rerio]
NP_001079240.1	96	42c,anx21,anx2lg,cal11,calpactin,clp11,gp11,p11,p11-A,s100a10,s100a10-a,s100a10-b,s100a10a,s100a10b	eukaryota>metazoa>chordata>vertebrata	Xenopus	laevis	protein S100-A10 isoform 1 [Xenopus laevis]
NP_001082820.1	96	s100a1	eukaryota>metazoa>chordata>vertebrata>actinopterygii	Danio	rerio	protein S100-A1 [Danio rerio]
XP_017206829.1	96	s100a1	eukaryota>metazoa>chordata>vertebrata>actinopterygii	Danio	rerio	protein S100-A1 isoform X1 [Danio rerio]
XP_018081132.1	96	LOC108696356	eukaryota>metazoa>chordata>vertebrata	Xenopus	laevis	PREDICTED: protein S100-P-like [Xenopus laevis]
XP_018089081.1	96	LOC446964	eukaryota>metazoa>chordata>vertebrata	Xenopus	laevis	PREDICTED: protein S100-A10-like isoform X1 [Xenopus laevis]
NP_001002552.1	95	si:dkey-110p14.2,wu:fq18b10,zgc:92776	eukaryota>metazoa>chordata>vertebrata>actinopterygii	Danio	rerio	protein S100-B [Danio rerio]
NP_005971.1	95	MIG9	eukaryota>metazoa>chordata>vertebrata	Homo	sapiens	protein S100-P [Homo sapiens]
NP_997926.1	95	fa91d07,mg:cb02f02,s100a4a,wu:fa91d07	eukaryota>metazoa>chordata>vertebrata>actinopterygii	Danio	rerio	ictacalcin [Danio rerio]
XP_003278569.3	95	S100P	eukaryota>metazoa>chordata>vertebrata	Nomascus	leucogenys	PREDICTED: protein S100-P [Nomascus leucogenys]
XP_003966755.1	95	s100p	eukaryota>metazoa>chordata>vertebrata>actinopterygii	Takifugu	rubripes	PREDICTED: protein S100-P [Takifugu rubripes]
XP_009294477.1	95	si:dkey-110p14.2,wu:fq18b10,zgc:92776	eukaryota>metazoa>chordata>vertebrata>actinopterygii	Danio	rerio	protein S100-B isoform X1 [Danio rerio]
XP_017314622.1	95	LOC108259544	eukaryota>metazoa>chordata>vertebrata>actinopterygii	Ictalurus	punctatus	PREDICTED: protein S100-B-like [Ictalurus punctatus]
XP_018087234.1	95	LOC108699645	eukaryota>metazoa>chordata>vertebrata	Xenopus	laevis	PREDICTED: protein S100-A13-like [Xenopus laevis]
XP_018087235.1	95	LOC108699645	eukaryota>metazoa>chordata>vertebrata	Xenopus	laevis	PREDICTED: protein S100-A13-like [Xenopus laevis]
XP_018087236.1	95	LOC108699645	eukaryota>metazoa>chordata>vertebrata	Xenopus	laevis	PREDICTED: protein S100-A13-like [Xenopus laevis]
XP_018087237.1	95	LOC108699645	eukaryota>metazoa>chordata>vertebrata	Xenopus	laevis	PREDICTED: protein S100-A13-like [Xenopus laevis]
XP_018087238.1	95	LOC108699645	eukaryota>metazoa>chordata>vertebrata	Xenopus	laevis	PREDICTED: protein S100-A13-like [Xenopus laevis]
XP_018087239.1	95	LOC108699645	eukaryota>metazoa>chordata>vertebrata	Xenopus	laevis	PREDICTED: protein S100-A13-like [Xenopus laevis]
XP_018087240.1	95	LOC108699646	eukaryota>metazoa>chordata>vertebrata	Xenopus	laevis	PREDICTED: protein S100-G-like [Xenopus laevis]
XP_018089082.1	95	LOC446964	eukaryota>metazoa>chordata>vertebrata	Xenopus	laevis	PREDICTED: protein S100-A10-like isoform X2 [Xenopus laevis]
XP_018089083.1	95	LOC108700424	eukaryota>metazoa>chordata>vertebrata	Xenopus	laevis	PREDICTED: protein S100-G-like [Xenopus laevis]
NP_001270255.1	94	S100A1	eukaryota>metazoa>chordata>vertebrata	Macaca	fascicularis	protein S100-A1 [Macaca fascicularis]
NP_001289102.1	94	S100A1	eukaryota>metazoa>chordata>vertebrata	Gallus	gallus	protein S100-A1 [Gallus gallus]
NP_006262.1	94	S100,S100-alpha,S100A	eukaryota>metazoa>chordata>vertebrata	Homo	sapiens	protein S100-A1 [Homo sapiens]
NP_035439.1	94	AI266795,S100,S100a	eukaryota>metazoa>chordata>vertebrata	Mus	musculus	protein S100-A1 [Mus musculus]
XP_003259374.1	94	S100A1	eukaryota>metazoa>chordata>vertebrata	Nomascus	leucogenys	PREDICTED: protein S100-A1 [Nomascus leucogenys]
XP_003259375.1	94	S100A1	eukaryota>metazoa>chordata>vertebrata	Nomascus	leucogenys	PREDICTED: protein S100-A1 [Nomascus leucogenys]
XP_003642677.1	94	S100A1	eukaryota>metazoa>chordata>vertebrata	Gallus	gallus	PREDICTED: protein S100-A1 isoform X1 [Gallus gallus]
XP_012366637.1	94	S100A1	eukaryota>metazoa>chordata>vertebrata	Nomascus	leucogenys	PREDICTED: protein S100-A1 [Nomascus leucogenys]
XP_018087241.1	94	LOC108699648	eukaryota>metazoa>chordata>vertebrata	Xenopus	laevis	PREDICTED: protein S100-A1-like [Xenopus laevis]
XP_018087242.1	94	LOC108699648	eukaryota>metazoa>chordata>vertebrata	Xenopus	laevis	PREDICTED: protein S100-A1-like [Xenopus laevis]
XP_018089091.1	94	LOC108700427	eukaryota>metazoa>chordata>vertebrata	Xenopus	laevis	PREDICTED: protein S100-A1-like [Xenopus laevis]
CAF90805.1	93	GSTEN:00005225:G:001	eukaryota>metazoa>chordata>vertebrata>actinopterygii	Tetraodon	nigroviridis	unnamed protein product, partial [Tetraodon nigroviridis]
NP_001306130.1	93	60B8AG,CAGA,CFAG,CGLA,CP-10,L1Ag,MA387,MIF,MRP8,NIF,P8	eukaryota>metazoa>chordata>vertebrata	Homo	sapiens	protein S100-A8 isoform d [Homo sapiens]
NP_002955.2	93	60B8AG,CAGA,CFAG,CGLA,CP-10,L1Ag,MA387,MIF,MRP8,NIF,P8	eukaryota>metazoa>chordata>vertebrata	Homo	sapiens	protein S100-A8 isoform d [Homo sapiens]
NP_035442.1	93	S100D9	eukaryota>metazoa>chordata>vertebrata	Mus	musculus	protein S100-A5 [Mus musculus]
XP_003259340.1	93	S100A8	eukaryota>metazoa>chordata>vertebrata	Nomascus	leucogenys	PREDICTED: protein S100-A8 isoform X3 [Nomascus leucogenys]
XP_003259342.1	93	S100A8	eukaryota>metazoa>chordata>vertebrata	Nomascus	leucogenys	PREDICTED: protein S100-A8 isoform X3 [Nomascus leucogenys]
XP_003969339.1	93	LOC101065443	eukaryota>metazoa>chordata>vertebrata>actinopterygii	Takifugu	rubripes	PREDICTED: ictacalcin-like [Takifugu rubripes]
XP_005541844.1	93	S100A8	eukaryota>metazoa>chordata>vertebrata	Macaca	fascicularis	PREDICTED: protein S100-A8 [Macaca fascicularis]
XP_006501242.1	93	S100D9	eukaryota>metazoa>chordata>vertebrata	Mus	musculus	PREDICTED: protein S100-A5 isoform X1 [Mus musculus]
XP_006501243.1	93	S100D9	eukaryota>metazoa>chordata>vertebrata	Mus	musculus	PREDICTED: protein S100-A5 isoform X1 [Mus musculus]
XP_006501244.1	93	S100D9	eukaryota>metazoa>chordata>vertebrata	Mus	musculus	PREDICTED: protein S100-A5 isoform X1 [Mus musculus]
XP_011607737.1	93	LOC101065443	eukaryota>metazoa>chordata>vertebrata>actinopterygii	Takifugu	rubripes	PREDICTED: ictacalcin-like [Takifugu rubripes]
XP_015312549.1	93	S100A8	eukaryota>metazoa>chordata>vertebrata	Macaca	fascicularis	PREDICTED: protein S100-A8 [Macaca fascicularis]
XP_017351975.1	93	LOC108280979	eukaryota>metazoa>chordata>vertebrata>actinopterygii	Ictalurus	punctatus	PREDICTED: protein S100-B-like [Ictalurus punctatus]
NP_001187203.1	92	S10I	eukaryota>metazoa>chordata>vertebrata>actinopterygii	Ictalurus	punctatus	ictacalcin [Ictalurus punctatus]
NP_001270589.1	92	S100B	eukaryota>metazoa>chordata>vertebrata	Macaca	fascicularis	protein S100-B [Macaca fascicularis]
NP_001289071.1	92	S100B	eukaryota>metazoa>chordata>vertebrata	Gallus	gallus	protein S100-B [Gallus gallus]
NP_002953.2	92	S100D	eukaryota>metazoa>chordata>vertebrata	Homo	sapiens	protein S100-A5 [Homo sapiens]
NP_005612.1	92	CAAF1,CAGC,CGRP,ENRAGE,MRP-6,MRP6,p6	eukaryota>metazoa>chordata>vertebrata	Homo	sapiens	protein S100-A12 [Homo sapiens]
NP_006263.1	92	NEF,S100,S100-B,S100beta	eukaryota>metazoa>chordata>vertebrata	Homo	sapiens	protein S100-B [Homo sapiens]
NP_033141.1	92	AI850290,Bpb	eukaryota>metazoa>chordata>vertebrata	Mus	musculus	protein S100-B [Mus musculus]
NP_989479.1	92	S100A6	eukaryota>metazoa>chordata>vertebrata	Gallus	gallus	protein S100-A6 [Gallus gallus]
XP_003259366.1	92	S100A5	eukaryota>metazoa>chordata>vertebrata	Nomascus	leucogenys	PREDICTED: protein S100-A5 isoform X2 [Nomascus leucogenys]
XP_003277452.1	92	S100B	eukaryota>metazoa>chordata>vertebrata	Nomascus	leucogenys	PREDICTED: protein S100-B [Nomascus leucogenys]
XP_003277454.1	92	S100B	eukaryota>metazoa>chordata>vertebrata	Nomascus	leucogenys	PREDICTED: protein S100-B [Nomascus leucogenys]
XP_003966278.2	92	LOC101071509	eukaryota>metazoa>chordata>vertebrata>actinopterygii	Takifugu	rubripes	PREDICTED: protein S100-A1-like [Takifugu rubripes]
XP_005541836.1	92	S100A5	eukaryota>metazoa>chordata>vertebrata	Macaca	fascicularis	PREDICTED: protein S100-A5 [Macaca fascicularis]
XP_005548529.1	92	S100B	eukaryota>metazoa>chordata>vertebrata	Macaca	fascicularis	PREDICTED: protein S100-B isoform X1 [Macaca fascicularis]
XP_012357257.1	92	S100B	eukaryota>metazoa>chordata>vertebrata	Nomascus	leucogenys	PREDICTED: protein S100-B [Nomascus leucogenys]
XP_015312559.1	92	S100A12	eukaryota>metazoa>chordata>vertebrata	Macaca	fascicularis	PREDICTED: LOW QUALITY PROTEIN: protein S100-A12 [Macaca fascicularis]
XP_016857520.1	92	S100D	eukaryota>metazoa>chordata>vertebrata	Homo	sapiens	PREDICTED: protein S100-A5 isoform X4 [Homo sapiens]
XP_016857521.1	92	S100D	eukaryota>metazoa>chordata>vertebrata	Homo	sapiens	PREDICTED: protein S100-A5 isoform X4 [Homo sapiens]
XP_016883913.1	92	NEF,S100,S100-B,S100beta	eukaryota>metazoa>chordata>vertebrata	Homo	sapiens	PREDICTED: protein S100-B isoform X1 [Homo sapiens]
XP_017323558.1	92	LOC108265566	eukaryota>metazoa>chordata>vertebrata>actinopterygii	Ictalurus	punctatus	PREDICTED: ictacalcin-like isoform X2 [Ictalurus punctatus]
XP_017323574.1	92	LOC108265573	eukaryota>metazoa>chordata>vertebrata>actinopterygii	Ictalurus	punctatus	PREDICTED: ictacalcin [Ictalurus punctatus]
XP_017323586.1	92	LOC108265578	eukaryota>metazoa>chordata>vertebrata>actinopterygii	Ictalurus	punctatus	PREDICTED: ictacalcin-like [Ictalurus punctatus]
XP_017323594.1	92	LOC108265578	eukaryota>metazoa>chordata>vertebrata>actinopterygii	Ictalurus	punctatus	PREDICTED: ictacalcin-like [Ictalurus punctatus]
XP_017323603.1	92	LOC108265578	eukaryota>metazoa>chordata>vertebrata>actinopterygii	Ictalurus	punctatus	PREDICTED: ictacalcin-like [Ictalurus punctatus]
XP_018087243.1	92	LOC108699649	eukaryota>metazoa>chordata>vertebrata	Xenopus	laevis	PREDICTED: protein S100-A2-like [Xenopus laevis]
XP_018089084.1	92	LOC108700425	eukaryota>metazoa>chordata>vertebrata	Xenopus	laevis	PREDICTED: protein S100-G-like [Xenopus laevis]
XP_421894.1	92	S100B	eukaryota>metazoa>chordata>vertebrata	Gallus	gallus	PREDICTED: protein S100-B isoform X1 [Gallus gallus]
NP_001313411.1	91	fd10a10,wu:fd10a10	eukaryota>metazoa>chordata>vertebrata>actinopterygii	Danio	rerio	uncharacterized protein LOC325758 [Danio rerio]
XP_003259339.1	91	S100A12	eukaryota>metazoa>chordata>vertebrata	Nomascus	leucogenys	PREDICTED: protein S100-A12 [Nomascus leucogenys]
XP_017324242.1	91	LOC108265907	eukaryota>metazoa>chordata>vertebrata>actinopterygii	Ictalurus	punctatus	PREDICTED: protein S100-A16-like [Ictalurus punctatus]
CAG10828.1	90	GSTEN:00032574:G:001	eukaryota>metazoa>chordata>vertebrata>actinopterygii	Tetraodon	nigroviridis	unnamed protein product [Tetraodon nigroviridis]
NP_055439.1	90	2A9,5B10,CABP,CACY,PRA	eukaryota>metazoa>chordata>vertebrata	Homo	sapiens	protein S100-A6 [Homo sapiens]
XP_003259360.1	90	S100A6	eukaryota>metazoa>chordata>vertebrata	Nomascus	leucogenys	PREDICTED: protein S100-A6 [Nomascus leucogenys]
XP_003966262.1	90	LOC101067460	eukaryota>metazoa>chordata>vertebrata>actinopterygii	Takifugu	rubripes	PREDICTED: protein S100-A16-like [Takifugu rubripes]
XP_005541837.1	90	S100A6	eukaryota>metazoa>chordata>vertebrata	Macaca	fascicularis	PREDICTED: protein S100-A6 [Macaca fascicularis]
XP_012366875.1	90	S100A6	eukaryota>metazoa>chordata>vertebrata	Nomascus	leucogenys	PREDICTED: protein S100-A6 [Nomascus leucogenys]
XP_012366876.1	90	S100A6	eukaryota>metazoa>chordata>vertebrata	Nomascus	leucogenys	PREDICTED: protein S100-A6 [Nomascus leucogenys]
XP_012366877.1	90	S100A6	eukaryota>metazoa>chordata>vertebrata	Nomascus	leucogenys	PREDICTED: protein S100-A6 [Nomascus leucogenys]
XP_012366878.1	90	S100A6	eukaryota>metazoa>chordata>vertebrata	Nomascus	leucogenys	PREDICTED: protein S100-A6 [Nomascus leucogenys]
XP_012366879.1	90	S100A6	eukaryota>metazoa>chordata>vertebrata	Nomascus	leucogenys	PREDICTED: protein S100-A6 [Nomascus leucogenys]
XP_012366880.1	90	S100A6	eukaryota>metazoa>chordata>vertebrata	Nomascus	leucogenys	PREDICTED: protein S100-A6 [Nomascus leucogenys]
XP_012366881.1	90	S100A6	eukaryota>metazoa>chordata>vertebrata	Nomascus	leucogenys	PREDICTED: protein S100-A6 [Nomascus leucogenys]
XP_016857522.1	90	2A9,5B10,CABP,CACY,PRA	eukaryota>metazoa>chordata>vertebrata	Homo	sapiens	PREDICTED: protein S100-A6 isoform X1 [Homo sapiens]
NP_035443.1	89	2A9,5B10,Cacy,CALCYCLIN,PRA	eukaryota>metazoa>chordata>vertebrata	Mus	musculus	protein S100-A6 [Mus musculus]
NP_038678.1	89	60B8Ag,AI323541,B8Ag,Caga,CFAg,CP-10,MRP8,p8	eukaryota>metazoa>chordata>vertebrata	Mus	musculus	protein S100-A8 [Mus musculus]
CAG10836.1	87	GSTEN:00032585:G:001	eukaryota>metazoa>chordata>vertebrata>actinopterygii	Tetraodon	nigroviridis	unnamed protein product [Tetraodon nigroviridis]
NP_001188085.1	87	s10ab	eukaryota>metazoa>chordata>vertebrata>actinopterygii	Ictalurus	punctatus	s100-a11 [Ictalurus punctatus]
XP_003969336.1	86	LOC101064776	eukaryota>metazoa>chordata>vertebrata>actinopterygii	Takifugu	rubripes	PREDICTED: protein S100-A13-like [Takifugu rubripes]
CAF87358.1	85	GSTEN:00038360:G:001	eukaryota>metazoa>chordata>vertebrata>actinopterygii	Tetraodon	nigroviridis	unnamed protein product [Tetraodon nigroviridis]
NP_001269112.1	85	si:dkey-105n5.1	eukaryota>metazoa>chordata>vertebrata>actinopterygii	Danio	rerio	protein S100-A11 [Danio rerio]
XP_017207662.1	85	si:dkey-105n5.1	eukaryota>metazoa>chordata>vertebrata>actinopterygii	Danio	rerio	PREDICTED: protein S100-A11 isoform X1 [Danio rerio]
CBY12809.1	81	GSOID_T00002868001	eukaryota>metazoa>chordata	Oikopleura	dioica	unnamed protein product [Oikopleura dioica]
CBY12423.1	80	GSOID_T00001792001	eukaryota>metazoa>chordata	Oikopleura	dioica	unnamed protein product [Oikopleura dioica]
NP_004048.1	79	CABP,CABP1,CABP9K,CALB3	eukaryota>metazoa>chordata>vertebrata	Homo	sapiens	protein S100-G [Homo sapiens]
NP_033919.1	79	AU042539,CaBP-D9K,CABP1,Cabp9k,Calb3	eukaryota>metazoa>chordata>vertebrata	Mus	musculus	protein S100-G [Mus musculus]
XP_003261140.1	79	S100G	eukaryota>metazoa>chordata>vertebrata	Nomascus	leucogenys	PREDICTED: protein S100-G [Nomascus leucogenys]
XP_005593108.1	79	S100G	eukaryota>metazoa>chordata>vertebrata	Macaca	fascicularis	PREDICTED: protein S100-G [Macaca fascicularis]
XP_016885330.1	79	CABP,CABP1,CABP9K,CALB3	eukaryota>metazoa>chordata>vertebrata	Homo	sapiens	PREDICTED: protein S100-G isoform X1 [Homo sapiens]

III. Accession numbers for prokaryotic relatives of proteins and protein families key to calcium storage and signaling, with their phylogenies

A. Accession numbers for selected calmodulin-like proteins with domain fusions

Accession numbers of proteins containing a calmoudlin-like EF-hand fused to some additional domains are provided below in clusters which correspond to the different fused domains discussed. Accession numbers are further annotated by the following columns in order from left to right: length, GenBank gene name, phylogeny, organism, and GenBank defline.

i. Fusions to Tic110-like domains

ADB95776.1	797	UCYN_11030	bacteria>cyanobacteria	Candidatus Atelocyanobacterium thalassa isolate ALOHA	Ca2+-binding protein (EF-Hand superfamily) [Candidatus Atelocyanobacterium thalassa isolate ALOHA]
KFF41613.1	812	ucyna2_00487	bacteria>cyanobacteria	Candidatus Atelocyanobacterium thalassa isolate SIO64986	Ca2+-binding protein (EF-Hand superfamily) [Candidatus Atelocyanobacterium thalassa isolate SIO64986]
ACB51194.1	815	cce_1844	bacteria>cyanobacteria	Cyanothece sp. ATCC 51142	unknown [Cyanothece sp. ATCC 51142]
ACK71329.1	802	PCC7424_2925	bacteria>cyanobacteria	Cyanothece sp. PCC 7424	putative signal transduction protein with EFhand domain [Cyanothece sp. PCC 7424]
ACU99706.1	815	Cyan8802_0768	bacteria>cyanobacteria	Cyanothece sp. PCC 8802	putative signal transduction protein with EFhand domain [Cyanothece sp. PCC 8802]
AFY37740.1	727	Lepto7376_1390	bacteria>cyanobacteria	Leptolyngbya sp. PCC 7376	EF hand repeat-containing protein [Leptolyngbya sp. PCC 7376]
AOC54009.1	723	amyaer_3304	bacteria>cyanobacteria	Microcystis aeruginosa NIES-2481	cAMP-binding protein [Microcystis aeruginosa NIES-2481]
AKE65596.1	723	MYAER_3258	bacteria>cyanobacteria	Microcystis aeruginosa NIES-2549	cAMP-binding protein [Microcystis aeruginosa NIES-2549]
BAG02189.1	723	MAE_23670	bacteria>cyanobacteria	Microcystis aeruginosa NIES-843	hypothetical protein MAE_23670 [Microcystis aeruginosa NIES-843]
ARI81737.1	723	BH695_2457	bacteria>cyanobacteria	Microcystis aeruginosa PCC 7806SL	hypothetical protein BH695_2457 [Microcystis aeruginosa PCC 7806SL]
AKV68896.1	723	VL20_3943	bacteria>cyanobacteria	Microcystis panniformis FACHB-1757	cAMP-binding protein [Microcystis panniformis FACHB-1757]
ABG51286.1	716	Tery_2045	bacteria>cyanobacteria	Trichodesmium erythraeum IMS101	putative signal transduction protein with EFhand domain [Trichodesmium erythraeum IMS101]
XP_011613778.1	196	cabp1	eukaryota>metazoa>chordata>vertebrata>actinopterygii	Takifugu rubripes	PREDICTED: calcium-binding protein 1 [Takifugu rubripes]

ii. Fusions to Heme-Oxygenase domains

ARF79381.1	455	B6264_11010	bacteria>actinobacteria Kitasatospora aureofaciens	calcium-binding protein [Kitasatospora aureofaciens]
BAJ33050.1	471	KSE_72950	bacteria>actinobacteria Kitasatospora setae KM-6054	hypothetical protein KSE_72950 [Kitasatospora setae KM-6054]
CCH31773.1	55	BN6_44930	bacteria>actinobacteria Saccharothrix espanaensis DSM 44229	hypothetical protein BN6_44930 [Saccharothrix espanaensis DSM 44229]
AIA07901.1	478	DC74_7481	bacteria>actinobacteria Streptomyces albulus	hypothetical protein DC74_7481 [Streptomyces albulus]
ANB04067.1	90	SAM40697_0104	bacteria>actinobacteria Streptomyces ambofaciens	calcium-binding protein [Streptomyces ambofaciens]
ANB10773.1	90	SAM40697_6821	bacteria>actinobacteria Streptomyces ambofaciens	calcium-binding protein [Streptomyces ambofaciens]
AKZ53175.1	89	SAM23877_0126	bacteria>actinobacteria Streptomyces ambofaciens ATCC 23877	putative calcium binding domain [Streptomyces ambofaciens ATCC 23877]
AKZ60588.1	89	SAM23877_7547	bacteria>actinobacteria Streptomyces ambofaciens ATCC 23877	putative calcium binding domain [Streptomyces ambofaciens ATCC 23877]
ANW19406.1	467	BB341_14850	bacteria>actinobacteria Streptomyces clavuligerus	calcium-binding protein [Streptomyces clavuligerus]
AGS67447.1	02	B446_03075	bacteria>actinobacteria Streptomyces collinus Tu 365	calcium binding protein [Streptomyces collinus Tu 365]
AGK81907.1	89	SFUL_7030	bacteria>actinobacteria Streptomyces fulvissimus DSM 40593	Putative calcium binding protein [Streptomyces fulvissimus DSM 40593]
AIR96185.1	03	SGLAU_00785	bacteria>actinobacteria Streptomyces glaucescens	putative thiopeptide-lantipeptide biosynthesis related protein [Streptomyces glaucescens]
AQW54393.1	87	SHXM_07856	bacteria>actinobacteria Streptomyces hygroscopicus	calcium-binding protein [Streptomyces hygroscopicus]
AEY93552.1	90	SHJG_8286	bacteria>actinobacteria Streptomyces hygroscopicus subsp. jinggangensis 5008	putative calcium binding protein [Streptomyces hygroscopicus subsp. jinggangensis 5008]
AGF67709.1	90	SHJGH_8047	bacteria>actinobacteria Streptomyces hygroscopicus subsp. jinggangensis TL01	putative calcium binding protein [Streptomyces hygroscopicus subsp. jinggangensis TL01]
ALO98209.1	90	SHL15_7196	bacteria>actinobacteria Streptomyces hygroscopicus subsp. limoneus	Putative calcium binding protein [Streptomyces hygroscopicus subsp. limoneus]
AQU65409.1	89	BBN63_03260	bacteria>actinobacteria Streptomyces niveus	calcium-binding protein [Streptomyces niveus]
ANZ20997.1	479	SNOUR_38845	bacteria>actinobacteria Streptomyces noursei ATCC 11455	hypothetical protein SNOUR_38845 [Streptomyces noursei ATCC 11455]
CUW25837.1	93	TUE45_00547	bacteria>actinobacteria Streptomyces reticuli	EF hand [Streptomyces reticuli]
AJC61110.1	479	GZL_08582	bacteria>actinobacteria Streptomyces sp. 769	hypothetical protein GZL_08582 [Streptomyces sp. 769]
AKL70893.1	470	M444_36785	bacteria>actinobacteria Streptomyces sp. Mg1	calcium-binding protein (plasmid) [Streptomyces sp. Mg1]
AKG43895.1	80	SXIM_25110	bacteria>actinobacteria Streptomyces xiamenensis	calcium binding protein [Streptomyces xiamenensis]

iii. Fusions to NO synthase domains

AFZ12720.1	1195	Cri9333_1836	bacteria>cyanobacteria	Crinalium epipsammum PCC 9333	Nitric oxide synthase NOS [Crinalium epipsammum PCC 9333]
AFZ21309.1	1186	Mic7113_5682	bacteria>cyanobacteria	Microcoleus sp. PCC 7113	nitric oxide synthase, oxygenase domain protein [Microcoleus sp. PCC 7113]
CAN91740.1	1163	nos	bacteria>proteobacteria>deltaproteobacteria	Sorangium cellulosum So ce56	Nitric-oxide synthase, oxygenase subunit [Sorangium cellulosum So ce56]
AGP34833.1	1151	SCE1572_10085	bacteria>proteobacteria>deltaproteobacteria	Sorangium cellulosum So0157-2	hypothetical protein SCE1572_10085 [Sorangium cellulosum So0157-2]

iv. Fusions to NADPH oxidase domains

OUW79836.1	492	CBD77_01470	bacteria	bacterium TMED217	hypothetical protein CBD77_01470 [bacterium TMED217]
OUU31996.1	495	CBC06_01230	bacteria	bacterium TMED46	hypothetical protein CBC06_01230 [bacterium TMED46]
OUV05729.1	492	CBC40_01300	bacteria	bacterium TMED80	hypothetical protein CBC40_01300 [bacterium TMED80]
ABW28148.1	659	AM1_3152	bacteria>cyanobacteria	Acaryochloris marina MBIC11017	oxidoreductase, putative [Acaryochloris marina MBIC11017]
OIP70026.1	669	AUK43_10790	bacteria>cyanobacteria	Oscillatoriales cyanobacterium CG2_30_40_61	ferric reductase [Oscillatoriales cyanobacterium CG2_30_40_61]
XP_019850316.1	926	LOC100638974	eukaryota>metazoa	Amphimedon queenslandica	PREDICTED: NADPH oxidase 5-like [Amphimedon queenslandica]
XP_002407725.1	1532	IscW_ISCW007865	eukaryota>metazoa	Ixodes scapularis	dual oxidase 1, putative [Ixodes scapularis]
XP_002410996.1	727	IscW_ISCW002630	eukaryota>metazoa	Ixodes scapularis	NADPH oxidase, putative, partial [Ixodes scapularis]
XP_022253299.1	956	LOC106469164	eukaryota>metazoa	Limulus polyphemus	NADPH oxidase 5-like [Limulus polyphemus]
XP_022253297.1	956	LOC106469164	eukaryota>metazoa	Limulus polyphemus	NADPH oxidase 5-like [Limulus polyphemus]
XP_013785082.1	956	LOC106469164	eukaryota>metazoa	Limulus polyphemus	NADPH oxidase 5-like [Limulus polyphemus]
XP_013793617.1	1498	LOC106477616	eukaryota>metazoa	Limulus polyphemus	dual oxidase-like [Limulus polyphemus]
XP_013772431.1	1451	LOC106457545	eukaryota>metazoa	Limulus polyphemus	dual oxidase-like [Limulus polyphemus]
XP_022235000.1	1004	LOC106475085	eukaryota>metazoa	Limulus polyphemus	dual oxidase-like, partial [Limulus polyphemus]
XP_013789730.1	703	LOC106473593	eukaryota>metazoa	Limulus polyphemus	NADPH oxidase 5-like [Limulus polyphemus]
NP_001171708.1	737	NOX5	eukaryota>metazoa>chordata>vertebrata	Homo sapiens	NADPH oxidase 5 isoform 2 [Homo sapiens]
NP_001171709.1	730	NOX5	eukaryota>metazoa>chordata>vertebrata	Homo sapiens	NADPH oxidase 5 isoform 3 [Homo sapiens]
XP_005559972.1	737	NOX5	eukaryota>metazoa>chordata>vertebrata	Macaca fascicularis	PREDICTED: NADPH oxidase 5 isoform X2 [Macaca fascicularis]
XP_005559971.1	744	NOX5	eukaryota>metazoa>chordata>vertebrata	Macaca fascicularis	PREDICTED: NADPH oxidase 5 isoform X1 [Macaca fascicularis]
XP_006499173.1	1545	A430065P05Rik,LNOX2,NOXEF2,P138-TOX,THOX2	eukaryota>metazoa>chordata>vertebrata	Mus musculus	PREDICTED: dual oxidase 2 isoform X1 [Mus musculus]
NP_808278.2	1517	A430065P05Rik,LNOX2,NOXEF2,P138-TOX,THOX2	eukaryota>metazoa>chordata>vertebrata	Mus musculus	dual oxidase 2 precursor [Mus musculus]
XP_018106923.1	1466	LOC108710292	eukaryota>metazoa>chordata>vertebrata	Xenopus laevis	PREDICTED: dual oxidase 1-like [Xenopus laevis]
EFX77976.1	1494	DAPPUDRAFT_320872	eukaryota>metazoa>crustacea	Daphnia pulex	hypothetical protein DAPPUDRAFT_320872 [Daphnia pulex]
EFX69361.1	1494	DAPPUDRAFT_329115	eukaryota>metazoa>crustacea	Daphnia pulex	hypothetical protein DAPPUDRAFT_329115 [Daphnia pulex]
XP_011677804.1	988	LOC752031	eukaryota>metazoa>echinodermata	Strongylocentrotus purpuratus	PREDICTED: NADPH oxidase 5 [Strongylocentrotus purpuratus]
XP_797866.3	930	LOC593293	eukaryota>metazoa>echinodermata	Strongylocentrotus purpuratus	PREDICTED: NADPH oxidase 5-like [Strongylocentrotus purpuratus]
XP_011672718.1	823	LOC580939	eukaryota>metazoa>echinodermata	Strongylocentrotus purpuratus	PREDICTED: NADPH oxidase 5-like, partial [Strongylocentrotus purpuratus]
XP_011666295.1	1593	Udx1	eukaryota>metazoa>echinodermata	Strongylocentrotus purpuratus	PREDICTED: dual oxidase 1 isoform X4 [Strongylocentrotus purpuratus]
XP_011666292.1	1723	Udx1	eukaryota>metazoa>echinodermata	Strongylocentrotus purpuratus	PREDICTED: dual oxidase 1 isoform X1 [Strongylocentrotus purpuratus]
NP_001118237.1	1671	Udx1	eukaryota>metazoa>echinodermata	Strongylocentrotus purpuratus	dual oxidase 1 precursor [Strongylocentrotus purpuratus]
XP_011666294.1	1630	Udx1	eukaryota>metazoa>echinodermata	Strongylocentrotus purpuratus	PREDICTED: dual oxidase 1 isoform X3 [Strongylocentrotus purpuratus]
XP_011666293.1	1682	Udx1	eukaryota>metazoa>echinodermata	Strongylocentrotus purpuratus	PREDICTED: dual oxidase 1 isoform X2 [Strongylocentrotus purpuratus]
XP_003242881.1	1175	LOC100165694	eukaryota>metazoa>hexapoda	Acyrthosiphon pisum	PREDICTED: NADPH oxidase 5 isoform X1 [Acyrthosiphon pisum]
XP_008180623.1	1168	LOC100165694	eukaryota>metazoa>hexapoda	Acyrthosiphon pisum	PREDICTED: NADPH oxidase 5 isoform X2 [Acyrthosiphon pisum]
XP_016766869.1	1510	LOC551970	eukaryota>metazoa>hexapoda	Apis mellifera	PREDICTED: dual oxidase isoform X1 [Apis mellifera]
XP_016766868.1	1510	LOC551970	eukaryota>metazoa>hexapoda	Apis mellifera	PREDICTED: dual oxidase isoform X1 [Apis mellifera]
XP_006560557.1	1510	LOC551970	eukaryota>metazoa>hexapoda	Apis mellifera	PREDICTED: dual oxidase isoform X1 [Apis mellifera]
XP_006560554.1	1510	LOC551970	eukaryota>metazoa>hexapoda	Apis mellifera	PREDICTED: dual oxidase isoform X1 [Apis mellifera]
XP_006560553.1	1510	LOC551970	eukaryota>metazoa>hexapoda	Apis mellifera	PREDICTED: dual oxidase isoform X1 [Apis mellifera]
XP_006560555.1	1466	LOC551970	eukaryota>metazoa>hexapoda	Apis mellifera	PREDICTED: dual oxidase isoform X2 [Apis mellifera]
XP_016771540.1	1033	LOC408451	eukaryota>metazoa>hexapoda	Apis mellifera	PREDICTED: NADPH oxidase 5 isoform X2 [Apis mellifera]
XP_006562353.1	1033	LOC408451	eukaryota>metazoa>hexapoda	Apis mellifera	PREDICTED: NADPH oxidase 5 isoform X2 [Apis mellifera]
XP_016771539.1	1084	LOC408451	eukaryota>metazoa>hexapoda	Apis mellifera	PREDICTED: NADPH oxidase 5 isoform X1 [Apis mellifera]
XP_021207539.1	1307	LOC101742234	eukaryota>metazoa>hexapoda	Bombyx mori	dual oxidase-like isoform X2 [Bombyx mori]
XP_021202445.1	1130	LOC101742585	eukaryota>metazoa>hexapoda	Bombyx mori	NADPH oxidase 5 [Bombyx mori]
XP_021202444.1	1130	LOC101742585	eukaryota>metazoa>hexapoda	Bombyx mori	NADPH oxidase 5 [Bombyx mori]
NP_001266413.1	1500	LOC101742234	eukaryota>metazoa>hexapoda	Bombyx mori	dual oxidase-like precursor [Bombyx mori]
XP_021207538.1	1321	LOC101742234	eukaryota>metazoa>hexapoda	Bombyx mori	dual oxidase-like isoform X1 [Bombyx mori]
XP_001844503.1	1482	CpipJ_CPIJ003117	eukaryota>metazoa>hexapoda	Culex quinquefasciatus	dual oxidase 1 [Culex quinquefasciatus]
NP_608715.2	1537	CG3131,Cy,dduox,dDuox,Dmel\CG3131,duox,DUOX,l(2)23Bb	eukaryota>metazoa>hexapoda	Drosophila melanogaster	dual oxidase, isoform B [Drosophila melanogaster]
NP_001259968.1	1537	CG3131,Cy,dduox,dDuox,Dmel\CG3131,duox,DUOX,l(2)23Bb	eukaryota>metazoa>hexapoda	Drosophila melanogaster	dual oxidase, isoform C [Drosophila melanogaster]
NP_001286485.1	1282	CG15924,CG34399,CG3896,Dmel\CG34399,dnox,dNox,NOX-1,Nox1	eukaryota>metazoa>hexapoda	Drosophila melanogaster	NADPH oxidase, isoform B [Drosophila melanogaster]
NP_611116.2	1340	CG15924,CG34399,CG3896,Dmel\CG34399,dnox,dNox,NOX-1,Nox1	eukaryota>metazoa>hexapoda	Drosophila melanogaster	NADPH oxidase, isoform E [Drosophila melanogaster]
NP_001286486.1	1340	CG15924,CG34399,CG3896,Dmel\CG34399,dnox,dNox,NOX-1,Nox1	eukaryota>metazoa>hexapoda	Drosophila melanogaster	NADPH oxidase, isoform D [Drosophila melanogaster]
NP_001097336.1	1340	CG15924,CG34399,CG3896,Dmel\CG34399,dnox,dNox,NOX-1,Nox1	eukaryota>metazoa>hexapoda	Drosophila melanogaster	NADPH oxidase, isoform C [Drosophila melanogaster]
XP_011152754.1	985	LOC105191215	eukaryota>metazoa>hexapoda	Harpegnathos saltator	PREDICTED: dual oxidase, partial [Harpegnathos saltator]
XP_011139577.1	1117	LOC105183263	eukaryota>metazoa>hexapoda	Harpegnathos saltator	PREDICTED: NADPH oxidase 5 [Harpegnathos saltator]
XP_011139567.1	1117	LOC105183263	eukaryota>metazoa>hexapoda	Harpegnathos saltator	PREDICTED: NADPH oxidase 5 [Harpegnathos saltator]
XP_011139560.1	1117	LOC105183263	eukaryota>metazoa>hexapoda	Harpegnathos saltator	PREDICTED: NADPH oxidase 5 [Harpegnathos saltator]
XP_972375.2	1137	LOC661099	eukaryota>metazoa>hexapoda	Tribolium castaneum	PREDICTED: NADPH oxidase 5 isoform X2 [Tribolium castaneum]
XP_008193792.1	1167	LOC661099	eukaryota>metazoa>hexapoda	Tribolium castaneum	PREDICTED: NADPH oxidase 5 isoform X1 [Tribolium castaneum]
XP_015836989.1	940	LOC661099	eukaryota>metazoa>hexapoda	Tribolium castaneum	PREDICTED: superoxide-generating NADPH oxidase heavy chain subunit C isoform X4 [Tribolium castaneum]
XP_008193793.1	980	LOC661099	eukaryota>metazoa>hexapoda	Tribolium castaneum	PREDICTED: superoxide-generating NADPH oxidase heavy chain subunit C isoform X3 [Tribolium castaneum]
XP_008193794.1	934	LOC661099	eukaryota>metazoa>hexapoda	Tribolium castaneum	PREDICTED: superoxide-generating NADPH oxidase heavy chain subunit C isoform X5 [Tribolium castaneum]
XP_008191438.1	1499	LOC659451	eukaryota>metazoa>hexapoda	Tribolium castaneum	PREDICTED: dual oxidase isoform X2 [Tribolium castaneum]
XP_008191437.1	1515	LOC659451	eukaryota>metazoa>hexapoda	Tribolium castaneum	PREDICTED: dual oxidase isoform X1 [Tribolium castaneum]
XP_015834049.1	1447	LOC659451	eukaryota>metazoa>hexapoda	Tribolium castaneum	PREDICTED: dual oxidase isoform X3 [Tribolium castaneum]
XP_019924043.1	1534	LOC105331097	eukaryota>metazoa>mollusca	Crassostrea gigas	PREDICTED: dual oxidase [Crassostrea gigas]
XP_019924044.1	1534	LOC105331097	eukaryota>metazoa>mollusca	Crassostrea gigas	PREDICTED: dual oxidase [Crassostrea gigas]
XP_019924045.1	1534	LOC105331097	eukaryota>metazoa>mollusca	Crassostrea gigas	PREDICTED: dual oxidase [Crassostrea gigas]
XP_019924046.1	1534	LOC105331097	eukaryota>metazoa>mollusca	Crassostrea gigas	PREDICTED: dual oxidase [Crassostrea gigas]

v. Fusions to cNMP-binding domains

AHF92076.1	766	OPIT5_19400	bacteria>verrucomicrobia	Opitutaceae bacterium TAV5	Crp/Fnr family transcriptional regulator [Opitutaceae bacterium TAV5]
XP_013754210.1	1020	AMSG_09767	eukaryota>apusozoa>apusomonadidae	Thecamonas trahens ATCC 50062	cyclic nucleotide-binding protein [Thecamonas trahens ATCC 50062]
XP_002184432.1	621	PHATRDRAFT_49634	eukaryota>stramenopiles	Phaeodactylum tricornutum CCAP 1055/1	predicted protein [Phaeodactylum tricornutum CCAP 1055/1]
XP_002508260.1	2382	MICPUN_60802	eukaryota>viridiplantae>chlorophyta	Micromonas commoda	ankyrin repeat protein [Micromonas commoda]
XP_003058949.1	2524	MICPUCDRAFT_58266	eukaryota>viridiplantae>chlorophyta	Micromonas pusilla CCMP1545	ankyrin repeat protein [Micromonas pusilla CCMP1545]
XP_013902851.1	583	MNEG_4122	eukaryota>viridiplantae>chlorophyta	Monoraphidium neglectum	hypothetical protein MNEG_4122 [Monoraphidium neglectum]

vi. Fusions to thioredoxin and/or sulfatase domains

AMV19056.1	553	VT03_14290	bacteria>planctomycetes	Planctomyces sp. SH-PL14	Arylsulfatase [Planctomyces sp. SH-PL14]
AMV19472.1	575	betC_4	bacteria>planctomycetes	Planctomyces sp. SH-PL14	Choline-sulfatase [Planctomyces sp. SH-PL14]
ADY61367.1	980	Plabr_3778	bacteria>planctomycetes	Rubinisphaera brasiliensis DSM 5305	Arylsulfatase [Rubinisphaera brasiliensis DSM 5305]
OYW31404.1	554	B7Z47_01190	bacteria>verrucomicrobia	Chthoniobacter sp. 12-60-6	iduronate-2-sulfatase [Chthoniobacter sp. 12-60-6]
OYW29661.1	371	B7Z47_04520	bacteria>verrucomicrobia	Chthoniobacter sp. 12-60-6	hypothetical protein B7Z47_04520, partial [Chthoniobacter sp. 12-60-6]
KRP37226.1	607	ABS34_03795	bacteria>verrucomicrobia	Opitutaceae bacterium BACL24 MAG-120322-bin51	hypothetical protein ABS34_03795 [Opitutaceae bacterium BACL24 MAG-120322-bin51]
PAW62905.1	742	B9S34_15375	bacteria>verrucomicrobia	Opitutae bacterium Tous-C1TDCM	hypothetical protein B9S34_15375 [Opitutae bacterium Tous-C1TDCM]
OYW77921.1	553	B7Z37_01760	bacteria>verrucomicrobia	Verrucomicrobia bacterium 12-59-8	iduronate-2-sulfatase [Verrucomicrobia bacterium 12-59-8]
OYW74718.1	753	B7Z37_16615	bacteria>verrucomicrobia	Verrucomicrobia bacterium 12-59-8	histidine kinase [Verrucomicrobia bacterium 12-59-8]
PAW68096.1	827	B9S38_11645	bacteria>verrucomicrobia	Verrucomicrobiae bacterium Tous-C4TDCM	hypothetical protein B9S38_11645 [Verrucomicrobiae bacterium Tous-C4TDCM]
XP_005787975.1	658	EMIHUDRAFT_462481	eukaryota>haptophyceae	Emiliania huxleyi CCMP1516	hypothetical protein EMIHUDRAFT_462481 [Emiliania huxleyi CCMP1516]
EJK62482.1	643	THAOC_16904	eukaryota>stramenopiles	Thalassiosira oceanica	hypothetical protein THAOC_16904 [Thalassiosira oceanica]
XP_001693206.1	358	CHLREDRAFT_205510	eukaryota>viridiplantae>chlorophyta	Chlamydomonas reinhardtii	EF-Hand domain-containing thioredoxin [Chlamydomonas reinhardtii]
XP_005845968.1	365	CHLNCDRAFT_135979	eukaryota>viridiplantae>chlorophyta	Chlorella variabilis	hypothetical protein CHLNCDRAFT_135979 [Chlorella variabilis]
XP_005652290.1	393	COCSUDRAFT_64362	eukaryota>viridiplantae>chlorophyta	Coccomyxa subellipsoidea C-169	EF-hand [Coccomyxa subellipsoidea C-169]
XP_002952510.1	434	VOLCADRAFT_105559	eukaryota>viridiplantae>chlorophyta	Volvox carteri f. nagariensis	hypothetical protein VOLCADRAFT_105559 [Volvox carteri f. nagariensis]

vii. Fusions to glyoxalase domains

AKJ65436.1	1393	L21SP4_02209	bacteria	Kiritimatiella glycovorans	esterase, PHB depolymerase family [Kiritimatiella glycovorans]
EJK50743.1	916	THAOC_30164	eukaryota>stramenopiles	Thalassiosira oceanica	hypothetical protein THAOC_30164 [Thalassiosira oceanica]

B. Accession numbers for selected bacterial PPIases

Accession numbers of bacterial FKBP-type PPIases are provided below. Accession numbers are further annotated by the following columns in order from left to right: length, GenBank gene name, phylogeny, organism, and GenBank defline. Sequences were found with a BLAST search using calstabin (FKBP1A) as the query.

KPQ36951.1	209	HLUCCA11_03280	bacteria>cyanobacteria	Phormidesmis priestleyi Ana	peptidylprolyl isomerase [Phormidesmis priestleyi Ana].
ASC74076.1	206	fbp_2	bacteria>cyanobacteria	Halomicronema hongdechloris C2206	FK506-binding protein [Halomicronema hongdechloris C2206].
ABG50757.1	203	Tery_1467	bacteria>cyanobacteria	Trichodesmium erythraeum IMS101	Peptidylprolyl isomerase [Trichodesmium erythraeum IMS101].
OKY73001.1	198	BJG00_02970	bacteria>cyanobacteria	Limnothrix sp. CACIAM 69d	peptidylprolyl isomerase [Limnothrix sp. CACIAM 69d].
AFZ07964.1	195	Osc7112_3600	bacteria>cyanobacteria	Oscillatoria nigro-viridis PCC 7112	Peptidylprolyl isomerase [Oscillatoria nigro-viridis PCC 7112].
BAU41942.1	193	fbp_1	bacteria>cyanobacteria	Leptolyngbya sp. O-77	FK506-binding protein [Leptolyngbya sp. O-77].
KPQ32092.1	192	HLUCCO16_20745	bacteria>cyanobacteria	Phormidium sp. OSCR	peptidylprolyl isomerase [Phormidium sp. OSCR].
ACB52376.1	188	cce_3028	bacteria>cyanobacteria	Crocosphaera subtropica ATCC 51142	FKBP-type peptidyl-prolyl cis-trans isomerase [Crocosphaera subtropica ATCC 51142].
AFY82996.1	188	Osci16304_3427	bacteria>cyanobacteria	Oscillatoria acuminata PCC 6304	FKBP-type peptidyl-prolyl cis-trans isomerase [Oscillatoria acuminata PCC 6304].
OIP67500.1	187	AUK43_19160	bacteria>cyanobacteria	Oscillatoriales cyanobacterium CG2_30_40_61	peptidylprolyl isomerase [Oscillatoriales cyanobacterium CG2_30_40_61].
AFZ21787.1	186	Mic7113_6195	bacteria>cyanobacteria	Microcoleus sp. PCC 7113	FKBP-type peptidyl-prolyl cis-trans isomerase [Microcoleus sp. PCC 7113].
AFZ54885.1	185	Cyan10605_2818	bacteria>cyanobacteria	Cyanobacterium aponinum PCC 10605	Peptidylprolyl isomerase [Cyanobacterium aponinum PCC 10605].
AFY65154.1	185	GEI7407_0656	bacteria>cyanobacteria	Geitlerinema sp. PCC 7407	Peptidylprolyl isomerase [Geitlerinema sp. PCC 7407].
AOX01819.1	185	BJP34_22405	bacteria>cyanobacteria	Moorea producens PAL-8-15-08-1	peptidylprolyl isomerase [Moorea producens PAL-8-15-08-1].
AFZ35513.1	185	Sta7437_1959	bacteria>cyanobacteria	Stanieria cyanosphaera PCC 7437	peptidylprolyl isomerase FKBP-type [Stanieria cyanosphaera PCC 7437].
AFY69165.1	183	Pse7367_0865	bacteria>cyanobacteria	Pseudanabaena sp. PCC 7367	peptidylprolyl isomerase [Pseudanabaena sp. PCC 7367].
AFY89428.1	182	Chro_4024	bacteria>cyanobacteria	Chroococcidiopsis thermalis PCC 7203	peptidylprolyl isomerase FKBP-type [Chroococcidiopsis thermalis PCC 7203].
AFZ46112.1	182	Cyast_0129	bacteria>cyanobacteria	Cyanobacterium stanieri PCC 7202	Peptidylprolyl isomerase [Cyanobacterium stanieri PCC 7202].
ADN16288.1	181	Cyan7822_4374	bacteria>cyanobacteria	Gloeothece verrucosa PCC 7822	Peptidylprolyl isomerase [Gloeothece verrucosa PCC 7822].
AKG20234.1	180	IJ00_01945	bacteria>cyanobacteria	Calothrix sp. 336/3	peptidylprolyl isomerase [Calothrix sp. 336/3].
AFZ13876.1	180	Cri9333_3037	bacteria>cyanobacteria	Crinalium epipsammum PCC 9333	peptidylprolyl isomerase FKBP-type [Crinalium epipsammum PCC 9333].
ACV02024.1	180	Cyan8802_3186	bacteria>cyanobacteria	Rippkaea orientalis PCC 8802	Peptidylprolyl isomerase [Rippkaea orientalis PCC 8802].
AFY56749.1	180	Riv7116_4321	bacteria>cyanobacteria	Rivularia sp. PCC 7116	FKBP-type peptidyl-prolyl cis-trans isomerase [Rivularia sp. PCC 7116].
ACK66970.1	179	PCC7424_0506	bacteria>cyanobacteria	Gloeothece citriformis PCC 7424	peptidylprolyl isomerase FKBP-type [Gloeothece citriformis PCC 7424].
ABW26812.1	177	AM1_1791	bacteria>cyanobacteria	Acaryochloris marina MBIC11017	peptidylprolyl isomerase, FKBP type [Acaryochloris marina MBIC11017].
AFZ32915.1	177	Glo7428_4473	bacteria>cyanobacteria	Gloeocapsa sp. PCC 7428	Peptidylprolyl isomerase [Gloeocapsa sp. PCC 7428].
AFY76487.1	177	Ple7327_1073	bacteria>cyanobacteria	Pleurocapsa sp. PCC 7327	FKBP-type peptidyl-prolyl cis-trans isomerase [Pleurocapsa sp. PCC 7327].
AFZ58822.1	176	Anacy_3421	bacteria>cyanobacteria	Anabaena cylindrica PCC 7122	Peptidylprolyl isomerase [Anabaena cylindrica PCC 7122].
AFY99589.1	176	Cal6303_0515	bacteria>cyanobacteria	Calothrix sp. PCC 6303	peptidylprolyl isomerase FKBP-type [Calothrix sp. PCC 6303].
BAU04625.1	174	FIS3754_05130	bacteria>cyanobacteria	Fischerella sp. NIES-3754	peptidylprolyl isomerase FKBP-type [Fischerella sp. NIES-3754].
AFY48068.1	174	Nos7524_2223	bacteria>cyanobacteria	Nostoc sp. PCC 7524	FKBP-type peptidyl-prolyl cis-trans isomerase [Nostoc sp. PCC 7524].
ARV57896.1	174	BZZ01_03930	bacteria>cyanobacteria	Nostocales cyanobacterium HT-58-2	peptidylprolyl isomerase [Nostocales cyanobacterium HT-58-2].
BAD79939.1	174	syc1749_d	bacteria>cyanobacteria	Synechococcus elongatus PCC 6301	FKBP-type peptidyl-prolyl cis-trans isomerase [Synechococcus elongatus PCC 6301].
ABB58384.1	174	Synpcc7942_2354	bacteria>cyanobacteria	Synechococcus elongatus PCC 7942	Peptidylprolyl isomerase [Synechococcus elongatus PCC 7942].
AFY36351.1	173	Cal7507_6047	bacteria>cyanobacteria	Calothrix sp. PCC 7507	Peptidylprolyl isomerase [Calothrix sp. PCC 7507].
AFY44255.1	173	Nos7107_3690	bacteria>cyanobacteria	Nostoc sp. PCC 7107	Peptidylprolyl isomerase [Nostoc sp. PCC 7107].
OIP78707.1	173	AUK48_01095	bacteria>cyanobacteria	Oscillatoriales cyanobacterium CG2_30_44_21	peptidylprolyl isomerase [Oscillatoriales cyanobacterium CG2_30_44_21].
KKS48935.1	172	UV11_C0001G0030	bacteria	Candidatus Giovannonibacteria bacterium GW2011_GWF2_42_19	Peptidyl-prolyl cis-trans isomerase [Candidatus Giovannonibacteria bacterium GW2011_GWF2_42_19].
OCY14293.1	172	BEV12_19805	bacteria>cyanobacteria	Microcystis aeruginosa CACIAM 03	peptidylprolyl isomerase [Microcystis aeruginosa CACIAM 03].
AFZ49582.1	171	Dacsa_0835	bacteria>cyanobacteria	Dactylococcopsis salina PCC 8305	FKBP-type peptidyl-prolyl cis-trans isomerase [Dactylococcopsis salina PCC 8305].
AFZ45554.1	171	PCC7418_3440	bacteria>cyanobacteria	Halothece sp. PCC 7418	Peptidylprolyl isomerase [Halothece sp. PCC 7418].
AKE64896.1	169	MYAER_2554	bacteria>cyanobacteria	Microcystis aeruginosa NIES-2549	FKBP-type peptidyl-prolyl cis-trans isomerase [Microcystis aeruginosa NIES-2549].
BAG05260.1	169	ytfC	bacteria>cyanobacteria	Microcystis aeruginosa NIES-843	FKBP-type peptidyl-prolyl cis-trans isomerase [Microcystis aeruginosa NIES-843].
ARI82550.1	169	BH695_3271	bacteria>cyanobacteria	Microcystis aeruginosa PCC 7806SL	hypothetical protein BH695_3271 [Microcystis aeruginosa PCC 7806SL].
AKV71136.1	169	VL20_6402	bacteria>cyanobacteria	Microcystis panniformis FACHB-1757	FKBP-type peptidyl-prolyl cis-trans isomerase [Microcystis panniformis FACHB-1757].
ADI64722.1	167	Aazo_2895	bacteria>cyanobacteria	'Nostoc azollae' 0708	Peptidylprolyl isomerase ['Nostoc azollae' 0708].
BAB72535.1	165	alr0577	bacteria>cyanobacteria	Nostoc sp. PCC 7120	FKBP-type peptidyl-prolyl cis-trans isomerase [Nostoc sp. PCC 7120].
AFY73302.1	165	Syn7502_01199	bacteria>cyanobacteria	Synechococcus sp. PCC 7502	FKBP-type peptidyl-prolyl cis-trans isomerase [Synechococcus sp. PCC 7502].
ABA19957.1	165	Ava_0331	bacteria>cyanobacteria	Trichormus variabilis ATCC 29413	Peptidylprolyl isomerase, FKBP-type [Trichormus variabilis ATCC 29413].
OIO94955.1	163	AUJ92_08850	bacteria>armatimonadetes	Armatimonadetes bacterium CG2_30_59_28	hypothetical protein AUJ92_08850 [Armatimonadetes bacterium CG2_30_59_28].
OBQ38831.1	163	AN487_06305	bacteria>cyanobacteria	Anabaena sp. CRKS33	peptidylprolyl isomerase [Anabaena sp. CRKS33].
BAT53155.1	163	NOS3756_21140	bacteria>cyanobacteria	Nostoc sp. NIES-3756	FKBP-type peptidyl-prolyl cis-trans isomerase [Nostoc sp. NIES-3756].
BAC88782.1	161	glr0841	bacteria>cyanobacteria	Gloeobacter violaceus PCC 7421	FKBP-type peptidyl-prolyl cis-trans isomerase [Gloeobacter violaceus PCC 7421].
KUK49877.1	159	XD75_0148	bacteria	Parcubacteria bacterium 33_209	Peptidyl-prolyl cis-trans isomerase [Parcubacteria bacterium 33_209].

[illegible]

-4)[&bootstrap=1.0]:1.88852,('TRIADDRAFT_16057__Trichoplax_adhaerens__XP_002113334.1':1.5399,(((((((('CAMK1G__Homo_sapiens__XP_016857355.1':0.01784,('Camk1g__Mus_musculus__XP_011237234.1':5.5E-4,'Camk1g__Mus_musculus__NP_659066.1':5.5E-4)[&bootstrap=0.708]:0.00504)[&bootstrap=1.0]:0.26485,(('camk1gb__Danio_rerio__XP_005174188.2':0.0,'camk1gb__Danio_re [illegible]

D. ORAI and Jiraiya

(((((('GUITHDRAFT_153454__Guillardia_theta__XP_005829825.1':1.39278,'Esi_0050_0029__Ectocarpus_siliculosus__CBJ26914.1':1.07151)[&bootstrap=0.848]:0.7041,((('A3Q56_02405__Intoshia_linei__OAF69866.1':0.98445,(((((('CAOG_00132__Capsaspora_owczarzaki__XP_004365003.1':1.27086,'LOC100634763__Amphimedon_queenslandica__XP_019848729.1':0.87202)[&bootstrap=0.21 [illegible]

V. Alignments

A. Alignment of representative Jiraya and ORAI family members

```
Secondary Structure                     ------HHHHHHHHHHHHHHHHHHHHHHHHHHHHH-----H---HHHH-----------------------HHH----------------- [illegible]
Hsapiens__NP_001177773.1            1   MARSY-GGRVLAAMTLLGIAAVVLAALGAQLLFQ-----LQAGRAELRGLRAEGLGQELGAGPGLPEDAAGT-----------------LLPLA-AAL-AA--LVLVLG-FTCLLLAALCGHLGAELAR [illegible]
Mmusculus__XP_006509768.1           1   MGRSY-GGRVLAAMTLLGIPAAVLVALAAQLLFQ-----LQAGRAELRRVRTDGLHPE------LDPDAGLPEAAAGA-----------LLPLA-TAL-AA--LAQVLG-LSCLLLAALCGHLGAELAR [illegible]
Ggallus__XP_015155607.2             1   MP-SY-PQRTLTVLLLFGTLSAAMALLASSLIFQ-----LPXGADLSGSGPGAVRGA--------LPESAAAV----------------LLPVS-AVL-AA--LCLVLN-VSCLLLCLLHGYFSTELCR [illegible]
Drerio__XP_003201087.1              1   MTHSY-SQRSLVILAFLGILSSIMAVLSVILIFQ-----LQTQQAVIKDSPTF------------VPKDITAV----------------LLPVS-TVL-SA--LSLTLN-LSSVVVCLLHSYFATEICR [illegible]
Tcastaneum__XP_971722.1           104   SKQVG-VKEMLVSLGLLCLVSLLLALLSLIFLLK-----ISPV----------------------TASDIRELLRAEQLTVITAEEYVV--VYEVT-LAL-CA--LTLSLN-LCCLLVCAIQFLFAVKLVKS [illegible]
Lgigantea__XP_009061052.1          76   PADNS-AVKFLYSLAVLCLASLILSALSFQVLIS-----LES-----------------------LEDGLKNTSGDGMLNSSAMYSI----VYEAT-TSL-AT--LVIGLD-LSCLMVCSMQCFFTAKILR [illegible]
Celegans__NP_491975.1              73   MDRKT-VHWNMLLLLFMSVCSALFTVVALQFHFSNAERFLR-----------------------TAKSPASAYRGV-------------IDVIR-DVI-ASFDVAIVILSILAFFVSALQLLFLMKIVKQT [illegible]
Hsapiens__NP_116179.2              74   LSWRK-LYLSRAKLKASSRTSALLSGFAMVAMVE-----VQ------------------------LDADHDYPPG---------------LLIAF-SAC-TT--VLVAVH-LFALMISTCILPNIEAVSNV [illegible]
Mmusculus__NP_780632.1             76   LSWRK-LYLSRAKLKASSRTSALLSGFAMVAMVE-----VQ------------------------LDTDHDYPPG---------------LLIVF-SAC-TT--VLVAVH-LFALMISTCILPNIEAVSNV [illegible]
Ggallus__NP_001025829.1            11   LSWRK-LYLSRAKLKASSRTSALLSGFAMVAMVE-----VQ------------------------LDAEHDYPRG---------------LLIAF-SAC-TT--VLVAVH-LFALMISTCILPNIEAVSNV [illegible]
Drerio__NP_991163.1                13   LSWRK-LYLSRAKLKASSRTSALLSGFAMVAMVE-----VQ------------------------LDTNHDYPPG---------------LLIAF-SAC-TT--VLVAVH-LFALMVSTCILPNIEAVSNV [illegible]
Tcastaneum__XP_008194887.1        136   LSWRK-LHMSRAKLKATATTSELLSGFAMVAMVE-----LQ------------------------INDDTAVPEW---------------LFVIF-AVC-TT--VLVAVH-LFALMISTYILPNIDAVAK [illegible]
Lgigantea__XP_009053888.1          14   LSWRH-LLLSRTKLKRSSRTSALLSGFAMMAIVE-----LE------------------------LDDDTEVPDN---------------LLICF-GVC-TT--LLVFVH-LLALLISTCILPYIEAVSGT [illegible]
Celegans__NP_001254835.1          107   LSEKY-NYDLSRAQLKASSRTSALLAGFAMVCLVE----LQ------------------------YDQSTPKP-----------------LLIVL-GVS-TS--LLVSVH-LLALMMSTCILPYMEAT [illegible]
Epallida__KXJ12979.1               18   LSWRR-LFLTRGKLKASSRTSGLMSGFAMIAMVE-----IQ------------------------LEKGIPEG-----------------LLIAF-SVI-TT--ILISVH-VFALMISVCILPNIESIASLH [illegible]
Tadhaerens__XP_002108336.1          4   IDIAP-VQSTNNIIGSNTTT---------------------N-----------------------CPPGHKCVPLY--------------LLVCF-AVL-TT--LLISVH-LCAVMISTCILPHIDAAES [illegible]
Aqueenslandica__XP_019848729.1      8   SNQLN-CILPVVSLLQVGLV-------------E-----IE------------------------VSPTTPVP-----------------LLIIF-AMH-SA--VLVFIH-LLSLMLATFLLPELEAA [illegible]
Srosetta__XP_004993864.1            6   ARRQ-LALFTHKLEQTGGTSALLSGFALVAMVE-----LN------------------------LPEGRTHVEEFDNNP-----------VFIF-SAV-TS--VLIALH-LFALMVSTSVLPYLKSAIEDIDHHLAQRALTARAKGQALHNLIGPQDTLFDDTTDTSHDN [illegible]
Smoellendorffii__XP_002970256.1    86   KNRRD-VEEKSEQLKAISNLAALFAGFAVVTLTQ-----FT------------------------IAPDPVLT-----------------VWIAFYGVL-TA--IAVGLM-TISMVTCTLILGSILKNGKSYVNEEAEEEFMFKCRDFVKSYRTGDKP [illegible]
Vcarteri__XP_002953952.1           81   RNRAE-VQEKAEQLKASALAALLAGFALVRYVEHRVRVLGTHAVHDPC-----------------ADDNRRYPKCSFLEFQFVVDANYNRALLPLF-ALT-TS--ITVGVE-VAAVVLCSLMLASIMRIGKMYVNEQEEAEFMHRCQIFITQYRP [illegible]
Tpseudonana__XP_002287927.1        38   DARRA-VDEKAQQLKVLANQSALVAGFSMVVLVE-----SN------------------------IPPDINGA-----------------LLTLF-GGV-TA--CVIALM-LVSTLNATYMLVAILRYDC [illegible]
Pinfestans__XP_002898394.1         62   SEHLV-CEARAEQLSTVSEQATLLCGFIVSAMCN-----VG------------------------VPDNVHAY-----------------VLFLY-TVSGTA--VCVSML-ICAVMCTNLQLAVTRYAAHTLEDSVRTMDMTQ [illegible]
Tgondii__XP_002366727.1            20   VQHME-VEYLVMVMNKITSLASLLSGFAASSFLQATG--LQ------------------------RGESWIKL-----------------IYVLF-TSS-A-----LGFM-LLVLLICTLCTMWGPGLAL [illegible]
Slemnae__CDW77297.1                20   IREKE-LNYWTTTFQTMASQSALLAG--------------------------------------------------------------NQILLFNL-----------ISSFCVMFGPGLALR [illegible]
Ehuxleyi__XP_005777770.1           20   LRQKE-LDYYVERYSNIATQASIVAGFAFDSLVE-----LE------------------------ISDDMRAALAPGGRWKAELRW----METVY-YGS-AS--LTMALA-LYTVCVASFATVYGHRLAL [illegible]
consensus/100%                          ............h...s........................................................................h..h......s......h.......s......... [illegible]
consensus/95%                           ............h...s.......................................s...................................l..h......s......h.h.......s.h.s.h... [illegible]
consensus/90%                           ........b....h...s..u.hhs........p......................s...............................lb.h..ss..ss..h.lsl..h.shhhssh.......h... [illegible]
consensus/85%                           ........b....h...s..u.lhs.h.......p.....h..................s.s............................lh.h..ss..ss..l.lsl..l.shhhssh.......h... [illegible]
consensus/80%                           .pb....h.b....l...u..uslhushuh..hhp.....lp.....................hs.s....s...................lh.hh.ssh.ss..l.lsl..l.slhlssh.......hs... [illegible]
consensus/75%                           .sbp..h.b....L...u..uulhuuhuh..hhp.....lp......................hs.s....s...................lh.hh.ssh.ss..lhlsl..lhslhlsshhh.....hsp.. [illegible]
consensus/70%                           hsbp..h.b...pLp..u..uulluuhuh.hhhp.....lp......................ls.s....s...................lh.hh.ssh.ss..lhlsl..lhslhlsshhh.....lsp.. [illegible]
```

VI. List of completely sequenced curated eukaryotic genomes used for the analysis.

TaxID	Species.name	Taxonomy
5855	Plasmodium vivax	Alveolata; Apicomplexa; Aconoidasida; Haemosporida; Plasmodiidae; Plasmodium; Plasmodium (Plasmodium)
73239	Plasmodium yoelii yoelii	Alveolata; Apicomplexa; Aconoidasida; Haemosporida; Plasmodiidae; Plasmodium; Plasmodium (Vinckeia); Plasmodium yoelii
36329	Plasmodium falciparum 3D7	Alveolata; Apicomplexa; Aconoidasida; Haemosporida; Plasmodiidae; Plasmodium; Plasmodium (Laverania); Plasmodium falciparum
5865	Babesia bovis	Alveolata; Apicomplexa; Aconoidasida; Piroplasmida; Babesiidae; Babesia
5866	Babesia bigemina	Alveolata; Apicomplexa; Aconoidasida; Piroplasmida; Babesiidae; Babesia
1133968	Babesia microti strain RI	Alveolata; Apicomplexa; Aconoidasida; Piroplasmida; Babesiidae; Babesia; Babesia microti
5875	Theileria parva	Alveolata; Apicomplexa; Aconoidasida; Piroplasmida; Theileriidae; Theileria
353154	Theileria annulata strain Ankara	Alveolata; Apicomplexa; Aconoidasida; Piroplasmida; Theileriidae; Theileria; Theileria annulata
1537102	Theileria equi strain WA	Alveolata; Apicomplexa; Aconoidasida; Piroplasmida; Theileriidae; Theileria; Theileria equi
869250	Theileria orientalis strain Shintoku	Alveolata; Apicomplexa; Aconoidasida; Piroplasmida; Theileriidae; Theileria; Theileria orientalis
353152	Cryptosporidium parvum Iowa II	Alveolata; Apicomplexa; Conoidasida; Coccidia; Eucoccidiorida; Eimeriorina; Cryptosporidiidae; Cryptosporidium; Cryptosporidium parvum
5802	Eimeria tenella	Alveolata; Apicomplexa; Conoidasida; Coccidia; Eucoccidiorida; Eimeriorina; Eimeriidae; Eimeria
99158	Hammondia hammondi	Alveolata; Apicomplexa; Conoidasida; Coccidia; Eucoccidiorida; Eimeriorina; Sarcocystidae; Hammondia
572307	Neospora caninum Liverpool	Alveolata; Apicomplexa; Conoidasida; Coccidia; Eucoccidiorida; Eimeriorina; Sarcocystidae; Neospora; Neospora caninum
508771	Toxoplasma gondii ME49	Alveolata; Apicomplexa; Conoidasida; Coccidia; Eucoccidiorida; Eimeriorina; Sarcocystidae; Toxoplasma; Toxoplasma gondii
441375	Cryptosporidium muris RN66	Alveolata; Apicomplexa; Conoidasida; Coccidia; Eucoccidiorida; Eimeriorina; Cryptosporidiidae; Cryptosporidium; Cryptosporidium muris
110365	Gregarina niphandrodes	Alveolata; Apicomplexa; Conoidasida; Gregarinasina; Eugregarinorida; Gregarinidae; Gregarina
5888	Paramecium tetraurelia	Alveolata; Ciliophora; Intramacronucleata; Oligohymenophorea; Peniculida; Parameciidae; Paramecium
5932	Ichthyophthirius multifiliis	Alveolata; Ciliophora; Intramacronucleata; Oligohymenophorea; Hymenostomatida; Ophryoglenina; Ichthyophthirius
312017	Tetrahymena thermophila SB210	Alveolata; Ciliophora; Intramacronucleata; Oligohymenophorea; Hymenostomatida; Tetrahymenina; Tetrahymenidae; Tetrahymena; Tetrahymena thermophila
423536	Perkinsus marinus ATCC 50983	Alveolata; Perkinsea; Perkinsida; Perkinsidae; Perkinsus; Perkinsus marinus
370355	Entamoeba invadens IP1	Amoebozoa; Archamoebae; Entamoebidae; Entamoeba; Entamoeba invadens
370354	Entamoeba dispar SAW760	Amoebozoa; Archamoebae; Entamoebidae; Entamoeba; Entamoeba dispar
1076696	Entamoeba nuttalli P19	Amoebozoa; Archamoebae; Entamoebidae; Entamoeba; Entamoeba nuttalli
294381	Entamoeba histolytica HM-1:IMSS	Amoebozoa; Archamoebae; Entamoebidae; Entamoeba; Entamoeba histolytica
1410327	Acytostelium subglobosum LB1	Amoebozoa; Mycetozoa; Dictyosteliida; Acytostelium; Acytostelium subglobosum
5786	Dictyostelium purpureum	Amoebozoa; Mycetozoa; Dictyosteliida; Dictyostelium
44689	Dictyostelium discoideum	Amoebozoa; Mycetozoa; Dictyosteliida; Dictyostelium
461836	Thecamonas trahens ATCC 50062	Apusozoa; Apusomonadidae; Thecamonas; Thecamonas trahens
2898	Cryptomonas paramecium	Cryptophyta; Cryptomonadales; Cryptomonadaceae; Cryptomonas
464988	Hemiselmis andersenii	Cryptophyta; Cryptomonadales; Hemiselmidaceae; Hemiselmis
905079	Guillardia theta CCMP2712	Cryptophyta; Pyrenomonadales; Geminigeraceae; Guillardia; Guillardia theta
55529	Guillardia theta	Cryptophyta; Pyrenomonadales; Geminigeraceae; Guillardia
157538	Leptomonas pyrrhocoris	Euglenozoa; Kinetoplastida; Trypanosomatidae; Leishmaniinae; Leptomonas
420245	Leishmania braziliensis MHOM/BR/75/M2904	Euglenozoa; Kinetoplastida; Trypanosomatidae; Leishmaniinae; Leishmania; Viannia; Leishmania braziliensis species complex; Leishmania braziliensis
71804	Trypanosoma grayi	Euglenozoa; Kinetoplastida; Trypanosomatidae; Trypanosoma; Trypanosoma with unspecified subgenus
185431	Trypanosoma brucei brucei TREU927	Euglenozoa; Kinetoplastida; Trypanosomatidae; Trypanosoma; Trypanozoon; Trypanosoma brucei; Trypanosoma brucei brucei
679716	Trypanosoma brucei gambiense DAL972	Euglenozoa; Kinetoplastida; Trypanosomatidae; Trypanosoma; Trypanozoon; Trypanosoma brucei; Trypanosoma brucei gambiense
435258	Leishmania infantum JPCM5	Euglenozoa; Kinetoplastida; Trypanosomatidae; Leishmaniinae; Leishmania; Leishmania; Leishmania donovani species complex; Leishmania infantum
5693	Trypanosoma cruzi	Euglenozoa; Kinetoplastida; Trypanosomatidae; Trypanosoma; Schizotrypanum
5679	Leishmania panamensis	Euglenozoa; Kinetoplastida; Trypanosomatidae; Leishmaniinae; Leishmania; Viannia; Leishmania guyanensis species complex
929439	Leishmania mexicana MHOM/GT/2001/U1103	Euglenozoa; Kinetoplastida; Trypanosomatidae; Leishmaniinae; Leishmania; Leishmania; Leishmania mexicana species complex; Leishmania mexicana
5661	Leishmania donovani	Euglenozoa; Kinetoplastida; Trypanosomatidae; Leishmaniinae; Leishmania; Leishmania; Leishmania donovani species complex
347515	Leishmania major strain Friedlin	Euglenozoa; Kinetoplastida; Trypanosomatidae; Leishmaniinae; Leishmania; Leishmania; Leishmania major species complex; Leishmania major
184922	Giardia lamblia ATCC 50803	Fornicata; Diplomonadida; Hexamitidae; Giardiinae; Giardia; Giardia intestinalis
280463	Emiliania huxleyi CCMP1516	Haptophyceae; Isochrysidales; Noelaerhabdaceae; Emiliania; Emiliania huxleyi
5762	Naegleria gruberi	Heterolobosea; Schizopyrenida; Vahlkampfiidae; Naegleria
431895	Monosiga brevicollis MX1	Opisthokonta; Choanoflagellida; Craspedida; Salpingoecidae; Monosiga; Monosiga brevicollis
946362	Salpingoeca rosetta	Opisthokonta; Choanoflagellida; Craspedida; Salpingoecidae; Salpingoeca
645134	Spizellomyces punctatus DAOM BR117	Opisthokonta; Fungi; Chytridiomycota; Chytridiomycetes; Spizellomycetales; Spizellomycetaceae; Spizellomyces; Spizellomyces punctatus
684364	Batrachochytrium dendrobatidis JAM81	Opisthokonta; Fungi; Chytridiomycota; Chytridiomycetes; Rhizophydiales; Rhizophydiales incertae sedis; Batrachochytrium; Batrachochytrium dendrobatidis
1328760	Xylona heveae TC161	Opisthokonta; Fungi; Dikarya; Ascomycota; saccharomyceta; Pezizomycotina; leotiomyceta; Xylonomycetes; Xylonomycetales; Xylonomycetaceae; Xylona; Xylona heveae
756982	Arthrobotrys oligospora ATCC 24927	Opisthokonta; Fungi; Dikarya; Ascomycota; saccharomyceta; Pezizomycotina; Orbiliomycetes; Orbiliales; Orbiliaceae; Arthrobotrys; Arthrobotrys oligospora
39416	Tuber melanosporum	Opisthokonta; Fungi; Dikarya; Ascomycota; saccharomyceta; Pezizomycotina; Pezizomycetes; Pezizales; Tuberaceae; Tuber
237561	Candida albicans SC5314	Opisthokonta; Fungi; Dikarya; Ascomycota; saccharomyceta; Saccharomycotina; Saccharomycetes; Saccharomycetales; Debaryomycetaceae; Candida/Lodderomyces clade; Candida; Candida albicans
367110	Neurospora crassa OR74A	Opisthokonta; Fungi; Dikarya; Ascomycota; saccharomyceta; Pezizomycotina; leotiomyceta; sordariomyceta; Sordariomycetes; Sordariomycetidae; Sordariales; Sordariaceae; Neurospora; Neurospora crassa
306902	Clavispora lusitaniae ATCC 42720	Opisthokonta; Fungi; Dikarya; Ascomycota; saccharomyceta; Saccharomycotina; Saccharomycetes; Saccharomycetales; Metschnikowiaceae; Clavispora; Clavispora lusitaniae
683960	Wickerhamomyces anomalus NRRL Y-366-8	Opisthokonta; Fungi; Dikarya; Ascomycota; saccharomyceta; Saccharomycotina; Saccharomycetes; Saccharomycetales; Phaffomycetaceae; Wickerhamomyces; Wickerhamomyces anomalus
763406	Pichia membranifaciens NRRL Y-2026	Opisthokonta; Fungi; Dikarya; Ascomycota; saccharomyceta; Saccharomycotina; Saccharomycetes; Saccharomycetales; Pichiaceae; Pichia; Pichia membranifaciens
559292	Saccharomyces cerevisiae S288c	Opisthokonta; Fungi; Dikarya; Ascomycota; saccharomyceta; Saccharomycotina; Saccharomycetes; Saccharomycetales; Saccharomycetaceae; Saccharomyces; Saccharomyces cerevisiae
796027	Sugiyamaella lignohabitans	Opisthokonta; Fungi; Dikarya; Ascomycota; saccharomyceta; Saccharomycotina; Saccharomycetes; Saccharomycetales; Trichomonascaceae; Sugiyamaella
28985	Kluyveromyces lactis	Opisthokonta; Fungi; Dikarya; Ascomycota; saccharomyceta; Saccharomycotina; Saccharomycetes; Saccharomycetales; Saccharomycetaceae; Kluyveromyces
1170230	Penicillium digitatum Pd1	Opisthokonta; Fungi; Dikarya; Ascomycota; saccharomyceta; Pezizomycotina; leotiomyceta; Eurotiomycetes; Eurotiomycetidae; Eurotiales; Aspergillaceae; Penicillium; Penicillium digitatum
306901	Chaetomium globosum CBS 148.51	Opisthokonta; Fungi; Dikarya; Ascomycota; saccharomyceta; Pezizomycotina; leotiomyceta; sordariomyceta; Sordariomycetes; Sordariomycetidae; Sordariales; Chaetomiaceae; Chaetomium; Chaetomium globosum
339724	Histoplasma capsulatum NAm1	Opisthokonta; Fungi; Dikarya; Ascomycota; saccharomyceta; Pezizomycotina; leotiomyceta; Eurotiomycetes; Eurotiomycetidae; Onygenales; Ajellomycetaceae; Histoplasma; Histoplasma capsulatum
1043004	Aureobasidium namibiae CBS 147.97	Opisthokonta; Fungi; Dikarya; Ascomycota; saccharomyceta; Pezizomycotina; leotiomyceta; dothideomyceta; Dothideomycetes; Dothideomycetidae; Dothideales; Aureobasidiaceae; Aureobasidium; Aureobasidium namibiae
284591	Yarrowia lipolytica CLIB122	Opisthokonta; Fungi; Dikarya; Ascomycota; saccharomyceta; Saccharomycotina; Saccharomycetes; Saccharomycetales; Dipodascaceae; Yarrowia; Yarrowia lipolytica
227321	Aspergillus nidulans FGSC A4	Opisthokonta; Fungi; Dikarya; Ascomycota; saccharomyceta; Pezizomycotina; leotiomyceta; Eurotiomycetes; Eurotiomycetidae; Eurotiales; Aspergillaceae; Aspergillus; Aspergillus nidulans
4896	Schizosaccharomyces pombe	Opisthokonta; Fungi; Dikarya; Ascomycota; Taphrinomycotina; Schizosaccharomycetes; Schizosaccharomycetales; Schizosaccharomycetaceae; Schizosaccharomyces
402676	Schizosaccharomyces japonicus yFS275	Opisthokonta; Fungi; Dikarya; Ascomycota; Taphrinomycotina; Schizosaccharomycetes; Schizosaccharomycetales; Schizosaccharomycetaceae; Schizosaccharomyces; Schizosaccharomyces japonicus
698492	Saitoella complicata NRRL Y-17804	Opisthokonta; Fungi; Dikarya; Ascomycota; Taphrinomycotina; Taphrinomycotina incertae sedis; Saitoella; Saitoella complicata
671144	Wallemia mellicola CBS 633.66	Opisthokonta; Fungi; Dikarya; Basidiomycota; Agaricomycotina; Wallemiomycetes; Wallemiales; Wallemiales incertae sedis; Wallemia; Wallemia mellicola
879819	Cutaneotrichosporon oleaginosus	Opisthokonta; Fungi; Dikarya; Basidiomycota; Agaricomycotina; Tremellomycetes; Trichosporonales; Trichosporonaceae; Cutaneotrichosporon
235443	Cryptococcus neoformans var. grubii H99	Opisthokonta; Fungi; Dikarya; Basidiomycota; Agaricomycotina; Tremellomycetes; Tremellales; Cryptococcaceae; Cryptococcus; Cryptococcus neoformans species complex; Cryptococcus neoformans; Cryptococcus neoformans var. grubii
597362	Agaricus bisporus var. burnettii JB137-S8	Opisthokonta; Fungi; Dikarya; Basidiomycota; Agaricomycotina; Agaricomycetes; Agaricomycetidae; Agaricales; Agaricaceae; Agaricus; Agaricus bisporus; Agaricus bisporus var. burnettii
240176	Coprinopsis cinerea okayama7#130	Opisthokonta; Fungi; Dikarya; Basidiomycota; Agaricomycotina; Agaricomycetes; Agaricomycetidae; Agaricales; Psathyrellaceae; Coprinopsis; Coprinopsis cinerea
4999	Kockovaella imperatae	Opisthokonta; Fungi; Dikarya; Basidiomycota; Agaricomycotina; Tremellomycetes; Tremellales; Cuniculitremaceae; Kockovaella
936046	Agaricus bisporus var. bisporus H97	Opisthokonta; Fungi; Dikarya; Basidiomycota; Agaricomycotina; Agaricomycetes; Agaricomycetidae; Agaricales; Agaricaceae; Agaricus; Agaricus bisporus; Agaricus bisporus var. bisporus
486041	Laccaria bicolor S238N-H82	Opisthokonta; Fungi; Dikarya; Basidiomycota; Agaricomycotina; Agaricomycetes; Agaricomycetidae; Agaricales; Tricholomataceae; Laccaria; Laccaria bicolor
561896	Postia placenta Mad-698-R	Opisthokonta; Fungi; Dikarya; Basidiomycota; Agaricomycotina; Agaricomycetes; Agaricomycetes incertae sedis; Polyporales; Coriolaceae; Postia; Postia placenta
764103	Mixia osmundae IAM 14324	Opisthokonta; Fungi; Dikarya; Basidiomycota; Pucciniomycotina; Mixiomycetes; Mixiales; Mixiaceae; Mixia; Mixia osmundae
578459	Rhodotorula graminis WP1	Opisthokonta; Fungi; Dikarya; Basidiomycota; Pucciniomycotina; Microbotryomycetes; Sporidiobolales; Sporidiobolaceae; Rhodotorula; Rhodotorula graminis
418459	Puccinia graminis f. sp. tritici CRL 75-36-700-3	Opisthokonta; Fungi; Dikarya; Basidiomycota; Pucciniomycotina; Pucciniomycetes; Pucciniales; Pucciniaceae; Puccinia; Puccinia graminis; Puccinia graminis f. sp. tritici
77020	Malassezia pachydermatis	Opisthokonta; Fungi; Dikarya; Basidiomycota; Ustilaginomycotina; Malasseziomycetes; Malasseziales; Malasseziaceae; Malassezia
237631	Ustilago maydis 521	Opisthokonta; Fungi; Dikarya; Basidiomycota; Ustilaginomycotina; Ustilaginomycetes; Ustilaginales; Ustilaginaceae; Ustilago; Ustilago maydis
1037660	Tilletiaria anomala UBC 951	Opisthokonta; Fungi; Dikarya; Basidiomycota; Ustilaginomycotina; Exobasidiomycetes; Georgefischeriales; Tilletiariaceae; Tilletiaria; Tilletiaria anomala
284813	Encephalitozoon cuniculi GB-M1	Opisthokonta; Fungi; Microsporidia; Apansporoblastina; Unikaryonidae; Encephalitozoon; Encephalitozoon cuniculi
578460	Nosema ceranae BRL01	Opisthokonta; Fungi; Microsporidia; Apansporoblastina; Nosematidae; Nosema; Nosema ceranae
481877	Enterocytozoon bieneusi H348	Opisthokonta; Fungi; Microsporidia; Apansporoblastina; Enterocytozoonidae; Enterocytozoon; Enterocytozoon bieneusi
993615	Vittaforma corneae ATCC 50505	Opisthokonta; Fungi; Microsporidia; Apansporoblastina; Nosematidae; Vittaforma; Vittaforma corneae
1485682	Mitosporidium daphniae	Opisthokonta; Fungi; Microsporidia; Microsporidia incertae sedis; Mitosporidium
881290	Nematocida parisii ERTm1	Opisthokonta; Fungi; Microsporidia; Microsporidia incertae sedis; Nematocida; Nematocida parisii
1354746	Ordospora colligata OC4	Opisthokonta; Fungi; Microsporidia; Microsporidia incertae sedis; Ordosporidae; Ordospora; Ordospora colligata
948595	Vavraia culicis subsp. floridensis	Opisthokonta; Fungi; Microsporidia; Pansporoblastina; Pleistophoridae; Vavraia; Vavraia culicis
64571	Lobosporangium transversale	Opisthokonta; Fungi; Mucoromycota; Mortierellomycotina; Mortierellales; Mortierellaceae; Lobosporangium
763407	Phycomyces blakesleeanus NRRL 1555(-)	Opisthokonta; Fungi; Mucoromycota; Mucoromycotina; Mucorales; Phycomycetaceae; Phycomyces; Phycomyces blakesleeanus
7955	Danio rerio	Opisthokonta; Metazoa; Eumetazoa; Bilateria; Deuterostomia; Chordata; Craniata; Vertebrata; Gnathostomata; Teleostomi; Euteleostomi; Actinopterygii; Actinopteri; Neopterygii; Teleostei; Osteoglossocephalai; Clupeocephala; Otomorpha; Ostariophysi; Otophysi; Cypriniphysae; Cypriniformes; Cyprinoidea; Cyprinidae; Danio
7719	Ciona intestinalis	Opisthokonta; Metazoa; Eumetazoa; Bilateria; Deuterostomia; Chordata; Tunicata; Ascidiacea; Enterogona; Phlebobranchia; Cionidae; Ciona
7668	Strongylocentrotus purpuratus	Opisthokonta; Metazoa; Eumetazoa; Bilateria; Deuterostomia; Echinodermata; Eleutherozoa; Echinozoa; Echinoidea; Euechinoidea; Echinacea; Echinoida; Strongylocentrotidae; Strongylocentrotus
7998	Ictalurus punctatus	Opisthokonta; Metazoa; Eumetazoa; Bilateria; Deuterostomia; Chordata; Craniata; Vertebrata; Gnathostomata; Teleostomi; Euteleostomi; Actinopterygii; Actinopteri; Neopterygii; Teleostei; Osteoglossocephalai; Clupeocephala; Otomorpha; Ostariophysi; Otophysi; Characiphysae; Siluriformes; Siluroidei; Ictaluridae; Ictalurus
9031	Gallus gallus	Opisthokonta; Metazoa; Eumetazoa; Bilateria; Deuterostomia; Chordata; Craniata; Vertebrata; Gnathostomata; Teleostomi; Euteleostomi; Sarcopterygii; Dipnotetrapodomorpha; Tetrapoda; Amniota; Sauropsida; Sauria; Archelosauria; Archosauria; Dinosauria; Saurischia; Theropoda; Coelurosauria; Aves; Neognathae; Galloanserae; Galliformes; Phasianidae; Phasianinae; Gallus
61853	Nomascus leucogenys	Opisthokonta; Metazoa; Eumetazoa; Bilateria; Deuterostomia; Chordata; Craniata; Vertebrata; Gnathostomata; Teleostomi; Euteleostomi; Sarcopterygii; Dipnotetrapodomorpha; Tetrapoda; Amniota; Mammalia; Theria; Eutheria; Boreoeutheria; Euarchontoglires; Primates; Haplorrhini; Simiiformes; Catarrhini; Hominoidea; Hylobatidae; Nomascus
10090	Mus musculus	Opisthokonta; Metazoa; Eumetazoa; Bilateria; Deuterostomia; Chordata; Craniata; Vertebrata; Gnathostomata; Teleostomi; Euteleostomi; Sarcopterygii; Dipnotetrapodomorpha; Tetrapoda; Amniota; Mammalia; Theria; Eutheria; Boreoeutheria; Euarchontoglires; Glires; Rodentia; Sciurognathi; Muroidea; Muridae; Murinae; Mus; Mus
9541	Macaca fascicularis	Opisthokonta; Metazoa; Eumetazoa; Bilateria; Deuterostomia; Chordata; Craniata; Vertebrata; Gnathostomata; Teleostomi; Euteleostomi; Sarcopterygii; Dipnotetrapodomorpha; Tetrapoda; Amniota; Mammalia; Theria; Eutheria; Boreoeutheria; Euarchontoglires; Primates; Haplorrhini; Simiiformes; Catarrhini; Cercopithecoidea; Cercopithecidae; Cercopithecinae; Macaca
10224	Saccoglossus kowalevskii	Opisthokonta; Metazoa; Eumetazoa; Bilateria; Deuterostomia; Hemichordata; Enteropneusta; Harrimaniidae; Saccoglossus
9606	Homo sapiens	Opisthokonta; Metazoa; Eumetazoa; Bilateria; Deuterostomia; Chordata; Craniata; Vertebrata; Gnathostomata; Teleostomi; Euteleostomi; Sarcopterygii; Dipnotetrapodomorpha; Tetrapoda; Amniota; Mammalia; Theria; Eutheria; Boreoeutheria; Euarchontoglires; Primates; Haplorrhini; Simiiformes; Catarrhini; Hominoidea; Hominidae; Homininae; Homo
7739	Branchiostoma floridae	Opisthokonta; Metazoa; Eumetazoa; Bilateria; Deuterostomia; Chordata; Cephalochordata; Branchiostomidae; Branchiostoma
8355	Xenopus laevis	Opisthokonta; Metazoa; Eumetazoa; Bilateria; Deuterostomia; Chordata; Craniata; Vertebrata; Gnathostomata; Teleostomi; Euteleostomi; Sarcopterygii; Dipnotetrapodomorpha; Tetrapoda; Amphibia; Batrachia; Anura; Pipoidea; Pipidae; Xenopodinae; Xenopus; Xenopus
31033	Takifugu rubripes	Opisthokonta; Metazoa; Eumetazoa; Bilateria; Deuterostomia; Chordata; Craniata; Vertebrata; Gnathostomata; Teleostomi; Euteleostomi; Actinopterygii; Actinopteri; Neopterygii; Teleostei; Osteoglossocephalai; Clupeocephala; Euteleosteomorpha; Neoteleostei; Eurypterygia; Ctenosquamata; Acanthomorphata; Euacanthomorphacea; Percomorphaceae; Eupercaria; Tetraodontiformes; Tetraodontoidei; Tetradontoidea; Tetraodontidae; Takifugu
7070	Tribolium castaneum	Opisthokonta; Metazoa; Eumetazoa; Bilateria; Protostomia; Ecdysozoa; Panarthropoda; Arthropoda; Mandibulata; Pancrustacea; Hexapoda; Insecta; Dicondylia; Pterygota; Neoptera; Endopterygota; Coleoptera; Polyphaga; Cucujiformia; Tenebrionoidea; Tenebrionidae; Tenebrionidae incertae sedis; Tribolium
7091	Bombyx mori	Opisthokonta; Metazoa; Eumetazoa; Bilateria; Protostomia; Ecdysozoa; Panarthropoda; Arthropoda; Mandibulata; Pancrustacea; Hexapoda; Insecta; Dicondylia; Pterygota; Neoptera; Endopterygota; Amphiesmenoptera; Lepidoptera; Glossata; Neolepidoptera; Heteroneura; Ditrysia; Obtectomera; Bombycoidea; Bombycidae; Bombycinae; Bombyx
7227	Drosophila melanogaster	Opisthokonta; Metazoa; Eumetazoa; Bilateria; Protostomia; Ecdysozoa; Panarthropoda; Arthropoda; Mandibulata; Pancrustacea; Hexapoda; Insecta; Dicondylia; Pterygota; Neoptera; Endopterygota; Diptera; Brachycera; Muscomorpha; Eremoneura; Cyclorrhapha; Schizophora; Acalyptratae; Ephydroidea; Drosophilidae; Drosophilinae; Drosophilini; Drosophila; Sophophora; melanogaster group; melanogaster subgroup
6850	Limulus polyphemus	Opisthokonta; Metazoa; Eumetazoa; Bilateria; Protostomia; Ecdysozoa; Panarthropoda; Arthropoda; Chelicerata; Merostomata; Xiphosura; Limulidae; Limulus
37621	Priapulus caudatus	Opisthokonta; Metazoa; Eumetazoa; Bilateria; Protostomia; Ecdysozoa; Scalidophora; Priapulida; Priapulidae; Priapulus
6412	Helobdella robusta	Opisthokonta; Metazoa; Eumetazoa; Bilateria; Protostomia; Lophotrochozoa; Annelida; Clitellata; Hirudinida; Hirudinea; Rhynchobdellida; Glossiphoniidae; Helobdella
7574	Lingula anatina	Opisthokonta; Metazoa; Eumetazoa; Bilateria; Protostomia; Lophotrochozoa; Brachiopoda; Linguliformea; Lingulata; Lingulida; Linguloidea; Lingulidae; Lingula
29159	Crassostrea gigas	Opisthokonta; Metazoa; Eumetazoa; Bilateria; Protostomia; Lophotrochozoa; Mollusca; Bivalvia; Pteriomorphia; Ostreoida; Ostreoidea; Ostreidae; Crassostrea
37653	Octopus bimaculoides	Opisthokonta; Metazoa; Eumetazoa; Bilateria; Protostomia; Lophotrochozoa; Mollusca; Cephalopoda; Coleoidea; Neocoleoidea; Octopodiformes; Octopoda; Incirrata; Octopodidae; Octopus
225164	Lottia gigantea	Opisthokonta; Metazoa; Eumetazoa; Bilateria; Protostomia; Lophotrochozoa; Mollusca; Gastropoda; Patellogastropoda; Lottioidea; Lottiidae; Lottia
7176	Culex quinquefasciatus	Opisthokonta; Metazoa; Eumetazoa; Bilateria; Protostomia; Ecdysozoa; Panarthropoda; Arthropoda; Mandibulata; Pancrustacea; Hexapoda; Insecta; Dicondylia; Pterygota; Neoptera; Endopterygota; Diptera; Nematocera; Culicomorpha; Culicoidea; Culicidae; Culicinae; Culicini; Culex; Culex; Culex pipiens complex
7460	Apis mellifera	Opisthokonta; Metazoa; Eumetazoa; Bilateria; Protostomia; Ecdysozoa; Panarthropoda; Arthropoda; Mandibulata; Pancrustacea; Hexapoda; Insecta; Dicondylia; Pterygota; Neoptera; Endopterygota; Hymenoptera; Apocrita; Aculeata; Apoidea; Apidae; Apinae; Apini; Apis
7029	Acyrthosiphon pisum	Opisthokonta; Metazoa; Eumetazoa; Bilateria; Protostomia; Ecdysozoa; Panarthropoda; Arthropoda; Mandibulata; Pancrustacea; Hexapoda; Insecta; Dicondylia; Pterygota; Neoptera; Paraneoptera; Hemiptera; Sternorrhyncha; Aphidiformes; Aphidomorpha; Aphidoidea; Aphididae; Aphidinae; Macrosiphini; Acyrthosiphon
6334	Trichinella spiralis	Opisthokonta; Metazoa; Eumetazoa; Bilateria; Protostomia; Ecdysozoa; Nematoda; Enoplea; Dorylaimia; Trichocephalida; Trichinellidae; Trichinella
6279	Brugia malayi	Opisthokonta; Metazoa; Eumetazoa; Bilateria; Protostomia; Ecdysozoa; Nematoda; Chromadorea; Spirurida; Filarioidea; Onchocercidae; Brugia
610380	Harpegnathos saltator	Opisthokonta; Metazoa; Eumetazoa; Bilateria; Protostomia; Ecdysozoa; Panarthropoda; Arthropoda; Mandibulata; Pancrustacea; Hexapoda; Insecta; Dicondylia; Pterygota; Neoptera; Holometabola; Hymenoptera; Apocrita; Aculeata; Vespoidea; Formicidae; Ponerinae; Ponerini; Harpegnathos
6239	Caenorhabditis elegans	Opisthokonta; Metazoa; Eumetazoa; Bilateria; Protostomia; Ecdysozoa; Nematoda; Chromadorea; Rhabditida; Rhabditoidea; Rhabditidae; Peloderinae; Caenorhabditis
6183	Schistosoma mansoni	Opisthokonta; Metazoa; Eumetazoa; Bilateria; Platyhelminthes; Trematoda; Digenea; Strigeidida; Schistosomatoidea; Schistosomatidae; Schistosoma
6945	Ixodes scapularis	Opisthokonta; Metazoa; Eumetazoa; Bilateria; Protostomia; Ecdysozoa; Panarthropoda; Arthropoda; Chelicerata; Arachnida; Acari; Parasitiformes; Ixodida; Ixodoidea; Ixodidae; Ixodinae; Ixodes
6198	Opisthorchis viverrini	Opisthokonta; Metazoa; Eumetazoa; Bilateria; Platyhelminthes; Trematoda; Digenea; Opisthorchiida; Opisthorchiata; Opisthorchiidae; Opisthorchis
45351	Nematostella vectensis	Opisthokonta; Metazoa; Eumetazoa; Cnidaria; Anthozoa; Hexacorallia; Actiniaria; Edwardsiidae; Nematostella
70779	Acropora digitifera	Opisthokonta; Metazoa; Eumetazoa; Cnidaria; Anthozoa; Hexacorallia; Scleractinia; Astrocoeniina; Acroporidae; Acropora
6087	Hydra vulgaris	Opisthokonta; Metazoa; Eumetazoa; Cnidaria; Hydrozoa; Hydroidolina; Anthoathecata; Aplanulata; Hydridae; Hydra
10228	Trichoplax adhaerens	Opisthokonta; Metazoa; Placozoa; Trichoplax
400682	Amphimedon queenslandica	Opisthokonta; Metazoa; Porifera; Demospongiae; Heteroscleromorpha; Haplosclerida; Niphatidae; Amphimedon
691883	Fonticula alba	Opisthokonta; Nucleariidae and Fonticula group; Fonticula
595528	Capsaspora owczarzaki ATCC 30864	Opisthokonta; Opisthokonta incertae sedis; Ichthyosporea; Capsaspora; Capsaspora owczarzaki
667725	Sphaeroforma arctica JP610	Opisthokonta; Opisthokonta incertae sedis; Ichthyosporea; Ichthyophonida; Sphaeroforma; Sphaeroforma arctica
412133	Trichomonas vaginalis G3	Parabasalia; Trichomonadida; Trichomonadidae; Trichomonas; Trichomonas vaginalis
227086	Bigelowiella natans	Rhizaria; Cercozoa; Chlorarachniophyceae; Bigelowiella
280699	Cyanidioschyzon merolae strain 10D	Rhodophyta; Bangiophyceae; Cyanidiales; Cyanidiaceae; Cyanidioschyzon; Cyanidioschyzon merolae
130081	Galdieria sulphuraria	Rhodophyta; Bangiophyceae; Cyanidiales; Cyanidiaceae; Galdieria
2769	Chondrus crispus	Rhodophyta; Florideophyceae; Gigartinales; Gigartinaceae; Chondrus
556484	Phaeodactylum tricornutum CCAP 1055/1	Stramenopiles; Bacillariophyta; Bacillariophyceae; Bacillariophycidae; Naviculales; Phaeodactylaceae; Phaeodactylum; Phaeodactylum tricornutum
296543	Thalassiosira pseudonana CCMP1335	Stramenopiles; Bacillariophyta; Coscinodiscophyceae; Thalassiosirophycidae; Thalassiosirales; Thalassiosiraceae; Thalassiosira; Thalassiosira pseudonana
12968	Blastocystis hominis	Stramenopiles; Blastocystis
944170	Blastocystis sp. subtype 4	Stramenopiles; Blastocystis; Blastocystis sp. subtypes
67593	Phytophthora sojae	Stramenopiles; Oomycetes; Peronosporales; Phytophthora
403677	Phytophthora infestans T30-4	Stramenopiles; Oomycetes; Peronosporales; Phytophthora; Phytophthora infestans
761204	Phytophthora parasitica INRA-310	Stramenopiles; Oomycetes; Peronosporales; Phytophthora; Phytophthora parasitica
695850	Saprolegnia parasitica CBS 223.65	Stramenopiles; Oomycetes; Saprolegniales; Saprolegniaceae; Saprolegnia; Saprolegnia parasitica
157072	Aphanomyces invadans	Stramenopiles; Oomycetes; Saprolegniales; Saprolegniaceae; Aphanomyces
112090	Aphanomyces astaci	Stramenopiles; Oomycetes; Saprolegniales; Saprolegniaceae; Aphanomyces
1156394	Saprolegnia diclina VS20	Stramenopiles; Oomycetes; Saprolegniales; Saprolegniaceae; Saprolegnia; Saprolegnia diclina
44056	Aureococcus anophagefferens	Stramenopiles; Pelagophyceae; Pelagomonadales; Aureococcus
3055	Chlamydomonas reinhardtii	Viridiplantae; Chlorophyta; Chlorophyceae; Chlamydomonadales; Chlamydomonadaceae; Chlamydomonas
3068	Volvox carteri f. nagariensis	Viridiplantae; Chlorophyta; Chlorophyceae; Chlamydomonadales; Volvocaceae; Volvox; Volvox carteri
145388	Monoraphidium neglectum	Viridiplantae; Chlorophyta; Chlorophyceae; Sphaeropleales; Selenastraceae; Monoraphidium
70448	Ostreococcus tauri	Viridiplantae; Chlorophyta; prasinophytes; Mamiellophyceae; Mamiellales; Bathycoccaceae; Ostreococcus
564608	Micromonas pusilla CCMP1545	Viridiplantae; Chlorophyta; prasinophytes; Mamiellophyceae; Mamiellales; Mamiellaceae; Micromonas; Micromonas pusilla
296587	Micromonas commoda	Viridiplantae; Chlorophyta; prasinophytes; Mamiellophyceae; Mamiellales; Mamiellaceae; Micromonas
554065	Chlorella variabilis	Viridiplantae; Chlorophyta; Trebouxiophyceae; Chlorellales; Chlorellaceae; Chlorella
574566	Coccomyxa subellipsoidea C-169	Viridiplantae; Chlorophyta; Trebouxiophyceae; Trebouxiophyceae incertae sedis; Coccomyxaceae; Coccomyxa; Coccomyxa subellipsoidea
3075	Auxenochlorella protothecoides	Viridiplantae; Chlorophyta; Trebouxiophyceae; Chlorellales; Chlorellaceae; Auxenochlorella
3702	Arabidopsis thaliana	Viridiplantae; Streptophyta; Streptophytina; Embryophyta; Tracheophyta; Euphyllophyta; Spermatophyta; Magnoliophyta; Mesangiospermae; eudicotyledons; Gunneridae; Pentapetalae; rosids; malvids; Brassicales; Brassicaceae; Camelineae; Arabidopsis
29760	Vitis vinifera	Viridiplantae; Streptophyta; Streptophytina; Embryophyta; Tracheophyta; Euphyllophyta; Spermatophyta; Magnoliophyta; Mesangiospermae; eudicotyledons; Gunneridae; Pentapetalae; rosids; rosids incertae sedis; Vitales; Vitaceae; Vitis
4577	Zea mays	Viridiplantae; Streptophyta; Streptophytina; Embryophyta; Tracheophyta; Euphyllophyta; Spermatophyta; Magnoliophyta; Mesangiospermae; Liliopsida; Petrosaviidae; commelinids; Poales; Poaceae; PACMAD clade; Panicoideae; Andropogonodae; Andropogoneae; Tripsacinae; Zea
4097	Nicotiana tabacum	Viridiplantae; Streptophyta; Streptophytina; Embryophyta; Tracheophyta; Euphyllophyta; Spermatophyta; Magnoliophyta; Mesangiospermae; eudicotyledons; Gunneridae; Pentapetalae; asterids; lamiids; Solanales; Solanaceae; Nicotianoideae; Nicotianeae; Nicotiana
3847	Glycine max	Viridiplantae; Streptophyta; Streptophytina; Embryophyta; Tracheophyta; Euphyllophyta; Spermatophyta; Magnoliophyta; Mesangiospermae; eudicotyledons; Gunneridae; Pentapetalae; rosids; fabids; Fabales; Fabaceae; Papilionoideae; Phaseoleae; Glycine; Soja
3218	Physcomitrella patens	Viridiplantae; Streptophyta; Streptophytina; Embryophyta; Bryophyta; Bryophytina; Bryopsida; Funariidae; Funariales; Funariaceae; Physcomitrella
88036	Selaginella moellendorffii	Viridiplantae; Streptophyta; Streptophytina; Embryophyta; Tracheophyta; Lycopodiidae; Selaginellales; Selaginellaceae; Selaginella
4113	Solanum tuberosum	Viridiplantae; Streptophyta; Streptophytina; Embryophyta; Tracheophyta; Euphyllophyta; Spermatophyta; Magnoliophyta; Mesangiospermae; eudicotyledons; Gunneridae; Pentapetalae; asterids; lamiids; Solanales; Solanaceae; Solanoideae; Solaneae; Solanum
1169540	Vitrella brassicaformis CCMP3155	Alveolata; Chromerida; Vitrella; Vitrella brassicaformis
47911	Entodinium caudatum	Alveolata; Ciliophora; Intramacronucleata; Litostomatea; Trichostomatia; Entodiniomorphida; Ophryoscolecidae; Entodinium
5949	Stylonychia lemnae	Alveolata; Ciliophora; Intramacronucleata; Spirotrichea; Stichotrichia; Sporadotrichida; Oxytrichidae; Stylonychinae; Stylonychia
1172189	Oxytricha trifallax	Alveolata; Ciliophora; Intramacronucleata; Spirotrichea; Stichotrichia; Sporadotrichida; Oxytrichidae; Oxytrichinae; Oxytricha
5963	Stentor coeruleus	Alveolata; Ciliophora; Postciliodesmatophora; Heterotrichea; Heterotrichida; Stentoridae; Stentor
2951	Symbiodinium microadriaticum	Alveolata; Dinophyceae; Suessiales; Symbiodiniaceae; Symbiodinium
5759	Entamoeba histolytica	Amoebozoa; Archamoebae; Entamoebidae; Entamoeba
180228	Paramoeba pemaquidensis	Amoebozoa; Discosea; Flabellinia; Dactylopodida; Paramoebidae; Paramoeba
65659	Acanthamoeba comandoni	Amoebozoa; Discosea; Longamoebia; Centramoebida; Acanthamoebidae; Acanthamoeba
361140	Acytostelium ellipticum	Amoebozoa; Mycetozoa; Dictyosteliida; Acytostelium
133408	Acytostelium leptosomum	Amoebozoa; Mycetozoa; Dictyosteliida; Acytostelium
670386	Polysphondylium pallidum PN500	Amoebozoa; Mycetozoa; Dictyosteliida; Polysphondylium; Polysphondylium pallidum
472931	Protostelium mycophagum	Amoebozoa; Mycetozoa; Protosteliales; Protosteliaceae; Protostelium
1195612	Chroomonas mesostigmatica CCMP1168	Cryptophyta; Pyrenomonadales; Chroomonadaceae; Chroomonas; Chroomonas mesostigmatica
5684	Leptomonas seymouri	Euglenozoa; Kinetoplastida; Trypanosomatidae; Leishmaniinae; Leptomonas
134006	Phytomonas sp. isolate EM1	Euglenozoa; Kinetoplastida; Trypanosomatidae; Phytomonas; unclassified Phytomonas
134013	Phytomonas sp. isolate Hart1	Euglenozoa; Kinetoplastida; Trypanosomatidae; Phytomonas; unclassified Phytomonas
59799	Angomonas deanei	Euglenozoa; Kinetoplastida; Trypanosomatidae; Strigomonadinae; Angomonas
28005	Strigomonas culicis	Euglenozoa; Kinetoplastida; Trypanosomatidae; Strigomonadinae; Strigomonas
598745	Giardia intestinalis ATCC 50581	Fornicata; Diplomonadida; Hexamitidae; Giardiinae; Giardia; Giardia intestinalis
5741	Giardia intestinalis	Fornicata; Diplomonadida; Hexamitidae; Giardiinae; Giardia
348837	Spironucleus salmonicida	Fornicata; Diplomonadida; Hexamitidae; Hexamitinae; Spironucleus
765915	Catenaria anguillulae PL171	Opisthokonta; Fungi; Blastocladiomycota; Blastocladiomycetes; Blastocladiales; Catenariaceae; Catenaria; Catenaria anguillulae
578462	Allomyces macrogynus ATCC 38327	Opisthokonta; Fungi; Blastocladiomycota; Blastocladiomycetes; Blastocladiales; Blastocladiaceae; Allomyces; Allomyces macrogynus
988480	Rozella allomycis CSF55	Opisthokonta; Fungi; Cryptomycota; Rozella; Rozella allomycis
743788	Fomitopsis pinicola FP-58527 SS1	Opisthokonta; Fungi; Dikarya; Basidiomycota; Agaricomycotina; Agaricomycetes; Agaricomycetes incertae sedis; Polyporales; Fomitopsidaceae; Fomitopsis; Fomitopsis pinicola
1069443	Mortierella verticillata NRRL 6337	Opisthokonta; Fungi; Mucoromycota; Mortierellomycotina; Mortierellales; Mortierellaceae; Mortierella; Mortierella verticillata
64571	Lobosporangium transversale	Opisthokonta; Fungi; Mucoromycota; Mortierellomycotina; Mortierellales; Mortierellaceae; Lobosporangium
747725	Mucor circinelloides f. lusitanicus CBS 277.49	Opisthokonta; Fungi; Mucoromycota; Mucoromycotina; Mucorales; Mucorineae; Mucoraceae; Mucor; Mucor circinelloides; Mucor circinelloides f. lusitanicus
246409	Rhizopus delemar RA 99-880	Opisthokonta; Fungi; Mucoromycota; Mucoromycotina; Mucorales; Mucorineae; Rhizopodaceae; Rhizopus; Rhizopus delemar
1938954	Bifiguratus adelaidae	Opisthokonta; Fungi; Mucoromycota; Mucoromycotina; Mucoromycotina incertae sedis; Bifiguratus
1220926	Mucor circinelloides f. circinelloides 1006PhL	Opisthokonta; Fungi; Mucoromycota; Mucoromycotina; Mucorales; Mucorineae; Mucoraceae; Mucor; Mucor circinelloides; Mucor circinelloides f. circinelloides
86635	Rhizopus microsporus var microsporus	Opisthokonta; Fungi; Mucoromycota; Mucoromycotina; Mucorales; Mucorineae; Rhizopodaceae; Rhizopus; Rhizopus microsporus
1754190	Neocallimastix californiae	Opisthokonta; Fungi; Neocallimastigomycota; Neocallimastigomycetes; Neocallimastigales; Neocallimastigaceae; Neocallimastix
1987568	Pecoramyces ruminatium	Opisthokonta; Fungi; Neocallimastigomycota; Neocallimastigomycetes; Neocallimastigales; Neocallimastigaceae; Pecoramyces
